# Integrated microbiome-metabolome-genome axis data of Laiwu and Lulai pigs

**DOI:** 10.1038/s41597-023-02191-2

**Published:** 2023-05-13

**Authors:** Xueshuang Lai, Zhenyang Zhang, Zhe Zhang, Shengqiang Liu, Chunyan Bai, Zitao Chen, Qamar Raza Qadri, Yifei Fang, Zhen Wang, Yuchun Pan, Qishan Wang

**Affiliations:** 1 Department of Animal Science, School of Agriculture and Biology, Shanghai Jiao Tong University, Shanghai, 200240 PR China; 2 Department of Animal Science, College of Animal Sciences, Zhejiang University, Hangzhou, 310030 PR China; 3 Hainan institute, Zhejiang University, Sanya, 310014 PR China; 4 Department of Animal Science, College of Animal Sciences, Jilin University, Changchui, 130015 PR China

**Keywords:** Metagenomics, Metabolomics

## Abstract

Excessive fat deposition can trigger metabolic diseases, and it is crucial to identify factors that can break the link between fat deposition and metabolic diseases. Healthy obese *Laiwu pigs* (LW) are high in fat content but resistant to metabolic diseases. In this study, we compared the fecal microbiome, fecal and blood metabolome, and genome of LW and *Lulai pigs* (LU) to identify factors that can block the link between fat deposition and metabolic diseases. Our results show significant differences in Spirochetes and *Treponema*, which are involved in carbohydrate metabolism, between LW and LU. The fecal and blood metabolome composition was similar, and some anti-metabolic disease components of blood metabolites were different between the two breeds of pigs. The predicted differential RNA is mainly enriched in lipid metabolism and glucose metabolism, which is consistent with the functions of differential microbiota and metabolites. The down-regulated gene *RGP1* is strongly negatively correlated with *Treponema*. Our omics data would provide valuable resources for further scientific research on healthy obesity in both human and porcine.

## Background & Summary

Excessive fat deposition can lead to chronic damage to organs and metabolic diseases^1–3^. However, genetic factors alone cannot fully explain these conditions^4^. The role of metabolic factors, such as gut microbiota and metabolites^5–8^, has gained increasing attention in understanding the causes of obesity-induced chronic metabolic diseases^9–11^. Changes in gut microbiota composition have been shown to trigger chronic metabolic diseases, including hypertension, atherosclerosis, and type 2 diabetes mellitus (T2DM)^12–14^. Microbiota produce essential metabolites such as trimethylamine N-oxide (TMAO) is directly linked with chronic metabolic diseases, such as atherosclerosis, T2DM, cardiovascular diseases (CVD) and stroke^15–18^. Moreover, Gut microbiota can ferment unabsorbed/undigested carbohydrates to produce aliphatic organic acids like short chain fatty acids (SCFAs)^19,20^. SCFAs can protect the host from diet-induced obesity through G protein-coupled receptors, and microbiota indirectly regulate host lipid metabolism through SCFAs^21–23^. Thus, gut microbes act as an endocrine organ, producing bioactive metabolites that affect host physiology^7,24–26^. Conversely, recent studies have shown that host genome can influence related phenotypes by altering the gut microbiota. For example, ABO genotypes can influence the gut microbiota structure by regulating N-acetylgalactosamine (GalNAc)^27^. Therefore, integrating omics analysis may help to identify key factors that protect individuals against metabolic diseases.

**Fig. 1:**
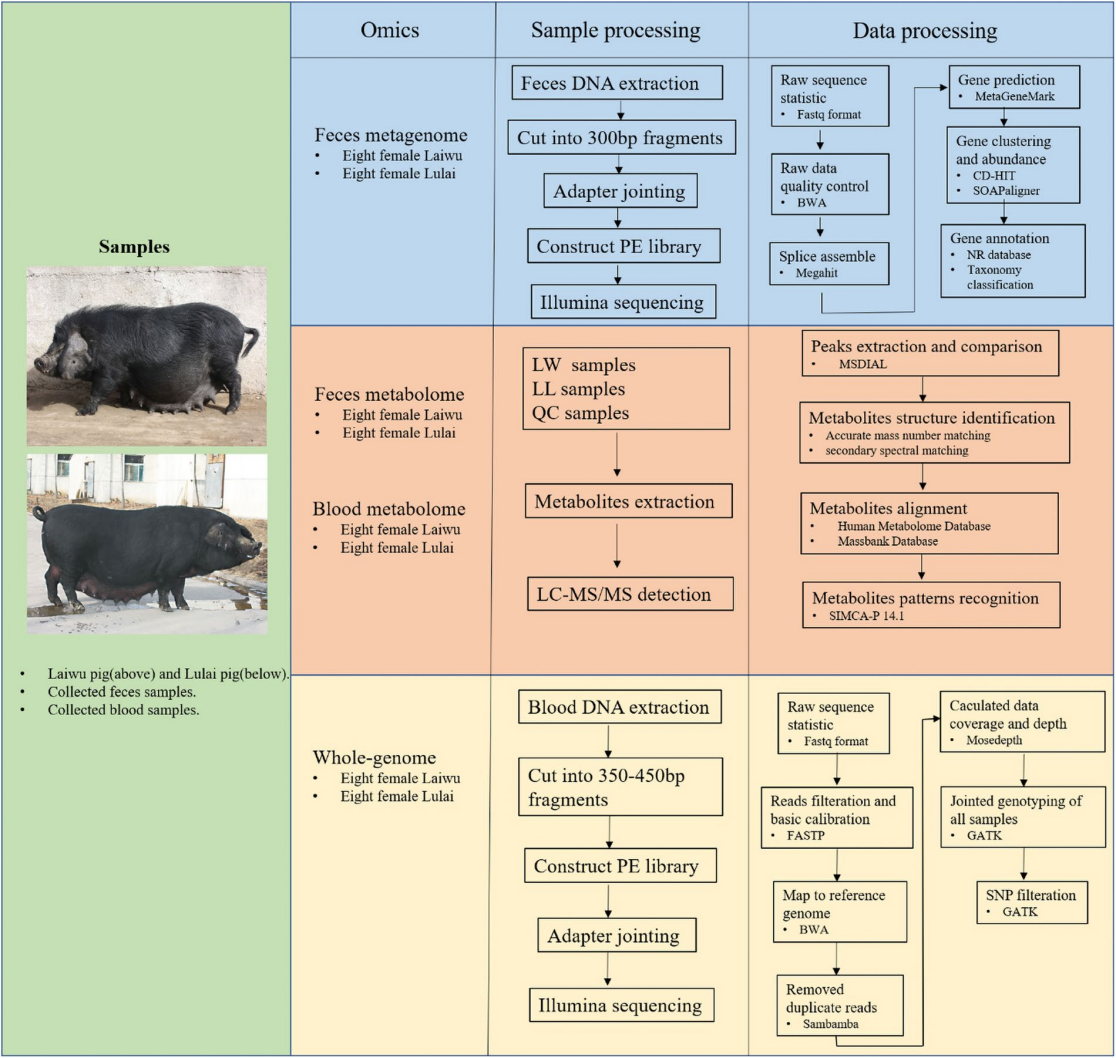
Schematic representation of the workflow for microbiome-metabolome-genome omics sample collection, sample processing, and data processing.

Pigs tend to resistant to metabolic diseases such as non-alcoholic fatty liver disease (NAFLD), T2DM and CVD though fed diets high in fat, fructose, and carbohydrates^28,29^. This phenomenon is similar to metabolic healthy obesity (MHO) who are obese but protect against metabolic diseases^30^. The Chinese demostic *Laiwu pig* (LW) is known for its high fat content, including subcutaneous fat and intramuscular fat (IMF)^31–34^. In particular, the IMF of LW was up to more than 7%, the average was up to 11.6%, and the highest individual was up to 21%. LW was crossed with the western commercial pig *Yorkshire pig* (YS) to breed the *Lulai pig* (LU) which has 50% LW gene infiltration^35^. The fat content of LU was lower than that of LW, and the IMF was about 5%. In this study, we chose eight LW and eight LU pigs with similar diet, hygiene, and environmental conditions for centralized management over two years (Table 1). We processed the fecal microbiome, fecal metabolome, blood metabolome, and whole genome of the target pigs (as shown in Fig. 1) to identify key factors that protect individuals against metabolic diseases through omics integration analysis.

**Table 1:** Gender and age information for each sample.

Sample ID	Gender	Age (days)	Sample ID	Gender	Age (days)
LW_F_1	Female	679d	LU_F_1	Female	743d
LW_F_2	Female	733d	LU_F_2	Female	748d
LW_F_3	Female	723d	LU_F_3	Female	741d
LW_F_4	Female	682d	LU_F_4	Female	737d
LW_F_5	Female	675d	LU_F_5	Female	737d
LW_F_6	Female	703d	LU_F_6	Female	714d
LW_F_7	Female	715d	LU_F_7	Female	743d
LW_F_8	Female	684d	LU_F_8	Female	728d

In conclusion, our study generated a high-quality dataset of fecal metagenome, fecal metabolome, blood metabolome, and whole-genome sequences from LW and LU pigs. The fecal metagenome produced 34.5 gigabyte (Gb) and 35 Gb of unassembled raw reads and revealed significant differences in the abundance of Spirochetes and *Treponema*, which are involved in carbohydrate metabolism. We identified a total of 1,220 metabolites in the fecal metabolome and 713 metabolites in the blood metabolome, both of which were rich in medium and long-chain fatty acids. Blood metabolome contained some anti-metabolic disease components, such as hydroxy fatty acids, tanshinone IIA, and betaine. The whole genome obtained an average of 21.9 Gb paired-end reads with a total number of 23.5 millions single nucleotide polymorphisms (SNPs) from 18 autosomes. The *Fst* analysis (fixation index, Wright’s F-statistics) of SNPs identified 4 KEGG (kyoto encyclopedia of genes and genomes) pathways, including bile secretion and fat digestion and absorption, which were enriched in the top 1% of *Fst*. The RNA expression analysis of adipose tissues of 16 pigs identified 412 differentially expressed genes, which were enriched in 9 KEGG pathways, including starch and sucrose metabolism and glycerophospholipid metabolism. The functional annotation of differential genes showed that lipid and glucose metabolism were the main enriched functions, consistent with the functions of differential microbiota and metabolites. Furthermore, the down-regulated gene *RGP1* was found to be strongly negatively correlated with *Treponema*, indicating that the expression of *RGP1* was closely related to the change of *Treponema* abundance. In summary, our study provides new insights into the role of gut microbiota, metabolites, and host genetics in the development of metabolic diseases. The identification of anti-obesity or anti-metabolic disease factors through the integration of microbiomic-metabolomic-genomic data has the potential to lead to the development of new therapeutic strategies for these diseases.

## Methods

### Animal rearing and samples collection

Our experiment was designed to compare eight female *Laiwu pigs* (LW) with eight female *Lulai pigs* (LU) which crossbred between LW and *Yorkshire* breeds. All pigs were born and raised for approximately two years (715 ± 33 days, Table 1) under uniform housing and feeding conditions at Jing-Qi-Shen pig farm in Jilin province, China. Temperature, humidity, and light varied with the natural climate conditions. Piglets from different mothers were used, and one piglet per litter was randomly chosen. Piglets were similar in age, with the oldest and youngest pigs in the experiment separated by 66 days. During the suckling period, piglets stayed with their mother, and then they were transferred to a pigsty with automatic feeders. Piglets were fed five times a day, three times before mating and once in the morning and evening after pregnancy. When the sows were sexually mature, they participated in normal sexual mating and birth. The sows were not pregnant at the time of sample collection.

Pig poop times were irregular and sample collection ranged from 10 a.m. to 5 p.m. Sampling was conducted for fecal and blood samples. To keep fecal samples free of contamination, we wear clean disposable sterile gloves and capture pig manure before it touches the ground. The fresh fecal samples were immediately preserved in sample collection tubes that were prepared and pre-filled with a bacterial DNA protective agent. The fecal samples were then placed into liquid nitrogen for rapid cooling. Two tubes of fecal samples were collected from each pig, one for microbiome profiling and another for metabolome profiling. The same group of pigs underwent an overnight fast of 14 hours before blood sample collection. Five milliliters of blood were collected from the jugular vein of each pig using a syringe. The fresh blood was preserved in a blood procoagulant tube and placed at room temperature for one hour. The blood mixture was then centrifuged at 3,000 g at 4 °C for 10 minutes. The upper serum of blood was transferred to a clean 1.5 mL tube. All fecal and blood samples were labeled and transported with dry ice to the laboratory for further processing.

### Microbe DNA extraction and sequencing

We used the E.Z.N.Asoil DNA isolation kit (OMEGA, Norcross, GA, U.S) to extract microbiota DNA following the manufacturer’s instructions. Absorbance at optical density (OD) 1.8 to 2.0 and 1% agarose gel electrophoresis were used to assess the DNA integrity and DNA quality, and our sample DNA met these criteria. The whole DNA sequence was cut into short fragments using a Covaris M220 system (Qsonica, USA). The 300 bp fragments were constructed into a pair-ends (PE) library using a TruSeq™ DNA sample preparation kit (Illumina, San Diego, CA). The PE library was assessed using Truseq PE cluster kit v3-cBot-HS (Illumina, San Diego, CA), and the library fragment amplification was performed using polymerase chain reaction (PCR). We used 1.5 μg samples for next generation sequencing (NGS) in an Illumina NovaSeq. 6000 platform.

### Microbiome data processing

The output NGS sequencing data were preserved in fastq format. Raw data were checked for quality control using Trimmomatic^36^ (v0.39) and processed using the following criteria: (a), if the average mass value was lower than 20 within the setting 50 bp sliding window, the tail of the unconformity quality reads were abandoned; (b), those sequences containing two unknown nucleotides (marked with N) were abandoned; (c), sequences with adaptor contamination were excluded; (d), sequence lengths below 50 bp and tail mass values lower than 20 were excluded. After trimming, high quality sequences remained. In order to exclude those sequences obtained from the host genome, the remaining sequences were mapped to the porcine DNA reference genome (Sscrofa 11.1), and Burrows-Wheeler Aligner^37^ (v0.7.17) was used to remove the high similarity reads. The remaining sequences were de novo assembled into contigs using Megahit^38^ (v1.1.1). Finally, the assembling contigs had their open reading frames (ORFs) predicted using MetaGeneMark^39^ (v3.25). Sequences were clustered using CD-HIT^40^ with parameters set at 95% consistency and 90% coverage. The longest sequences of each cluster were selected to construct a non-redundant gene catalog. Then, the above remaining high-quality sequences were compared to the non-redundant gene catalog (set at 95% identity) using SOAPaligner^41^, and we obtained a particular gene set and gene abundance. The gene set was compared to the Non-Redundant Protein Sequence database (NR database) using BLAST (v2.2.28) to obtain the taxonomic annotation and abundance (alignment parameter e-value was set as 1e-5). Finally, the taxonomic abundances of the six classification levels of kingdom, phylum, class, order, family, genus, and species were analyzed.

### Fecal and blood metabolite extraction

Fecal and blood samples were extracted and analyzed separately. Before sample processing, we preliminarily prepared 3 quality control (QC) samples which were mixed LW and LU samples in equal amounts. Then, LW, LU and QC samples were separated to 100 μl by mixing with 100 μl pre-cooled water and 800 μl precooled methanol/acetonitrile (1:1, v/v). The mixtures were placed on the ice bath and subjected to ultrasound for 60 minutes. To precipitate out the proteins, the mixtures were transferred to a refrigerator at −20 °C and incubated for 1 hour. The supernatant was transferred to clean sterile tubes and was centrifuged at 16,000 g, 4 °C for 20 minutes. Next, we used a high-speed vacuum enrichment centrifuge to dry the supernatant. The dried powder was resuspended by adding 100 μL acetonitrile/water solution (1:1, v/v), and this solution was centrifuged at 16,000 g, 4 °C for 15 minutes.

### Chromatographic separation and mass spectrometry

Chromatographic separation was performed by Agilent 1290 Infinity LC Ultra-High Performance Liquid Chromatography (UHPLC) platform with a quadrupole time-of-flight mass spectrometry (AB Sciex Triple TOF 5600) and HILIC column (Agilent 1290 infinity). QC samples which were used to evaluate the system stability and data reliability were inserted into the sample queue. The column temperature was 25 °C, and the flow rate was 0.3 mL/min. There were two mobile phases, phase A contained water, 25 mM ammonium acetate, and 25 mM ammonia water, Phase B only contained acetonitrile. The mobile phase system running procedure was set as follows: 95% B at 0–0.5 min; 95% to 65% of B at 0.5–7 min; 65% to 40% of B at 7–9 min; 40% B maintained at 9–10 min; 40% to 95% of B at 10–11.1 min; 95% B maintained at 11.1–16 min.

The positive or negative ion mode of components was detected using electrospray ionization (ESI). ESI source condition was set as follows: ion source gas1 (Gas1), 60 psi; ion source gas2 (Gas2), 60 psi; curtain gas (CUR), 30 psi; source temperature, 600 °C; ionsapary voltage floating (ISVF), ±5500 V; TOF MS scan m/z range, 60–1200 Da; product ion scan m/z range, 25–1200 Da; TOF MS scan accumulation time, 0.15 s/spectra; product ion scan accumulation time, 0.03 s/spectra. Secondary mass spectrometry was obtained using information dependent acquisition (IDA) and was used in high sensitivity mode, declustering potential (DP), ±60 V; collision energy, 30 eV. IDA was set as follows: exclude isotopes within 4 Da; candidate ions to monitor per cycle, 6.

### Metabolite data processing

The raw mass spectrometry (MS) data were converted into mzXML files by ProteoWizard. The program XCMS in MSDIAL software was used for peak alignment, retention time correction, and extraction of peak area. For the extracted data, removed the ion peaks with missing values >50% in the group. The positive and negative ion peaks then were integrated, and the software SIMCA-P 14.1 (Umetrics, Umea, Sweden) was used for pattern recognition. Accurate mass matching (<25 ppm) and secondary spectrum matching were used for metabolite structure identification, and the database such as Human Metabolome Database (HMDB) and Massbank Database were searched. After retrieving metabolites, metabolites were classified using MSDIAL search software. The data was normalized by Pareto-scaling for subsequent analysis.

### Blood DNA extraction and sequencing

Blood DNA extraction was carried out in accordance with the TruSeq DNA LT Sample Prep Kit (Illumina, San Diego, CA) protocol. DNA quality was assessed by measuring absorbance at OD 1.6 to 1.8 using a NanoDrop 2000 Spectrophotometer (Thermo Fischer Scientific, USA), while DNA integrity was confirmed via 1% agarose gel electrophoresis. Subsequently, the DNA sequence was fragmented into 350–450 bp fragments using Covaris M220. The fragment ends were repaired and phosphorylated, followed by the connection of the adaptor using the NextFlex^TM^ Rapid DNA-Seq Kit (Bioo Scientific, USA). Finally, the library was amplified via 15 cycles of PCR to enrich small fragments. The quality and concentration of the library were determined using Qubit (Thermo Fischer Scientific, USA), and PE150 sequencing was performed on the Illumina NovaSeq. 6000 platform.

### Genome data processing

The sequencing data was saved in the fastq format. The Fastp^42^ (v0.20.0) was used with default parameters to read, filter and profile the quality of the reads. BWA^37^ (v0.7.17) was used to map high-quality reads to the pig reference genome (Sscrofa11.1). SAM files were converted to BAM files by SamTools^43^ (v1.10). Duplicate reads were removed using Sambamba^44^ (v0.7.1). The data coverage and depth were calculated using Mosdepth^45^ (v0.2.9). GATK^46^ (v4.1.6) Haplotypecalle was used to process each sample and generate an intermediate GVCF, which was used for joint genotyping of all samples in genotype GVCFs. Finally, SNPs were filtered based on the following criteria: (1) QD < 2.0, FS > 60.0, MQ < 40.0, MQRankSum <−12.5, ReadPosRankSum <−8.0, SOR > 3.0; (2) minor allele frequency (MAF) < 0.01; (3) call rate of GATK variants < 0.9. The number of SNPs obtained is shown in Table 4. Genotype density distribution was mapped using the CMplot R package. Principal components analysis (PCA) was calculated using Plink^47^ (v1.9). Population genetic structure analysis was performed using Admixture^48^ (v1.3.0). PCA and Admixture analyses included the SNPs of *Yorkshire pigs* (YS), *Duroc pigs* (DU) and *Landrace pigs* (LR) were obtained from the PHARP database^49^ (http://alphaindex.zju.edu.cn/PHARP/index.php). *F*_*ST*_ analysis was performed using VCFtools^50^ (v0.1.13,–fst-window-size 50,000–fst-window-step 10,000. Window size 50 K, step size 10 K). Gene expression prediction was performed using the FarmGTEx TWAS-server^51,52^ (http://twas.farmgtex.org/). Functional annotation for gene ontology (GO) and KEGG was performed using http://kobas.cbi.pku.edu.cn/.

### Metagenomic data analysis

**Fig. 2:**
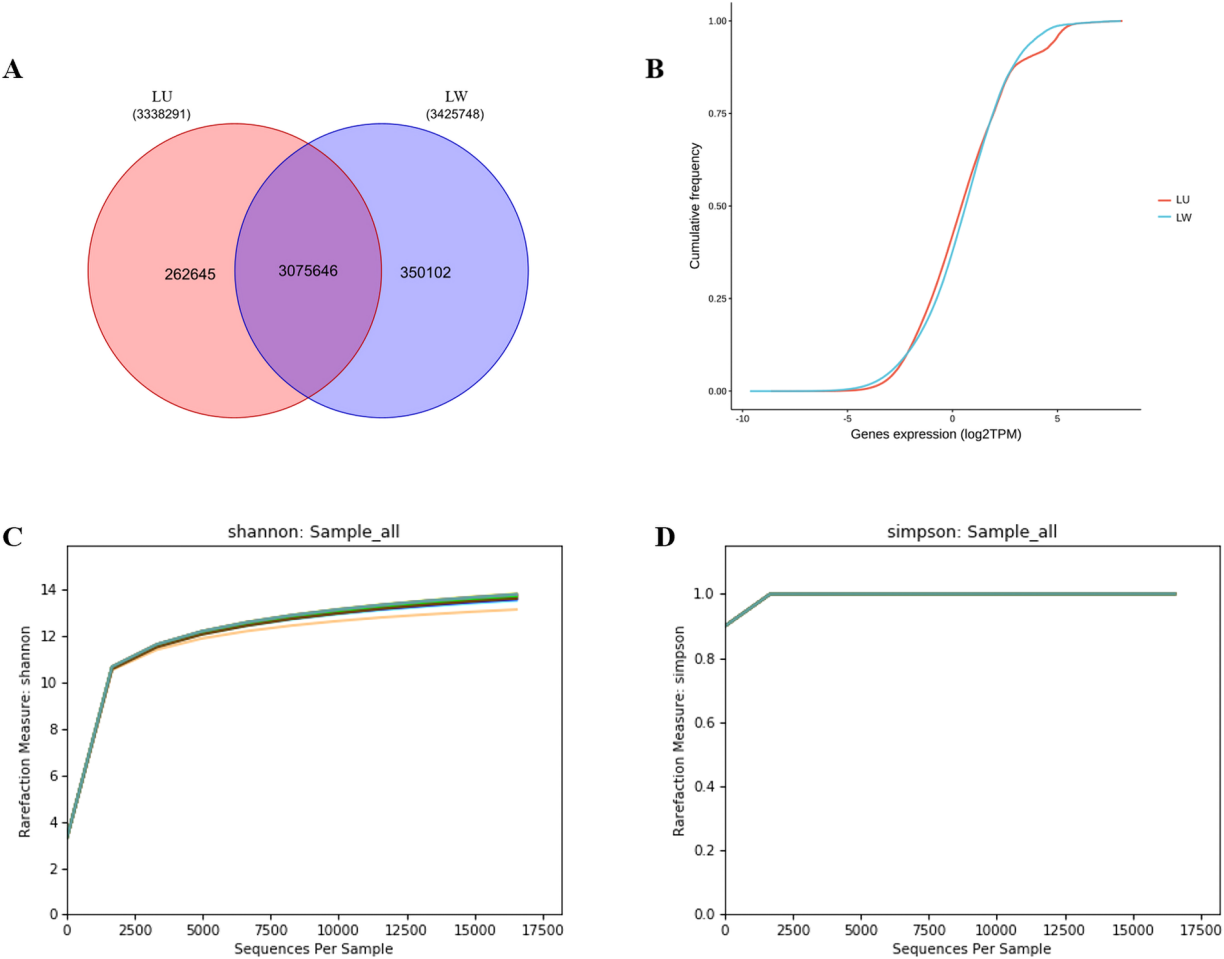
Microbial gene statistics and diversity comparison between LW and LU. (**A**) Gene statistics. (**B**) Cumulative frequency statistics of genes. (**C**) Shannon diversity index. (D). Simpson diversity index.

The fecal metagenome generated 34.5 Gb and 35 Gb of unassembled raw reads from LW and LU samples, respectively. After quality control, the sequence Q20 ratio (bases with a mass value of 20 as a percentage of the total number of bases) exceeded 96.99% and Q30 ratio exceeded 91.67%, indicating that the data quality was suitable for further analysis. On average, 5.5 million and 5.7 million clean reads were obtained from LW and LU datasets, respectively (Table 2). The intergroup diversity of the sequences between the two porcine breeds was calculated using shannon and simpson diversity index, and there was no notable difference in the overall sequences (Fig. 2C,D). The LU group was infiltrated with 50% of LW genes and maintained in a consistent environment for approximately two years, which may account for the indiscriminate microbial composition of the two groups of pigs. Clean reads were assembled into contigs and clustered based on 95% similarity and 90% coverage to generate a non-redundant gene catalog comprising a total of 4.2 million ORFs with an average length of 622 base pairs. Gene annotation revealed that 262,645 genes were unique to LU and 350,102 genes were unique to LW (Fig. 2A). Despite having more sequences and contigs than LW, LU had fewer annotated genes. In contrast to previous reports on the lower gene counts and bacterial diversity in obese individuals^53–55^, our results show that the more obese pigs have a higher gene count, which is contrary to the previous finding. The cumulative frequency statistics of gene abundances from the two porcine breeds showed no significant difference in most intervals, but genes with a count of nearly 40 were significantly more abundant in LW than in LU (Fig. 2B). This finding indicates that the two porcine breeds have different compositions, mainly located in this interval.

**Table 2:** Statistics of sequences, contigs, ORFs, and the mass value of clean reads for each sample.

Sample ID	Clean reads	Error%	Q20%	Q30%	GC%	Contigs (number)	ORFs (number)
LW-F-1	25,742,676	0.0237	98.49	95.50	50.82	204,891	369,142
LW-F-2	31,905,416	0.0242	98.29	95.03	52.5	244,783	449,000
LW-F-3	37,403,822	0.0236	98.53	95.62	53.85	306,291	538,258
LW-F-4	60,663,316	0.0265	97.42	92.68	52.84	380,835	739,118
LW-F-5	64,310,816	0.0267	97.33	92.52	52.74	427,285	834,279
LW-F-6	84,473,838	0.0264	97.47	92.79	53.71	449,988	937,422
LW-F-7	57,571,190	0.0264	97.51	92.82	50.49	411,427	791,341
LW-F-8	76,238,898	0.0256	97.82	93.46	52.63	540,800	1,036,977
LU-F-1	33,028,042	0.0240	98.4	95.18	52.1	251,573	461,917
LU-F-2	29,230,424	0.0238	98.48	95.44	50.94	238,659	424,624
LU-F-3	31,118,364	0.0235	98.6	95.78	52.93	199,744	371,059
LU-F-4	74,557,864	0.0257	97.78	93.39	52.62	414,445	826,178
LU-F-5	63,920,876	0.0262	97.57	93.00	52.54	480,887	915,972
LU-F-6	71,637,054	0.0277	96.99	91.67	54.26	401,994	841,977
LU-F-7	67,836,418	0.0256	97.87	93.52	50.46	470,125	927,982
LU-F-8	80,789,052	0.0262	97.56	92.95	51.87	413,080	849,927

### Microbiota taxonomic assessment

**Fig. 3:**
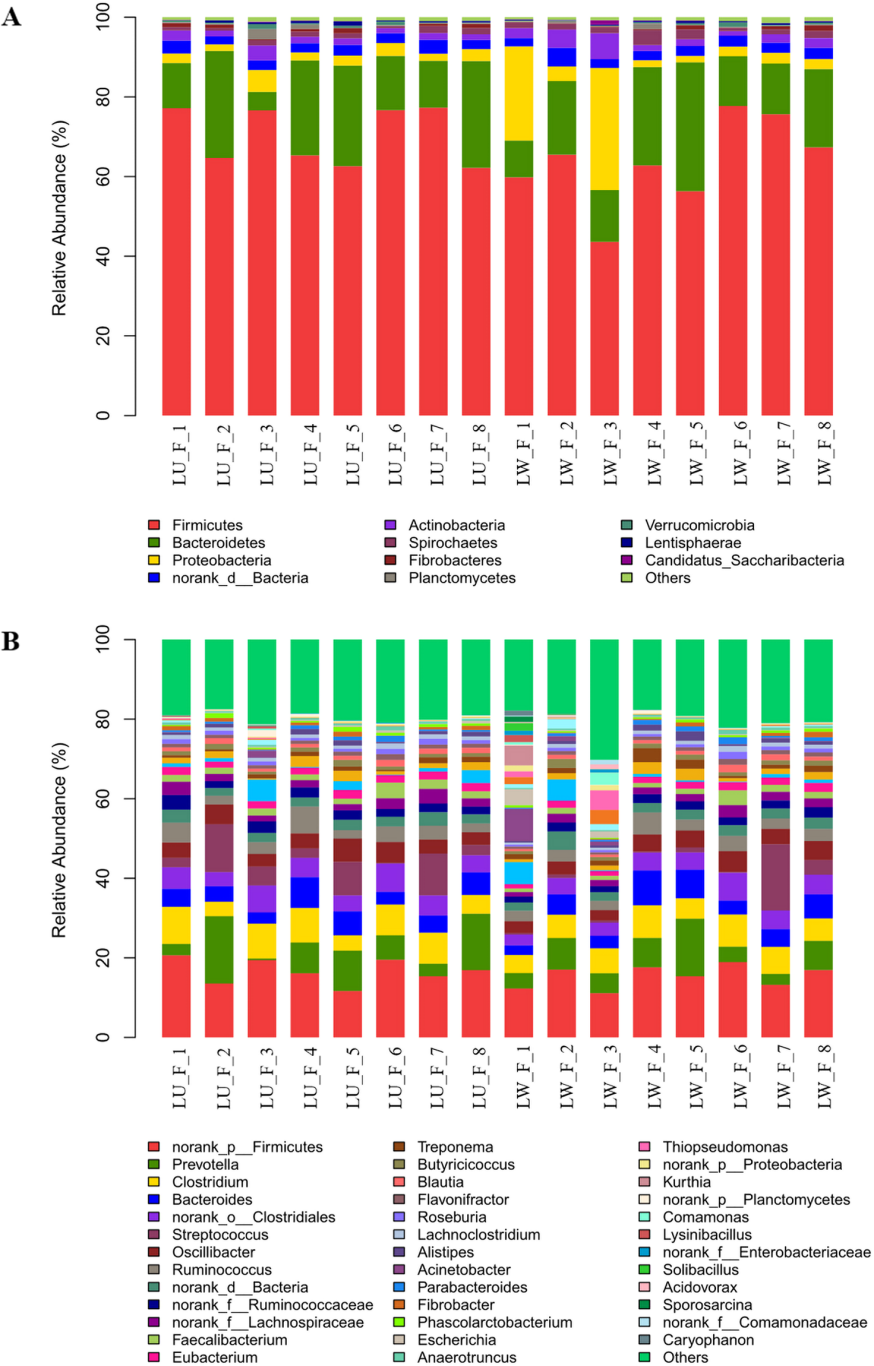
Composition of high-abundance microbiota in LW and LU. (A) Phylum classification level. (B) Genus classification level.

**Fig. 4:**
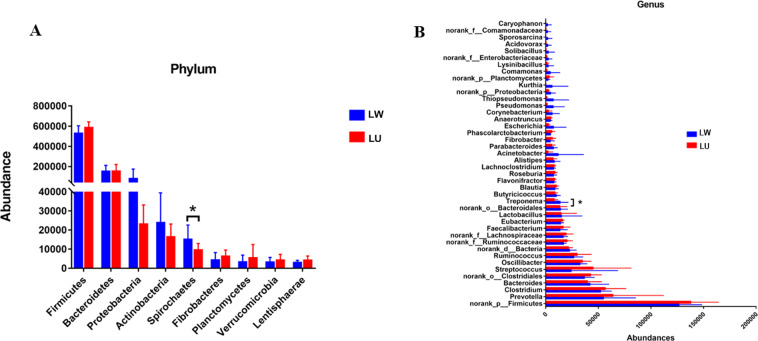
Differential microbiota at the phylum and genus taxonomic levels in LW and LU. (A) Phylum classification level. (B) Genus classification level.

The highly similar microbial environment of LW and LU may be attributed to the high degree of gene infiltration and rearing environment. However, the remaining differential microorganisms are likely to be involved in fat deposition, leading to differences in the fat content of the two pig breeds. Therefore, we conducted further analysis to identify the microbial differences between LW and LU. We summarized the microbiome at six taxonomic classification levels, including phylum, class, order, family, genus, and species. In LW, we detected a total of 146 phyla, 90 classes, 323 orders, 304 families, 2,691 genera, and 14,570 species (Table 3). Meanwhile, LU showed 145 phyla, 90 classes, 321 orders, 306 families, 2,651 genera, and 14,324 species (Table 3). Due to unknown taxonomic annotations at the class and family levels, the statistics were lower. At the phylum classification level, Firmicutes (66.94%), Bacteroidetes (17.93%) and Proteobacteria (5.69%) were the predominant phyla, with Actinobacteria (2.38%), Spirochaetes (1.46%), Fibrobacteres (0.62%), and Planctomycetes (0.5%) also being present in significant amounts (Fig. 3A, Supplementary Table S5). The total proportion of Firmicutes, Bacteroidetes, and Proteobacteria reached 91%, with the strongest niche competition, as the ratio was trading off (Supplementary Table S1). At the genus classification level, the predominant genera were *Clostridium* (6.55%), *Bacteroides* (4.93%), *Prevotella* (7.15%), *Streptococcus* (4.2%), *Oscillibacter* (4.05%), *Ruminococcus* (3.39%), *Faecalibacterium* (1.8%), and *Eubacterium* (1.8%) (Fig. 3B, Supplementary Table S2). We conducted a wilcoxon rank-sum test to analyze the differences between the phylum and genus taxonomic levels of LW and LU. The results revealed a significant difference in Spirochaetes abundance between LW and LU at the phylum classification level (Fig. 4A). Spirochaetes have been reported to be involved in the metabolic process of carbohydrates^56–59^. At the genus taxonomic level, there was a significant difference in *Treponema* abundance between LW and LU (Fig. 4B). *Treponema* is a genus belonging to Spirochaetes.

**Table 3:** Number of microbiota at six taxonomic levels in LW and LU.

Levels	Laiwu pigs	Lulai pigs	Laiwu-only	Lulai-only
Phylum	146	145	1	0
Class	90	90	0	0
Order	323	321	2	0
Family	304	306	2	4
Genus	2,691	2,651	50	10
Specie	14,570	14,324	255	9

### Metabolites data profiling

**Fig. 5:**
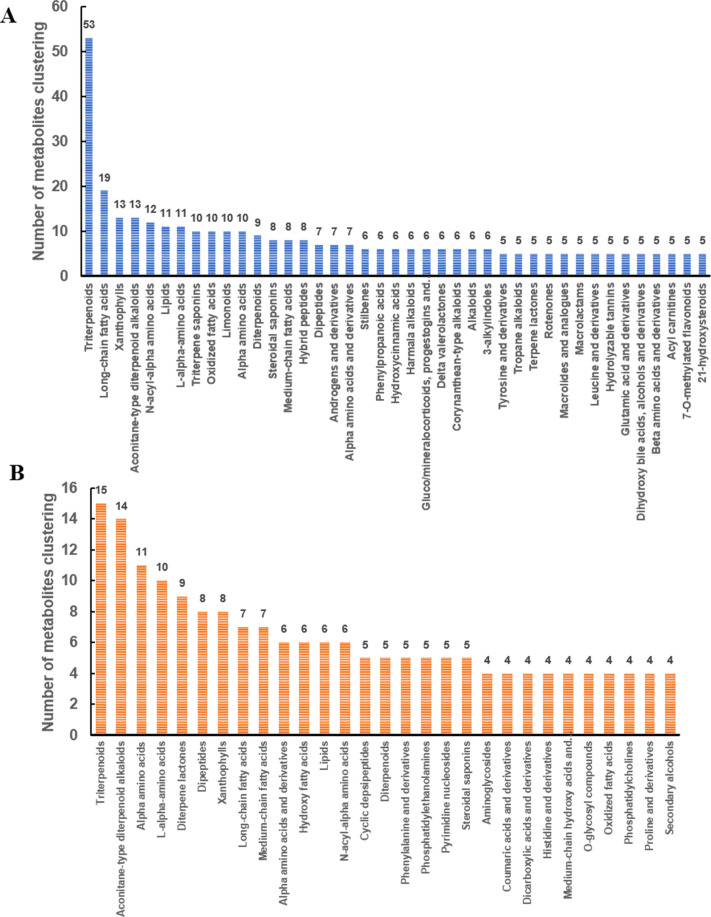
Classification of fecal and blood metabolome metabolites. (A) Fecal metabolome metabolites. (B) Blood metabolome metabolites. The number of metabolite components is ranked in descending order. The numerical values indicate the number of metabolites per class.

The total ion flow patterns (TIC) of the quality control (QC) samples were compared under positive and negative ion detection modes. The response strength and retention time of each chromatographic peak overlapped, indicating that the variation caused by instrument error is minimal and the data quality is reliable. For the fecal metabolome, we extracted 12,226 positive ion peaks and 6,891 negative ion peaks, of which 703 positive ion peaks and 517 negative ion peaks were annotated. The 1,220 annotated metabolites were categorized into 453 classes, including triterpenoids, long-chain fatty acids, and xanthophylls, with 53, 19, 13 kinds of metabolites, respectively (Fig. 5A, Supplementary Table S3). In the blood metabolome, we detected 5,977 positive ion peaks and 3,081 negative ion peaks, of which 368 positive ion peaks and 345 negative ion peaks were annotated. The 713 annotated metabolites were categorized into 360 classes, including triterpenoids, aconitane-type diterpenoid alkaloids, and alpha amino acids, with 15, 14, 11 metabolites, respectively (Fig. 5B, Supplementary Table S4). It is worth noting that long-chain and medium-chain fatty acids were the major fatty acids in both the fecal and blood metabolomes. These fatty acids are easily oxidized and hydrolyzed, and can reduce blood lipids and cholesterol, which may be related to the lower susceptibility of pigs to obesity-related metabolic diseases. The composition of the fecal metabolome was similar to that of the blood metabolome, containing triterpenoids, xanthophylls, long-chain fatty acids, medium-chain fatty acids, lipids, and alpha amino acids (Fig. 5A,B). The composition of the main metabolites of the two metabolomes is highly similar, and some of their substances are likely related.

### Comparison of blood metabolites

Blood metabolites play a crucial role in regulating physiological health, and understanding their influence can provide insight into how pigs are protected from metabolic diseases. To investigate this, we analyzed the blood metabolome and measured the influence intensity and explanatory ability of metabolite expression patterns using variable importance for the projection (VIP) obtained through an OPLS-DA model. We selected metabolites with VIP >1 and Pvalue < 0.05 (one-way ANOVA for multi-group comparison) to identify those with significant differences. Our results revealed 81 metabolites that differed significantly between the two porcine groups (Supplementary Table S5). Of these, 41 metabolites were more abundant in LW, including angelicin, securinine, hypoxanthine, betaine, cytidine, homocysteine, curdione, inosine, isopimpinellin, 5-methoxypsoralen, palmitoylcarnitine, citrate, stearic acid, cytarabine, licochalcone A, and N-acetylneuraminic acid. On the other hand, 40 metabolites were more abundant in LU, including nitrazepam, acetaminophen, icosanoic acid, gabapentin, spegatrine, juarezic acid, dehydroeffusol, gomisin H, and DL-2-hydroxyvaleric acid. Notably, some of these changing blood metabolites may be related to the fat content of pigs, as they have been shown to have anti-adipogenesis and anti-chronic metabolic disease effects. For instance, hydroxy fatty acids have been reported to exhibit anti-diabetic and anti-inflammatory effects^60^, and tanshinone IIA is used to treat cardiovascular diseases and has anti-adipogenesis effects^61–63^. Betaine has anti-fatty liver and anti-inflammatory properties, which can prevent hyperglycemia and reduce insulin resistance^64–66^.

### Genomic data analysis

**Fig. 6:**
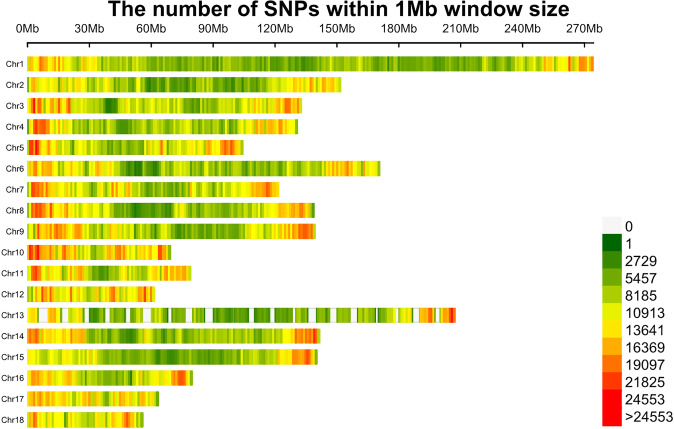
Distribution of SNPs on chromosomes. The x-axis shows the chromosomal position (in Mb), and the y-axis represents chromosomes. Different colors correspond to the number of SNPs in each 1 Mb genome block.

**Fig. 7:**
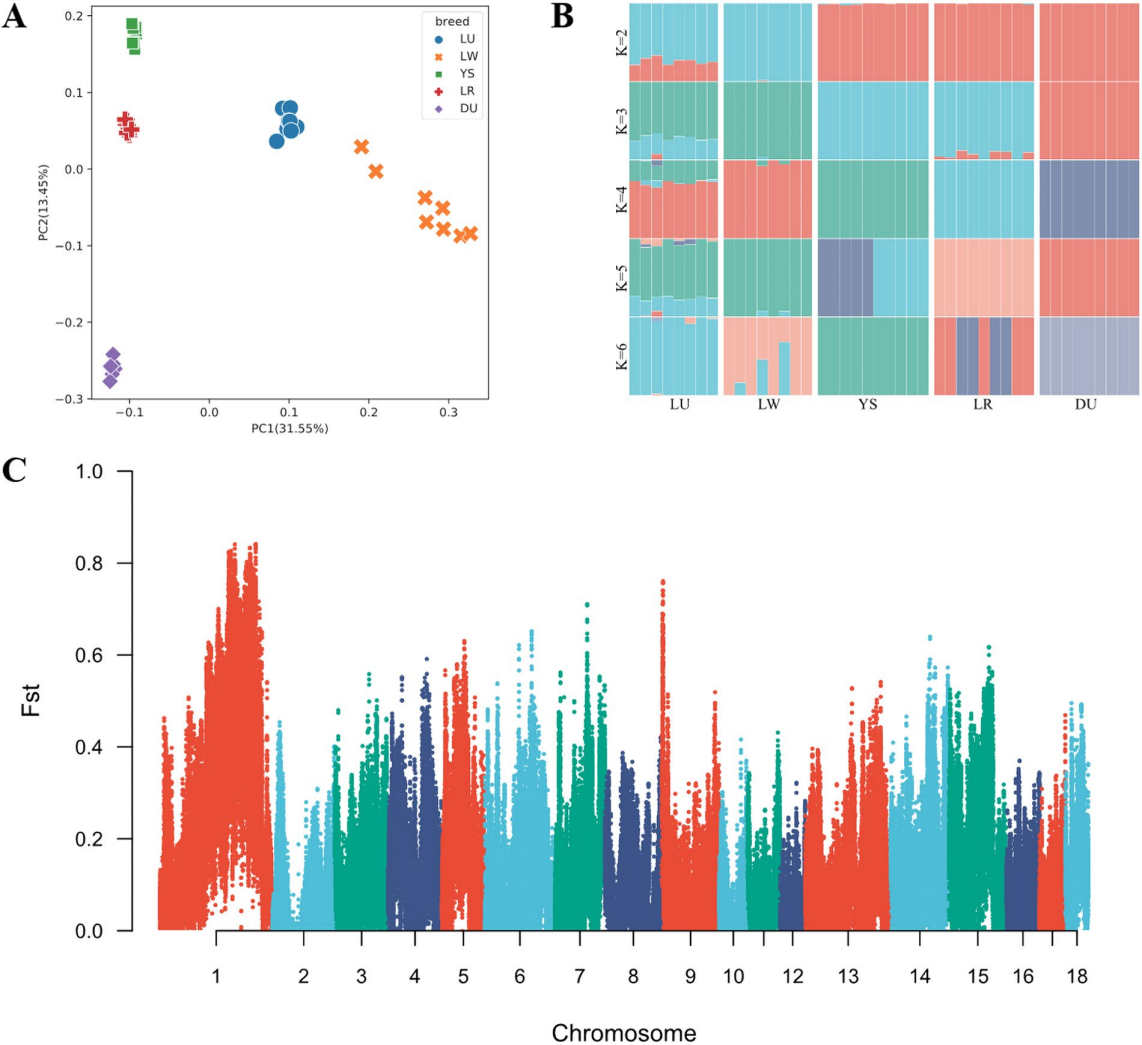
Pedigree and group differentiation between LW and LU. (**A**) Principal component analysis results of LW, LU, YS, LR and DU pig breeds. Blue, orange, red, pruple and green markers represent LW, LU, YS, LR and DU pigs, respectively. (**B**) Ancestry composition results with the assumed number of ancestries at K = 2. K is an adjustable parameter representing the number of possible ancestral varieties. Through the calculation of the cross validation error, we obtained K = 2 as the best K value. (**C**) Manhattan plot based on *Fst* of LW and LU.

The LW and LU samples yielded an average of 22 Gb and 21.9 Gb paired-end reads, respectively, from which 144.6 million and 143.5 million clean reads were obtained after quality control. The genomic data quality was high, with all sequence Q20 ratios above 95.69% and Q30 ratios above 89.27% (Supplementary Table S6). The average genomic sequencing depth was 6.8-fold, with coverage reaching 97%, and a total of 22.7 million SNPs (minor allele frequency ≥ 0.05) were obtained from 18 autosomes after assembly, SNP calling, and SNPs filtering (Table 4). The high-density of nucleotide diversity in 1 mbyte (Mb) non-overlapping window covers all genomes (Fig. 6). PCA and admixture analyses revealed clear differences in the pedigree of LW and LU, with LW and LU pig breeds being well-distinguished from *Yorkshire pig* breed (Fig. 7A,B). Additionally, the *Fst* method was used to detect the selection signatures between LW and LU. The *Fst* peak value was up to 0.8, which means that their group differentiation is relatively high (Fig. 7C). Top 1% *Fst* can be annotated to 811 genes (Supplementary Table S7). These genes were annotated by functional enrichment, resulting in 6 GO pathways and 4 KEGG pathways, including bile secretion and fat digestion and absorption (Supplementary Table S10). RNA expression analysis using the FarmGTEx TWAS-server predicted a total of 2,930 genes in individual adipose tissues in LW and LU, of which 146 were up-regulated and 266 were down-regulated (Supplementary Table S8). The differential gene functions were annotated, resulting in 6 GO pathways and 9 KEGG pathways, including starch and sucrose metabolism and glycerophospholipid metabolism (Supplementary Table S10). Additionally, spearman correlation analysis identified 42 genes strongly associated with the differential microbiota *Treponema* at the genus taxonomic level, including 2 upregulated genes (ENSSSCG00000025565 and ENSSSCG00000049578) and 1 downregulated gene *RGP1* (| Cor | > 0.6, Pvalue < 0.05, Supplementary Table S9).

**Table 4:** Number of SNPs on 18 autosomes.

Chromosomes	Num_total	Num_filter	Num_pass
Chr1	2,285,394	29,787	2,237,351
Chr2	1,425,932	22,513	1,392,987
Chr3	1,459,802	15,900	1,434,774
Chr4	1,345,484	15,208	1,321,148
Chr5	1,243,806	15,421	1,221,213
Chr6	1,663,634	21,691	1,630,207
Chr7	1,404,598	20,442	1,375,680
Chr8	1,435,381	16,227	1,410,026
Chr9	1,541,622	22,400	1,510,094
Chr10	1,035,035	13,280	1,017,191
Chr11	965,732	9,157	951,359
Chr12	832,849	11,737	816,548
Chr13	1,321,298	18,608	1,288,998
Chr14	1,454,835	19,616	1,425,439
Chr15	1,262,841	15,796	1,237,265
Chr16	943,461	9,972	928,273
Chr17	858,256	12,570	841,122
Chr18	691,507	6,542	681,053

## Data Records

This study presents four distinct datasets: fecal metagenome, fecal metabolome, blood metabolome, and whole genome. Raw data for the metagenome and genome are stored in the NCBI Sequence Read Archive in fastq format. We have conducted preliminary quality control and statistical analyses. Supplementary tables containing taxonomic ratio are provided.

### FASTQ data

The raw fastq files for metagenomic sequencing data are available in the NCBI SRA database under BioProject PRJNA747893^67^ (NCBI Accession column in Supplementary Table S11), with the project title “Metagenomic Data of Laiwu Pigs and Lulai Pigs”. The raw fastq files for whole-genome sequencing (WGS) data are also available in the NCBI SRA database under BioProject PRJNA749115^68^ (NCBI Accession column in Supplementary Table S12), with the project title “Whole Genome Sequencing Data of LW and LL”.

### Metabolome data

The raw files for fecal metabolome and blood metabolome data are stored in the MetaboLights database^69^ under the study unique identifier MTBLS397769^70^. The project title is “Integrated Microbiome-Metabolome-Genome Axis Data of Laiwu and Lulai Pigs in China”.

### Metadata statistics

We conducted quality control and preliminary analysis on raw data from multiple omics to facilitate more rapid reuse by scholars. The preliminary statistical data can be accessed through supplementary information. At the same time, the Excel version attachment has been uploaded to http://alphaindex.zju.edu.cn/ALPHADB/download.html. The supplementary information includes:

1. Table S1: Phylum classification ratio for each sample.
2. Table S2: Genus classification ratio for each sample.
3. Table S3: Complete list of fecal metabolites for *Laiwu pigs* and *Lulai pigs*.
4. Table S4: Complete list of blood metabolites for *Laiwu pigs* and *Lulai pigs*.
5. Table S5: 81 different blood metabolites between *Laiwu pigs* and *Lulai pigs*.
6. Table S6: Clean data information for the whole genome.
7. Table S7: 811 annotated genes for top 1% Fst.
8. Table S8: 2,930 genes for adipose tissues predicted from SNPs using FarmGTEx TWAS-server.
9. Table S9: Strong correlation between predicted genes and *Treponema*.
10. Table S10: Functional enrichment annotation for Fst and RNA using GO and KEGG.
11. Table S11: NCBI SRA accession column for PRJNA747893.
12. Table S12: NCBI SRA accession column for PRJNA749115.

## Technical Validation

To ensure sample authenticity and prevent contamination during the sampling process, disposable PE gloves were used to collect fecal samples immediately after defecation by the target pigs. Samples were then transferred to specific fecal sample preservation tubes, and their unique sample IDs were matched with DNA extraction and sequencing IDs. The quality of the DNA was confirmed using a NanoDrop 2000 spectrophotometer and agarose gel electrophoresis. High-quality DNA sequencing was performed using NovaSeq. 6000 sequencing technology. Raw data obtained from metagenomic and whole-genome sequencing were subjected to quality control to obtain high-quality reads for further analysis. The Q30 values for the raw metagenomic sequencing data of 16 samples ranged from 91.65% to 95.8%. After quality control, the Q30 values ranged from 91.67% to 95.78%. For whole-genome raw data, the Q30 range was 88.55% to 90.45%, while the Q30 range of clean reads after quality control was 89% to 91%. To control metagenomic gene abundance, transcripts per million (TPM) normalization was used. The total ion current mode (TIC) of QC samples in positive and negative ion detection modes were imposed and compared. The response strength and retention time of each chromatographic peak were coincidental, indicating that the instrument error was minimal and that the data quality was reliable.

## Supplementary information

Preliminary metadata statistics

## Data Availability

The software required for data processing and analysis and image generation in this study are accessible, the software versions as follows: 1. Trimmomatic (v0.39, http://www.usadellab.org/cms/index.php?page=trimmomatic) 2. BWA(v0.7.17, http://bio-bwa.sourceforge.net) 3. Megahit (http://i.cs.hku.hk/~alse/hkubrg/projects/idba_ud/) 4. MetaGeneMark (v3.25, http://exon.gatech.edu/meta_gmhmmp.cgi) 5. CD-HIT (http://www.bioinformatics.org/cd-hit/) 6. SOAPaligner (http://soap.genomics.org.cn/) 7. BLASTP (BLAST v2.2.28+, http://blast.ncbi.nlm.nih.gov/Blast.cgi) 8. MSDIAL (v4.7, http://prime.psc.riken.jp/compms/msdial/main.html) 9. SIMCA-P (v14.1) 10. Fastp (v0.20.0, http://opengene.org/fastp/fastp) 11. Samtools (v1.10) 12. Sambamba (v0.7.1) 13. Mosdepth (v0.2.9) 14. Picard Tools (v2.0.1) 15. Bcftools (v1.939) 16. GATK (v4.1.6) 17. Plink (v1.9, Complete flag index - PLINK 1.9 (cog-genomics.org)) 18. Admixture (v 1.3.0) 19. VCFtools (v0.1.13) 20. FarmGTEx TWAS-server (http://twas.farmgtex.org/)
