# Supplementary material for: Integrated microbiome-metabolome-genome axis data of Laiwu and Lulai pigs
Source: Sci Data. 2023 May 13;10:280. doi: 10.1038/s41597-023-02191-2 (PMC10183000; doi:10.1038/s41597-023-02191-2)
Supplement: Supplementary file 1 — Preliminary metadata statistics [file 41597_2023_2191_MOESM1_ESM.pdf]

Supplementary Information for

Integrated microbiome-metabolome-genome axis data of Laiwu and Lulai pigs

Xueshuang Lai, Zhenyang Zhang, Zhe Zhang, Shengqiang Liu, Chunyan Bai, Zitao Chen, Qamar Raza Qadri, Yifei Fang, Zhen Wang, Yuchun Pan*, Qishan Wang*

*Corresponding authors: Qishan Wang (wangqishan@zju.edu.cn)

This PDF file includes:

Table S1: Phylum classification ratio for each sample……………………………………………………………………1

Table S2: Genus classification ratio for each sample…………………………………………………………………..2

Table S3: Complete list of fecal metabolites for *Laiwu pigs* and *Lulai pigs*……………………………………………..3

Table S4: Complete list of blood metabolites for *Laiwu pigs* and *Lulai pigs*…………………………………………....36

Table S5: 81 different blood metabolites between *Laiwu pigs* and *Lulai pigs*……………………………………………..55

Table S6: Clean data information for the whole genome…………………………………………………….………..57

Table S7: 811 annotated genes for top 1% Fst…………………………………………………………………………58

Table S8: 2,930 genes for adipose tissues predicted from SNPs using FarmGTEx TWAS-server………………………………123

Table S9: Strong correlation between predicted genes and *Treponema*………………………………………………...189

Table S10: Functional enrichment annotation for Fst and RNA using GO and KEGG…………………………………………190

Table S11: NCBI SRA accession column for PRJNA747893……………………………………………………….……….191

Table S12: NCBI SRA accession column for PRJNA749115…………………………………………………………………..192

Table S1: Phylum classification ratio for each sample.

Taxonomy	LU_1	LU_2	LU_3	LU_4	LU_5	LU_6	LU_7	LU_8	LW_1	LW_2	LW_3	LW_4	LW_5	LW_6	LW_7	LW_8
Firmicutes	0.77	0.65	0.77	0.65	0.63	0.77	0.77	0.62	0.60	0.66	0.44	0.63	0.56	0.78	0.76	0.67
Bacteroidetes	0.11	0.27	0.05	0.24	0.25	0.14	0.12	0.27	0.09	0.18	0.13	0.25	0.32	0.12	0.13	0.20
Proteobacteria	0.02	0.02	0.06	0.02	0.03	0.03	0.02	0.03	0.24	0.04	0.31	0.02	0.02	0.02	0.03	0.03
norank_d__Bacteria	0.03	0.02	0.02	0.02	0.03	0.02	0.03	0.02	0.02	0.05	0.02	0.02	0.02	0.03	0.03	0.03
Actinobacteria	0.03	0.02	0.04	0.02	0.02	0.01	0.02	0.01	0.03	0.05	0.07	0.01	0.02	0.01	0.02	0.02
Spirochaetes	0.01	0.01	0.01	0.01	0.01	0.01	0.02	0.02	0.01	0.01	0.01	0.04	0.02	0.01	0.01	0.02
Fibrobacteres	0.01	0.01	0.00	0.01	0.01	0.00	0.01	0.01	0.00	0.00	0.00	0.00	0.01	0.00	0.01	0.01
Planctomycetes	0.00	0.00	0.03	0.01	0.00	0.00	0.00	0.00	0.00	0.01	0.00	0.01	0.00	0.00	0.00	0.00
Verrucomicrobia	0.00	0.00	0.01	0.00	0.01	0.01	0.00	0.00	0.00	0.00	0.00	0.00	0.00	0.01	0.00	0.00
Lentisphaerae	0.00	0.01	0.00	0.01	0.01	0.00	0.00	0.00	0.00	0.00	0.00	0.00	0.01	0.00	0.01	0.00
Candidatus_Saccharibacteria	0.00	0.00	0.00	0.00	0.00	0.00	0.00	0.00	0.00	0.00	0.01	0.00	0.00	0.00	0.00	0.00
Others	0.01	0.01	0.01	0.01	0.01	0.01	0.01	0.01	0.01	0.01	0.01	0.01	0.01	0.01	0.01	0.01

Table S2: Genus classification ratio for each sample.

**Taxonomy**	**LU_1**	**LU_2**	**LU_3**	**LU_4**	**LU_5**	**LU_6**	**LU_7**	**LU_8**	**LW_1**	**LW_2**	**LW_3**	**LW_4**	**LW_5**	**LW_6**	**LW_7**	**LW_8**
norank_p__Firmicutes	0.20626	0.13518	0.19389	0.16120	0.11661	0.19527	0.15350	0.16913	0.12295	0.17032	0.11098	0.17615	0.15347	0.18926	0.13220	0.16926
Prevotella	0.02882	0.16957	0.00436	0.07738	0.10152	0.06106	0.03172	0.14194	0.03892	0.07967	0.05006	0.07404	0.14482	0.03894	0.02756	0.07366
Clostridium	0.09314	0.03606	0.08744	0.08689	0.03833	0.07752	0.07797	0.04704	0.04525	0.05852	0.06292	0.08159	0.05119	0.08073	0.06801	0.05623
Bacteroides	0.04522	0.03904	0.02864	0.07730	0.06037	0.03205	0.04292	0.05661	0.02389	0.05097	0.03146	0.08778	0.07201	0.03518	0.04433	0.06041
norank_o__Clostridiales	0.05425	0.03550	0.06761	0.04849	0.04005	0.07107	0.05086	0.04319	0.02717	0.04118	0.03314	0.04504	0.04345	0.06967	0.04666	0.04929
Streptococcus	0.02372	0.12026	0.04762	0.02390	0.08456	0.00185	0.10504	0.02587	0.00392	0.00848	0.00529	0.00251	0.01273	0.00220	0.16669	0.03715
Oscillibacter	0.03854	0.04985	0.03156	0.03795	0.05827	0.05237	0.03528	0.03157	0.02997	0.03316	0.02596	0.04286	0.04234	0.05237	0.03872	0.04730
Ruminococcus	0.05000	0.02176	0.02974	0.06728	0.02109	0.03893	0.03454	0.02250	0.02634	0.02902	0.02325	0.05569	0.02777	0.03814	0.02599	0.03102
norank_d__Bacteria	0.03242	0.01959	0.02333	0.02271	0.02632	0.02464	0.03447	0.02282	0.02062	0.04659	0.02182	0.02337	0.02468	0.02692	0.02525	0.02812
norank_f__Ruminococcaceae	0.03679	0.01712	0.02866	0.02546	0.02353	0.01926	0.02160	0.01958	0.01477	0.02211	0.01520	0.02232	0.02084	0.01946	0.02032	0.02625
norank_f__Lachnospiraceae	0.03363	0.01825	0.01494	0.01808	0.01588	0.02705	0.03688	0.02073	0.01153	0.02206	0.01563	0.01693	0.01832	0.03098	0.02181	0.02200
Faecalibacterium	0.01687	0.01584	0.01768	0.01435	0.01343	0.03922	0.02341	0.01769	0.00905	0.01492	0.01062	0.01129	0.01351	0.03738	0.01703	0.01647
Eubacterium	0.02027	0.01517	0.01842	0.01692	0.02175	0.01888	0.02015	0.02078	0.01059	0.01825	0.01213	0.01558	0.01734	0.02025	0.01847	0.02253
Lactobacillus	0.00889	0.00900	0.05419	0.00202	0.02211	0.00132	0.00868	0.03231	0.05565	0.05351	0.00193	0.00711	0.00401	0.00159	0.00897	0.00843
norank_o__Bacteroidales	0.01430	0.01685	0.00262	0.02718	0.02647	0.00830	0.01197	0.01989	0.00725	0.01537	0.01165	0.02927	0.02882	0.00868	0.01379	0.01885
Treponema	0.00648	0.00470	0.01093	0.01136	0.01046	0.00342	0.01529	0.01407	0.01241	0.01297	0.01284	0.03572	0.02325	0.00571	0.00991	0.01619
Butyricicoccus	0.00968	0.01366	0.00645	0.01040	0.01673	0.00888	0.00839	0.00870	0.00786	0.02290	0.00852	0.01175	0.01264	0.00919	0.01096	0.01272
Blautia	0.00965	0.01475	0.00745	0.00883	0.01039	0.01639	0.01339	0.01367	0.00561	0.01046	0.00615	0.00836	0.00921	0.01871	0.01484	0.01002
Flavonifractor	0.00896	0.00864	0.00843	0.00818	0.01054	0.01453	0.00994	0.01012	0.00609	0.00942	0.00576	0.00908	0.00917	0.01457	0.01071	0.01056
Roseburia	0.01206	0.01029	0.00953	0.00696	0.00711	0.01342	0.01485	0.00921	0.00462	0.00823	0.00565	0.00626	0.00753	0.01817	0.00910	0.00969
Lachnoclostridium	0.01041	0.00801	0.00883	0.00826	0.00791	0.01344	0.01103	0.00911	0.00521	0.00942	0.00584	0.00847	0.00837	0.01425	0.00966	0.00956
Alistipes	0.00589	0.00928	0.00147	0.01412	0.01299	0.00375	0.00782	0.01133	0.00348	0.00722	0.00551	0.01472	0.02377	0.00464	0.01052	0.00790
Acinetobacter	0.00119	0.00015	0.01691	0.00019	0.00023	0.00037	0.00039	0.00029	0.08118	0.01238	0.01015	0.00024	0.00018	0.00024	0.00193	0.00129
Parabacteroides	0.00462	0.00527	0.00195	0.00911	0.00948	0.01507	0.00389	0.00435	0.00264	0.00754	0.00416	0.01180	0.01212	0.01676	0.00421	0.00931
Fibrobacter	0.01039	0.00908	0.00172	0.00700	0.01244	0.00068	0.00626	0.01062	0.00104	0.00142	0.00226	0.00264	0.01017	0.00074	0.00770	0.01402
Phascolarctobacterium	0.00273	0.01212	0.00362	0.00383	0.01197	0.00710	0.00734	0.00624	0.00576	0.00409	0.00255	0.00395	0.00751	0.00498	0.00621	0.00798
Escherichia	0.00106	0.00087	0.00259	0.00271	0.00342	0.00585	0.00056	0.00614	0.04014	0.00235	0.01662	0.00166	0.00043	0.00269	0.00117	0.00124
Anaerotruncus	0.00557	0.00377	0.00482	0.00479	0.00482	0.01019	0.00558	0.00481	0.00317	0.00406	0.00322	0.00473	0.00451	0.01014	0.00631	0.00497
Corynebacterium	0.00543	0.00092	0.01262	0.00016	0.00034	0.00028	0.00081	0.00195	0.00932	0.02376	0.01486	0.00010	0.00031	0.00023	0.00363	0.00392
Pseudomonas	0.00168	0.00095	0.00239	0.00075	0.00110	0.00130	0.00119	0.00087	0.01800	0.00122	0.03576	0.00095	0.00098	0.00119	0.00316	0.00201
Thiopseudomonas	0.00321	0.00000	0.00277	0.00000	0.00000	0.00001	0.00002	0.00000	0.01450	0.00012	0.04897	0.00001	0.00000	0.00000	0.00010	0.00013
norank_p__Proteobacteria	0.00123	0.00167	0.00305	0.00301	0.00414	0.00379	0.00169	0.00449	0.01468	0.00201	0.01330	0.00238	0.00143	0.00237	0.00261	0.00239
Kurthia	0.00006	0.00000	0.00018	0.00002	0.00002	0.00001	0.00001	0.00000	0.05013	0.00239	0.00009	0.00002	0.00002	0.00001	0.00002	0.00002
norank_p__Planctomycetes	0.00145	0.00138	0.01569	0.00657	0.00101	0.00131	0.00058	0.00152	0.00200	0.00297	0.00060	0.00836	0.00083	0.00121	0.00066	0.00093
Comamonas	0.00089	0.00002	0.00422	0.00002	0.00002	0.00003	0.00003	0.00002	0.00656	0.00070	0.03073	0.00003	0.00001	0.00002	0.00012	0.00004
Lysinibacillus	0.00278	0.00008	0.00652	0.00013	0.00010	0.00014	0.00014	0.00010	0.01761	0.00095	0.00040	0.00012	0.00009	0.00014	0.00029	0.00020
norank_f__Enterobacteriaceae	0.00035	0.00037	0.00107	0.00114	0.00144	0.00249	0.00012	0.00078	0.01103	0.00056	0.00769	0.00064	0.00011	0.00092	0.00017	0.00048
Solibacillus	0.00025	0.00000	0.00102	0.00000	0.00000	0.00000	0.00002	0.00000	0.01947	0.00121	0.00068	0.00001	0.00001	0.00000	0.00005	0.00006
Acidovorax	0.00029	0.00003	0.00112	0.00002	0.00004	0.00004	0.00003	0.00001	0.00233	0.00014	0.01191	0.00002	0.00004	0.00004	0.00005	0.00003
Sporosarcina	0.00006	0.00001	0.00006	0.00002	0.00002	0.00004	0.00003	0.00003	0.01415	0.00016	0.00028	0.00002	0.00003	0.00005	0.00004	0.00003
norank_f__Comamonadaceae	0.00027	0.00001	0.00116	0.00001	0.00001	0.00001	0.00001	0.00000	0.00212	0.00012	0.01115	0.00002	0.00001	0.00001	0.00004	0.00001
Caryophanon	0.00004	0.00001	0.00031	0.00000	0.00001	0.00000	0.00000	0.00000	0.01255	0.00073	0.00003	0.00000	0.00000	0.00000	0.00000	0.00001
Others	0.19022	0.17494	0.21242	0.18529	0.20294	0.20868	0.20158	0.18991	0.17847	0.18636	0.30230	0.17643	0.19198	0.22127	0.21003	0.20731

Table S3: Complete list of fecal metabolites for *Laiwu pigs* and *Lulai pigs*.

Metabolite name	RT(min)	m/z	Ontology	LU-1	LU-2	LU-3	LU-4	LU-5	LU-6	LU-7	LU-8	LW-1	LW-2	LW-3	LW-4	LW-5	LW-6	LW-7	LW-8
3'-Deoxycapsanthin	1.077	568.42621	Xanthophylls	240025	151599	146846	206380	174496	79168	86543	90855	47303	35604	31927	18353	98687	99807	35996	151875
Antheraxanthin	1.094	584.4209	Xanthophylls	67568	58649	86129	59036	35381	46138	27561	19705	26082	34023	18788	8008	29435	40282	24062	31654
Bayogenin	2.126	489.35419	Triterpenoids	93776	82352	149489	106818	14456	4900	42512	35524	1631	11132	41337	3832	73638	8794	11413	8758
3,4-Dihydrospirilloxanthin	4.76	598.45471	Xanthophylls	12043	11944	11056	4526	845	4436	1412	807	1359	1923	2108	83	498	3695	881	1088
Auroxanthin	1.346	600.41907	Tetraterpenoids	2553	4791	1256	33456	20950	28544	19560	25545	1396	2478	1080	7243	11902	10940	1644	7404
Canthaxanthin	1.127	565.39893	Xanthophylls	10043	12283	9630	24260	12838	14947	16234	8083	14007	4566	4288	2533	9973	12397	6235	11659
(-)-Nicotine	3.783	163.12276	Pyrrolidinylpyridines	192032	254643	168138	839	1456	1199	973	4156	7622	36640	373804	1091	1738	1508	1091	1781
(-)-Riboflavin	7.992	377.14154	Flavins	676	3412	626	94179	63101	43866	114711	50488	367	649	233	108257	52501	38913	87049	78741
(+-)-Baclofen	13.168	212.05736	Gamma amino acids and derivatives	100	317	177	9753	381	189	11344	122	47	268	218	661	156	125	884	25909
(+)-Tubocurarine chloride	5.37	609.3197	Diarylethers	216	364	123	23997	3622	155858	2565	12626	97	87	292	6082	2573	106164	13373	3245
(2-oxo-2,3-dihydro-1H-indol-3-yl)acetic acid	6.553	190.05324	Indolyl carboxylic acids and derivatives	1134	1314	271	1869	764	983	5624	38538	326394	1008	299	2040	3295	3156	2463	4706
(2R)-6-methylpiperidine-2-carboxylic acid	6.3	166.07397	L-alpha-amino acids	254	274	96	16692	1927	475	164679	1413	95	78	122	3686	823	1352	27974	149996
(3beta)-lup-20(29)-ene-3,28-diol	1.543	465.3566	Triterpenoids	3407	5178	3062	104171	84923	64651	14025	3239	3143	4099	3961	486	38844	57668	9656	47426
(3beta,22E)-stigmasta-5,22-dien-3-ol	1.553	435.3454	Stigmastanes and derivatives	13944	4006	11112	5044	4003	19522	13900	63575	17055	10200	3902	365	1729	18948	14507	3836
(9Z)-9-octadecenoic acid	3.136	283.26108	Long-chain fatty acids	136780	334825	123577	9685	16410	15162	12983	21887	164143	149581	51193	1981	9200	15524	29933	9163
(9Z,12Z,15Z)-9,12,15-octadecatrienoic acid	4.62	301.19858	Lineolic acids and derivatives	620	409	502	197405	125501	4271	129675	24193	940	475	378	402151	222759	14320	208851	158103
(R)-(-)-Phenylephrine	5.277	168.11264	1-hydroxy-4-unsubstituted benzenoids	16101	25290	1780	499	628	272	560	655	2865	2989	4866	228	345	188	361	631
(Z)-5,8,11-trihydroxyoctadec-9-enoic acid	4.34	329.27167	Long-chain fatty acids	142418	371213	64720	11198	20114	936	3309	4711	46898	64080	58738	4954	24448	900	3989	24515
1-(2-(1H-indol-3-yl)ethyl)urea	13.51	204.12263	3-alkylindoles	292	203	54	2781	12403	2439	4701	1895	99	47	50	5331	1865	1049	2280	3721
1-(3-(Trifluoromethyl)phenyl)piperazine	4.567	231.11885	Phenylpiperazines	36809	25277	7724	1386	941	405	1230	1372	630	456	4268	827	1016	839	1717	684
1-(3,4-Dimethoxycinnamoyl)piperidine	6.496	298.14285	Cinnamic acids and derivatives	209	219	64	176494	205252	100311	185359	206070	93	152	43	201955	159754	87662	220473	120422
1-(4-methoxyphenyl)ethanone	4.619	151.0612	Alkyl-phenylketones	8414	6974	4368	15701	391	641	199675	522	448	44811	2765	3093	1057	311	39943	168348
1,2Alpha-Epoxydeacetoxydihydrogedunin	3.183	455.20416	Limonoids	6881	21497	31782	4856	4726	24538	5596	5100	14041	25753	30547	8946	4485	14230	5428	3758
1,2-Cyclohexanediol	1.881	117.10182	Cyclohexanols	30705	22323	36951	158	161	103	74	96	4586	15609	20838	146	235	246	85	185
1,2-Dierucoyl-sn-glycero-3-phosphocholine	11.674	897.69006	1-acyl-sn-glycero-3-phosphocholines	17879	53561	53527		7	9	7	7	74578	19441	10439	17	6		5	12
1,2-dihydroxyheptadec-16-en-4-yl acetate	2.939	329.26846	Long-chain fatty alcohols	29582	48805	44522	10967	4473	1980	5543	2714	47198	42093	40033	994	3729	2138	9052	5922
1,2-dihydroxyheptadec-16-yn-4-yl acetate	3.1	327.25122	Long-chain fatty alcohols	4344	5594	5693	1743	80815	34516	66232	30575	5992	11072	4666	1904	7664	34694	116206	73908
1,2-Dipalmitoyl-sn-glycero-3-phosphate	3.654	647.48218	1,2-diacylglycerol-3-phosphates	17649	5156	9477	55	636	695	173	1036	4771	3853	6858	817	115	656	433	735
1,3,7-Trimethyluric acid	13.629	230.18475	Xanthines	44	41	78	153023	126643	469136	367183	111129	32	22	31	293877	347306	353818	88859	128652
1,3-Dideacetyl-7-Deacetoxy-7-Oxokhivorin	1.474	457.22559	Naphthopyrans	202	251	127	36834	2963	1123	2319	56739	215	66	313	177720	44055	1654	2214	1286
10-Gingerol	1.025	373.22824	Gingerols	463	843	997	13187	21232	27908	25954	16466	1398	1490	1528	2793	26885	45101	27027	18897
10-hydroxyusambarine	5.535	467.27408	Harmala alkaloids	184	220	242	4573	2004	279	97974	2604	234	50	371	9224	179863	366	589	188789
11,12-Methylenedioxykopsinaline	8.32	399.20471	Aspidofractine alkaloids	8	16	27	53718	123404	95953	18761	202921	17	36	8	11015	139349	15114	165535	4591
11A-Acetoxyprogesterone	14.297	373.2439	Gluco/mineralocorticoids, progestogins and derivatives	212	233	75	276	117803	5304	1877	5114	80	161	124	1431	521	504	382	438

11-Keto-beta-boswellic acid	3.282	471.35092	Triterpenoids	7269	5832	9991	37439	52550	82676	70750	32120	13786	7540	8584	9855	57004	20629	104160	88343
11-Oxomogroside IIIE	6.55	983.50708	Cucurbitacin glycosides	53	21	18	451	249	23945	85	125	7	15	21	3732	139	19002	666	88
12'-Apoastaxanthinal	3.552	380.24353	Diterpenoids	1054	3338	2461	17740	46048	11060	21010	4225	2481	1525	1826	6546	40610	6235	7961	51390
12-epinapelline	1.519	382.23792	Napelline-type diterpenoid alkaloids	5758	14632	4889	36950	36553	14044	30798	82635	11628	7910	2160	1499	20210	11716	30198	12356
13-HODE	4.105	295.22952	Lineolic acids and derivatives	8111	74510	54247	891	3743	8127	8613	1045	27180	211684	287018	1405	1755	1215	13078	7081
13-Oxyingenol dodecanoat	2.884	569.3454	Tigliane and ingenane diterpenoids	134905	104776	72635	10864	18260	18406	22346	4475	5873	69201	33290	9410	33648	12282	8652	63943
14,15beta-Dihydroxyklaineanone	3.094	397.17999	Quassinoids	641	1861	1626	3193	2320	12737	1531	25707	2072	487	1145	3232	7861	57092	5512	1649
16-Hydroxyhexadecanoic acid	3.938	271.22897	Long-chain fatty acids	1106557	2130871	2138501	976	8865	6443	2784	944	635167	1389861	94460	320	1239	7069	2996	1414
17-HDoHE	5.338	343.21414	Very long-chain fatty acids	4020	11090	4527	21666	33434	22898	28141	75256	38237	7141	4328	32880	38110	6805	38536	40488
18-Aminoabieta-8,11,13-Triene Sulfate	3.354	406.20389	Diterpenoids	29802	41356	20607	4831	6482	6710	5958	6922	16946	37689	49563	7198	3350	4069	5797	4828
19-O-Methylsiphonaxanthin	2.847	614.43756	Xanthophylls	1960	15879	2303	357	1457	227	2579	4278	20156	2384	4071	931	171	3135	2752	1600
19S-Methoxytubotaiwine	1.096	353.20477	Strychnos alkaloids	5184	3896	4560	14883	8028	23236	7553	4552	14116	7389	12314	4208	14374	4396	11860	9621
1-Cinnamoylpyrrolidine	1.093	202.12331	Cinnamic acids and derivatives	28824	10311	78924	1209380	1346942	2703940	2329445	914027	7516	48430	12635	1437175	1906212	1678264	1195854	1740767
1-Hexylamine	16.912	102.12714	Monoalkylamines	22883	29224	13804	470	504	425	379	437	28366	27444	28000	301	244	493	560	158
1h-indole-3-butanoic acid	10.262	204.08717	3-alkylindoles	80	76	138	1374	1196	52658	1408	4298	143	117	172	256	410	97124	898	270
1H-indole-3-carboxamide	3.446	161.07001	Indolecarboxamides and derivatives	4880	4627	2888	491	340	1068	7155	594	170424	2964	627	346	173	249	2636	9354
1-hydroxyanthraquinone	6.493	223.02875	Anthraquinones	139	16	73	38175	26395	3769	22489	27518	98	131	120	27866	819	4401	32961	22497
1-Isothiocyanato-9-(methylsulfinyl)-nonane	5.479	248.11389	Sulfoxides	40756	36301	4696	389906	383616	134308	264996	297120	50060	24749	10585	314044	197410	101328	287704	200702
1-Methylguanosine	3.069	296.09454	Purine nucleosides	93	90	169	17890	45656	17544	23829	76939	465	207	230	39037	82977	23903	28175	29337
1-Methylnicotinamide	11.281	137.07079	Nicotinamides	31367	18492	1495	46	41	30	23	163	80	1586	1076	106	35	50	254	41
1-Myristoyl-2-hydroxy-sn-glycero-3-phosphate	6.44	381.19092	1-acylglycerol-3-phosphates	907	410	330	115517	121941	42057	106798	89869	890	745	353	121203	82395	39273	148902	87503
1-Naphthol-5-sulfonic acid	6.186	223.02612	1-naphthalene sulfonates	100	55	76	1060	1124	646	1558	1149	289	42	16	502	886	21585	41401	1426
2-(1H-indol-3-yl)ethanamine	12.784	161.09433	Tryptamines and derivatives	381	62	61	1932	291	7645	62635	306	128	328	204	288	3330	100	6647	20288
2-(4-hydroxyphenyl)-4H-chromen-4-one	13.781	239.0607	Monohydroxyflavonoids	19783	23357	17578	92	196	113	88	98	32465	20475	22656	150	349	100	90	321
2-(Perfluorotetradecyl)ethan-1-ol	13.34	762.96411	PFSA	5237	4597	4557	5	2	8	12	4	9651	5555	19133	5	9	6	4	7
2,2`,4,4`-Tetrahydroxybenzophenone	6.063	245.03604	Benzophenones	19	19	132	1631	1122	35857	2219	51292	125	32	46	653	427	33206	2316	2106
2',2'-Difluoro-2'-deoxyuridine	4.919	263.04297	Pyrimidine 2'-deoxyribonucleosides	19836	8995	5764	58490	16641	3548	17410	14630	2812	6230	3650	11933	27527	5511	17004	89832
2,3-dihydro-1H-carbazol-4(9H)-one	6.192	186.1133	Carbazoles	55	84	66	200878	133707	514512	154736	100618	75	154	111	87438	47445	442908	144278	141161
2,3-dihydromicrocolin D	5.35	730.55914		7828	22038	5353	941	5896	920	4400	3063	2032	3752	7350	63	761	1797	2800	378
2,3-Dihydroxybiphenyl	13.592	185.05551	Biphenyls and derivatives	20098	20641	26759	72	31	54	102	40	10799	18758	21626	76	35	63	12	103
2,3-dihydroxypropyl palmitate	1.497	353.26413	1-monoacylglycerols	66010	64522	93811	45848	37105	39217	40615	51115	78510	55226	43270	1433	22886	32546	48823	37239
2,3-dihydroxypropyl stearate	9.04	381.3075	1-monoacylglycerols	2	2	2	15133	31980	8048	14675	26305	3	3	3	8799	27962	8174	18860	12935
2',4',6'-Trihydroxydihydrochalcone	4.708	257.08447		8461	31566	9266	35216	12017	1552	2697	294	520	7614	3682	40845	12695	1532	461	16898
2,4-Diaminobenzenesulfonic acid	0.862	187.00789	NA	1291585	1241287	842794	13370	822	713	3308	550	6146	1579792	482763	56452	5794	1243	3368	17170
2',4-Dihydroxy-3,4',6'-Trimethoxychalcone	10.259	329.10486		62	196	142	6827404	3089801	1096874	2492992	1545301	80	101	49	6470800	3135170	873450	2421729	3298917
2,5-Dichlorolichexanthone	13.761	355.01053		189033	312776	154858	124	173	48	318	27	234230	180191	143440	240	179	124	201	151
2,6-dihydroxyanthraquinone	6.463	239.0401		372	45	255	56758	11589	8661	9285	6829	106	306	398	81911	10269	4729	10935	4304

2,6-di-tert-butyl-4-methylphenol	17.378	219.17714	Phenylpropanes	25232	42727	36642	11440	7213	8082	7929	16229	51350	100263	27532	9289	8480	10291	9830	12043
2,8-Quinolinediol	4.341	162.05455	Quinolones and derivatives	3091851	1229134	719411	117113	21755630	241112	1575072	15093226	2010136	659854	337161	710250	1209059	914349	1590670	1291690
2-[(1S)-1-hydroxyethyl]-1H-quinazolin-4-one	4.104	191.08282		282639	263907	243467	232806	107931	87183	226755	95293	243346	170754	94879	56270	160125	81751	209868	106667
24-epimakisterone A	4.872	495.31747	Hydroxy bile acids, alcohols and derivatives	4865	15900	6691	1396	4182	2197	373	1770	15181	4150	8310	8670	1842	1843	600	1330
2-acetoxy-6-pentadecylbenzoic acid	4.109	389.27347	Acylsalicylic acids	2331948	465191	2551354	2849	42360	62465	36172	31684	1049457	786537	5096445	49400	16306	54664	123195	59821
2-Aminophenol	9.784	108.05603	Aniline and substituted anilines	6	3	11	14972	13665	1420	16341	7936	3	12	14	22874	13346	2031	19792	11274
2-Benzoxazolinone	4.172	136.03899	Benzoxazolones	69052	18406	51881	254	137	578	25962	248	173	87792	19219	6888	817	150	4534	12964
2-Chloro-6-O-methylnorlichexanthone	11.45	305.02722		3419008	3549540	557922	92	27	44	62	39	5758384	743885	912315	57	35	123	100	227
2-Chloronorlichexanthone	11.41	291.01328		37626	53695	34957	18	8	31	26	9	5260	5220	9819	87	12	41	45	31
2-Hydroxy-4-methylpentanoate	4.996	131.07025	Hydroxy fatty acids	3597	135601	769	3636	34371	19125	88497	5126	3409	2137	6640	5452	3694	10243	83535	74347
2-Hydroxybenzaldehyde	4.248	121.03076	Hydroxybenzaldehydes	718546	720293	222316	57205	8788	693	54209	5602	18027	303976	128827	9866	24280	1459	14521	38267
2-Hydroxyisobutyric acid	6.106	103.04166	Alpha hydroxy acids and derivatives	11	51	23	4836	4892	5930	30609	14613	6	8	6	4675	190247	73211	428265	56763
2-Hydroxyphenylacetic acid	3.239	151.04247	2(hydroxyphenyl)acetic acids	2542	2854	1152	3708	22621	13668	40697	15997	3502	2939	2423	29959	35705	19450	35998	37989
2-Methoxycinnamic acid	8.684	177.06995	Coumaric acid and derivatives	3	79	114	496	1315	1718	511	48002	6	3		49	357	2235	661	1177
2-Methylglutaric acid	12.357	145.05151	Methyl-branched fatty acids	566	411	789	13564	14809	2604	10008	25386	625	645	528	270	3463	2998	11430	24165
2-Methylpyrrolidine	10.705	86.09592	Pyrrolidines	320	608	226	445	485	732	91550	1357	225	280	324	42207	6464	775	1465	49422
2-Naphthalenesulfonic acid	3.474	207.00005	2-naphthalene sulfonates	2216	961	422	29988	124315	71504	7746	164978	948	1857	825	198390	90668	100895	149593	62183
2-O-Acetyl-20-hydroxyecdysone	4.2	523.32391	Pentahydroxy bile acids, alcohols and derivatives	1430	6715	4385	11761	2019	154665	8983	7958	2720	3576	3571	11394	4515	153400	7530	9044
2-O-E-p-coumaroyl alphitolic acid	3.478	617.40399		3310	1094	2380	1695	8002	1546	4241	3105	611	348	1019	8111	981	291	6144	16678
2'-O-Methyl-5-methyluridine	4.855	271.09454	Pyrimidine nucleosides	2385	900	580	3526	298	551	837	328	359	19702	8734	3811	1403	263	782	551
2'-O-Methylcytidine	11.956	256.08453	Pyrimidine nucleosides	62	84	64	30842	13685	7849	12742	262	64		100	18346	6486	3814	4579	4974
2''-O-rhamnosyl icariside II	10.195	683.19733	Flavonoid-3-O-glycosides	2		8	66981	19996	4620	18007	12993	8	8	10	41929	36604	5335	24544	36751
2-Piperidone	2.436	100.07618	Piperidinones	164	170	68	208560	3643	3864	3082	6007	409	237	368	416	1821	755	39439	6680
2-Propanamidoacetic acid	7.412	130.05444	N-acyl-alpha amino acids	2		3	221	1383	695	8049	1242	3	7	12	27898	16632	1005	874	4493
3-(2-Hydroxyphenyl)propanoic acid	4.541	165.05855	Phenylpropanoic acids	30364	569849	270453	15160	7812	16713	463259	5477	78026	99661	9865	49309	62357	2373	114822	486686
3-(3-Hydroxyphenyl)propionic acid	4.399	165.05913	Phenylpropanoic acids	44124	537054	22992	59962	12550	5090	70466	22193	78026	10973	8628	110669	167661	2227	53029	84918
3,3'-Di-O-methylellagic acid	13.858	353.02359	Hydrolyzable tannins	134728	113920	137887	129	1313	559	1902	22	106965	135162	133468	74	55	147	757	46
3',4',5',6'-tetradehydrogeissospermine	11.162	629.35162		77	162	88	6223	3459	51983	5805	1647	82	46	78	8852	2668	27199	4749	2533
3,4,5-trimethoxycinnamic acid	6.144	237.02373	Coumaric acids and derivatives	30	47	92	360118	272441	404375	158504	265005	231	170	37	390255	185890	421989	217579	185302
3,4-Dichlorophenylurea	13.523	204.99115	Dichlorobenzenes	20873	17423	26300	99	107	25	35	19	22038	35918	39318	271	66	77	37	107
3',4'-Dimethoxy-7-hydroxyflavone	3.67	297.07565	4'-O-methylated flavonoids	50437	171652	19671	69148	167975	103242	229756	93853	221311	129126	30053	127542	114164	100857	261697	121664
3,4-Dimethoxybenzoic acid	6.428	183.18216	P-methoxybenzoic acids and derivatives	12446	16790	14207	98	52	168		119	13767	20252	27539		21	145	41	
3,4-Methylenedioxyethamphetamine	1.634	208.13326	Benzodioxoles	79193	181	211	33		166	460	76	103	171	59	48	37	144	150	150
3,5-Dicaffeoylquininic acid	11.173	515.09363	Quinic acids and derivatives	140	55	45	12	50	72	54	44	10829	20172	8190	33	94	22	53	56
3,5-dihydroxydecanoic acid	6.721	227.12317	Medium-chain hydroxy acids and derivatives	897	313	110	893678	122005	18125	1342765	14307	85	221	292	1114027	120819	14204	539048	1257843
3,9-Dimethoxypterocarpan	4.59	283.09937		78679	145845	31508	110933	24470	3096	22428	6292	47082	134187	40475	124969	35895	4616	17281	24984

3-[(1-Carboxyvinyl)oxy]benzoic acid	13.481	247.00197	Phenoxyacetic acid derivatives	120071	72212	90125	195	159	74	236	14	175956	99218	232996	211	136	201	164	129
3-[(Z)-heptadec-10-enyl]benzene-1,2-diol	3.362	369.26657	Catechols	34793	21595	48500	27508	4474	21406	28764	40583	31808	41562	26557	5957	9573	15360	20791	18319
3-Acetyl-11-keto-beta-boswellic Acid	3.56	535.34778	Triterpenoids	2092	2979	1994	11589	38909	19180	24026	20497	1645	1806	2689	16090	44980	15790	30306	28658
3-Epilupeol	3.218	425.3605	Triterpenoids	12577	6603	5017	46	208	66	199	103	4899	2585	249	196	147	59	125	240
3-Genistein-8-C-glucoside	3.182	431.17633	Isoflavonoid C-glycosides	5741	6266	3449	64428	72710	33680	106340	55256	3505	4949	4676	128576	82235	49393	105736	72945
3-Hexen-1-ol O-b-D-glucopyranoside	13.149	263.14697	Fatty acyl glycosides of mono- and disaccharides	46	97	187	18852	1763	1123	18520	744	98	53	73	4510	1480	466	1254	51127
3-hydroxy-1-(4-hydroxyphenyl)propan-1-one	5.479	167.09177	Alkyl-phenylketones	7539	2448	3223	66258	32470	20353	142701	30546	1543	2109	1274	25420	20925	5039	80949	124155
3-hydroxy-2-octylpentanedioic acid	6.6	259.15726	Medium-chain hydroxy acids and derivatives	504	297	386	19492	218697	19106	412036	546119	52	33	95	934079	115696	40997	25954	29392
3-Hydroxyaspartic acid	6.459	148.02776	Aspartic acid and derivatives	22045	6430	4335	111	81	58	24	29	66	9279	12331	140	26	155	50	251
3-hydroxybenzo(a)pyrene	3.431	267.0918	Benzopyrenes	132776	45953	3869	6425	14741	9168	52041	6426	1450	4271	1766	23286	9442	9075	26421	45894
3-HYDROXYBUTANOIC ACID	6.226	103.04147	Beta hydroxy acids and derivatives	11	24	4	63455	104271	165932	380742	335716	4	8	7	204507	189878	73023	428320	151110
3-Indoxyl sulfate	1.444	212.00449	Arylsulfates	382570	320523	450801	299790	166	89	551505	207	1712	706551	178503	4923	405	74	3849	7024
3-methyl-2-(2-nonen-1-yl)-4-Quinolinol	3.203	284.20654		37901	103312	101234	380	773	725	294	259	45422	234513	7758	493	742	370	349	353
3-Methyladipic acid	12.504	159.06763	Medium-chain fatty acids	86	56	66	1210482	1043991	347218	468314	234414	169	246	129	852363	399124	119104	165178	318062
3-Methyloxyindole	1.225	148.07791	Indolines	1237	1053	240	1197	1128	2374	4764	1716	341	410	1040	23000	1025	1436	4947	3017
3-Nitrotyrosine	13.592	225.05286	Tyrosine and derivatives	20476	21792	23028	86	87	83	33	105	15745	20167	20503	26	43	12	28	131
3-O-Acetyl-16alpha-hydroxytrametenolic acid	4.7	537.33533	Triterpenoids	2660	3064	5969	20022	51396	14383	53991	25823	1207	6008	5051	86521	22767	4695	64737	21571
3-Phenyllactic acid	4.742	165.05812	Phenylpropanoic acids	16165	460547	11920	2552	16899	16284	222138	2973	9141	48412	4715	3593	9784	2289	34664	215107
4-(1H-indol-3-yl)butan-2-one	1.546	188.1075	3-alkylindoles	972	223	307	49736	98354	236317	150647	170134	90	53	116	2364	191613	229333	416484	213566
4-(sec-butoxy)benzoic acid	13.784	217.08281	Benzoic acids	73046	74342	71029	125	239	179	123	141	111584	83077	88400	432	586	556	326	157
4',5,7-trihydroxy-3,6-dimethoxyflavone	14.596	328.98489	6-O-methylated flavonoids	51	35	50	15096	40529	12017	42356	16769	44	16	26	38587	29713	2323	32496	44459
4,5-dihydroxy-3-propylcyclopent-2-en-1-one	5.642	155.04745	1,2-diols	995	490	79	3809	1249	876	72734	1566	113	340	1046	2611	1403	1017	18729	50880
4:2 Fluorotelomer alcohol	14.597	263.01562	PFSA	37	54	92	17531	50359	12686	59425	18224	26	16	26	72233	30485	2690	38890	85645
4-Amino-N,N-dimethylbenzenesulfonamide	6.498	201.07178	NA	1598	242	411	38923	57707	24515	49852	23068	890	629	1120	66856	100807	38930	110038	126147
4-aminovaleric acid betaine	9.874	160.13379	NA	57	63	44	5187	43824	13171	76573	6180	16	56	17	73316	22332	14408	28469	55085
4-epivulgarin	3.974	287.12509	Eudesmanolides, secoeudesmanolides, and derivatives	767	311	416	739	705	2283	2414	610	381	507	401	92257	936	3235	4055	9596
4-Guanidinobutyric acid	13.522	146.09137	Gamma amino acids and derivatives	62	269	339	15616	7098	3304	7323	2148	638	339	19	5559	5551	1511	3964	6691
4'-hydroxy-2',4,6'-trimethoxychalcone	2.921	315.12408	Cinnamylphenols	1690	1221	17305	8835	113522	127250	136224	152101	379	3098	1257	3361	82164	127900	184131	77075
4-Hydroxy-3-methoxycinnamaldehyde	0.986	179.06262	Methoxyphenols	40040	24055	36092	386	306	886	400	476	14085	22474	48901	1286	248	90	26	305
4-hydroxy-3-tetratrenylbenzoic acid	9.451	411.3009		37	39	34	660	530	395	16592	483	19	79	45	1261	588	339	272	15484
4-Hydroxybenzoic acid	5.254	137.02718	Hydroxybenzoic acid derivatives	22181	7306	4496	20488	9661	11134	3810	2303	1278	11164	7177	32299	24719	12170	8614	10192
4-Hydroxyhippuric acid	8.734	194.04688	Hippuric acids	328	7152	5935	2527	84	41	324	1824	15	9821	8	2025	31	50	155	697
4-Hydroxyquinoline	1.577	146.05975	Hydroquinolones	2202	1289	2119	8024	2012091	109720	221869	855002	1630	3879	2801	164912	15417	196824	191618	60906
4-methoxy-9H-xanthen-9-one	13.596	249.0441	Xanthones	15594	15275	17064	40	25	4	68	22	9836	13133	15315	14	14	29	17	35
4-METHYL-5-THIAZOLEETHANOL	4.224	144.04375	4,5-disubstituted thiazoles	19461	791	2164	2259	109276	1158	4868	97975	8422	118	197	2093	4386	2288	6131	6823

4-Methylabyssinone V	1.074	421.1897		2977	13633	2486	109605	64209	26925	44225	78144	8917	1328	1704	249693	53911	23473	92295	66597
4-nitroquinoline 1-oxide	5.702	189.0341	4-nitroquinoline N-oxides	251	140	184	60121	67605	25234	374	51613	257	173	216	53012	38642	28829	50821	469
4-oxo-5-phenylpentanoic acid	13.65	210.11224	Medium-chain keto acids and derivatives	1667	3173	2003	101430	116895	37986	91392	61639	4043	1484	1180	125265	87654	13672	40527	29408
4-Pyridoxate	7.092	182.03389	Pyridinecarboxylic acids	18	42	26	6754	8777	2343	24827	4479	5	2	9	2790	6630	3103	9945	46021
4-Pyridoxic acid	2.956	182.04755	Pyridinecarboxylic acids	213914	267570	57097	37466	12469	34483	113683	219279	210567	150134	40181	12050	5054	37241	68683	104332
4-Quinolinecarboxylic acid	6.574	174.05692	Quinoline carboxylic acids	151	140	36	192	4909	399	3703	4993	17825	66	91	205	1889	160	5773	4351
5-(8,11,14-pentadecatrienyl)resorcinol	1.11	313.26465	Resorcinols	641418	578958	429835	752720	738031	798074	772042	768647	650356	605512	448321	452110	697660	883601	701496	730476
5,6-Dihydro-5-methyluracil	13.517	129.06413	Hydropyrimidines	646	2963	806	19016	636	1406	18444	145	880	421	750	1902	265	224	2525	16412
5,6-Dihydroxylutein	1.433	602.43195	Xanthophylls	24852	23878	27085	54355	63724	56078	45362	52893	16024	16010	10859	14329	48389	54671	27906	39151
5,7-DIHYDROXYISOFLAVONE	5.414	253.10962	Isoflavones	2984	3533	652	19351	19892	21053	21112	20858	9683	1446	622	25348	16466	19991	37636	19450
5Alpha-Cholestanol	3.164	387.25308	Cholesterols and derivatives	120	235	97	201	1053230	150560	413338	98052	612	250	626	472610	397454	195070	351294	310585
5-Aminoimidazole-4-carboxamide	3.806	125.03788	Aminoimidazoles	4984	81192	1555	2263	11390	619	10688	1314	1724	4946	3288	3555	967	349	5956	9641
5-Aminonaphthalene-2-sulfonic acid	6.639	222.03847	2-naphthalene sulfonates	32	26	41	2637	4899	1784	26398	327827	494	117	9	1092	4371	1172	9548	10691
5-Aminosalicylic??acid	6.909	152.03992	Aminobenzoic acids	5	30	57	2656	670	1264	32569	393	45	59	35	600	2762	411	7245	12692
5-Aminovaleric acid betaine	13.834	160.13158	NA	325	114	351	416657	625393	1286723	732395	479666	1228	282	72	271626	768545	1349978	440450	794858
5-Demethylnobiletin	3.262	389.26825	8-O-methylated flavonoids	55331	203188	745228	274931	944637	753420	984482	474819	84589	158070	153166	27500	521633	866492	756098	839403
5-Hydroxyindoleacetic acid	6.644	192.06381	Indole-3-acetic acid derivatives	100	44	25	475	13265	5292	9319	80360	1141	47	30	1460	5765	10257	14002	10814
5-Hydroxymethylcytidine	13.163	272.07767	NA	64	28	53	6491	459	94	6474	56	34		130	1005	40	167	455	22188
5-Methyl-1H-benzotriazole	4.141	132.05185	Benzotriazoles	4433	2718	3786	4195	730	31893	104434	22246	610	8310	2980	195	1011	8691	132928	155702
5-O-Caffeoylquinic acid methyl ester	5.01	367.09262	Quinic acids and derivatives	35384	79279	23087	4803	88592	7851	88770	120398	66176	28871	6024	5339	77187	17263	107401	58930
5-O-methyllicoricidin	1.041	437.242	7-O-methylated isoflavonoids	2532	2894	4529	199	90	566	143	114	9430	4570	13601	289	135	410	275	121
5'-S-Methylthioadenosine	4.687	298.09348	5'-deoxy-5'-thionucleosides	82357	78823	14843	4418	1426	551	1867	295	70915	59094	23709	15379	3007	14735	6030	14395
6-(methylamino)hexane-1,2,3,4,5-pentaol	1.567	196.13419	Hexoses	288	1680	3777	4716	1368	17364	4523	800	303	615	13312	59810	4540	21400	3420	4786
6,8-Dimethyl-4-hydroxycoumarin	5.863	189.03136	4-hydroxycoumarins	179	101	42	9218	13967	7803	35812	11717	596	69	132	7497	15158	3107	26940	24393
6-Hydroxyflavone	3.453	237.02063	Flavones	876	474	446	56790	124660	249599	11619	145313	2232	1387	179	148043	78577	309401	182115	33939
6-Hydroxysumatrol	3.31	425.13596	Rotenones	3577	3915	6860	12829	85605	6634	5690	61864	4694	1951	771	8291	15743	4865	3636	7378
6-methoxy-4-methyl-2H-chromen-2-one	4.267	191.08212	Coumarins and derivatives	23139	23828	22862	212737	25275	49340	73747	19027	18978	15617	17365	167509	77693	67335	230519	144369
7,3',4'-Trihydroxyflavone	3.105	269.04938	Flavones	10514	6840	4367	790	1238	1065	1997	1393	1509	117847	12883	2997	1488	1250	1641	411
7,4'-Dimethoxy-3-hydroxyflavone	5.658	297.07535	Flavonols	444	1118	482	39716	12134	502	656	4043	584	444	256	30481	14373	1400	15072	2260
7,4'-DIMETHOXYISOFLAVONE	4.639	281.07822	7-O-methylisoflavones	26878	53923	27330	12972	4394	4202	8915	5037	4924	49642	10670	46862	4955	1629	8563	7792
7,4-Di-O-methylapigenin	3.666	299.0889	7-O-methylated flavonoids	37543	185229	59893	151296	168064	118167	195756	158571	123856	143406	38040	9578	160030	113609	230427	123258
7,8-dimethoxy-benzo[d][1,2]oxazin-1-one	8.843	230.04042	Benzoxazines		46	4	75	1462	1168	1361	134882	514	18	13	185	68	282	238	487
7,8-dimethylalloxazine (lumichrome)	3.478	243.08542		22109	5432	6849	4121	9060	783	25963	2650	130427	2954	4496	715	702	997	968	3062
7-Deacetoxy-7-Oxokhivorin	3.221	541.2403	Naphthopyrans	557	1370	615	3668	5010	11812	3940	7840	2747	2095	86729	7253	6359	21096	4568	1454
7-Diethylamino-4-methylcoumarin	1.441	232.133	Coumarins and derivatives	73	76	64	21172	37356	105921	51972	62502	36	54	225	22210	71452	87865	116911	51295
7-Hydroxymitragynine	8.432	415.23697	Corynanthean-type alkaloids	8	32	12	34527	26306	45476	47124	35853	22	24	8	1568	50400	42276	56732	42578
7-methanesulfinylheptanenitrile	1.566	174.09196	Glucosinolate breakdown metabolites	120	81	431	80933	93551	181787	524384	138387	1017	379	586	2903	167010	132503	465915	440940

7-Methylguanosine	3.05	298.11029	Purine nucleosides	1357	158	1108	22487	57189	21362	29277	63938	688	1091	162	16780	71204	22693	43368	34124
7-Oxocholesterol	1.588	401.33948	Cholesterols and derivatives	8367	4887	24395	15674	2404	24471	34952	265395	10598	968	1972	118	16092	22860	36919	3254
8,9-epoxy-3,10-diisobutyryloxythymol	6.393	321.18063	Phenol esters	2750	5620	3624	635904	522700	3176127	496750	663836	6643	1437	5987	466102	279591	2501970	604327	426043
8:1+2O fatty acyl hexoside	6.284	321.14627	Fatty acyl hexosides	13727	22473	16635	895	2268	4068	1172	3181	43551	17837	9295	3664	1770	3050	405	2114
8'-Apocapsorbinal	4.364	448.30078	Triterpenoids	2514	3187	5140	5200	9236	63347	16241	16343	2777	3662	1636	3404	43763	73788	6645	11682
8'-Apozeaxanthinal	7.259	432.30569	Triterpenoids	140	344	81	17573	17810	394506	30570	29309	128	25	35	3232	43151	130374	28915	26816
8Beta-Hydroxycarapin, 3,8-Hemiacetal	4.481	483.21375	Limonoids	2691	3791	5676	96143	33360	8694	6046	30382	3873	2556	2046	57303	50664	7570	22281	12881
8-Methylsulfinyloctyl isothiocyanate	6.14	231.08208	Sulfoxides	76	268	126	59231	2075	764	5456	1596	207	568	86	69828	1769	1470	4321	2218
8phiC8SPC (STANDARD)	4.951	299.09024	NA	1296	2364	1001	65765	17095	5338	21354	9406	729	588	1599	60045	16961	1702	25292	24972
8-Prenylnaringenin	3.7	339.13916	8-prenylated flavanones	7937	18951	10220	148341	153201	149600	93227	154866	1787	10097	731	145934	83012	100242	112447	85898
9,10-DiHOME	3.349	313.24026	Long-chain fatty acids	109936	187418	66443	13787	28575	10859	18151	27130	63236	77067	96458	4474	7275	14104	40192	31108
9-methoxycarbonyldec-9-enoic acid	3.071	229.14438	Medium-chain fatty acids	674	434	257	547	56710	41412	12810	9308	784	487	463	7888	15024	36746	20268	10804
9-Nitro-20(S)-camptothecin	10.254	392.10025	Carbolines	31	51	20	35431	56276	22044	36575	27368	58	18	20	27137	56597	12492	33713	37916
9-Trans-Palmitelaidic acid	1.569	253.22067	Long-chain fatty acids	50	37	79	16977	10561	20178	25712	12455	119	53	22	7341	12561	14042	23795	22789
ABAMECTIN	5.407	895.53015	Macrolides and analogues	20		5	3650	4055	914	176	3570	24	8	6	162	15490	774	514	87
Abietic acid	10.194	301.20416	Diterpenoids	5111	4976	44614	867	2990	1711	2961	1113	181	104	86	2784	769	1064	3047	1810
Abruquinone B	10.211	413.10727	Isoflavanquinones	11	17	26	28082	27087	7428	5103	21964	19	18	13	22770	19882	18186	47230	49875
Acacetin Diacetate	8.076	367.07837	4'-O-methylated flavonoids	9455	23502	9480	246	85	99	75	130	16752	8548	4953	228	299	137	216	160
Acamprosate	4.151	180.01736	Organosulfonic acids	5792	2978	3978	20635	1200	63995	174171	65386	1099	10708	4350	190	728	10021	181948	196053
Acebutolol HCl	4.286	337.21271	Alkyl-phenylketones	157652	337020	356125	82679	184426	5290	112388	12918	851694	256523	280230	20324	101058	7875	73380	129902
Acepromazine	13.794	327.15393	Phenothiazines	6762	2534	14107	21353	22563	3460	8362	865	1465	18145	8210	26012	14019	1338	3974	10326
ACETYL ISOGAMBOGIC ACID	8.74	671.32104		90	55	50	553	570	53397	869	617	117	51	26	2543	203	17197	649	532
Acetylcarnitine	11.518	204.12248	Acyl carnitines			144	715216	20560	5279	236662	9531	344	104	320	11895	3679	655	10518	1299426
Acetylcholine	13.753	146.11623	Acyl cholines	464	350	220	691897	872665	1453610	918184	1067997	1876	407	298	409763	747054	1469820	624329	956076
Acitretin	13.91	327.18982	Retinoids	37	79	83	117667	78687	32124	37032	37102	52	281	312	126244	54658	18141	53208	26470
Aconine	8.008	500.27704	Aconitane-type diterpenoid alkaloids	10	29	35	5480	17560	2091	122382	7035	37	17	24	440	8738	2651	3650	3078
ACTINONIN	1.503	403.29745	N-acyl-alpha amino acids and derivatives	1643	4784	2887	8308	6237	26520	5257	11078	1790	554	1936	867	5004	60846	6772	12443
Adenine	6.327	134.04927	6-aminopurines	4677	9306	247	56155	150788	4159	98983	146536	26723	1344	1606	113262	109487	6457	66125	218562
Adenosine	6.611	268.10272		1031	2524	677	58930	48982	38802	76956	160618	1073	1437	152	52899	111275	23405	54551	28782
Adipic acid	13.08	145.05237	Medium-chain fatty acids	646	1694	681	165202	118215	91862	107663	74633	481	1239	1003	219425	66990	24919	94766	128520
Adonirubin	1.615	580.39392	Xanthophylls	1568	1143	1221	933	7458	5455	2377	25870	920	1016	414	530	1694	7569	582	3994
afzelechin	5.812	273.13782	Flavan-3-ols	159	69	85	139237	78975	8132	57965	85940	246	95	82	193832	95923	14551	98661	51692
agnuside	3.214	465.20346	Iridoid O-glycosides	1000	1215	2576	7856	6728	57544	5454	36184	1281	1397	431	7359	13353	62273	16074	7711
Alachlor ESA	1.483	314.10974	Anilides	82	69	1103	21622	152355	61133	144968	20140	90	573	698	238620	353592	51309	13075	130855
Alanine	12.95	88.04062	Alanine and derivatives	74	176	78	51571	6605	13153	111220	8901	206	139	247	17513	6130	3425	45754	53623
Alanine betaine	9.683	132.10136	Alanine and derivatives	13458	341	2016	679	619	460	3215	231	12155	42261	14987	480	330	616	717	3374
Alisol B Acetate	7.689	515.36426	Triterpenoids	28	30	22	28069	38128	19133	2283	24659	27	5	10	324	2661	26165	4179	981
Alisol A,24-acetate	1.522	533.37067	Triterpenoids	20666	27672	26295	9012	20947	17872	11388	6109	6121	7277	13264	1424	7405	20261	15582	10624

Alisol C Monoacetate	2.886	551.33807	Triterpenoids	176693	134088	87076	16046	26822	25737	32942	6142	8328	79777	26965	14062	44316	17009	7207	86244
ALLANTOIN	7.036	157.03667	Imidazoles	20	1821	3194	29939	2098	501	4453	1237	16	6479	311	54168	2033	144	1847	3105
Allopregnanolone	3.844	339.29297		21619	89399	14497	467	444	552	319	184	1025	6564	3203	158	267	830	116	180
Aloe-emodin	3.318	271.06259	Anthraquinones	19630	2366	173220	87	199	1570	11209	1046	2401	22135	3963	261	225	381	2053	845
alpha,beta-Dihydroresveratrol	9.114	229.08946	Stilbenes	30	32	75	2258	2127	722	1505	12645	149	47	43	2012	1183	1652	1541	686
Alpha-Ergocryptine	2.915	494.33871	Lysergamides	3018	4934	4439	16072	8456	3857	8960	15478	7025	6783	2525	5996	5636	3414	22115	5776
alpha-guaiaconic acid	4.695	339.13986	2,5-diphenylfurans	903	996	575	2042	5502	2461	3540	58323	565	386	551	2684	2142	1749	3569	2559
alpha-Hederin	4.045	749.46338	Triterpenoids	1160	5681	869	3975	15938	4175	6475	16784	3458	264	679	4507	6841	4398	21367	4024
Alpha-Mangostin	3.176	409.25641	8-prenylated xanthones	37207	88003	154367	6909	1407	2225	543	872	54591	97644	120203	2568	1470	2730	423	938
Alpha-pyrrolidinovalerophenone	0.914	232.16812	Alkyl-phenylketones	18259	13447	18897	2560	2621	4105	4282	3823	10184	16901	20524	2042	3173	4175	4821	2619
alpha-TOCHOPHERYL ACETATE	0.839	490.42673	Vitamin E compounds	87283	48500	52446	9769	11969	14177	11586	7747	32542	10359	6474	7608	10810	19710	7265	11620
Altenuene	13.96	315.06647	2-benzopyrans	1657888	2024976	1621119	32	2	36	29	18	2069187	893304	1797713	11	28	19	23	5
Altertoxin I	11.153	353.09833	Perylenequinones	10524	24062	16281	1910	1409	385	1202	854	19725	10409	9794	2819	1087	1218	1586	2578
Ambroxol	13.765	376.99417	Phenylmethylamines	43364	71062	36890	6	85	100	85	108	50127	42432	36683	74	86	343	71	30
Aminocaproic acid	13.653	132.10133	Medium-chain fatty acids	27	30	69	2363	3471	3177	5815	1542	122	10	69	14503	8643	2222	2781	1746
Amino-nitro-toluene	4.162	153.06544	Others	208989	67750	174808	15634	1939	1234	18035	1118	11175	333145	68802	19833	3118	1648	31864	68222
Amphotericin B	11.455	925.01141	Aminoglycosides	989732	97343	68779	6		57		15	1816829	1331355	978880	6	4	27	15	
Anabasamine	8.506	254.15382	Bipyridines and oligopyridines	7	8	30	181454	271067	170556	253680	259742	9	6	18	880	274232	183963	371480	170239
andrastin A	4.107	485.26187		211647	32237	255717	3572	16628	26864	66539	42462	116834	65043	240697	11338	7038	82309	99195	34344
andrograpanin	8.416	319.23694	Diterpene lactones	16	13	23	412	967	139298	156846	288	18	6	6	139296	153911	141552	180577	105792
Androstane-3,17-diol	0.941	293.24426	Androgens and derivatives	18001	33871	15090	9211	5495	12433	7533	12827	9790	9805	11467	869	3604	8067	7071	13444
Androstenedione	3.22	287.19934	Androgens and derivatives	948	1821	2396	79148	9258	28664	19272	10336	1719	9110	1382	11561	7455	2685	21106	91910
Anemarsaponin E	5.942	935.52148	Steroidal saponins	5	8	8	2515	2001	12775	3961	12070	27	21	57	1538	1733	25571	2253	1705
Angelic anhydride	0.882	205.08598	Dicarboxylic acids and derivatives	1203584	953311	1433538	208903	175737	133342	221292	166559	800286	1300541	1617489	186150	144615	128884	205269	179676
Anileridine	4.155	353.22626	Phenylpiperidines	35088	82901	53405	10185	5126	4751	8013	6516	45007	28106	14429	12275	4272	4564	4043	4598
Anisodamine	11.284	306.17819	Tropane alkaloids	267	191	214	7220	6854	22018	4000	12439	150		138	6741	3403	14002	3208	4284
Anserine	14.009	241.12849	Hybrid peptides		117	158	42392	93	55	217	70	92	222	71	63	20	15	23	177
anthraquinone	13.552	209.0533	Anthraquinones	22574	22118	24298	30	46	27	44	14	14598	18806	21018	177	64	38	64	68
ANTIMYCIN A (A1 shown)	6.177	552.28735	Acylaminobenzoic acid and derivatives	26	67	50	9327	30060	53556	37297	49883	95	50	33	33805	65729	41334	33254	24065
Antimycin A4	5.07	507.2493	Acylaminobenzoic acid and derivatives	854	304	878	5507	7068	2159	986	11344	1092	82	370	1508	18604	4061	7446	9524
Aphidicolin	3.123	337.23724	Aphidicolane and stemodane diterpenoids	112649	266800	79900	698	217	229	712	869	129559	144549	98923	304	289	251	707	1056
APIGENIN DIMETHYL ETHER	3.211	299.22476	7-O-methylated flavonoids	8799	13698	4316	34152	102383	33335	80575	150722	31214	4680	5376	3269	35532	17104	129408	109240
Apiin	14.236	563.18701	Flavonoid-7-O-glycosides	80	64	9	2275	3580	5164	6573	3260	763	38	78	3035	5889	2064	4654	14799
Aprobarbital	3.215	209.08813	Barbituric acid derivatives	463	888	151	321	53600	32308	486	40596	6945	464	52	408	10231	28670	3588	247
ARABITOL(D)	8.399	151.06094	Sugar alcohols	3	6	3	25966	2348	624	5401	55728	5	3	3	7810	1657	1526	1516	1671
Arachidonyl dopamine	5.341	440.31168	N-acyldopamines	827	551	626	5669	4928	87260	18499	15715	576	220	579	5091	7916	56109	14284	4814
Arctigenin	5.075	371.17346	Dibenzylbutyrolactone lignans	2214	5413	4820	114020	70993	434855	36397	198502	3388	3536	1844	13364	74377	508580	90157	90931
Arjungenin	3.398	505.3526	Triterpenoids	85728	184513	141739	84127	861719	23744	170398	196493	44457	52116	119960	108123	688386	43875	486901	614648

ARTEMISIN	8.762	261.16232	Eudesmanolides, secoeudesmanolides, and derivatives	184	463	347	7725	4435	91864	7296	4683	117	81	13	22476	4587	69028	9216	5101
Artemisinin	13.818	283.16483	Terpene lactones	733	1751	2563	137105	111175	57957	92596	69114	3821	5703	2211	163497	78441	31984	86353	63494
Artesunate	5.842	383.16968	Artemisinins	1836	1549	1515	2732	1949	1786	1155	3050	41163	3421	3019	3333	7679	3126	3447	4682
arthrobactin	0.673	475.35205	Hydroxy fatty acids	10	9	44	1763	11996	1809	214	80282	8	10	5	456	4072	1609	189	862
Asparagine	10.792	131.04813	Asparagine and derivatives	38	43	15	3342	754	3023	50197	432	23	57	23	690	1439	726	13684	66856
Aspartame	7.056	295.13831	Peptides	56	25	34	257520	183439	75800	133833	118716	18	29	39	187095	92946	62418	177289	84279
Aspicilin	5.364	329.23065		5528	10517	6145	74842	329838	17604	297638	218892	6580	5138	6608	37924	87493	46024	205758	319857
Astaxanthin	5.662	597.39087	Xanthophylls	43	178	49	927554	726577	548694	18121	741957	151	103	88	397139	782454	470567	671524	1043211
Atalaphylline	5.346	378.24136	Acridones	11870	37532	10106	5171	11315	3526	9136	10824	14548	2388	1992	4591	5235	4601	4936	9263
Atazanavir sulfate	1.511	353.19971	Valine and derivatives	1103	689	1298	8269	9559	73649	13692	7152	1502	1082	1605	8719	9594	38732	12099	13574
Atractylon	3.225	239.14906	Eremophilane, 8,9-secoeremophilane and furoeremophilane sesquiterpenoids	44434	193241	386681	950	8230	136839	14361	28328	46958	440544	13918	31077	7111	122085	15342	6759
atropine methyl bromide	3.817	305.18454	Tropane alkaloids	1481	711	1078	1137271	2403515	1591724	1241958	1549933	227	1548	1414	183897	1017973	1415064	1582678	1612529
ATROPINE SULFATE	10.643	675.30835	Beta hydroxy acids and derivatives	53	38	62	42463	23114	169503	25538	26591	38	47	109	42882	18262	137095	24007	12911
auranticin A	4.713	439.22757	Depsides and depsidones	2478	3878	1317	11929	22821	20544	19431	9104	2263	2927	3907	18428	18479	6095	23003	30554
Auraptene	3.26	299.14072	Terpene lactones	1945	3050	2105	33506	32934	48271	44828	70514	2248	4580	1777	2326	20698	89925	38266	22512
Aurilide B	3.217	856.72748	Cyclic depsipeptides	12835	7413	15185	6393	5320	334	1002	567	1663	4766	4447		152	2375	1303	4947
austadiol	1.45	235.06616	Azaphilones	2303	2002	3868	24358	8661	6773	10746	10953	2314	3385	2476	44545	13098	6259	15794	11153
AVOCADYNE	3.033	307.22403	Long-chain fatty alcohols	2349	6348	13183	23078	52086	35285	43858	64019	3683	2556	5484	5710	8488	31713	65736	40510
Avocadyne Acetate	3.201	327.25378		10001	22089	6389	12216	12610	36093	71564	40140	50992	836	1394	2531	4404	7022	12760	88471
Azelaic acid	10.821	187.09724		133	280	245	19464	63952	184489	11753	66973	218	336	203	1941372	163961	151073	264332	24062
Baeomycesic acid	3.345	373.08536		895	1831	1595	1056	4777	21085	5502	17539	1090	2049	256	14321	3170	19099	7069	11815
Baicalein	4.112	269.21518	Flavones	24737	15484	21077	1241	1370	601	2641	444	12997	81130	83324	483	744	636	1312	1144
baicalin	12.906	445.12473	Flavonoid-7-O-glucuronides	95	68	78	25670	5940	496	1010	848		71	89	4560	193	379	1796	788
Baohuoside I	7.904	537.16919	Flavonoid-3-O-glycosides	918	413	473	66781	1413	2730	6421	1342	606	477	340	934	1371	3038	2273	1393
Barbatolic acid	13.925	389.05524		65883	20073	20802	28	27	2	33	135	35321	49996	22589	19	9	11	45	21
barceloneic acid A	4.642	319.07486	Diphenylethers	4831	3185	5078	127261	26129	4942	28037	10513	1316	2678	2188	89046	31434	7000	29983	32517
Bavachinin A	2.908	337.20892	6-prenylated flavanones	107231	169919	33165	27	21275	79310	178307	78493	4392	54085	8917	133956	44175	12078	353	69848
Benazepril	3.406	423.18613	Dipeptides	89874	40317	43614	294200	421127	120426	190327	342616	66202	46240	30544	397211	485448	188166	224693	643596
Benzamide	13.995	122.07071	Benzamides	110	313	38	920	4352	3591	5501	8531	121	237	102	4118	4154	2341	5835	2356
Benzoylhypaconine	7.773	574.30768	Aconitane-type diterpenoid alkaloids	14	7	16	19360	29610	4674	503	30936	17	9	25	663	29668	19825	36366	962
Berberrubine	4.35	323.10172	Isoquinolines and derivatives	42275	9204	3602	1225	1209670	2153	20508	744192	16058	4337	2725	5367	15690	7184	26988	18694
Bergamottin	6.616	339.16821	Terpene lactones	167	198	116	232729	321209	188792	204103	365522	214	519	208	265494	228737	211796	268778	131580
Bergenin	11.007	327.09015	Gallic acid and derivatives	194	339	183	462870	351517	15012	173276	113624	80	66	26	30911	187983	26570	82450	120332
Bernardioside A	5.915	667.40649	Triterpene saponins	50	121	85	4161	5886	104818	3227	2085	166	126	149	47892	4654	23139	2478	1536
Bestatin	8.864	309.18149	Hybrid peptides	363	2179	636	61985	23197	222688	39929	28097	464	1346	1972	82540	16666	246159	32053	39409
beta-amyrin-H2O	1.451	409.37576	Triterpenoids	2801	19113	6114	2728	1593	3445	3681	10302	2565	5405	3925	657	1166	1390	1460	3364

Beta-Collatolic acid	3.203	525.22235	Isocoumarins and derivatives	293	521	495	886	2154	3484	4161	3439	605	2087	124435	4707	1801	7472	1300	916
beta-Cyano-alanine	1.556	113.03709	Alpha amino acids	107	146	149	6799	755	5698	151885	1162	207	91	368	4062	3986	480	28808	159866
Beta-Ecdysterone	5.816	481.29697	Hydroxy bile acids, alcohols and derivatives	239	207	283	7573	7288	2235	3972	2978	124	93	146	7556	1752	349	3187	30298
beta-Hydroxymyristic acid	3.715	243.20047	Long-chain fatty acids	30106	39498	18328	296	543	306	1636	3942	9684	12395	8336	327	738	1243	295	1333
Betaine	10.107	118.08557	Alpha amino acids	98517	186772	252993	438176	143605	297870	1146397	161834	114605	115018	119269	81401	108227	30331	399237	988142
Bezafibrate	2.847	360.11139	Phenoxyacetic acid derivatives	154597	111945	65583	1468	234	2866	448	5398	223017	111431	29915	1059	1111	3132	2836	344
Bilirubin	3.254	585.26892	Bilirubins	4248	11824	737	766484	785410	2249290	1269720	2171928	1307	2504	3441	15045	543413	2662385	1083769	553605
Biliverdin	6.49	581.23065	Bilirubins	9722	1335	4944	735	794	729	1029	380	271	2173	19558	419	783	1338	640	1023
bilobol	1.086	319.26102	Resorcinols	7735	11677	5092	22985	21189	28932	30100	70991	6461	1046	3126	10149	21509	27620	38140	4490
BIOTIN	7.052	245.09555		29	12	69	56258	30848	13427	28137	22413	12	18	18	47123	19863	11283	35052	26874
Biselyngbyaside	4.585	627.44775		6509	11681	6739	9452	802	681	4067	1241	1698	13742	8703	524	6105	855	2969	2975
BiseokeaniamideA	1.249	818.44257	Hybrid peptides	15985	4122	3564	2111	317	2029	288	5363	23320	34540	25846	2331	226	1090	290	7137
Bisphenol S	10.97	251.03531	Benzenesulfonyl compounds	12	11	11	3022	65	44	14639	13	18	44	16	541	91	78	516	20073
bonactin	5.246	399.22861		247133	10737	48091	9122	1859	12132	898	4101	64682	23482	12579	4171	2700	4010	6500	2669
brassinolide	3.435	503.3389	Brassinolides and derivatives	26338	29656	3765	15460	85305	111394	54613	152750	26527	4750	18552	49776	30011	103550	44051	33985
Brazilin	4.468	285.11172	1-benzopyrans	1421727	1876561	1246600	2241852	514678	119779	416249	282939	767891	2180786	1320328	1788387	942289	125059	400644	619153
brevicarine	8.451	268.16888	Harmala alkaloids	7	15	11	111829	143405	91237	161102	189000	19	28	11	98831	176479	83558	221672	10740
Buellin	13.825	433.01758		4563	5571	3221	108	20	73	47	53	7425	5423	5670	25	31	16	34	50
Bufalin	3.701	387.24863	Bufanolides and derivatives	1183	3883	1230	31698	54463	196392	110548	73748	2156	2526	4279	9720	51274	189388	144391	85358
Bufotalin	3.292	445.23145	Bufanolides and derivatives	660	688	2618	5192	5678	25856	135628	27100	904	1553	95	8718	9396	190869	45341	69855
Bullatine B	1.133	460.27078	Aconitane-type diterpenoid alkaloids	211858	224776	222622	18556	21620	14549	11280	21318	210082	223433	216595	2385	30014	13775	10958	20613
Butenafine HCl	4.907	318.22461	Naphthalenes	337	1446	400	91412	80284	18984	45098	22656	866	513	754	31522	256425	11496	123420	416570
Butylamine	16.898	74.09582	Monoalkylamines	222774	359213	95570	358	901	678	1066	296	336498	288767	291281	616	942	1353	534	792
Butyryl carnitine (isomer of 920)	9.881	232.15262	Acyl carnitines	43	47	40	72294	7004	287	45221	2640	84	84	116	500	474	70	499	11139
Cafestol	3.216	339.19147	Naphthofurans	5329	8486	10862	196759	155104	1384058	129095	143030	55357	10565	22281	32355	81670	1166404	275774	158899
Caffeic acid	3.674	135.04712	Hydroxycinnamic acids	355	940	815	5500	347547	151518	262557	441582	4357	1951	11453	221182	509078	229172	233898	298176
Caffeoyl lysine	7.489	307.13159	Caffeic acid and derivatives	6	57	14	37084	29209	2228	4007	11478	76	30	9	4569	494	467	270	238
Caftaric acid	13.894	335.03635	Coumaric acids and derivatives	47012	55109	60339	35	278	47	4	41	48555	64327	46293	125	31	72	17	40
calopocarpin	3.396	323.18738	Pterocarpans	4631	4549	5110	24703	47005	11994	11174	3032	4627	5876	1336	13641	47301	18049	91546	24306
Calycanthine	5.402	347.23907	Aminoquinolines and derivatives	1842	1182	1376	9646	25584	224433	27647	302794	14204	3369	1093	78764	21620	58282	21715	17135
calycosin-7-O-beta-D-glucoside	3.067	429.19452	Isoflavonoid O-glycosides	51580	39307	16908	46813	6033	45243	184173	118040	29249	34334	10379	50346	16049	33633	72135	147436
Campesterol	1.508	423.35571	Ergosterols and derivatives	20322	30933	28227	19096	16866	46723	42204	13660	14394	17935	8466	744	14501	32909	19802	25000
Canthaxanthin (Euglenanone)	3.38	587.39996	Xanthophylls	5688	6402	1881	16626	16868	8591	11738	18950	7273	4202	2242	5436	8931	6590	15021	18243
canthin-6-one	9.376	243.06007	Indolonaphthyridine alkaloids	486	74	118212	182	159	168	61	228	111	146486	315	119	49		150	76
Capecitabine	3.122	358.14398	5'-deoxyribonucleosides	7906	30778	27604	6799	2259	737	2783	468	1170	46728	27605	8621	953	217	2405	2792
Capecitabine (Xeloda)	3.092	360.15811	5'-deoxyribonucleosides	17377	65312	64390	111068	7065	10370	31001	10363	2048	63501	23440	23061	2981	1883	20699	155230
CAPERATIC ACID	5.274	401.22742	Tricarboxylic acids and derivatives	78227	4097	15038	5880	4535	3403	3003	3138	13949	7999	4972	7773	3401	5791	2627	2233

Capsanthone	4.993	582.4245	Xanthophylls	1111	229	618	907	1077	202878	2070	3532	365	179	309	708	3952	33112	1382	2356
Carbetamide	3.423	237.12396	Phenylcarbamic acid esters	1544	386	580	11671	4947	269453	63063	22339	589	631	915	98216	9667	92013	51300	8928
Carnitine	13.252	162.11183	Carnitines	90	51	99	2070806	426865	141771	687546	130849	110	40	37	146864	61223	149353	313121	2201338
Carnosine	14.185	225.09814	Hybrid peptides	252	75	52	163196	12782	861	73120	2822	61	65	133	667	188	27	241	441
Carylophyllene Oxide	0.947	205.16103	Epoxides	16879	12840	21739	1976	2264	2051	2371	2461	13409	29417	52153	2768	2607	2031	2823	2250
catharanthine	1.139	359.17084	Ibogan-type alkaloids	136	350	647	60236	55526	5218	19001	98215	63	826	391	802	49341	10664	20045	29155
Celastrol	3.739	449.29498	Triterpenoids	746145	403997	632954	6132	8466	131817	17315	6821	1039392	1058923	381778	10201	5913	1470214	41000	16408
Cephaeline	5.387	467.27408	Emetine alkaloids	2161	2636	1644	158096	89439	56359	518	125620	1835	2162	1927	4473	179863	41050	68317	1410
cephalotaxine	1.681	316.1561	Cephalotaxus alkaloids	23763	6437	3658	1075	274	181	7570	1488	31477	9597	1648	124	119	212	2639	364
Ceridimine	3.925	497.27325		711	782	1388	1574	8724	1349	9612	7054	1120	826	1131	74268	72413	1746	11043	14716
Cevadine	0.901	632.37299	Cerveratrum-type alkaloids	742	651	1054	12574	13913	11649	18559	14459	632	490	388	1034	13289	14571	16216	8152
Chasmanine	10.767	474.25674	Aconitane-type diterpenoid alkaloids	28	54	47	27910	25794	226973	78523	52322	66	63	61	48353	31010	248668	73586	22621
chaulmoogric acid	2.978	281.24796	Long-chain fatty acids	3207176	4131794	1373312	416708	440356	136604	263545	258856	1515934	2051308	2190871	32435	261101	131186	338175	279258
CHEMBL1616521	1.468	413.19064	Morphinans	11081	17728	2873	34251	8971	9463	38861	71150	2414	3889	2015	120866	39948	11917	16009	37295
Chenodeoxycholic acid	4.613	357.27853	Dihydroxy bile acids, alcohols and derivatives	8519	13586	13749	184316	393737	24336604	4291034	2872378	24078	1132	12914	7821	37337	23247350	3649133	1538295
CHENODIOL	4.787	391.28604	Dihydroxy bile acids, alcohols and derivatives	9453109	450035	5780891	18570	92024	279114	106167	223844	5783156	1139706	3487237	13160	54042	731379	308764	45169
Chikusetsusaponin III	6.399	915.53656	Triterpene saponins	116	85	162	459	158	168188	901	2860	142	79	339	3394	1221	174791	330	454
Chikusetsusaponin IV	5.695	925.45422	Triterpene saponins	5	10	20	18128	2991	20299	31	31566	11	21	6	18059	7946	14345	1541	79
Chiodectonic acid	13.999	333.02353		106537	95529	65272	21	36	37	57	14	119564	63666	110364	59	23	47		23
Chloratranol	5.918	185.01253	Hydroxybenzaldehydes	237	175	68	4000	1054	667	33822	1872	657	139	101	1915	1137	525	1483	38942
Chloridazon	4.152	220.02617	Pyridazinones	2500	3449	2761	124021	137602	119209	187318	103150	5142	9178	7285	277039	73830	99907	62674	204766
Cholecalciferol	0.92	385.34448	Vitamin D and derivatives	14077	24420	4381	2109	3355	4296	4493	2802	8670	4378	5198	2235	2856	9462	2036	3334
Cholest-4,6-Dien-3-One	4.178	383.27625	Cholesterols and derivatives	37100	47795	47486	1799	1211	1251	1647	276	33213	31588	37295	3880	691	664	1387	948
Cholesterol	1.473	369.34213	Cholesterols and derivatives	1365	12847	1077	157	159	20042	1218	1885	1416	2660	1974	142	382	19422	412	6024
Cholic acid	3.517	391.28372		71920	31029	146611	604758	11991900	22374974	16960458	11334915	88678	40107	157776	9642594	3761297	21047304	22107186	12143999
Choline	11.607	104.10629	Cholines	163		151	476601	508208	732400	601724	572184	204	459	890	330584	404990	332467	596245	597022
Chrysanthemyl Alcohol	1.008	172.17004	Monocyclic monoterpenoids	30586	21662	30185	156	177	333	595	145	5184	13211	41047	941	72	564	431	102
Cilastatin	5.59	357.15601	N-acyl-alpha amino acids	630	758	655	10918	20264	898	148499	15019	522	655	438	11623	7969	4612	8785	76324
Cimicidanol-3-O-alpha-L-arabinoside	12.021	639.33386	Cycloartanols and derivatives	88	88	57	6734	6006	156156	33283	48596	91	186	64	1852	9262	152095	19378	5635
Cinanserin	8.684	341.16833	Cinnamic acid amides	130	33	319	7038	3335	42469	2983	9168	15	14	6	12206	2338	31245	4309	2847
CINCHONIDINE	1.043	293.17889	Cinchona alkaloids	27395	13615	13231	26084	31687	63131	44713	19059	37169	35577	106399	15313	37164	34237	44081	34310
cinchonine	1.382	317.14685	Cinchona alkaloids	199	953	120	26	134	35552	1966	421	52	43	97	5513	138	11652	3099	63
Cinnamoylcholine	1.237	234.14755	Cinnamic acid esters	58	50	96	9067	23012	3756	21143	37916	216	177	93	10846	16514	17370	42130	3641
CINNAMYL ALCOHOL	9.84	135.09099	Cinnamyl alcohols	21	12	22	23792	24455	408	15693	15271	15	30	23	41983	21642	927	16825	4787
Cinobufagin	3.699	487.23541	Bufanolides and derivatives	24132	12384	14883	16357	11951	147979	22939	8019	30564	20092	25672	26651	14232	109846	26204	20192
cis,cis-Muconic acid	14.593	141.01797	Medium-chain fatty acids	2314	2522	3681	541079	1055527	430719	1392064	528802	5892	2081	3361	1441539	848246	154472	900673	1811412
CITRATE	14.272	191.02132	Tricarboxylic acids and derivatives	206562	307797	132486	30	64	73	34	27	851118	229090	103126	119	75	71	104	78

Citrulline	13.902	176.103	L-alpha-amino acids	207	184	414	25104	18769	14590	10207	22760	2338	941	623	8642	10095	15508	10332	7345
Ciwujianoside B	4.073	1189.59729	Diterpene glycosides		29	26	79	132	3468			88	12	55		53	18186		
Cladribine	1.326	284.0607	Purine 2'-deoxyribonucleosides	33	9	12	9131	28786	871	2887	75598	284	413	8	4485	48390	964	2008	14352
Clobenpropit	4.673	307.07736	Chlorobenzenes	1201	1893	1325	48387	1133	1839	1862	1001	1090	1418	389	14986	3070	1579	1844	2815
Clomid	3.182	406.19443		811	1114	409	58836	48326	14120	59430	100537	1261	1423	620	31961	72386	13728	78705	46494
Clozapine (Clozaril)	8.842	164.06989	Dibenzodiazepines	26	81	49	4706	4236	51212	6430	2953	252	17	43	8041	2537	31290	5796	3321
Cocaine	4.264	304.14948	Benzoic acid esters	1545	1338	516	271063	9483	5440	70472	9868	1211	606	375	10223	83182	16321	66107	97929
Cochlioquinone A	3.694	531.29358	Oxanes	3538	10738	6899	2958	10607	88547	9658	13451	6452	1816	1708	14210	14899	74927	12029	2431
Cohumulone	1.03	347.19156	Acyloins	13825	46248	11257	750	706	895	1318	4790	7597	11616	3627	1212	951	492	689	5453
Colchicine	9.108	398.17505	Tropones	16	15	20	36634	9814	616	6679	1435	47	28	6	9014	4372	288	6474	8713
Conessine	4.206	374.36182	Conanine-type alkaloids	173234	139197	136826	90	168	166		110	114631	101917	124005	77	180	171	95	200
Convallatoxin	4.175	549.29053	Cardenolide glycosides and derivatives	1746	1121	700	3105	5725	9917	47979	6921	2662	697	3260	3408	4607	3237	10117	43611
Coproporphyrin I	7.336	655.28217	Porphyrins	17	62	21	27835	889	1322	4039	563	11	21	11	17335	274	1216	12197	647
Cortisone	3.558	359.19284	21-hydroxysteroids	1399999	1180351	1674844	1327786	1458884	318026	124585	1199572	1345995	823784	399425	1623742	1267395	531093	1024072	974651
Cortodoxone	1.072	345.21103	21-hydroxysteroids	29448	62127	28677	555	3150	93142	1840	6789	207727	19216	17963	1045	2189	1282	1051	1096
coumaroylquinic acid	5.617	337.13596		679	703	984	81479	37016	8455	2601	23600	430	313	460	42815	25604	15766	23998	4993
Creatine	13.152	132.07661	Alpha amino acids and derivatives	49	127	181	976210	62117	6530	1040891	14842		57	44	96015	4562	6611	67305	1883414
Creatinine	6.718	114.06589	Alpha amino acids and derivatives	52214	12502	5748	4779642	820903	10854	6854132	88635	66	17545	21269	5762778	762225	1692	2880670	6208118
Crocetin	4.514	329.18338	Acyclic diterpenoids	46102	47262	224771	62685	85735	165255	60324	120515	146757	298300	86120	73422	61495	185191	56174	60040
Cucurbitacin I	1.496	513.29083	Cucurbitacins	1153	1625	1045	2704	4149	50243	7102	12980	1994	691	1143	10861	6385	46158	6358	3616
Cucurbitacin IIb	4.414	521.34149	Cucurbitacins	2398	5540	2708	108235	99808	11537	28367	25043	4086	2144	3377	148673	92544	5651	43590	54595
CUDA	3.168	719.4809	Medium-chain fatty acids	2124	4720	1336	13386	15087	10285	12284	16996	7320	2199	970	310	9664	6620	12342	12880
CUDA* (internal standard)	4.134	339.26114		57675	51419	131398	1130	1560	525	1117	1818	19935	79122	346864	2477	2584	2144	949	1302
culantraramine	1.997	491.3696	Aromatic monoterpenoids	3016	1288	1509	60564	35176	3702	21337	82806	936	754	4475	2307	12950	5573	185131	19326
culpin	10.268	241.12512	Prenylated hydroquinones	59	191	66	122579	56684	20806	45174	31322	72	56	43	95279	48741	16858	44428	64644
Curdione	1.502	259.14539	Germacrane sesquiterpenoids	5296	1270	6375	2523	1173	70446	9517	1046	1029	462	701	414	2714	11407	11083	6765
curvularin	3.234	291.12961	Macrolides and analogues	4155	5008	5441	101010	75462	149238	63034	130861	3800	3548	1819	238678	161084	129774	120554	65482
Cyanidin	13.817	310.0206	7-hydroxyflavonoids	4156	7302	4310	34	104	45	37	90	6041	6034	3713	37	60	89	172	279
Cyclo(proline-leucine)	1.442	211.14259	Alpha amino acids and derivatives	484	1455	2515	1325	1466	46219	3578	2249	1812	1508	2504	2292	2462	38642	1198	1745
Cycloaspeptide H_130057	2.93	672.33838	Oligopeptides	6587	2751	7056	569	320	885	679	486	7357	9715	13780	1537	399	592	341	262
Cyclopamine	7.681	412.31927	Jerveratrum-type alkaloids	79	50	83	17224	7177	8982	625492	11112	98	56	7	12838	10285	12131	53721	6955
Cyclo-prolylglycine	10.113	155.08057	Amino acids	91	88	114	20263	51403	34797	9682	44768	89	46	154	65844	21479	29176	35689	16441
cyclovirobuxine D	0.921	425.33661	Triterpenoids	45937	63088	55389	178620	92552	34717	61023	79321	35535	45713	26137	17322	143069	36463	62863	96207
Cymarine	3.856	547.29871	Cardenolide glycosides and derivatives	10044	10430	4702	11648	7821	27818	11405	17005	7859	6860	1607	7882	33122	24093	9543	16736
D-(-)-3-PHOSPHOGLYCERIC ACID	14.622	186.99518	Sugar acids and derivatives	14	88	20	19671	90060	10041	192989	19692	37	118	72	272103	56046	1794	75415	271370
D-(+)-Galacturonic acid	8.726	193.03709	Glucuronic acid derivatives	6	59	94	49069	482	494	6958	4600	31	90	3	36440	566	201	5039	15451
D-(+)-Glucosamine hydrochloride	4.689	180.10109	Hexoses	1422	423	1751	16474	28878	13905	5242	284944	2817	1459	1635	4692	3537	4064	3841	17391

D-2-Aminoadipic acid	12.995	162.0755	L-alpha-amino acids		99	51	1806	1775	4832	44647	351	134	87		258	1174	879	7224	29961
dabinol	5.402	425.2374	Rotenones	3075	1532	2653	41033	53552	12049	65708	41227	3754	1386	9326	18779	22393	4902	89973	79477
Daidzein	14.611	292.95804	Isoflavones	12	55	26	3445	27264	1293	61904	3419	19	14	13	96671	13376	204	20783	93239
Daidzin	10.112	439.09665		9	20	5	7023	5684	13636	8456	3899	27	52	28	29768	12496	8809	6125	7348
Darutigenol	3.357	345.26199	Diterpenoids	26171	5897	2677	4897	20876	44686	52168	125697	11210	4256	3335	19453	5387	54354	52593	17053
Darutoside	3.812	507.30713	Diterpene glycosides	1449	2758	2315	22246	5488	7587	15722	19610	1088	1500	2689	4397	58843	6850	21814	41076
Daurisoline	9.711	611.33435	Benzylisoquinolines	279	1952	1541	44510	34753	502801	44144	30252	429	1049	1643	78014	26613	435299	38167	30123
Deacetylgedunin	3.212	439.20712	Limonoids	1323	4930	2533	40859	70037	43055	60046	21602	2770	1574	2229	57136	74907	37219	68423	100323
Debromoaplysiatoxin	3.211	615.23181	Macrolides and analogues	177	339	582	11493	18336	26553	47744	26608	70	126	99	3936	23749	158497	15539	31290
Decahydrogambogic Acid	4.48	637.37811	Pyranoxanthones	5683	35759	12037	1421	2742	378	335	1277	2218	3725	1834	1283	7823	1037	5420	1272
deferrioxamine E	7.796	618.3877	Macrolactams	16	11	76	1351	495	1267	101836	1240	33	14	30	7181	1154	987	3484	1626
dehydroabietic acid	5.531	299.18716	Diterpenoids	454858	1585826	792771	24881	84363	2300	89185	28919	573068	518005	263372	107312	86544	4983	47770	139999
Dehydroeburicoic acid monoacetate	4.461	533.35303	Triterpenoids	21898	39539	39342	2936	3910	3141	3149	5339	4843	16733	38551	2295	3112	3410	5162	6555
Dehydroeffusol	6.256	251.10286	Phenanthrols	28	20	32	6877	1072	517	17956	956	96	81	87	2496	898	663	2526	104917
Dehydroepiandrosterone	3.444	289.20676	Androgens and derivatives	696	850	763	2399	2204	2444	3905	5611	1156	605	1374	24676	3293	1042	9140	2636
Dehydropachymic acid	5.693	549.34198	Triterpenoids	86966	169996	146914	5629271	3232316	6092863	2821018	5105318	50700	219793	33235	5892550	2326380	5622388	5335148	421685
Dehydrorotenone	8.64	415.11316	Rotenones	9	18	11	494	118	156	121	47902		6	8	183	67	201	81	56
dehydroxynocardamine	3.414	583.46112	Macrolactams	13232	10925	12346	596	3491	1907	290	1910	8028	12216	50868	479	396	596	3032	5130
Delsoline	4.611	490.27756	Aconitane-type diterpenoid alkaloids	579	1139	1008	2214	178251	1505	20580	55713	979	483	866	4676	9150	1464	17493	12683
delta-Decalactone	1.226	171.14948	Delta valerolactones	428192	334352	430138	78901	63553	42680	83126	69978	243242	330999	931224	184993	39693	39003	66271	53675
deltamine	8.06	466.29816	Aconitane-type diterpenoid alkaloids	107	22	9	12391	9388	8294	24445	14269	12	13	10	515	27612	6650	7616	42951
Deoxycholic acid	4.599	391.28659	Dihydroxy bile acids, alcohols and derivatives	526831	22816	280828	138917	196470	784386	3387880	112060	2161305	57037	4075710	269585	561855	5322416	269944	158186
DEP_216.1383_16.4	1.102	216.1378	NA	5740	3031	10677	493304	486662	1454654	757447	673723	3396	10820	5808	297401	710389	994225	973921	602063
Dereplicator Identification - rhapsamine	1.014	557.47906		38343	53543	31879	15110	21175	4714	15442	9524	19460	15882	22519	2929	10894	3834	7333	29492
Derrustone	6.442	325.07056	7-O-methylisoflavones	10726	4863	3231	8807	6110	1258	4659	6212	11446	20328	22369	3203	1627	979	2626	5263
desmethylenylnocardamine	7.494	604.35168	Macrolactams	7	14	44	5662	12728	11507	1521	12253	67	40	27	814	20718	34738	37310	1095
d-estradiol	13.826	295.16489	Estrogens and derivatives	1058	2510	1427	280986	242361	121136	185825	212540	3918	9667	3243	351594	182004	81371	218327	115732
destruxin A	7.879	576.33679	Cyclic depsipeptides	6	7	19	2854	4280	3110	21851	3302	3	7	9	1739	2757	1720	2914	3809
D-FRUCTOSE	13.814	179.06033	Monosaccharides	330	585	283	9711	1758	2012	3655	2326	359	595	129	2404	1254	1926	2073	5012
DGMG 18:3	2.915	721.33954	Lipids	180	133	251	1519	6029	2006	5063	26449	242	598	67	313	2321	2623	35182	3405
Diallyl Sulfide	8.853	136.94008	Allyl sulfur compounds	181629	48807	50684		20		4	30	300012	113954	30325	5	4		15	
Dibutyl phthalate	0.882	279.15836	Benzoic acid esters	2757399	2106860	3240906	432926	412798	315795	488386	365238	1776912	2842040	3645159	452520	292171	271375	403571	460238
Dicoumaroyl Spermidine	10.632	438.23972	Coumaric acid and derivatives	19	38	41	22742	32199	21426	28369	29099	28	50	38	39100	36123	21644	29403	30683
Diferuloyl glycerol	5.438	443.13229	Ferulic acid and derivatives	159	84	107	5458	22685	41219	657	28585	455	101	117	730	8665	44037	19821	825
Diferuloyl putrescine	3.47	441.20291	Ferulic acid and derivatives	1355	751	1325	1001321	1036318	319587	1055891	1105923	2177	1507	1157	279957	1822111	4131098	376749	2220651
Diffractaic Acid	11.196	397.06512	Depsides and depsidones	457	607	32	13	113	9	42	83	19381	24397	13914	133	15	17	11	39
DIGITOXIN	5.569	763.37061		166	48	84	1432	1538	33688	255	1874	61	139	30	14214	809	12412	11887	238
DIGOXIN	1.442	779.43384		311	577	42	486	837	6331	2183	7181	223	32	446	407	536	4894	2391	410

dihydroalbocycline	3.091	311.22211	Macrolides and analogues	30322	32625	51005	39716	21532	13659	144126	11257	76919	48303	25909	49380	14400	9987	81987	65562
Dihydroartemisinic acid	5.104	237.18364	Sesquiterpenoids	438	295	629	69337	81442	7814	30954	22799	164	101	285	59544	20427	4196	32153	81440
Dihydrocapsaicin	1.791	308.22278	Methoxyphenols	8871	4342	7703	1013	677	659	1003	633	38370	8342	7222	589	172	830	506	273
Dihydrocelastryl Diacetate	0.846	535.27448	Phenanthrenes and derivatives	163	240	109	5481	11713	41825	27576	50722	1369	225	1629	6207	7337	80276	83740	7324
Dihydrogedunic Acid, Methyl Ester	3.298	475.2179		23788	6605	24385	2193	7779	14907	8473	4941	52151	25847	52593	12740	3962	12440	12041	5014
Dihydropiplartine	5.75	320.24121	Cinnamic acids and derivatives	943	777	584	33717	103013	5159	42083	40926	378	341	908	6359	20867	6163	76028	88989
Dimethyl ketal of oxysporidinone_130004	3.861	536.37964	Terpene glycosides	3603	77295	1620	4838	6322	12222	2186	11623	961	2101	2137	1752	49548	14261	2134	26498
Diosgenin	1.03	415.35391	Triterpenoids	33872	21274	39020	38746	39127	28373	24341	4329	22588	21198	17425	18858	35061	25108	11807	22343
Diphenoxylate	5.268	453.25677	Diphenylacetonitriles	359	560	283	3114	4539	1508	7842	16225	547	324	447	3391	31938	4490	3638	23172
Diphenylcyclopropenone	5.98	207.07417	Benzene and substituted derivatives	1007	821	725	106828	89024	26573	82460	75708	2368	493	630	123679	68857	20954	101472	59108
Disaccharides ((2Methyl-Hex)-Pen)	6.622	385.14423	Sugars	247	516	310	1747701	929593	4320369	163868	955351	1069	161	525	64252	58631	1921939	897971	42222
DL-2-Hydroxyvaleric acid	5.566	117.05646	Hydroxy fatty acids	1042	2203	146	116841	194244	39527	19959	107593	531	347	212	106829	106441	2828	245000	5089
DL-5-HYDROXYLYSINE	6.057	161.08492	Alpha amino acids	36	47	10	2863	2060	1234	60214	2778	52	61	8	8257	2090	1647	9283	18300
DL-Liquiritigenin	4.113	255.11909	Flavanones	343863	472316	46858	189345	147623	51914	162037	107955	40409	26317	162291	150960	220652	82182	222201	219638
DL-Palmitoylcarnitine	4.119	400.3746	Acyl carnitines	29908	37190	23517	193	269	171	275		26393	19097	27623		41	171		149
DL-p-Hydroxyphenyllactic acid	5.989	181.05341	Phenylpropanoic acids	86	3	37	22579	2544	12284	110443	3757	166	86	65	25712	10738	6211	46121	148750
Docosahexanoic acid	4.113	327.25473	Very long-chain fatty acids	1156453	2979364	80944	4404	19650	331	5130	1548	55874	209311	399762	24119	10158	1052	3641	21394
Docosatetraenoic acid	4.572	331.25443	Very long-chain fatty acids	95998	185747	133414	6191	17801	1092	3591	10994	91585	70544	136884	1775	7135	1429	1182	14787
Drospirenone	1.434	367.23083	Spironolactones and derivatives	14753	11110	7778	6655	15205	23769	18226	17348	14966	13348	1413	19094	8379	16731	11233	7501
D-Tetrahydropalmatine	8.793	356.19586	Protoberberine alkaloids and derivatives	31	80	67	162427	188680	81163	194162	210426	31	75	89	157284	196734	88422	255041	132701
DUARTIN (-)	5.733	331.11377	3'-hydroxy,4'-methoxyisoflavonoids	160	362	336	17519	42431	9108	1169	14083	1236	458	433	4684	29155	16844	25539	3680
Duguesuramine	3.497	336.29828		4864	809	248	86537	8593	248	1687	263	437	2390	2321	613	41341	398	1384	41679
eburnamonine	0.87	295.26651	Eburnan-type alkaloids	12631	17423	8305	22054	24430	45261	28878	43764	9287	2746	3302	1747	11575	28189	32836	15994
Ecgonine	6.226	184.09769	Tropane alkaloids	15	26	8	88382	68163	339495	74507	50278	11	4	5	77907	70167	268462	95616	93662
echinulin	4.802	462.32245	Alpha amino acids and derivatives	9222	11513	15757	16609	20443	7728	26884	19290	13026	9789	6786	6094	9730	21050	22843	32767
Ecliptasaponin A	1.482	635.42163	Triterpene saponins	27951	21783	54199	149526	124286	29048	76817	52866	58355	49759	43191	2155	43472	52253	129633	64018
Ectoine	12.405	143.08122		131	64	164	3906	4895	175278	620991	3053	106	71	211	4057	1605	99782	99581	287555
Eicosenoic acid	2.92	309.27664	Long-chain fatty acids	27238	22859	16714	459	76	140	189	64	7620	12383	10248	88	167	262	87	208
ellipticine	4.461	269.11444	Carbazoles	32238	28298	33083	69120	6816	1765	8630	4596	15715	25781	11287	56263	21777	598	15614	17801
Emetine	5.395	481.29047	Emetine alkaloids	512	821	197	64662	36094	14039	1307	28536	228	438	159	5334	53197	11968	18240	41888
Emetine Dihydrochloride	4.985	575.23718	Emetine alkaloids	275	145	266	2844	4301	33480	36421	1868	267	70	24	1029	2658	21476	12662	3720
Emodic acid	14.003	299.00345		1107	874	397	26197	19565	25302	33777	52093	1007	596	1433	40117	44169	66971	68771	48744
Enterolactone	1.674	299.12741	Lignols	24043	3455	5485	841	1300	429	2385	1082	42703	11502	3338	135	305	273	2607	1259
Epanorin	3.787	434.1597	Leucine and derivatives	649	916	1188	7668	3032	2468	9592	37729	1001	630	620	6106	2640	1488	3547	2001
Epiafzelechin Trimethyl Ether	5.533	315.12543	7-O-methylated flavonoids	502	218	298	19427	348876	236444	27679	838995	1138	102	48	13777	13627	16014	46008	8914
epicatechin gallate	13.923	481.03793	Catechin gallates	351	238	5	710	35	157	2251	650	158	146		8367	4782	125	105	8615
Epoxygedunin	3.179	497.28857	Limonoids	1623	1339	625	2166	995	26087	1091	4411	1912	733	46573	3424	2501	9543	3455	1121

Eprosartan	5.467	423.16968	Benzoic acids	509	405	416	14102	53798	18286	2301	38447	754	219	245	140442	28680	30894	49568	3367
Ergosterol	1.483	397.33945	Ergosterols and derivatives	6462	39907	9712	15301	16413	6860	1972	1759	2775	13223	6035	42	21670	3778	1591	6261
Ergosterol Acetate	3.281	465.38266		1020	1680	399	240	1011	10995	895	1672	356	414	107	221	490	17011	527	1083
Ergosterol Peroxide_120246	0.964	429.33438	Ergostane steroids	53395	39236	78849	20213	12056	3257	9235	365	29103	36345	16535	7190	10887	7786	6200	10205
Ergothioneine	13.032	228.0928	Histidine and derivatives	515	185	382	18808	4342	2160	3187	1729	197	74	214	23902	6501	1302	1718	3051
Eriocitrin	5.59	597.17456	Flavonoid-7-O-glycosides	17	7	7	5012	4578	60760	61	24982	88	6	10	11676	2542	139957	14717	49
Ervafoline	9.015	645.34338	Aspidospermatan-type alkaloids	9	26	11	44146	40269	747862	31939	42280	3	10	33	5757	42730	330960	28178	19079
Esculin	4.147	339.06918	Coumarin glycosides	1077	1192	1096	11984	30391	12107	31578	30843	1372	793	1061	93558	9055	48596	54012	37627
ESTRADIOL	6.04	271.15601	Estrogens and derivatives	531	532	902	261745	231397	64976	155809	133120	844	181	112	156056	11499	5376	9653	12883
Estriol	13.424	253.15381	Estrogens and derivatives	995	38	378	260644	104177	24890	51361	12695	26	382	215	320695	94047	15922	40542	38260
Estrone-3-(beta-D-glucuronide)	3.858	445.18616	Steroid glucuronide conjugates	10080	6655	3553	35066	152980	8502	84227	79521	78080	2901	2767	47076	13047	15180	52488	52186
Ethionine	2.045	164.07095	L-alpha-amino acids	1305	128	2199	692	157454	5227	145294	1222818	211	1753	159	2756	173	5020	226478	2431
ETHYL 3-INDOLEACETATE	12.778	202.07246		143	187	68	16191	141275	14061	9691	19166	69	131	56	19239	11042	10549	6065	27944
Ethylbeta-carboline	13.337	197.12685	Harmala alkaloids	86	73	48	446	1432	2362	9943	358	75	84	116	1148	1375	509	3376	32382
Ethylenediaminetetraacetic acid EDTA	13.941	293.09631		3164313	2775653	2207294	119	66	114	115	69	1688780	2051165	2763528	150	143	67	52	74
Ethylparaben	7.535	165.04315	p-Hydroxybenzoic acid alkyl esters	25	46	9	33619	27108	12085	22299	16793	65	36	7	47954	18900	12363	29869	19364
Eudesmin	1.199	387.18057	Furanoid lignans	1039601	996867	1001493	8163	7202	12762	16837	12433	1088832	1006950	941559	11439	16550	12757	11164	10958
Eupafolin	16.876	317.05634	6-O-methylated flavonoids	20367	51274	22457	33	54	26	37	44	41853	33619	31406	12	26	16	47	18
Euparin	13.777	215.06856	Benzofurans	91370	79255	80328	481	135	317	156	103	140209	100871	101411	145	107	408	251	466
euphodendroidin S	5.054	849.32849	Jatrophane and cyclojatrophane diterpenoids	36	19	31	14462	11079	6309	24910	14953	53	73	40	2092	9012	28765	4426	15477
evodiamine	4.456	304.15646	Beta carbolines	99191	113497	108982	250176	11937	5815	50345	11902	32856	138561	39074	225302	74369	4417	40336	78589
Exemestane	13.789	297.18057	Androgens and derivatives	6135	10651	7698	222118	166245	80238	135634	91304	13126	11518	6589	292656	115528	34580	93221	83267
FA 18:1+1O	3.021	297.2453	Oxidized fatty acids	1178565	3386699	290334	104833	244904	133280	181290	54371	1064937	1632750	2187776	127045	128025	65616	72331	173210
FA 18:1+2O	3.618	313.24222	Oxidized fatty acids	81940	116212	67008	6510	4543	1934	21520	4481	15298	26177	100686	3109	8786	6273	1156	9521
FA 18:1+3O	5.184	329.2338	Oxidized fatty acids	8016	3874	3175	214614	2120119	624000	1145981	2750942	3788	3614	11172	121792	213442	713127	2146625	1630786
FA 18:2+2O	4.313	311.22476	Oxidized fatty acids	1637233	651257	1484797	23737	112201	21255	27590	26171	218315	585611	9115227	90371	153662	39146	16338	31483
FA 18:2+3O	4.817	327.22171	Oxidized fatty acids	800848	2907699	936918	28074	151141	21730	111747	38371	908610	1223428	1336753	349788	57311	13196	49035	77362
FA 18:2+4O	5.583	343.21463	Oxidized fatty acids	7997	3103	6915	37766	97739	1723	73037	55066	14559	1853	1108	31272	31021	3883	67787	88150
FA 18:3+1O	3.073	295.22678	Oxidized fatty acids	370751	240016	342874	24787	43069	119827	64001	71810	256517	301695	157909	96819	48216	118055	96956	46157
FA 18:4+1O	3.898	291.19397	Oxidized fatty acids	19596	46951	12746	277	249	125	411	208	12516	27052	26414	329	193	63	171	915
FA 18:5+2O	4.338	305.18872	Oxidized fatty acids	640	442	408	286078	337187	210052	248367	760951	2472	760	826	74027	75254	180698	418426	282788
FA 9:1+1O	3.343	171.10263	Oxidized fatty acids	71304	58718	63214	2162	5984	3231	10411	4132	45262	73897	67825	2530	9444	4364	8449	8241
Fenpropidin	4.392	274.27527	Phenylpropanes	4247216	4320317	4441420	295	490	151	369	150	3375323	5786286	11397674	527	194	582	419	442
ferric coprogen	3.485	844.28748		100	141	54	1768	748	19251	6171	2664	80	530	75	105	16166	3143	952	759
feruloyltyramine	4.139	352.12756	Methoxyphenols	465	244	693	2684	33548	439	6922	36346	913	1153	603	8714	4261	1457	9402	2313
Fesoterodine fumarate (Toviaz)	3.31	412.28619	Diphenylmethanes	15407	22020	15676	12240	7145	66284	119025	3886	18080	13198	7067	8404	9870	3379	30100	279375
Fipronil sulfone	14.113	450.92307	Phenylpyrazoles	13	41	26	342397	322746	384758	86390	399560		14	49	180251	303607	517583	82690	277722

Flucofuron	13.759	414.97498	N-phenylureas	5677	8611	4991	36	64	166	132	103	6908	5476	4633	44	76	114	218	126
Fluorometholone	7.255	377.22046	Gluco/mineralocorticoids, progestogins and derivatives	127	71	127	3392	2792	2307	243438	660	155	104	50	27595	3335	2320	2886	15442
Fluphenazine	12.175	436.18689	Phenothiazines		105		60	153	182	12628	76		64	108	58	107	209	10883	416
forskolin	3.322	411.24887	Triterpenoids	10120	12393	61672	4944	12104	6010	3285	11775	14253	23973	17012	4684	7461	16923	22730	4925
FT-PFCA	11.318	127.00365	PFSA	45535	37089	741	28	110	45	11	14	15407	16886	8443	78	45	19	52	41
FT-PFSA	1.672	226.97987	PFSA	77471	85386	89540	28	83		30	103	31513	21083	63912	24	39	12	25	44
FT-sulfone	14.597	532.98914	PFSA	25	42	17	2756	11210	1721	16686	2860		16	73	18134	8608	232	10455	20887
FT-sulfoxide	11.63	552.03802	PFSA	144138	248441	687751	26	14	6	19	4	131940	162400	292770	49	7	42	15	5
FT-thioether	11.309	387.00299	PFSA	652472	157	43	86	128	77	36	34	528643	672070	957756	100	25	17	21	43
FUSIDIC ACID	5.865	515.32428	Steroid esters	1764	1372	1403	23925	24787	3129	10621	21045	2421	1558	1613	12986	26417	5209	28911	3420
Fuziline	7.594	454.29056	Aconitane-type diterpenoid alkaloids	21	1490	503	16569	5509	1082	4789	6522	4781	50	40	1694	48975	1246	3265	8108
Galanthamine hydrobromide	3.57	390.0676	Galanthamine-type amaryllidaceae alkaloids	69	43	252	1056	21990	44204	34171	21418	167	29	375	21338	6089	29774	33654	20053
GAMBOGIC ACID	4.678	627.28693		540	1359	579	23386	13410	59093	27776	15124	811	715	670	12945	11308	52976	29312	26287
gamma-Glutamylglutamine	6.584	274.09641	Dipeptides	3	13	15	8698	7947	355	3395	5788	21	28	36	8454	20569	793	1059	4929
gamma-mangostin	7.153	395.16306		23	69	7	34413	53182	16955	34618	46143	91	21	10	30438	62158	25145	50188	29376
Gangaleoidin	14.595	410.98392		37	28		14526	53002	7151	81932	13571	55	51	29	85607	37920	891	41338	110289
Ganoderenic acid E	3.626	551.25244	Triterpenoids	240	370	922	3809	6941	29001	17824	14137	707	73	164	2589	8422	26147	5881	10324
Ganoderic Acid A	5.99	517.30066	Triterpenoids	140	295	259	12341	7736	63545	25844	4194	519	157	420	9937	10581	88064	19629	22274
Ganoderic Acid B	2.989	519.32861	Triterpenoids	10553	1809	7324	28926	5360	79932	32309	62973	13982	3224	4936	135905	6940	72763	21534	20448
Ganoderic acid D	2.986	537.271	Triterpenoids	240	4041	588	3188	5185	66596	18004	7974	1042	446	752	4318	8544	74129	8935	9969
Ganoderic Acid F	3.297	593.27185	Triterpenoids	1967	7365	1313	7820	9615	9118	2334	2836	38079	922	635	2041	5123	1687	3682	6101
Ganoderic acid I	5.425	555.31213	Triterpenoids	146	96	306	29307	36071	48954	19704	9495	266	99	197	9227	9682	21556	27409	8622
Ganodermanondiol	5.1	457.33948	Triterpenoids	1372	1101	1171	21828	321373	28595	159217	534828	2914	1020	1184	2776	1751826	195076	308752	179571
Ganolactone B	8.21	459.26285	Triterpenoids	17	6	30	28892	39284	35681	6516	53529	20	11	6	11757	37818	33431	68959	2166
Garcinolic Acid	5.521	645.29944		214	95	201	9409	3609	4314	2134	7506	279	300	109	4354	22905	3910	7039	736
Gardenin B	13.92	381.07825	8-O-methylated flavonoids		195		27488	5859	6221	34952	17130	104	27		117386	76532	4117	7008	115615
Gardneramine	3.618	413.20789	3-alkylindoles	3385	1438	3062	45441	58035	6444	17334	13336	1914	845	4025	19468	35915	4726	20055	37488
Gaultherin	3.96	153.06543	Phenolic glycosides	19852	23235	25534	19586	574	2348	1007	1351	2689	120612	16507	9655	4129	151	2631	1775
Gedunol	5.417	483.27621	Limonoids	15643	17192	9412	1476026	709486	830021	1326	908260	15233	11864	22037	378478	1367448	610542	679561	5556
gelsemine	4.023	345.15247	Gelsemium alkaloids	748	435	562	14431	43815	21457	39252	45365	495	640	461	135683	17673	22223	61671	35548
Gelsenicine	1.547	327.17288	Indole and derivatives	158	89	558	324	4965	22254	2893	1439	109	771	378	246	4087	44957	1532	1659
Genistein	13.339	562.93079	Isoflavones	35102	65011	54322	13	10	20	13		11950	29351	33176	6	10	16	8	19
Gentiobiose	13.904	360.14828	O-glycosyl compounds		129	3	109404	15853	18278	32287	42442	137	511	111	140690	107811	32243	16928	64001
Germbudine	6.708	710.42126	Alkaloids	46	45	33	11726	9833	4398	7615	18581	41	140	82	192114	19286	6515	15156	14609
Germinaline	5.546	796.4386	Alkaloids	41	68	30	365	434	36882	73	1258	30	16	6	503	650	32170	1038	240
gibberellic acid	8.734	347.15274	C19-gibberellin 6-carboxylic acids	46	564	756	10095	7890	93635	9933	9012	13	25	6	35455	6224	62259	10331	5228
ginkgolide B	4.753	442.15869		6742	3049	17371	1282	486	153	293	275	792	10247	21640	661	465	124	95	394

ginnalin A	3.266	467.21136	Galloyl esters	6169	7807	11154	13407	6816	161345	46411	17247	7300	3444	6503	13946	12427	145766	55510	20219
Ginsenoside F1	5.818	639.44849	Triterpene saponins	1942	3079	1857	766	4735	348	2150	1435	9163	1346	3001	396	669	456	1990	1573
Ginsenoside Rg2	2.835	807.50555	Triterpenoids	14166	29516	4950	2083	403	1421	634	1288	1553	1561	488	89	353	109	5739	267
Ginsenoside Rg3(R-FORM)	8.077	783.49982	Triterpene saponins	174	403	754	4171	6911	28816	7047	27992	698	783	532	8333	76	23008	10176	5667
Ginsenoside Rk2	4.156	605.43011	Triterpenoids	15945	6349	29031	5388	1637	3624	1887	1426	2330	43036	69802	2415	2875	879	2779	2293
Gitogenin	2.988	455.31097	Triterpenoids	191599	179228	138674	47578	25308	19889	8866	27875	87298	173636	63523	6428	34799	70937	31433	40122
Gitoxigenin Diacetate	2.847	473.28519		7709	8537	9859	63	196	121	171	113	11104	11713	11537	179	59	116	350	52
Glabrol	4.743	393.20508	8-prenylated flavanones	23466	15648	24349	8812	14007	14531	18625	12187	11007	18864	9816	1157	12296	7008	18371	9668
Glucuronate	13.576	193.0358	Glucuronic acid derivatives	4115	30238	18454	119	54	56	27	74	2891	38897	9271	167	72	110	23	37
glutamate conjugated chenodeoxycholic acid	7.722	544.33789		7	41	22	80566	63093	45922	350781	28562	6	25	16	2460	34341	80639	16758	33171
Glutamic acid	6.53	146.06268	Glutamic acid and derivatives	13	46	21	750	1407	4120	3072	87323	25203	102	24	112	1735	13806	2614	3291
Glutamine	13.718	145.06454	Alpha amino acids	1056	1386	3774	16755	1042	1447	6142	1642	2617	1832	6589	900	1184	1539	3444	856
Glutamyltyrosine	5.568	309.10629	Amino acids	311	288	250	61870	19163	1649	1546	29698	206	230	81	52017	8172	1800	2761	1885
Glutaric acid	13.334	131.03601	Dicarboxylic acids and derivatives	83	40	140	11426	10595	56313	24077	15099	44	108	276	8090	3385	19962	9472	28117
GLYCERALDEHYDE	6.702	89.02557	Monosaccharides	45	21	4	707720	251606	95707	382054	355446	23	51	33	126859	83097	23046	254842	198247
Glycerol	3.678	91.05598	Sugar alcohols	56	993	1370	4022	110023	61656	83269	144218	1479	252	1231	79495	156372	81219	77643	111151
Glycine-Betaine	13.835	118.08611	Alpha amino acids	30106	25143	44489	24624	32979	44988	79001	27823	22050	50565	38757	53976	63033	22939	32781	71056
Glycitein	2.959	285.07565	Isoflavones	429	1115	335	27104	1747	22159	431777	3696	402	439	325	1380	3658	831	55900	141112
GLYCOCHENODEOXYCHOLIC ACID	7.26	450.32007	Glycinated bile acids and derivatives	51	108	52	24005	45356	578321	54469	53221	56	36	49	3412	90251	506081	47955	63144
GLYCOCHOLATE	8.524	464.30243	Glycinated bile acids and derivatives	512	889	501	48764	30610	361772	49062	15835	585	1298	559	34005	67231	253522	58986	20467
glycocholic acid	5.358	929.5882		34	9	45	190	341	13432	130	485	55	28	20	59	190	17951	370	128
glycodeoxycholic acid	3.191	919.63495		168	236	89	39	230	99	12	430		138	49912		58	15	27	97
glycohyocholic acid	6.141	448.30399		532	636	367	22395	17872	71367	41223	19473	576	366	224	36183	27088	52787	27397	43664
glycohyodeoxycholic acid	6.938	448.30786	Glycinated bile acids and derivatives	105	55	158	57023	35600	477733	81414	57860	211	29	140	71782	109911	153976	75601	88789
glycolithocholic acid	5.48	456.31458		3503	5341	4104	85548	77872	30212	49670	90003	5337	2886	1040	101146	68890	26048	75638	33687
glycoursodeoxycholic acid	7.292	448.30997		111	96	123	79935	119513	3103552	176099	71068	79	17	75	137929	445844	2431601	153847	218387
Glycyrrhizic acid	5.013	823.38513	Triterpene saponins	83	161	105	3851	6838	1899	9725	10847	2452	75	39	1118	3138	11705	1620	2987
Gomisin H	1.048	419.23087	Hydrolyzable tannins	6026	834	3450	133718	205265	213791	486806	593512	1306	5374	2551	117618	218284	185246	336201	174510
Goniomedine A	5.942	649.34967		27	33	105	4221	4180	4112	21724	3150	24	74	306	2540	5106	27773	6932	10103
GOSERELIN ACETATE	5.545	635.3197		12	29	78	1350	815	214716	575	3917	34	14	23	67606	6555	241800	159760	454
Gossypetin	13.763	357.00745	Flavonols	94468	163376	77965	25	158	171	127	83	123079	90995	73760	66	101	229	274	169
Gracillin	10.9	885.50427	Steroidal saponins	6	2	7	14915	3244	308629	8166	8134	2	17	7	14086	3401	191985	2615	2794
GUAIAZULENE	13.346	216.15785	Guaianes	70	90	95	26558	51726	8140	41002	8538	177	88	39	32137	9705	5101	51107	23280
Guanine	12.172	151.14378	Purines and purine derivatives	77	74	89	108031	2411	18	12184	28	50	92	115	64319	73	5	289	3272
Guggulsterone E&Z	5.153	313.23798	Androgens and derivatives	2715	3744	1148	19730	184963	45031	99071	182064	4073	1803	1112	2410	6135	31046	131740	69617
HAEMATOPORPHYRIN	5.657	597.35938	Porphyrins	263	231	111	8003	70542	297324	1696	346671	38	188	138	91115	130540	1152603	135482	1960
Halometasone	5.522	443.14743	21-hydroxysteroids	179	251	124	12648	19160	48055	1606	34188	395	133	160	18097	8289	2368	18162	918
Halovir A	1.12	866.62341		7768	9392	8076	115	426	362	287	243	9596	7375	13430	57	458	79	87	517

Harmalol	6.604	199.1008	Alkaloids	127	264	113	33450	47034	29434	60918	48019	1978	132	138	32646	31554	55629	32987	138228
harpagoside	11.45	492.99512	Iridoid O-glycosides	34848	56933	66054	24	20	11	29	9	14263	9857	10537	36	18	22	8	20
harringtonine	3.062	532.26196	Cephalotaxus alkaloids	239	313	923	4786	9544	45593	7390	9211	1126	313	349	2879	6608	34264	7245	5204
havanensin	5.827	453.2601	Limonoids	92	428	366	3797	7016	1804	2743	5189	285	128	110	5247	4931	918	6255	146648
Hecogenin	7.727	431.30643	Triterpenoids	4286	10900	3253	781	508	1367	581	555	1996	1179	60	472	744	484	793	1587
heliocurassavicine n-oxyde	13.857	324.1796	Pyrrolizidines	478	1034	678	366651	242790	93363	199301	196480	1063	2871	474	398877	198931	63909	283411	104294
Hematoxylin	5.65	301.11444	1-benzopyrans	420	1231	1035	51700	29797	1500	32677	8758	805	949	823	21031	10745	2067	29135	22254
heminordihydroguaiaretic acid	5.091	315.22293	Dibenzylbutane lignans	39984	25744	20833	6661	3411	3640	1148	505	2206	18121	5515	4419	8114	881	2961	3562
Heptadecafluorooctan-1-ol	14.608	434.94955	PFSA	27	25	87	5977	11882	3751	13202	4580	39	16		6918	8405	559	9179	6966
Heptadecanoic acid	2.904	269.25299	Long-chain fatty acids	155285	57257	93610	1788	814	154	309	154	84499	58853	26739	430	78	417	405	464
Hernandezine	11.304	675.29797	Tannins	23	22	17	11640	4977	198425	11322	37199	137	134	91	1270	6085	160930	5729	7367
Hexachlorophene	8.838	402.83453	Diphenylmethanes	6877	658	312	7	3	41	16	35	9854	8949	4448	6	5	10	42	7
hexadecanedioic acid	3.095	309.207	Long-chain fatty acids	168829	147122	273275	39021	28453	16696	59141	30343	131137	202420	86391	75298	55269	26998	18583	28285
Hexosyl LPE 16:0	7.434	616.35114	Lipids	50	64	26	11977	18652	25261	1637	22727	45	163	22	1100	16015	11919	43822	2834
Hexosyl LPE 18:2	6.506	638.32581	Lipids	154	165	337	3401	1901	6635	3114	4164	327	166	348	1715	47725	3394	4627	1072
hippeastrine	1.528	316.12344	Homolycorine-type amaryllidaceae alkaloids	92	264	108	17791	109181	67684	170845	23695	144	106	786	1289	196203	52520	9396	172246
HIPPURATE	5.878	178.05138	Hippuric acids	175	42	199	1260670	7805	171	293	3372	252	131	28	3060029	6458	857	1083	951
Hippuric acid	5.855	180.06645	Hippuric acids	332	202	79	48601	1186	809	1426	421	254	131	175	114801	1730	386	1713	2443
Hirsuteine	7.199	365.19958	Corynanthean-type alkaloids	102	86	69	46448	31447	9580	27312	12555	267	24	62	7686	11098	8244	37513	21179
Hirsutine	8.287	369.21964	Corynanthean-type alkaloids	12	22	8	12804	21584	19134	813	28453	5	17	27	389	28141	19775	35041	1090
hispanolone	8.494	319.2377	Colensane and clerodane diterpenoids	19	7	17	116445	114322	135676	156328	191490	30	12	9	2140	151097	146414	173299	101825
Homoarginine	13.601	187.10741	Alpha amino acids	15751	16970	1771	378	859	404	458	433	4573	2705	2032	751	618	220	439	449
homoorientin	3.486	447.20703	Flavonoid C-glycosides	6171	6993	2622	7081	7376	14102	4189	16887	1861	1795	2116	21387	4330	13369	17202	55915
Huperzine A	3.694	265.11539	Quinolones and derivatives	626	1973	352	832	596	697	1295	5360	38320	216	590	2053	4122	37615	1827	2136
Hydrocinnamic acid	3.284	149.06201	Phenylpropanoic acids	243114	812102	350828	585966	1638850	770859	2250754	1763656	956048	1459554	47943	3190946	4639140	1117150	1632624	2684170
Hydrocortisone	7.439	363.2059	21-hydroxysteroids	20	17	37	1194	917	481	270291	629	39	10	29	31441	1258	1149	1254	13381
HYDROCORTISONE HEMISUCCINATE	5.24	463.23727	Gluco/mineralocorticoids, progestogins and derivatives	237	160	200	16794	16673	1734	10829	19895	178	142	218	3106	48668	1803	8817	19875
HYDROQUINIDINE	1.472	325.18472		46395	35128	33897	77445	42738	43511	50839	36736	101938	102431	166834	31540	49651	20633	47080	52880
Hydroxygardnutine	3.13	383.14969	Alkaloids	6763	14058	4177	19925	32673	19176	23780	26872	4921	4517	1957	50284	39163	34053	28665	13303
Hydroxysuberic acid	13.399	189.07616	Organic acids	41	59	98	14419	17993	9198	15186	7986	113	80	125	16028	7239	6777	8445	13475
hyocholic acid	8.263	431.26688		19	18	38	742515	767046	746751	11223	347456	5	11	6	4279	469360	873503	1376497	6123
hyodeoxycholic acid	4.285	783.57587		1020	505	626	149	2491	4154284	21350	18328	22306	450	1606	842	194	4594642	39662	7549
HYOSCYAMINE	4.905	290.26764	Tropane alkaloids	22727	110184	36205	319	497	105	501	671	45142	59495	197711	16297	336	419	327	126
Hypaconine	13.257	470.26239	Aconitane-type diterpenoid alkaloids	31	69	52	64775	64962	996055	5508	43330	141	44		261194	3843	176192	113739	2042
Hypaconitine	3.505	616.32074	Aconitane-type diterpenoid alkaloids	547	631	1905	10331	8988	3415	6397	14734	1355	537	260	1020	20735	4379	8868	12522
Hypoxanthine	6.41	137.04439	Hypoxanthines	4047	5214	650	703202	296208	96189	1551999	245615	3864	2616	776	258937	276242	153347	329318	4488264
Ibogaine	2.977	311.22159	Ibogan-type alkaloids	1705	2286	3971	29023	34262	22378	141718	15324	2456	3019	1517	19958	19595	18951	78636	66009

Ibuprofen	7.108	205.10747	Phenylpropanoic acids	2	20	39	24821	26975	8279	17684	9629	10	15	45	17049	15448	11780	24772	24166
Ilicicolin A	1.113	389.24655	Sesquiterpenoids	20780	18207	17413	1921	2188	2938	1667	3225	31791	18792	8555	2107	2747	2930	2000	1302
ilimaquinone	11.475	433.00153	Prenylquinones	5707032	6434512	1190898	22	12	15		18	9665321	3983026	6522638	5	16	12	25	90
Imbricaric acid	3.155	415.18698		6208	14460	26279	75978	96685	35822	63791	84713	25147	6342	4674	126789	78801	30287	144918	57402
Imidazole	3.652	69.04424	Imidazoles	192134	137809	82085	2651	10326	5802	24979	8932	123716	139894	62042	32713	9926	8192	15184	40536
Incensole	4.823	329.23267	Sesquiterpenoids	370189	815389	249549	17034	70083	12505	45445	34763	362325	348013	454335	245865	47461	10954	40863	32071
Indinavir	3.651	612.34674	Alpha amino acid amides	1149	726	798	1214	2640	26082	4988	4990	949	1333	962	4485	1611	30135	5117	5661
Indole-3-acetyl-L-leucine	3.38	289.15411	Leucine and derivatives	2677	1194	1490	1680	1696	173352	5401	3764	2419	1174	966	2137	1200	28995	10045	2198
Indole-3-carbinol	1.383	148.07445	3-alkylindoles	8488	2555	3252	301	19637	17380	19852	134774	264589	1936	1270	3714	278	30979	15283	3501
Indole-3-carboxylic acid	13.681	160.02608	Indolecarboxylic acids and derivatives	10435	13137	11755	1519	667	628	1553	2182	12142	10679	10562	990	774	1076	1700	1678
Ingenol	0.942	349.2178	Tigliane and ingenane diterpenoids	16258	23255	11610	11832	7928	5691	16965	10267	17476	16689	5533	23785	6994	5935	12774	5342
Ingenol-3,4-5,20-diacetonide	7.802	429.25293	Ketals	8	16	20	10638	13576	11986	2580	256763	59	9	6	2111	224012	215792	341443	2823
Ingenol-5,20-acetonide	5.936	411.21484	Ketals	420	1255	1443	87275	59492	10946	43631	33808	1890	873	712	102393	54432	8734	48796	45713
Ingenol-5,20-acetonide-3-O-angelate	5.59	471.26962	Fatty acid esters	482	400	218	3974	4066	1882	95536	4300	398	213	172	38843	3839	1396	6018	408187
Inosine	7.901	267.07431	Purine nucleosides	14	34	79	614809	10417	30050	109921	13429	16	56	6	4806	29384	92079	12172	6506
Inosine-5'-diphosphate	13.923	426.99942	Purine ribonucleoside diphosphates	92696	23902	29218	19	27	558	262	37	4877	34092	18779	258	61	48	174	13
iodinated analogue of makaluvone	11.106	329.00742	Pyrrolo[4,3,2-de]quinolines	527	491	1013	173	378	53	199	310	107536	72742	70533	340	123	747	101	305
IQ	6.761	197.08093	Quinolines and derivatives	40	12	27	477	318	3236	11841	429	31	4	18	452	426	538	1431	21649
Irbesartan	3.732	427.24899	Biphenyls and derivatives	104035	44453	130935	472	3047	5499	867	1359	109931	92144	135325	3726	3025	1959	1355	982
IRGANOX 1035	4.489	665.38055	Phenylpropanes	1181	3544	12803	5526	6641	5023	3702	4251	48826	18838	3930	6706	7475	4480	10022	987
Irgarol	8.428	254.1543	Methylthio-s-triazines	17	14	34	3975	268296	170556	254935	4139	12	14	29	171317	274232	183963	4989	167403
Irgarol-descyclopropyl	3.286	214.10754	Methylthio-s-triazines	2439	4359	1384	29193	38522	261104	35417	26538	4323	1731	5944	58230	14300	174585	64552	40683
Irigenin Trimethyl Ether	3.638	401.242	7-O-methylisoflavones	2761	19302	23696	333	1091	1907	2880	717	5766	5383	2941	1672	2157	2415	569	1227
Irigenin, 7-Benzyl Ether	4.901	449.23404	3'-hydroxy,4'-methoxyisoflavonoids	62704	3489	34278	2267	2138	1918	1529	2762	33746	9877	32863	1104	1593	3860	1432	724
Irinotecan	3.26	587.29517	Camptothecins	855	1693	662	24636	56961	416560	139131	186587	438	396	837	2886	35418	180687	132921	66393
Isobutyric acid	3.489	87.04657	Carboxylic acids	6883	69778	31940	7022	141575	82598	176547	381466	57155	63430	19157	196125	263883	156156	205772	126093
Isocurcumenol	1.004	235.15343	Guaianes	10254	8169	14191	847	1399	1278	3490	1063	3747	10815	14102	3194	1448	1067	1981	2536
Isoferulic Acid	8.703	195.04929	Hydroxycinnamic acids	14	51	17	12640	80	76	2149	1349	21	8	12	13064	208	76	1557	4268
Isoginsenoside Rh3	4.007	627.4054	Triterpenoids	1495	1377	743	9018	7325	28608	6304	19929	1455	2501	2521	11129	2842	27788	7721	3796
isokaempferide	5.19	299.0899	3-O-methylated flavonoids	12670	24257	9940	41487	2774	1625	2753	3009	10247	26343	12997	48608	3392	1914	2899	3484
Isoleucine	13.285	132.09712	Isoleucine and derivatives	104	66	112	22964	1443	369	2243	854	318	346	122	4565	3427	83	749	4297
Isoleucylvaline	7.407	231.16879	Dipeptides	17	81	26	78717	21688	26615	60742	29465	76	45	14	63172	29286	39753	50969	66393
Isoliquiritin apioside	3.599	551.15973	Flavonoid O-glycosides	51	33	127	1081	2062	23235	2881	7487	121	72	71	6327	4045	20197	7555	4512
Isomuronic acid	7.157	365.23746		19	48	9	3283	6390	5949	11615	23258	74	12	16	60488	10854	2245	7670	1439
ISOPALMITIC ACID	6.399	274.27167	Long-chain fatty acids	338	490	705	1522507	2769407	165059	78624	2703966	667	269	1190	1673	2014784	62939	140070	39963
Isorenieratene	3.301	528.36511	Diterpenoids	54167	42884	86738	128423	93421	136683	65875	77121	59633	114614	120648	6093	77707	152614	79293	163257
isorhapontin	11.051	419.10068	Stilbene glycosides	39	24	74	12783	3184	2210	3566	1586	15	32	19	11166	3888	1418	673	4723
Isorhynchophylline	3.845	385.21045	Indolizidines	39708	8872	4824	3034	2962	2044	3563	2741	5472	11140	3156	7465	2561	3795	6621	2130

ISOROTENONE	5.82	393.20023	Rotenones	1759	1459	1272	46750	29560	8469	96076	20160	3824	3544	2121	34899	24255	11149	23818	46187
isosakuranetin	13.472	286.99585	4'-O-methylated flavonoids	33172	27276	23540	66	31	18	44	20	37965	44156	34666	54	15	281	59	58
isosteviol	3.151	319.22348	Diterpenoids	6754	7626	9466	147458	65710	41672	48548	6265	2932	6002	8539	6737	74326	33992	26603	188350
Isotalatizidine	4.725	408.27237	Aconitane-type diterpenoid alkaloids	18551	2911	147705	14786	14780	28704	7108	5459	1693	95377	37709	17902	10923	5573	13817	21470
Isoxsuprine	1.458	302.18951	Phenylpropanes	2840	571	724	178	500	1835	42059	581	128	2586	574	2674	89	2080	10664	26874
Ivermectin	10.814	897.50421	Milbemycins	3	11	3	1632	917	107960	560	4166	4	3	9	398	1564	117421	135	435
Jatrointelone C from S.guyanensis	1.505	316.2085		42110	37595	57205	456	320	262	1224	642	45321	56885	63174	1288	456	534	343	1276
Jervine	3.682	426.29587	Jerveratrum-type alkaloids	1357	858	608	7622	21745	114695	126971	19830	1640	990	823	1715	20723	39754	44826	12250
Juarezic Acid	0.855	172.99242	Styrenes	86670	58282	1409857	3544	58	189	1117	89	729	499294	77332	2645	152	47	320	998
Kajiichigoside F1	1.543	651.42181	Triterpene saponins	11665	12811	37077	177961	118783	26231	100984	76794	26318	24469	23532	1811	60204	75133	140355	59230
Kalopanaxsaponin H	10.055	913.50439	Triterpenoids	8	9	8	11	54	92077	77	176	10	2	21	83	30	64381	192	50
Karacoline	8.235	378.24005	Aconitane-type diterpenoid alkaloids	23	4	7	114997	132537	118495	8378	159423	62	7	6	3717	122064	111488	156266	6585
Kaurenic acid	6.343	301.20435	Kaurane diterpenoids	27519	59438	422080	17115	101306	3381	30587	20978	654587	80469	58830	20711	14663	6847	59689	38635
Ketoleucine	3.645	129.05602	Short-chain keto acids and derivatives	18110	520608	17224	3281397	1100798	1145929	1099112	166411	31941	214102	54258	1423990	1626488	400804	1268963	133319
Ketovaline	3.776	115.04108	Short-chain keto acids and derivatives	1943	47199	1214	443839	209799	208044	112100	16736	7197	31119	8900	108401	94523	58187	263313	59701
Kinetin	4.065	214.07639	6-alkylaminopurines	997	3514	279	33293	91115	28730	77330	109488	2548	822	607	12951	14823	14565	33222	39139
Kirenol	4.688	339.26395	Diterpenoids	2790	1231	1097	15075	16052	264040	57851	49897	3046	886	1384	11887	12855	300838	60159	27392
kojic acid	13.582	180.97627	Pyranones and derivatives	69060	21623	77882	585	827	825	1374	666	79355	114285	62089	2943	2435	1425	1630	2012
Koparin	2.883	299.1312	3'-hydroxy,4'-methoxyisoflavonoids	13082	5031	9101	14764	171	81	160	387	7728	9135	8033	4529	419	150	6381	60
korseveriline	1.036	432.35779	Cerveratrum-type alkaloids	22171	18899	25488	22752	14673	6058	7610	3599	13533	22490	13289	6441	11866	5851	4146	9837
Koumine	6.85	307.16599	3-alkylindoles	206	209	204	33632	44315	166379	27135	43228	287	138	348	41471	29101	138074	28654	19169
Kukoamine B	8.314	553.31378	Catechols	37	31	24	88618	94950	71667	150002	126818	55	11	22	2943	105227	66732	111166	76716
Kurahyne	6.251	861.58478	Hybrid peptides	9	12	10	914	133	17049	957	193	7	3	6	98	240	13976	392	271
Kuwanon C	1.206	421.14899		7213	7407	8196	450	453	155	156	246	10618	31213	49855	1314	307	480	452	570
Kynurenic acid	5.826	188.03827	Quinoline carboxylic acids	147	261	287	4750	5686	1512	61431	1330	602	99	204	9243	1366	8501	4405	232176
L-5-Oxoproline	13.771	128.03809	Alpha amino acids and derivatives	8259	8811	2369	318	4679	2529	3007	258	9438	2508	4260	257	1549	2620	506	791
Lactamide	10.761	90.05355	Secondary alcohols	2	1	3	4627	231	1885	54708	24	3	8	7	806	649	102	8705	64276
Lactic acid	7.187	89.02464	Alpha hydroxy acids and derivatives	2	18	12	145672	36822	6516	73829	48326	20	2	5	34274	10025	3264	38828	6595
Lactose	4.156	342.29669	O-glycosyl compounds	59537	32085	44626	12073	17213	643	1266	4463	19928	20504	31809	508	6477	712	1555	9648
Lagochilin	0.879	374.29199		944	75706	880	716	3739	190	3845	2165	14141	499	844	5545	885	267	2745	4197
lamalbid	5.854	421.1366		3973	4075	2352	4984	4381	2587	8251	3651	84708	8533	9272	6098	2615	2193	2378	6230
lappaconitine	6.183	607.31097	Lappaconitine-type diterpenoid alkaloids	205	989	177	14495	21414	148416	39726	16913	291	262	575	13366	9571	14057	5819	12257
L-Arachidonoylcarnitine	9.93	448.3396		30	10	12	16	891	126900	62460	4882	10	41	38	687	217	24551	10791	147
Lariciresinol	5.89	359.15875	Lignols	1012	9404	12137	1331	1818	556	1302	1375	45674	13009	11071	2801	2679	1007	3405	6681
L-ASPARAGINE	10.759	133.05975	Asparagine and derivatives	1	5	7	4269	173	1486	52465	22	4	3	5	436	355	24	6578	63355
LAUROYLCARNITINE	5.358	342.26767	Acyl carnitines	17466	78882	21907	3766	10178	3058	8537	15760	24606	4395	3603	2097	8140	5613	6242	13767
L-beta-Homophenylalanine	5.371	180.10144	Beta amino acids and derivatives	49601	63501	75827	10465	17578	23923	4901	16911	147295	77452	107804	5256	8400	23460	3860	6321

L-beta-Homoproline	12.085	130.08487	Pyrrolidines	73	75	85	44906	15852	32465	30978	12146	242	467	64	74909	23352	25500	26945	26955
L-beta-homotryptophan-HCl	12.823	217.10898	Beta amino acids and derivatives	66	56	192	8918	9412	10876	7993	4511	96	62	280	10572	3004	6361	5296	6157
L-beta-Homotyrosine	2.898	196.09705	Beta amino acids and derivatives	5266	4820	3952	2572	8908	121969	19055	16248	854	2580	2396	34489	28726	66949	20866	30985
L-Carnosine	14.227	227.11284	Hybrid peptides	1647	3237	1406	34259	4581	1205	76275	1713	1102	1083	340	437	463	279	629	282
L-Citrulline	13.903	174.09109	L-alpha-amino acids	284	264	304	9077	6664	5594	4800	9790	721	283	574	2983	5559	7644	4316	5836
L-Cysteine Sulfinic acid	6.054	152.00473	L-alpha-amino acids	30	25	3	49209	40606	16551	40444	30637	53	15	7	48596	33698	21733	50417	34960
Leoidin	13.925	411.02322	Depsides and depsidones	72729	32434	31831	1	11	823	33	26	15143	51889	29661	26	53	14	10	67
Leprapinic acid	3.031	351.08258		21134	85009	72151	2013	1270	412	6190	1747	125446	17039	10514	2457	886	1935	3841	1048
Leucine	10.323	130.08958	Leucine and derivatives	2268	12612	362	48330	119560	74364	109005	99214	1737	812	1386	18878	79493	112202	89664	70408
Leucylproline	13.648	229.15506	Dipeptides	168	41	242	265941	6794	20976	200473	2864	273	154	126	18071	5336	2594	28834	280912
Leupeptin	3.127	427.28091	Dipeptides	16696	62556	35735	10866	14751	17394	10055	12548	2920	21899	14806	9605	4728	8654	13531	12174
Leupeptin Pr-LL	3.626	441.32175	Peptides	3216	7682	4690	13698	43638	5536	43257	31452	2691	3398	2412	18073	52385	16341	12480	60453
Levosulpiride	4.458	340.11694	Benzenesulfonamides	13292	8764	7581	2099	2334	16559	25005	47964	15989	9077	8466	36115	17221	2619	1657	9499
L-Glutamic acid	13.73	146.04651	Glutamic acid and derivatives	78967	82388	18195	21871	43280	25382	18678	27869	97153	20889	34949	8395	22176	28995	16703	33440
L-Homocarnosine	3.013	239.09264	Hybrid peptides	1627	1797	2529	20719	15929	12275	16108	31376	2539	1588	3792	24443	13179	8818	18079	9775
licoricidin	3.32	423.23074		32825	169828	384856	7608	147610	3371	15684	143698	23083	89166	162360	6491	3784	2226	2208	4765
Limonin	5.446	469.2572	Limonoids	12245	9286	6538	4209722	2940006	912970	1626	3177388	9823	12115	14747	835092	6359725	669387	1025989	3730
Linalool	6.846	155.15236	Acyclic monoterpenoids	36352	37101	48118	38	12	107	81	40	29753	50699	65256		8	124	44	70
Linoleic acid	1.542	279.23163	Lineolic acids and derivatives	110	128	98	985820	652688	1811051	893986	1523251	213	119	150	525806	863704	2158489	929271	687809
linolenic acid	4.344	301.20093	Lineolic acids and derivatives	777	1302	2790	2872	1351	1794	1264	547	1226	603	2032	1066735	1945	182	2570	1594
Linoleoyl Ethanolamide	1.14	324.29083	N-acylethanolamines	16652	16504	18763	33851	20781	21033	22523	22068	19920	19364	24783	3149	17432	27699	12279	29734
LITHOCHOL-11-ENIC ACID	3.209	373.27673	Monohydroxy bile acids, alcohols and derivatives	73856	253637	730561	77829	87074	125827	123947	67295	245621	270864	538589	91955	71194	109403	125744	162665
Lithocholic Acid	3.301	375.30222	Monohydroxy bile acids, alcohols and derivatives	521201	60854	512931	63629	212967	1402546	1016050	577127	759360	367754	1004416	263673	191174	1321571	1171543	760448
L-Leucine	10.02	132.1003	Leucine and derivatives	778	540	175	373730	105807	82784	620756	122085	2580	117	155	144831	133300	130529	199605	343568
L-methionine sulfone	9.741	182.05653	L-alpha-amino acids	31704	17270	62635	199	146	395	106	242	5574	1698	465	329	317	446	327	285
L-Norvaline	11.195	116.07345	L-alpha-amino acids	55	48	18	99846	45893	20543	82455	25131	83	64	91	28758	35582	20322	53801	61116
Lobaric Acid	0.735	455.1683		7	27	53	2263	3312	549	1036	27995	65	13	12	713	3448	1180	546	1805
Lobodirin	2.954	493.11365		179	413	163	821	3677	4149	7638	16188	486	960	336	613	4271	4623	17476	2976
Loganin	3.495	391.16541	Iridoid O-glycosides	375	697	768	1534	21606	10175	29504	1113	354	343	473	11118	1432	25656	2944	1660
Loggerpeptin A	7.837	1015.67883		8		7	7469	908	1292	2883	1484	7	5	7	6	2445	839	224	2713
Lopinavir (ABT-378)	6.774	651.36804	Valine and derivatives	7	13	29	2311	23116	215881	17172	19382	13	17	25	38040	8325	80348	23991	1994
Lotaustralin	3.096	300.12622	Cyanogenic glycosides	1114	1900	194	49369	55743	21814	29638	93264	1087	878	1119	5259	89069	22569	45635	71498
Lotusine	7.142	337.15167	Benzylisoquinolines	472	238	530	3259656	3598175	2074192	2868593	3370249	395	360	492	1744651	2960504	1865823	3061899	2107034
Lovastatin	3.769	443.22098	Delta valerolactones	1466	9514	2540	21324	18242	5929	29112	4110	143726	8126	5179	5589	4536	73691	19757	8081
LPC 16:0	7.926	540.33087	Lipids	6	3	9	16101	13420	10793	14089	51714	17	22	8	5624	96890	11883	11567	59271
LPC 18:1	7.756	522.35406	Lipids	37	50	14	692879	461653	559547	1245081	760908	32	24	53	14853	924211	686481	320732	676454
LPC 18:2	7.815	520.33801	Lipids	37	16	15	1269652	526669	411424	925511	553055	20	41	24	32724	530443	445324	357413	580265
LPC 18:3	7.857	518.32367	Lipids	9	37	8	46189	36479	32920	176424	41244	15	15	13	6305	40544	24919	27259	52295

LPE 16:0	8.088	452.27853	Lipids	23	9	23	205215	363732	70635	215303	552100	80	28	2	52133	1245910	52319	126554	763155
LPE 18:1	8.002	478.29404	Lipids	23	11	18	78878	167365	32345	257530	174219	19	8	9	14837	345339	19165	85057	512060
LPE 18:2	8.05	476.27725	Lipids	23	18	16	76492	67515	19862	138108	60149	11	7	15	16722	205842	20762	32485	87836
L-PIPECOLIC ACID	11.414	128.07317	Alpha amino acids	171	6	59	1279	1153	5257	55724	3085	71	110	182	1136	2794	1229	10788	27355
L-PROLINE	11.371	114.05556	Proline and derivatives	119	1468	683	79209	25886	12339	67212	24233	110	371	227	19671	16536	22257	29483	34603
LTB4_20-Hydroxy	2.895	351.22314	Leukotrienes	1672858	3833242	356463	168	379847	269721	360645	696199	42260	602945	114223	339068	319004	114348	1117	427891
LTD4	3.978	495.26602	Leukotrienes	782	2595	616	193835	87053	34068	203964	463667	6115	70	1198	47894	1536086	71023	181090	576395
L-Tyrosine	8.738	182.08134	Tyrosine and derivatives	637	1651	950	46539	34594	512682	49758	30113	238	190	45	215643	29085	340419	58979	23406
Lucidenic acid B	5.748	475.28189	Triterpenoids	317	266	163	4626	3734	226	26914	2546	221	246	103	1815	2802	640	3334	35533
Lucidenic acid D	2.985	515.25482	Triterpenoids	435	11	758	86292	42120	37762	28932	38675	586	263	497	19862	27180	22949	85431	18845
Lupinine	10.144	187.18068	Lupinine-type alkaloids	3	8	27	1075	6814	1606	4208	78495	20	7	27	427	213	2396	9240	5091
luteolin 4'-O-glucoside	4.772	447.14398	Flavonoid O-glycosides	18817	15665	13305	1697	1844	1216	896	1029	13942	33078	44415	1544	2202	1361	1551	1420
Luteolin-7-O-glucoside	4.223	447.13449	Flavonoid-7-O-glycosides	28805	30249	9772	12489	4411	4677	11084	1975	35142	63996	117052	3020	11615	1911	9403	4050
L-Valine	11.089	118.08871	Valine and derivatives	57290	41401	65822	133586	6618	53085	98944	4080	36975	372	39429	65214	5747	1825	2166	64815
Macamide B	4.3	346.33054	N-acyl amines	145812	147414	112516	109	150	339	228	68	96100	149327	231988	181	270	326	298	115
madecassic acid	3.308	505.34897	Triterpenoids	83423	184345	150995	582224	154930	186768	245112	367313	56471	58113	100903	2252505	615361	140717	429280	567738
maesopsin	8.95	310.89999	Auronols	884	1423	5	8	7	22	30		106	7995	4139	15	4	16	8	
Magnolol	6.256	265.12363	Biphenyls and derivatives	25096	62641	46539	2495	2194	794	1559	2508	122378	46446	20533	3620	1514	700	2332	1553
makaluvamine K	13.703	322.19833	Pyrrolo[4,3,2-de]quinolines	2404	3856	3955	43521	40476	15706	32293	20871	4584	3038	1853	50109	24591	3680	9001	11979
Makaluvamine N	14.318	265.99496	Pyrrolo[4,3,2-de]quinolines	601	1529	594	3945	21763	7160	20248	18268	1961	1335	958	15087	14478	7090	20290	17247
maltotriose	14.123	527.15277	Oligosaccharides	4834	2696	4721	29	52	21	81	86	8842	2723	2017	43	30	96	5	67
Man2GlcNAcFucGlcNAc	1.108	895.32672	Acylaminosugars	4795	5185	1839	37			33	182	5949	6004	6453	10	22	367		
mangostin	4.14	433.16888	8-prenylated xanthones	3075	6610	2209	2641	280	551	2377	1416	213852	5446	3539	3513	878	2073	767	606
Mannose 6-phosphate	6.51	259.01346	Hexose phosphates	15	7	14	31625	5606	3048	57713	7030	53	33	17	5733	1631	5126	28611	17098
Maoecrystal A	7.789	389.20139	Kaurane diterpenoids	15	11	7	122818	1025	1467	10553	71330	11	8	24	3692	121597	1204	123865	14989
Mappain	0.923	449.27744	Stilbenes	11034	16868	12857	112566	116356	54746	162306	123630	12278	10599	7501	29345	104289	44533	113545	115544
Matrine	5.794	249.18256	Matrine alkaloids	129	288	193	36104	121890	5038	30893	44447	264	180	120	19814	22092	7859	68702	104886
MCPA	6.322	199.03091	Chlorophenoxyacetates	10	92	46	22610	14909	16553	22050	9690	95	97	72	83371	26948	1929	1238	31535
Mebeverine	0.898	430.24387	P-methoxybenzoic acids and derivatives	15537	21268	26549	21354	22633	25591	38432	26602	20388	28250	34377	46495	31708	25084	32440	27536
Medicagenic acid	4.244	525.34021	Triterpenoids	2794	7550	4419	8608	6060	190745	8579	7346	3528	6627	3914	10661	3804	201825	16317	7992
Melezitose	11.338	503.19031	Oligosaccharides	85	60	26	11861	2586	499	805	58			118	17750	2457	226	479	1655
Melibiose	13.753	365.10388	O-glycosyl compounds	34879	6538	6681	716	6317	6371	9600	6833	353	2850	3911	2495	3954	5940	4555	13003
Menaquinone-4	5.376	483.26669	Menaquinones	851	602	1010	12668	9354	15430	4717	7329	308	158	338	3786	10868	7983	29665	526
Mesaconine	9.157	486.25967	Aconitane-type diterpenoid alkaloids	8	12	17	23490	15312	303813	13574	32537	21	24	17	9670	1947	232403	12130	15811
meso-dihydroguaiaretic acid	4.434	353.16513	Dibenzylbutane lignans	1062	1481	400	3673	55730	4078	4771	116689	1449	866	606	2816	55058	18158	10907	3458
Mesoridazine	6.567	387.15631		75	158	54	147057	73433	183745	17645	63932	74	84	172	48885	8045	82478	72040	16372
meso-Zeaxanthin	3.291	569.42749	Xanthophylls	7659	5132	7598	17732	14191	76720	33422	14550	6180	5792	5319	412	8746	47131	20308	10208
Metamitron-desamino	7.292	186.07896	1,2,4-triazines	4	2	11	11770	82592	4498	71224	22534	6	4	14	2903	52223	18687	81023	45032

Metazachlor OXA	9.945	272.09756	Alpha amino acids and derivatives	17	46	127	19973	9726	6344	14090	7486	37	35	15	13992	12690	14418	12737	15838
Methadone	3.298	310.23721	Diphenylmethanes	16006	21958	13316	42204	77637	25035	123320	69385	23032	8961	8860	3000	63529	22480	78949	73051
Methanesulfonate	16.059	94.98158	Organosulfonic acids	27219	20766	15899	91229	122032	115054	119188	120850	10241	10721	20798	131959	118974	79275	120392	126606
Methioninesulfoxide	3.936	164.0376	Alpha amino acids	34874	14210	19690	932	3901	3106	150305	2853	7130	13131	4744	3499	2192	1253	23424	83178
Methoxyfenozide	6.661	367.21942	Benzoic acids and derivatives	130	389	340	31091	27861	13615	26377	23958	579	169	51	36557	25878	12682	34262	23689
Methoxy-MCA-Albicidin	1.121	857.29761		8906	7370	8586		134		13		12049	7295	17765	73	87	77		195
Methyl 3,4,5-trimethoxycinnamate	13.748	291.01129	Coumaric acids and derivatives	6664	6803	6917	64	43	68	55	125	7468	6939	7625	25	132	90	135	113
Methyl Deoxycholate	0.922	407.32718		5369	7164	5289	10950	6043	2630	5523	6175	2521	4625	3635	1982	10047	3873	4611	7945
Methylgallate	5.736	183.03705	Galloyl esters	101	129	110	90583	47522	13178	241	34855	89	187	244	72249	43348	13324	52625	324
Methyl-mappain	1.59	463.29147	Stilbenes	3668	3107	2444	15031	65217	105601	39990	42716	2755	1109	1071	9299	56352	183342	89708	54702
METHYLPREDNISOLONE	5.548	375.22458	21-hydroxysteroids	381	284	337	995757	348884	4245	5985	15435	405	88	207	411519	31780	3558	91125	1300
Metolachlor morpholinone	1.422	234.1478	Phenylmorpholines	462	133	187	35673	48833	100320	56197	95626	185	222	106	57109	126444	121025	188547	59987
Metribuzin	6.775	215.09898	Aryl thioethers	92	268	182	15435	54267	1216	2376	1350	149	147	194	46099	15358	2353	4499	4131
Mevalonic Acid Lactone	10.503	148.09546	Delta valerolactones	125	35	71	1379	365	911	31480	320	7	118	24	292	385	135	3135	11040
MGMG 16:3	3.749	531.2868	Lipids	7317	2538	3307	4251	7114	151697	2792	19191	3816	871	966	11772	9520	26679	1586	5389
Microcolin E	1.569	752.51337		12780	31345	37154	2610	1236	733	5567	2229	7539	116199	742870	198	4088	1720	2965	1479
Microcolin F	3.505	768.60944		3563	6812	13505	1138	2814	109	827	2102	2030	4653	4584	174	788	773	214	2756
Microcolin G	3.94	726.41968		276	842	177	16728	14784	46702	25895	19049	250	92	500	6641	5999	69784	27961	17582
Microcolin M	10.958	691.29175		7	23	9	2589	1998	65792	1111	4727	23	6	23	579	4117	46291	888	1952
Miltirone	3.407	305.1543	Tanshinones, isotanshinones, and derivatives	13016	44195	46045	1102	3092	5444	1451	5003	11164	81169	12575	3431	983	5379	2816	1284
Miquelianin (=Q3G)	11.2	479.06113	Flavonoid-3-O-glucuronides	2	28	2	15	34	6	15	18	15076	16845	6329	14	21	15	11	43
Mitragynine	4.551	399.22351	Corynanthean-type alkaloids	1124	2286	1148	315724	185299	74506	315369	229675	578	720	1275	11361	160754	97596	312473	216179
Molassamide	10.44	985.6236		5	4	1	32	392	602245	6828	852	4	1	2	364	252	224394	1145	412
mollugin	5.895	285.11777	Naphthopyrans	171	966	325	54832	29811	3678	21293	10625	705	554	185	28001	32166	1534	19804	22740
Momordicoside A	5.364	839.49719	Cucurbitacin glycosides	78	29	9	1065	761	71549	683	696	5	34	3	224	435	44065	2551	375
Monoisobutyl phthalate	3.84	221.08226	Benzoic acid esters	27633	20738	29669	1570	2536	1856	2861	1436	18626	23474	51217	981	4156	529	2383	1460
Monoolein	4.415	357.30289	1-monoacylglycerols	3476	1867	2114	962	1326	301504	191638	70159	302	1790	1246	930	6230	386563	192101	118267
Moroidin-[QLLVWRSH]	10.451	509.27277		14	87	80	4325	3660	53747	5036	7530	62	25	25	3600	3190	39262	4366	5037
Morpholine	5.165	88.07541	Morpholines	2299	1579	1950	1111	3056	58252	76203	3525	2447	1775	2303	5013	2494	13370	53458	1874
Mucate	6.327	209.01657	Glucuronic acid derivatives	11	39	70	1190	1111	500	18143	864	51	79	27	1283	1062	508	700	23213
Mundulone	4.595	457.28043	6-prenylated isoflavanones	1504	1409	3760	12537	3813	4288	17451	7669	1103	3362	1713	371156	7413	1242	10967	7593
m-Xylene-4-sulfonic acid	5.607	185.01642	p-Methylbenzenesulfonates	9917	1570	355	25102	49005	13768	223	40016	17935	867	375	19752	11416	13638	49639	363
Mycophenolic Acid	4.5	319.11795		2649	3931	1665	105933	71201	34745	22315	37929	3048	4767	3339	95310	71783	12379	25900	26078
Mycosporine serinol	13.558	262.12756		318	53	121	23131	17040	9038	15835	11686	187	175	187	28349	11916	4392	9789	11477
Myristic acid	2.964	227.20314	Long-chain fatty acids	323136	260331	377400	27207	30659	15056	18888	12609	199713	883446	518031	24942	27392	10127	13263	21363
Myxovirescin A	4.52	624.44299	Macrolide lactams	19435	10091	9891	4163	1556	551	1904	1890	12861	1958	3434	2013	1775	1817	1216	1118
N-(3-Methoxybenzyl)oleamide	3.477	424.31842	Anisoles	1844	2284	2840	4654	14197	103557	82332	36347	2325	2076	1017	1471	31516	66489	27972	21418

N-(3-Methoxybenzyl)palmitamide	1.603	376.3186	Anisoles	18525	11906	10056	57	1773	14419	13275	19988	15932	6642	8322	410	764	14801	7467	7696
N-(9-oxodecyl)acetamide	4.536	236.17078	Acetamides	198	222	333	357	1624	91719	16110	406	622	508	983	2050	1639	2152	2822	27398
N,N-Diethyl-4-hydroxybenzamide	1.429	194.11795	Benzamides	2010	704	3143	1388	7068	123158	7494	7134	660	656	14104	54967	8845	66683	5848	4674
N,N-Dimethylaniline	3.306	122.09639	Dialkylarylamines	171994	171330	424625	49882	83515	40359	61856	70140	300805	243475	90012	86245	58038	40846	34045	34235
N,N-Dimethyldodecylamine N-oxide	6.795	230.24603	Long-chain alkyl amine oxides	30437	32206	24147	471	120	149	504	43	27674	35774	67817	213	121	146	183	99
N1-Acetylspermine	3.018	245.2231	Acetamides	30506	47622	25211	1622	900	2210	2202	2315	21519	29679	36083	279	1628	373	4784	1642
N4-Acetylsulfadimethoxine	10.261	351.08521	Benzenesulfonamides	46	25	24	35474	36848	16121	18882	26193	101	19	50	24986	41103	11595	31934	39688
N-Acetyl Mesalazine	3.738	194.0529	Acylaminobenzoic acid and derivatives	3296	4107	645	13405	1750	4642	116888	3363	1163	4456	307	9113	5230	2207	11566	73840
N-Acetylaspartic acid	13.649	174.04027	Aspartic acid and derivatives	20261	19228	8432	191	348	176	94	199	14470	11744	8761	76	83	31	111	132
N-Acetylcaprolactam	1.494	156.10152	NA	6870	55375	19288	55809	27033	456396	29442	5066	67937	36905	33997	148967	14790	261880	41796	29164
N-Acetyl-DL-glutamic acid	13.401	190.07051	Glutamic acid and derivatives	90	189	41	17413	56940	21185	10679	14357	141	142	95	18203	7001	14973	8351	21204
N-ACETYL-DL-METHIONINE	5.997	190.0535	Methionine and derivatives	41	62	33	43247	26569	30462	36692	36622	138	11	40	24219	63526	42292	33058	49642
N-ACETYL-DL-SERINE	10.372	146.04652	N-acyl-alpha amino acids	31402	161080	4998	40	188	299	211	102	4347	93	42	203	232	82	27	29
N-Acetylglutamic acid	13.431	188.05681	Glutamic acid and derivatives	79	34	28	198358	474392	194656	70876	110478	68	25	102	121690	46790	116831	42054	192830
N-Acetylglycine	9.29	116.03784	N-acyl-alpha amino acids	64	77	83	1863	9250	2016	3539	3903	13	49	20	10953	9092	1777	3717	14515
N-Acetylleucine	5.911	172.10101	Leucine and derivatives	10	335	59	29687	12324	15742	41837	13798	167	41	14	19011	26225	31521	24402	40173
N-Acetylmuramic Acid	10.413	292.10184	Acylaminosugars	85	89	45	32856	32756	12783	8604	20101	138	26	91	11474	17122	21039	10367	13637
N-acetylphenylalanine	5.655	206.08362	Phenylalanine and derivatives	330	190	863	32116	15836	1025	666	6693	103	1044	79	28494	17473	4176	19543	330
N-acetyltryptophan	10.925	245.13768	N-acyl-alpha amino acids	137	84	69	1115	6081	60493	5340	25436	2090	108	110	1286	1396	50056	1030	4949
Nakijiquinone B	8.915	444.28552		44	64	69	724	1083	7969	3146	97729	84	107	219	1537	845	14445	1447	1285
Nalidixic acid (NegGram)	6.141	233.09093	Naphthyridine carboxylic acids and derivatives	23	39	18	55728	1585	166	2433	359	94	36	35	29695	342	828	901	780
NALPHA-ACETYL-L-LYSINE	13.857	189.12405	N-acyl-alpha amino acids	261	116	48	15468	40805	15442	15191	22665	135	44	144	21841	11569	10695	11463	11577
Naproxen	7.883	231.1105	Naphthalenes	91	32	67	33913	26380	10283	25848	21504	51	29	23	43189	19470	9207	18938	15007
N-BENZOYL(D5) GLYCINE	6.583	180.06442	Hippuric acids	44	19751	25726	790	714	662	520	1413	133	30696	913	419	351	477	520	996
N-Benzyloxycarbonylglycine	8.73	208.06146	Benzyloxycarbonyls	832	61491	38208	107	40	196	350	2984	60	10286	2610	401	215	87	108	113
N-Carbamoyl-L-Aspartic acid	5.524	175.02834	Aspartic acid and derivatives	10715	7635	16977	192749	114406	19556	194	108613	7941	4303	1373	62242	281259	14562	131294	317
N-Cinnamoylglycine	5.814	204.06569	N-acyl-alpha amino acids	115	330	181	18865	596	1033	1629	461	360	140	61	21388	841	808	919	6673
neoandrographolide	3.553	498.31738	Diterpene glycosides	11322	3978	11692	31711	69848	91252	146019	198324	33688	2821	2056	6746	60832	114738	171301	53340
Neoruscogenin	3.315	429.31265	Triterpenoids	50215	35081	32533	32918	31202	52803	45379	107352	29453	21491	24149	5705	6119	126470	66506	41240
Neotame	6.399	379.23157	Peptides	115	557	218	40580	47127	100093	36003	34661	122	45	178	42974	15909	94058	43422	40730
N-epsilon-Acetyllysine	13.865	187.10817	D-alpha-amino acids	158	378	912	4681	13615	4461	6382	5922	178	148	116	5651	4103	3381	2407	9272
Nerolidol	7.075	245.18799	Sesquiterpenoids	8	44	14	52768	9702	83780	24397	19527	12	17	47	62421	41210	41507	68638	60682
N-Formyl-L-Methionine	5.351	176.03694	Methionine and derivatives	1181	556	1746	732	3124	193	386	103123	5439	494	369	200	5242	619	972	726
N-Fructosyl pyroglutamate	11.901	290.09317	N-Fructosyl amino acids	108	114	108	719	1118	12585	226	388	119	64	64	411	416	48948	155	361
NIACIN	6.461	122.02653	Pyridinecarboxylic acids	9	8	19	105391	109091	38270	103216	120536	24	46	40	31857	167800	48130	103429	164860
Nicosulfuron	1.661	409.08774	Pyridinesulfonamides	10322	5456	3304	116	170	236	386	217	41059	4504	9185	253	341	162	111	193
Nicotinamide	3.215	123.05648	Nicotinamides	27162	42654	12000	66235	6731	11032	13975	5516	58249	17519	2918	7646	27330	9037	27339	4804

N-Isovalerylglycine	6.007	158.08435	N-acyl-alpha amino acids	9	11	21	305984	33574	2986	12217	8725	38	23	19	171713	24629	2125	10863	9349
N-Methyllysine	4.404	159.10246	L-alpha-amino acids	223371	197309	77998	799	1271	1351	888	2275	197349	74035	250117	2033	5332	465	704	1634
N-Methyltyrosine	13.586	196.09663	Tyrosine and derivatives	83	79	36	266783	242128	77786	165636	86158	37	51	72	331017	162390	25886	65726	47091
N-Octanoylsphingosine,D-erythro	8.546	426.40744	Ceramides	9	11	7	16596	6275	3471	18522	3903	7	6	7	9	17311	2723	15736	13890
N-omega-Acetylhistamine	6.502	154.09872	N-acetyl-2-arylethylamines	55	401	84	2004	752	2047	36212	1592	118	174	135	24037	920	966	7347	20068
Nonic Acid	13.098	226.98581	Medium-chain fatty acids	61735	125484	51357	56	31	15	10	62	61919	52878	184081	76	25	35	85	22
norambreinolide	1.117	251.18901	Naphthofurans	50410	47123	88009	6227	1797	688	112	3281	26530	59760	72617	6114	3188	1012	3949	3557
Norleucine	9.95	130.0883	L-alpha-amino acids	8	28	27	545502	219147	134652	281255	84674	288	12	33	121378	157196	196560	98647	147197
Norrhizocarpic acid	10.968	454.12231		5	14	39	30456	633	661	12261	69	29	12	4	15185	1499	129	2387	16794
Notoginsenoside Fe	6.381	939.50031	Triterpenoids	10	19	19	1122	1742	15901	744	8298	6	4	7	2279	2074	59662	2750	1525
N-Tigloylglycine	1.102	158.09598	N-acyl-alpha amino acids	270666	155564	226607	199	416	6688	269	1612	45317	42087	329020	2061	146	406	125	34
O-Acetylsolasodine	8.476	456.36411	Spirosolanes and derivatives	44	16	47	17205	18784	6727	15381	10495	11	20	9	73	10396	6328	19179	12294
Obacunone	3.309	455.22043	Limonoids	801	1220	1321	9960	10710	7638	14961	22877	18341	721	501	7005	16716	80106	3767	63088
Obtusaquinone	10.182	253.0957	P-quinomethanes	35	74	47	22802	836	10327	64439	21134	47	107	26	272	56	14732	60577	52846
Ochrolifuanine A	10.454	439.27676	Harmala alkaloids	49	48	131	38296	41103	1074	5073	777	35	51	87	105497	18963	1356	1845	8177
Octadecanedioic acid	4.197	313.23999	Long-chain fatty acids	29353016	33400288	41659504	51860	60022	10354	38544	21852	15684436	27109800	21040766	32907	37843	22931	45006	58880
Octyl acetate	2.943	195.123	Fatty alcohol esters	40751	68016	192721	239	684	1180	2151	445	69318	210825	110274	17194	567	855	917	1000
Oleamide	1.114	282.27997	Fatty amides	2582598	2503199	1900020	1827831	1766570	1439451	1551661	1552302	1991421	1822181	1557975	318529	1332832	1586272	1208433	1651477
Oleanane -4H, + 2O	1.894	441.37222	Triterpene saponins	15968	9764	10283	9298	2032	2521	9664	4577	47066	15196	11515	246	2474	1153	12618	4535
OLEANDOMYCIN PHOSPHATE	5.434	688.42474	Aminoglycosides	1564	1346	13306	48971	81149	6839	21428	34179	29	43	156	2242	52357	3658	20145	109358
oleanolic acid	1.529	457.36639	Triterpenoids	13532	28609	24674	50553	46385	123399	57321	72539	34746	18021	37532	1341	37794	115306	75396	49762
Oleic acid	1.533	281.2496	Long-chain fatty acids	173	62	85	736891	986929	985667	824603	910075	197	76	49	253602	513226	1219947	931796	663522
Oleoyl sarcosine	0.901	354.28806	N-acyl-alpha amino acids	66761	75905	110760	5372	4962	7382	8626	4136	80643	112827	137094	19369	5017	6024	5562	7214
Olivetolcarboxylic acid	3.141	223.10403		506	4214	105	1245	74933	18393	1284	83174	13943	596	353	2411	10490	24043	1021	941
Olmesartan	3.256	445.18448	Biphenyls and derivatives	2731	5388	2964	17305	35043	54178	49284	78441	7328	1107	2072	60260	19550	41185	114886	44305
Ophiopogonoside A	0.9	419.26764	Terpene glycosides	14265	16881	13147	191597	187698	192027	295572	213598	9042	10982	10434	9930	198615	165222	294084	282798
O-PHOSPHO-L-SERINE	1.549	183.98996	Alpha amino acids	7349	9448	9114	102	198	103	47	80	6068	11723	16907	126	163	40	28	114
Ophthalmic acid	13.881	290.134	Oligopeptides	207	102	14	69025	54395	24702	29020	41531	118	213	2158	60353	29333	15357	43869	16941
oregonin	3.478	477.30472	Linear diarylheptanoids	2272	2694	5206	919	84883	82119	10756	33968	8238	3176	6463	15101	20243	85150	18394	4870
ormosanine	2.95	318.29886	Ormosia-type alkaloids	19954	19599	2338	34833	32456	23144	36819	10690	1630	1951	3947	475	17291	21015	43118	41776
Ornibactin C4	6.413	681.33612	Oligopeptides	71	9	15	3465	4907	33566	4441	2228	55	105	26	2192	1736	28855	5053	1377
Ornidazole	3.725	218.01906	Nitroimidazoles	2589	5359	5984	25927	125554	136919	83004	11550	5937	5650	2741	87876	51798	57161	46035	63638
Orotic acid	6.18	155.00998	Pyrimidinecarboxylic acids	74	21	8	34484	32492	17700	88592	43624	103	12	16	8426	45287	4103	3114	141481
osajin	3.632	403.26025	6-prenylated isoflavanones	2210	28900	17618	334	1180	63	638	586	4220	5683	4078	4401	1374	1546	1605	2235
Oseltamivir carboxylate	4.475	283.15881	Gamma amino acids and derivatives	19818	37112	14586	4332	2852	1045	3967	1557	73926	10498	10888	2255	3338	1648	5490	1874
Otenzepad	3.17	420.24765	1,4-benzodiazepines	340	711	335	456	145871	25549	45671	21400	1249	12130	381	99122	62597	30089	58695	42528
O-Tigloylgymnemagenin, 21-	4.1	611.38855	Triterpenoids	2509	505	1494	41232	79683	1255165	184707	94266	2686	1018	633	167847	39261	1032600	169075	208577
Oxyacanthine	10.905	609.32611	Lignans, neolignans and related compounds	111	100	77	73496	67796	268761	75739	70134	468	403	214	79628	17168	286701	75946	33315

Oxymetholone	4.132	331.23373	Androgens and derivatives	10997	46959	4878	1338	7345	521	918	12439	1130	601	779	574	1203	2773	1607	2745
Palmatine	4.87	352.15555	Protoberberine alkaloids and derivatives	18377	10723	13412	42517	39831	2934	22712	7271	9435	12764	2791	7736	15732	2912	18407	23959
Palmitic Acid	3.46	255.23413		442096	381418	185312	716	1480	716	393	961	92307	377009	114183	438	565	724	1500	3650
Palmitoylcarnitine	7.283	400.33942		84	236	172	24148	4018	4500	17088	3686	157	102	79	94	710	3991	2153	2132
Panaxydiol	3.329	283.17508	Long-chain fatty alcohols	26615	83767	103999	3261	1015	895	1500	1793	18559	170671	31117	2092	902	1422	1027	1323
Pannaric acid	16.851	315.04364		157382	216305	97417	204	290	327	149	174	143653	147733	251166	147	301	240	129	273
Pantethine	3.191	553.25977	Beta amino acids and derivatives	1502	4229	2196	2545	14370	5704	1985	11031	4743	2157	59054	10789	8654	10695	3274	7237
Pantothenate	7.271	218.10568	Secondary alcohols	7	5	3	89354	25495	22925	94723	11603	11	5	2	6883	18526	28781	37509	87506
papaverine	1.449	340.17566	Benzylisoquinolines	864	79	196	10025	7830	271428	33762	8236	57477	1750	1520	73159	5448	103670	27656	4835
Paracetamol	5.975	152.07246	1-hydroxy-2-unsubstituted benzenoids	206	193	507	9678	28766	4912	5176	6467	2883	429	263	8566	15811	6617	69997	6642
PC(16:0/11,12-EpETE)	6.193	854.57727		14	4	4	365	968	17993	1154	520	14	14	3	188	941	17888	946	89
PC(16:0/12-HETE)	4.835	856.55914		1089	1549	1665	518	792	13024	397	686	172	1160	708	214	200	13243	393	292
PC(18:1/13-HODE)	5.037	858.57947		494	560	1839	3356	8318	98383	6974	7310	1921	899	1419	1286	3746	80654	6396	2872
p-Coumaryl alcohol	3.495	149.06239	Cinnamyl alcohols	24642	420164	106544	126686	579694	151031	1200062	1032264	86777	153924	19725	3495965	2902228	250635	798063	1445029
Peimine	1.504	432.34366	Cerveratrum-type alkaloids	6783	8000	25977	7819	3310	5018	5984	1222	3319	39465	21377	270	25273	3641	7290	2564
peiminine	4.049	430.33167	Cerveratrum-type alkaloids	24922	29698	22168	4579	1915	5368	3033	5603	20348	11262	13694	693	7105	5945	3343	4909
Peimisine	0.893	450.29501	Jerveratrum-type alkaloids	9785	13346	14659	46319	52484	16027	63563	46402	8181	11363	6811	10820	52034	15967	45475	61012
Pellasoren A	7.325	432.31042	Delta valerolactones	9	102	36	15415	18507	317356	30570	29523	51	3	26	19361	41876	273591	28915	26538
Pelletierine Hydrochloride	1.417	178.10829	Piperidines	19364	17775	25218	85	28	200	50	108	5349	19091	18767	48	117	37	27	98
Pentadecanoic acid	1.585	241.21982	Long-chain fatty acids	780	302	506	1417764	1038633	159374	456921	268446	310	268	97	592555	539274	141572	497064	765761
Perindopril	3.264	369.24179	Dipeptides	28805	19843	43364	50926	58642	270675	134907	200751	21187	39571	11618	14242	62359	283965	156174	125391
Perivine	5.948	339.16861	Vobasan alkaloids	392	486	145	1499571	2906299	759608	1179721	1517304	669	129	853	671854	1313849	1382645	1765752	727205
Perseitol	5.979	211.02885	Sugar alcohols	171	53	41	333104	99165	25412	2592	37138	120	46	40	441196	143221	27326	92947	14787
PFCA-unsaturated	13.759	374.98349	PFSA	27030	45063	20327	1350	2064	2335	770	1026	33360	26678	20620	384	1124	939	590	653
PFSA-pentafluorosulfide	14.142	506.91162	PFSA	13	12	34	42130	30027	41772	24282	42245	34	18	12	19292	31991	49612	13635	27694
PFSA-unsaturated	14.096	310.93619	PFSA	23	30	57	115707	153931	155077	81174	68656	43	86	41	123643	154270	171364	84981	148968
PFSM-amine	11.24	985.00861	PFSA	7	2	2	15	12	11			24857	29422	39847	23	11	11	11	21
PFSM-ammonio	11.294	872.03436	PFSA	2929	2	2		21		11	16	10438	11045	12936	23		10	7	8
Phencyclidine	12.374	244.20201	Aralkylamines	115		47	31936	24805	12701	19798	21587	99	83	177	22423	78077	14516	6774	37367
Phenethylamine	17.003	122.09431	Phenethylamines	211393	243221	198043	56285	136369	159052	95792	80297	217063	221612	212549	83091	124533	116590	24232	22948
Phenylacetaldehyde	9.873	121.06393	Phenylacetaldehydes	45	57	53	15716	23420	4483	34820	10017	18	44	10	16198	18538	3868	29180	11449
Phenylacetylglycine	5.802	192.06706	N-acyl-alpha amino acids	11	40	24	649548	4138	808	393	1249	52	10	22	1121240	2548	419	2805	2673
Phenylalanine	7.203	166.07191	Phenylalanine and derivatives	732	633	625	26729	5530	7744	418950	690	27	908	291	10658	5193	3085	75400	315756
Phenyl-beta-glucopyranoside	3.083	255.06927	Phenolic glycosides	44623	9314	7237	7666	6835	5762	3395	15891	1781	7071	1579	17298	7124	5907	7568	3012
Phenylethanol + Hex-Pen	10.795	461.16052	Alkyl benzyl hexosides	7	26	25	25773	302	2628	73082	278	47	33	16	3652	1861	241	11971	139920
phenylethylamide 359	3.275	360.30768	N-acyl amines	244906	419949	183668	3547	3722	56920	27707	33212	182062	155700	139116	1522	2644	65198	36332	17699
Phomalone	4.212	253.11395		3682	3246	1001	12784	91508	29202	23245	47659	2693	2123	936	9037	60774	17028	49068	10788
Phosphatidylcholine 14	3.168	760.52112	Phosphatidylcholines	10781	26354	12607	13626	47341	67744	19275	32331	1469	5536	1261	29173	30700	69003	20425	62774

Phosphatidylcholine 15	5.252	822.58069	Phosphatidylcholines	458	332	293	403	1978	24272	1903	1372	628	133	839	138	267	23862	904	948
Phosphatidylcholine 16	3.187	762.53821	Phosphatidylcholines	5589	16100	22853	6367	30359	40861	7428	19664	1128	1101	1120	25353	25245	91165	17285	43139
Phosphatidylcholine lyso 16	7.64	524.33801	2-acyl-sn-glycero-3-phosphocholines	26	6	17	908	1953	1705	1261	6746	35	3	16	698	7960	1392	919	4693
Phosphatidylcholine lyso 17	7.56	538.35217	2-acyl-sn-glycero-3-phosphocholines	12	15	35	167158	3643	5519	6721	8998	10	30	16	2668	8852	3314	3335	12018
Phosphatidylethanolamine 16	3.928	738.50146	Phosphatidylethanolamines	18813	12718	10993	7868	8246	3132	261	425	7923	2928	3577	298	6824	5850	1332	9052
Phosphatidylethanolamine 17	3.217	778.53247	Phosphatidylethanolamines	4068	3614	3132	6457	14027	37173	3900	12672	995	4520	888	24789	9852	47069	4208	6849
Phosphatidylethanolamine 18	5.309	764.52551	Phosphatidylethanolamines	13841	23720	10704	2611	4367	1963	1890	1981	381	10908	13785	2652	1523	1549	2329	1586
Phosphatidylethanolamine 20	3.88	810.5094	Phosphatidylethanolamines	248	174	171	389	511	22811	7401	2938	114	39	113	1297	2440	36106	14207	4707
Phosphatidylethanolamine alkenyl 16	5.012	746.51282	1-(1Z-alkenyl),2-acylglycerophosphoethanolamines	53179	438556	32132	3767	10104	2752	8684	6363	11733	43092	24235	2465	6305	2963	6376	53408
Phosphatidylethanolamine alkenyl 18	5.286	728.55444	1-(1Z-alkenyl),2-acylglycerophosphoethanolamines	19588	22750	14913	4009	8332	2097	1943	2217	923	14321	19367	3944	311	2135	1288	5667
Phosphatidylethanolamine lyso 16	7.598	452.2789	2-acyl-sn-glycero-3-phosphoethanolamines	26	26	13	5217	2291	2152	2078	2541	4114	688	11	1412	26298	1419	988	3775
Phosphatidylethanolamine lyso 17	8.038	466.29611	2-acyl-sn-glycero-3-phosphoethanolamines	335	146	268	18713	40126	5437	9866	33828	57	118	230	6329	113520	5637	5046	53153
Phosphatidylethanolamine lyso 20	8	498.26526	2-acyl-sn-glycero-3-phosphoethanolamines	166	57	32	1553	5530	1092	67092	1216	70	66	36	1228	1136	204	585	2438
Phosphatidylethanolamine lyso alkenyl 16	2.939	436.27527	Glycerophosphoethanolamines	122	295	392	25911	18920	28839	50792	37692	82	225	113	29496	19521	25085	140198	59164
Phosphatidylethanolamine lyso alkenyl 18	7.68	462.29822	Glycerophosphoethanolamines	370	865	901	223258	197984	37295	90392	220837	1471	386	548	32605	190012	27565	63577	260884
Phosphatidylglyceride 16	3.953	745.50952	Phosphatidylglycerols	40886	6856	15070	313	216	490	306	132	1311	4349	4044	210	285	330	379	258
Phosphatidylglyceride 18	4.722	819.53955	Phosphatidylglycerols	596	542	686	296	8527	253735	54250	57147	305	427	566	2787	1263	292382	52201	22382
Phosphatidylinositol 16	5.872	881.50964	Phosphatidylinositols	29	17	67	2133	737	16133	1849	4661	72	31	81	979	1017	10773	1643	397
Phosphatidylinositol 18	5.413	907.51251	Phosphatidylinositols	121	92	137	3097	6046	423	15	4557	50	87	126	12	12011	452	408	23
Phosphatidylinositol lyso 16	3.125	571.29584	2-acyl-sn-glycerol-3-phosphoinositols	3642	5110	4012	29779	42342	46725	25638	36647	6036	2674	7748	45924	23786	91585	17943	15697
Phosphatidylinositol lyso 18	4.239	597.284	2-acyl-sn-glycerol-3-phosphoinositols	694	1080	690	1546	2348	8038	2833	2659	202	710	1677	3308	3659	58168	4851	1543
Phosphatidylinositol lyso 20	11.038	619.28088	2-acyl-sn-glycerol-3-phosphoinositols	28	64	62	7822	8495	11208	5549	20270	30	5	5	9340	5970	11962	8381	4541
Phthalamic acid	2.96	166.04926	Benzoic acids	14552	15976	6351	10544	6934	21818	61173	65429	16130	13847	1156	6498	1986	23452	18082	50017
Phthalic anhydride	0.882	149.02341	Phthalic anhydrides	911163	727234	1031248	159003	143561	105912	148632	154080	624719	954673	1255451	148053	114024	94817	158326	139955
P-HYDROXYBENZOIC ACID	3.062	137.02493	Hydroxybenzoic acid derivatives	2338	5529	1575	2820	11745	9838	23436	11636	6254	1598	8493	20451	16872	13530	9105	12164
Phytolaccagenin	8.44	555.33234	Triterpenoids	21	60	26	68578	62916	68044	87006	116390	49	13	45	573	69986	65561	96884	47377
PI(16:0/10-HDoHE)	4.231	897.51093		141	592	215	857	1131	58321	311	23740	834	385	179	870	1333	81553	1295	770
PI(17:0/15-HEPE)	6.798	885.52448		8	34	43	2432	113	66401	1360	414	16	45	31	223	156	7370	2506	297
PI(18:0/12-HETE)	11.584	901.51672		172	89	215	91	20	28499	25	7	87	323	69	177	13	13431	63	27
PI(18:0/9-HODE)	8.16	877.51764		227	130	331	1244	5868	139	164	6990	925	350	304	99	45175	174	118	11116
PI(18:1/16,17-EpDPE)	5.409	923.51544		42	29	174	9824	7145	1005	357	9218	221	47	33	112	26114	285	2119	21
Picrotin	6.278	309.10107	Furopyrans	111	83	90	47436	16240	1284	1673	12566	251	64	55	26460	6521	1997	3572	10272
Pilocarpine	12.991	209.12947	Alkaloids and derivatives	108	110	55	76233	85168	14697	57378	53423	250	69	91	109883	47655	11585	56014	28346
Pinoquercetin	5.491	315.04068		35637	21364	35513	1768	1119	943	887	3114	4357	36178	22726	24096	1413	692	1242	1165
Pinostrobin	6.222	268.99756	7-O-methylated flavonoids		5	6	10551	281	6827	2191	255	41	8	4	5737	410	86	255	72360
Piperanine	4.939	305.185	Benzodioxoles	2417	23270	11974	100015	189389	546675	80723	295453	5584	5046	2463	7884	66016	835523	55290	147690

PIPERIDINE	10.906	86.09565	Piperidines	297	229	308	66535	122809	56153	219337	183817	398	115	230	172854	328640	55753	153474	102116
PIPERINE	5.321	303.16995	Alkaloids and derivatives	165	218	283	54927	106201	124617	35794	196956	420	325	248	8308	28295	162141	33077	60995
Pleiomutinine	5.533	615.36206	Pleiocarpaman alkaloids	81	97	109	36286	47993	9017	33100	24793	296	50	132	15070	21140	2825	21865	20080
Podototarin	3.236	571.40594	Diterpenoids	1223	3295	3990	3850	2183	86740	4936	4964	8791	1283	2975	2813	1746	56282	6540	8333
Polygalic Acid	5.394	511.29941	12-alpha-hydroxysteroids	3651	740	221	133899	128090	117197	697028	165888	147	83	225	6434	261312	56978	255515	556937
Polyphyllin A	5.007	599.3645	Steroidal saponins	50364	21337	96816	4296	6443	17282	5564	8372	67741	43554	26271	26039	3847	8255	6995	6361
Polyporenic acid C	8.512	483.33533	Monohydroxy bile acids, alcohols and derivatives	13	26	23	12670	10375	47506	12230	4885	52	23	8	7531	19294	42160	16369	6489
Pomiferin	3.726	419.25058	6-prenylated isoflavanones	1164	9658	24029	1251	1185	2299	1729	2389	1307	3685	214	2836	1985	966	2651	984
Precyasterone	3.302	521.2926	Withanolides and derivatives	1944	1490	962	41494	50560	29748	50123	55073	3337	1664	620	19236	57895	32196	45903	33577
pregn-4-ene-3,20-dione	2.911	315.21463	Gluco/mineralocorticoids, progestogins and derivatives	640	1013	1425	1002698	483370	46086	615665	201834	1691	1039	319	51496	303475	28501	787099	760496
Pregnenolone	0.952	317.24966	Gluco/mineralocorticoids, progestogins and derivatives	4340	22112	5983	53252	30998	9966	22371	28375	2849	1042	3098	17070	42075	9244	33695	5502
Pristimerin	5.778	463.27057	Triterpenoids	8779	802	3622	103350	67900	5460	4166	1946	507	5874	3379	44097	77333	2193	11430	28308
Procaine	12.752	237.15843	Benzoic acid esters	79	64	139	1658450	1740005	484297	1089864	1087808	413	98	172	3042520	951922	373102	1000652	759286
Progesterone	0.935	315.28943	Gluco/mineralocorticoids, progestogins and derivatives	4687	38323	7551	9483	25181	12028	22225	29169	16688	5067	7202	1502	7515	9228	21874	17538
Proline	11.359	116.07024	Proline and derivatives	119	99	990	349186	121523	44092	241766	77856	242	134	83	102535	64482	75975	101860	100900
Prolintane	5.154	218.21086	Amphetamines and derivatives	71606	241709	229123	43		34		29	152543	308931	370851	30	74	86	11	105
Propachlor ESA	9.219	256.06	Anilides	46	88	28	28905	20280	5957	11597	9939	39	182	169	27112	30714	10896	18615	24999
Propionic acid	3.426	73.03116	Carboxylic acids	32146	77341	79484	59821	248995	65685	156732	227514	132383	76074	11984	238781	391342	120204	108437	379327
Propionylcarnitine	0.882	218.15411	Acyl carnitines	9634	16205	7378	108926	121193	108750	220968	155642	11927	13768	10417	48065	94623	77381	149758	126902
Propyl gallate	4.145	213.06445	Galloyl esters	51282	39899	38900	2741	1578	998	5826	2397	26460	31841	33088	4556	1918	1954	2193	2233
Propyl thiosulfate	3.111	154.98555	Sulfonic acids	13785	13259	5412	67178	38466	10329	107328	19380	5717	25354	4973	105543	92273	13773	143249	104741
Propylparaben	12.287	179.07193	p-Hydroxybenzoic acid alkyl esters	129	96	147	37946	8579	3245	8506	2810	127	166	59	33838	7366	3266	5986	11370
Protostemonine	1.02	418.22595	Stemoamide-type alkaloids	1547	1669	1227	98309	157148	141573	381829	363701	1542	1931	2449	51997	140071	174173	346122	107609
PRZ_M429	14.201	429.01703	NA	7148	16936	6877	5	21	108	12	25	42584	5273	8183	14	7	10	6	
Pseudocopsinine	3.286	339.20172	Aspidospermatan-type alkaloids	7784	14133	5992	161904	80463	867549	169019	55337	33877	11344	8299	41367	21227	613663	252967	123739
Pseudojervine	4.397	632.35834	Steroidal saponins	14379	6065	2909	1071	18922	102	2371	15478	5143	1606	1582	501	2899	1093	3588	2489
pseudolaric acid B	1.398	433.19174	Diterpene lactones	359	641	197	11019	22567	14052	16122	42611	113	2181	145	5876	46578	7466	10618	24805
P-Toluenesulfonic acid	2.98	171.01411	p-Methylbenzenesulfonates	2432	1599	1050	20060	27463	15877	33926	36106	1936	3747	2462	13258	33037	18039	63096	33537
purpactin A	3.306	413.16205	Diarylethers	29539	27089	28575	28422	31260	59644	105276	233879	36654	11251	5794	64042	48532	51078	87787	86959
Putative (3-hydroxyoctadecanoyl)glycine	3.101	358.29364	N-acyl-alpha amino acids	338804	467172	134826	1749	3535	6177	3407	2068	46469	110931	112252	4771	6851	2396	1333	6245
putative heterocyst glycolipid	2.784	576.49213	Fatty acyl glycosides of mono- and disaccharides	20716	13311	31614	64	190	21	41	129	2930	1532	2745	24	68	35	396	55
Puwainaphycin A	8.077	618.36523	Cyclic peptides	50	60	31	248825	491033	426467	2638	468454	38	6	9	501	507057	455118	548555	1633
Puwainaphycin C	6.857	614.32562	Macrolactams	69	129	58	6592	2362	4429	3791	1712	32	48	50	2112	18541	4630	2093	4612
Puwainaphycin D	10.665	613.3562	Macrolactams	66	49	68	437412	154146	2097314	272084	334958	62	56	132	442324	175846	1537264	246217	218379
Pyrafluefen-ethyl	13.954	413.02884	Phenylpyrazoles	67993	20059	49125	15	9	5	22	25	36192	41952	45918	14	21	76	30	29

PYRIDOXINE	5.043	170.0815		368	689	448	98207	182433	59605	117693	277814	754	328	220	442920	232540	25692	175460	106252
Pyrimethanil	1.185	200.10889	Aniline and substituted anilines	145	246	118	7262	8612	12047	13732	17853	141	233	260	2989	14921	16275	18253	17526
Pyroglutamic acid	10.024	128.03792	Alpha amino acids and derivatives	43	117	46	175381	71070	59134	140892	59315	49	54	146	25362	61402	40400	44694	107556
Pyrrolidine	11.657	72.081	Pyrrolidines	169	578	492	87653	148853	113243	145373	97838	199	279	103	237249	245449	121626	142050	77641
Pyrrolnitrin	14.112	254.97037	Phenylpyrroles	93	149	50	197251	212064	231202	86515	239888	789	37	32	160120	208295	300804	83432	199413
PYRROMYCIN	1.592	586.30585	Anthracyclines	245	173	3775	101776	18023	30695	19100	23320	9182	5321	642	328	4293	29910	24211	10433
Pyrroxanthin 5,8-furanoxide	3.089	612.34839	Terpene lactones	948	695	342	2544	1117	6400	5969	28633	719	315	475	1384	1532	4301	2230	2938
Pyruvate	4.422	87.01022	Alpha-keto acids and derivatives	2749	2011	715	37585	9836	587	47182	2403	1430	1781	2353	35627	7429	1042	293870	7329
Quebrachitol	5.578	193.01878	Cyclohexanols	125	27	66	28537	7574	2866	376	6967	6484	211	175	43894	13017	4578	14430	165
Quercetin	13.485	302.9696	Flavonols	25380	20679	16401	4	28	10	17	4	9601	34012	17903	85	53	146	127	52
Quercetin 3-O-malonylglucoside	14.113	548.90668	Flavonoid-3-O-glycosides		9	19	133339	113411	137765	30275	150823	22	12	31	81650	116946	195673	29606	98225
Quercetin Pentamethyl Ether	3.88	373.30356	7-O-methylated flavonoids	3942	1482	9773	885	9631	35399	23242	12396	1797	443	6818	5524	3931	28798	26099	17232
Quillaic acid	3.965	509.33878	Triterpenoids	135188	264566	174859	89963	71835	27885	47497	9033	101410	191801	350337	41642	10044	19188	25226	20065
quinine	13.815	325.17371	Cinchona alkaloids	4598	7852	7679	386091	323175	133414	227436	240211	8286	11172	5172	435730	255818	65280	212784	133649
quinine anhydre	1.443	347.16571	Cinchona alkaloids	3190	1056	24507	348	440	2987	871	423	126	5410	33707	704	256	3849	523	70
Quinolinic Acid	5.784	166.01907		329	247	102	803	328	155	60611	745	324	265	188	959	668	798	351	37855
Quinolone	1.917	146.05969	Hydroquinolones	213	79	80	160826	276161	81667	313838	4083928	243	239	184	4030	29967	239698	1477433	80822
R-3-Amino-5-(methylthio)pentanoic acid	8.67	162.058	Beta amino acids and derivatives		5	66	8096	24620	35835	15479	976098	19	3	8	4385	5218	61733	2688	10913
Raddeanin A	11.133	919.51593	Triterpenoids	3	18	10	126	163	39076	88	363	2	1427	1399	224	11	34595	49	54
Ramipril	6.34	415.2236	Dipeptides	5379	13788	8564	8400	4209	1603	2287	2566	6994	6226	14395	2860	5066	834	1897	3367
Ranolazine (Ranexa)	6.343	428.2489	Anisoles	100	289	139	140512	119389	396618	136051	174763	332	183	190	86501	78206	329377	127970	71384
Rebaudioside C	2.96	973.43744	Steviol glycosides		137	4848	11	16934	12186	29356	278	55	150	178	89	1432	5942	18666	34889
rescinnamine	5.754	633.32587	Yohimbine alkaloids	1349	304	1539	21827	27030	71293	288296	168479	133	1699	753	62298	7559	39192	6533	536194
Reserpic acid	3.808	399.18152	Corynanthean-type alkaloids	58108	39192	37580	348505	250812	165399	204193	230978	33951	34117	3297	396247	287730	110557	247453	185049
RESERPINE	4.116	607.45758	Yohimbine alkaloids	22795	15366	28183	2302	3606	8142	4385	1438	7020	162237	247371	3578	4563	8254	4605	1488
Resveratrol 4'-Methyl Ether	5.455	241.14847	Stilbenes	2260	323	484	57045	38348	19923	42701	43760	1313	1877	328	36256	16922	26699	66699	41708
rhein	8.726	283.08344	Anthracenecarboxylic acids	243	11494	20341	599	527	226	167	421	211	93535	3682	287	328	370	100	409
Rhodoxanthin	1.544	562.37842	Xanthophylls	1119	4796	14658	1635	1261	3898	2754	3051	3775	12221	1314	388	1480	3295	1645	38125
Ricinoleic acid	5.4	299.25546	Long-chain fatty acids	1340	3056	163	46985	33727	6162	4915	33433	309	677	1172	305	65244	3703	10245	22697
Rivastigmine tartrate (Exelon)	12.092	251.17494	Phenoxy compounds	60	637	71	1855416	1907583	631912	1317195	1177929	118	354	123	2130305	1686042	464035	484555	1028846
Roccellic acid	4.336	299.2244		53946	17291	35084	2066	1334	302	1171	330	25990	28783	40636	226	554	156	658	618
Rofecoxib	13.873	313.05173	Stilbenes	4437998	3760030	3543722	32	17	1605	406	64	4736707	3085171	3426365	56	26	54	46	33
Roquefortine C	5.448	388.17993	Pyrroloindoles	302	317	498	14707	47091	108665	1224	72370	466	212	158	3018	39728	50608	47975	11895
Roseotoxin A	5.201	608.3894	Cyclic depsipeptides	358	456	543	24683	28019	7027	11591	8296	396	264	280	4744	9109	5313	22387	15905
Rosuvastatin	5.898	480.16818	Phenylpyrimidines	53	236	39	143577	3010	1268	441	2018	642	69	24	306107	2194	2183	1590	1717
Rotundine	4.695	356.17612	Protoberberine alkaloids and derivatives	1080	371	463	4632	3469	51621	15775	7269	844	2025	2261	3587	2676	27371	14405	4046
Royal jelly acid	1.993	187.12187	Medium-chain hydroxy acids and derivatives	123089	30025	97283	1239	7045	10879	1649	55116	9677	10278	152265	507	4095	5347	28352	6674

Rubusoside	9.859	643.33051	Steviol glycosides	120	240	121	19708	19834	147502	25978	14084	159	216	200	25516	16306	198034	26364	11911
Rutaevin	6.217	487.20282	Steroid lactones	18	66	85	56114	13484	1382	9003	3855	99	23	22	3613	15339	1073	8244	9480
S-(2-Carboxypropyl)glutathione	11.099	392.10547	Peptides	1939	7558	5099	780	283	222	436	401	230	147	5	3623	816	241	300	675
S4:17(P3:15/F1:2)	0.877	654.328	Saccharolipids	13953	15196	16485	363	292	12	373	272	23340	23779	23294	48	354	405	304	255
S4:20(P3:18/F1:2)	4.034	723.32336	Saccharolipids	737	813	244	5523	3174	2979	1086	45665	1671	323	578	2327	3551	2373	7534	1988
Saccharin	1.454	181.99368	Benzothiazoles	348	206	678	89297	78835	15853	70236	12982	482	141	207	169214	81237	21038	82679	49983
S-Adenosyl-L-homocysteine	6.745	383.13104	5'-deoxy-5'-thionucleosides	21	63	13	31334	33812	420717	4997	29184	89	103	80	1532	528	78655	53765	597
Saikosaponin C	5.439	925.51324	Triterpene saponins	30	41	62	10501	7704	793	84	11298	30	77	64	768	35090	874	2219	20
Salazinic acid	14.598	387.02304		27	22	24	5022	31825	3172	72081	4984	27	31	21	90060	19306	441	25341	152793
Salicin	10.245	285.0943	Phenolic glycosides	59	84	106	69867	97816	32516	71430	48111	66	36	28	124291	109789	29541	73837	86205
Salicylic acid	4.553	137.02782	Salicylic acids	3082	4496	2400	19190	9032	8915	108403	943	4543	1601	1736	2102	7501	1369	26076	245790
Salmeterol	1.637	416.27173	Benzyl alcohols	1657	8063	3752	12435	24147	35810	27627	40767	845	4327	2222	1096	32929	31408	62856	25965
Salvianolic acid F	13.863	337.048	Stilbenes	35634	28054	15795	131	435	35	87	59	20439	7654	19427	79	92	89	107	21
Sanggenone D	1.458	731.20654	Linear diarylheptanoids	186	67	270	44338	23990	20068	28988	23946	218	250	89	46540	30999	16978	32034	26146
SAQUINAVIR MESYLATE	10.673	671.4046		7	19	14	21746	8130	190355	9406	14902	8	13	19	18177	6081	65539	5429	5238
Sarcinaxanthin monoglucoside	5.514	866.59637	Triterpenoids	17062	54	41	101	392		98	187	35	15	6	204	137		755	246
Sarcosine	12.344	90.05435	Alpha amino acids	363	131	251	9884	1017	1964	25000	329	376	110	199	2862	5118	15	7692	16015
Sarsasapogenin	5.231	439.32773	Triterpenoids	754	364	551	1767	1656	10530	51654	2065	95	420	214	1412	787	1025	9676	4309
Schisandrin A	1.592	417.21478	Hydrolyzable tannins	1524	463	2050	10652	32569	18665	18833	70092	1432	2578	2920	389	49107	21303	34269	30107
Schisandrone	7.201	357.17776	Aryltetralin lignans	62	14	44	137178	131149	79102	122171	160409	34	32	39	136881	104155	90836	153115	85265
Schisanhenol	1.021	403.20355	Hydrolyzable tannins	1017	673	1517	26651	28682	30394	66773	69477	711	1481	1607	10606	22520	35648	67435	23527
Schizantherin A	5.548	559.2113	Hydrolyzable tannins	15	13	30	336	515	151	16355	193	117	35	6	32745	1525	581	10733	366
Schweinfurthin F	1.64	479.28275	Xanthenes	646	541	330	2028	16296	32241	13326	15627	1096	945	439	1286	20038	64249	36669	62379
Sclerotiamide_130024	8.217	464.21585	2,2-dimethyl-1-benzopyrans	7	12	13	4985	7994	2985	2987	5868	11	5	19	1344	18379	1481	3288	5852
Scopularide H	3.595	700.46674		6110	15239	13750	46523	38940	8472	35601	43777	15825	9973	7472	1553	47809	12847	41137	37982
Scytophycin B	3.769	802.54718	Diterpene lactones	645	653	98	2114	2861	35726	2804	2316	332	191	1032	360	1792	30682	1732	1707
Scytophycin E	2.835	804.55688	Diterpene lactones	12061	22924	1973	1155	272	302	513	1000	1858	13339	2110	64	127	466	437	463
Sebacic acid	6.558	201.11372	Medium-chain fatty acids	78772	122810	185146	19249	17668	5533	15990	12967	216104	107739	90409	33193	15560	10964	13594	27846
Secoisolariciresinol	3.067	407.17197	Lignols	115983	105099	197468	27416	78974	56393	31495	743028	107499	121549	28634	213866	94784	57034	41374	35105
Secopenitrem D_120253	7.697	570.3526	Naphthopyrans	9	12	39	7979	18313	5433	152152	5828	20	5	11	1614	3629	11900	4564	5756
securinine	1.418	218.11794	Indolizidines	307	778	986	82006	179901	411608	236973	290660	727	891	438	94110	300744	424162	640199	272005
Senecionine	8.541	336.16949	Macrolides and analogues	18	16	20	1897564	2292408	2125179	2510324	2497311	7	6	17	22144	2353786	2481713	2793850	1938771
Seneciphyllin	5.734	334.16461	Alkaloids and derivatives	2301	4601	1081	3048	1677	1595	2872	6389	187352	3663	363	1514	4945	1172	2196	1911
Serine-Cholic Acid	5.975	478.31033	Glycinated bile acids and derivatives	183	1348	206	6919	14500	1274	3730	35225	166	129	533	3502	27645	2302	13283	2374
Sildenafil - Dark Web Drugs	3.183	473.19946		3552	14450	26205	7853	3111	32100	26221	13221	9025	9122	20383	23233	22104	13256	27496	10465
silybin	3.192	481.23929	Flavonolignans	327	4752	478	6959	15081	29118	15178	28905	533	679	22014	70958	14699	29249	26799	13675
Simvastatin M+Na	5.247	441.25931	Delta valerolactones	350	418	381	689758	444753	29230	211446	502667	754	427	493	80253	1645075	26161	190862	675734
sinapic acid	6.003	247.05615	Hydroxycinnamic acids	29	7	6	10582	1309	2587	13149	10062	18	47	75	163	241	4344	23547	23955

SkF 83566	14.003	330.03836	Benzazepines	517573	683028	401094	25	19	16	12	14	536169	373113	689903	13		25		
skimmianine	7.056	260.10287	Dihydrofuranoquinolines	35	14	32	162151	113067	53290	77694	75695	27	36	27	117148	57132	49944	108867	61467
skimmin	14.558	363.03448	Coumarin glycosides	242315	129022	95269	2	7	5	3	10	169646	107612	112907	11	8	14	6	7
Smenospongiarine	4.887	414.29907	Prenylquinones	3515	1919	3559	13188	6208	94124	15873	14663	1719	1347	2035	1263	7737	92817	11900	3547
Smenospongidine	1.102	310.30936		171193	172684	126599	39278	41459	36332	35962	31242	81760	57729	52289	8706	25761	29485	20056	27499
Smenospongine	11.451	268.99933	Prenylquinones	8542736	9583736	8951128	6	9	7	12	23	6687419	2631364	1611292	14	11	21	30	91
sn-Glycero-3-phosphocholine	13.822	258.10849	Glycerophosphocholines	144	173	86	156309	3800	1526	10151	6502	265	252	36	6612	2052	778	1504	6185
Sodium Deoxycholate	1.05	393.28247	Dihydroxy bile acids, alcohols and derivatives	178159	168781	287732	134064	134427	77855	150806	76481	304168	248247	361790	284816	138643	57377	89647	188710
Solanidine (not validated)	4.283	398.32343	Steroidal saponins	11797	13839	2968	295	415	1575	905	436	4447	839	6519	487	825	3841	1466	485
Solasodiene (not validated)	3.991	396.34698	Steroidal saponins	32521	46192	29386	1214	2205	2346	2663	23658	35193	11398	16631	147	995	2767	1679	1829
SOLASODINE	1.342	431.34665	Spirosolanes and derivatives	3389	3382	2196	45618	43363	51965	15078	2899	2527	7139	1148	7070	26847	42462	7718	13143
Solasonine	11.753	884.51459	Steroidal saponins	94319	109896	502933	16	25	962	40	26	83519	81325	57467	21	21	156	5	29
Solorinic acid	10.994	383.10031		41	84	95	16477	13187	40571	6814	19629	22	35	47	11121	4365	35332	4630	7131
sophocarpine	4.256	247.18019	Matrine alkaloids	533	668	104	24715	71623	135149	43999	45015	753	198	258	22895	20794	124780	25222	41685
Sophoridine	13.805	271.16354	Matrine alkaloids	6460	10705	775	10364	139442	70029	87106	114503	24132	641	208	9887	14987	33045	90735	20303
SPA-9C (TENTATIVE)	3.031	313.1124	NA	25281	166179	142340	1751	5084	6310	2572	2090	229652	18643	12190	574	1834	5722	30587	895
SPECTINOMYCIN	4.469	331.12341	1,4-dioxanes	1817	25630	6615	5872	12928	4209	3038	10661	3175	2508	2476	26198	4001	7522	7010	6500
SPIRAMYCIN	5.243	843.5249	Aminoglycosides	20	50	27	3449	462	82033	752	2450	25	46	61	173	481	50020	680	477
Spiroxamine	1.158	298.27124	Ketals	945	35817	21242	54993	46633	37966	42783	57725	38867	31250	35557	6986	33804	39963	27657	48542
Stearic acid	16.361	283.26471	Long-chain fatty acids	100726	134837	291763	39125	48074	11402	68248	92578	141768	38462	168521	81011	151610	106827	107461	83885
stepharine	6.364	298.14377	Proaporphines	4189	5255	1998	712	779	507	1066	1009	8743	3748	2015	13865	680	646	1658	926
Stercobilin	9.354	595.34558	Bilirubins	27	55	182	12279643	6628181	65456444	8824771	15246519	23	194	262	23585882	3331766	85673656	6658818	1607806
steviolbioside	10.204	641.32263	Steviol glycosides	57	206	46	5220	3690	88757	2593	847	121	64	222	6430	6625	30141	4307	1829
Stigmasterol	1.247	395.36844	Stigmastanes and derivatives	1263	805	308	5618	11263	9695	9301	13222	1541	376	463	353	2985	11984	5638	4190
Stigmasterol glucoside	1.107	575.42792	Stigmastanes and derivatives	11674	12515	3050	2480	143	113	2971	417	8867	5000	5243	175	1365	873	1494	1796
Strophanthidin	7.335	443.2662	Cardenolides and derivatives	34	102	32	2381	7689	3320	13565	4350	28	34	53	2575	3091	4348	7368	350951
Strychnofoline	5.594	483.27606	Harmala alkaloids	145	90	427	3659	2075	420	60014	1873	238	88	56	5734	1079	371	5288	39408
Strychnophylline	4.233	566.34528	Harmala alkaloids	21732	10776	3517	1346	1232	10129	2806	1911	43188	6532	6395	16072	6660	13493	9545	5102
Subsessiline	3.109	717.35107	Aspidospermatan-type alkaloids	60	177	72	260	10176	664	206	3851	194	72	85	279	18528	955	1170	7613
Sufentanil	8.202	387.20798	Anilides	8	11	6	51456	70538	57797	2845	86061	15	12	7	948	87876	84818	84226	4119
Sulfamethazine	14.009	279.08356	Aminobenzenesulfonamides	128	220	302	29157	142	184	363	215		300	141	158	142	59	69	441
sulochrin	3.186	331.12125	Benzophenones	1506	348	918	6412	24804	1986	2795	83035	1292	1082	234	14240	3613	6976	3568	1340
Syringomycin E	4.412	613.49554	Cyclic depsipeptides	14636	14026	32031	1505	640	635	758	865	3682	13008	27407	421	1680	742	2912	1442
Syrosingopine	3.134	711.29065	Alkaloids	2562	14009	8382	734	8611	3357	8905	11932	14903	767	516	1662	10678	7594	10213	8240
tabersonine	4.66	337.17664	Plumeran-type alkaloids	15334	13540	26129	26324	7822	12334	10067	29414	6641	20164	36851	3078	18965	22761	11403	28512
Talatisamine	3.087	422.28925	Aconitane-type diterpenoid alkaloids	17969	32479	32441	29437	9295	2763	4167	2472	86023	40231	48539	83374	31003	3223	8487	14277
Tanshinone IIA	8.892	317.11557	Tanshinones, isotanshinones, and derivatives	40118	60502	57328	607	2046	358	719	1755	21342	73431	58386	946	1102	513	1309	664

Taurine	10.985	124.00797	Organosulfonic acids	75	44	136	85277	4599	6864	297187	2818	51	39	20	35869	14008	1727	52417	493182
Taurocholic acid	5.658	514.28528	Trihydroxy bile acids, alcohols and derivatives	193	132	663	29551	35867	13043	388	18436	43	263	209	30043	56135	18527	22094	1185
taurohyodeoxycholic acid	4.608	498.29507		1320	1323	2842	43012	43701	56703	21711	48803	3475	2293	1650	50958	52236	28002	15174	18375
taurolithocholic acid	3.697	482.29047		4987	1316	542	1302	3723	13189	34215	2191	3591	3755	637	5338	5963	10852	9008	49370
Tebufenozide	2.903	351.19934	Benzoic acids and derivatives	220751	525557	56842	302	70654	106249	262809	93009	8793	93624	14229	29972	69128	16152	235	133478
Teflubenzuron	13.769	380.98853	Dichlorobenzenes	15086	23816	12504	48	115	137	22	40	16281	13444	12293	31	80	93	77	31
Telocinobufagin	0.939	403.2363	Bufanolides and derivatives	24055	37117	37957	590589	623654	307319	691012	660009	41902	32844	26384	180062	667362	253400	486678	590870
Teraspiridole C_130091	6.547	565.32959	Naphthofurans	288	530	200	55960	23186	73445	15337	47174	375	252	135	42081	14900	21395	23224	12820
Terbutryn	8.482	242.1525	Methylthio-s-triazines	7	7	15	21545	19828	16010	10359	26919	22	13	9	780	24593	17640	27400	2791
Terfenadine	3.12	472.33835	Diphenylmethanes	31348	17817	53760	50776	43368	26525	25094	30531	33057	62300	36189	8462	51209	33684	23148	26785
terpestacin	10.87	420.3053	Secondary alcohols	20	72	49	207	406	57125	11433	2264	24	23	23	305	398	17929	1815	2027
terracinolide C	8.995	761.29718	Delta valerolactones	4	7	1	195	44	95748	1245	404	36	9	10	313	21	83071	842	144
testosterone	4.135	289.23764	Androgens and derivatives	43038	31079	43956	509	928	133	1619	419	13751	17435	45807	579	303	1095	847	273
Tetraglyme	1.529	223.15373	Dialkyl ethers	57551	39047	63812	113	1096	286	1336	265	28562	54429	49624	259	334	2713	958	741
Tetrahydroalstonine	3.339	397.17566	Alkaloids	23321	25480	18671	44948	69044	40440	103334	130354	47779	22277	9215	66542	62573	19539	24444	41052
Tetrahydrocurcumin	3.132	373.1496	Curcuminoids	786	5008	4209	18507	5022	1796	3298	1060	379	1154	739	5853	2364	1435	4626	16768
Tetrahydrogambogic Acid	5.749	631.32794	Pyranoxanthones	116	261	273	76644	52338	206690	1056484	449267	90	94	229	87336	193207	144981	14831	1998367
Tetrahydrospirilloxanthin	4.151	600.47858	Xanthophylls	13158	7024	24514	786	447	1987	2828	1003	850	33208	49837	801	833	4677	507	1408
Tetrasaccharides (Hex-Hex-Hex-Hex)	14.4	665.22125	Sugars	110	54	68	364	4907	3212	5415	4414	7684	603	83	1885	4944	1468	577	6458
Thalsimine	12.205	637.28339	Tannins	52	71	91	92400	97290	65690	76962	125711		53	108	138810	62680	64024	86375	69164
Thelephoric acid	14.037	353.02603		159744	194198	182501	54	20	11	22	20	142478	207387	190193		16	14		17
Thermopsine	12.797	245.14972	Anagyrine-type alkaloids	189	79		17221	666	6572	68874	1594	53	166	122	565	1815	741	13139	157487
Thiamine	13.985	265.10995	Thiamines	106	83	98	13291	52142	41744	64441	109509	436		105	50424	53940	29300	66597	27002
Thiamphenicol (Thiophenicol)	13.823	356.00967	Benzenesulfonyl compounds	27875	32262	29197	28	83	138	25	32	40387	33994	14775	32	125	44	84	159
Thiophanic acid	14.023	392.88202	Xanthones		139	24	57649	60590	71059	61039	57496		150	113	49899	54286	70422	51374	70365
Threonine	9.809	120.08001	L-alpha-amino acids	198	15	44	99454	44628	41333	60509	26831	121	89	135	46767	27340	49535	35738	31109
threonine conjugated cholic acid	7.783	510.35355		8	36	29	71128	25853	31823	23025	69621	24	9	22	926	113114	37053	13528	75687
Thuringione	11.4	374.96228		169980	152610	188188	11	4	8		10	68838	78594	23727	7	7	5		7
Thymol	0.984	149.09962	Aromatic monoterpenoids	4177	1768	3139	208	233	264	409	158	1357	4652	7975	241	184	263	370	409
Thymol-beta-D-glucoside	1.121	311.24808	Terpene glycosides	63416	66865	42986	90879	88977	91604	80368	78479	96114	98746	94932	65334	71272	105844	94946	80398
tigogenin	1.608	417.33588	Triterpenoids	10408	7559	4927	7059	3482	80876	26604	82421	12761	5549	6086	165	9248	68257	53055	10356
Timosaponin B II	4.14	921.52563	Steroidal saponins	19	24	88	641	592	6547	1624	3173	653	11	583	2742	374	23263	2213	950
TOBRAMYCIN	5.541	485.28662	4,6-disubstituted 2-deoxystreptamines	235	492	595	10554	2875	704	155564	2417	214	113	266	26897	4045	156	4674	280542
Toddalolactone	10.195	331.11426	Coumarins and derivatives	34	6	30	146929	72109	23842	58068	32960	31	18	11	76138	68106	20560	51682	68515
tomatidine	10.075	416.33441	Spirosolanes and derivatives	46	48	138	76228	84950	32441	18670	64281	57	43	42	111542	50782	27199	44622	10540
Tomatidine HCl	3.312	452.33231	Spirosolanes and derivatives	6515	3764	11749	6023	15231	139123	58130	22012	9961	8114	6093	2222	21385	94909	27244	23643
Toosendanin	5.576	597.23346	Limonoids	32	53	16	5965	459	28991	654	15713	69	70	77	6737	1594	37218	5499	211

Torasemide	4.385	347.11823	Pyridinesulfonamides	1203	1688	2355	57411	41307	3635	6991	163811	1934	939	1224	20942	39096	6211	9007	15619
Tormentic acid	1.66	489.35324	Triterpenoids	2769	5470	2895	19089	2557	23648	8808	3360	2277	2532	4080	359115	1777	7314	12032	7937
trans-4-Coumaric acid	4.85	163.039	Hydroxycinnamic acids	8859	79041	5966	2196	1021	978	2254	714	4160	56920	7281	3410	1176	414	1729	793
trans-4-Hydroxy-3-methoxycinnamate	4.311	193.05466	Hydroxycinnamic acids	2300	833	1660	41027	17237	2996	30598	8398	4840	1597	1229	22125	8927	1333	63888	7398
trans-4-Hydroxy-L-proline	0.946	130.0661	Proline and derivatives	2155	2045	3105	684	348	11527	3585	5753	1194	3368	15889	1181	737	1610	2696	523
trans-Caffeic acid	4.639	179.03725	Hydroxycinnamic acids	52160	55075	56858	455	264	1295	1096	618	52545	47963	72040	2355	1086	871	1491	320
TRANS-CINNAMALDEHYDE	7.055	133.07162	Cinnamaldehydes	59	23	59783	179	57	301	32	320	38	44527	27	88	116	154	32	238
Trans-Vaccenic acid	2.863	281.24896	Long-chain fatty acids	2406384	1699640	1573201	28727	43239	55813	37194	45461	2866153	2167212	1120054	15722	27074	70911	39157	29553
Trehalose	13.904	341.10901		547	106	263	58779	4778	5103	13111	17715	114	509	118	83162	67824	1800	5827	91526
triacanthine	12.684	226.10724	6-aminopurines	76	55	127	48748	77536	100388	66995	43735	96		74	138118	102340	21881	21555	26033
Triacetyl resveratrol	5.733	353.10556	Stilbene glycosides	297	2403	697	6237	17420	5032	2474	4296	31358	914	154	20284	6896	4371	3248	4320
Tridecanoic acid	2.994	213.18727	Long-chain fatty acids	30264	21943	33756	6291	7616	8384	3869	6125	25913	49134	65159	3440	4557	7134	2929	1308
TRIDESACETOXYKHIVORIN	3.343	483.30722	Limonoids	1754	3346	6917	8230	9493	5267	9660	10445	2427	3135	144330	16539	3906	27058	11794	7362
Triethyl citrate	0.932	277.1286	Tricarboxylic acids and derivatives	14212	20665	19753	877	502	606	1034	723	10113	27506	25427	1759	935	626	782	745
Triethylphosphate	1.009	183.07866	Trialkyl phosphates	16982	12667	14324	959	638	2880	1206	1443	6843	6567	19481	2290	1046	1369	2180	2428
Trifluoroacetic acid	1.485	112.98881	PFSA	1459	13699	29677	165099	114755	60807	125233	114855	46975	636082	1204503	86819	84242	55332	65728	94573
Trigonelline	10.599	138.05363	Alkaloids and derivatives	787	607	1695	59785	6986	12071	683278	9058	237	1891	282	28226	4992	451	84601	204202
Tri-isobutyl phosphate	0.917	267.1756	Trialkyl phosphates	7788	6333	11825	395	501	457	634	237	3827	8995	14243	727	315	493	584	314
Trimethylamine N-oxide	12.174	76.07397	Trialkyl amine oxides	269	238	206	87298	8883	167	21654	136	187	194	74	61968	906	14	3594	12991
Trimipramine	5.642	295.22223	Dibenzazepines	6084	6557	15097	5217	1542	522	4534	4003	30892	1949	4714	1532	5414	376	3975	2977
Trinexapac	12.303	223.06198	Vinylogous acids	997	516	859	19949	4623	2145	4165	2162	249	1049	413	17625	4232	612	1130	7254
Trinitrotoluene	6.223	227.02635	Nitrobenzenes	43	76	15	53016	48711	12220	22639	40481	116	83	30	173273	55769	20139	80718	66410
Triptophenolide	1.475	311.16998	Oxosteroids	37727	34554	31059	55297	32719	37504	32167	27722	71513	96936	139846	28108	35837	18870	28096	40065
Tropinone	1.437	140.10648	Tropane alkaloids	4820	9468	11914	8288	13562	55333	27475	19333	33473	9217	4417	144632	16860	43118	26818	25800
Tropisetron	6.692	283.15427	Indolecarboxylic acids and derivatives	258	704	311	25963	36713	12600	32916	54522	892	351	300	66611	34947	20585	37808	29326
Tryptophan	13.273	203.09485	Indolyl carboxylic acids and derivatives	59	71	124	19169	11533	7837	11681	5932	62	100	36	24031	5492	6370	9235	7874
Tsugaric acid A	4.206	521.35254	Triterpenoids	13863	50726	54011	77659	21662	8808	36557	15449	6968	27816	26362	71889	18954	3732	18788	30962
Tuberostemonine	7.185	376.22229	Stichoneurine-type alkaloids	81	75	84	1113	1871	1429	2246	1988	132	40	39	2393	1623	608	1145	42719
Tyr	11.39	180.06903	Tyrosine and derivatives	79	275	218	41764	11235	44578	42359	21064	299	104	76	19324	30551	43518	33285	32157
Tyrosine	11.378	182.08159	Tyrosine and derivatives	181		164	95442	41780	38598	76750	44551	184	128	55	78835	47548	80723	41889	32887
Ubenimex (Bestatin)	8.702	309.18097	Hybrid peptides	31	56	110	49221	16106	162435	23943	21181	22	67	74	120449	9929	155935	26464	26920
Ubiquinone 30	3.051	597.46375	Ubiquinones	25418	9068	34856	28858	5542	5964	6541	2923	6307	51019	51839	636	26122	2230	7682	5258
ugaferin	5.392	449.26108	Germacrane sesquiterpenoids	668	864	917	75594	55935	24353	4056	64622	777	530	560	9348	70432	16449	35757	5951
Uracil	6.184	111.02193	Pyrimidones	29	5	9	12177	10354	6945	26420	17830	66	3	49	1953	15090	1927	1270	46399
URATE	6.49	167.01497	Xanthines	55	37	66	3519	738	1072	52954	564	12	27	54	2220	735	422	540	27910
Urea	4.521	61.03981	Ureas	92833	149885	706197	844	151	144	1155	314	61033	457170	165438	7207	792	133	356	782
Ureidoglutaric acid	8.754	189.04326	Glutamic acid and derivatives	910	9476	6884	71	43	143	51	178	1360	13995	7591	157	137	50	95	79
Uric acid	11.107	167.02357	Xanthines	277	215	168	17617	16053	9592	71819	11393	87	189	133	7943	22164	9585	13933	78997

Uridine	6.258	243.06412	Pyrimidine nucleosides	215	198	988	64274	14497	39931	67281	48186	737	161	46	19954	189061	82851	38940	54965
Urobilinogen	3.933	593.33282		560	1218	2051	1366591	1012308	27756534	4245239	587325	464	481	1542	1841311	723398	17534272	2987316	2206134
UROCANATE	7.614	137.0359	Imidazolyl carboxylic acids and derivatives	8	7	3	19820	7990	6837	8137	9026	23	10	14	3842	6428	6701	9583	9237
UROCANIC ACID	7.592	139.05045	Imidazolyl carboxylic acids and derivatives	19	10	49	89910	31853	25440	32427	39383	31	15	19	19383	17598	31578	31338	36890
Ursinoic Acid	13.804	299.00681	2,2-dimethyl-1-benzopyrans	6768	2735	5399	52	131	84	123	247	10690	10233	8309	65	104	158	447	207
Ursocholanic Acid	4.115	359.2822	Bile acids, alcohols and derivatives	154005	300034	123761	583	12931	4857	9271	3370	240849	128982	128414	2096	16155	5839	12123	17419
URSODIOL	3.796	391.28143	Dihydroxy bile acids, alcohols and derivatives	37638	14376	12121	1884	16561	252911	76593	36418	20123	29790	32226	8032	5181	280964	92038	42202
Ursolic acid	3.533	455.32159		13270	102948	32559	1749	1982	2495	9273	583	4187	8471	4771	11797	12767	1562	4152	4912
Ursonic acid	3.227	477.34122	Triterpenoids	5933	3156	11941	10537	23497	12178	28654	15944	2835	12343	39254	6592	18013	8109	16172	14974
Uvarigrin	0.988	609.50391	Annonaceous acetogenins	94278	108975	63170	35141	30259	7834	25905	9565	36597	37264	49513	7271	19867	6155	8807	47732
Valaciclovir	8.877	323.15918	Alpha amino acid esters	1582	2873	2337	45429	27280	701464	40622	14948	1339	401	1215	129027	25379	401598	53544	24816
Valsartan	1.182	436.23608	Valine and derivatives	367	426	460	19797	27414	22192	25333	37777	403	527	163	5786	20420	16686	16582	18483
Veraguensin	3.22	395.19598	7,7'-epoxylignans	1213	1817	783	50966	56644	11784	99276	35922	12024	418	760	126576	35788	28791	127057	95009
Verapamil	5.186	455.27478	Phenylbutylamines	669	1114	478	1282689	940429	95123	646197	1373079	1116	637	673	119872	3206776	87109	604448	2173330
veratramine	4.29	410.3284	Fluorenes	4166	764	9746	991	4646	543338	80020	34840	4441	547	1190	1118	6641	540188	72885	43668
Veratrosine	0.91	594.33234	Fluorenes	543	916	94	7074	5879	11064	16057	10668	318	228	1	753	17154	8198	16772	6403
Vercuronium	4.233	557.44592	Steroid esters	6220	3236	17528	552	605	637	115	728	1126	16675	18180	1111	1127	524	673	1990
Villosinol	3.279	425.22754	Rotenones	14301	61643	89151	3569	2292	1467	4388	2362	4731	26989	40904	3186	2644	3336	3692	2595
vincamine	13.816	355.19501	Eburnan-type alkaloids	4044	7456	4881	76905	62934	22851	44482	37763	6717	14276	5207	88260	54176	12794	44649	31280
Vincanidine	1.479	309.15939	Strychnos alkaloids	191	97	239	11883	18456	102047	168226	43507	263	232	293	6198	17296	213073	125262	30642
Vinpocetine	4.811	351.21362	Eburnan-type alkaloids	56986	260622	159523	16274	12706	17025	8876	5521	119113	126040	139181	27784	6927	6150	26738	16529
vitamine a acetate	0.981	329.26846	Retinoids	17923	79597	21776	8492	10616	4735	14919	7980	39596	21206	42030	9126	5750	2223	10975	15022
Voacamine	3.785	727.39319	Ibogan-type alkaloids	488	330	512	1309	14767	2419	12725	47478	113	288	229	2322	1636	1017	10939	3039
Vulpinic Acid	4.27	321.08875	Butenolides	1025	1734	1214	11352	62283	1899	4421	42682	2531	2419	2149	5523	4385	3865	4965	4458
Wilforgine	3.255	880.2876	Terpene lactones	10331	11908	716	95	996	1355	1759	887	180699	21394	1473	1755	1371	5669	537	2670
Withanone	5.398	471.26926	Withanolides and derivatives	418	4089	2152	468943	319163	61637	6962	394118	2583	2738	3534	3103	882202	44490	121326	53105
Xanthine	7.123	153.03973	Xanthines	7	884	52	16084	18300	3555	41370	6705	34	69	55	6640	12419	8635	12820	65499
Xanthurenic Acid	8.777	204.03203	Quinoline carboxylic acids	353	621	573	544	5123	1032	994	8203	11233	709	22	3983	4038	479	1820	1421
Xylose	7.728	149.04744	Pentoses	11	7	7	75346	28173	6337	24927	6351		17	9	174449	15587	9572	128367	7069
YOHIMBIC ACID HYDRATE	1.465	339.20206	Corynanthean-type alkaloids	36249	31778	31075	143763	107800	79190	119673	89139	90774	81818	117060	72115	93431	64684	86987	96311
Zapotinin	3.745	327.08777	6-O-methylated flavonoids	103828	205926	28692	333548	360914	198487	171954	171323	80815	149979	24989	299280	317912	109234	242806	587638
zearalenol	6.425	319.16635		2893	5002	2642	340112	311420	1798508	362671	246823	3917	2151	3463	280200	213357	1525020	414845	196157
Zearalenone	6.59	317.14514	Zearalenones	80	301	252	11321	17039	48060	23658	24873	685	162	143	10389	14356	47114	25725	22031
Zeatin	6.595	220.10341		74	8	63	21449	39089	18524	22583	57618	77	33	49	19243	32834	18812	26519	36311
Ziyuglycoside II	6.513	627.38745	Triterpenoids	178	38	111	12745	12072	70872	14176	9551	73	34	72	43535	2711	17458	8546	12645

Table S4: Complete list of blood metabolites for *Laiwu pigs* and *Lulai pigs*.

Metabolite name	RT(min)	m/z	Ontology	LU-1	LU-2	LU-3	LU-4	LU-5	LU-6	LU-7	LU-8	LW-1	LW-2	LW-3	LW-4	LW-5	LW-6	LW-7	LW-8
(-)-Shikimic acid	10.373	173.05794	Shikimic acids and derivatves	26566	16792	14655	29990	66923	14619	40313	20096	19433	10927	67766	17930	15544	8398	22037	10944
(+)-Dihydrokavain	13.522	231.09502	Kavalactones	4647	9043	4559	4942	4891	7045	5813	6077	2891	20954	4481	4560	5008	4825	5967	16734
(2R)-6-methylpiperidine-2-carboxylic acid	9.842	144.10159	L-alpha-amino acids	29718	44671	11894	14732	7951	17533	13251	6791	20013	24591	22373	252289	23781	9199	16893	1043
(2Z)-3,7-dimethyl-2,6-octadienyl acetate	12.525	219.13123	Fatty alcohol esters	17726	15011	12983	4637	3153	21053	18994	4379	37144	4602	3255	5095	5971	1685	14976	3326
(S)-1-carbamoylpyrrolidine-2-carboxylic acid	13.854	159.07619	Proline and derivatives	68370	27455	32780	33170	33519	45899	34644	24281	47164	26362	28387	22301	39916	29212	22636	27614
(S)-3-Amino-5-methylhexanoic acid	9.18	144.10524	Beta amino acids and derivatives	12141	30979	23732	11345	14541	14564	24781	19521	12171	19534	26183	23192	17787	13223	11421	3759
1',2'-dihydro-2',6-dihydroxyrotenone	12.733	467.11246	Rotenones	1292	626	2414	16817	1978	4475	1254	3165	15201	869	1959	1080	8515	490	1442	3468
1,4-Butynediol	13.241	85.03127	Primary alcohols	15259	16721	19924	14029	14277	17411	20675	14454	11063	19095	18500	10627	13825	16435	11917	6733
1,4-naphthalene-dione	1.338	158.04784	Naphthoquinones	16207	17710	17721	21726	16059	5905	12698	19473	10456	27173	14084	19641	14160	28592	35928	21417
12-Cytisineacetamide	12.065	248.14975	Cytisine and derivatives	9748	7201	14700	12896	8705	15672	10464	13241	23659	14148	12226	8030	12627	21414	10354	9056
13-alpha-(21)-Epoxyeurycomanone	13.679	447.11639	Quassinoids	49218	7340	7316	44520	42789	6768	39823	35507	43156	8047	8108	5840	43816	7861	35187	28482
13-HpOTrE(r)	1.277	309.20471	Lineolic acids and derivatives	7846	6905	7151	23709	7342	6078	16487	11433	7577	13141	8024	5691	5147	2832	8036	3608
14-deoxy-11,12-dihydroandrographolide	1.089	333.20856	Diterpene lactones	24132	53161	24878	27772	19048	86208	22770	31325	21265	17427	13943	37987	23492	51002	35532	44694
14-Deoxyandrographolide	1.039	335.22733	Diterpene lactones	13514	27965	12303	16915	8979	45187	24722	20979	14932	6326	8000	27027	31853	38423	17895	83247
19S-Methoxytubotaiwine	1.074	353.19495	Strychnos alkaloids	6939	7287	6620	7522	16512	2747	11715	3569	8701	5491	8656	9755	5669	3859	9604	2847
1-Aminocyclopropane-1-carboxylic acid	13.585	102.05318	Alpha amino acids	32162	37190	30065	33200	30320	32196	38099	35665	35834	46043	38497	34944	27316	34663	36251	15801
1-desoxymethylsphinganine	1.414	284.29428	1,2-aminoalcohols	132043	66141	55800	159013	106915	100899	74941	108660	107806	72726	221731	78493	55455	103504	75355	44852
1-Hydroxy-2-naphthoic acid	7.958	187.04327	Naphthalenecarboxylic acids	27344	8062	26026	6988	60584	83104	29383	55736	5686	10907	9431	5124	12319	7521	23362	42640
1-Hydroxyanthraquinone	11.456	224.05632	Anthraquinones	36370	39162	40284	50009	60689	29181	67704	43916	40543	31955	62814	39496	29356	34754	42222	20515
1-hydroxypyrene	7.299	217.07289	Pyrenes	51740	17971	3734	25418	29490	15776	15031	17836	22050	2256	23802	11143	17921	35327	17451	9779
1-METHYLADENOSINE	12.946	280.10504	Purine nucleosides	73662	25541	17187	24202	32353	59981	18725	15613	20520	8713	37529	10462	15867	18941	11046	27151
1-Methylnicotinamide	13.527	137.07176	Nicotinamides	25577	32083	42429	17690	17069	29934	25293	18426	26178	26561	23139	26594	23986	24678	7747	16621
1-O-b-D-glucopyranosyl sinapate	11.173	385.0896	Hydroxycinnamic acid glycosides	7690	5667	14562	4334	13384	13732	14792	6609	9487	4209	10458	6419	4712	5316	7090	522
2-(Perfluorotetradecyl)ethan-1-ol	14.926	762.97235	PFSA	24815	29854	15797	19994	27307	29994	35824	24263	18494	38891	31551	35202	34705	30001	35431	39845
2,2`,4,4`-Tetrahydroxybenzophenone	1.089	245.04886	Benzophenones	44408	114412	89811	27068	21109	262810	95575	489923	56166	11732	234948	84773	285374	254632	186916	335101
2',2'-Difluoro-2'-deoxyuridine	4.9	263.04593	Pyrimidine 2'-deoxyribonucleosides	150223	114536	63116	91739	43941	69812	132645	25872	97671	170038	67587	181290	174637	79289	146264	14932
2,3-dihydromicrocolin A	1.346	772.63684		5404	5755	678	1645	8343	776	5725	4315	4495	3066	8063	2404	817	5939	2350	1534
2,3-Dihydroxybiphenyl	7.265	185.05823	Biphenyls and derivatives	61117	80430	70	56425	45600	1060	50982	60035	98971	52823	31810	90595	35792	83739	70112	408
2',4',6'-Trihydroxydihydrochalcone	9.797	257.08051		31034	39611	15580	41411	14550	28138	21609	16123	41801	38362	62652	28341	12537	29565	85746	91583
2,4-Diaminobenzenesulfonic acid	0.718	187.00627	NA	1378149	1365240	1332639	722827	916917	1745051	667445	457677	461880	652132	928237	468783	279827	451422	458211	333585
2',4-Dihydroxychalcone	10.733	239.08012	2'-Hydroxychalcones	364143	269156	184051	252591	211420	403492	43187	58484	34809	473366	113820	255437	179341	155289	44783	300342
2,5-Dichlorolichexanthone	13.487	355.00967		311953	291007	275220	410521	449248	374902	410504	349700	415224	324943	525219	495026	341335	488039	577013	201842
2,6-di-tert-butyl-4-methylphenol	0.978	219.17924	Phenylpropanes	113179	174617	71869	205954	168207	98160	189433	100212	144144	225722	149309	178055	173340	162876	148973	96222
2-amino-10-methanesulfinyldecanoic acid	3.03	250.14304	Glucosinolate breakdown metabolites	4854	5002	14950	3422	2339	106605	11347	37184	3519	3563	14778	15183	17456	19935	15071	18911
2-Aminoadipic acid	13.744	160.06413	L-alpha-amino acids	20265	28196	25949	22884	22116	15812	18849	6865	21828	29117	13987	31721	5095	22222	10478	78665
2-Chloro-6-O-methylnorlichexanthone	14.799	305.02277		4863753	3511925	2782554	4329743	3553820	4810314	3857614	4914379	4366734	5745249	3285118	4879427	4776110	2901581	4879790	5389879

2'-Deoxycytidine 5'-diphosphate	13.567	386.03485	Organic pyrophosphates	49633	53747	23740	63571	36292	49674	29108	31750	54616	28829	10952	44818	78409	58701	29149	14561
2-Deoxyribose 5'-phosphate	6.721	213.0174	Pentose phosphates	13869	43932	52662	19089	59490	38663	40569	21228	42758	32445	62226	48006	71909	57882	23624	16925
2-Hydroxy-4-methylpentanoate	1.321	131.07265	Hydroxy fatty acids	27112	15327	21426	25608	31233	30098	23858	14401	16940	22078	22061	18901	18085	18715	15418	30065
2-Hydroxyisocaproic acid	13.863	131.08453	Hydroxy fatty acids	91113	53513	71510	58516	50789	80051	82905	44700	60759	76616	90246	49664	71322	33902	48063	37273
2-methyl-4-(pyridin-2-yl)but-3-yn-2-ol	10.958	184.06985	Pyridines and derivatives	17157	20026	9626	25762	36535	20049	25993	21731	22907	21093	38714	21414	15989	10850	25097	9504
2-Methylpyrrolidine	9.811	86.09554	Pyrrolidines	495717	437323	416173	449988	582225	537362	557972	462699	506538	680147	708104	600560	627316	487404	441780	412968
2'-O-Methyl-5-methyluridine	13.459	271.09088	Pyrimidine nucleosides	5579	2495	1013	1385	2035	1242	1249	2499	2211	309	876	991	2908	7610	2179	11364
2-Oxobutyric acid	12.123	101.02486	Short-chain keto acids and derivatives	45808	57331	34010	66013	27449	31781	72077	65543	26659	117004	79512	54421	21797	30780	68803	102126
2-Oxoglutarate	8.304	145.01376	Gamma-keto acids and derivatives	178105	156019	55280	235808	61379	68977	144033	163861	97807	7366	4519	52954	46633	815	302299	590700
2-Phenylacetamide	11.238	136.07523	Phenylacetamides	61622	63125	77625	48035	60786	87250	67802	33043	69970	63691	39920	64767	61165	45308	49130	59181
2-Piperidone	3.124	100.07643	Piperidinones	7851	2706	4872	151130	11711	3402	8650	7620	4308	11359	6657	3943	4760	3823	7194	4039
3,3'-Di-O-methylellagic acid	13.714	353.02045	Hydrolyzable tannins	162928	385332	696118	318305	254984	434787	621582	447537	279000	599207	603196	631613	434909	384540	549533	79176
3,4-Dihydroxymandelic acid	12.227	183.0401	Catechols	10741	12522	17301	11693	14445	38126	31552	10883	25579	3969	13260	13602	7409	7781	14500	5095
3,4-Dihydroxyphenylglycol	7.282	169.06186	Catechols	8538	3703	81	13500	23999	769	33164	8563	9250	1987	50249	4753	3563	2022	6312	395
3,5-dihydroxydecanoic acid	6.695	227.12439	Medium-chain hydroxy acids and derivatives	51369	72128	26591	33581	30059	49966	62518	23734	40201	31892	124577	57654	65167	17512	44000	61682
33FTA	10.993	241.01154	Medium-chain fatty acids	65594	3295	11478	5115	50725	70282	10993	20789	17945	1867	8604	3567	31341	30488	28158	4611
3-acetylthiazolidine-4-carboxylic acid	7.321	176.03693	Alpha amino acids and derivatives	128	27057	12221	13118	2259	1543	8627	16823	1104	77602	2600	35151	17874	38422	24742	345
3-Chlorotyrosine	11.926	214.03824	Tyrosine and derivatives	21138	14485	7741	10007	4448	6850	2911	11467	17798	1725	4283	874	12416	88644	14201	73536
3-Deoxycaryoptinol	1.052	433.22778	Furofurans	2784	8880	1984	7732	4449	14237	7982	1929	11011	18878	2889	12045	6024	9852	6860	18439
3-deoxysappanchalcone	1.044	269.01312	Cinnamylphenols	13082	6238	9166	11032	6632	7509	15313	4126	7534	13614	11598	10655	6613	6030	6164	561
3-Hexen-1-ol O-b-D-glucopyranoside	13.108	263.14639	Fatty acyl glycosides of mono- and disaccharides	96939	135046	134448	116232	109256	117024	165354	79459	125453	134196	101702	109467	83626	87762	84307	80875
3-hydroxy-1,2-dimethylpyridin-4(1H)-one	10.048	140.06725	Methylpyridines	110710	107356	114949	109900	83805	108227	79345	109730	102093	157527	202139	121490	143422	84446	108992	88647
3-Hydroxy-3-methylglutaric acid	11.443	161.04718	Hydroxy fatty acids	46365	40661	36770	28251	23661	44061	47185	23626	27195	25241	38268	25214	26477	35029	21863	43840
3-Hydroxyanthranilic acid	13.108	154.05818	Hydroxybenzoic acid derivatives	37546	15025	46205	36620	39267	46832	51996	12154	50395	23595	46258	18615	38068	80111	11652	43988
3-Hydroxycinnamic acid	11.251	163.04393	Hydroxycinnamic acids	5147	6667	16980	4502	10300	21235	11268	10891	7780	10644	6085	16444	10109	7524	8181	3934
3-Hydroxysebacic acid	1.314	217.11357	Medium-chain hydroxy acids and derivatives	19182	10563	19022	29050	22441	15956	23476	17085	14762	18048	21513	21720	19295	15954	21298	649
3-Indoxyl sulfate	1.233	212.00266	Arylsulfates	252290	104021	228518	38148	203117	349568	61943	147938	178783	109356	78348	181323	220835	137182	107760	75965
4-(1H-indol-3-yl)butan-2-one	1.002	188.10678	3-alkylindoles	6315	21942	5099	4535	2771	19669	10174	2593	2985	5303	1748	3413	2929	5254	2808	5515
4-(sec-butoxy)benzoic acid	13.433	217.08052	Benzoic acids	6719	9138	12102	8324	9401	26232	17092	14082	13735	7228	14734	48094	13681	14586	39231	8231
4',5,7-trihydroxy-3,6-dimethoxyflavone	15.003	328.98636	6-O-methylated flavonoids	147864	138953	133955	129768	151330	125197	67200	140348	73675	127560	132484	139560	144734	55595	139561	155171
4,5-dihydroxy-3-propylcyclopent-2-en-1-one	7.281	155.04622	1,2-diols	51489	66939	55288	54723	94981	40561	114009	50977	58596	23960	124643	76671	22906	22473	32482	47
4,7,8-trimethoxy-3,5-dimethylchromen-2-one	11.026	263.06519	Coumarins and derivatives	12298	6100	7132	9947	23873	21651	12266	1510	12015	17243	6459	11102	9453	5665	17195	4925
4:2 Fluorotelomer alcohol	14.687	263.01318	PFSA	188063	205751	179798	158155	171458	147187	188347	160935	151305	180141	148023	163082	183942	215448	193970	183549
4-ACETAMIDOBUTANOATE	7.185	144.06891	Gamma amino acids and derivatives	25222	12539	8479	27447	39423	13497	16244	13633	20714	11595	16139	16543	8206	7501	15144	8787
4-aminovaleric acid betaine	3.081	160.13332	NA	29846	21659	15853	77784	47513	10150	40447	17771	20565	13481	48221	21351	17664	7848	13108	7048
4-Hexylresorcinol	1.281	195.12421	Resorcinols	7680	6392	8677	33449	32419	12792	9440	5561	26303	18172	6181	16049	20178	24839	83192	60356
4-HYDROXY-L-PROLINE	12.837	130.05205	Proline and derivatives	160821	133818	136234	157562	101243	206394	354259	119371	186517	107256	258367	75107	168110	193253	157883	106706
4-Hydroxyquinoline	1.45	146.06113	Hydroquinolones	103078	190937	141212	121235	72094	178148	115500	179574	66405	174836	45308	210153	239209	118452	148656	15180

4-methoxy-9H-xanthen-9-one	12.231	249.05415	Xanthones	31950	38874	53417	31863	37686	148351	56494	32458	104677	16301	45191	45083	25829	28856	33781	5392
4'-Methoxychalcone	10.652	239.10472		117	1307	170	312	89	118	74	557	27521	22504	421	13435	93	560	348922	144
4'-Methoxyflavanone	11.553	253.13068	4'-O-methylated flavonoids	1188	729	5835	1592	612	16679	754	4225	644	10890	1364	793	738	865	3877	5622
4-Methylabyssinone V	1.006	421.22247		51	1880	92	2312	6041	3626	5151	2083	5109	19348	1661	4309	8212	3686	3755	669
4-Methylumbelliferyl glucuronide	13.502	351.05695	Coumarin glycosides	6272	14622	11157	5688	5940	14475	11319	7387	6461	6147	12393	4767	7396	5348	6898	3599
4-nitroquinoline 1-oxide	7.286	189.03654	4-nitroquinoline N-oxides	342944	417790	353957	315571	631975	293069	814102	320189	353763	165481	878706	526661	174383	171941	214709	74259
4-Pyridoxic acid	5.289	182.04819	Pyridinecarboxylic acids	48460	42037	29324	67627	61140	31948	59179	45612	54439	33521	56326	37167	30211	24700	37835	32252
4-Thiouridine	12.397	261.05646	Pyrimidine nucleosides	23009	62978	120253	34618	25914	15391	109230	41246	51792	24492	25257	58153	54862	29122	28084	2040
5,6-Dihydro-5-methyluracil	12.812	129.06488	Hydropyrimidines	135035	153515	154075	121107	136008	200566	156342	196555	158831	136321	206388	155422	207663	206940	158083	191733
5,6-Dimethylbenzimidazole	14.22	145.10222	Benzimidazoles	53339	59937	32894	69026	62843	39464	59934	75004	34571	38116	74108	69964	93226	57393	48326	54265
53FTA	13.867	341.01346	Medium-chain fatty acids	2204	2998	164	5484	3112	5175	2526	677	5601	2324	2680	3349	717	1128	8272	2651
5Alpha-Cholestan-3Beta-Ol-6-One	3.491	403.25272		5534	30446	40339	68226	49796	58724	42627	82486	46516	96935	27658	36959	6606	45220	76739	1381
5-Flurocytosine	1.332	128.01817	Halopyrimidines	5985	33966	22754	33992	3325	5449	6059	22692	13801	65950	11642	35538	31743	26080	57479	31617
5-Hydroxyindoleacetic acid	12.928	192.07516	Indole-3-acetic acid derivatives	7239	18597	9845	17467	10213	26965	10501	12791	19810	40087	50838	26843	11570	63848	16283	50
5-Methoxyflavone	13.785	331.05246	5-O-methylated flavonoids	1834001	1819125	2886317	1809216	2108272	4744618	3643106	3621766	1647154	1639295	3394220	1592776	3238739	4786298	2030960	1702738
5-Methoxypsoralen	10.519	215.03598	5-methoxypsoralens	24876	106720	15419	21282	75093	14204	32306	45569	24187	62011	55921	112447	135716	152525	90230	194101
5-Methoxytryptamine	1.339	189.11276	Tryptamines and derivatives	47779	23374	30474	77282	67951	65912	54412	28385	34330	43806	32032	31902	36797	59645	40901	795
5-O-Caffeoylquinic acid methyl ester	10.564	367.10785	Quinic acids and derivatives	379305	223438	518498	305323	486598	384911	611841	317069	1003767	835983	336164	482621	1217143	753130	531210	554965
5'-S-Methylthioadenosine	1.282	298.08301	5'-deoxy-5'-thionucleosides	2802	35750	25505	86	49540	17	51	1794	25	202	285	183	275	275	34	48
6-(methylamino)hexane-1,2,3,4,5-pentaol	3.055	196.10622	Hexoses	11162	23635	19128	20983	21858	2699	15967	35881	7147	42133	10838	18641	27808	29933	35028	19572
6,7-dimethoxy-2,2-dimethyl-2h-1-benzopyran	13.487	243.10887	2,2-dimethyl-1-benzopyrans	34725	6547	3401	2077	3666	26599	3482	5194	7142	5482	3914	3206	5604	22309	5754	2877
6,8-Diprenylorobol	12.737	445.13205	6-prenylated isoflavanones	8853	7574	24642	139283	45736	43549	27675	31888	131223	4551	17078	17128	83036	7372	25000	46436
6:2 Fluorotelomer alcohol	12.107	363.00345	PFSA	1811	1861	1744	2340	662	2280	2080	2206	1842	1003	2864	1350	719	2811	4419	19038
6-Hydroxy-7-methoxycoumarin	7.408	215.04871	Hydroxycoumarins	759	156	91847	265	493	68977	63727	757	340	106703	40697	125603	432	659	116624	354
6-hydroxy-7-methyoxyscytophycin E	7.29	834.53357	Diterpene lactones	2027	1733	2493	4017	4236	8959	16183	19065	4303	24421	24617	24560	3385	13997	26892	3229
6-isopropoxynicotinic acid	9.643	204.04565	Pyridinecarboxylic acids	66767	56803	24033	22224	36239	38315	46458	36145	21958	19167	38694	42441	39385	59533	26661	21800
6-Methoxydihydrosanguinarine	13.572	364.11649	Phenanthridines and derivatives	14799	15325	7729	18100	10456	21347	10141	16127	10916	6913	9417	12261	21933	11627	14249	12371
6-O-Methylarthothelin	14.906	374.96014		445451	474356	392958	427529	443149	476421	436482	448520	417063	535349	423542	466481	506636	563668	408532	346394
7,3',4'-Trihydroxyflavone	10.33	269.08707	Flavones	18204	569	13475	1181	9774	24590	7944	10930	426	15966	11123	22916	813	7787	10110	20007
7,8,7',8'-Tetradehydroastaxanthin	1.16	592.35492	Diterpenoids	7090	8131	11904	8136	12316	18841	12565	10865	7132	9538	6695	7366	3129	8835	7079	15703
7,8-Didehydroastaxanthin	6.622	594.37524	Triterpenoids	13135	6559	8866	19012	14674	12250	25916	6033	7997	23301	17469	22150	7171	9707	22417	37250
7-beta-Hydroxylathyrol	1.252	351.21588	Diterpenoids	24131	33317	21885	20371	22153	34976	21838	26716	29989	27142	30385	25039	35685	38838	20111	56905
7-Hydroxymitragynine	1.167	415.2085	Corynanthean-type alkaloids	36077	95073	68177	62392	75253	123120	77837	89677	39920	81871	15483	49768	42688	73430	56125	249699
7-Oxocholesterol	1.076	401.3392	Cholesterols and derivatives	322113	467667	231361	496931	312866	869123	635353	408469	386098	294659	213328	433142	192818	508977	426242	483056
8'-Apocapsorbinal	7.306	448.3002	Triterpenoids	12989	15577	4747	6259	5660	7968	3992	5163	854	16642	2863	3025	11447	17916	1581	720
8-Deacetyl yunaconitine	7.897	646.36584	Aconitane-type diterpenoid alkaloids	2251	9274	9999	7229	4417	5047	2891	10947	3636	5240	16188	9726	3134	7870	7214	5666
8-Gingerol	13.198	323.21835	Gingerols	6318	5951	4018	6437	8723	9793	1698	9293	7811	6995	19336	8782	5309	51368	4161	4288
9-(2,3-dihydroxypropoxy)-9-oxononanoic acid	13.112	261.13403	Medium-chain fatty acids	43917	60154	37371	48079	49974	46391	47015	21531	53962	30594	40334	25233	24433	24465	35558	23285

9-HPODE	1.293	311.22592	Lineolic acids and derivatives	29600	15441	23911	23730	32135	32563	26011	18763	21842	35558	18234	15808	16126	15758	16795	32680
9-Trans-Palmitelaidic acid	1.27	253.21695	Long-chain fatty acids	9609	6689	6074	8219	11073	6462	6356	13983	9183	8740	5205	5748	21370	5097	6787	28557
Acacetin Diacetate	13.513	367.07993	4'-O-methylated flavonoids	3468	3218	1459	2617	1719	941	1489	2860	4636	390	1615	1115	2387	8387	4700	17097
ACARBOSE	14.391	668.25726	Aminocyclitol glycosides	18674	15321	8757	19500	6699	18153	13253	8628	16116	15895	20355	13881	7349	16755	12902	9760
Acetaminophen	0.721	152.07118	1-hydroxy-2-unsubstituted benzenoids	4506	13222	18777	5069	11277	6466	5847	4542	882	856	918	872	241	147	627	405
Acetylcarnitine	11.477	204.12202	Acyl carnitines	2200163	966373	1114603	1457127	1583356	1058980	1691335	906453	2058525	3178906	2534654	2101117	1369744	863963	1736609	848336
Aconine	7.532	522.28564	Aconitane-type diterpenoid alkaloids	21953	40183	9761	9233	17885	49157	4312	7182	35452	31590	12792	25705	22459	24807	13881	18149
Actrarit	1.361	192.06523	Benzene and substituted derivatives	12151	7627	7705	6292	54549	13922	62253	7965	5233	8591	4697	4924	3114	7038	5981	26003
Adenine	4.866	134.0605	6-aminopurines	27990	24639	64096	59716	22547	10562	39826	43770	68536	36990	61639	32707	21899	5838	99652	3219
ADENOSINE	13.5	266.08319	Purine nucleosides	16601	10478	6717	6263	6993	15240	7653	6407	5995	2941	13115	3139	6320	6489	2848	8377
Adenosine 3'-monophosphate	13.768	348.06976	Ribonucleoside 3'-phosphates	239710	154912	83160	137502	163796	104606	112179	182154	256391	212063	130123	144305	113507	355522	121517	127249
Adenosine 5'-diphosphate	13.836	426.02158	Purine ribonucleoside diphosphates	308835	178789	235645	285320	343476	128582	202856	252815	233972	113068	114081	186340	218439	95993	309630	21632
Adenosine 5'-triphosphate	13.848	505.99179	Purine ribonucleoside triphosphates	41298	24230	52311	48438	62895	25010	34833	29017	20975	11111	11121	23979	54489	6679	63228	3207
Adenosine-3',5'-diphosphate Sodium salt	13.837	428.03543	Purine ribonucleoside 3',5'-bisphosphates	129898	81347	93912	123406	151985	66468	80320	123269	116198	67397	63435	77471	131458	63692	126139	125080
Adenosine-3-monophosphate	13.778	346.05707	Ribonucleoside 3'-phosphates	393703	293140	141769	231848	317091	128590	178216	262724	489488	595725	203133	364649	152599	376961	237586	2300006
Adonirubin	7.151	580.41412	Xanthophylls	3741	3129	2524	2044	5026	1241	1439	3351	2698	3267	2064	3462	2593	797	1430	41844
Ajmaline	1.335	327.19974	Ajmaline-sarpagine alkaloids	6426	7588	1548	20374	16214	13682	2019	7862	14861	11098	5556	8626	18013	19761	66538	20556
Ala-Ala	13.874	159.08167	Dipeptides	5669	2122	2215	4323	9073	2702	11045	3396	1951	2325	14325	2876	2492	1026	1901	3609
Ala-Ile	13.097	201.12436	Dipeptides	9705	22770	11144	11036	12498	15006	14050	15316	911	8378	13216	910	17752	8008	5503	5459
Alanine	12.834	88.04097	Alanine and derivatives	822579	444031	518467	573022	416089	655292	456292	404593	577537	579108	893114	509935	298402	434455	556218	634030
Alisol C Monoacetate	1.053	529.36871	Triterpenoids	10999	12993	11078	18367	10580	15751	17473	15478	7636	9328	7664	7830	6798	10453	13132	21384
ALLANTOIN	6.85	157.03859	Imidazoles	72754	86095	132591	64423	19743	139832	96436	16086	46495	62234	88063	40485	18868	64123	35063	10955
Alpha Cyperone	12.101	241.15248	Eudesmane, isoeudesmane or cycloeudesmane sesquiterpenoids	454782	482407	461787	525300	197780	508337	157438	470072	1076305	595229	625446	562388	713076	583186	590345	364298
alpha-Chaconine	1.318	852.51862	Steroidal saponins	29319	33830	30476	24878	25353	25361	29940	36105	25842	32603	30265	33155	42365	28084	29892	6039
alpha-D-Glucose-1,6-diphosphate	13.943	338.99155	Hexose phosphates	11207	8618	7342	9775	11663	3345	4497	9795	14489	7222	2996	7747	7164	11793	21312	8458
Alpha-Ergocryptine	7.356	534.29541	Lysergamides	17921	39982	99236	45737	54491	35148	97673	61502	51379	54193	12728	123181	28132	107686	129864	30946
alpha-Methylhistidine	12.92	170.09042	Histidine and derivatives	477389	760780	616100	790098	575715	1239170	617562	634830	1038162	2104759	1990476	1169314	451224	2011729	893733	67610
alpha-Solanine	1.323	868.52893	Steroidal saponins	12434	20118	21297	19453	19996	21766	31744	26678	11838	27532	18863	23252	20339	29136	24447	6782
Altenuene	13.681	315.06662	2-benzopyrans	2772023	2276873	1630715	2295047	2671789	2254067	1984653	2390058	2546053	2207980	1948411	2094176	2832494	2623266	2332582	2591707
Ambroxol	13.49	376.98904	Phenylmethylamines	18104	16586	15872	23305	21890	22948	24654	18128	20514	18871	24917	27157	20187	27103	25049	14618
AMP	13.47	348.08862	Purine ribonucleoside monophosphates	26919	35671	75028	5987	37278	3001	7542	42818	83194	67951	56244	9807	48536	38596	70350	31854
Angelic anhydride	0.892	205.08672	Dicarboxylic acids and derivatives	330565	474728	469071	532867	485632	537009	536818	499743	248670	270975	249806	271820	417301	361981	352693	812076
Angelicin	7.282	187.05186	Angular furanocoumarins	88	18360	143	13457	3593	350	57	28497	9914	115262	7117	50919	32680	32935	42278	576
Arabinofuranosyluracil	14.223	243.07483	Pyrimidine nucleosides	70036	51332	46890	86628	67198	49770	75643	88725	39154	43864	83920	68499	118270	52634	49230	64060
ARABITOL(D)	8.227	151.06186	Sugar alcohols	122823	96495	102228	84316	105394	58764	80143	51074	43973	28476	86333	116403	134709	78924	84526	3825
Arachidonic acid	1.268	303.23325	Long-chain fatty acids	14898	19498	9696	21428	13441	19138	21873	32500	10834	48781	28140	26305	27739	32457	29687	15598
Arachidonyl dopamine	7.57	440.33163	N-acyldopamines	2107	2903	1440	2467	1698	3841	4215	4706	3751	5802	11393	890	4353	14696	6754	6532
Arctigenin	10.285	371.14249	Dibenzylbutyrolactone lignans	10426	14146	47196	16302	1478	18245	16162	29686	3416	5070	2385	3953	59481	3974	5370	10638

arctiin	7.867	552.25635	Lignan glycosides	3561	8339	6218	9143	4158	11263	4030	11924	5354	25777	17019	19069	3820	6240	10895	2560
Ascorbic acid	12.054	175.0275	Butenolides	325582	234046	362530	332127	332265	440084	215350	237696	642542	39059	195281	233063	343837	682244	481095	832221
asiatic acid	1.058	511.36084	Triterpenoids	10415	16859	18218	25962	22515	19067	31104	24664	11054	17312	15385	12815	11368	11281	18405	32347
Aspartame	13.608	293.10056	Peptides	28457	20164	3863	10769	16274	6325	15447	21266	13361	6955	7753	5401	558	3968	19643	7378
Aspartate	7.297	132.01392	Aspartic acid and derivatives	137444	147665	93	203964	206265	105	27	134965	159988	138626	343822	178520	95229	102846	140711	212
aspergillusidone A	13.81	371.00165		48528	57090	71005	44171	54129	70459	72236	64870	45319	66731	63524	57612	62045	92662	55330	37144
Astaxanthin	7.918	596.39081	Xanthophylls	40281	37727	47846	50326	32468	29287	47973	46726	36961	41552	48151	48446	31617	43688	64495	525
Atalaphylline	10.693	378.24991	Acridones	9991	45211	17265	73925	27982	18091	8228	6202	32839	34689	3435	26649	77532	24504	87962	44288
Atractylon	3.666	239.14842	Eremophilane, 8,9-secoeremophilane and furoeremophilane sesquiterpenoids	10875	12950	9568	41984	25722	30342	2114	13124	39702	44482	14677	38318	43097	33199	156092	67699
Atrazine-desethyl	9.816	188.07156	1,3,5-triazine-2,4-diamines	228650	392407	205241	234233	208855	461908	133021	183671	226588	166848	257489	312004	248923	209438	209432	188991
Aucubin	7.669	347.12491	Iridoid O-glycosides	31712	24967	16704	22234	49914	20405	23995	26451	32270	18684	32269	17614	27950	16238	26497	18157
aurapten	1.275	297.15253	Terpene lactones	18663	18789	11064	14326	87558	7114	8886	9903	21067	13819	9658	54222	24492	10488	10468	12901
Baicalin	13.701	445.09265	Flavonoid-7-O-glucuronides	6234	1784	864	6315	9258	1351	7360	4888	5504	857	505	1390	3628	730	4873	652
Baohuoside I	7.651	537.16833	Flavonoid-3-O-glycosides	3774	8131	7234	5700	11133	6232	2022	8123	11510	15659	2047	6720	15021	21864	37287	706
Barbatolic acid	13.746	389.05386		214059	53249	83155	73570	94053	159743	147828	99736	75243	7192	77478	28937	93788	470179	43035	9176
Benzene-1,2,4-triol	4.578	125.02411	Hydroxyquinols and derivatives	2314	11205	21903	12399	7538	6736	11028	19526	2526	6448	8311	16351	11857	5103	26447	378
benzoin	7.48	211.07709	Benzoins	3763	1588	371	7983	1324	11065	4820	2385	473	299	800	1071	3086	204	905	2097
Benzoylhypaconine	8.043	574.31042	Aconitane-type diterpenoid alkaloids	32976	45331	41432	58976	33238	60635	63203	21977	44539	44169	69427	24525	21474	31676	27534	4625
Benzoylmesaconine	7.651	590.31366	Aconitane-type diterpenoid alkaloids	8913	10239	13637	16206	21284	32733	27713	35149	5000	18225	10844	27819	3745	28897	44527	4111
bergenin	13.467	329.09634	Gallic acid and derivatives	8641	10768	7734	432	3605	9371	600	613	5690	11382	17545	4396	6145	753	565	256
Beta-Asarone	7.696	231.08377	Anisoles	3627	1660	5190	5806	4696	3522	1784	2235	9418	5091	1853	2575	3632	10193	26269	3254
beta-Carotene-5,6-epoxide	1.173	552.43658	Xanthophylls	1401	506	2168	8596	5163	3572	2198	1700	2012	1322	83	1637	954	941	1951	3930
betaine	10.787	118.08544	Alpha amino acids	55309	9172	44012	5932	761	36315	20574	9465	149079	61092	43154	78994	92731	11545	43781	6478
Bezafibrate	11.347	360.10193	Phenoxyacetic acid derivatives	12330	16984	14289	13054	18078	20192	19715	20350	13227	16752	17562	9320	26621	27740	12702	12772
Biselyngbyaside	0.988	627.45721		6945	16264	11433	16002	6188	5598	7936	6342	4025	3061	4021	7056	4509	2336	6586	13300
Bisphenol S	10.931	249.02567	Benzenesulfonyl compounds	56081	45673	57491	51226	32569	39353	77561	82478	41424	113820	33421	35832	21944	39933	39069	28055
Biuret	7.476	102.02108	Isoureas	41133	23048	15301	36017	53014	13881	33879	28176	25648	10149	45065	18793	12548	4851	26551	9013
Brazilein	7.225	283.08447	Indanes	90212	56151	6785	105010	136305	34295	7931	90902	127938	233600	144107	83559	66589	130226	92650	3061
Bulleyaconitine A	8.128	650.35779	Aconitane-type diterpenoid alkaloids	13546	20120	23365	37461	12299	17550	54838	20282	18059	30825	39846	14895	9772	28895	31396	964
Butin	13.736	273.04739	Flavanones	3092	8239	27669	7995	5220	41975	43459	37601	5095	6528	31313	10018	21865	53920	14466	2245
Butylamine	8.819	74.09611	Monoalkylamines	193	289	122377	85	283	12658	31314	199	441	6187	254	1383	296	443	1878	187
Cafestol	1.004	317.20926	Naphthofurans	15927	26480	14454	13453	16884	35723	18934	17729	17867	12214	8580	23588	21173	29120	21208	69767
Caffeyl alcohol	10.055	165.04364	Cinnamyl alcohols	38607	39010	29573	51128	18689	45074	41819	27312	51303	14776	36985	44637	35353	12807	40564	7698
Caftaric acid	13.806	335.03549	Coumaric acids and derivatives	24760	19088	13368	15098	20927	16690	15927	16841	15310	14879	19424	16268	20033	21942	13688	22610
Canavanine	13.795	177.08524	L-alpha-amino acids	1474	21419	35942	17764	13594	77266	52402	72344	8149	4952	40476	13467	59765	75511	26730	10090
canthin-6-one	11.392	243.04105	Indolonaphthyridine alkaloids	7349	7517	7548	13288	9148	3642	13104	13598	16987	6589	14289	4785	6946	4316	13979	9430
Capsanthone	7.716	582.42358	Xanthophylls	30648	34311	29405	15416	39545	40855	29749	33969	34725	30679	46870	35332	48305	45933	30816	52211
Captopril	11.684	216.05153	Amino acids	153732	281376	273839	276016	52626	38863	135427	305075	157809	186234	100478	256135	171536	237546	548533	201796

Carnosine	14.146	225.09801	Hybrid peptides	56200	104631	58832	32477	55768	36841	37155	33831	51258	54605	59051	38520	73192	51646	27426	29509
Carnosol	1.102	331.18741	Diterpene lactones	8342	13611	7229	11184	10746	21507	12738	7841	5384	9650	5344	11025	8027	13173	9043	19510
Carylophyllene Oxide	0.954	205.16084	Epoxides	4550	9295	1773	15012	7429	6256	17571	6334	6549	19570	9339	4793	14745	11605	8337	5040
caudatin	9.868	491.3024	Gluco/mineralocorticoids, progestogins and derivatives	2263	10223	14572	2524	2275	14314	1917	2158	3592	3930	9994	4972	4470	1406	4086	205
Cefaclor	11.524	366.04437	N-acyl-alpha amino acids and derivatives	14425	44242	23026	21069	9347	2276	30361	24691	9340	27877	16914	40535	3928	12711	32161	1255
Cefditoren pivoxil	13.597	643.1225	Cephalosporins	1544	2766	1515	2121	1332	3029	2460	2623	1450	2450	1819	1152	2677	2845	1748	3961
Chasmanine	1.011	474.27139	Aconitane-type diterpenoid alkaloids	34407	97539	15527	19685	202233	14114	17587	21583	27233	10564	5147	112893	74429	12226	10085	162026
CHENODIOL	1.294	391.2851	Dihydroxy bile acids, alcohols and derivatives	14793	4632	7012	1994	6323	6131	7813	4264	3113	11979	24852	2675	18897	4164	5982	3500
Chiodectonic acid	13.808	333.02713		17956	17679	11244	14764	14153	12833	10415	8188	11099	10226	9891	9381	12766	11984	12975	6770
Chloridazone-desphenyl	7.295	146.02528	Aminopyridazines	624	17132	117	18308	6335	4685	167	22455	7431	54276	33465	37647	16481	23466	26203	165
Chlorprothixene	7.382	316.09799	Thioxanthenes	434	180	7354	119	158	13368	16060	12028	18	6069	11502	7907	335	121	7309	178
Cholecalciferol	1.18	385.34348	Vitamin D and derivatives	20653	15854	18642	25788	19617	21712	18818	22354	16966	12713	13683	13541	12546	15674	14205	21127
Cholest-4,6-Dien-3-One	1.077	381.3187	Cholesterols and derivatives	19225	11144	12808	19352	13188	8023	11263	8059	14366	3824	8742	8861	4751	8587	10022	538
Cholesterol	0.994	369.35095	Cholesterols and derivatives	23933	9624	25338	46700	25883	11833	26357	29943	37327	19796	43435	15447	9526	21239	27933	105657
Cholic acid	5.073	834.60132		8169	3098	10445	11076	37125	15519	13054	20154	11689	6599	7867	18839	13638	6859	12152	30407
Choline	11.858	104.10504	Cholines	478998	148842	554251	363204	467912	361899	431887	655288	654195	626466	838454	699970	901093	654514	572353	520381
Chrysanthemic Acid	1.239	186.14935	Monocyclic monoterpenoids	27232	57272	45761	40861	68507	58934	38897	64914	24803	10801	39150	15305	19540	25303	29893	100717
Cimetidine	7.872	253.12309		1775	2220	7415	5386	5154	3304	2059	3908	5676	5131	1632	1421	3417	5780	19249	873
CINCHONIDINE	1.027	293.17911	Cinchona alkaloids	20270	35915	17906	17046	31797	19114	22980	12577	24653	21214	48257	18320	14901	18197	26744	10670
Cinnamoylglycine	13.588	204.05307	N-acyl-alpha amino acids	5458	6468	253	10319	9910	6667	5388	9766	15501	9914	5862	1730	7986	6743	9160	11303
Ciprofloxacin	13.468	332.14429	Quinoline carboxylic acids	6169	4031	3017	3380	4105	11077	3145	3426	7258	4450	18500	3438	3753	17766	2450	1215
cis,cis-Muconic acid	10.356	141.01743	Medium-chain fatty acids	86857	73305	62685	91232	77593	69561	79079	101239	69430	68371	87169	87778	96200	43493	76044	61299
cis-Aconitate	13.235	173.01062	Tricarboxylic acids and derivatives	145252	152148	181286	114414	131994	176108	198266	137000	94154	223083	188723	98619	118371	171260	108639	55563
Citraconic acid	13.043	129.01927	Methyl-branched fatty acids	25328	25639	35575	27761	21590	19654	34081	13608	20322	17290	28872	14788	22584	30464	16592	6823
Citramalic acid	13.027	147.03285	Hydroxy fatty acids	136150	119806	158957	124677	185872	177022	164862	57553	145198	132506	119055	62544	97726	178522	83579	142027
CITRATE	13.704	191.02176	Tricarboxylic acids and derivatives	28558	96046	117029	26529	25215	11105	10567	10310	74434	82250	101684	65576	77699	113750	75711	9783
Citropen	1.459	207.07703	Coumarins and derivatives	81986	162866	129138	104985	34400	53501	101170	169049	79217	164824	88731	190859	212347	135867	120368	201
Citrulline	13.859	214.05775	L-alpha-amino acids	124389	67520	99543	65356	59958	113275	99372	54218	112924	84814	100391	69650	95517	59193	54754	56857
Clarithromycin	6.777	746.48645	Aminoglycosides	11386	10404	3958	16117	8293	9189	10825	4812	11134	8787	9720	8213	4395	3424	14227	210
Cleviprex (Clevidipine)	13.615	478.09479	Dihydropyridinecarboxylic acids and derivatives	4037	5223	3133	4407	4764	4714	4178	4821	5132	5690	3951	4632	4582	7338	5592	2150
Cochlioquinone A	1.036	531.30713	Oxanes	20511	3982	3837	1325	10487	24	5603	27038	2653	18	9095	2388	2676	1347	4110	3301
conessine	1.111	379.31161	Conanine-type alkaloids	7964	3320	15436	29223	43648	60093	7186	6086	25693	27395	394	38823	13418	14136	23970	24929
Convallatoxin	7.72	549.28796	Cardenolide glycosides and derivatives	3792	2494	1450	3132	1953	5140	1571	1318	1595	559	2105	527	864	657	1862	828
Crassicauline A	7.583	666.35193	Aconitane-type diterpenoid alkaloids	8262	16899	8139	15174	9245	15141	12839	3083	7248	17946	10928	8286	7583	13513	19555	1402
Creatine	13.105	132.07532	Alpha amino acids and derivatives	660151	2366825	2860448	1389023	2708995	2933399	2378948	1577558	2941391	3652190	2699739	2669774	2218350	2494101	1637465	2364743
Creatinine	6.575	114.06558	Alpha amino acids and derivatives	973350	1053360	945889	859242	759930	1454636	593135	509534	492572	440955	772213	522376	370942	582265	750709	432075
CRUSTECDYSONE	6.978	498.34454	Hydroxy bile acids, alcohols and derivatives	133494	94681	252286	184481	35685	147078	319182	174698	212205	110963	133689	200756	187590	73185	89694	66528
CUDA	7.451	719.52472	Medium-chain fatty acids	29094	248	3329	14811	18210	9192	27206	2170	1279	3120	667	875	194	1157	15846	461

Curdione	11.205	259.16443	Germacrane sesquiterpenoids	34811	13681	50852	41269	35794	17165	66906	94698	106669	175478	67131	39131	57532	95271	153990	35756
Cyanidin	12.05	288.07416	7-hydroxyflavonoids	213736	159998	170267	67842	121118	200830	112128	131463	119607	56186	151627	31544	121741	173619	58144	16430
cyclopamine	5.836	412.31738	Jerveratrum-type alkaloids	406402	411789	359614	322807	438866	428242	459059	472502	271655	210136	361174	342725	479712	409140	368069	152542
Cyclo-prolylglycine	5.672	155.08061	Alpha amino acids and derivatives	64043	16576	37896	72763	227801	26812	156432	50774	49673	44700	193801	58266	4220	2317	37944	5374
Cytarabine	8.808	244.09518	Pyrimidine nucleosides	15929	12008	4794	16823	8916	14548	10664	9172	24712	18291	8887	18386	23054	24574	34603	26192
Cytidine	8.824	242.07794	Pyrimidine nucleosides	14310	18743	7148	19077	10472	5972	16022	16263	27012	30439	12657	53222	33584	41648	54711	35377
CYTIDINE 5'-TRIPHOSPHATE	13.766	481.97443	Pyrimidine ribonucleoside triphosphates	7751	7608	1448	4784	5903	1855	3060	4273	10189	10424	2975	6512	3146	8230	3297	22414
D-(-)-3-PHOSPHOGLYCERIC ACID	14.719	186.99516	Sugar acids and derivatives	855635	685891	423823	616500	1046710	518607	608221	671767	970168	933477	888072	634415	1017854	1145975	545547	1555420
D-(-)-quinic acid	10.703	191.06027	Quinic acids and derivatives	3776	1216	3052	2737	1049	1771	2734	808	870	1725	1238	942413	1523	1024	1040	189
D-(+)-Malic acid	13.444	133.01425	Beta hydroxy acids and derivatives	882213	773316	1129406	833844	591234	602987	1014673	970106	718632	1028523	800729	565157	970303	730697	820114	1296645
D-(+)-Pantothenic acid	7.467	220.11708	Secondary alcohols	492	561	65685	368	112	89325	95723	84796	168	80087	58743	56179	788	143565	57939	160
D-(+)-TREHALOSE	7.662	377.13568	O-glycosyl compounds	92827	75297	42392	75918	219759	61099	70218	82057	95354	61191	87812	59432	93506	72937	64900	110981
D-2-Aminoadipic acid	13.74	162.07994	L-alpha-amino acids	19381	19470	16844	14651	17441	13274	13289	6564	20163	12101	9891	15890	5606	24530	5798	5287
Daidzein	14.905	292.95853	Isoflavones	334174	425788	390559	357145	396095	378758	410081	384003	377775	413791	423366	310012	463992	501223	394524	309392
D-Ala-D-ala	13.54	161.08968	Dipeptides	9165	2882	4461	7739	16762	4018	16027	6313	5185	4294	19476	4207	2232	1333	4053	1430
Dehydro fucoxanthin acetate	8.034	682.42047	Xanthophylls	46406	37440	52835	48117	18964	28239	43561	42057	40635	35596	23403	51009	30774	25909	36850	9421
Dehydroeffusol	1.154	251.1055	Phenanthrols	12782	16404	7292	10065	27229	24375	22456	15370	4782	9734	7163	8824	4180	9213	5967	18245
Delsoline	8.114	490.28601	Aconitane-type diterpenoid alkaloids	2512	1316	5326	3381	4987	4988	1959	2807	4164	4387	1450	2966	2323	4663	8235	627
deltaline	7.804	508.26205	Aconitane-type diterpenoid alkaloids	19821	63076	69626	33897	29460	33129	29159	42451	22917	26323	55923	42481	13944	35948	36796	1758
DEOXYCARNITINE	13.74	146.11671	Straight chain fatty acids	25133	21103	10467	14445	16552	23679	16960	11250	27254	33760	35791	12863	15770	30530	12956	15483
Deoxyvasicinone	13.288	187.07164	Quinazolines	43957	57166	64537	53252	55472	99955	88940	89698	52413	48674	77572	56754	95218	95389	65842	70018
DErySphingosine	5.917	300.29089	1,2-aminoalcohols	1422	2288	371	1609	1208	1161	2883	1058	3616	17592	1259	2239	7278	10230	1516	503
D-Erythrose-4-phosphate	0.652	199.00839	Monosaccharide phosphates	46898	59540	27140	13832	13623	17277	14482	9574	7670	2869	4298	6759	7627	1705	3383	2742
Desethyl atrazine	9.684	188.06967	1,3,5-triazine-2,4-diamines	125981	218607	151384	149154	151388	262505	177669	104618	158299	173673	244646	232020	184463	84188	196050	85360
destruxin A	7.068	578.28937	Cyclic depsipeptides	1784	2377	530	317	7405	3627	1136	16102	2730	1011	14624	2100	11727	4162	1067	3110
DETHIOBIOTIN	7.266	213.12602	Imidazolyl carboxylic acids and derivatives	39065	8896	5563	22354	18635	324	19349	29084	31887	2259	2804	8527	32547	3215	32498	49
D-FRUCTOSE	13.808	179.05965	Monosaccharides	39168	40868	29595	26697	22520	27244	28767	20193	27408	29429	23853	25733	22973	14568	21757	26876
Dibutyl phthalate	0.892	279.15848	Benzoic acid esters	771107	1139155	1163378	1315457	1157189	1274115	1268883	1199435	568675	623142	565011	631494	968383	865158	836127	1991972
Dihydroferuloyl Octylamine	1.515	310.23685	Ferulic acid and derivatives	25249	61111	36186	31039	16583	3779	72615	53112	34160	27801	15584	14822	2329	11142	11220	2800
Dihydro-4,4-dimethyl-2,3-furandione	13.639	127.05125	Gamma butyrolactones	10936	13663	14812	12503	14672	15721	18651	11629	13467	19082	22805	17016	13294	12143	12944	19544
dihydroalbocycline	0.946	333.20181	Macrolides and analogues	12532	21746	18029	12816	14797	25496	17021	27356	9653	4334	9079	8343	16294	20405	12369	69001
Dihydrokaempferol	7.257	287.06015	Flavanonols	14484	23779	473	8410	9242	1110	15634	11270	10262	11613	16754	13803	16724	16046	9773	150
DIHYDROSTREPTOMYCIN SULFATE	13.486	682.117	Aminocyclitol glycosides	2827	3048	9745	396	4818	331	875	5093	9500	6436	7006	976	4545	6230	11606	2528
Dimethyl sulfoxide	2.995	79.02052	Sulfoxides	120471	24617	22150	35440	89430	119559	99982	38332	40297	40637	39633	42092	82701	258476	62570	60033
Diosgenin	0.984	415.3566	Triterpenoids	4628	7052	5769	6262	4471	8594	7975	9230	10865	3560	1911	4816	7530	4145	7977	29776
DL-2-Hydroxyvaleric acid	1.348	117.0564	Hydroxy fatty acids	54161	16902	39857	41360	76970	48954	47597	18442	17702	20316	18826	28731	19289	14365	17257	41916
DL-3-Aminoisobutyric acid	13.589	102.05631	Beta amino acids and derivatives	52272	65053	59718	62016	49214	50598	72583	66405	48953	74565	71858	64881	34077	48419	62279	77610
DL-beta-Hydroxybutyric acid	6.302	103.04113	Beta hydroxy acids and derivatives	14729	2934	8042	2891	1889	3295	3538	1067	1877	1580	3421	2946	777	3675	1272	87698

DL-Cystathionine	0.733	223.07553	L-cysteine-S-conjugates	8632	9061	13588	4125	5187	18375	4498	2835	576	1353	3133	1555	746	2740	2854	5301
d-LIMONENE	11.515	159.11162	Menthane monoterpenoids	6315	1809	1909	7644	37430	1208	11605	3997	792	2507	36718	6613	353	407	932	674
DL-p-Hydroxyphenyllactic acid	6.603	181.05568	Phenylpropanoic acids	25880	21453	39386	1396	7126	33523	3030	7082	3747	6645	19943	24656	14845	2104	3111	28258
docosanoic acid	1.086	363.31662	Very long-chain fatty acids	3945	11120	10149	5647	9879	8403	6320	6524	5008	8863	2444	7387	4581	4730	3881	13655
Dodemorph	1.412	282.27554	Morpholines	156549	107576	78910	207968	124150	127136	106696	132872	179924	95501	340670	112270	67171	173038	139601	93616
Domperidone	10.336	424.13501	Benzimidazoles	9134	6669	13213	8495	9956	9340	10571	12031	8484	27066	7135	5726	14001	6813	7001	10495
D-PANTOTHENIC ACID	7.373	218.10635	Secondary alcohols	262988	345446	56795	195060	145623	96975	169741	222766	100273	127516	133992	150683	423319	425544	122286	61347
Ecgonine	1.248	186.11215	Tropane alkaloids	987	1753	2218	1494	1110	1432	2531	1702	1202	995	1995	1601	1794	1315	50277	1778
Ectoine	12.121	143.08191		37306	33425	36880	25045	37756	52122	75037	44005	42708	23191	48766	28210	54055	44197	18595	35243
Ellagic acid	14.153	303.02789	Hydrolyzable tannins	4677	10546	9042	3692	4586	5946	5020	3757	6840	6643	6563	3461	8609	4886	3485	11335
Ellipticine	13.073	245.11539	Carbazoles	45233	45263	373275	74531	22220	826299	52428	632730	12437	747155	139509	17065	34513	36480	380762	1026986
Erucamide	1.09	338.33829	Fatty amides	58865	278422	183507	246498	238239	250009	449655	118111	66649	340959	147077	163472	141656	132820	146802	419277
Erythromycin	7.599	734.45416	Aminoglycosides	100916	99989	120561	87215	109029	92207	112077	100832	94248	57881	99193	91506	80963	68716	78473	51254
Erythromycin A enol ether	5.728	716.44922	Aminoglycosides	11114	32323	15039	35175	18315	12697	6530	44803	19430	7171	65717	7394	48622	5193	11479	11494
Ethylbeta-carboline	10.715	195.08878	Alkaloids	2449	13002	5763	11151	8646	9375	22322	5938	3638	2841	5583	14861	21366	6496	6285	21606
Ethylbutanoate	3.268	115.07871	Fatty acid esters	8377	5293	5385	11360	5625	4864	6685	6068	9128	4715	10300	5610	6138	4547	3564	369
Ethylenediaminetetraacetic acid EDTA	13.743	293.0975		1387838	573158	1030089	583751	1226629	2855669	1543683	1870082	772094	342115	1132114	295753	1961453	3307601	487409	982182
Etidronate	15.104	204.97108	Bisphosphonates	54139	52319	51245	20523	57184	54619	61003	59186	48870	24004	53908	55020	30650	22088	29374	56026
Eupafolin	16.859	317.05344	6-O-methylated flavonoids	147952	261290	123900	88665	232186	61673	23027	156933	51039	155497	90453	30246	262102	39600	159427	44678
Euparin	13.726	215.06761	Benzofurans	19318	30559	77909	27280	21666	127776	124254	106712	16689	16197	81584	28622	75647	145170	31133	8843
Euphorbia factor L14 - Lathyrane diterpenoid	7.741	676.3576	Diterpenoids	16704	33612	38320	36267	17379	27036	42052	55050	18558	26541	41831	29873	28626	23077	20191	3883
FA 18:0+2O+SO4	1.124	395.18555	Oxidized fatty acids	2560	6757	26607	1026	1500	63	280	3847	952	424	475	492	7383	319	405	215
FA 18:2+2O	4.234	311.22681	Oxidized fatty acids	130904	96220	82459	69676	56329	66366	68993	39135	42298	99239	47569	48090	48001	86973	25567	38357
FA 18:3+1O	1.255	295.22604	Oxidized fatty acids	61154	72432	67393	87579	58060	35834	60239	52380	70660	87758	60354	29449	43914	24282	28530	53124
FA 9:2+1O	1.296	169.09148	Oxidized fatty acids	24452	21369	21759	57160	74783	24996	66425	25909	22109	28302	60388	18825	12574	17706	34837	8706
Famotidine	10.683	336.07751	2,4-disubstituted thiazoles	32142	48268	42733	2210	3527	2494	20770	6230	4988	16140	10507	8070	31770	4221	2472	632
Fenoldopam	10.855	304.07358	Benzazepines	286906	122236	116625	107174	116939	171868	198073	302620	212115	481199	243461	153026	357710	360949	127251	214381
Feruloyl Lactate	6.129	281.04129	Ferulic acid and derivatives	82111	15531	12304	5662	7313	30426	49012	4902	8888	49841	8336	64808	39347	34457	8131	5947
feruloyltyramine	10.231	312.17184	Methoxyphenols	8818	8569	36939	15199	4141	10150	17957	14956	3699	15691	6228	20482	12002	6063	6845	15208
Fipronil	15.057	434.93863	Phenylpyrazoles	22011	17598	16901	18284	10730	16152	19490	22660	17822	18482	20316	13843	18773	18181	13651	16274
Fipronil sulfone	14.043	450.92493	Phenylpyrazoles	160860	116349	111975	129447	117953	150965	125505	122862	133645	97606	107460	85051	125448	82184	102519	169943
Flavanone base + 6O	12.705	319.05743	Flavanone O-glycosides	28776	26534	17618	36274	19583	40291	16600	27365	41672	8119	35047	31462	33707	7186	19480	55841
Flavin adenine dinucleotide	13.501	786.16455	Flavin nucleotides	3397	3614	3567	3311	2758	2926	4313	3578	2934	3433	2504	3832	3214	2835	2413	2226
Flavone base + 4O, O-MalonylHex	13.57	535.09406	Flavone O-glycosides	6664	9333	5483	9423	4840	5671	6377	6287	8104	7805	2020	9261	9558	11117	5005	3669
Flonicamid	12.619	228.05585	Nicotinamides	23971	18498	25144	41409	34901	25557	42118	30282	31773	21000	33445	30225	18418	24957	29458	28379
Fludioxonil	12.228	247.04073	Benzodioxoles	164039	167618	255306	153628	187712	408520	313707	140526	291276	45355	169508	189901	95138	99104	150280	80050
Flufenacet OXA	8.1	224.0668	Alpha amino acids and derivatives	21861	23168	28749	26235	28323	6071	22475	26087	16146	26374	20258	51529	36114	27272	88452	5051
fraxin	10.416	369.12286	Coumarin glycosides	185083	143365	311622	170872	116975	199516	209076	285649	80863	135273	72837	154539	516866	87306	103119	187868

FT-PFCA	14.862	926.97717	PFSA	34197	15551	26407	29829	28033	24094	36651	26326	36014	34438	17748	40257	23875	30899	29321	24469
FT-sulfone	14.984	532.98816	PFSA	44477	58083	27880	37013	38154	58230	31854	62222	45961	59846	58172	44616	58177	60306	26811	51748
FT-sulfonic_acid	13.816	562.94244	PFSA	17481	36428	28980	27068	28049	25990	35233	30998	20539	44152	44339	42405	28517	39126	29985	33457
FT-sulfoxide	13.792	752.00476	PFSA	3967	3232	3099	4596	5278	2875	3440	5772	7194	6035	2924	6051	2749	5889	4383	21849
FT-thioether	14.795	387.00223	PFSA	462902	387722	335244	359424	330468	372699	667649	308928	229476	169682	260348	237547	696077	612047	599093	383846
Fucoxanthin	1.344	658.40424	Xanthophylls	663	590	5771	1060	4806	986	11404	2567	562	5835	569	2042	484	3055	2839	844
Furanodienon	8.417	253.11916	Germacrane sesquiterpenoids	9218	11024	9962	8556	12533	4887	8805	14633	11118	6229	32970	5772	8391	1152	16187	4567
Fuziline	7.195	454.29239	Aconitane-type diterpenoid alkaloids	11009	7268	148473	5577	77151	16606	88028	15535	9559	116492	19725	14908	17910	36778	163365	38722
Gabapentin	3.155	172.13165	Gamma amino acids and derivatives	74549	87302	99760	157071	202379	82577	53377	123731	33202	5681	119382	40654	5682	38869	74439	11876
Galactose	10.731	198.09541	Hexoses	104482	203906	111651	65887	40445	134789	123982	159409	113712	119911	82968	89402	304682	246601	79330	10447
gamma-Glutamylglutamine	7.333	274.09912	Dipeptides	46385	3756	558	17081	96382	109	9646	6602	27588	271	10399	4112	1537	5934	10203	22954
gamma-Glutamylleucine	12.382	259.1322	Dipeptides	22652	32247	23346	27084	16439	15998	22547	17716	19233	39190	31912	23504	10305	12484	18816	58620
Gangaleoidin	14.689	410.987		225873	254778	194880	195040	195978	201702	218740	202989	180609	230523	192863	194697	206700	230131	136535	241236
Ganoderic Acid G	7.51	555.29797	Triterpenoids	33830	27993	22045	27363	24916	22443	27135	33041	32323	15983	21047	36114	37866	22301	21555	21460
Ganoderic Acid H	7.513	573.30859	Triterpenoids	40575	32133	30579	21713	37577	30449	35550	47053	32100	30096	20931	37971	34698	28556	29809	22512
Garcinolic Acid	7.279	664.35291	Pyranoxanthones	6494	18045	2436	7890	20438	3729	2150	16043	14629	19460	5710	9340	10463	13479	8074	1934
Gaultherin	2.921	153.06465	Phenolic glycosides	104862	287827	221350	26565	45286	91242	57305	40383	22300	9008	13285	28546	81769	43353	14920	14140
Gemcitabine	9.486	262.04822	Pyrimidine 2'-deoxyribonucleosides	3512	10859	6424	5722	4657	6738	6511	5394	4974	5619	4016	10182	2851	5239	8657	9856
Genistein	14.689	562.92487	Isoflavones	70673	65550	73176	62998	69238	78669	73795	66179	53586	61649	67747	58682	83988	80152	77180	60018
Gentiobiose	13.404	360.15164	O-glycosyl compounds	661	812	13520	188	859	1555	1826	220	816	25946	718	46089	501	1200	638	733
Germbudine	7.917	710.42072	Cerveratrum-type alkaloids	47088	36782	61687	68066	34045	36829	56208	36470	36637	40520	42447	52328	41797	31226	58851	2192
Germinaline	7.501	752.43762	Cerveratrum-type alkaloids	43751	29339	73115	79007	32404	65077	86574	75073	24105	38080	40657	73704	66394	39757	70470	37684
Gigantol	7.871	275.10922	Stilbenes	2483	3154	8855	6618	8558	3425	3330	6102	7552	6292	2768	3139	5274	10575	37366	2272
Ginkgolide A	9.628	407.13788	Ginkgolides and bilobalides	17297	17265	34524	1279	26117	713	3653	19417	30133	54869	39644	6315	37132	26230	39501	59344
Ginsenoside Rg6	8.57	765.5097	Triterpenoids	3747	1187	1872	3721	6404	1775	3059	1748	2158	1399	1410	1057	647	800	1656	66
Ginsenoside Rh8	6.759	637.42603	Triterpenoids	16283	28082	44407	24643	27331	46427	16363	14762	48831	7191	29281	21552	28346	30411	22572	3429
Glucohirsutin	10.852	492.11713	Alkylglucosinolates	3950	5364	9543	7759	6762	16117	10362	10767	17750	19433	15924	10625	17998	16427	8305	4014
Gluconate	12.879	195.05161	Medium-chain hydroxy acids and derivatives	313704	204807	335904	476469	125549	397966	850164	159175	360152	455446	367112	295013	200840	224394	135604	57816
Glucose	10.746	179.05724	Hexoses	1846312	1431853	2915330	4044976	2234601	2110163	1162074	5231498	4103661	1128081	3561437	4156295	5260504	6171108	904817	635607
Glucuronate	13.223	193.03737	Glucuronic acid derivatives	16631	55357	50531	9391	15291	27576	37816	11206	25640	33216	27848	30197	38104	27658	27174	17902
glutamate conjugated cholic acid	7.707	538.34436		16864	93821	59152	75921	121822	32543	31605	54393	30779	81905	83684	55265	17788	183322	79103	9006
Glutamic acid	13.583	148.05922	Glutamic acid and derivatives	1051270	1279080	1082846	1151222	1024880	1187964	1319411	1258912	1307024	1909835	1505372	1266946	936272	1198115	1222069	533581
Glutamine	13.636	147.07651	Alpha amino acids	209012	202049	146676	196088	243131	212183	261085	197399	202232	295711	308199	217066	255086	274408	211593	133467
Glutaric acid	12.868	131.03737	Dicarboxylic acids and derivatives	5008	4335	5156	17222	4712	5366	5068	3572	5595	6492	3760	3844	4180	4976	4198	5004
Glutathione	13.497	308.09314		15623	24966	60249	11947	24593	11220	9961	24902	54894	47906	41004	18434	27039	25169	61115	15536
GLYCINE	13.497	74.02598	Alpha amino acids	236101	254940	208719	215252	152058	183397	253473	241538	158963	276527	220358	173672	217574	135858	184043	292088
GLYCOCHENODEOXYCHOLIC ACID	7.248	450.3197	Glycinated bile acids and derivatives	133425	34232	1532	86203	52941	9552	1011	87482	13220	142261	100255	52810	69669	250992	54804	2553
glycocholic acid	7.347	464.30133		189823	282500	121076	95506	77802	48509	20683	41647	5866	62073	44868	43445	91713	186780	24852	18943

glycoursodeoxycholic acid	6.755	448.30606		493333	202483	1854605	293023	473926	1401183	222671	318971	46367	527499	882002	235938	239943	1020310	248912	22696
Gomisin H	0.964	419.23471	Hydrolyzable tannins	10133	18379	18639	18619	8171	9030	12087	6261	5099	8245	5801	6964	4366	5661	2451	15836
Goniothalenol	6.478	250.10236	Furopyrans	64640	25158	22764	53689	10788	84285	70254	83855	83198	100955	69086	72786	73212	99772	163443	10560
Gossypetin	13.488	357.00507	Flavonols	162276	154627	147006	209485	228067	191883	224820	179423	222370	168659	257826	274900	167134	262304	301617	105039
GUANINE	8.501	150.04411	Purines and purine derivatives	9131	16477	7068	6420	15260	14516	13161	21989	21692	49222	16582	13068	6677	34587	19968	82775
Guanosine 5'-diphosphate-D-mannose	13.884	604.06787	Purine nucleotide sugars	7537	5604	7980	2872	12367	5215	6024	8484	9065	1436	1286	4335	7046	5643	538	3025
Guanosine 5'-monophosphate	13.48	364.05823	Purine ribonucleoside monophosphates	11752	15763	38410	2603	17630	1316	2989	19694	30106	28576	28593	5839	20834	20661	30031	6003
Guanosine-5'-diphosphate sodium salt	7.162	444.0488	Purine ribonucleoside diphosphates	220	5320	16168	90	6844	6638	4869	7621	3950	10477	9856	7093	5330	13142	10116	0
Halovir B	5.263	860.58441		22994	11550	9658	10736	14021	13953	17919	20680	14493	7950	7619	11835	7703	13758	10029	4547
harpagoside	14.7	492.9899	Iridoid O-glycosides	191686	147011	138405	205471	182529	135731	196407	177262	147873	145726	223904	190855	204579	204666	191444	185582
Hecogenin	1.12	431.31439	Triterpenoids	14350	21315	11328	8973	15759	23614	14691	14581	17442	5774	5830	10740	7001	9372	13165	19299
Hectochlorin	14.781	666.97443	Cyclic depsipeptides	24434	18998	9079	20652	15805	14929	16682	17499	26447	21763	23461	24057	17514	37894	30663	9741
Hematein	10.209	299.07413	1-benzopyrans	9151	13792	12700	24385	21883	13192	4839	23912	16500	21788	5615	8135	28001	14615	10901	12649
Heme B	16.009	616.16992	Metalloporphyrins	162870	109238	32621	20116	325512	139359	70367	22576	495965	143538	401706	60432	46395	15639	9864	36791
heteratisine	1.053	414.21286	Quinolidines	26552	55647	33814	85936	48640	164647	118242	37871	43756	185150	28623	95145	42791	94760	58498	27561
hippeastrine	10.64	338.08411	Homolycorine-type amaryllidaceae alkaloids	27424	36276	32321	8038	11342	7131	19332	6783	25546	14839	38488	5062	32632	14697	6145	32569
HIPPURATE	4.72	178.05057	Hippuric acids	718213	214519	1098392	577205	327294	929066	380575	71598	929233	347852	209804	801730	561747	196197	242802	31525
Hirsuteine	1.071	367.21411	Corynanthean-type alkaloids	6255	10743	18121	40019	5712	73604	42773	24309	5484	53176	4984	53197	31457	50365	33095	119139
His	13.859	154.06364	Histidine and derivatives	16630	19619	17574	15975	18251	15132	22791	17836	16284	37283	44743	33783	23993	16860	26661	98760
Histamine	14.556	112.08556	2-arylethylamines	8371	18322	4637	4334	3040	5588	3907	3860	7032	9625	7127	12325	9748	5137	6138	13586
Homocysteine	13.094	136.05037	Alpha amino acids	26842	12038	7662	9034	32020	18004	9169	11360	52742	36237	29955	28527	28362	49190	25193	31343
Homoharringtonine	7.495	546.27899	Cephalotaxus alkaloids	20960	19397	7121	7409	23494	20082	8153	14152	14144	17245	14028	13359	31686	20583	8903	15393
Huperzine B	10.05	257.14896	Phenanthrolines	110976	170011	127717	110668	100288	180295	52575	77173	137134	142254	191576	102906	165210	61708	84029	71274
HYDRANGENOSIDE C	7.442	601.33563	Terpene glycosides	91153	80276	45982	57000	74506	49874	57721	93104	81557	55993	81461	86702	67474	113609	55305	37458
Hydrochlorothiazide	1.266	296.03525	1,2,4-benzothiadiazine-1,1-dioxides	2780	1082	2115	3656	5758	216	4266	3391	2019	3127	11260	6499	178	34	3481	1507
HYDROQUINIDINE	1.257	325.18741		59447	65046	34984	53996	305443	31261	25516	35128	85517	45324	24490	197303	116597	27346	23128	52711
Hydroxybutorphanol	1.323	344.22421	Phenanthrenes and derivatives	35757	36645	23141	71287	76329	84294	27156	34608	117192	50890	36770	58644	99130	80461	262579	116638
hyocholic acid	5.533	817.57135		108197	144477	15066	29198	68035	78630	59308	135202	137971	58168	61198	97566	60757	27745	81786	273363
Hypaconine	7.885	492.26334	Aconitane-type diterpenoid alkaloids	4288	9068	4999	6121	1321	3751	4781	5416	4157	5784	6507	5063	4752	2592	5249	20069
Hypoxanthine	6.358	137.04544	Hypoxanthines	475628	590701	345719	1383726	2193237	495424	767011	676262	3761104	2816755	500983	2746862	2193719	959692	6381571	1297805
icosanoic acid	1.113	335.29538	Long-chain fatty acids	9918	28345	26579	38186	58048	10374	60098	13476	7585	23372	16658	5171	8011	13504	11432	13914
ilimaquinone	14.806	433.00006	Prenylquinones	3347950	3700171	1893248	1878130	1828597	2107518	2175232	3837336	3259666	2618237	4215978	2418585	3753499	2911997	3677025	2226056
Imatinib	7.715	492.26685	Benzanilides	18658	25802	19970	23256	18112	3871	17357	9122	17995	17631	13311	12342	6880	16066	20318	339
Indinavir	7.289	612.37909	Alpha amino acid amides	51304	10465	149	7704	30119	7813	2891	13760	15163	9744	4691	9679	32547	23894	20133	1756
indirubin	12.405	263.07169	Indolines	6238	14686	21931	4282	5452	5875	20228	10171	14246	8374	3031	11443	14397	10552	6869	10758
Indole-3-acetyl-L-tryptophan	11.189	360.13354	Amino acids	439278	159874	591621	150658	190959	255748	390540	346599	148091	218024	231084	261526	242192	360257	117794	279694
Indole-3-carboxylic acid	13.151	160.02663	Indolecarboxylic acids and derivatives	14500	16274	14427	13455	16897	17224	18461	16507	16851	11450	15866	13199	17293	20056	19410	5155
Indole-3-pyruvic acid	9.655	202.0322	Indolyl carboxylic acids and derivatives	23904	20930	7558	7237	13947	9450	16512	12288	5739	4293	10580	13296	9876	17820	7742	4217

Indoline	9.644	120.08073	Indolines	461419	490792	427826	345462	401996	590254	554891	441969	378180	598455	531719	366309	306428	549101	351215	363210
Ingenol	1.103	349.20221	Tigliane and ingenane diterpenoids	26848	46868	28963	33391	27574	86724	44887	40958	20320	57246	17069	44063	36916	64819	38728	94397
inosine	7.884	291.07483	Purine nucleosides	4130	12456	17035	8280	16876	3994	6811	17730	20753	21851	3478	15611	26995	27223	49413	10882
Inosine-5'-diphosphate	14.701	427.0159	Purine ribonucleoside diphosphates	186507	172442	217475	149541	226450	153656	185809	187865	102321	224865	163797	85299	203998	282913	212316	151148
Inosine-5'-monophosphate	13.851	347.04178	Purine ribonucleoside monophosphates	1400981	1364687	717735	1436184	1015192	308172	635932	721844	2205604	706198	283841	1197617	952916	660311	1001815	792987
Iopromide	14.934	791.89557	4-halobenzoic acids and derivatives	7939	6773	7424	6746	6491	7145	5911	6955	6999	6570	5696	6008	7968	7886	6823	4719
Iretol	4.543	155.08533	Methoxyphenols	9891	2737	3070	12173	37355	3667	15415	4281	5409	2353	14756	5240	1126	377	3562	2964
irigenin	13.935	361.10251	3'-hydroxy,4'-methoxyisoflavonoids	10594	8662	6404	11438	8536	10890	10475	9880	10262	9547	9944	8848	12380	10405	10366	9467
Isocucurbitacin B	7.452	581.3056	Cucurbitacins	13774	19604	10772	9391	12944	10163	12851	16455	17607	14270	13949	18889	23612	23831	9669	12064
Isocurcumenol	10.05	235.16368	Guaianes	120245	308897	389444	198655	129966	489500	142291	126798	243374	218018	379841	169937	210461	101054	152635	191968
isofraxidin	7.136	221.01413	7-hydroxycoumarins	29570	16992	10245	22225	26938	13701	9806	31630	20207	41088	34950	28172	30060	23579	36045	13142
Isoguvacine	11.941	126.05814	Hydropyridines	4608	4146	2136	1850	1057	1738	1152	3366	4038	626	685	451	3839	22590	3505	17309
Isoleucylaspartate	13.06	247.12453	Dipeptides	1945	1477	26196	4089	919	64411	735	20300	935	35512	7271	1161	2027	3174	23962	1785
Isopimpinellin	11.56	247.05788	8-methoxypsoralens	17966	13621	4719	8601	26721	18664	44453	37176	12828	32950	96364	42613	46402	59426	34188	29329
isosakuranetin	13.451	285.1088	4'-O-methylated flavonoids	3881	1485	967	1594	1043	1265	870	1240	1932	586	669	423	2147	11320	2080	6700
Isosulochrin	13.382	333.08417	Benzophenones	44032	33123	17624	29121	25145	50387	21828	24680	33736	14878	10193	29579	46303	32285	20319	23860
Isotalatizidine	12.734	408.25616	Aconitane-type diterpenoid alkaloids	9905	1442	4310	13491	29270	6928	28072	11096	16024	3200	71181	6113	1319	316	5151	945
Juarezic Acid	0.704	172.99211	Styrenes	377596	535476	227408	169318	184597	145558	146659	91751	110712	42236	144644	105622	144704	44460	96190	66263
Kajiichigoside F1	8.985	651.43292	Triterpene saponins	27436	11128	29432	57023	46648	30392	26282	20653	19615	26407	37931	27310	11291	17392	26528	3185
Karacoline	1.097	400.23447	Aconitane-type diterpenoid alkaloids	7470	12187	18178	34393	7506	52382	48109	24602	6123	47692	3871	28729	18696	33120	30143	36630
karakin	13.837	522.06628	Tricarboxylic acids and derivatives	17967	25852	11900	11053	10873	13537	11052	8416	12035	6238	5107	17974	13345	11792	5428	5221
Ketoleucine	1.295	129.056	Short-chain keto acids and derivatives	357848	220639	408235	381969	241788	174283	457765	226030	156793	409503	409237	364901	288531	316348	373323	784376
Khasianine	7.595	722.45306	Steroidal saponins	59752	44081	46035	54221	57124	27375	74860	32577	33916	25682	37099	31948	25951	15811	31441	10501
Kirenol	0.962	361.22699	Diterpenoids	5841	8499	9030	6829	10643	11221	6115	9622	3570	1657	2566	5646	8134	4601	6551	34881
KOBUSONE	1.25	240.19322	Ketones	3071	7537	8077	8992	14276	10557	18663	5066	2415	3677	3937	3856	3556	2954	3095	9063
kojic acid	14.741	141.0172	Pyranones and derivatives	5520837	7919608	5850403	6017093	5134409	3938449	5131587	6686500	13987153	7208320	3599983	4751835	3454820	4331372	6999923	8519297
L-(-)-Phenylalanine	9.63	164.07196	Phenylalanine and derivatives	2020247	2182293	2644672	2335245	2771289	3099950	2775781	1905065	2084637	3280512	2221213	2511571	1767134	2056148	1882632	3716844
L-3-Methylhistidine	12.961	168.08018	Histidine and derivatives	57699	99734	59128	110304	91971	157170	83096	91043	101386	193004	191725	117496	61928	409911	111112	139669
Lactamide	12.838	90.05282	Secondary alcohols	119842	97886	146576	90738	96053	95160	125265	84968	76958	143639	121238	127061	110102	83253	112700	65452
Lactic acid	7.24	89.02481	Alpha hydroxy acids and derivatives	3916604	2344969	5109065	3068704	3116566	2831856	3960665	3266513	3360132	3353138	3112444	2443645	3811082	2755360	4485095	10763856
lapachol	10.985	243.09952	Vitamin K compounds	33955	40721	81570	77977	31187	76212	65412	74927	65011	120514	28285	35605	30985	54255	70119	38084
L-ASPARTATE	13.642	132.03358	Aspartic acid and derivatives	39763	49530	56181	41740	29522	55615	51561	64742	31893	102366	66496	43622	40560	51810	49828	111213
L-Carnitine	13.202	162.11232	Carnitines	1546626	1249158	1235507	1074612	1509469	1711942	720322	1573465	1585379	1614829	2545517	1641786	1841317	1580740	983272	1145128
L-Cysteine Sulfinic acid	7.121	152.00404	L-alpha-amino acids	90873	45557	5315	82799	107014	5198	20532	74836	59653	2539	7422	6536	35024	21891	57957	1184
Leoidin	13.731	413.03372	Depsides and depsidones	74460	39491	28642	36391	52989	35974	28286	25907	46974	30059	29372	28768	31466	51082	31448	60089
LEVODOPA	10.461	196.06242	Tyrosine and derivatives	27145	31252	23526	83326	54625	15613	62644	19051	27971	58206	41292	19017	12426	19729	23201	5481
L-Glutamic acid	13.588	146.04648	Glutamic acid and derivatives	2181089	2349422	2394943	2396455	2023042	2059820	2756152	2495203	2152374	2898603	2924367	2369140	1447821	1945687	2518137	3104222
L-Histidine	13.655	156.07553	Histidine and derivatives	139199	113966	216421	175193	162117	180923	225507	172072	158970	359505	276798	241584	146907	164082	153245	61309

L-HISTIDINOL	10.316	140.08504	Aralkylamines	39663	12017	4637	4920	8391	25515	6747	7970	10673	9380	2584	3862	12500	68557	9438	24022
Licochalcone A	7.368	361.13708	Retrochalcones	28464	42271	533	18922	61004	16210	34634	77917	64302	50145	43300	113430	97027	44697	45002	2961
Linalool	6.413	155.15514	Acyclic monoterpenoids	21629	12953	26812	23288	18937	20468	24461	27966	24968	29435	16938	30309	38656	22215	23162	7241
Linoleic acid	1.261	279.23389	Lineolic acids and derivatives	161049	223932	112252	189090	112014	45578	132502	203282	126345	196019	95906	156828	220219	46615	103663	213572
Linoleoyl Ethanolamide	1.123	324.29056	N-acylethanolamines	6468	12635	18685	13927	9610	19615	23108	18398	6173	19803	8008	10647	13213	12500	10694	36006
L-kynurenine	13.949	416.91367	Alkyl-phenylketones	12128	10189	10766	10447	13764	3673	4609	12300	15689	13228	5741	10860	8807	20241	26822	5386
L-LEUCINE	9.825	130.08795	Leucine and derivatives	4609271	2480352	4807172	4269456	5372094	6098317	3564052	3233527	3104562	7278724	5462712	3212338	3741958	4179927	2966246	2558752
L-LYSINE	14.548	145.10037	Alpha amino acids	113824	104708	40440	91432	104344	60631	101828	107672	75537	81051	121385	108841	143443	67593	71137	117790
L-Norvaline	11.062	116.07319	L-alpha-amino acids	1498136	1341231	1178674	1699349	1733990	1499202	1013450	1068151	1393873	1518377	1233619	1726397	899143	791254	1394232	2625523
Loggerpeptin B	14.393	1156.65417		10529	8616	13028	20618	11636	23678	17970	11395	13575	10597	14745	15493	19066	13881	23057	8173
Lorazepam	13.57	319.0123	1,4-benzodiazepines	3280	4407	1902	2806	3146	2650	1738	2159	4546	3436	4245	4144	4231	4034	3513	2355
L-ORNITHINE	15.873	131.084	Alpha amino acids	105570	76784	102943	74950	70024	87463	190337	85464	65431	162761	119209	126473	95940	60400	72043	97569
LPC 16:0	7.832	496.33896	Lipids	4621819	4333304	4114656	4923217	4050596	4096438	4890661	3239452	5531747	5065105	5217610	6446378	5749448	5506766	3800708	5736399
LPC 18:1	7.746	522.35272	Lipids	1168322	877163	852589	951088	1104305	1126883	961363	1023712	986254	999120	1134186	1140800	1276122	1762891	829698	1605804
LPC 18:2	7.802	520.33887	Lipids	1046640	626898	443819	251560	762561	609662	301233	512359	552363	356387	306717	647949	1185285	348690	228548	1547687
LPE 16:0	8.066	452.2778	Lipids	139721	199200	114929	198473	160939	176887	156350	78506	166087	250106	201813	160295	329657	248508	157128	27106
LPE 18:1	7.974	478.29523	Lipids	91614	125462	54916	71672	91479	107586	66019	73095	106243	140472	76553	70553	161338	109317	69337	28594
LPE 18:2	8.031	476.27896	Lipids	71790	99286	27694	11674	41069	49240	12330	23687	50807	27537	22396	47537	93839	24581	21691	22626
L-PIPECOLIC ACID	4.544	128.07246	Alpha amino acids	4906	1293	2961	5799	27796	4175	10818	2853	2387	1736	12942	2807	731	273	2241	1839
L-PROLINE	11.284	114.05757	Proline and derivatives	1464842	1822590	1299769	1707542	1920979	1792924	1746016	1236261	1337291	1590506	1671082	1444995	876979	1446468	1037944	942540
LTC4	7.965	624.31854	Oligopeptides	2947	8197	3130	12157	1603	3837	2476	2550	4607	5858	7511	4697	1852	4116	8509	31
Lucidenic acid B	1.337	497.23517	Triterpenoids	7085	8882	6194	17396	28546	18255	7846	11948	19328	12724	3339	13143	15449	17595	24283	410
Lufenuron	13.733	508.99103	N-phenylureas	6189	2517	1441	1716	2621	1374	2599	938	3726	945	1017	1900	1538	3374	1153	466
Lupinine	1.439	170.15306	Lupinine-type alkaloids	52483	64884	49630	73808	59265	59233	37397	52135	48615	37793	76588	43855	58670	48639	46589	75513
L-Valine	11.04	118.08582	Valine and derivatives	662855	456360	465335	531058	466191	536770	379377	448048	416268	656780	510711	409306	464797	406866	422960	280486
Lycopene-5,6-diol	1.249	570.45184	Xanthophylls	10782	22677	11505	5394	37744	10174	7423	12683	9623	8971	13984	23481	24654	7191	6739	39113
Lyngbyapeptin A	7.391	720.44147	Phenylalanine and derivatives	51209	31627	6134	186030	194648	56509	174430	108795	57570	89050	117459	79883	49918	41150	90917	9770
Lysine	14.544	147.11086	D-alpha-amino acids	33110	30565	18318	25427	30323	20292	26784	32303	26260	29503	46058	34597	50888	34845	21886	4827
MALEIC ACID	13.448	115.00446	Dicarboxylic acids and derivatives	51529	47700	64597	48986	33968	33222	55804	52888	43640	54556	43970	34872	53291	41035	49963	67103
Mebeverine	0.913	430.24429	P-methoxybenzoic acids and derivatives	303755	442246	483027	625275	491318	486623	531563	494382	207809	190515	204055	250470	378332	349816	326395	1413612
Meglutol	11.992	161.04601	Hydroxy fatty acids	20251	19304	15301	14874	13506	16543	22995	11125	10093	13515	14209	12765	10193	12021	9316	13179
Melibiose	13.419	365.10413	O-glycosyl compounds	828	1275	13534	798	536	2000	2311	178	451	32331	646	40498	363	798	402	368
Mesaconic acid	13.235	129.01936	Methyl-branched fatty acids	72496	73301	85326	60059	66850	83211	96877	68289	49156	97693	83501	50946	63412	79626	51591	32113
Mesaconine	7.986	508.26712	Aconitane-type diterpenoid alkaloids	4661	4490	5324	6951	1224	3787	14379	5832	8755	4400	5354	31924	7981	3081	5652	37916
Metformin	1.15	130.12424	Biguanides	33479	55482	57924	118116	70916	90835	102302	62850	19749	41611	20027	29651	20917	34801	45203	101952
Methanesulfonate	14.583	94.98116	Organosulfonic acids	34131	28730	20177	25849	30196	23802	24438	25885	26943	25916	23971	26061	25832	28992	25732	35813
Methionine	10.485	148.04272	Methionine and derivatives	129379	95129	124271	81766	65077	112922	86753	48844	67983	181555	87158	100054	59130	49667	60617	98137
Methioninesulfoxide	13.547	166.05138	Alpha amino acids	49769	42129	48208	36570	52772	52643	40177	25117	50681	70382	33391	43142	23354	40871	31156	12586

methyl 1-methyl-9H-beta-carbolin-7-yl ether	13.725	235.0901	Harmala alkaloids	76384	106359	209320	97015	91731	558726	386009	371713	75438	41682	225006	64614	312574	710917	119424	99504
Methyl 3,4,5-trimethoxycinnamate	13.559	291.00784	Coumaric acids and derivatives	3989	3972	2641	2707	3857	4435	2921	3281	4442	3900	4103	4123	4394	4284	3892	17659
methyl chlorogenate	11.062	367.1095		628380	584903	273532	227589	767891	210507	341186	104038	464754	341605	973985	575904	178849	395107	209921	414481
Methyl Heptadecanoic acid	1.259	283.26498	Fatty acid methyl esters	87695	100750	68494	97371	157826	99894	133362	168077	119261	142067	82832	95554	154831	98579	95459	284017
methyl orsellinate	9.557	200.0912	p-Hydroxybenzoic acid alkyl esters	52405	27655	44008	33169	39252	23284	17901	21183	98072	7238	18890	36994	12819	24339	19890	6838
Methylacetate	12.437	73.03059	Methyl esters	11863	12044	18538	10201	11086	10743	20895	12127	10974	27876	13468	13783	10503	18067	12978	23272
Microcolin A	1.298	770.55273		10649	14339	17105	9243	17075	9333	20353	8526	5221	10490	11251	8979	11328	10458	11318	3803
Microcolin E	8.484	752.51263		11211	5553	10419	14963	13924	4665	10530	10227	8454	5982	6728	6260	1751	2421	7764	2259
Microcolin H	6.297	756.54779		12130	12595	7905	8169	8898	5730	8785	7677	10679	7918	7409	7738	13236	7666	6648	13648
Miltirone	1.318	305.15454	Tanshinones, isotanshinones, and derivatives	1617	2051	1727	10101	5410	6801	613	3364	7290	3388	4854	2127	9392	7578	40819	9385
Mirificin	13.706	571.12103	Isoflavonoid C-glycosides	1632	2854	1776	2553	1892	4537	2657	3640	1229	1411	4874	2516	3880	4433	3095	2713
Monensin sodium salt	7.424	694.3952	Ketals	37436	39376	40336	69540	42235	38049	56431	36553	48619	53131	53275	51623	14123	27482	63000	5839
Morpholine	9.126	88.07454	Morpholines	58174	153286	158748	81427	48815	162067	97267	127080	124765	181088	50303	140794	155786	150472	117352	109836
myricitrin	10.859	503.05344	Flavonoid-3-O-glycosides	2902	2672	4370	3582	894	1582	3955	3615	3252	8084	1678	1206	6290	1675	3124	3453
Myrislignan	7.373	375.18683	Lignans, neolignans and related compounds	12103	81196	78887	85100	32441	83082	102721	82272	5694	108637	81088	102669	67002	67806	107198	1133
Myxovirescin A	1.3	624.44043	Macrolide lactams	1472	2517	1279	1674	4278	2830	1627	2273	692	216	517	326	3182	1510	962	8332
Myxoxanthophyll	1.321	730.50909	Xanthophylls	22529	25418	12644	21510	16425	43815	20775	19356	24099	24618	24347	23513	27966	28976	28355	6777
N-(3-Methoxybenzyl)oleamide	6.368	424.34229	Anisoles	167168	14172	7063	24362	5271	127920	2247	8586	14861	11733	71763	5692	233068	13392	10402	107630
N-(9-oxodecyl)acetamide	12.123	236.15172	Acetamides	6626	3587	1896	1838	4436	2260	2917	1747	2188	6368	3496	1780	2101	24001	3307	3470
N,N-dimethyl-7H-purin-6-amine	10.217	186.08632	6-alkylaminopurines	72750	19782	7703	10524	11530	54348	10322	12135	23596	17735	5997	9124	23179	142784	14002	36619
N,N-Dimethylaniline	2.91	122.09647	Dialkylarylamines	115714	142098	225739	90285	99974	131287	100854	174676	58092	66494	75759	73884	105257	94403	84905	129137
N,N-Dimethylarginine	15.874	203.14729	NA	44202	55342	70676	41962	50976	61222	94419	55785	55870	124948	63486	65898	76238	99636	58681	31179
N4-Acetylsulfamethoxazole	13.8	296.06253	Benzenesulfonamides	21078	27620	35477	24472	18245	14454	35558	35755	51067	101236	48317	44292	37764	31518	40123	16157
N-Acetylaspartic acid	13.174	174.04291	Aspartic acid and derivatives	79268	64398	72262	68431	69592	53879	87574	81681	58134	60680	120583	71452	43278	57808	86023	43891
N-Acetylaspartylglutamic acid	12.342	303.08478	Dipeptides	15664	1998	2284	8354	40437	4150	7378	3896	17453	608	15919	2218	861	2843	5909	13012
N-ACETYL-DL-SERINE	9.452	146.04694	N-acyl-alpha amino acids	15965	18896	11731	24358	16063	19354	29186	15788	16567	23247	17707	16958	14493	20280	17891	20694
N-Acetylglutamic acid	12.97	188.05626	Glutamic acid and derivatives	62837	51965	71729	53622	45428	34567	46071	26976	35837	108724	52837	43168	23351	45481	60188	46818
N-Acetylglycine	7.5	116.03801	N-acyl-alpha amino acids	20573	12867	14773	12363	15194	8220	21007	12214	10752	6680	9547	7956	10681	7526	10199	40614
N-Acetylneuraminic acid	12.694	310.11423	N-acylneuraminic acids	28106	14323	56355	7474	40504	6722	12111	22758	46470	64892	58007	44995	35822	19475	50111	19345
N-acetyl-O-methyltyrosine	13.452	236.07762	Phenylalanine and derivatives	64926	33355	23594	16854	28591	37136	21498	28958	30609	16632	58882	8020	26819	32805	18742	41478
N-Acetyl-ornithine	13.377	175.10666	N-acyl-L-alpha-amino acids	101844	208384	439172	131167	110376	175230	253935	61965	121551	81820	183798	70219	122822	214572	52753	63955
NALOXONE HYDROCHLORIDE	12.239	386.11075	Phenanthrenes and derivatives	8470	2076	3567	9580	21168	6245	9322	5572	9254	618	9252	4076	1253	52	2210	908
N-alpha-Acetyl-L-ornithine	13.35	173.09399	N-acyl-L-alpha-amino acids	145686	295095	452329	192524	186229	165632	294305	71385	133064	69938	223858	73009	138069	230606	60434	23506
N-Benzyloleamide	6.291	372.31238	N-acyl amines	14701	4445	3270	2000	3431	14025	1879	8162	17163	10596	7289	4189	25755	4858	5543	167
N-Benzyloxycarbonylglycine	7.234	208.06331	Benzyloxycarbonyls	31991	45212	858	26256	49654	443	480	20989	19347	17991	27107	22180	41756	12851	18262	717
Nelfinavir	7.593	566.31757	Alpha amino acid amides	47716	68378	40047	81715	59066	59311	76011	27131	38296	54128	60470	51812	54431	44356	65954	484
Nervonic Acid	1.17	367.34088	Very long-chain fatty acids	12659	17658	14927	24295	26862	25031	22117	23707	13293	10774	5879	12972	9132	13114	16447	30017
N-Fructosyl pyroglutamate	11.986	290.08838	N-Fructosyl amino acids	30676	42864	35151	61448	31470	33333	78919	38805	21180	54473	29359	149858	44911	29419	27814	29502

Nicotinamide	1.294	123.05547	Nicotinamides	364005	321333	201997	299788	388935	533870	275539	341151	540655	498115	585069	492435	986712	716915	520215	8942469
N-Isovaleroylglycine	6.53	158.08176	N-acyl-alpha amino acids	42358	36217	43169	75540	96793	33500	48721	32101	19742	19560	58028	34868	17850	12505	15409	23858
Nitrazepam	1.273	282.08972	1,4-benzodiazepines	45897	27245	20233	217	0	294	520	23420	45	58	97	158	158	51	517	220
Nizatidine	10.411	330.12616	2,4-disubstituted thiazoles	6479	23541	19557	7076	9240	9292	9100	8011	4356	6536	4591	15486	13402	3637	5197	4657
n-methyl-2,4-dihydroxy-3-phenylquinoline	12.905	252.10504	Phenylquinolines	13139	5666	5148	4612	3738	8745	5097	4959	2086	2921	16155	1830	5902	18672	3807	2838
N-Methylcytisine	14.003	227.11182	Cytisine and derivatives	100059	81009	65820	67072	164612	58611	122195	152415	95782	235628	237571	149548	109021	173005	121302	473
N-Oleoylethanolamine	1.135	326.3063	N-acylethanolamines	4306	8707	5855	5521	3986	9208	7497	7298	2968	6579	4553	4161	6850	8095	5276	20703
Nonic Acid	14.935	226.98833	Medium-chain fatty acids	375910	385547	402718	338722	368341	407312	373020	406290	366515	462682	410088	347744	421102	476822	396038	317360
Norleucine	10.186	132.10118	L-alpha-amino acids	565807	567439	619906	545338	666982	668632	608722	441365	615396	926301	577668	683478	457111	870137	464859	414183
Norvaline betaine	1.447	160.13327	NA	35409	16733	37311	73548	52855	13074	39091	17593	37640	9787	45707	13676	33300	28809	42242	4299
O-Acetyl-L-carnitine	11.37	204.12117	Acyl carnitines	284301	922817	1369646	1460897	1586954	861801	452463	800463	1736444	731126	910745	168758	984396	1134876	1051002	51884
Obtusaquinone	11.679	253.09639	P-quinomethanes	12513	10647	27337	45509	19263	12993	24687	7715	34506	8226	30825	19449	19256	8881	12234	8448
Octadecanedioic acid	4.171	313.23917	Long-chain fatty acids	6428	4356	15825	12830	5225	10036	8797	1182	7255	19146	3409	8580	2732	3678	849	3662
Oleamide	1.108	282.27942	Fatty amides	12235	18463	19764	26471	16376	48002	38046	21913	11113	20445	16053	14605	31471	54312	24510	66898
OLEANDOMYCIN PHOSPHATE	6.019	688.45306	Aminoglycosides	12728	4717	10462	9730	14906	20073	12245	15048	17477	1575	11635	13201	17817	3742	3755	174239
Oleandrin	7.455	599.32758	Cardenolide glycosides and derivatives	20136	25175	26999	11872	15323	18169	15394	17570	23855	21105	20113	22009	29616	27289	14336	15493
Oleanonic acid	7.944	455.33426	Triterpenoids	11432	8684	10401	9798	7303	4534	10496	9110	7571	7417	11674	11325	5432	8710	8455	3043
O-PHOSPHO-L-SERINE	13.61	184.00108	Alpha amino acids	4067	4996	28361	3564	4175	4710	11473	4826	3308	6024	12513	9527	3422	4321	4475	3097
OPHTHALMIC ACID	7.251	288.11194	Peptoid-peptide hybrids	35838	2802	455	9783	80366	2211	1990	5144	38379	551	9229	2732	367	14507	2710	985
Ornithine	15.874	133.09526	Alpha amino acids	41084	32409	18224	24603	17720	59151	77258	43981	32405	68472	83410	65652	54016	42793	46518	18151
ORTH0-AMINOBENZOIC ACID	1.099	136.04239	Aminobenzoic acids	15179	19770	29868	18173	20902	8430	18341	14473	15614	18823	20639	23362	8734	5998	17640	7302
Orthanilic acid	1.326	172.00992	Benzenesulfonic acids and derivatives	4179	18136	13743	22717	2633	2583	4156	15181	7869	39539	8541	21174	19850	14636	34514	19512
Oseltamivir carboxylate	9.828	283.16751	Gamma amino acids and derivatives	32626	44785	71946	37563	69097	66097	50087	56174	27682	62354	30586	47248	44300	65015	22220	29179
Oxaprozin	11.465	294.10489	Phenyl-1,3-oxazoles	2418	8240	4757	15795	3794	9753	21725	1646	4929	17439	11752	13044	9308	9699	11987	7267
Oxidized glutathione	13.938	611.15369	Peptides	7303	6668	3385	15904	7879	16878	4196	4548	21943	3072	1381	8245	25323	6290	2775	2950
Oxysophocarpine	12.111	285.15277	Matrine alkaloids	19671	9311	3555	2823	7709	24627	3140	9137	17563	2378	5282	1440	4872	48916	13202	3041
PAF (Platelet Activating Factor)	7.717	524.40942	1-alkyl,2-acetylglycero-3-phosphocholines	19994	18628	8535	22244	30582	25999	12259	22338	18028	13967	15460	12809	19538	20845	12868	26430
Palmitoylcarnitine	6.44	400.3418		13787	14071	8417	13759	9914	16762	5067	19475	35462	21187	19020	11872	31514	25733	40598	81034
Palmyramide A	1.347	672.41956	Cyclic depsipeptides	850	4985	18049	5474	40754	10421	24986	13375	2721	29485	956	6483	333	20947	14772	2936
Pangamic Acid Sodium	13.621	302.08911	Alpha amino acid esters	3386	2715	1008	4108	6522	4317	2926	4145	7815	561	3541	956	4894	2204	4930	6523
Pannaric acid	16.785	315.04153		297895	564405	260850	374351	211005	320405	502554	566285	244060	464504	150750	147216	289289	294160	399349	543955
Pantethine	7.584	572.29565	Beta amino acids and derivatives	17995	12613	19741	39577	19443	18013	22575	40977	10034	19003	25836	47541	13254	13989	23788	838
Pantothenate	7.366	220.11513	Secondary alcohols	65962	95944	75	44113	39901	87705	90583	81013	30970	80087	56281	54428	150488	138094	57545	678
Paxisterol_120160	0.992	443.31192	Ergostane steroids	3795	3746	4966	3192	4635	7168	4443	7456	7397	1854	1263	4881	6757	2361	6747	27080
PC(14:0/10-HDoHE)	5.345	852.54694		19718	42976	65824	91387	60217	41302	83903	38072	14119	20544	38024	13567	18785	3376	72013	199
PC(15:0/12-HEPE)	5.96	840.51294		4687	7375	11985	19985	10739	3411	13315	5614	5374	2940	2357	6335	1391	2563	2551	70
PC(15:0/12-HETE)	1.311	842.53284		9341	6311	6021	9043	6743	7987	8453	5428	10429	5826	7215	6325	6350	8566	8444	1621
PC(17:0/12-HETE)	1.311	870.57538		6377	4702	4618	4837	5132	4415	5090	5182	6344	2925	3689	5419	4391	5225	3865	4311

PC(17:0/9-HODE)	1.321	846.55566		84320	47015	55169	56055	43885	35150	70379	50008	56049	33867	29153	49417	31072	23479	47718	3399
PC(17:1/13-HODE)	6.153	844.54688		6651	5471	3332	6669	9031	6853	7154	4834	6157	3032	7185	6345	6488	6405	6229	11015
PC(17:1/18-HETE)	1.314	868.57703		3709	2082	2444	4118	4749	4063	3396	2776	3831	2810	3534	3392	4895	6288	4418	11693
PC(18:1/13-HODE)	1.32	858.56793		8808	7714	7593	7268	8438	8428	9388	6672	7825	6324	5864	8799	6072	7619	7570	925
PC(18:1e/12-HETE)	1.339	868.60522		2826	2205	1654	3004	4894	5738	2910	2782	4104	1775	5280	2611	5227	7167	4102	9620
PE(18:0/10-HDoHE)	6.436	806.51685		15653	11741	11930	13705	8287	6068	12903	7746	9400	8727	4971	9408	6703	2803	6164	224
PE(18:1/9-HODE)	1.316	756.51031		12965	10874	7719	13949	13403	10836	15298	9590	16562	10565	9666	10144	7310	13085	14358	2456
Pellasoren A	7.378	432.30734	Delta valerolactones	3143	730	57936	379	732	57939	25721	401	188	87759	54154	34640	1074	1314	30151	363
Pelletierine Hydrochloride	10.051	176.09615	Piperidines	25042	39928	29296	24998	21359	70244	12551	36638	25295	17800	24025	20634	49995	16332	29086	1477
Pentadecafluoroheptan-1-ol	13.855	384.99954	PFSA	155905	175126	92085	171559	138845	53362	90563	87626	228053	123851	61330	167450	138094	91263	117309	181227
Perindopril	15.039	369.24567	Dipeptides	91213	69986	76653	78593	103410	71910	86516	92818	97800	150173	129666	95494	88738	106776	60333	1229
Pestalotin	7.273	215.13864	Dihydropyranones	101341	20128	401	68242	43860	9874	926	83235	92786	6808	11697	25674	107307	10107	92217	2958
PFAP-FT_diPAP	14.893	938.98737	PFSA	4153	4513	3068	1515	5489	6917	4095	4311	2616	2702	3135	1444	2221	2812	2603	3222
PFAP-FT_PAP	13.493	376.97754	PFSA	5178	5251	5320	7710	8208	5988	7314	6173	6357	5132	7124	6849	5037	7146	8862	6026
PFAP-N_PFSE_disubstituted	14.8	902.97766	PFSA	93221	99838	51743	93795	25175	37107	104912	55326	91516	83129	49716	58088	89978	27585	46559	66510
PFCA-diether_Hsubstituted	14.944	696.97473	PFSA	23710	9861	19187	28980	13760	26325	20354	25702	6938	15587	15502	15605	28831	36143	15372	43509
PFCA-H	14.8	223.01968	PFSA	787755	1097111	918583	1563284	586159	1666469	1335944	1874721	1584185	1064070	1024271	1260752	1642672	1070779	1707885	1848415
PFCA-pentafluorosulfide	13.864	520.93109	PFSA	26103	38283	25951	31364	25520	15136	27708	29603	34574	33933	22217	35181	29475	22834	29680	38688
PFCA-perfluoroalkyl_branched	14.69	762.96057	PFSA	21371	14499	12198	18441	18067	22773	26802	25258	17433	17746	17644	19635	22741	21982	22354	31570
PFCA-perfluoroalkyl_dioic_acid	14.993	638.94983	PFSA	8486	5296	7797	5021	7849	5583	5343	6637	3252	4378	5038	2802	5443	11124	4419	8664
PFCA-unsaturated	13.807	774.92883	PFSA	4984	9061	9076	6768	5448	8740	10686	7357	4352	8970	11143	9511	6751	11650	5949	4643
PFCA-unsaturated_ether	14.811	1090.95654	PFSA	6161	7725	5004	9238	9655	5403	11343	10326	3241	4153	8923	6348	21508	13502	8724	3367
PFOH-perfluoroalkyl_alcohol	13.81	734.97595	PFSA	30887	44900	41654	43239	37844	42800	36312	34855	26428	47598	44531	37626	37875	50071	31035	23795
PFOH-unsaturated	14.942	802.96344	PFSA	6185	8755	5356	8485	5236	6599	11337	8861	5200	6022	7851	8694	7058	9410	9017	8583
PFSA-unsaturated	14.216	310.93961	PFSA	138413	213141	84664	233391	135532	75240	199803	120696	91283	207989	205132	203444	274418	154364	85523	83115
PFSM-amine	14.8	633.03339	PFSA	1657601	1703982	1848781	1418879	1764421	1597104	2025135	1660807	1274224	1964645	1871767	1772698	1403822	2342328	1275966	1829005
PFSM-ammonio	13.788	714.04614	PFSA	8191	6545	3408	4792	6816	3708	4599	8181	6257	5446	5339	7035	5411	10758	4285	20210
PFSM-perfluoroalkyl_sulfonamide_Me	13.849	561.95947	PFSA	16801	16245	20572	17182	17918	13862	14107	24788	15072	13527	16571	17315	18887	9333	24759	4178
PFSM-sulfonic_acid	13.833	869.9408	PFSA	3622	3298	3880	4365	3979	1452	3677	5893	3965	3305	2686	3854	3055	2208	5976	748
Phenacylamine Hydrochloride	11.927	170.04723	Alkyl-phenylketones	12203	10598	4776	3833	2222	3795	2653	7841	8945	903	2412	932	9203	65396	9017	48305
PHENATURIC ACID	1.026	192.06917	N-acyl-alpha amino acids	13643	9467	11249	17112	21820	11336	19692	9400	17068	11441	15354	13622	8588	8063	13897	10546
Phenylacetylglycine	4.584	192.06903	N-acyl-alpha amino acids	586391	510318	1253575	681229	629829	1059407	277842	188606	713243	394081	516742	415616	302098	244741	342194	55901
Phenylalanine	9.643	166.08656	Phenylalanine and derivatives	1660679	1689681	1562676	811149	1496788	2037781	1724186	951062	1410857	2218728	2283632	1178505	1160219	1477650	1272282	705914
Phillygenin	1.005	373.17819	Furanoid lignans	2730	11780	4202	9119	5408	25118	13064	3907	7684	11294	3180	15158	10052	21595	9033	9843
Phorbol	1.095	365.19531	Tigliane and ingenane diterpenoids	9193	17702	13799	22904	9694	37020	28579	18074	7475	26399	5979	24428	14459	26570	23233	21870
Phosphatidylcholine 15	6.24	822.56903	Phosphatidylcholines	9114	5156	11015	17580	10981	4869	10957	10290	9256	4664	10430	5746	2752	7991	9367	2837
Phosphatidylcholine 16	1.349	848.58032	Phosphatidylcholines	26775	16917	33996	12133	24298	11164	53407	18019	11764	24241	4142	10763	9094	8401	14686	1710
Phosphatidylcholine 18	1.315	816.59473	Phosphatidylcholines	15931	9701	10855	13766	13855	14743	18422	8504	15984	11543	7896	9161	12594	9914	10146	7258

Phosphatidylcholine alkenyl 16	6.24	820.57257	1-(1Z-alkenyl),2-acyl-glycerophosphocholines	13855	11405	15769	20255	17726	8770	18809	13793	14594	7994	14624	10109	8102	10067	12256	5936
Phosphatidylcholine lyso 17	7.949	540.36902	2-acyl-sn-glycero-3-phosphocholines	19098	18791	11456	13615	17359	18668	14608	16706	21186	17501	15504	23528	21147	49456	17635	7923
Phosphatidylcholine lyso 19	7.833	568.40271	2-acyl-sn-glycero-3-phosphocholines	6121	8434	6790	7255	8041	6745	7323	10204	10553	6273	7224	11572	8394	17190	6745	3215
Phosphatidylcholine lyso 22	7.265	598.35352	2-acyl-sn-glycero-3-phosphocholines	6388	4068	113	6815	3727	964	408	1138	7294	5612	3070	6084	6502	3465	3159	50
Phosphatidylethanolamine 16	1.322	762.51538	Phosphatidylethanolamines	2277	1606	1096	1428	1511	1915	2349	958	1883	1092	669	1705	1553	1432	785	12018
Phosphatidylethanolamine 17	5.152	774.50165	Phosphatidylethanolamines	17611	18018	12155	25654	9166	3711	14150	2651	15092	7586	16618	9777	4477	6610	17552	121
Phosphatidylethanolamine 18	5.286	794.54041	Phosphatidylethanolamines	5744	2375	2408	13022	2135	2314	2647	2162	3755	3604	4615	3153	4735	1186	3484	470
Phosphatidylethanolamine 19	6.667	802.5434	Phosphatidylethanolamines	7342	3756	5044	3671	6991	11207	9130	2781	9695	4826	8321	7279	3751	2806	6974	230
Phosphatidylethanolamine 20	1.319	814.53387	Phosphatidylethanolamines	31481	13598	18669	25824	20372	15660	25172	25150	30750	9824	18044	16712	13701	22513	21607	1191
Phosphatidylethanolamine alkenyl 16	5.943	746.50409	1-(1Z-alkenyl),2-acylglycerophosphoethanolamines	8849	7043	3253	5702	2030	7083	1982	1258	1633	699	3493	4110	2468	1699	4248	65
Phosphatidylethanolamine alkenyl 18	1.292	770.53613	1-(1Z-alkenyl),2-acylglycerophosphoethanolamines	16262	5018	10372	22226	10906	6944	15372	10141	14809	14569	18657	11508	10878	9809	11840	855
Phosphatidylethanolamine alkenyl 20	1.295	800.54321	1-(1Z-alkenyl),2-acylglycerophosphoethanolamines	6522	4375	4601	9804	6866	5320	8668	5182	7647	4509	6023	5172	2964	4773	7150	359
Phosphatidylethanolamine lyso 20	7.752	506.32373	2-acyl-sn-glycero-3-phosphoethanolamines	5226	3619	3352	3434	4526	4214	3858	3712	4156	3494	4022	4246	4690	7420	3676	1648
Phosphatidylethanolamine lyso alkenyl 16	7.736	436.28751	Glycerophosphoethanolamines	3678	4856	1726	3555	5091	4225	3252	2305	4212	6457	5278	3679	6144	6542	2759	2456
Phosphatidylethanolamine lyso alkenyl 18	6.712	462.2803	Glycerophosphoethanolamines	3278	3752	40587	2912	1235	4098	432	996	133	2226	5587	1525	1076	1897	2072	535
Phosphatidylinositol lyso 18	7.441	599.31732	2-acyl-sn-glycerol-3-phosphoinositols	486747	382731	175072	326727	323341	131279	239202	313624	442011	129075	357062	359644	270867	411774	298304	5343
Phosphatidylserine 18	7.165	786.53363	Phosphatidylserines	3451	49408	1639	117326	107281	54687	104622	77749	80304	81718	97328	63979	74201	109280	106959	574
Phosphatidylserine 19	1.329	848.5274	Phosphatidylserines	13554	9476	13718	7419	11424	6643	15811	7847	8180	10910	2968	7089	4964	5549	9011	685
Phosphotyrosine	9.723	260.02783	Phenylalanine and derivatives	1288	623	7134	647	684	16980	297	14537	1707	45158	2400	1188	2361	1908	11792	86537
Phthalic anhydride	0.892	149.02298	Phthalic anhydrides	284709	364581	362883	404572	382384	415544	418414	395234	241410	249182	228052	252024	358913	313567	307199	536255
Physcion	13.115	283.11145	Anthraquinones	49391	62422	38217	51496	37587	35542	64776	29968	65409	49571	52144	47540	52233	65285	52510	23269
phytic acid	13.942	682.86981	Inositol phosphates	2299	1625	1766	2354	2406	424	492	2964	3317	1458	406	1307	1808	2918	4272	702
Pleiokomenine A	0.997	717.43036		4211	19828	4417	24214	3355	7434	17617	2780	4240	13012	3685	11672	3228	5349	4810	2999
Podototarin	6.789	609.39435	Diterpenoids	2091	2066	40751	2905	18231	2404	58957	8066	6526	18486	3107	14314	2169	10907	13597	1099
Polyphyllin A	7.304	599.33508	Steroidal saponins	9424	20132	33	10237	7644	16530	116	9601	1770	252	702	10442	12969	13957	5681	912
Polyporenic acid C	8.278	483.33728	Monohydroxy bile acids, alcohols and derivatives	18939	34680	36387	11337	8315	21343	4202	10298	1200	17517	13110	9281	20536	35006	5590	16159
Procaine	1.272	237.15916	Others	8617	7945	7627	3051	5473	3948	4038	7873	5260	3674	8856	6555	22183	6813	8206	41958
Proline	11.278	116.06963	Proline and derivatives	1375717	1843883	1803657	1583302	1813402	2084012	1830313	1284316	1746639	2806336	2037362	2025711	1323087	1891568	1678460	1263715
Prometon	1.106	226.17969	2-methoxy-1,3,5-triazines	2057	2578	5483	7902	4369	5587	6834	9698	1373	9185	1870	5332	6960	2992	5786	51380
Propionylcarnitine	10.596	218.13693	Acyl carnitines	26769	64590	126244	51721	44213	115330	27833	88153	92179	317433	100691	127939	53427	117050	38600	67171
PROTOVERATRINE A	7.169	816.49609	Cerveratrum-type alkaloids	59182	77904	3708	85708	56645	3071	1299	65575	87720	2974	5558	90035	32853	56473	109746	2470
PS(16:0/11,12-EET)	1.305	798.51862		12718	8912	8079	17250	10365	10325	15652	9962	15397	7829	9432	11377	5136	8272	12544	345
PS(18:0/14,15-EpETE)	6.671	824.5274		58610	64767	54021	52357	29120	17377	39247	27925	52062	20911	17494	46668	22561	8576	25607	422
PS(18:1/16,17-EpDPE)	6.397	848.5108		10604	8587	11613	9572	5799	4090	9323	7458	7531	4695	6129	7455	4522	4702	6598	164
PS(18:1/9-HODE)	6.606	800.50897		13200	22417	25288	35697	14195	26940	5987	8798	33491	4455	29289	15607	5958	11209	25179	201
Pseudouridine	8.542	243.06454	Nucleoside and nucleotide analogues	80953	111520	105065	50863	59068	73403	87175	38344	44513	59400	57124	53710	66058	79974	45297	26014
Putative Peptaibol Trichodermide D analogue	7.716	1161.76392	Polypeptides	17645	15299	24864	22077	8353	9166	21755	26073	16787	11167	16007	21550	11952	19681	24415	3637
PUTRESCINE DIHYDROCHLORIDE	1.016	178.08426	Monoalkylamines	2831	6389	4048	7096	6749	8103	13151	4730	3405	5385	3390	5257	4246	3177	4947	8454

PYRIDOXINE	13.583	168.02762	Pyridoxines	16804	15019	7665	16492	14129	13391	11279	14640	14462	12549	12000	12556	13543	15655	15177	16625
Pyroglutamic acid	9.795	128.03484	Alpha amino acids and derivatives	1535228	1699976	970112	1856038	958022	1292453	1388175	1209769	2080587	1711149	2706341	709253	836607	1595359	3762459	3667658
Pyrrolidine	11.047	72.07996	Pyrrolidines	436405	266576	239989	296067	357750	349206	248541	259695	332287	387560	311918	331767	349839	229945	263358	257337
Pyrrolnitrin	14.39	254.96828	Phenylpyrroles	754056	737070	677349	808120	722245	845076	509614	773431	832385	672433	753672	726170	816788	651542	882557	672950
Pyruvic acid	1.339	87.00921	Alpha-keto acids and derivatives	186783	248893	284759	344365	187523	73772	233216	246284	173183	252403	224488	502084	357556	308587	452881	417144
quercetin 3-O-glucuronide	10.882	501.06113	Flavonoid-3-O-glucuronides	17600	22475	29426	25995	9351	13487	26786	22359	17968	38900	11861	10479	25519	12219	13665	29845
Quercetin 3-O-malonylglucoside	14.393	548.89795	Flavonoid-3-O-glycosides	522182	470443	478508	603836	488631	605435	562259	543265	635794	427720	511904	482425	587557	442084	662650	465607
Quillaic acid	8.27	509.33456	Triterpenoids	7140	6387	18081	6130	12981	6357	18762	9527	6129	7995	1094	8219	1319	7067	11343	271
rabelomycin	10.796	337.07593	Angucyclines	4787	31900	25109	24583	14885	10616	19684	14345	33538	10081	18004	25216	32461	22889	33757	38126
Ranitidine	1.332	313.12692	Aralkylamines	4254	6675	4541	6315	9745	3572	7473	7093	4301	5604	6992	7959	6838	3378	6982	45940
reynosin	13.07	249.15446	Eudesmanolides, secoeudesmanolides, and derivatives	9894	2932	3962	1823	2431	1556	2170	2372	4014	5846	12117	3322	5038	3832	3700	15795
Roccellic Acid	9.247	623.40997	Long-chain fatty acids	9314	3482	4342	26610	8493	1734	7542	5134	8398	3743	17499	13895	3866	6717	5453	5249
Rofecoxib	13.681	313.05414	Stilbenes	4447281	3884297	3078770	4029596	4718158	3229386	3562319	3733693	4060656	3277796	2973064	3447525	4292701	3487805	4112621	2325195
Roquefortine C	1.114	390.27768	Pyrroloindoles	4844	14929	11158	23372	24360	10599	46178	8525	3586	18636	9678	6009	3590	5881	6543	2933
Roseotoxin A	6.199	608.38971	Cyclic depsipeptides	6638	10020	19284	6352	18125	14959	13617	7273	7851	5208	12828	15453	11403	5592	9683	3833
Rosmarinic acid	10.974	743.15277	Coumaric acids and derivatives	7152	9559	13354	8498	4897	6235	13103	10209	7413	22083	3292	1783	8971	4069	7317	3865
Royal jelly acid	0.957	187.12817	Medium-chain hydroxy acids and derivatives	10323	15651	13372	18927	18526	23901	27946	15898	8598	9435	6623	8801	14299	16488	10423	47722
Rutaecarpine	11.616	288.09988	Beta carbolines	5267	7069	5978	4833	2647	4987	5359	6151	8663	24126	7742	12724	6610	10410	11437	4531
Salazinic acid	14.803	387.02457		6693302	6974249	6716033	5966405	4769912	6363743	5493895	6641333	6079466	8038774	4749523	7278329	6579855	3694346	6417045	6966934
Salvianolic acid F	13.651	337.04749	Stilbenes	44501	37220	17397	35471	23627	35443	29385	34957	37157	32527	25122	31102	47242	42609	35285	53597
Scabioside C	1.055	789.46301	Triterpenoids	9264	29255	11724	49134	20847	101260	52018	9401	19274	117127	10483	63815	11862	38018	25482	10483
Scopularide B / Arenamide B	4.22	644.39099	Cyclic depsipeptides	6315	1522	1087	2515	2012	3232	1681	551	3751	688	2399	1270	1161	809	989	1658
Scopularide F	5.537	728.47992		4598	6190	1401	4079	7855	13979	4117	1635	5407	1977	5298	4628	44862	5096	7496	19442
Scopularide H	1.341	700.45386		13875	24734	12445	27189	54188	47364	26985	26212	22304	14303	6878	20066	10183	24733	23195	1708
Scytonemin A	7.938	732.39111	Hybrid peptides	29428	27855	36424	34941	23775	18270	31261	42685	22499	22771	33911	36661	16936	24952	27320	14322
Scytophycin B	7.301	802.52063	Diterpene lactones	3179	3441	75831	9058	3021	72102	133495	89398	4170	76971	70767	52886	4283	6216	78015	1120
Scytophycin C	1.317	828.52905	Diterpene lactones	223247	250230	319625	230401	231924	223916	329569	237613	199078	327802	163090	251382	217561	210023	264548	18982
Scytophycin D	1.326	844.5238	Diterpene lactones	40227	70588	149204	73920	87356	44792	173619	85239	43654	162629	30712	82259	38844	69375	95848	12131
Scytophycin E	6.642	804.53021	Diterpene lactones	37623	33583	31434	30769	25571	22974	28805	18751	30676	6403	22925	17325	18273	8582	17113	43967
sebacic acid	12.966	203.13927	Medium-chain fatty acids	120178	242859	151868	98737	130738	176158	158022	228658	11891	151275	98818	21153	200007	52213	23650	205712
Sec-O-glucosylhamaudol	13.646	461.13153	Pyranochromenes	80601	72536	50879	76764	88788	73176	77358	68932	60982	84259	87885	71636	108308	101964	78429	51228
securinine	7.313	240.09711	Indolizidines	24604	74041	56	38542	36666	451	315	65947	59190	149586	57973	68334	67157	187773	88214	1829
Sennoside B	13.71	861.19177	Anthracenecarboxylic acids	2906	3092	2715	3874	2818	9884	3669	7071	3348	1414	4053	1194	6869	5295	2431	69
Sinapinic acid	7.599	223.08069	Hydroxycinnamic acids	17128	15928	5628	18418	22333	31566	13091	9082	18037	14232	16858	19779	4783	7008	9569	6075
Sinomenine	7.391	328.15231	Morphinans	6320	10455	709	5685	5875	3682	5053	7311	5554	4531	7836	5531	7011	7738	6523	1667
skimmianine	13.442	262.12512	Dihydrofuranoquinolines	11409	12142	13812	15459	16041	19813	13147	18087	36252	38697	30011	20884	16156	21120	16783	8680
skimmin	10.508	323.08185	Coumarin glycosides	5211	21321	25926	7273	13723	34331	37388	9481	18627	17959	25580	11632	12135	7491	11870	9710
Smenospongiarine	7.363	414.29398	Prenylquinones	288	582	47067	676	597	41486	18214	34492	122	58704	43773	21456	439	1272	20099	181

Smenospongine	14.747	268.99762	Prenylquinones	2377768	3686323	1673142	1880861	3126778	2618958	1542809	1443055	3043729	2894101	2968562	2047554	2594563	3857739	3061476	3995398
sn-Glycero-3-phosphocholine	13.797	258.10919	Glycerophosphocholines	123823	103693	83680	120153	100450	62775	105133	140960	183474	224679	110687	110928	142155	171288	124830	104700
Sodium Deoxycholate	1.048	393.28558	Dihydroxy bile acids, alcohols and derivatives	1766090	3802613	1856596	3001311	2302802	3926101	4325058	2283837	2001755	3772312	1302798	2985032	2347943	2984200	2575461	6691584
Solanidine base + O-Hex-dHex	7.021	706.45715	Steroidal saponins	9762	7372	16753	24181	32327	20359	25142	13445	8879	12472	16278	8662	5804	4728	13367	18481
Solorinin	11.03	296.13803		465598	528550	645441	344768	642938	911518	588685	442325	437880	918291	580324	1082329	485236	627829	389050	919616
Sorbitol	10.398	181.07222	Sugar alcohols	166259	329086	356618	197631	94458	207740	195271	394503	54898	71051	63920	196654	809088	50791	123871	72075
Sotalol	13.048	271.10324	Sulfanilides	37920	37785	28442	42493	37598	34919	39919	25166	34287	39839	28594	40101	29632	18526	32618	33540
Soy bean phospholipid	0.925	678.48956	Phosphatidylcholines	18008	40928	23506	31862	12908	11234	19721	16025	7807	6862	7837	10674	7712	5000	5883	28456
Spegatrine	1.12	348.28427	Macroline alkaloids	4208	21934	17853	20657	23751	11972	30502	8315	4195	9458	8429	8014	4806	6792	8824	11880
SPI_256.2273_14.6	1.152	256.22754	Ketals	2043	14064	12737	17105	28556	17291	11744	7960	1800	5913	2108	3334	2120	2833	5101	3554
Stearic acid	2.911	283.26968	Long-chain fatty acids	19389	19431	18825	41656	24537	44900	17939	13303	62221	20665	54129	42759	41588	60462	58430	59952
Succinic acid	7.322	117.02055	Dicarboxylic acids and derivatives	164027	124153	6831	158231	128253	5357	502	123105	183510	216462	98363	131159	129014	165493	137868	2314
Sucrose	13.42	341.10837	O-glycosyl compounds	2376	3933	8332	2786	1817	5771	4053	756	3481	19971	932	514278	1392	1708	1096	405
Sumatriptan	11.162	294.11972	Tryptamines and derivatives	230977	203501	286080	152518	234688	191945	149252	104008	169734	328001	217736	280631	195713	297264	77819	123578
Tacrolimus	1.298	802.46875	Macrolide lactams	6106	4381	4150	5945	5135	4400	5712	3476	5746	4995	4967	5096	3537	4719	6877	5
Tanshinone IIA	0.902	317.1196	Tanshinones, isotanshinones, and derivatives	13752	15744	19449	27818	28491	30564	36042	27585	9545	13166	9187	12600	19291	19872	17994	8394
Taurine	10.913	124.00821	Organosulfonic acids	2359432	2584460	989190	2067159	1973068	1351182	3125524	1639010	1399656	3727607	1219898	1011523	2787805	1564677	1385797	1588779
taurohyocholic acid	7.758	538.27563	Trihydroxy bile acids, alcohols and derivatives	9491	16709	18280	27870	17530	9021	42439	14668	20888	38782	41879	18294	12426	17991	30846	6330
taurohyodeoxycholic acid	1.3	498.27771		65178	8270	9717	31078	13458	15352	4031	9396	1602	32914	27962	10006	11175	49636	9638	4612
Taxifolin 7-rhamnoside	14.103	451.12701	Flavonoid-7-O-glycosides	4923	82	36	5442	4160	29	3881	5044	5702	74	48	201	5264	83	3320	2307
Teflubenzuron	13.491	380.98615	Dichlorobenzenes	4891	6023	4301	7448	8040	8767	7403	6906	8750	6426	9189	9838	7860	9998	6688	5723
Tenuifoliside A	14.479	705.17896	Coumaric acids and derivatives	7964	15031	20209	4170	12466	6054	14321	17637	6713	34139	11113	13697	7142	19826	5579	4726
Terbumeton	4.2	226.17787	1,3,5-triazine-2,4-diamines	4742	12466	6663	11514	40636	5173	14661	4827	4734	6592	10888	6045	5662	4364	6833	4205
Terfenadine	1.259	472.31021	Diphenylmethanes	10906	30527	4495	3243	71729	2769	4378	7450	8913	2271	995	33758	34595	2476	2226	33980
terrein	5.659	153.06895	1,2-diols	23739	6321	24086	76321	122480	12691	59342	12073	21098	3970	187460	28966	4024	1467	6828	2168
Tetrasaccharides (Hex-Hex-Hex-Hex)	14.478	665.2149	Sugars	6086	9350	13444	3756	8021	4151	8669	11705	4186	20762	4959	9622	4309	12233	3371	52804
Theaflavin	13.723	587.10522	Catechins	876	4324	7879	2504	1271	12851	8086	9880	1304	1952	10028	2617	10295	11355	6218	1002
Theanine	13.377	197.08688	Glutamine and derivatives	12991	24800	40235	10553	6223	19143	27084	3844	13071	6045	19528	3667	14929	24392	6058	8769
Thelephoric acid	13.707	351.01385		122986	334769	607979	233261	221501	340371	560768	394688	219792	518521	526331	595583	322289	259971	408526	275405
Theophylline	10.69	203.0526	Xanthines	15718	62791	33771	15418	36254	80813	39429	67086	21622	98558	90436	96631	112055	104177	32591	7210
Thiazolidine-4-carboxylic acid	7.306	134.02655	L-alpha-amino acids	241162	286996	26	317781	356746	136	160	437858	282894	504439	777126	391291	241429	245788	367230	5771
Thiophanic acid	14.141	392.87967	Xanthones	173399	102242	77569	91390	111263	171496	103387	50493	87296	58539	119612	172759	102803	137726	182336	220145
Threonic acid	10.515	135.03082	Sugar acids and derivatives	196677	162472	194877	30300	173305	203759	176203	146883	29265	126246	251733	154597	138669	119325	28277	61040
Threonine	13.033	118.05106	L-alpha-amino acids	605684	432642	215110	589334	484616	448165	458763	125280	260628	477138	357160	569361	377426	209400	366956	374398
threonine conjugated cholic acid	7.772	510.35095		59348	63453	65377	58836	45995	53277	61975	30352	51020	54749	34745	54903	48305	22075	28503	57080
Thuringione	14.698	374.96039		298594	271837	203054	238230	295480	308195	382850	345089	332519	333808	287935	331659	336559	459661	353788	301132
Thymol-beta-D-glucoside	1.262	311.17007		53036	60170	33848	43246	343848	30215	25317	36141	77688	44967	22309	197988	109457	26346	23830	56961
tigogenin	1.123	417.33612	Triterpenoids	28373	24311	17915	27663	28051	40192	28565	26305	26710	13054	10036	16780	10081	16325	21353	31979

Tioconazole	13.603	386.97214	Benzylethers	899	1120	5103	568	772	1616	3772	948	766	2867	3574	2716	2037	1016	1370	1098
Tobramycin, N-propionyl derivative likely	7.473	524.29785	4,6-disubstituted 2-deoxystreptamines	192669	181885	88866	163618	194090	141037	192560	109792	158442	318506	15874	71603	209810	288883	104542	157284
Toddalolactone	13.578	309.12582	Coumarins and derivatives	20610	10984	6075	6267	14911	17098	11338	8378	11653	5435	24410	4826	13370	22934	7653	7318
Tolytoxin	1.323	832.5282	Diterpene lactones	20077	30017	23214	24234	26415	17341	17588	29306	16577	35948	11060	23254	22807	26289	20066	9996
Toradol	7.354	254.08252	Aryl-phenylketones	18829	23618	6755	11011	18322	7477	11680	16516	14014	8572	12913	10142	20326	25586	10176	53
trans-Cinnamic acid	9.623	147.0444	Cinnamic acids	57399	82772	73360	66802	68383	87221	71279	58234	58230	60898	63912	57461	52213	62299	41822	53846
Trans-Vaccenic acid	1.259	281.24966	Long-chain fatty acids	154731	202828	117533	207549	179607	73417	196751	388925	185981	222827	171740	155122	453239	130614	150581	550684
tricosanoic acid	1.065	377.32648	Very long-chain fatty acids	6612	16235	10029	7332	23233	11424	8763	8855	6212	7710	3351	19073	8723	9031	5754	24097
Trifluoroacetic acid	1.269	112.98656	PFSA	906337	691161	536779	745160	994170	927516	877109	617850	978411	839376	837891	721912	875892	913337	832227	921005
Trigonelline	10.562	138.0537	Alkaloids and derivatives	20916	185961	274326	63012	71162	204308	245980	121138	153734	275241	288645	151413	191588	55161	69204	268475
Trinitrotoluene	4.553	227.01451	Nitrobenzenes	25256	54327	38389	31965	19268	5759	8208	32432	41093	5772	12435	22687	10768	2509	29844	1388
Triptolide	10.96	383.13565	Oxepanes	9535	30967	9463	32332	9768	17070	16468	24136	17948	44143	32025	37425	36140	26582	38678	22351
Tris(2-chloroethyl)phosphate	14.682	284.97046	Trialkyl phosphates	33524	35416	23638	41177	33034	48281	50049	51853	31769	53012	37553	36201	49562	49964	44148	14076
Tritosulfuron	13.77	444.02655	S-triazinyl-2-sulfonylureas	38853	32663	14437	22064	26313	7237	16940	23406	39319	41871	23322	32077	15819	49839	14857	144967
Tryptophan	9.719	203.08455	Indolyl carboxylic acids and derivatives	141379	306479	179165	149500	191110	384967	260233	60615	153806	235462	204187	229691	201871	56538	153089	344220
Tyrosine	11.24	180.06749	Tyrosine and derivatives	2280433	2104183	2647269	1990272	1842543	2333930	1722455	1823847	1420910	2207182	1642599	2427989	1089482	1069791	939772	1641233
UDP-D-glucose	13.764	565.05707	Pyrimidine nucleotide sugars	46767	27770	19338	50933	42679	19680	28903	37447	36841	14799	10034	30285	46748	21387	88615	13223
Urapidil	1.324	386.2106	Phenylpiperazines	1365	4679	3474	4760	3057	1228	10437	1600	1330	1520	2003	2358	1669	1425	1814	579
Urea	4.361	61.03984	Ureas	191023	159450	405297	180377	263662	209406	131584	169499	343989	258624	226017	140482	298873	196862	409397	442431
Uridine 5'-diphosphate	13.842	403.00583	Pyrimidine ribonucleoside diphosphates	5786	2629	4367	5179	4907	2338	2022	5324	5389	2058	2533	4628	6956	3124	10776	625
Uridine 5'-diphospho-N-acetylglucosamine	13.727	606.07446	Pyrimidine nucleotide sugars	5183	5571	3897	7304	7415	4721	4614	7012	5789	7185	5132	5427	8270	12421	20508	8488
Uridine 5'-monophosphate	13.796	323.03424	Pyrimidine ribonucleoside monophosphates	28921	10550	6848	7110	16854	17777	8269	6797	14663	12418	5598	8249	6599	25916	20701	28707
veratramine	1.338	432.27725	Fluorenes	13673	66265	59897	124926	118662	130087	39927	60228	131209	87258	38378	81972	103022	103734	294261	470
Veratrosine	7.504	594.34021	Fluorenes	31733	67308	3827	67224	42006	54093	53414	40983	36547	90045	65162	46025	81856	47852	39082	45418
Vicriviroc Malate	7.22	534.29437	Trifluoromethylbenzenes	39346	2442	2052	19377	20826	1519	5334	3473	25135	2418	7796	4095	38892	2546	2435	20892
Vinpocetine	1.104	351.21796	Eburnan-type alkaloids	31793	69034	34924	31408	19282	62762	45551	45052	28620	24861	16524	50676	24905	50863	43823	80518
Vinyl Carbamate	7.308	88.02257	Carbamate esters	27741	44502	34	36807	50056	39717	110	51566	39104	54533	97736	52579	30380	33499	46941	542
Xanthurenic Acid	12.305	204.0358		5323	12236	20242	7163	4620	5930	11618	14147	8408	23766	7025	12292	18020	20300	16059	5886
YOHIMBIC ACID HYDRATE	1.125	339.20102	Corynanthean-type alkaloids	94840	73594	46001	70537	582734	8917	37003	71808	106797	23210	30147	203637	120562	20730	29600	21117
Zearalenone	9.662	317.14331	Zearalenones	27841	25973	31123	18891	38813	31216	30019	27503	22241	37483	23541	26116	24438	23117	18041	34427
Zeatin	4.865	254.07137	6-alkylaminopurines	14189	13042	9864	21678	64299	23835	9213	17309	29679	19648	18864	21400	5969	5208	11615	6882

Table S5: 81 different blood metabolites between *Laiwu pigs* and *Lulai pigs*.

Metabolite name	VIP	FC	log2(fc)	Pvalue	LU-1	LU-2	LU-3	LU-4	LU-5	LU-6	LU-7	LU-8	LW-1	LW-2	LW-3	LW-4	LW-5	LW-6	LW-7	LW-8
1-Hydroxy-2-naphthoic acid	1.824550	3.497952	0.543814	0.024407	27344	8062	26026	6988	60584	83104	29383	55736	5686	10907	9431	5124	12319	7521	23362	42640
2,4-Diaminobenzenesulfonic acid	8.521770	2.030191	0.307537	0.011068	1378149	1365240	1332639	722827	916917	1745051	667445	457677	461880	652132	928237	468783	279827	451422	458211	333585
2,5-Dichlorolichexanthone	3.191570	0.793837	-0.100269	0.040445	311953	291007	275220	410521	449248	374902	410504	349700	415224	324943	525219	495026	341335	488039	577013	201842
5-Hydroxyindoleacetic acid	1.533530	0.433608	-0.362903	0.024041	7239	18597	9845	17467	10213	26965	10501	12791	19810	40087	50838	26843	11570	63848	16283	50
5-Methoxypsoralen	2.290840	0.463693	-0.333770	0.034103	24876	106720	15419	21282	75093	14204	32306	45569	24187	62011	55921	112447	135716	152525	90230	194101
5-O-Caffeoylquinic acid methyl ester	6.215510	0.547210	-0.261846	0.015255	379305	223438	518498	305323	486598	384911	611841	317069	1003767	835983	336164	482621	1217143	753130	531210	554965
6-hydroxy-7-methyoxyscytophycin E	1.140240	0.420495	-0.376239	0.039172	2027	1733	2493	4017	4236	8959	16183	19065	4303	24421	24617	24560	3385	13997	26892	3229
7-Hydroxymitragynine	1.657210	1.528464	0.184255	0.048110	36077	95073	68177	62392	75253	123120	77837	89677	39920	81871	15483	49768	42688	73430	56125	249699
Acetaminophen	1.141020	13.429785	1.128069	0.001323	4506	13222	18777	5069	11277	6466	5847	4542	882	856	918	872	241	147	627	405
Alpha Cyperone	6.209810	0.600649	-0.221379	0.006804	454782	482407	461787	525300	197780	508337	157438	470072	1076305	595229	625446	562388	713076	583186	590345	364298
alpha-Methylhistidine	9.608210	0.517391	-0.286181	0.018016	477389	760780	616100	790098	575715	1239170	617562	634830	1038162	2104759	1990476	1169314	451224	2011729	893733	67610
Angelic anhydride	5.511960	1.556717	0.192210	0.000252	330565	474728	469071	532867	485632	537009	536818	499743	248670	270975	249806	271820	417301	361981	352693	812076
Angelicin	2.143160	0.194012	-0.712171	0.026299	88	18360	143	13457	3593	350	57	28497	9914	115262	7117	50919	32680	32935	42278	576
betaine	2.336470	0.330673	-0.480602	0.019975	55309	9172	44012	5932	761	36315	20574	9465	149079	61092	43154	78994	92731	11545	43781	6478
Brazilein	2.785770	0.525389	-0.279519	0.042394	90212	56151	6785	105010	136305	34295	7931	90902	127938	233600	144107	83559	66589	130226	92650	3061
Chloridazone-desphenyl	1.762420	0.307060	-0.512777	0.008686	624	17132	117	18308	6335	4685	167	22455	7431	54276	33465	37647	16481	23466	26203	165
Cholecalciferol	1.035000	1.439748	0.158286	0.000263	20653	15854	18642	25788	19617	21712	18818	22354	16966	12713	13683	13541	12546	15674	14205	21127
Choline	6.262100	0.612385	-0.212975	0.001936	478998	148842	554251	363204	467912	361899	431887	655288	654195	626466	838454	699970	901093	654514	572353	520381
Chrysanthemic Acid	2.030460	2.136480	0.329699	0.001051	27232	57272	45761	40861	68507	58934	38897	64914	24803	10801	39150	15305	19540	25303	29893	100717
CITRATE	2.289450	0.481622	-0.317293	0.022667	28558	96046	117029	26529	25215	11105	10567	10310	74434	82250	101684	65576	77699	113750	75711	9783
Creatinine	6.503700	1.590892	0.201641	0.018871	973350	1053360	945889	859242	759930	1454636	593135	509534	492572	440955	772213	522376	370942	582265	750709	432075
Curdione	2.790000	0.447033	-0.349660	0.018486	34811	13681	50852	41269	35794	17165	66906	94698	106669	175478	67131	39131	57532	95271	153990	35756
Cytarabine	1.176710	0.532748	-0.273478	0.006810	15929	12008	4794	16823	8916	14548	10664	9172	24712	18291	8887	18386	23054	24574	34603	26192
Cytidine	1.868480	0.373135	-0.428134	0.001370	14310	18743	7148	19077	10472	5972	16022	16263	27012	30439	12657	53222	33584	41648	54711	35377
Dehydroeffusol	1.191290	2.386080	0.377685	0.003755	12782	16404	7292	10065	27229	24375	22456	15370	4782	9734	7163	8824	4180	9213	5967	18245
D-Erythrose-4-phosphate	1.613690	5.160724	0.712711	0.012038	46898	59540	27140	13832	13623	17277	14482	9574	7670	2869	4298	6759	7627	1705	3383	2742
Dibutyl phthalate	8.811400	1.606889	0.205986	0.000214	771107	1139155	1163378	1315457	1157189	1274115	1268883	1199435	568675	623142	565011	631494	968383	865158	836127	1991972
DL-2-Hydroxyvaleric acid	1.893690	2.206913	0.343785	0.008056	54161	16902	39857	41360	76970	48954	47597	18442	17702	20316	18826	28731	19289	14365	17257	41916
Erythromycin	1.776650	1.260940	0.100694	0.006670	100916	99989	120561	87215	109029	92207	112077	100832	94248	57881	99193	91506	80963	68716	78473	51254
Fipronil sulfone	1.758410	1.235057	0.091687	0.021771	160860	116349	111975	129447	117953	150965	125505	122862	133645	97606	107460	85051	125448	82184	102519	169943
Gabapentin	2.851260	2.424126	0.384555	0.015916	74549	87302	99760	157071	202379	82577	53377	123731	33202	5681	119382	40654	5682	38869	74439	11876
Ginkgolide A	1.550210	0.450052	-0.346737	0.021555	17297	17265	34524	1279	26117	713	3653	19417	30133	54869	39644	6315	37132	26230	39501	59344
Glutamine	2.268750	0.827056	-0.082465	0.046387	209012	202049	146676	196088	243131	212183	261085	197399	202232	295711	308199	217066	255086	274408	211593	133467
Gomisin H	1.010760	2.297513	0.361258	0.004098	10133	18379	18639	18619	8171	9030	12087	6261	5099	8245	5801	6964	4366	5661	2451	15836
Goniothalenol	2.371290	0.548726	-0.260644	0.018991	64640	25158	22764	53689	10788	84285	70254	83855	83198	100955	69086	72786	73212	99772	163443	10560
Gossypetin	2.296160	0.791867	-0.101348	0.043176	162276	154627	147006	209485	228067	191883	224820	179423	222370	168659	257826	274900	167134	262304	301617	105039
Hectochlorin	1.090440	0.664574	-0.177456	0.009896	24434	18998	9079	20652	15805	14929	16682	17499	26447	21763	23461	24057	17514	37894	30663	9741
His	1.223570	0.630399	-0.200384	0.016812	16630	19619	17574	15975	18251	15132	22791	17836	16284	37283	44743	33783	23993	16860	26661	98760
Homocysteine	1.727100	0.441089	-0.355474	0.001976	26842	12038	7662	9034	32020	18004	9169	11360	52742	36237	29955	28527	28362	49190	25193	31343
Hypoxanthine	15.537100	0.313096	-0.504323	0.021141	475628	590701	345719	1383726	2193237	495424	767011	676262	3761104	2816755	500983	2746862	2193719	959692	6381571	1297805
icosanoic acid	1.421410	2.500738	0.398068	0.037811	9918	28345	26579	38186	58048	10374	60098	13476	7585	23372	16658	5171	8011	13504	11432	13914
inosine	1.191470	0.462117	-0.335248	0.034799	4130	12456	17035	8280	16876	3994	6811	17730	20753	21851	3478	15611	26995	27223	49413	10882
isofraxidin	1.183780	0.658417	-0.181499	0.026739	29570	16992	10245	22225	26938	13701	9806	31630	20207	41088	34950	28172	30060	23579	36045	13142
Isopimpinellin	1.703090	0.463188	-0.334242	0.035461	17966	13621	4719	8601	26721	18664	44453	37176	12828	32950	96364	42613	46402	59426	34188	29329
Juarezic Acid	3.863110	2.386932	0.377840	0.035622	377596	535476	227408	169318	184597	145558	146659	91751	110712	42236	144644	105622	144704	44460	96190	66263
Khasianine	1.707940	1.716753	0.234708	0.006144	59752	44081	46035	54221	57124	27375	74860	32577	33916	25682	37099	31948	25951	15811	31441	10501
Licochalcone A	1.819090	0.534962	-0.271677	0.046580	28464	42271	533	18922	61004	16210	34634	77917	64302	50145	43300	113430	97027	44697	45002	2961
LPC 16:0	11.334100	0.803542	-0.094992	0.010760	4621819	4333304	4114656	4923217	4050596	4096438	4890661	3239452	5531747	5065105	5217610	6446378	5749448	5506766	3800708	5736399
LPE 16:0	2.605750	0.708168	-0.149864	0.038409	139721	199200	114929	198473	160939	176887	156350	78506	166087	250106	201813	160295	329657	248508	157128	27106
Mebeverine	6.070450	1.769911	0.247951	0.000348	303755	442246	483027	625275	491318	486623	531563	494382	207809	190515	204055	250470	378332	349816	326395	1413612
Metformin	2.573370	2.443469	0.388007	0.001812	33479	55482	57924	118116	70916	90835	102302	62850	19749	41611	20027	29651	20917	34801	45203	101952
N,N-Dimethylaniline	2.809370	1.692124	0.228432	0.009815	115714	142098	225739	90285	99974	131287	100854	174676	58092	66494	75759	73884	105257	94403	84905	129137
N4-Acetylsulfamethoxazole	1.886730	0.525171	-0.279699	0.016974	21078	27620	35477	24472	18245	14454	35558	35755	51067	101236	48317	44292	37764	31518	40123	16157
N-Acetylneuraminic acid	1.674490	0.515392	-0.287863	0.021235	28106	14323	56355	7474	40504	6722	12111	22758	46470	64892	58007	44995	35822	19475	50111	19345
Nervonic Acid	1.196170	1.793264	0.253644	0.001472	12659	17658	14927	24295	26862	25031	22117	23707	13293	10774	5879	12972	9132	13114	16447	30017
Nicotinamide	6.262420	0.549707	-0.259869	0.002094	364005	321333	201997	299788	388935	533870	275539	341151	540655	498115	585069	492435	986712	716915	520215	8942469
N-Isovaleroylglycine	1.753030	2.008008	0.302765	0.028655	42358	36217	43169	75540	96793	33500	48721	32101	19742	19560	58028	34868	17850	12505	15409	23858
Nitrazepam	1.247220	95.161767	1.978462	0.043958	45897	27245	20233	217	0	294	520	23420	45	58	97	158	158	51	517	220
N-Methylcytisine	2.741150	0.633164	-0.198484	0.039304	100059	81009	65820	67072	164612	58611	122195	152415	95782	235628	237571	149548	109021	173005	121302	473
Palmitoylcarnitine	1.433860	0.477901	-0.320662	0.003852	13787	14071	8417	13759	9914	16762	5067	19475	35462	21187	19020	11872	31514	25733	40598	81034
PC(14:0/10-HDoHE)	1.835830	2.150286	0.332496	0.031211	19718	42976	65824	91387	60217	41302	83903	38072	14119	20544	38024	13567	18785	3376	72013	199
PC(17:0/9-HODE)	1.365130	1.428344	0.154833	0.041505	84320	47015	55169	56055	43885	35150	70379	50008	56049	33867	29153	49417	31072	23479	47718	3399
Phosphatidylcholine 16	1.184790	2.071448	0.316274	0.045393	26775	16917	33996	12133	24298	11164	53407	18019	11764	24241	4142	10763	9094	8401	14686	1710
Phthalic anhydride	4.088350	1.358620	0.133098	0.000997	284709	364581	362883	404572	382384	415544	418414	395234	241410	249182	228052	252024	358913	313567	307199	536255
Royal jelly acid	1.022140	1.693879	0.228882	0.010490	10323	15651	13372	18927	18526	23901	27946	15898	8598	9435	6623	8801	14299	16488	10423	47722
Scytophycin E	1.258740	1.655596	0.218954	0.008889	37623	33583	31434	30769	25571	22974	28805	18751	30676	6403	22925	17325	18273	8582	17113	43967

**sebacic acid**	3.077700	2.046160	0.310940	0.021777	120178	242859	151868	98737	130738	176158	158022	228658	11891	151275	98818	21153	200007	52213	23650	205712
**securinine**	3.143840	0.310434	-0.508030	0.007611	24604	74041	56	38542	36666	451	315	65947	59190	149586	57973	68334	67157	187773	88214	1829
**skimmianine**	1.287620	0.583210	-0.234175	0.008190	11409	12142	13812	15459	16041	19813	13147	18087	36252	38697	30011	20884	16156	21120	16783	8680
**sn-Glycero-3-phosphocholine**	2.583130	0.688723	-0.161956	0.018166	123823	103693	83680	120153	100450	62775	105133	140960	183474	224679	110687	110928	142155	171288	124830	104700
**Solanidine base + O-Hex-dHex**	1.010160	1.861745	0.269920	0.029350	9762	7372	16753	24181	32327	20359	25142	13445	8879	12472	16278	8662	5804	4728	13367	18481
**Soy bean phospholipid**	1.423030	2.943732	0.468898	0.002664	18008	40928	23506	31862	12908	11234	19721	16025	7807	6862	7837	10674	7712	5000	5883	28456
**Spegatrine**	1.153380	2.410938	0.382186	0.009983	4208	21934	17853	20657	23751	11972	30502	8315	4195	9458	8429	8014	4806	6792	8824	11880
**SPI 256.2273 14.6**	1.253550	4.203681	0.623630	0.003538	2043	14064	12737	17105	28556	17291	11744	7960	1800	5913	2108	3334	2120	2833	5101	3554
**Stearic acid**	1.790700	0.514270	-0.288808	0.004339	19389	19431	18825	41656	24537	44900	17939	13303	62221	20665	54129	42759	41588	60462	58430	59952
**Tanshinone IIA**	1.225340	1.716750	0.234707	0.008341	13752	15744	19449	27818	28491	30564	36042	27585	9545	13166	9187	12600	19291	19872	17994	8394
**Theophylline**	1.984140	0.552755	-0.257467	0.041954	15718	62791	33771	15418	36254	80813	39429	67086	21622	98558	90436	96631	112055	104177	32591	7210
**tigogenin**	1.289450	1.694101	0.228939	0.003414	28373	24311	17915	27663	28051	40192	28565	26305	26710	13054	10036	16780	10081	16325	21353	31979
**trans-Cinnamic acid**	1.342320	1.246783	0.095791	0.012250	57399	82772	73360	66802	68383	87221	71279	58234	58230	60898	63912	57461	52213	62299	41822	53846
**Triptolide**	1.497000	0.562469	-0.249901	0.008589	9535	30967	9463	32332	9768	17070	16468	24136	17948	44143	32025	37425	36140	26582	38678	22351
**Tyrosine**	7.794410	1.356935	0.132559	0.037036	2280433	2104183	2647269	1990272	1842543	2333930	1722455	1823847	1420910	2207182	1642599	2427989	1089482	1069791	939772	1641233

Table S6: Clean data information for the whole genome.

Sample ID	Index	Size（Gb）	Reads	Depth	Coverage	Q20（%）	Q30（%）	GC（%）
LW-F-1	TCGGTCGA-GCACTTGG	21.28	139765006	6.56	0.97	96.45	90.76	41.89
LW-F-2	CGCGCCAA-CCTAGCCA	22.452	147332746	6.95	0.97	96.24	90.36	42.26
LW-F-3	CGATTATC-ATCAACTC	19.223	126204358	6.13	0.97	96.56	91.02	42.4
LW-F-4	TCGGTAAG-TGAGTTAG	23.052	151508352	7.27	0.97	96.35	90.56	42.13
LW-F-5	TCGGATGT-ATCCGCAG	25.171	165468352	7.74	0.97	96.48	90.85	42.31
LW-F-6	CGAGCCGG-CAAGCCGC	20.611	135367908	6.50	0.97	96.31	90.51	42.43
LW-F-7	CTATCATT-AGGTGGTC	20.457	134365496	6.25	0.97	96.3	90.44	42.04
LW-F-8	CTACTGAC-CATCCTCC	20.902	137241236	6.54	0.97	96.25	90.41	42.2
LU-F-1	TTATTATG-TGCGCTAA	18.39	120811848	5.83	0.97	96.31	90.49	42.24
LU-F-2	TTAGAGTC-TCGTCACA	23.607	155000630	7.36	0.97	96.55	91.02	42.08
LU-F-3	TCTTAAGT-ATTCAGCG	20.531	134947224	6.47	0.97	96.26	90.34	42.26
LU-F-4	TCGGTCGA-GCACTTGG	21.28	139765006	6.56	0.97	96.45	90.76	41.89
LU-F-5	CGAGCCGG-CAAGCCGC	20.611	135367908	6.50	0.97	96.31	90.51	42.43
LU-F-6	TCGAAGCT-CTCAGTGC	21.555	141474748	6.72	0.97	96.29	90.47	42.45
LU-F-7	CGAACGGA-CCGGTAGG	23.365	153061414	7.40	0.97	95.69	89.27	42.18
LU-F-8	AAGACGAA-AATCTATT	22.03	144934502	6.85	0.97	96.56	91.04	42.21

Table S7: 811 annotated genes for top 1% Fst.

CHR	BP1	BP2	FST	chr	start_pos	end_pos	width	strand	gene_id	gene_name	gene_biotype
16	48600001	48650000	0.419002	16	48644464	48693635	49172	-	ENSSSCG00000039883	NA	lncRNA
16	48610001	48660000	0.393737	16	48644464	48693635	49172	-	ENSSSCG00000039883	NA	lncRNA
16	48620001	48670000	0.370223	16	48644464	48693635	49172	-	ENSSSCG00000039883	NA	lncRNA
16	48630001	48680000	0.386674	16	48644464	48693635	49172	-	ENSSSCG00000039883	NA	lncRNA
16	48640001	48690000	0.388168	16	48644464	48693635	49172	-	ENSSSCG00000039883	NA	lncRNA
16	48650001	48700000	0.368107	16	48644464	48693635	49172	-	ENSSSCG00000039883	NA	lncRNA
16	48660001	48710000	0.371475	16	48644464	48693635	49172	-	ENSSSCG00000039883	NA	lncRNA
16	48670001	48720000	0.383088	16	48644464	48693635	49172	-	ENSSSCG00000039883	NA	lncRNA
16	48680001	48730000	0.380486	16	48644464	48693635	49172	-	ENSSSCG00000039883	NA	lncRNA
16	48690001	48740000	0.375741	16	48644464	48693635	49172	-	ENSSSCG00000039883	NA	lncRNA
16	48610001	48660000	0.393737	16	48659175	48662360	3186	+	ENSSSCG00000048660	NA	lncRNA
16	48620001	48670000	0.370223	16	48659175	48662360	3186	+	ENSSSCG00000048660	NA	lncRNA
16	48630001	48680000	0.386674	16	48659175	48662360	3186	+	ENSSSCG00000048660	NA	lncRNA
16	48640001	48690000	0.388168	16	48659175	48662360	3186	+	ENSSSCG00000048660	NA	lncRNA
16	48650001	48700000	0.368107	16	48659175	48662360	3186	+	ENSSSCG00000048660	NA	lncRNA
16	48660001	48710000	0.371475	16	48659175	48662360	3186	+	ENSSSCG00000048660	NA	lncRNA
16	48630001	48680000	0.386674	16	48679966	48690760	10795	+	ENSSSCG00000044917	NA	lncRNA
16	48640001	48690000	0.388168	16	48679966	48690760	10795	+	ENSSSCG00000044917	NA	lncRNA
16	48650001	48700000	0.368107	16	48679966	48690760	10795	+	ENSSSCG00000044917	NA	lncRNA
16	48660001	48710000	0.371475	16	48679966	48690760	10795	+	ENSSSCG00000044917	NA	lncRNA
16	48670001	48720000	0.383088	16	48679966	48690760	10795	+	ENSSSCG00000044917	NA	lncRNA
16	48680001	48730000	0.380486	16	48679966	48690760	10795	+	ENSSSCG00000044917	NA	lncRNA
16	48690001	48740000	0.375741	16	48679966	48690760	10795	+	ENSSSCG00000044917	NA	lncRNA
16	48640001	48690000	0.388168	16	48687074	48692112	5039	+	ENSSSCG00000046046	NA	lncRNA
16	48650001	48700000	0.368107	16	48687074	48692112	5039	+	ENSSSCG00000046046	NA	lncRNA
16	48660001	48710000	0.371475	16	48687074	48692112	5039	+	ENSSSCG00000046046	NA	lncRNA
16	48670001	48720000	0.383088	16	48687074	48692112	5039	+	ENSSSCG00000046046	NA	lncRNA
16	48680001	48730000	0.380486	16	48687074	48692112	5039	+	ENSSSCG00000046046	NA	lncRNA
16	48690001	48740000	0.375741	16	48687074	48692112	5039	+	ENSSSCG00000046046	NA	lncRNA
16	49440001	49490000	0.354429	16	49409066	49539916	130851	+	ENSSSCG00000047960	NA	lncRNA
16	49450001	49500000	0.389916	16	49409066	49539916	130851	+	ENSSSCG00000047960	NA	lncRNA
16	49460001	49510000	0.455132	16	49409066	49539916	130851	+	ENSSSCG00000047960	NA	lncRNA
16	49470001	49520000	0.464842	16	49409066	49539916	130851	+	ENSSSCG00000047960	NA	lncRNA
16	49480001	49530000	0.427624	16	49409066	49539916	130851	+	ENSSSCG00000047960	NA	lncRNA
16	49490001	49540000	0.41886	16	49409066	49539916	130851	+	ENSSSCG00000047960	NA	lncRNA
16	49500001	49550000	0.424468	16	49409066	49539916	130851	+	ENSSSCG00000047960	NA	lncRNA
16	49510001	49560000	0.430194	16	49409066	49539916	130851	+	ENSSSCG00000047960	NA	lncRNA
16	49520001	49570000	0.43549	16	49409066	49539916	130851	+	ENSSSCG00000047960	NA	lncRNA
16	49530001	49580000	0.432365	16	49409066	49539916	130851	+	ENSSSCG00000047960	NA	lncRNA
16	49510001	49560000	0.430194	16	49556811	49557933	1123	-	ENSSSCG00000049237	NA	lncRNA
16	49520001	49570000	0.43549	16	49556811	49557933	1123	-	ENSSSCG00000049237	NA	lncRNA
16	49530001	49580000	0.432365	16	49556811	49557933	1123	-	ENSSSCG00000049237	NA	lncRNA
16	49540001	49590000	0.436986	16	49556811	49557933	1123	-	ENSSSCG00000049237	NA	lncRNA

16	49550001	49600000	0.3924	16	49556811	49557933	1123	-	ENSSSCG00000049237	NA	lncRNA
16	49720001	49770000	0.356251	16	49760300	49766471	6172	+	ENSSSCG00000046443	NA	lncRNA
16	50040001	50090000	0.587405	16	50089947	50102101	12155	+	ENSSSCG00000050829	NA	lncRNA
16	50050001	50100000	0.508961	16	50089947	50102101	12155	+	ENSSSCG00000050829	NA	lncRNA
16	50060001	50110000	0.52181	16	50089947	50102101	12155	+	ENSSSCG00000050829	NA	lncRNA
16	50070001	50120000	0.524409	16	50089947	50102101	12155	+	ENSSSCG00000050829	NA	lncRNA
16	50080001	50130000	0.518185	16	50089947	50102101	12155	+	ENSSSCG00000050829	NA	lncRNA
16	50090001	50140000	0.542376	16	50089947	50102101	12155	+	ENSSSCG00000050829	NA	lncRNA
16	50100001	50150000	0.582993	16	50089947	50102101	12155	+	ENSSSCG00000050829	NA	lncRNA
16	50520001	50570000	0.628039	16	50560971	50571203	10233	+	ENSSSCG00000041085	NA	lncRNA
16	50530001	50580000	0.617944	16	50560971	50571203	10233	+	ENSSSCG00000041085	NA	lncRNA
16	50540001	50590000	0.608262	16	50560971	50571203	10233	+	ENSSSCG00000041085	NA	lncRNA
16	50550001	50600000	0.611194	16	50560971	50571203	10233	+	ENSSSCG00000041085	NA	lncRNA
16	50560001	50610000	0.596529	16	50560971	50571203	10233	+	ENSSSCG00000041085	NA	lncRNA
16	50570001	50620000	0.599225	16	50560971	50571203	10233	+	ENSSSCG00000041085	NA	lncRNA
16	50550001	50600000	0.611194	16	50593909	50625382	31474	+	ENSSSCG00000045534	NA	lncRNA
16	50560001	50610000	0.596529	16	50593909	50625382	31474	+	ENSSSCG00000045534	NA	lncRNA
16	50570001	50620000	0.599225	16	50593909	50625382	31474	+	ENSSSCG00000045534	NA	lncRNA
16	50580001	50630000	0.558347	16	50593909	50625382	31474	+	ENSSSCG00000045534	NA	lncRNA
16	50590001	50640000	0.537101	16	50593909	50625382	31474	+	ENSSSCG00000045534	NA	lncRNA
16	50600001	50650000	0.512078	16	50593909	50625382	31474	+	ENSSSCG00000045534	NA	lncRNA
16	50610001	50660000	0.534021	16	50593909	50625382	31474	+	ENSSSCG00000045534	NA	lncRNA
16	50620001	50670000	0.530474	16	50593909	50625382	31474	+	ENSSSCG00000045534	NA	lncRNA
16	50550001	50600000	0.611194	16	50597530	50603379	5850	+	ENSSSCG00000049195	NA	lncRNA
16	50560001	50610000	0.596529	16	50597530	50603379	5850	+	ENSSSCG00000049195	NA	lncRNA
16	50570001	50620000	0.599225	16	50597530	50603379	5850	+	ENSSSCG00000049195	NA	lncRNA
16	50580001	50630000	0.558347	16	50597530	50603379	5850	+	ENSSSCG00000049195	NA	lncRNA
16	50590001	50640000	0.537101	16	50597530	50603379	5850	+	ENSSSCG00000049195	NA	lncRNA
16	50600001	50650000	0.512078	16	50597530	50603379	5850	+	ENSSSCG00000049195	NA	lncRNA
16	50630001	50680000	0.561223	16	50679652	50683824	4173	-	ENSSSCG00000044465	NA	lncRNA
16	50640001	50690000	0.57756	16	50679652	50683824	4173	-	ENSSSCG00000044465	NA	lncRNA
16	50650001	50700000	0.595894	16	50679652	50683824	4173	-	ENSSSCG00000044465	NA	lncRNA
16	50660001	50710000	0.610016	16	50679652	50683824	4173	-	ENSSSCG00000044465	NA	lncRNA
16	50670001	50720000	0.617236	16	50679652	50683824	4173	-	ENSSSCG00000044465	NA	lncRNA
16	50680001	50730000	0.581546	16	50679652	50683824	4173	-	ENSSSCG00000044465	NA	lncRNA
16	50670001	50720000	0.617236	16	50711700	50729797	18098	+	ENSSSCG00000041934	NA	lncRNA
16	50680001	50730000	0.581546	16	50711700	50729797	18098	+	ENSSSCG00000041934	NA	lncRNA
16	50690001	50740000	0.525755	16	50711700	50729797	18098	+	ENSSSCG00000041934	NA	lncRNA
16	50700001	50750000	0.481302	16	50711700	50729797	18098	+	ENSSSCG00000041934	NA	lncRNA
16	50710001	50760000	0.459049	16	50711700	50729797	18098	+	ENSSSCG00000041934	NA	lncRNA
16	50720001	50770000	0.381445	16	50711700	50729797	18098	+	ENSSSCG00000041934	NA	lncRNA
16	50670001	50720000	0.617236	16	50714946	50733266	18321	+	ENSSSCG00000045483	NA	lncRNA
16	50680001	50730000	0.581546	16	50714946	50733266	18321	+	ENSSSCG00000045483	NA	lncRNA
16	50690001	50740000	0.525755	16	50714946	50733266	18321	+	ENSSSCG00000045483	NA	lncRNA
16	50700001	50750000	0.481302	16	50714946	50733266	18321	+	ENSSSCG00000045483	NA	lncRNA

16	50710001	50760000	0.459049	16	50714946	50733266	18321	+	ENSSSCG00000045483	NA	lncRNA
16	50720001	50770000	0.381445	16	50714946	50733266	18321	+	ENSSSCG00000045483	NA	lncRNA
16	50810001	50860000	0.433671	16	50845621	50853137	7517	-	ENSSSCG00000046318	NA	lncRNA
16	50820001	50870000	0.400144	16	50845621	50853137	7517	-	ENSSSCG00000046318	NA	lncRNA
16	50920001	50970000	0.442659	16	50969238	50988246	19009	-	ENSSSCG00000045258	NA	lncRNA
16	50930001	50980000	0.496802	16	50969238	50988246	19009	-	ENSSSCG00000045258	NA	lncRNA
16	50940001	50990000	0.489762	16	50969238	50988246	19009	-	ENSSSCG00000045258	NA	lncRNA
16	50950001	51000000	0.513872	16	50969238	50988246	19009	-	ENSSSCG00000045258	NA	lncRNA
16	50960001	51010000	0.459745	16	50969238	50988246	19009	-	ENSSSCG00000045258	NA	lncRNA
16	50970001	51020000	0.422944	16	50969238	50988246	19009	-	ENSSSCG00000045258	NA	lncRNA
16	50980001	51030000	0.377498	16	50969238	50988246	19009	-	ENSSSCG00000045258	NA	lncRNA
16	51020001	51070000	0.424092	16	51068577	51076844	8268	-	ENSSSCG00000050883	NA	lncRNA
16	51030001	51080000	0.416616	16	51068577	51076844	8268	-	ENSSSCG00000050883	NA	lncRNA
16	51040001	51090000	0.407665	16	51068577	51076844	8268	-	ENSSSCG00000050883	NA	lncRNA
16	51050001	51100000	0.372637	16	51068577	51076844	8268	-	ENSSSCG00000050883	NA	lncRNA
16	51700001	51750000	0.373765	16	51668311	51716298	47988	+	ENSSSCG00000041586	NA	lncRNA
16	51710001	51760000	0.385899	16	51668311	51716298	47988	+	ENSSSCG00000041586	NA	lncRNA
16	52200001	52250000	0.39686	16	52205273	52211614	6342	+	ENSSSCG00000048334	NA	lncRNA
16	52210001	52260000	0.365407	16	52205273	52211614	6342	+	ENSSSCG00000048334	NA	lncRNA
16	52320001	52370000	0.37859	16	52339637	52352461	12825	+	ENSSSCG00000047817	NA	lncRNA
16	52330001	52380000	0.403737	16	52339637	52352461	12825	+	ENSSSCG00000047817	NA	lncRNA
16	52340001	52390000	0.424991	16	52339637	52352461	12825	+	ENSSSCG00000047817	NA	lncRNA
16	52350001	52400000	0.444782	16	52339637	52352461	12825	+	ENSSSCG00000047817	NA	lncRNA
16	54270001	54320000	0.388387	16	54303550	54305987	2438	+	ENSSSCG00000044222	NA	lncRNA
16	54280001	54330000	0.426473	16	54303550	54305987	2438	+	ENSSSCG00000044222	NA	lncRNA
16	54290001	54340000	0.378112	16	54303550	54305987	2438	+	ENSSSCG00000044222	NA	lncRNA
16	54450001	54500000	0.367601	16	54372873	54519530	146658	+	ENSSSCG00000041925	NA	lncRNA
16	54460001	54510000	0.366364	16	54372873	54519530	146658	+	ENSSSCG00000041925	NA	lncRNA
16	49510001	49560000	0.430194	16	49557731	49630412	72682	+	ENSSSCG00000043993	NA	lncRNA
16	49520001	49570000	0.43549	16	49557731	49630412	72682	+	ENSSSCG00000043993	NA	lncRNA
16	49530001	49580000	0.432365	16	49557731	49630412	72682	+	ENSSSCG00000043993	NA	lncRNA
16	49540001	49590000	0.436986	16	49557731	49630412	72682	+	ENSSSCG00000043993	NA	lncRNA
16	49550001	49600000	0.3924	16	49557731	49630412	72682	+	ENSSSCG00000043993	NA	lncRNA
16	50370001	50420000	0.459486	16	50411574	50423969	12396	+	ENSSSCG00000048792	NA	lncRNA
16	50380001	50430000	0.364146	16	50411574	50423969	12396	+	ENSSSCG00000048792	NA	lncRNA
16	50400001	50450000	0.354296	16	50411574	50423969	12396	+	ENSSSCG00000048792	NA	lncRNA
16	50410001	50460000	0.363066	16	50411574	50423969	12396	+	ENSSSCG00000048792	NA	lncRNA
16	50420001	50470000	0.380448	16	50411574	50423969	12396	+	ENSSSCG00000048792	NA	lncRNA
16	50580001	50630000	0.558347	16	50624355	50734346	109992	-	ENSSSCG00000043931	NA	lncRNA
16	50590001	50640000	0.537101	16	50624355	50734346	109992	-	ENSSSCG00000043931	NA	lncRNA
16	50600001	50650000	0.512078	16	50624355	50734346	109992	-	ENSSSCG00000043931	NA	lncRNA
16	50610001	50660000	0.534021	16	50624355	50734346	109992	-	ENSSSCG00000043931	NA	lncRNA
16	50620001	50670000	0.530474	16	50624355	50734346	109992	-	ENSSSCG00000043931	NA	lncRNA
16	50630001	50680000	0.561223	16	50624355	50734346	109992	-	ENSSSCG00000043931	NA	lncRNA
16	50640001	50690000	0.57756	16	50624355	50734346	109992	-	ENSSSCG00000043931	NA	lncRNA

16	50650001	50700000	0.595894	16	50624355	50734346	109992	-	ENSSSCG00000043931	NA	lncRNA
16	50660001	50710000	0.610016	16	50624355	50734346	109992	-	ENSSSCG00000043931	NA	lncRNA
16	50670001	50720000	0.617236	16	50624355	50734346	109992	-	ENSSSCG00000043931	NA	lncRNA
16	50680001	50730000	0.581546	16	50624355	50734346	109992	-	ENSSSCG00000043931	NA	lncRNA
16	50690001	50740000	0.525755	16	50624355	50734346	109992	-	ENSSSCG00000043931	NA	lncRNA
16	50700001	50750000	0.481302	16	50624355	50734346	109992	-	ENSSSCG00000043931	NA	lncRNA
16	50710001	50760000	0.459049	16	50624355	50734346	109992	-	ENSSSCG00000043931	NA	lncRNA
16	50720001	50770000	0.381445	16	50624355	50734346	109992	-	ENSSSCG00000043931	NA	lncRNA
16	26760001	26810000	0.374303	16	26809516	26813776	4261	+	ENSSSCG00000041879	NA	lncRNA
16	26770001	26820000	0.381186	16	26809516	26813776	4261	+	ENSSSCG00000041879	NA	lncRNA
16	26780001	26830000	0.368525	16	26809516	26813776	4261	+	ENSSSCG00000041879	NA	lncRNA
16	26790001	26840000	0.359121	16	26809516	26813776	4261	+	ENSSSCG00000041879	NA	lncRNA
16	26750001	26800000	0.359153	16	26707463	26752042	44580	+	ENSSSCG00000016865	FBXO4	protein_coding
16	48500001	48550000	0.376202	16	48482052	48642576	160525	-	ENSSSCG00000023818	MRPS27	protein_coding
16	48510001	48560000	0.425764	16	48482052	48642576	160525	-	ENSSSCG00000023818	MRPS27	protein_coding
16	48520001	48570000	0.50633	16	48482052	48642576	160525	-	ENSSSCG00000023818	MRPS27	protein_coding
16	48530001	48580000	0.525406	16	48482052	48642576	160525	-	ENSSSCG00000023818	MRPS27	protein_coding
16	48540001	48590000	0.526788	16	48482052	48642576	160525	-	ENSSSCG00000023818	MRPS27	protein_coding
16	48550001	48600000	0.535826	16	48482052	48642576	160525	-	ENSSSCG00000023818	MRPS27	protein_coding
16	48560001	48610000	0.506134	16	48482052	48642576	160525	-	ENSSSCG00000023818	MRPS27	protein_coding
16	48570001	48620000	0.477086	16	48482052	48642576	160525	-	ENSSSCG00000023818	MRPS27	protein_coding
16	48580001	48630000	0.479274	16	48482052	48642576	160525	-	ENSSSCG00000023818	MRPS27	protein_coding
16	48590001	48640000	0.461711	16	48482052	48642576	160525	-	ENSSSCG00000023818	MRPS27	protein_coding
16	48600001	48650000	0.419002	16	48482052	48642576	160525	-	ENSSSCG00000023818	MRPS27	protein_coding
16	48610001	48660000	0.393737	16	48482052	48642576	160525	-	ENSSSCG00000023818	MRPS27	protein_coding
16	48620001	48670000	0.370223	16	48482052	48642576	160525	-	ENSSSCG00000023818	MRPS27	protein_coding
16	48630001	48680000	0.386674	16	48482052	48642576	160525	-	ENSSSCG00000023818	MRPS27	protein_coding
16	48640001	48690000	0.388168	16	48482052	48642576	160525	-	ENSSSCG00000023818	MRPS27	protein_coding
16	48530001	48580000	0.525406	16	48575195	48580644	5450	+	ENSSSCG00000046085	NA	protein_coding
16	48540001	48590000	0.526788	16	48575195	48580644	5450	+	ENSSSCG00000046085	NA	protein_coding
16	48550001	48600000	0.535826	16	48575195	48580644	5450	+	ENSSSCG00000046085	NA	protein_coding
16	48560001	48610000	0.506134	16	48575195	48580644	5450	+	ENSSSCG00000046085	NA	protein_coding
16	48570001	48620000	0.477086	16	48575195	48580644	5450	+	ENSSSCG00000046085	NA	protein_coding
16	48580001	48630000	0.479274	16	48575195	48580644	5450	+	ENSSSCG00000046085	NA	protein_coding
16	48560001	48610000	0.506134	16	48604627	48636183	31557	+	ENSSSCG00000016975	PTCD2	protein_coding
16	48570001	48620000	0.477086	16	48604627	48636183	31557	+	ENSSSCG00000016975	PTCD2	protein_coding
16	48580001	48630000	0.479274	16	48604627	48636183	31557	+	ENSSSCG00000016975	PTCD2	protein_coding
16	48590001	48640000	0.461711	16	48604627	48636183	31557	+	ENSSSCG00000016975	PTCD2	protein_coding
16	48600001	48650000	0.419002	16	48604627	48636183	31557	+	ENSSSCG00000016975	PTCD2	protein_coding
16	48610001	48660000	0.393737	16	48604627	48636183	31557	+	ENSSSCG00000016975	PTCD2	protein_coding
16	48620001	48670000	0.370223	16	48604627	48636183	31557	+	ENSSSCG00000016975	PTCD2	protein_coding
16	48630001	48680000	0.386674	16	48604627	48636183	31557	+	ENSSSCG00000016975	PTCD2	protein_coding
16	48660001	48710000	0.371475	16	48708610	49048841	340232	-	ENSSSCG00000016976	ZNF366	protein_coding
16	48670001	48720000	0.383088	16	48708610	49048841	340232	-	ENSSSCG00000016976	ZNF366	protein_coding
16	48680001	48730000	0.380486	16	48708610	49048841	340232	-	ENSSSCG00000016976	ZNF366	protein_coding

16	48690001	48740000	0.375741	16	48708610	49048841	340232	-	ENSSSCG00000016976	ZNF366	protein_coding
16	48700001	48750000	0.382189	16	48708610	49048841	340232	-	ENSSSCG00000016976	ZNF366	protein_coding
16	48710001	48760000	0.384365	16	48708610	49048841	340232	-	ENSSSCG00000016976	ZNF366	protein_coding
16	48720001	48770000	0.372212	16	48708610	49048841	340232	-	ENSSSCG00000016976	ZNF366	protein_coding
16	48880001	48930000	0.352894	16	48708610	49048841	340232	-	ENSSSCG00000016976	ZNF366	protein_coding
16	48890001	48940000	0.371054	16	48708610	49048841	340232	-	ENSSSCG00000016976	ZNF366	protein_coding
16	48900001	48950000	0.362221	16	48708610	49048841	340232	-	ENSSSCG00000016976	ZNF366	protein_coding
16	48910001	48960000	0.353375	16	48708610	49048841	340232	-	ENSSSCG00000016976	ZNF366	protein_coding
16	48920001	48970000	0.364821	16	48708610	49048841	340232	-	ENSSSCG00000016976	ZNF366	protein_coding
16	48920001	48970000	0.364821	16	48960941	48974694	13754	+	ENSSSCG00000048350	NA	protein_coding
16	49720001	49770000	0.356251	16	49766897	49771634	4738	-	ENSSSCG00000034500	MSX2	protein_coding
16	50110001	50160000	0.606762	16	50158695	50160800	2106	+	ENSSSCG00000046456	NA	protein_coding
16	50120001	50170000	0.592027	16	50158695	50160800	2106	+	ENSSSCG00000046456	NA	protein_coding
16	50130001	50180000	0.588711	16	50158695	50160800	2106	+	ENSSSCG00000046456	NA	protein_coding
16	50140001	50190000	0.5889	16	50158695	50160800	2106	+	ENSSSCG00000046456	NA	protein_coding
16	50150001	50200000	0.597354	16	50158695	50160800	2106	+	ENSSSCG00000046456	NA	protein_coding
16	50160001	50210000	0.580381	16	50158695	50160800	2106	+	ENSSSCG00000046456	NA	protein_coding
16	50140001	50190000	0.5889	16	50186591	50189123	2533	-	ENSSSCG00000031466	NA	protein_coding
16	50150001	50200000	0.597354	16	50186591	50189123	2533	-	ENSSSCG00000031466	NA	protein_coding
16	50160001	50210000	0.580381	16	50186591	50189123	2533	-	ENSSSCG00000031466	NA	protein_coding
16	50170001	50220000	0.623493	16	50186591	50189123	2533	-	ENSSSCG00000031466	NA	protein_coding
16	50180001	50230000	0.661507	16	50186591	50189123	2533	-	ENSSSCG00000031466	NA	protein_coding
16	50160001	50210000	0.580381	16	50206076	50362213	156138	-	ENSSSCG00000016979	NSG2	protein_coding
16	50170001	50220000	0.623493	16	50206076	50362213	156138	-	ENSSSCG00000016979	NSG2	protein_coding
16	50180001	50230000	0.661507	16	50206076	50362213	156138	-	ENSSSCG00000016979	NSG2	protein_coding
16	50190001	50240000	0.652202	16	50206076	50362213	156138	-	ENSSSCG00000016979	NSG2	protein_coding
16	50200001	50250000	0.643325	16	50206076	50362213	156138	-	ENSSSCG00000016979	NSG2	protein_coding
16	50210001	50260000	0.629891	16	50206076	50362213	156138	-	ENSSSCG00000016979	NSG2	protein_coding
16	50220001	50270000	0.634796	16	50206076	50362213	156138	-	ENSSSCG00000016979	NSG2	protein_coding
16	50230001	50280000	0.544174	16	50206076	50362213	156138	-	ENSSSCG00000016979	NSG2	protein_coding
16	50240001	50290000	0.50303	16	50206076	50362213	156138	-	ENSSSCG00000016979	NSG2	protein_coding
16	50250001	50300000	0.43476	16	50206076	50362213	156138	-	ENSSSCG00000016979	NSG2	protein_coding
16	50260001	50310000	0.473145	16	50206076	50362213	156138	-	ENSSSCG00000016979	NSG2	protein_coding
16	50340001	50390000	0.391622	16	50206076	50362213	156138	-	ENSSSCG00000016979	NSG2	protein_coding
16	50350001	50400000	0.423085	16	50206076	50362213	156138	-	ENSSSCG00000016979	NSG2	protein_coding
16	50360001	50410000	0.491266	16	50206076	50362213	156138	-	ENSSSCG00000016979	NSG2	protein_coding
16	50340001	50390000	0.391622	16	50383767	50400163	16397	-	ENSSSCG00000035512	C5orf47	protein_coding
16	50350001	50400000	0.423085	16	50383767	50400163	16397	-	ENSSSCG00000035512	C5orf47	protein_coding
16	50360001	50410000	0.491266	16	50383767	50400163	16397	-	ENSSSCG00000035512	C5orf47	protein_coding
16	50370001	50420000	0.459486	16	50383767	50400163	16397	-	ENSSSCG00000035512	C5orf47	protein_coding
16	50380001	50430000	0.364146	16	50383767	50400163	16397	-	ENSSSCG00000035512	C5orf47	protein_coding
16	50400001	50450000	0.354296	16	50383767	50400163	16397	-	ENSSSCG00000035512	C5orf47	protein_coding
16	50380001	50430000	0.364146	16	50426816	50499065	72250	-	ENSSSCG00000016981	CPEB4	protein_coding
16	50400001	50450000	0.354296	16	50426816	50499065	72250	-	ENSSSCG00000016981	CPEB4	protein_coding
16	50410001	50460000	0.363066	16	50426816	50499065	72250	-	ENSSSCG00000016981	CPEB4	protein_coding

16	50420001	50470000	0.380448	16	50426816	50499065	72250	-	ENSSSCG00000016981	CPEB4	protein_coding
16	50430001	50480000	0.44937	16	50426816	50499065	72250	-	ENSSSCG00000016981	CPEB4	protein_coding
16	50440001	50490000	0.498237	16	50426816	50499065	72250	-	ENSSSCG00000016981	CPEB4	protein_coding
16	50450001	50500000	0.463001	16	50426816	50499065	72250	-	ENSSSCG00000016981	CPEB4	protein_coding
16	50460001	50510000	0.450361	16	50426816	50499065	72250	-	ENSSSCG00000016981	CPEB4	protein_coding
16	50470001	50520000	0.506233	16	50426816	50499065	72250	-	ENSSSCG00000016981	CPEB4	protein_coding
16	50480001	50530000	0.579487	16	50426816	50499065	72250	-	ENSSSCG00000016981	CPEB4	protein_coding
16	50490001	50540000	0.611117	16	50426816	50499065	72250	-	ENSSSCG00000016981	CPEB4	protein_coding
16	50460001	50510000	0.450361	16	50504971	50511438	6468	+	ENSSSCG00000049075	NA	pseudogene
16	50470001	50520000	0.506233	16	50504971	50511438	6468	+	ENSSSCG00000049075	NA	pseudogene
16	50480001	50530000	0.579487	16	50504971	50511438	6468	+	ENSSSCG00000049075	NA	pseudogene
16	50490001	50540000	0.611117	16	50504971	50511438	6468	+	ENSSSCG00000049075	NA	pseudogene
16	50500001	50550000	0.618874	16	50504971	50511438	6468	+	ENSSSCG00000049075	NA	pseudogene
16	50510001	50560000	0.623924	16	50504971	50511438	6468	+	ENSSSCG00000049075	NA	pseudogene
16	50670001	50720000	0.617236	16	50711667	50716710	5044	-	ENSSSCG00000050306	NA	pseudogene
16	50680001	50730000	0.581546	16	50711667	50716710	5044	-	ENSSSCG00000050306	NA	pseudogene
16	50690001	50740000	0.525755	16	50711667	50716710	5044	-	ENSSSCG00000050306	NA	pseudogene
16	50700001	50750000	0.481302	16	50711667	50716710	5044	-	ENSSSCG00000050306	NA	pseudogene
16	50710001	50760000	0.459049	16	50711667	50716710	5044	-	ENSSSCG00000050306	NA	pseudogene
16	50690001	50740000	0.525755	16	50734440	50743414	8975	+	ENSSSCG00000016982	NA	protein_coding
16	50700001	50750000	0.481302	16	50734440	50743414	8975	+	ENSSSCG00000016982	NA	protein_coding
16	50710001	50760000	0.459049	16	50734440	50743414	8975	+	ENSSSCG00000016982	NA	protein_coding
16	50720001	50770000	0.381445	16	50734440	50743414	8975	+	ENSSSCG00000016982	NA	protein_coding
16	50940001	50990000	0.489762	16	50989432	51004484	15053	+	ENSSSCG00000016983	STC2	protein_coding
16	50950001	51000000	0.513872	16	50989432	51004484	15053	+	ENSSSCG00000016983	STC2	protein_coding
16	50960001	51010000	0.459745	16	50989432	51004484	15053	+	ENSSSCG00000016983	STC2	protein_coding
16	50970001	51020000	0.422944	16	50989432	51004484	15053	+	ENSSSCG00000016983	STC2	protein_coding
16	50980001	51030000	0.377498	16	50989432	51004484	15053	+	ENSSSCG00000016983	STC2	protein_coding
16	50990001	51040000	0.398331	16	50989432	51004484	15053	+	ENSSSCG00000016983	STC2	protein_coding
16	51000001	51050000	0.403999	16	50989432	51004484	15053	+	ENSSSCG00000016983	STC2	protein_coding
16	51040001	51090000	0.407665	16	51086011	51089165	3155	+	ENSSSCG00000036285	NKX2-5	protein_coding
16	51050001	51100000	0.372637	16	51086011	51089165	3155	+	ENSSSCG00000036285	NKX2-5	protein_coding
16	51330001	51380000	0.391774	16	51267588	51395039	127452	-	ENSSSCG00000016989	RPL26L1	protein_coding
16	51340001	51390000	0.35291	16	51267588	51395039	127452	-	ENSSSCG00000016989	RPL26L1	protein_coding
16	51330001	51380000	0.391774	16	51283272	51403520	120249	-	ENSSSCG00000016988	ERGIC1	protein_coding
16	51340001	51390000	0.35291	16	51283272	51403520	120249	-	ENSSSCG00000016988	ERGIC1	protein_coding
16	51700001	51750000	0.373765	16	51733131	51858772	125642	+	ENSSSCG00000027378	SH3PXD2B	protein_coding
16	51710001	51760000	0.385899	16	51733131	51858772	125642	+	ENSSSCG00000027378	SH3PXD2B	protein_coding
16	51720001	51770000	0.39093	16	51733131	51858772	125642	+	ENSSSCG00000027378	SH3PXD2B	protein_coding
16	52030001	52080000	0.353627	16	52003735	52166161	162427	+	ENSSSCG00000022478	STK10	protein_coding
16	52200001	52250000	0.39686	16	52212655	52337913	125259	+	ENSSSCG00000023307	FBXW11	protein_coding
16	52210001	52260000	0.365407	16	52212655	52337913	125259	+	ENSSSCG00000023307	FBXW11	protein_coding
16	52220001	52270000	0.370548	16	52212655	52337913	125259	+	ENSSSCG00000023307	FBXW11	protein_coding
16	52230001	52280000	0.373242	16	52212655	52337913	125259	+	ENSSSCG00000023307	FBXW11	protein_coding
16	52240001	52290000	0.372157	16	52212655	52337913	125259	+	ENSSSCG00000023307	FBXW11	protein_coding

16	52250001	52300000	0.363341	16	52212655	52337913	125259	+	ENSSSCG00000023307	FBXW11	protein_coding
16	52260001	52310000	0.385654	16	52212655	52337913	125259	+	ENSSSCG00000023307	FBXW11	protein_coding
16	52270001	52320000	0.374701	16	52212655	52337913	125259	+	ENSSSCG00000023307	FBXW11	protein_coding
16	52320001	52370000	0.37859	16	52212655	52337913	125259	+	ENSSSCG00000023307	FBXW11	protein_coding
16	52330001	52380000	0.403737	16	52212655	52337913	125259	+	ENSSSCG00000023307	FBXW11	protein_coding
16	52220001	52270000	0.370548	16	52264386	52265157	772	+	ENSSSCG00000023757	NA	protein_coding
16	52230001	52280000	0.373242	16	52264386	52265157	772	+	ENSSSCG00000023757	NA	protein_coding
16	52240001	52290000	0.372157	16	52264386	52265157	772	+	ENSSSCG00000023757	NA	protein_coding
16	52250001	52300000	0.363341	16	52264386	52265157	772	+	ENSSSCG00000023757	NA	protein_coding
16	52260001	52310000	0.385654	16	52264386	52265157	772	+	ENSSSCG00000023757	NA	protein_coding
16	52360001	52410000	0.453246	16	52404004	52412792	8789	-	ENSSSCG00000044666	SMIM23	protein_coding
16	52370001	52420000	0.412508	16	52404004	52412792	8789	-	ENSSSCG00000044666	SMIM23	protein_coding
16	52380001	52430000	0.372595	16	52404004	52412792	8789	-	ENSSSCG00000044666	SMIM23	protein_coding
16	52390001	52440000	0.383553	16	52404004	52412792	8789	-	ENSSSCG00000044666	SMIM23	protein_coding
16	52410001	52460000	0.365894	16	52404004	52412792	8789	-	ENSSSCG00000044666	SMIM23	protein_coding
16	53210001	53260000	0.358568	16	53203887	53228718	24832	-	ENSSSCG00000021902	GABRP	protein_coding
16	53220001	53270000	0.386576	16	53203887	53228718	24832	-	ENSSSCG00000021902	GABRP	protein_coding
16	53220001	53270000	0.386576	16	53267523	53650721	383199	-	ENSSSCG00000017003	KCNIP1	protein_coding
16	53230001	53280000	0.405312	16	53267523	53650721	383199	-	ENSSSCG00000017003	KCNIP1	protein_coding
16	53240001	53290000	0.390913	16	53267523	53650721	383199	-	ENSSSCG00000017003	KCNIP1	protein_coding
16	53250001	53300000	0.410639	16	53267523	53650721	383199	-	ENSSSCG00000017003	KCNIP1	protein_coding
16	53260001	53310000	0.358241	16	53267523	53650721	383199	-	ENSSSCG00000017003	KCNIP1	protein_coding
16	54020001	54070000	0.393103	16	53843248	54248638	405391	-	ENSSSCG00000017008	DOCK2	protein_coding
16	54030001	54080000	0.460068	16	53843248	54248638	405391	-	ENSSSCG00000017008	DOCK2	protein_coding
16	54040001	54090000	0.459978	16	53843248	54248638	405391	-	ENSSSCG00000017008	DOCK2	protein_coding
16	54050001	54100000	0.399679	16	53843248	54248638	405391	-	ENSSSCG00000017008	DOCK2	protein_coding
16	54020001	54070000	0.393103	16	53941568	54057671	116104	+	ENSSSCG00000017010	INSYN2B	protein_coding
16	54030001	54080000	0.460068	16	53941568	54057671	116104	+	ENSSSCG00000017010	INSYN2B	protein_coding
16	54040001	54090000	0.459978	16	53941568	54057671	116104	+	ENSSSCG00000017010	INSYN2B	protein_coding
16	54050001	54100000	0.399679	16	53941568	54057671	116104	+	ENSSSCG00000017010	INSYN2B	protein_coding
16	54270001	54320000	0.388387	16	54254227	54303544	49318	-	ENSSSCG00000025965	SPDL1	protein_coding
16	54280001	54330000	0.426473	16	54254227	54303544	49318	-	ENSSSCG00000025965	SPDL1	protein_coding
16	54290001	54340000	0.378112	16	54254227	54303544	49318	-	ENSSSCG00000025965	SPDL1	protein_coding
4	64350001	64400000	0.410386	4	64393933	64394021	89	-	ENSSSCG00000051781	NA	snoRNA
4	64360001	64410000	0.459059	4	64393933	64394021	89	-	ENSSSCG00000051781	NA	snoRNA
4	64370001	64420000	0.477647	4	64393933	64394021	89	-	ENSSSCG00000051781	NA	snoRNA
4	64380001	64430000	0.438988	4	64393933	64394021	89	-	ENSSSCG00000051781	NA	snoRNA
4	64390001	64440000	0.35721	4	64393933	64394021	89	-	ENSSSCG00000051781	NA	snoRNA
4	128800001	128850000	0.352649	4	128823397	128828344	4948	-	ENSSSCG00000042877	NA	lncRNA
4	128800001	128850000	0.352649	4	128834275	128843545	9271	-	ENSSSCG00000046427	NA	lncRNA
4	43330001	43380000	0.402126	4	43368034	43394583	26550	+	ENSSSCG00000036325	NA	lncRNA
4	43340001	43390000	0.427681	4	43368034	43394583	26550	+	ENSSSCG00000036325	NA	lncRNA
4	43350001	43400000	0.402518	4	43368034	43394583	26550	+	ENSSSCG00000036325	NA	lncRNA
4	43360001	43410000	0.421463	4	43368034	43394583	26550	+	ENSSSCG00000036325	NA	lncRNA
4	43370001	43420000	0.396767	4	43368034	43394583	26550	+	ENSSSCG00000036325	NA	lncRNA

4	43380001	43430000	0.375809	4	43368034	43394583	26550	+	ENSSSCG00000036325	NA	lncRNA
4	43390001	43440000	0.368418	4	43368034	43394583	26550	+	ENSSSCG00000036325	NA	lncRNA
4	88850001	88900000	0.380191	4	88845460	88855286	9827	-	ENSSSCG00000030246	FCRLA	protein_coding
4	88850001	88900000	0.380191	4	88873762	88989769	116008	-	ENSSSCG00000006350	NA	protein_coding
4	88860001	88910000	0.403109	4	88873762	88989769	116008	-	ENSSSCG00000006350	NA	protein_coding
4	88870001	88920000	0.386601	4	88873762	88989769	116008	-	ENSSSCG00000006350	NA	protein_coding
4	88880001	88930000	0.377281	4	88873762	88989769	116008	-	ENSSSCG00000006350	NA	protein_coding
4	88890001	88940000	0.359855	4	88873762	88989769	116008	-	ENSSSCG00000006350	NA	protein_coding
4	43210001	43260000	0.41466	4	43252453	43255906	3454	+	ENSSSCG00000047712	NA	lncRNA
4	43220001	43270000	0.404618	4	43252453	43255906	3454	+	ENSSSCG00000047712	NA	lncRNA
4	43230001	43280000	0.39642	4	43252453	43255906	3454	+	ENSSSCG00000047712	NA	lncRNA
4	43240001	43290000	0.382181	4	43252453	43255906	3454	+	ENSSSCG00000047712	NA	lncRNA
4	43250001	43300000	0.392383	4	43252453	43255906	3454	+	ENSSSCG00000047712	NA	lncRNA
4	43330001	43380000	0.402126	4	43319249	43331148	11900	-	ENSSSCG00000050887	NA	lncRNA
4	82620001	82670000	0.372728	4	82632779	82642274	9496	-	ENSSSCG00000044380	NA	lncRNA
4	82630001	82680000	0.415166	4	82632779	82642274	9496	-	ENSSSCG00000044380	NA	lncRNA
4	82640001	82690000	0.525871	4	82632779	82642274	9496	-	ENSSSCG00000044380	NA	lncRNA
4	82620001	82670000	0.372728	4	82647773	82650675	2903	-	ENSSSCG00000043420	NA	lncRNA
4	82630001	82680000	0.415166	4	82647773	82650675	2903	-	ENSSSCG00000043420	NA	lncRNA
4	82640001	82690000	0.525871	4	82647773	82650675	2903	-	ENSSSCG00000043420	NA	lncRNA
4	82650001	82700000	0.578011	4	82647773	82650675	2903	-	ENSSSCG00000043420	NA	lncRNA
4	89870001	89920000	0.379598	4	89870539	89908244	37706	+	ENSSSCG00000006380	SLAMF1	protein_coding
4	89880001	89930000	0.360064	4	89870539	89908244	37706	+	ENSSSCG00000006380	SLAMF1	protein_coding
4	89890001	89940000	0.372055	4	89870539	89908244	37706	+	ENSSSCG00000006380	SLAMF1	protein_coding
4	89900001	89950000	0.362552	4	89870539	89908244	37706	+	ENSSSCG00000006380	SLAMF1	protein_coding
4	89890001	89940000	0.372055	4	89932202	89969666	37465	+	ENSSSCG00000006381	CD84	protein_coding
4	89900001	89950000	0.362552	4	89932202	89969666	37465	+	ENSSSCG00000006381	CD84	protein_coding
4	90210001	90260000	0.37459	4	90197088	90261551	64464	+	ENSSSCG00000006388	DCAF8	protein_coding
4	90220001	90270000	0.388497	4	90197088	90261551	64464	+	ENSSSCG00000006388	DCAF8	protein_coding
4	90220001	90270000	0.388497	4	90263584	90274144	10561	-	ENSSSCG00000032164	PEA15	protein_coding
4	60990001	61040000	0.364535	4	61037373	61042286	4914	+	ENSSSCG00000045745	NA	protein_coding
4	61010001	61060000	0.361118	4	61037373	61042286	4914	+	ENSSSCG00000045745	NA	protein_coding
4	24840001	24890000	0.389265	4	24648199	25870858	1222660	+	ENSSSCG00000031031	CSMD3	protein_coding
4	24850001	24900000	0.45651	4	24648199	25870858	1222660	+	ENSSSCG00000031031	CSMD3	protein_coding
4	24860001	24910000	0.499994	4	24648199	25870858	1222660	+	ENSSSCG00000031031	CSMD3	protein_coding
4	24870001	24920000	0.509821	4	24648199	25870858	1222660	+	ENSSSCG00000031031	CSMD3	protein_coding
4	24880001	24930000	0.519896	4	24648199	25870858	1222660	+	ENSSSCG00000031031	CSMD3	protein_coding
4	24890001	24940000	0.514359	4	24648199	25870858	1222660	+	ENSSSCG00000031031	CSMD3	protein_coding
4	24900001	24950000	0.491925	4	24648199	25870858	1222660	+	ENSSSCG00000031031	CSMD3	protein_coding
4	24910001	24960000	0.433337	4	24648199	25870858	1222660	+	ENSSSCG00000031031	CSMD3	protein_coding
4	24920001	24970000	0.373462	4	24648199	25870858	1222660	+	ENSSSCG00000031031	CSMD3	protein_coding
4	24960001	25010000	0.355797	4	24648199	25870858	1222660	+	ENSSSCG00000031031	CSMD3	protein_coding
4	24970001	25020000	0.359137	4	24648199	25870858	1222660	+	ENSSSCG00000031031	CSMD3	protein_coding
4	88880001	88930000	0.377281	4	88922098	88923415	1318	+	ENSSSCG00000043911	NA	protein_coding
4	88890001	88940000	0.359855	4	88922098	88923415	1318	+	ENSSSCG00000043911	NA	protein_coding

4	36310001	36360000	0.35594	4	36303501	36388952	85452	+	ENSSSCG00000006064	SNX31	protein_coding
4	36310001	36360000	0.35594	4	36303502	36444335	140834	+	ENSSSCG00000006065	ANKRD46	protein_coding
4	36880001	36930000	0.371209	4	36856937	37042908	185972	+	ENSSSCG00000006069	RGS22	protein_coding
4	36890001	36940000	0.370171	4	36856937	37042908	185972	+	ENSSSCG00000006069	RGS22	protein_coding
4	36880001	36930000	0.371209	4	36917760	36918263	504	-	ENSSSCG00000031418	NA	protein_coding
4	36890001	36940000	0.370171	4	36917760	36918263	504	-	ENSSSCG00000031418	NA	protein_coding
4	42920001	42970000	0.392593	4	42878254	42931121	52868	-	ENSSSCG00000006108	TMEM67	protein_coding
4	42930001	42980000	0.365078	4	42878254	42931121	52868	-	ENSSSCG00000006108	TMEM67	protein_coding
4	42920001	42970000	0.392593	4	42946778	42969356	22579	+	ENSSSCG00000006110	RBM12B	protein_coding
4	42930001	42980000	0.365078	4	42946778	42969356	22579	+	ENSSSCG00000006110	RBM12B	protein_coding
4	42920001	42970000	0.392593	4	42957712	42985459	27748	-	ENSSSCG00000037963	CIBAR1	protein_coding
4	42930001	42980000	0.365078	4	42957712	42985459	27748	-	ENSSSCG00000037963	CIBAR1	protein_coding
4	43190001	43240000	0.417364	4	43235326	43239144	3819	-	ENSSSCG00000047343	NA	protein_coding
4	43200001	43250000	0.404312	4	43235326	43239144	3819	-	ENSSSCG00000047343	NA	protein_coding
4	43210001	43260000	0.41466	4	43235326	43239144	3819	-	ENSSSCG00000047343	NA	protein_coding
4	43220001	43270000	0.404618	4	43235326	43239144	3819	-	ENSSSCG00000047343	NA	protein_coding
4	43230001	43280000	0.39642	4	43235326	43239144	3819	-	ENSSSCG00000047343	NA	protein_coding
4	43660001	43710000	0.359406	4	43707902	43793419	85518	+	ENSSSCG00000037733	TRIQK	protein_coding
4	43670001	43720000	0.377146	4	43707902	43793419	85518	+	ENSSSCG00000037733	TRIQK	protein_coding
4	43680001	43730000	0.37571	4	43707902	43793419	85518	+	ENSSSCG00000037733	TRIQK	protein_coding
4	43690001	43740000	0.409914	4	43707902	43793419	85518	+	ENSSSCG00000037733	TRIQK	protein_coding
4	43700001	43750000	0.39842	4	43707902	43793419	85518	+	ENSSSCG00000037733	TRIQK	protein_coding
4	43710001	43760000	0.359092	4	43707902	43793419	85518	+	ENSSSCG00000037733	TRIQK	protein_coding
4	43720001	43770000	0.356521	4	43707902	43793419	85518	+	ENSSSCG00000037733	TRIQK	protein_coding
4	43730001	43780000	0.357178	4	43707902	43793419	85518	+	ENSSSCG00000037733	TRIQK	protein_coding
4	43740001	43790000	0.362636	4	43707902	43793419	85518	+	ENSSSCG00000037733	TRIQK	protein_coding
4	43750001	43800000	0.386356	4	43707902	43793419	85518	+	ENSSSCG00000037733	TRIQK	protein_coding
4	43760001	43810000	0.377701	4	43707902	43793419	85518	+	ENSSSCG00000037733	TRIQK	protein_coding
4	50330001	50380000	0.360171	4	50276417	50345369	68953	-	ENSSSCG00000006135	CPNE3	protein_coding
4	50340001	50390000	0.384254	4	50276417	50345369	68953	-	ENSSSCG00000006135	CPNE3	protein_coding
4	50330001	50380000	0.360171	4	50344893	50389087	44195	+	ENSSSCG00000006136	RMDN1	protein_coding
4	50340001	50390000	0.384254	4	50344893	50389087	44195	+	ENSSSCG00000006136	RMDN1	protein_coding
4	50350001	50400000	0.357777	4	50344893	50389087	44195	+	ENSSSCG00000006136	RMDN1	protein_coding
4	50330001	50380000	0.360171	4	50370088	50509113	139026	-	ENSSSCG00000006137	WWP1	protein_coding
4	50340001	50390000	0.384254	4	50370088	50509113	139026	-	ENSSSCG00000006137	WWP1	protein_coding
4	50350001	50400000	0.357777	4	50370088	50509113	139026	-	ENSSSCG00000006137	WWP1	protein_coding
4	50640001	50690000	0.432743	4	50614313	50642340	28028	+	ENSSSCG00000022935	SLC7A13	protein_coding
4	110320001	110370000	0.365475	4	110311507	110329568	18062	+	ENSSSCG00000006820	EPS8L3	protein_coding
4	110320001	110370000	0.365475	4	110330028	110332962	2935	+	ENSSSCG00000006821	GSTM3	protein_coding
4	110330001	110380000	0.387774	4	110330028	110332962	2935	+	ENSSSCG00000006821	GSTM3	protein_coding
4	110330001	110380000	0.387774	4	110378017	110486771	108755	-	ENSSSCG00000037808	NA	protein_coding
4	110340001	110390000	0.407357	4	110378017	110486771	108755	-	ENSSSCG00000037808	NA	protein_coding
4	110350001	110400000	0.380206	4	110378017	110486771	108755	-	ENSSSCG00000037808	NA	protein_coding
4	60980001	61030000	0.374905	4	60948329	60998368	50040	-	ENSSSCG00000006171	CRISPLD1	protein_coding
4	60990001	61040000	0.364535	4	60948329	60998368	50040	-	ENSSSCG00000006171	CRISPLD1	protein_coding

4	62020001	62070000	0.372647	4	62014257	62098402	84146	+	ENSSSCG00000006177	UBE2W	protein_coding
4	62030001	62080000	0.37025	4	62014257	62098402	84146	+	ENSSSCG00000006177	UBE2W	protein_coding
4	62040001	62090000	0.362481	4	62014257	62098402	84146	+	ENSSSCG00000006177	UBE2W	protein_coding
4	62850001	62900000	0.372188	4	62880267	63277761	397495	-	ENSSSCG00000006185	KCNB2	protein_coding
4	62860001	62910000	0.366854	4	62880267	63277761	397495	-	ENSSSCG00000006185	KCNB2	protein_coding
4	64680001	64730000	0.373575	4	64679170	64707710	28541	-	ENSSSCG00000006191	XKR9	protein_coding
4	64690001	64740000	0.369832	4	64679170	64707710	28541	-	ENSSSCG00000006191	XKR9	protein_coding
4	64680001	64730000	0.373575	4	64710771	64748936	38166	+	ENSSSCG00000006192	LACTB2	protein_coding
4	64690001	64740000	0.369832	4	64710771	64748936	38166	+	ENSSSCG00000006192	LACTB2	protein_coding
4	64810001	64860000	0.35899	4	64749244	64836126	86883	+	ENSSSCG00000006193	TRAM1	protein_coding
4	64820001	64870000	0.364367	4	64749244	64836126	86883	+	ENSSSCG00000006193	TRAM1	protein_coding
4	64830001	64880000	0.363511	4	64749244	64836126	86883	+	ENSSSCG00000006193	TRAM1	protein_coding
4	64910001	64960000	0.352916	4	64954120	65246572	292453	+	ENSSSCG00000006194	NCOA2	protein_coding
4	64920001	64970000	0.362322	4	64954120	65246572	292453	+	ENSSSCG00000006194	NCOA2	protein_coding
4	64930001	64980000	0.374148	4	64954120	65246572	292453	+	ENSSSCG00000006194	NCOA2	protein_coding
4	64940001	64990000	0.421363	4	64954120	65246572	292453	+	ENSSSCG00000006194	NCOA2	protein_coding
4	64950001	65000000	0.43011	4	64954120	65246572	292453	+	ENSSSCG00000006194	NCOA2	protein_coding
4	64960001	65010000	0.398414	4	64954120	65246572	292453	+	ENSSSCG00000006194	NCOA2	protein_coding
4	64970001	65020000	0.362483	4	64954120	65246572	292453	+	ENSSSCG00000006194	NCOA2	protein_coding
4	67760001	67810000	0.36069	4	67613640	67844337	230698	-	ENSSSCG00000006202	CSPP1	protein_coding
4	67770001	67820000	0.36109	4	67613640	67844337	230698	-	ENSSSCG00000006202	CSPP1	protein_coding
4	67760001	67810000	0.36069	4	67772806	67815977	43172	+	ENSSSCG00000006204	PPP1R42	protein_coding
4	67770001	67820000	0.36109	4	67772806	67815977	43172	+	ENSSSCG00000006204	PPP1R42	protein_coding
4	67770001	67820000	0.36109	4	67816760	67826257	9498	+	ENSSSCG00000049935	TCF24	protein_coding
4	82620001	82670000	0.372728	4	82663581	82697813	34233	-	ENSSSCG00000006299	TBX19	protein_coding
4	82630001	82680000	0.415166	4	82663581	82697813	34233	-	ENSSSCG00000006299	TBX19	protein_coding
4	82640001	82690000	0.525871	4	82663581	82697813	34233	-	ENSSSCG00000006299	TBX19	protein_coding
4	82650001	82700000	0.578011	4	82663581	82697813	34233	-	ENSSSCG00000006299	TBX19	protein_coding
4	82660001	82710000	0.60787	4	82663581	82697813	34233	-	ENSSSCG00000006299	TBX19	protein_coding
4	82670001	82720000	0.646961	4	82663581	82697813	34233	-	ENSSSCG00000006299	TBX19	protein_coding
4	82680001	82730000	0.647939	4	82663581	82697813	34233	-	ENSSSCG00000006299	TBX19	protein_coding
4	82690001	82740000	0.641769	4	82663581	82697813	34233	-	ENSSSCG00000006299	TBX19	protein_coding
4	82670001	82720000	0.646961	4	82712896	82734487	21592	-	ENSSSCG00000031617	SFT2D2	protein_coding
4	82680001	82730000	0.647939	4	82712896	82734487	21592	-	ENSSSCG00000031617	SFT2D2	protein_coding
4	82690001	82740000	0.641769	4	82712896	82734487	21592	-	ENSSSCG00000031617	SFT2D2	protein_coding
4	82700001	82750000	0.603501	4	82712896	82734487	21592	-	ENSSSCG00000031617	SFT2D2	protein_coding
4	82710001	82760000	0.525518	4	82712896	82734487	21592	-	ENSSSCG00000031617	SFT2D2	protein_coding
4	82720001	82770000	0.452718	4	82712896	82734487	21592	-	ENSSSCG00000031617	SFT2D2	protein_coding
4	82730001	82780000	0.365668	4	82712896	82734487	21592	-	ENSSSCG00000031617	SFT2D2	protein_coding
4	82710001	82760000	0.525518	4	82756044	82794715	38672	-	ENSSSCG00000006301	TIPRL	protein_coding
4	82720001	82770000	0.452718	4	82756044	82794715	38672	-	ENSSSCG00000006301	TIPRL	protein_coding
4	82730001	82780000	0.365668	4	82756044	82794715	38672	-	ENSSSCG00000006301	TIPRL	protein_coding
4	83270001	83320000	0.359672	4	83235470	83316405	80936	-	ENSSSCG00000006306	MPZL1	protein_coding
4	83280001	83330000	0.372031	4	83235470	83316405	80936	-	ENSSSCG00000006306	MPZL1	protein_coding
4	83290001	83340000	0.366084	4	83235470	83316405	80936	-	ENSSSCG00000006306	MPZL1	protein_coding

4	83280001	83330000	0.372031	4	83322208	83404102	81895	-	ENSSSCG00000006307	RCSD1	protein_coding
4	83290001	83340000	0.366084	4	83322208	83404102	81895	-	ENSSSCG00000006307	RCSD1	protein_coding
4	83950001	84000000	0.369065	4	83975347	84040709	65363	-	ENSSSCG00000006316	MAEL	protein_coding
4	83960001	84010000	0.486643	4	83975347	84040709	65363	-	ENSSSCG00000006316	MAEL	protein_coding
4	83970001	84020000	0.517642	4	83975347	84040709	65363	-	ENSSSCG00000006316	MAEL	protein_coding
4	83980001	84030000	0.456035	4	83975347	84040709	65363	-	ENSSSCG00000006316	MAEL	protein_coding
4	83990001	84040000	0.366069	4	83975347	84040709	65363	-	ENSSSCG00000006316	MAEL	protein_coding
4	84650001	84700000	0.353698	4	84597425	84700186	102762	+	ENSSSCG00000006321	FAM78B	protein_coding
8	51300001	51350000	0.409131	8	51344286	51344401	116	-	ENSSSCG00000018686	NA	snoRNA
8	51310001	51360000	0.368028	8	51344286	51344401	116	-	ENSSSCG00000018686	NA	snoRNA
8	13150001	13200000	0.365462	8	13129486	13170938	41453	-	ENSSSCG00000045236	NA	lncRNA
8	13160001	13210000	0.379288	8	13129486	13170938	41453	-	ENSSSCG00000045236	NA	lncRNA
8	13170001	13220000	0.363258	8	13129486	13170938	41453	-	ENSSSCG00000045236	NA	lncRNA
8	42560001	42610000	0.42698	8	42606611	42611079	4469	+	ENSSSCG00000042370	NA	protein_coding
8	42570001	42620000	0.422803	8	42606611	42611079	4469	+	ENSSSCG00000042370	NA	protein_coding
8	42580001	42630000	0.399994	8	42606611	42611079	4469	+	ENSSSCG00000042370	NA	protein_coding
8	42560001	42610000	0.42698	8	42609670	42611120	1451	-	ENSSSCG00000050455	NA	protein_coding
8	42570001	42620000	0.422803	8	42609670	42611120	1451	-	ENSSSCG00000050455	NA	protein_coding
8	42580001	42630000	0.399994	8	42609670	42611120	1451	-	ENSSSCG00000050455	NA	protein_coding
8	57350001	57400000	0.361224	8	57393054	57397483	4430	+	ENSSSCG00000048199	NA	protein_coding
8	57360001	57410000	0.35897	8	57393054	57397483	4430	+	ENSSSCG00000048199	NA	protein_coding
8	57370001	57420000	0.376255	8	57393054	57397483	4430	+	ENSSSCG00000048199	NA	protein_coding
8	57380001	57430000	0.427558	8	57393054	57397483	4430	+	ENSSSCG00000048199	NA	protein_coding
8	57390001	57440000	0.483446	8	57393054	57397483	4430	+	ENSSSCG00000048199	NA	protein_coding
8	40690001	40740000	0.397713	8	40738206	40741480	3275	+	ENSSSCG00000044823	NA	lncRNA
8	40700001	40750000	0.394751	8	40738206	40741480	3275	+	ENSSSCG00000044823	NA	lncRNA
8	40710001	40760000	0.368824	8	40738206	40741480	3275	+	ENSSSCG00000044823	NA	lncRNA
8	41850001	41900000	0.359875	8	41897748	41913738	15991	+	ENSSSCG00000043179	NA	lncRNA
8	42560001	42610000	0.42698	8	42603559	42606458	2900	+	ENSSSCG00000045149	NA	lncRNA
8	42570001	42620000	0.422803	8	42603559	42606458	2900	+	ENSSSCG00000045149	NA	lncRNA
8	42580001	42630000	0.399994	8	42603559	42606458	2900	+	ENSSSCG00000045149	NA	lncRNA
8	46820001	46870000	0.511333	8	46865919	46919757	53839	+	ENSSSCG00000039268	NA	lncRNA
8	46830001	46880000	0.511051	8	46865919	46919757	53839	+	ENSSSCG00000039268	NA	lncRNA
8	46840001	46890000	0.511434	8	46865919	46919757	53839	+	ENSSSCG00000039268	NA	lncRNA
8	46850001	46900000	0.469521	8	46865919	46919757	53839	+	ENSSSCG00000039268	NA	lncRNA
8	46860001	46910000	0.428737	8	46865919	46919757	53839	+	ENSSSCG00000039268	NA	lncRNA
8	52310001	52360000	0.468372	8	52339436	52342962	3527	+	ENSSSCG00000047648	NA	lncRNA
8	52320001	52370000	0.485628	8	52339436	52342962	3527	+	ENSSSCG00000047648	NA	lncRNA
8	52330001	52380000	0.574129	8	52339436	52342962	3527	+	ENSSSCG00000047648	NA	lncRNA
8	52340001	52390000	0.597283	8	52339436	52342962	3527	+	ENSSSCG00000047648	NA	lncRNA
8	52310001	52360000	0.468372	8	52339991	52343138	3148	-	ENSSSCG00000045390	NA	lncRNA
8	52320001	52370000	0.485628	8	52339991	52343138	3148	-	ENSSSCG00000045390	NA	lncRNA
8	52330001	52380000	0.574129	8	52339991	52343138	3148	-	ENSSSCG00000045390	NA	lncRNA
8	52340001	52390000	0.597283	8	52339991	52343138	3148	-	ENSSSCG00000045390	NA	lncRNA
8	55030001	55080000	0.370401	8	55075027	55093709	18683	+	ENSSSCG00000042303	NA	lncRNA

8	55040001	55090000	0.360416	8	55075027	55093709	18683	+	ENSSSCG00000042303	NA	lncRNA
8	55820001	55870000	0.392897	8	55833306	55842939	9634	+	ENSSSCG00000044724	NA	lncRNA
8	55830001	55880000	0.403956	8	55833306	55842939	9634	+	ENSSSCG00000044724	NA	lncRNA
8	55840001	55890000	0.369372	8	55833306	55842939	9634	+	ENSSSCG00000044724	NA	lncRNA
8	56360001	56410000	0.389391	8	56405430	56406970	1541	-	ENSSSCG00000050886	NA	lncRNA
8	114300001	114350000	0.375538	8	114251528	114373427	121900	+	ENSSSCG00000031901	PAPSS1	protein_coding
8	114310001	114360000	0.4012	8	114251528	114373427	121900	+	ENSSSCG00000031901	PAPSS1	protein_coding
8	114320001	114370000	0.414636	8	114251528	114373427	121900	+	ENSSSCG00000031901	PAPSS1	protein_coding
8	114330001	114380000	0.396985	8	114251528	114373427	121900	+	ENSSSCG00000031901	PAPSS1	protein_coding
8	114340001	114390000	0.360405	8	114251528	114373427	121900	+	ENSSSCG00000031901	PAPSS1	protein_coding
8	51410001	51460000	0.371014	8	51458021	51462826	4806	+	ENSSSCG00000050575	NA	protein_coding
8	51420001	51470000	0.398169	8	51458021	51462826	4806	+	ENSSSCG00000050575	NA	protein_coding
8	51430001	51480000	0.434967	8	51458021	51462826	4806	+	ENSSSCG00000050575	NA	protein_coding
8	51440001	51490000	0.489493	8	51458021	51462826	4806	+	ENSSSCG00000050575	NA	protein_coding
8	51450001	51500000	0.47883	8	51458021	51462826	4806	+	ENSSSCG00000050575	NA	protein_coding
8	51460001	51510000	0.466838	8	51458021	51462826	4806	+	ENSSSCG00000050575	NA	protein_coding
8	115960001	116010000	0.357793	8	115902991	116031863	128873	-	ENSSSCG00000023548	GSTCD	protein_coding
8	115970001	116020000	0.377167	8	115902991	116031863	128873	-	ENSSSCG00000023548	GSTCD	protein_coding
8	115980001	116030000	0.395188	8	115902991	116031863	128873	-	ENSSSCG00000023548	GSTCD	protein_coding
8	115990001	116040000	0.359606	8	115902991	116031863	128873	-	ENSSSCG00000023548	GSTCD	protein_coding
8	115990001	116040000	0.359606	8	116031929	116060220	28292	+	ENSSSCG00000030617	INTS12	protein_coding
8	12820001	12870000	0.380995	8	12806878	12969296	162419	-	ENSSSCG00000008748	LCORL	protein_coding
8	12830001	12880000	0.480148	8	12806878	12969296	162419	-	ENSSSCG00000008748	LCORL	protein_coding
8	12840001	12890000	0.503408	8	12806878	12969296	162419	-	ENSSSCG00000008748	LCORL	protein_coding
8	12850001	12900000	0.517652	8	12806878	12969296	162419	-	ENSSSCG00000008748	LCORL	protein_coding
8	12860001	12910000	0.521489	8	12806878	12969296	162419	-	ENSSSCG00000008748	LCORL	protein_coding
8	12870001	12920000	0.503327	8	12806878	12969296	162419	-	ENSSSCG00000008748	LCORL	protein_coding
8	12880001	12930000	0.434216	8	12806878	12969296	162419	-	ENSSSCG00000008748	LCORL	protein_coding
8	12890001	12940000	0.406037	8	12806878	12969296	162419	-	ENSSSCG00000008748	LCORL	protein_coding
8	12900001	12950000	0.437182	8	12806878	12969296	162419	-	ENSSSCG00000008748	LCORL	protein_coding
8	12910001	12960000	0.441923	8	12806878	12969296	162419	-	ENSSSCG00000008748	LCORL	protein_coding
8	12920001	12970000	0.452666	8	12806878	12969296	162419	-	ENSSSCG00000008748	LCORL	protein_coding
8	12930001	12980000	0.506076	8	12806878	12969296	162419	-	ENSSSCG00000008748	LCORL	protein_coding
8	12940001	12990000	0.511421	8	12806878	12969296	162419	-	ENSSSCG00000008748	LCORL	protein_coding
8	12950001	13000000	0.491916	8	12806878	12969296	162419	-	ENSSSCG00000008748	LCORL	protein_coding
8	12960001	13010000	0.502029	8	12806878	12969296	162419	-	ENSSSCG00000008748	LCORL	protein_coding
8	12890001	12940000	0.406037	8	12934250	12937796	3547	-	ENSSSCG00000043042	NA	pseudogene
8	12900001	12950000	0.437182	8	12934250	12937796	3547	-	ENSSSCG00000043042	NA	pseudogene
8	12910001	12960000	0.441923	8	12934250	12937796	3547	-	ENSSSCG00000043042	NA	pseudogene
8	12920001	12970000	0.452666	8	12934250	12937796	3547	-	ENSSSCG00000043042	NA	pseudogene
8	12930001	12980000	0.506076	8	12934250	12937796	3547	-	ENSSSCG00000043042	NA	pseudogene
8	120870001	120920000	0.426612	8	120819142	120879388	60247	-	ENSSSCG00000009179	MTTP	protein_coding
8	120870001	120920000	0.426612	8	120879523	120917433	37911	+	ENSSSCG00000009180	TRMT10A	protein_coding
8	120880001	120930000	0.406813	8	120879523	120917433	37911	+	ENSSSCG00000009180	TRMT10A	protein_coding
8	120890001	120940000	0.400137	8	120879523	120917433	37911	+	ENSSSCG00000009180	TRMT10A	protein_coding

8	120900001	120950000	0.403776	8	120879523	120917433	37911	+	ENSSSCG00000009180	TRMT10A	protein_coding
8	120910001	120960000	0.410246	8	120879523	120917433	37911	+	ENSSSCG00000009180	TRMT10A	protein_coding
8	120870001	120920000	0.426612	8	120918176	121266652	348477	-	ENSSSCG00000009181	C4orf17	protein_coding
8	120880001	120930000	0.406813	8	120918176	121266652	348477	-	ENSSSCG00000009181	C4orf17	protein_coding
8	120890001	120940000	0.400137	8	120918176	121266652	348477	-	ENSSSCG00000009181	C4orf17	protein_coding
8	120900001	120950000	0.403776	8	120918176	121266652	348477	-	ENSSSCG00000009181	C4orf17	protein_coding
8	120910001	120960000	0.410246	8	120918176	121266652	348477	-	ENSSSCG00000009181	C4orf17	protein_coding
8	120920001	120970000	0.366105	8	120918176	121266652	348477	-	ENSSSCG00000009181	C4orf17	protein_coding
8	120930001	120980000	0.367409	8	120918176	121266652	348477	-	ENSSSCG00000009181	C4orf17	protein_coding
8	121070001	121120000	0.358274	8	120918176	121266652	348477	-	ENSSSCG00000009181	C4orf17	protein_coding
8	120930001	120980000	0.367409	8	120970077	120973895	3819	+	ENSSSCG00000051012	NA	protein_coding
8	121070001	121120000	0.358274	8	121020910	121108190	87281	+	ENSSSCG00000009182	NA	protein_coding
8	131420001	131470000	0.360533	8	131285239	131472785	187547	-	ENSSSCG00000009220	DMP1	protein_coding
8	131430001	131480000	0.367283	8	131285239	131472785	187547	-	ENSSSCG00000009220	DMP1	protein_coding
8	131440001	131490000	0.361082	8	131285239	131472785	187547	-	ENSSSCG00000009220	DMP1	protein_coding
8	131420001	131470000	0.360533	8	131319983	131472761	152779	-	ENSSSCG00000009221	DSPP	protein_coding
8	131430001	131480000	0.367283	8	131319983	131472761	152779	-	ENSSSCG00000009221	DSPP	protein_coding
8	131440001	131490000	0.361082	8	131319983	131472761	152779	-	ENSSSCG00000009221	DSPP	protein_coding
8	131420001	131470000	0.360533	8	131387413	131431421	44009	+	ENSSSCG00000009222	SPARCL1	protein_coding
8	131430001	131480000	0.367283	8	131387413	131431421	44009	+	ENSSSCG00000009222	SPARCL1	protein_coding
8	131430001	131480000	0.367283	8	131472576	131569993	97418	+	ENSSSCG00000009225	HSD17B11	protein_coding
8	131440001	131490000	0.361082	8	131472576	131569993	97418	+	ENSSSCG00000009225	HSD17B11	protein_coding
8	37720001	37770000	0.366036	8	37530815	37809761	278947	-	ENSSSCG00000008813	CORIN	protein_coding
8	37730001	37780000	0.362073	8	37530815	37809761	278947	-	ENSSSCG00000008813	CORIN	protein_coding
8	37740001	37790000	0.367548	8	37530815	37809761	278947	-	ENSSSCG00000008813	CORIN	protein_coding
8	37750001	37800000	0.35856	8	37530815	37809761	278947	-	ENSSSCG00000008813	CORIN	protein_coding
8	37750001	37800000	0.35856	8	37797875	37875540	77666	-	ENSSSCG00000008816	NFXL1	protein_coding
8	37840001	37890000	0.39427	8	37797875	37875540	77666	-	ENSSSCG00000008816	NFXL1	protein_coding
8	37850001	37900000	0.433745	8	37797875	37875540	77666	-	ENSSSCG00000008816	NFXL1	protein_coding
8	37860001	37910000	0.409812	8	37797875	37875540	77666	-	ENSSSCG00000008816	NFXL1	protein_coding
8	37870001	37920000	0.391352	8	37797875	37875540	77666	-	ENSSSCG00000008816	NFXL1	protein_coding
8	37850001	37900000	0.433745	8	37896388	37950408	54021	-	ENSSSCG00000038160	CNGA1	protein_coding
8	37860001	37910000	0.409812	8	37896388	37950408	54021	-	ENSSSCG00000038160	CNGA1	protein_coding
8	37870001	37920000	0.391352	8	37896388	37950408	54021	-	ENSSSCG00000038160	CNGA1	protein_coding
8	37880001	37930000	0.378511	8	37896388	37950408	54021	-	ENSSSCG00000038160	CNGA1	protein_coding
8	39760001	39810000	0.364617	8	39715534	40145887	430354	-	ENSSSCG00000008836	SCFD2	protein_coding
8	39770001	39820000	0.369889	8	39715534	40145887	430354	-	ENSSSCG00000008836	SCFD2	protein_coding
8	39780001	39830000	0.393863	8	39715534	40145887	430354	-	ENSSSCG00000008836	SCFD2	protein_coding
8	39990001	40040000	0.35991	8	39715534	40145887	430354	-	ENSSSCG00000008836	SCFD2	protein_coding
8	40000001	40050000	0.353235	8	39715534	40145887	430354	-	ENSSSCG00000008836	SCFD2	protein_coding
8	40010001	40060000	0.370378	8	39715534	40145887	430354	-	ENSSSCG00000008836	SCFD2	protein_coding
8	40020001	40070000	0.374136	8	39715534	40145887	430354	-	ENSSSCG00000008836	SCFD2	protein_coding
8	41460001	41510000	0.365474	8	41402043	41493735	91693	+	ENSSSCG00000008842	KIT	protein_coding
8	41470001	41520000	0.37975	8	41402043	41493735	91693	+	ENSSSCG00000008842	KIT	protein_coding
8	41480001	41530000	0.385584	8	41402043	41493735	91693	+	ENSSSCG00000008842	KIT	protein_coding

8	41490001	41540000	0.380782	8	41402043	41493735	91693	+	ENSSSCG00000008842	KIT	protein_coding
8	41840001	41890000	0.355909	8	41809116	41856339	47224	-	ENSSSCG00000008844	KDR	protein_coding
8	41850001	41900000	0.359875	8	41809116	41856339	47224	-	ENSSSCG00000008844	KDR	protein_coding
8	42880001	42930000	0.392713	8	42856469	43092692	236224	-	ENSSSCG00000008853	TLL1	protein_coding
8	42890001	42940000	0.408947	8	42856469	43092692	236224	-	ENSSSCG00000008853	TLL1	protein_coding
8	42900001	42950000	0.387249	8	42856469	43092692	236224	-	ENSSSCG00000008853	TLL1	protein_coding
8	42910001	42960000	0.360994	8	42856469	43092692	236224	-	ENSSSCG00000008853	TLL1	protein_coding
8	43510001	43560000	0.384139	8	43503667	43695628	191962	-	ENSSSCG00000008854	CPE	protein_coding
8	43520001	43570000	0.360322	8	43503667	43695628	191962	-	ENSSSCG00000008854	CPE	protein_coding
8	44720001	44770000	0.377152	8	44708746	44740980	32235	+	ENSSSCG00000008868	TDO2	protein_coding
8	44730001	44780000	0.438593	8	44708746	44740980	32235	+	ENSSSCG00000008868	TDO2	protein_coding
8	44740001	44790000	0.469978	8	44708746	44740980	32235	+	ENSSSCG00000008868	TDO2	protein_coding
8	44720001	44770000	0.377152	8	44728792	44765041	36250	-	ENSSSCG00000008867	CTSO	protein_coding
8	44730001	44780000	0.438593	8	44728792	44765041	36250	-	ENSSSCG00000008867	CTSO	protein_coding
8	44740001	44790000	0.469978	8	44728792	44765041	36250	-	ENSSSCG00000008867	CTSO	protein_coding
8	44750001	44800000	0.452042	8	44728792	44765041	36250	-	ENSSSCG00000008867	CTSO	protein_coding
8	44760001	44810000	0.378501	8	44728792	44765041	36250	-	ENSSSCG00000008867	CTSO	protein_coding
8	47530001	47580000	0.395419	8	47473787	47601359	127573	+	ENSSSCG00000008875	RXFP1	protein_coding
8	47540001	47590000	0.395774	8	47473787	47601359	127573	+	ENSSSCG00000008875	RXFP1	protein_coding
8	47550001	47600000	0.398947	8	47473787	47601359	127573	+	ENSSSCG00000008875	RXFP1	protein_coding
8	47560001	47610000	0.383712	8	47473787	47601359	127573	+	ENSSSCG00000008875	RXFP1	protein_coding
8	47560001	47610000	0.383712	8	47604751	47608601	3851	-	ENSSSCG00000028492	C4orf46	protein_coding
8	47560001	47610000	0.383712	8	47608689	47657436	48748	+	ENSSSCG00000008877	ETFDH	protein_coding
8	47720001	47770000	0.39051	8	47702578	47838521	135944	+	ENSSSCG00000031175	FNIP2	protein_coding
8	47730001	47780000	0.400806	8	47702578	47838521	135944	+	ENSSSCG00000031175	FNIP2	protein_coding
8	47740001	47790000	0.372162	8	47702578	47838521	135944	+	ENSSSCG00000031175	FNIP2	protein_coding
8	47750001	47800000	0.362329	8	47702578	47838521	135944	+	ENSSSCG00000031175	FNIP2	protein_coding
8	50220001	50270000	0.364127	8	50264272	51052864	788593	-	ENSSSCG00000027183	FSTL5	protein_coding
8	50230001	50280000	0.395591	8	50264272	51052864	788593	-	ENSSSCG00000027183	FSTL5	protein_coding
8	50240001	50290000	0.416764	8	50264272	51052864	788593	-	ENSSSCG00000027183	FSTL5	protein_coding
8	50250001	50300000	0.428298	8	50264272	51052864	788593	-	ENSSSCG00000027183	FSTL5	protein_coding
8	50260001	50310000	0.421981	8	50264272	51052864	788593	-	ENSSSCG00000027183	FSTL5	protein_coding
8	50270001	50320000	0.416514	8	50264272	51052864	788593	-	ENSSSCG00000027183	FSTL5	protein_coding
8	50280001	50330000	0.391765	8	50264272	51052864	788593	-	ENSSSCG00000027183	FSTL5	protein_coding
8	50290001	50340000	0.360273	8	50264272	51052864	788593	-	ENSSSCG00000027183	FSTL5	protein_coding
8	50490001	50540000	0.416233	8	50264272	51052864	788593	-	ENSSSCG00000027183	FSTL5	protein_coding
8	50500001	50550000	0.424103	8	50264272	51052864	788593	-	ENSSSCG00000027183	FSTL5	protein_coding
8	50510001	50560000	0.422267	8	50264272	51052864	788593	-	ENSSSCG00000027183	FSTL5	protein_coding
8	50520001	50570000	0.384311	8	50264272	51052864	788593	-	ENSSSCG00000027183	FSTL5	protein_coding
8	50530001	50580000	0.393809	8	50264272	51052864	788593	-	ENSSSCG00000027183	FSTL5	protein_coding
8	50540001	50590000	0.391579	8	50264272	51052864	788593	-	ENSSSCG00000027183	FSTL5	protein_coding
8	50550001	50600000	0.3579	8	50264272	51052864	788593	-	ENSSSCG00000027183	FSTL5	protein_coding
8	50870001	50920000	0.373064	8	50264272	51052864	788593	-	ENSSSCG00000027183	FSTL5	protein_coding
8	50880001	50930000	0.379016	8	50264272	51052864	788593	-	ENSSSCG00000027183	FSTL5	protein_coding
8	50890001	50940000	0.365615	8	50264272	51052864	788593	-	ENSSSCG00000027183	FSTL5	protein_coding

8	50900001	50950000	0.361271	8	50264272	51052864	788593	-	ENSSSCG00000027183	FSTL5	protein_coding
8	50980001	51030000	0.419224	8	50264272	51052864	788593	-	ENSSSCG00000027183	FSTL5	protein_coding
8	50990001	51040000	0.461885	8	50264272	51052864	788593	-	ENSSSCG00000027183	FSTL5	protein_coding
8	51000001	51050000	0.487379	8	50264272	51052864	788593	-	ENSSSCG00000027183	FSTL5	protein_coding
8	51010001	51060000	0.472206	8	50264272	51052864	788593	-	ENSSSCG00000027183	FSTL5	protein_coding
8	51020001	51070000	0.474586	8	50264272	51052864	788593	-	ENSSSCG00000027183	FSTL5	protein_coding
8	51030001	51080000	0.432651	8	50264272	51052864	788593	-	ENSSSCG00000027183	FSTL5	protein_coding
8	51040001	51090000	0.376653	8	50264272	51052864	788593	-	ENSSSCG00000027183	FSTL5	protein_coding
8	51530001	51580000	0.413913	8	51574978	51576852	1875	+	ENSSSCG00000045819	NA	protein_coding
8	51540001	51590000	0.452776	8	51574978	51576852	1875	+	ENSSSCG00000045819	NA	protein_coding
8	51550001	51600000	0.450993	8	51574978	51576852	1875	+	ENSSSCG00000045819	NA	protein_coding
8	51560001	51610000	0.42912	8	51574978	51576852	1875	+	ENSSSCG00000045819	NA	protein_coding
8	51570001	51620000	0.417073	8	51574978	51576852	1875	+	ENSSSCG00000045819	NA	protein_coding
8	52310001	52360000	0.468372	8	52326545	52329300	2756	-	ENSSSCG00000026079	TKTL2	protein_coding
8	52320001	52370000	0.485628	8	52326545	52329300	2756	-	ENSSSCG00000026079	TKTL2	protein_coding
8	52310001	52360000	0.468372	8	52343233	52375044	31812	+	ENSSSCG00000008890	TMA16	protein_coding
8	52320001	52370000	0.485628	8	52343233	52375044	31812	+	ENSSSCG00000008890	TMA16	protein_coding
8	52330001	52380000	0.574129	8	52343233	52375044	31812	+	ENSSSCG00000008890	TMA16	protein_coding
8	52340001	52390000	0.597283	8	52343233	52375044	31812	+	ENSSSCG00000008890	TMA16	protein_coding
8	52350001	52400000	0.542864	8	52343233	52375044	31812	+	ENSSSCG00000008890	TMA16	protein_coding
8	52360001	52410000	0.5641	8	52343233	52375044	31812	+	ENSSSCG00000008890	TMA16	protein_coding
8	52370001	52420000	0.551206	8	52343233	52375044	31812	+	ENSSSCG00000008890	TMA16	protein_coding
8	52330001	52380000	0.574129	8	52375510	52951031	575522	-	ENSSSCG00000039175	MARCHF1	protein_coding
8	52340001	52390000	0.597283	8	52375510	52951031	575522	-	ENSSSCG00000039175	MARCHF1	protein_coding
8	52350001	52400000	0.542864	8	52375510	52951031	575522	-	ENSSSCG00000039175	MARCHF1	protein_coding
8	52360001	52410000	0.5641	8	52375510	52951031	575522	-	ENSSSCG00000039175	MARCHF1	protein_coding
8	52370001	52420000	0.551206	8	52375510	52951031	575522	-	ENSSSCG00000039175	MARCHF1	protein_coding
8	52380001	52430000	0.488727	8	52375510	52951031	575522	-	ENSSSCG00000039175	MARCHF1	protein_coding
8	52390001	52440000	0.473081	8	52375510	52951031	575522	-	ENSSSCG00000039175	MARCHF1	protein_coding
8	52400001	52450000	0.488453	8	52375510	52951031	575522	-	ENSSSCG00000039175	MARCHF1	protein_coding
8	52410001	52460000	0.453897	8	52375510	52951031	575522	-	ENSSSCG00000039175	MARCHF1	protein_coding
8	52420001	52470000	0.425045	8	52375510	52951031	575522	-	ENSSSCG00000039175	MARCHF1	protein_coding
8	52430001	52480000	0.456456	8	52375510	52951031	575522	-	ENSSSCG00000039175	MARCHF1	protein_coding
8	52440001	52490000	0.47715	8	52375510	52951031	575522	-	ENSSSCG00000039175	MARCHF1	protein_coding
8	52450001	52500000	0.473156	8	52375510	52951031	575522	-	ENSSSCG00000039175	MARCHF1	protein_coding
8	52460001	52510000	0.482833	8	52375510	52951031	575522	-	ENSSSCG00000039175	MARCHF1	protein_coding
8	52470001	52520000	0.542221	8	52375510	52951031	575522	-	ENSSSCG00000039175	MARCHF1	protein_coding
8	52480001	52530000	0.541287	8	52375510	52951031	575522	-	ENSSSCG00000039175	MARCHF1	protein_coding
8	52490001	52540000	0.543094	8	52375510	52951031	575522	-	ENSSSCG00000039175	MARCHF1	protein_coding
8	52500001	52550000	0.559342	8	52375510	52951031	575522	-	ENSSSCG00000039175	MARCHF1	protein_coding
8	52510001	52560000	0.556224	8	52375510	52951031	575522	-	ENSSSCG00000039175	MARCHF1	protein_coding
8	52520001	52570000	0.477435	8	52375510	52951031	575522	-	ENSSSCG00000039175	MARCHF1	protein_coding
8	52530001	52580000	0.431045	8	52375510	52951031	575522	-	ENSSSCG00000039175	MARCHF1	protein_coding
8	52540001	52590000	0.419421	8	52375510	52951031	575522	-	ENSSSCG00000039175	MARCHF1	protein_coding
8	52550001	52600000	0.389682	8	52375510	52951031	575522	-	ENSSSCG00000039175	MARCHF1	protein_coding

8	52690001	52740000	0.354706	8	52375510	52951031	575522	-	ENSSSCG00000039175	MARCHF1	protein_coding
8	52700001	52750000	0.362553	8	52375510	52951031	575522	-	ENSSSCG00000039175	MARCHF1	protein_coding
8	52710001	52760000	0.38882	8	52375510	52951031	575522	-	ENSSSCG00000039175	MARCHF1	protein_coding
8	52490001	52540000	0.543094	8	52536298	52539588	3291	+	ENSSSCG00000042503	NA	pseudogene
8	52500001	52550000	0.559342	8	52536298	52539588	3291	+	ENSSSCG00000042503	NA	pseudogene
8	52510001	52560000	0.556224	8	52536298	52539588	3291	+	ENSSSCG00000042503	NA	pseudogene
8	52520001	52570000	0.477435	8	52536298	52539588	3291	+	ENSSSCG00000042503	NA	pseudogene
8	52530001	52580000	0.431045	8	52536298	52539588	3291	+	ENSSSCG00000042503	NA	pseudogene
8	55480001	55530000	0.356675	8	55463441	55600939	137499	+	ENSSSCG00000008903	CRACD	protein_coding
8	55820001	55870000	0.392897	8	55787215	55829798	42584	+	ENSSSCG00000032353	NA	protein_coding
8	55820001	55870000	0.392897	8	55862191	55901633	39443	-	ENSSSCG00000008898	NA	protein_coding
8	55830001	55880000	0.403956	8	55862191	55901633	39443	-	ENSSSCG00000008898	NA	protein_coding
8	55840001	55890000	0.369372	8	55862191	55901633	39443	-	ENSSSCG00000008898	NA	protein_coding
8	55850001	55900000	0.361607	8	55862191	55901633	39443	-	ENSSSCG00000008898	NA	protein_coding
8	56300001	56350000	0.383068	8	56279894	56357835	77942	-	ENSSSCG00000008913	IGFBP7	protein_coding
8	56310001	56360000	0.388205	8	56279894	56357835	77942	-	ENSSSCG00000008913	IGFBP7	protein_coding
8	56320001	56370000	0.433216	8	56279894	56357835	77942	-	ENSSSCG00000008913	IGFBP7	protein_coding
8	56330001	56380000	0.434064	8	56279894	56357835	77942	-	ENSSSCG00000008913	IGFBP7	protein_coding
8	56340001	56390000	0.404474	8	56279894	56357835	77942	-	ENSSSCG00000008913	IGFBP7	protein_coding
8	56350001	56400000	0.392863	8	56279894	56357835	77942	-	ENSSSCG00000008913	IGFBP7	protein_coding
8	63360001	63410000	0.35511	8	63285489	63604734	319246	-	ENSSSCG00000008919	EPHA5	protein_coding
8	63380001	63430000	0.364639	8	63285489	63604734	319246	-	ENSSSCG00000008919	EPHA5	protein_coding
8	63390001	63440000	0.355764	8	63285489	63604734	319246	-	ENSSSCG00000008919	EPHA5	protein_coding
8	65230001	65280000	0.406013	8	65238088	65315714	77627	-	ENSSSCG00000008921	CENPC	protein_coding
8	65240001	65290000	0.410091	8	65238088	65315714	77627	-	ENSSSCG00000008921	CENPC	protein_coding
8	65250001	65300000	0.408804	8	65238088	65315714	77627	-	ENSSSCG00000008921	CENPC	protein_coding
8	65260001	65310000	0.394931	8	65238088	65315714	77627	-	ENSSSCG00000008921	CENPC	protein_coding
8	65270001	65320000	0.401015	8	65238088	65315714	77627	-	ENSSSCG00000008921	CENPC	protein_coding
8	65280001	65330000	0.39995	8	65238088	65315714	77627	-	ENSSSCG00000008921	CENPC	protein_coding
8	65290001	65340000	0.405688	8	65238088	65315714	77627	-	ENSSSCG00000008921	CENPC	protein_coding
8	65300001	65350000	0.398148	8	65238088	65315714	77627	-	ENSSSCG00000008921	CENPC	protein_coding
8	65310001	65360000	0.389584	8	65238088	65315714	77627	-	ENSSSCG00000008921	CENPC	protein_coding
8	65290001	65340000	0.405688	8	65332522	65392965	60444	+	ENSSSCG00000008923	STAP1	protein_coding
8	65300001	65350000	0.398148	8	65332522	65392965	60444	+	ENSSSCG00000008923	STAP1	protein_coding
8	65310001	65360000	0.389584	8	65332522	65392965	60444	+	ENSSSCG00000008923	STAP1	protein_coding
8	65320001	65370000	0.38688	8	65332522	65392965	60444	+	ENSSSCG00000008923	STAP1	protein_coding
8	65360001	65410000	0.358567	8	65332522	65392965	60444	+	ENSSSCG00000008923	STAP1	protein_coding
8	65370001	65420000	0.368861	8	65332522	65392965	60444	+	ENSSSCG00000008923	STAP1	protein_coding
8	65380001	65430000	0.372746	8	65332522	65392965	60444	+	ENSSSCG00000008923	STAP1	protein_coding
8	65390001	65440000	0.369233	8	65332522	65392965	60444	+	ENSSSCG00000008923	STAP1	protein_coding
8	65360001	65410000	0.358567	8	65381053	65488925	107873	-	ENSSSCG00000008924	UBA6	protein_coding
8	65370001	65420000	0.368861	8	65381053	65488925	107873	-	ENSSSCG00000008924	UBA6	protein_coding
8	65380001	65430000	0.372746	8	65381053	65488925	107873	-	ENSSSCG00000008924	UBA6	protein_coding
8	65390001	65440000	0.369233	8	65381053	65488925	107873	-	ENSSSCG00000008924	UBA6	protein_coding
8	65400001	65450000	0.387056	8	65381053	65488925	107873	-	ENSSSCG00000008924	UBA6	protein_coding

8	65410001	65460000	0.373775	8	65381053	65488925	107873	-	ENSSSCG00000008924	UBA6	protein_coding
8	65420001	65470000	0.378998	8	65381053	65488925	107873	-	ENSSSCG00000008924	UBA6	protein_coding
8	65430001	65480000	0.399998	8	65381053	65488925	107873	-	ENSSSCG00000008924	UBA6	protein_coding
8	65440001	65490000	0.415007	8	65381053	65488925	107873	-	ENSSSCG00000008924	UBA6	protein_coding
8	65450001	65500000	0.394417	8	65381053	65488925	107873	-	ENSSSCG00000008924	UBA6	protein_coding
8	65460001	65510000	0.40705	8	65381053	65488925	107873	-	ENSSSCG00000008924	UBA6	protein_coding
8	65450001	65500000	0.394417	8	65493138	65561989	68852	-	ENSSSCG00000029658	NA	protein_coding
8	65460001	65510000	0.40705	8	65493138	65561989	68852	-	ENSSSCG00000029658	NA	protein_coding
8	65840001	65890000	0.391212	8	65859658	65875032	15375	-	ENSSSCG00000038616	NA	protein_coding
8	65850001	65900000	0.402083	8	65859658	65875032	15375	-	ENSSSCG00000038616	NA	protein_coding
8	65860001	65910000	0.37533	8	65859658	65875032	15375	-	ENSSSCG00000038616	NA	protein_coding
8	65840001	65890000	0.391212	8	65882623	65902437	19815	-	ENSSSCG00000008932	TMPRSS11B	protein_coding
8	65850001	65900000	0.402083	8	65882623	65902437	19815	-	ENSSSCG00000008932	TMPRSS11B	protein_coding
8	65860001	65910000	0.37533	8	65882623	65902437	19815	-	ENSSSCG00000008932	TMPRSS11B	protein_coding
8	67010001	67060000	0.353579	8	67058472	67069291	10820	+	ENSSSCG00000009266	ODAM	protein_coding
8	67310001	67360000	0.362255	8	67312972	67326700	13729	+	ENSSSCG00000008936	AMTN	protein_coding
8	67310001	67360000	0.362255	8	67331204	67334935	3732	+	ENSSSCG00000047617	NA	protein_coding
8	67690001	67740000	0.375073	8	67648042	67702227	54186	+	ENSSSCG00000008942	MOB1B	protein_coding
8	67700001	67750000	0.368671	8	67648042	67702227	54186	+	ENSSSCG00000008942	MOB1B	protein_coding
8	67690001	67740000	0.375073	8	67706880	68290496	583617	+	ENSSSCG00000008943	SLC4A4	protein_coding
8	67700001	67750000	0.368671	8	67706880	68290496	583617	+	ENSSSCG00000008943	SLC4A4	protein_coding
8	67710001	67760000	0.40673	8	67706880	68290496	583617	+	ENSSSCG00000008943	SLC4A4	protein_coding
8	67720001	67770000	0.397679	8	67706880	68290496	583617	+	ENSSSCG00000008943	SLC4A4	protein_coding
8	67730001	67780000	0.365452	8	67706880	68290496	583617	+	ENSSSCG00000008943	SLC4A4	protein_coding
8	68200001	68250000	0.3709	8	67706880	68290496	583617	+	ENSSSCG00000008943	SLC4A4	protein_coding
8	68210001	68260000	0.363539	8	67706880	68290496	583617	+	ENSSSCG00000008943	SLC4A4	protein_coding
8	68240001	68290000	0.356085	8	67706880	68290496	583617	+	ENSSSCG00000008943	SLC4A4	protein_coding
8	68250001	68300000	0.37176	8	67706880	68290496	583617	+	ENSSSCG00000008943	SLC4A4	protein_coding
15	62830001	62880000	0.382668	15	62749399	62908220	158822	+	ENSSSCG00000048898	NA	lncRNA
15	62840001	62890000	0.377053	15	62749399	62908220	158822	+	ENSSSCG00000048898	NA	lncRNA
15	62850001	62900000	0.36048	15	62749399	62908220	158822	+	ENSSSCG00000048898	NA	lncRNA
15	64100001	64150000	0.385023	15	64147332	64155988	8657	+	ENSSSCG00000051479	NA	lncRNA
15	64110001	64160000	0.382946	15	64147332	64155988	8657	+	ENSSSCG00000051479	NA	lncRNA
15	64120001	64170000	0.396764	15	64147332	64155988	8657	+	ENSSSCG00000051479	NA	lncRNA
15	64130001	64180000	0.412359	15	64147332	64155988	8657	+	ENSSSCG00000051479	NA	lncRNA
15	64140001	64190000	0.395798	15	64147332	64155988	8657	+	ENSSSCG00000051479	NA	lncRNA
15	64920001	64970000	0.401557	15	64923594	64928106	4513	-	ENSSSCG00000043178	NA	lncRNA
15	64920001	64970000	0.401557	15	64934371	64943139	8769	-	ENSSSCG00000044287	NA	lncRNA
15	64930001	64980000	0.464372	15	64934371	64943139	8769	-	ENSSSCG00000044287	NA	lncRNA
15	64940001	64990000	0.465798	15	64934371	64943139	8769	-	ENSSSCG00000044287	NA	lncRNA
15	67920001	67970000	0.363608	15	67959645	67964551	4907	-	ENSSSCG00000046382	NA	lncRNA
15	105230001	105280000	0.395457	15	105278920	105279021	102	+	ENSSSCG00000026451	U6	snRNA
15	105240001	105290000	0.437171	15	105278920	105279021	102	+	ENSSSCG00000026451	U6	snRNA
15	105250001	105300000	0.466652	15	105278920	105279021	102	+	ENSSSCG00000026451	U6	snRNA
15	105260001	105310000	0.577348	15	105278920	105279021	102	+	ENSSSCG00000026451	U6	snRNA

15	105270001	105320000	0.583202	15	105278920	105279021	102	+	ENSSSCG00000026451	U6	snRNA
15	105310001	105360000	0.543506	15	105353662	105353751	90	+	ENSSSCG00000022334	U6	snRNA
15	105320001	105370000	0.542239	15	105353662	105353751	90	+	ENSSSCG00000022334	U6	snRNA
15	105330001	105380000	0.577213	15	105353662	105353751	90	+	ENSSSCG00000022334	U6	snRNA
15	105340001	105390000	0.488921	15	105353662	105353751	90	+	ENSSSCG00000022334	U6	snRNA
15	105350001	105400000	0.485906	15	105353662	105353751	90	+	ENSSSCG00000022334	U6	snRNA
15	73850001	73900000	0.354682	15	73772251	73973610	201360	+	ENSSSCG00000043455	NA	lncRNA
15	73860001	73910000	0.373604	15	73772251	73973610	201360	+	ENSSSCG00000043455	NA	lncRNA
15	73870001	73920000	0.432985	15	73772251	73973610	201360	+	ENSSSCG00000043455	NA	lncRNA
15	73880001	73930000	0.442803	15	73772251	73973610	201360	+	ENSSSCG00000043455	NA	lncRNA
15	73890001	73940000	0.416415	15	73772251	73973610	201360	+	ENSSSCG00000043455	NA	lncRNA
15	73970001	74020000	0.388352	15	73772251	73973610	201360	+	ENSSSCG00000043455	NA	lncRNA
15	73850001	73900000	0.354682	15	73889813	73894913	5101	+	ENSSSCG00000048454	NA	lncRNA
15	73860001	73910000	0.373604	15	73889813	73894913	5101	+	ENSSSCG00000048454	NA	lncRNA
15	73870001	73920000	0.432985	15	73889813	73894913	5101	+	ENSSSCG00000048454	NA	lncRNA
15	73880001	73930000	0.442803	15	73889813	73894913	5101	+	ENSSSCG00000048454	NA	lncRNA
15	73890001	73940000	0.416415	15	73889813	73894913	5101	+	ENSSSCG00000048454	NA	lncRNA
15	74010001	74060000	0.371726	15	74057540	74064320	6781	+	ENSSSCG00000048891	NA	lncRNA
15	63640001	63690000	0.353058	15	63680291	63680397	107	-	ENSSSCG00000035191	U6	snRNA
15	77560001	77610000	0.385504	15	77474816	77564456	89641	-	ENSSSCG00000015945	METTL8	protein_coding
15	134830001	134880000	0.358751	15	134813525	134856185	42661	-	ENSSSCG00000043599	NA	lncRNA
15	134840001	134890000	0.383103	15	134813525	134856185	42661	-	ENSSSCG00000043599	NA	lncRNA
15	77560001	77610000	0.385504	15	77564692	77603620	38929	+	ENSSSCG00000015947	DCAF17	protein_coding
15	77570001	77620000	0.40401	15	77564692	77603620	38929	+	ENSSSCG00000015947	DCAF17	protein_coding
15	77580001	77630000	0.396669	15	77564692	77603620	38929	+	ENSSSCG00000015947	DCAF17	protein_coding
15	77590001	77640000	0.396177	15	77564692	77603620	38929	+	ENSSSCG00000015947	DCAF17	protein_coding
15	77600001	77650000	0.38764	15	77564692	77603620	38929	+	ENSSSCG00000015947	DCAF17	protein_coding
15	64930001	64980000	0.464372	15	64971653	64971759	107	-	ENSSSCG00000036729	U6	snRNA
15	64940001	64990000	0.465798	15	64971653	64971759	107	-	ENSSSCG00000036729	U6	snRNA
15	64950001	65000000	0.476182	15	64971653	64971759	107	-	ENSSSCG00000036729	U6	snRNA
15	64960001	65010000	0.483831	15	64971653	64971759	107	-	ENSSSCG00000036729	U6	snRNA
15	64970001	65020000	0.488557	15	64971653	64971759	107	-	ENSSSCG00000036729	U6	snRNA
15	77600001	77650000	0.38764	15	77649040	77695138	46099	+	ENSSSCG00000025049	CYBRD1	protein_coding
15	67920001	67970000	0.363608	15	67952210	68048963	96754	+	ENSSSCG00000015889	NA	lncRNA
15	64590001	64640000	0.442188	15	64630710	64637033	6324	+	ENSSSCG00000047320	NA	protein_coding
15	64600001	64650000	0.428726	15	64630710	64637033	6324	+	ENSSSCG00000047320	NA	protein_coding
15	64610001	64660000	0.425384	15	64630710	64637033	6324	+	ENSSSCG00000047320	NA	protein_coding
15	64620001	64670000	0.408089	15	64630710	64637033	6324	+	ENSSSCG00000047320	NA	protein_coding
15	64630001	64680000	0.416199	15	64630710	64637033	6324	+	ENSSSCG00000047320	NA	protein_coding
15	93830001	93880000	0.354898	15	93611856	93981110	369255	-	ENSSSCG00000016035	COL5A2	protein_coding
15	94010001	94060000	0.354049	15	93981215	94026707	45493	+	ENSSSCG00000016036	WDR75	protein_coding
15	94220001	94270000	0.412898	15	94261905	94272121	10217	+	ENSSSCG00000016041	NA	protein_coding
15	94230001	94280000	0.414311	15	94261905	94272121	10217	+	ENSSSCG00000016041	NA	protein_coding
15	94240001	94290000	0.393795	15	94261905	94272121	10217	+	ENSSSCG00000016041	NA	protein_coding
15	94220001	94270000	0.412898	15	94261905	94272129	10225	+	ENSSSCG00000041398	ASDURF	protein_coding

15	94230001	94280000	0.414311	15	94261905	94272129	10225	+	ENSSSCG00000041398	ASDURF	protein_coding
15	94240001	94290000	0.393795	15	94261905	94272129	10225	+	ENSSSCG00000041398	ASDURF	protein_coding
15	94230001	94280000	0.414311	15	94274526	94340027	65502	+	ENSSSCG00000016042	ANKAR	protein_coding
15	94240001	94290000	0.393795	15	94274526	94340027	65502	+	ENSSSCG00000016042	ANKAR	protein_coding
15	69340001	69390000	0.352739	15	69365997	69368570	2574	+	ENSSSCG00000044821	NA	protein_coding
15	94370001	94420000	0.374087	15	94351324	94601889	250566	+	ENSSSCG00000016045	PMS1	protein_coding
15	94380001	94430000	0.402312	15	94351324	94601889	250566	+	ENSSSCG00000016045	PMS1	protein_coding
15	94390001	94440000	0.4066	15	94351324	94601889	250566	+	ENSSSCG00000016045	PMS1	protein_coding
15	94400001	94450000	0.404361	15	94351324	94601889	250566	+	ENSSSCG00000016045	PMS1	protein_coding
15	94410001	94460000	0.404525	15	94351324	94601889	250566	+	ENSSSCG00000016045	PMS1	protein_coding
15	94420001	94470000	0.402013	15	94351324	94601889	250566	+	ENSSSCG00000016045	PMS1	protein_coding
15	94430001	94480000	0.386448	15	94351324	94601889	250566	+	ENSSSCG00000016045	PMS1	protein_coding
15	94440001	94490000	0.391505	15	94351324	94601889	250566	+	ENSSSCG00000016045	PMS1	protein_coding
15	94450001	94500000	0.409959	15	94351324	94601889	250566	+	ENSSSCG00000016045	PMS1	protein_coding
15	94460001	94510000	0.415223	15	94351324	94601889	250566	+	ENSSSCG00000016045	PMS1	protein_coding
15	94470001	94520000	0.40715	15	94351324	94601889	250566	+	ENSSSCG00000016045	PMS1	protein_coding
15	94480001	94530000	0.402904	15	94351324	94601889	250566	+	ENSSSCG00000016045	PMS1	protein_coding
15	94370001	94420000	0.374087	15	94359333	94377303	17971	-	ENSSSCG00000016044	ORMDL1	protein_coding
15	94430001	94480000	0.386448	15	94478021	94791797	313777	+	ENSSSCG00000033941	C2orf88	protein_coding
15	94440001	94490000	0.391505	15	94478021	94791797	313777	+	ENSSSCG00000033941	C2orf88	protein_coding
15	94450001	94500000	0.409959	15	94478021	94791797	313777	+	ENSSSCG00000033941	C2orf88	protein_coding
15	94460001	94510000	0.415223	15	94478021	94791797	313777	+	ENSSSCG00000033941	C2orf88	protein_coding
15	94470001	94520000	0.40715	15	94478021	94791797	313777	+	ENSSSCG00000033941	C2orf88	protein_coding
15	94480001	94530000	0.402904	15	94478021	94791797	313777	+	ENSSSCG00000033941	C2orf88	protein_coding
15	94610001	94660000	0.356077	15	94478021	94791797	313777	+	ENSSSCG00000033941	C2orf88	protein_coding
15	94620001	94670000	0.357633	15	94478021	94791797	313777	+	ENSSSCG00000033941	C2orf88	protein_coding
15	94610001	94660000	0.356077	15	94600517	94912171	311655	+	ENSSSCG00000041649	NA	protein_coding
15	94620001	94670000	0.357633	15	94600517	94912171	311655	+	ENSSSCG00000041649	NA	protein_coding
15	94610001	94660000	0.356077	15	94620269	94628546	8278	-	ENSSSCG00000016047	MSTN	protein_coding
15	94620001	94670000	0.357633	15	94620269	94628546	8278	-	ENSSSCG00000016047	MSTN	protein_coding
15	74190001	74240000	0.354955	15	74217009	74221983	4975	+	ENSSSCG00000048880	NA	protein_coding
15	104090001	104140000	0.382849	15	104071268	104099188	27921	-	ENSSSCG00000016091	NA	protein_coding
15	104090001	104140000	0.382849	15	104099384	104152527	53144	+	ENSSSCG00000016092	SGO2	protein_coding
15	105190001	105240000	0.367572	15	105105234	105250563	145330	-	ENSSSCG00000016107	C2CD6	protein_coding
15	105230001	105280000	0.395457	15	105105234	105250563	145330	-	ENSSSCG00000016107	C2CD6	protein_coding
15	105240001	105290000	0.437171	15	105105234	105250563	145330	-	ENSSSCG00000016107	C2CD6	protein_coding
15	105250001	105300000	0.466652	15	105105234	105250563	145330	-	ENSSSCG00000016107	C2CD6	protein_coding
15	105230001	105280000	0.395457	15	105252479	105276771	24293	-	ENSSSCG00000035161	TMEM237	protein_coding
15	105240001	105290000	0.437171	15	105252479	105276771	24293	-	ENSSSCG00000035161	TMEM237	protein_coding
15	105250001	105300000	0.466652	15	105252479	105276771	24293	-	ENSSSCG00000035161	TMEM237	protein_coding
15	105260001	105310000	0.577348	15	105252479	105276771	24293	-	ENSSSCG00000035161	TMEM237	protein_coding
15	105270001	105320000	0.583202	15	105252479	105276771	24293	-	ENSSSCG00000035161	TMEM237	protein_coding
15	105230001	105280000	0.395457	15	105276822	105319030	42209	-	ENSSSCG00000026184	MPP4	protein_coding
15	105240001	105290000	0.437171	15	105276822	105319030	42209	-	ENSSSCG00000026184	MPP4	protein_coding
15	105250001	105300000	0.466652	15	105276822	105319030	42209	-	ENSSSCG00000026184	MPP4	protein_coding

15	105260001	105310000	0.577348	15	105276822	105319030	42209	-	ENSSSCG00000026184	MPP4	protein_coding
15	105270001	105320000	0.583202	15	105276822	105319030	42209	-	ENSSSCG00000026184	MPP4	protein_coding
15	105280001	105330000	0.566955	15	105276822	105319030	42209	-	ENSSSCG00000026184	MPP4	protein_coding
15	105290001	105340000	0.585178	15	105276822	105319030	42209	-	ENSSSCG00000026184	MPP4	protein_coding
15	105300001	105350000	0.566851	15	105276822	105319030	42209	-	ENSSSCG00000026184	MPP4	protein_coding
15	105310001	105360000	0.543506	15	105276822	105319030	42209	-	ENSSSCG00000026184	MPP4	protein_coding
15	105280001	105330000	0.566955	15	105328718	105401108	72391	-	ENSSSCG00000016109	ALS2	protein_coding
15	105290001	105340000	0.585178	15	105328718	105401108	72391	-	ENSSSCG00000016109	ALS2	protein_coding
15	105300001	105350000	0.566851	15	105328718	105401108	72391	-	ENSSSCG00000016109	ALS2	protein_coding
15	105310001	105360000	0.543506	15	105328718	105401108	72391	-	ENSSSCG00000016109	ALS2	protein_coding
15	105320001	105370000	0.542239	15	105328718	105401108	72391	-	ENSSSCG00000016109	ALS2	protein_coding
15	105330001	105380000	0.577213	15	105328718	105401108	72391	-	ENSSSCG00000016109	ALS2	protein_coding
15	105340001	105390000	0.488921	15	105328718	105401108	72391	-	ENSSSCG00000016109	ALS2	protein_coding
15	105350001	105400000	0.485906	15	105328718	105401108	72391	-	ENSSSCG00000016109	ALS2	protein_coding
15	105360001	105410000	0.439182	15	105328718	105401108	72391	-	ENSSSCG00000016109	ALS2	protein_coding
15	105370001	105420000	0.380036	15	105328718	105401108	72391	-	ENSSSCG00000016109	ALS2	protein_coding
15	105390001	105440000	0.44402	15	105328718	105401108	72391	-	ENSSSCG00000016109	ALS2	protein_coding
15	105400001	105450000	0.356038	15	105328718	105401108	72391	-	ENSSSCG00000016109	ALS2	protein_coding
15	105360001	105410000	0.439182	15	105401164	105506117	104954	+	ENSSSCG00000022296	CDK15	protein_coding
15	105370001	105420000	0.380036	15	105401164	105506117	104954	+	ENSSSCG00000022296	CDK15	protein_coding
15	105390001	105440000	0.44402	15	105401164	105506117	104954	+	ENSSSCG00000022296	CDK15	protein_coding
15	105400001	105450000	0.356038	15	105401164	105506117	104954	+	ENSSSCG00000022296	CDK15	protein_coding
15	105410001	105460000	0.383312	15	105401164	105506117	104954	+	ENSSSCG00000022296	CDK15	protein_coding
15	105420001	105470000	0.385838	15	105401164	105506117	104954	+	ENSSSCG00000022296	CDK15	protein_coding
15	105430001	105480000	0.366223	15	105401164	105506117	104954	+	ENSSSCG00000022296	CDK15	protein_coding
15	105480001	105530000	0.378069	15	105401164	105506117	104954	+	ENSSSCG00000022296	CDK15	protein_coding
15	105490001	105540000	0.401445	15	105401164	105506117	104954	+	ENSSSCG00000022296	CDK15	protein_coding
15	61430001	61480000	0.355687	15	61440656	62047336	606681	+	ENSSSCG00000022671	GALNT13	protein_coding
15	61510001	61560000	0.37337	15	61440656	62047336	606681	+	ENSSSCG00000022671	GALNT13	protein_coding
15	61520001	61570000	0.380903	15	61440656	62047336	606681	+	ENSSSCG00000022671	GALNT13	protein_coding
15	61530001	61580000	0.383145	15	61440656	62047336	606681	+	ENSSSCG00000022671	GALNT13	protein_coding
15	63610001	63660000	0.373025	15	63592698	63728634	135937	+	ENSSSCG00000015872	GPD2	protein_coding
15	63620001	63670000	0.392687	15	63592698	63728634	135937	+	ENSSSCG00000015872	GPD2	protein_coding
15	63630001	63680000	0.357516	15	63592698	63728634	135937	+	ENSSSCG00000015872	GPD2	protein_coding
15	63640001	63690000	0.353058	15	63592698	63728634	135937	+	ENSSSCG00000015872	GPD2	protein_coding
15	64320001	64370000	0.404622	15	64353472	64382152	28681	-	ENSSSCG00000029753	CYTIP	protein_coding
15	64330001	64380000	0.411925	15	64353472	64382152	28681	-	ENSSSCG00000029753	CYTIP	protein_coding
15	64340001	64390000	0.435048	15	64353472	64382152	28681	-	ENSSSCG00000029753	CYTIP	protein_coding
15	64350001	64400000	0.423577	15	64353472	64382152	28681	-	ENSSSCG00000029753	CYTIP	protein_coding
15	64360001	64410000	0.416912	15	64353472	64382152	28681	-	ENSSSCG00000029753	CYTIP	protein_coding
15	64370001	64420000	0.361999	15	64353472	64382152	28681	-	ENSSSCG00000029753	CYTIP	protein_coding
15	64430001	64480000	0.403997	15	64455989	64529107	73119	-	ENSSSCG00000015873	ACVR1C	protein_coding
15	64520001	64570000	0.363649	15	64455989	64529107	73119	-	ENSSSCG00000015873	ACVR1C	protein_coding
15	64700001	64750000	0.387395	15	64748761	64892153	143393	-	ENSSSCG00000015874	ACVR1	protein_coding
15	64710001	64760000	0.40109	15	64748761	64892153	143393	-	ENSSSCG00000015874	ACVR1	protein_coding

15	64720001	64770000	0.422299	15	64748761	64892153	143393	-	ENSSSCG00000015874	ACVR1	protein_coding
15	64730001	64780000	0.436439	15	64748761	64892153	143393	-	ENSSSCG00000015874	ACVR1	protein_coding
15	64740001	64790000	0.41562	15	64748761	64892153	143393	-	ENSSSCG00000015874	ACVR1	protein_coding
15	64750001	64800000	0.399636	15	64748761	64892153	143393	-	ENSSSCG00000015874	ACVR1	protein_coding
15	64760001	64810000	0.366474	15	64748761	64892153	143393	-	ENSSSCG00000015874	ACVR1	protein_coding
15	64920001	64970000	0.401557	15	64955472	64956059	588	+	ENSSSCG00000032755	NA	protein_coding
15	64930001	64980000	0.464372	15	64955472	64956059	588	+	ENSSSCG00000032755	NA	protein_coding
15	64940001	64990000	0.465798	15	64955472	64956059	588	+	ENSSSCG00000032755	NA	protein_coding
15	64950001	65000000	0.476182	15	64955472	64956059	588	+	ENSSSCG00000032755	NA	protein_coding
15	65020001	65070000	0.462679	15	65062034	65431023	368990	-	ENSSSCG00000015876	CCDC148	protein_coding
15	65030001	65080000	0.415474	15	65062034	65431023	368990	-	ENSSSCG00000015876	CCDC148	protein_coding
15	65040001	65090000	0.359844	15	65062034	65431023	368990	-	ENSSSCG00000015876	CCDC148	protein_coding
15	65270001	65320000	0.360941	15	65062034	65431023	368990	-	ENSSSCG00000015876	CCDC148	protein_coding
15	65330001	65380000	0.359074	15	65062034	65431023	368990	-	ENSSSCG00000015876	CCDC148	protein_coding
15	65340001	65390000	0.361056	15	65062034	65431023	368990	-	ENSSSCG00000015876	CCDC148	protein_coding
15	65350001	65400000	0.359105	15	65062034	65431023	368990	-	ENSSSCG00000015876	CCDC148	protein_coding
15	65360001	65410000	0.384866	15	65062034	65431023	368990	-	ENSSSCG00000015876	CCDC148	protein_coding
15	65370001	65420000	0.37026	15	65062034	65431023	368990	-	ENSSSCG00000015876	CCDC148	protein_coding
15	65380001	65430000	0.359201	15	65062034	65431023	368990	-	ENSSSCG00000015876	CCDC148	protein_coding
15	65390001	65440000	0.363062	15	65062034	65431023	368990	-	ENSSSCG00000015876	CCDC148	protein_coding
15	65040001	65090000	0.359844	15	65088284	65131789	43506	+	ENSSSCG00000021938	UPP2	protein_coding
15	65390001	65440000	0.363062	15	65430309	65680889	250581	+	ENSSSCG00000015878	PKP4	protein_coding
15	65570001	65620000	0.373161	15	65430309	65680889	250581	+	ENSSSCG00000015878	PKP4	protein_coding
15	65580001	65630000	0.401059	15	65430309	65680889	250581	+	ENSSSCG00000015878	PKP4	protein_coding
15	65590001	65640000	0.405697	15	65430309	65680889	250581	+	ENSSSCG00000015878	PKP4	protein_coding
15	68180001	68230000	0.353846	15	68107546	68208884	101339	+	ENSSSCG00000015892	PSMD14	protein_coding
15	68190001	68240000	0.403227	15	68107546	68208884	101339	+	ENSSSCG00000015892	PSMD14	protein_coding
15	68200001	68250000	0.464288	15	68107546	68208884	101339	+	ENSSSCG00000015892	PSMD14	protein_coding
15	68180001	68230000	0.353846	15	68213562	68222991	9430	+	ENSSSCG00000015891	TBR1	protein_coding
15	68190001	68240000	0.403227	15	68213562	68222991	9430	+	ENSSSCG00000015891	TBR1	protein_coding
15	68200001	68250000	0.464288	15	68213562	68222991	9430	+	ENSSSCG00000015891	TBR1	protein_coding
15	68210001	68260000	0.450422	15	68213562	68222991	9430	+	ENSSSCG00000015891	TBR1	protein_coding
15	68220001	68270000	0.466737	15	68213562	68222991	9430	+	ENSSSCG00000015891	TBR1	protein_coding
15	68180001	68230000	0.353846	15	68224395	68656318	431924	+	ENSSSCG00000015893	SLC4A10	protein_coding
15	68190001	68240000	0.403227	15	68224395	68656318	431924	+	ENSSSCG00000015893	SLC4A10	protein_coding
15	68200001	68250000	0.464288	15	68224395	68656318	431924	+	ENSSSCG00000015893	SLC4A10	protein_coding
15	68210001	68260000	0.450422	15	68224395	68656318	431924	+	ENSSSCG00000015893	SLC4A10	protein_coding
15	68220001	68270000	0.466737	15	68224395	68656318	431924	+	ENSSSCG00000015893	SLC4A10	protein_coding
15	68230001	68280000	0.459478	15	68224395	68656318	431924	+	ENSSSCG00000015893	SLC4A10	protein_coding
15	68240001	68290000	0.455069	15	68224395	68656318	431924	+	ENSSSCG00000015893	SLC4A10	protein_coding
15	68250001	68300000	0.429104	15	68224395	68656318	431924	+	ENSSSCG00000015893	SLC4A10	protein_coding
15	68260001	68310000	0.413188	15	68224395	68656318	431924	+	ENSSSCG00000015893	SLC4A10	protein_coding
15	68270001	68320000	0.378081	15	68224395	68656318	431924	+	ENSSSCG00000015893	SLC4A10	protein_coding
15	68280001	68330000	0.36507	15	68224395	68656318	431924	+	ENSSSCG00000015893	SLC4A10	protein_coding
15	68310001	68360000	0.368825	15	68224395	68656318	431924	+	ENSSSCG00000015893	SLC4A10	protein_coding

15	68320001	68370000	0.388275	15	68224395	68656318	431924	+	ENSSSCG00000015893	SLC4A10	protein_coding
15	68330001	68380000	0.387666	15	68224395	68656318	431924	+	ENSSSCG00000015893	SLC4A10	protein_coding
15	68340001	68390000	0.374227	15	68224395	68656318	431924	+	ENSSSCG00000015893	SLC4A10	protein_coding
15	68280001	68330000	0.36507	15	68325371	68325893	523	-	ENSSSCG00000032982	NA	protein_coding
15	68310001	68360000	0.368825	15	68325371	68325893	523	-	ENSSSCG00000032982	NA	protein_coding
15	68320001	68370000	0.388275	15	68325371	68325893	523	-	ENSSSCG00000032982	NA	protein_coding
15	68330001	68380000	0.387666	15	68370814	68375817	5004	-	ENSSSCG00000044131	NA	pseudogene
15	68340001	68390000	0.374227	15	68370814	68375817	5004	-	ENSSSCG00000044131	NA	pseudogene
15	69080001	69130000	0.355167	15	69059270	69554981	495712	-	ENSSSCG00000015898	KCNH7	protein_coding
15	69090001	69140000	0.385042	15	69059270	69554981	495712	-	ENSSSCG00000015898	KCNH7	protein_coding
15	69100001	69150000	0.387313	15	69059270	69554981	495712	-	ENSSSCG00000015898	KCNH7	protein_coding
15	69110001	69160000	0.404362	15	69059270	69554981	495712	-	ENSSSCG00000015898	KCNH7	protein_coding
15	69120001	69170000	0.447244	15	69059270	69554981	495712	-	ENSSSCG00000015898	KCNH7	protein_coding
15	69130001	69180000	0.441439	15	69059270	69554981	495712	-	ENSSSCG00000015898	KCNH7	protein_coding
15	69140001	69190000	0.442054	15	69059270	69554981	495712	-	ENSSSCG00000015898	KCNH7	protein_coding
15	69150001	69200000	0.439507	15	69059270	69554981	495712	-	ENSSSCG00000015898	KCNH7	protein_coding
15	69160001	69210000	0.456773	15	69059270	69554981	495712	-	ENSSSCG00000015898	KCNH7	protein_coding
15	69170001	69220000	0.437212	15	69059270	69554981	495712	-	ENSSSCG00000015898	KCNH7	protein_coding
15	69180001	69230000	0.407571	15	69059270	69554981	495712	-	ENSSSCG00000015898	KCNH7	protein_coding
15	69190001	69240000	0.362244	15	69059270	69554981	495712	-	ENSSSCG00000015898	KCNH7	protein_coding
15	69200001	69250000	0.374795	15	69059270	69554981	495712	-	ENSSSCG00000015898	KCNH7	protein_coding
15	69210001	69260000	0.377297	15	69059270	69554981	495712	-	ENSSSCG00000015898	KCNH7	protein_coding
15	69220001	69270000	0.396592	15	69059270	69554981	495712	-	ENSSSCG00000015898	KCNH7	protein_coding
15	69230001	69280000	0.39369	15	69059270	69554981	495712	-	ENSSSCG00000015898	KCNH7	protein_coding
15	69240001	69290000	0.389725	15	69059270	69554981	495712	-	ENSSSCG00000015898	KCNH7	protein_coding
15	69250001	69300000	0.387645	15	69059270	69554981	495712	-	ENSSSCG00000015898	KCNH7	protein_coding
15	69260001	69310000	0.378337	15	69059270	69554981	495712	-	ENSSSCG00000015898	KCNH7	protein_coding
15	69270001	69320000	0.361956	15	69059270	69554981	495712	-	ENSSSCG00000015898	KCNH7	protein_coding
15	69280001	69330000	0.389686	15	69059270	69554981	495712	-	ENSSSCG00000015898	KCNH7	protein_coding
15	69340001	69390000	0.352739	15	69059270	69554981	495712	-	ENSSSCG00000015898	KCNH7	protein_coding
15	69440001	69490000	0.368905	15	69059270	69554981	495712	-	ENSSSCG00000015898	KCNH7	protein_coding
15	69450001	69500000	0.418603	15	69059270	69554981	495712	-	ENSSSCG00000015898	KCNH7	protein_coding
15	69460001	69510000	0.438649	15	69059270	69554981	495712	-	ENSSSCG00000015898	KCNH7	protein_coding
15	69470001	69520000	0.466165	15	69059270	69554981	495712	-	ENSSSCG00000015898	KCNH7	protein_coding
15	69480001	69530000	0.495134	15	69059270	69554981	495712	-	ENSSSCG00000015898	KCNH7	protein_coding
15	69490001	69540000	0.463799	15	69059270	69554981	495712	-	ENSSSCG00000015898	KCNH7	protein_coding
15	69500001	69550000	0.405924	15	69059270	69554981	495712	-	ENSSSCG00000015898	KCNH7	protein_coding
15	69510001	69560000	0.385272	15	69059270	69554981	495712	-	ENSSSCG00000015898	KCNH7	protein_coding
15	69340001	69390000	0.352739	15	69366006	69368570	2565	-	ENSSSCG00000047423	NA	protein_coding
15	72280001	72330000	0.372026	15	72293614	72342435	48822	-	ENSSSCG00000015907	GALNT3	protein_coding
15	72290001	72340000	0.40183	15	72293614	72342435	48822	-	ENSSSCG00000015907	GALNT3	protein_coding
15	72300001	72350000	0.359775	15	72293614	72342435	48822	-	ENSSSCG00000015907	GALNT3	protein_coding
15	72660001	72710000	0.356768	15	72529875	72678067	148193	-	ENSSSCG00000024204	SCN1A	protein_coding
15	72670001	72720000	0.36509	15	72529875	72678067	148193	-	ENSSSCG00000024204	SCN1A	protein_coding
15	73590001	73640000	0.376444	15	73433932	73741041	307110	+	ENSSSCG00000015917	XIRP2	protein_coding

15	73600001	73650000	0.400847	15	73433932	73741041	307110	+	ENSSSCG00000015917	XIRP2	protein_coding
15	73610001	73660000	0.440344	15	73433932	73741041	307110	+	ENSSSCG00000015917	XIRP2	protein_coding
15	73620001	73670000	0.487033	15	73433932	73741041	307110	+	ENSSSCG00000015917	XIRP2	protein_coding
15	73630001	73680000	0.483909	15	73433932	73741041	307110	+	ENSSSCG00000015917	XIRP2	protein_coding
15	73640001	73690000	0.484258	15	73433932	73741041	307110	+	ENSSSCG00000015917	XIRP2	protein_coding
15	73650001	73700000	0.490785	15	73433932	73741041	307110	+	ENSSSCG00000015917	XIRP2	protein_coding
15	73660001	73710000	0.444133	15	73433932	73741041	307110	+	ENSSSCG00000015917	XIRP2	protein_coding
15	73670001	73720000	0.400058	15	73433932	73741041	307110	+	ENSSSCG00000015917	XIRP2	protein_coding
15	73680001	73730000	0.370465	15	73433932	73741041	307110	+	ENSSSCG00000015917	XIRP2	protein_coding
15	74190001	74240000	0.354955	15	74236390	74341441	105052	+	ENSSSCG00000034351	B3GALT1	protein_coding
18	31130001	31180000	0.353041	18	31144296	31144402	107	+	ENSSSCG00000039642	U6	snRNA
18	31140001	31190000	0.397204	18	31144296	31144402	107	+	ENSSSCG00000039642	U6	snRNA
18	31130001	31180000	0.353041	18	31178731	31178873	143	-	ENSSSCG00000018679	U4	snRNA
18	31140001	31190000	0.397204	18	31178731	31178873	143	-	ENSSSCG00000018679	U4	snRNA
18	17830001	17880000	0.354699	18	17843842	17851772	7931	-	ENSSSCG00000050311	NA	lncRNA
18	17840001	17890000	0.382521	18	17843842	17851772	7931	-	ENSSSCG00000050311	NA	lncRNA
18	17850001	17900000	0.37667	18	17843842	17851772	7931	-	ENSSSCG00000050311	NA	lncRNA
18	17840001	17890000	0.382521	18	17886662	17889559	2898	-	ENSSSCG00000049852	NA	lncRNA
18	17850001	17900000	0.37667	18	17886662	17889559	2898	-	ENSSSCG00000049852	NA	lncRNA
18	17860001	17910000	0.384293	18	17886662	17889559	2898	-	ENSSSCG00000049852	NA	lncRNA
18	17870001	17920000	0.373076	18	17886662	17889559	2898	-	ENSSSCG00000049852	NA	lncRNA
18	50710001	50760000	0.378985	18	50721507	50726785	5279	+	ENSSSCG00000049289	NA	lncRNA
18	50720001	50770000	0.430801	18	50721507	50726785	5279	+	ENSSSCG00000049289	NA	lncRNA
18	53070001	53120000	0.382128	18	53052829	53400687	347859	-	ENSSSCG00000045227	NA	lncRNA
18	53080001	53130000	0.389906	18	53052829	53400687	347859	-	ENSSSCG00000045227	NA	lncRNA
18	53090001	53140000	0.416867	18	53052829	53400687	347859	-	ENSSSCG00000045227	NA	lncRNA
18	53100001	53150000	0.450616	18	53052829	53400687	347859	-	ENSSSCG00000045227	NA	lncRNA
18	53110001	53160000	0.406997	18	53052829	53400687	347859	-	ENSSSCG00000045227	NA	lncRNA
18	53130001	53180000	0.402553	18	53052829	53400687	347859	-	ENSSSCG00000045227	NA	lncRNA
18	53140001	53190000	0.392874	18	53052829	53400687	347859	-	ENSSSCG00000045227	NA	lncRNA
18	53150001	53200000	0.399184	18	53052829	53400687	347859	-	ENSSSCG00000045227	NA	lncRNA
18	53160001	53210000	0.403653	18	53052829	53400687	347859	-	ENSSSCG00000045227	NA	lncRNA
18	53170001	53220000	0.4549	18	53052829	53400687	347859	-	ENSSSCG00000045227	NA	lncRNA
18	53180001	53230000	0.439689	18	53052829	53400687	347859	-	ENSSSCG00000045227	NA	lncRNA
18	53190001	53240000	0.429528	18	53052829	53400687	347859	-	ENSSSCG00000045227	NA	lncRNA
18	53200001	53250000	0.432745	18	53052829	53400687	347859	-	ENSSSCG00000045227	NA	lncRNA
18	53210001	53260000	0.437717	18	53052829	53400687	347859	-	ENSSSCG00000045227	NA	lncRNA
18	54550001	54600000	0.432659	18	54590774	54601054	10281	-	ENSSSCG00000051549	NA	lncRNA
18	54560001	54610000	0.405402	18	54590774	54601054	10281	-	ENSSSCG00000051549	NA	lncRNA
18	17830001	17880000	0.354699	18	17853313	17974185	120873	+	ENSSSCG00000051610	NA	lncRNA
18	17840001	17890000	0.382521	18	17853313	17974185	120873	+	ENSSSCG00000051610	NA	lncRNA
18	17850001	17900000	0.37667	18	17853313	17974185	120873	+	ENSSSCG00000051610	NA	lncRNA
18	17860001	17910000	0.384293	18	17853313	17974185	120873	+	ENSSSCG00000051610	NA	lncRNA
18	17870001	17920000	0.373076	18	17853313	17974185	120873	+	ENSSSCG00000051610	NA	lncRNA
18	13630001	13680000	0.392873	18	13593771	13636179	42409	+	ENSSSCG00000016527	NA	protein_coding

18	13630001	13680000	0.392873	18	13674301	13811976	137676	-	ENSSSCG00000030386	NUP205	protein_coding
18	13640001	13690000	0.372776	18	13674301	13811976	137676	-	ENSSSCG00000030386	NUP205	protein_coding
18	14330001	14380000	0.376785	18	14267955	14539017	271063	-	ENSSSCG00000016535	CALD1	protein_coding
18	14340001	14390000	0.364588	18	14267955	14539017	271063	-	ENSSSCG00000016535	CALD1	protein_coding
18	14350001	14400000	0.389557	18	14267955	14539017	271063	-	ENSSSCG00000016535	CALD1	protein_coding
18	14360001	14410000	0.39043	18	14267955	14539017	271063	-	ENSSSCG00000016535	CALD1	protein_coding
18	17830001	17880000	0.354699	18	17501855	17832503	330649	-	ENSSSCG00000016549	MKLN1	protein_coding
18	18380001	18430000	0.383554	18	18416259	18462575	46317	+	ENSSSCG00000016556	CEP41	protein_coding
18	18390001	18440000	0.374714	18	18416259	18462575	46317	+	ENSSSCG00000016556	CEP41	protein_coding
18	18400001	18450000	0.378117	18	18416259	18462575	46317	+	ENSSSCG00000016556	CEP41	protein_coding
18	18410001	18460000	0.356101	18	18416259	18462575	46317	+	ENSSSCG00000016556	CEP41	protein_coding
18	18470001	18520000	0.354562	18	18461721	18598032	136312	-	ENSSSCG00000016557	CPA1	protein_coding
18	18480001	18530000	0.36816	18	18461721	18598032	136312	-	ENSSSCG00000016557	CPA1	protein_coding
18	18490001	18540000	0.395179	18	18461721	18598032	136312	-	ENSSSCG00000016557	CPA1	protein_coding
18	18500001	18550000	0.417104	18	18461721	18598032	136312	-	ENSSSCG00000016557	CPA1	protein_coding
18	18510001	18560000	0.404661	18	18461721	18598032	136312	-	ENSSSCG00000016557	CPA1	protein_coding
18	18520001	18570000	0.366393	18	18461721	18598032	136312	-	ENSSSCG00000016557	CPA1	protein_coding
18	18490001	18540000	0.395179	18	18530759	18581003	50245	-	ENSSSCG00000016555	CPA4	protein_coding
18	18500001	18550000	0.417104	18	18530759	18581003	50245	-	ENSSSCG00000016555	CPA4	protein_coding
18	18510001	18560000	0.404661	18	18530759	18581003	50245	-	ENSSSCG00000016555	CPA4	protein_coding
18	18520001	18570000	0.366393	18	18530759	18581003	50245	-	ENSSSCG00000016555	CPA4	protein_coding
18	18510001	18560000	0.404661	18	18557518	18577682	20165	-	ENSSSCG00000016559	CPA2	protein_coding
18	18520001	18570000	0.366393	18	18557518	18577682	20165	-	ENSSSCG00000016559	CPA2	protein_coding
18	18610001	18660000	0.356225	18	18604028	18613277	9250	-	ENSSSCG00000016562	SSMEM1	protein_coding
18	18610001	18660000	0.356225	18	18615258	18644708	29451	+	ENSSSCG00000016563	TMEM209	protein_coding
18	18620001	18670000	0.397332	18	18615258	18644708	29451	+	ENSSSCG00000016563	TMEM209	protein_coding
18	18630001	18680000	0.387854	18	18615258	18644708	29451	+	ENSSSCG00000016563	TMEM209	protein_coding
18	18640001	18690000	0.389417	18	18615258	18644708	29451	+	ENSSSCG00000016563	TMEM209	protein_coding
18	18620001	18670000	0.397332	18	18667364	18723067	55704	-	ENSSSCG00000027131	KLHDC10	protein_coding
18	18630001	18680000	0.387854	18	18667364	18723067	55704	-	ENSSSCG00000027131	KLHDC10	protein_coding
18	18640001	18690000	0.389417	18	18667364	18723067	55704	-	ENSSSCG00000027131	KLHDC10	protein_coding
18	18650001	18700000	0.388987	18	18667364	18723067	55704	-	ENSSSCG00000027131	KLHDC10	protein_coding
18	18660001	18710000	0.361173	18	18667364	18723067	55704	-	ENSSSCG00000027131	KLHDC10	protein_coding
18	18690001	18740000	0.355341	18	18667364	18723067	55704	-	ENSSSCG00000027131	KLHDC10	protein_coding
18	18700001	18750000	0.401579	18	18667364	18723067	55704	-	ENSSSCG00000027131	KLHDC10	protein_coding
18	18710001	18760000	0.458296	18	18667364	18723067	55704	-	ENSSSCG00000027131	KLHDC10	protein_coding
18	18720001	18770000	0.451861	18	18667364	18723067	55704	-	ENSSSCG00000027131	KLHDC10	protein_coding
18	18700001	18750000	0.401579	18	18740845	18775877	35033	+	ENSSSCG00000016565	ZC3HC1	protein_coding
18	18710001	18760000	0.458296	18	18740845	18775877	35033	+	ENSSSCG00000016565	ZC3HC1	protein_coding
18	18720001	18770000	0.451861	18	18740845	18775877	35033	+	ENSSSCG00000016565	ZC3HC1	protein_coding
18	18730001	18780000	0.441462	18	18740845	18775877	35033	+	ENSSSCG00000016565	ZC3HC1	protein_coding
18	18740001	18790000	0.398606	18	18740845	18775877	35033	+	ENSSSCG00000016565	ZC3HC1	protein_coding
18	20280001	20330000	0.384222	18	20261476	20653590	392115	-	ENSSSCG00000016590	SND1	protein_coding
18	20290001	20340000	0.413968	18	20261476	20653590	392115	-	ENSSSCG00000016590	SND1	protein_coding
18	20300001	20350000	0.425549	18	20261476	20653590	392115	-	ENSSSCG00000016590	SND1	protein_coding

18	20310001	20360000	0.417638	18	20261476	20653590	392115	-	ENSSSCG00000016590	SND1	protein_coding
18	20320001	20370000	0.427693	18	20261476	20653590	392115	-	ENSSSCG00000016590	SND1	protein_coding
18	20330001	20380000	0.456983	18	20261476	20653590	392115	-	ENSSSCG00000016590	SND1	protein_coding
18	20340001	20390000	0.578841	18	20261476	20653590	392115	-	ENSSSCG00000016590	SND1	protein_coding
18	20350001	20400000	0.543546	18	20261476	20653590	392115	-	ENSSSCG00000016590	SND1	protein_coding
18	20360001	20410000	0.462964	18	20261476	20653590	392115	-	ENSSSCG00000016590	SND1	protein_coding
18	20370001	20420000	0.359824	18	20261476	20653590	392115	-	ENSSSCG00000016590	SND1	protein_coding
18	20280001	20330000	0.384222	18	20319699	20322875	3177	+	ENSSSCG00000016589	LRRC4	protein_coding
18	20290001	20340000	0.413968	18	20319699	20322875	3177	+	ENSSSCG00000016589	LRRC4	protein_coding
18	20300001	20350000	0.425549	18	20319699	20322875	3177	+	ENSSSCG00000016589	LRRC4	protein_coding
18	20310001	20360000	0.417638	18	20319699	20322875	3177	+	ENSSSCG00000016589	LRRC4	protein_coding
18	20320001	20370000	0.427693	18	20319699	20322875	3177	+	ENSSSCG00000016589	LRRC4	protein_coding
18	50710001	50760000	0.378985	18	50705391	50715037	9647	+	ENSSSCG00000016745	DDX56	protein_coding
18	50710001	50760000	0.378985	18	50726894	50757649	30756	+	ENSSSCG00000016746	NPC1L1	protein_coding
18	50720001	50770000	0.430801	18	50726894	50757649	30756	+	ENSSSCG00000016746	NPC1L1	protein_coding
18	50730001	50780000	0.438128	18	50726894	50757649	30756	+	ENSSSCG00000016746	NPC1L1	protein_coding
18	50740001	50790000	0.470649	18	50726894	50757649	30756	+	ENSSSCG00000016746	NPC1L1	protein_coding
18	50750001	50800000	0.466911	18	50726894	50757649	30756	+	ENSSSCG00000016746	NPC1L1	protein_coding
18	50710001	50760000	0.378985	18	50759095	50830334	71240	+	ENSSSCG00000016747	NUDCD3	protein_coding
18	50720001	50770000	0.430801	18	50759095	50830334	71240	+	ENSSSCG00000016747	NUDCD3	protein_coding
18	50730001	50780000	0.438128	18	50759095	50830334	71240	+	ENSSSCG00000016747	NUDCD3	protein_coding
18	50740001	50790000	0.470649	18	50759095	50830334	71240	+	ENSSSCG00000016747	NUDCD3	protein_coding
18	50750001	50800000	0.466911	18	50759095	50830334	71240	+	ENSSSCG00000016747	NUDCD3	protein_coding
18	50760001	50810000	0.501859	18	50759095	50830334	71240	+	ENSSSCG00000016747	NUDCD3	protein_coding
18	50770001	50820000	0.507665	18	50759095	50830334	71240	+	ENSSSCG00000016747	NUDCD3	protein_coding
18	50780001	50830000	0.512692	18	50759095	50830334	71240	+	ENSSSCG00000016747	NUDCD3	protein_coding
18	50790001	50840000	0.460877	18	50759095	50830334	71240	+	ENSSSCG00000016747	NUDCD3	protein_coding
18	50800001	50850000	0.412321	18	50759095	50830334	71240	+	ENSSSCG00000016747	NUDCD3	protein_coding
18	50810001	50860000	0.371902	18	50759095	50830334	71240	+	ENSSSCG00000016747	NUDCD3	protein_coding
18	50750001	50800000	0.466911	18	50790472	51028917	238446	+	ENSSSCG00000016751	GCK	protein_coding
18	50760001	50810000	0.501859	18	50790472	51028917	238446	+	ENSSSCG00000016751	GCK	protein_coding
18	50770001	50820000	0.507665	18	50790472	51028917	238446	+	ENSSSCG00000016751	GCK	protein_coding
18	50780001	50830000	0.512692	18	50790472	51028917	238446	+	ENSSSCG00000016751	GCK	protein_coding
18	50790001	50840000	0.460877	18	50790472	51028917	238446	+	ENSSSCG00000016751	GCK	protein_coding
18	50800001	50850000	0.412321	18	50790472	51028917	238446	+	ENSSSCG00000016751	GCK	protein_coding
18	50810001	50860000	0.371902	18	50790472	51028917	238446	+	ENSSSCG00000016751	GCK	protein_coding
18	50840001	50890000	0.358045	18	50790472	51028917	238446	+	ENSSSCG00000016751	GCK	protein_coding
18	50850001	50900000	0.388304	18	50790472	51028917	238446	+	ENSSSCG00000016751	GCK	protein_coding
18	50860001	50910000	0.401775	18	50790472	51028917	238446	+	ENSSSCG00000016751	GCK	protein_coding
18	50870001	50920000	0.426481	18	50790472	51028917	238446	+	ENSSSCG00000016751	GCK	protein_coding
18	50880001	50930000	0.460624	18	50790472	51028917	238446	+	ENSSSCG00000016751	GCK	protein_coding
18	50890001	50940000	0.414428	18	50790472	51028917	238446	+	ENSSSCG00000016751	GCK	protein_coding
18	50900001	50950000	0.373727	18	50790472	51028917	238446	+	ENSSSCG00000016751	GCK	protein_coding
18	50910001	50960000	0.354508	18	50790472	51028917	238446	+	ENSSSCG00000016751	GCK	protein_coding
18	50840001	50890000	0.358045	18	50861466	50957945	96480	+	ENSSSCG00000039535	CAMK2B	protein_coding

18	50850001	50900000	0.388304	18	50861466	50957945	96480	+	ENSSSCG00000039535	CAMK2B	protein_coding
18	50860001	50910000	0.401775	18	50861466	50957945	96480	+	ENSSSCG00000039535	CAMK2B	protein_coding
18	50870001	50920000	0.426481	18	50861466	50957945	96480	+	ENSSSCG00000039535	CAMK2B	protein_coding
18	50880001	50930000	0.460624	18	50861466	50957945	96480	+	ENSSSCG00000039535	CAMK2B	protein_coding
18	50890001	50940000	0.414428	18	50861466	50957945	96480	+	ENSSSCG00000039535	CAMK2B	protein_coding
18	50900001	50950000	0.373727	18	50861466	50957945	96480	+	ENSSSCG00000039535	CAMK2B	protein_coding
18	50910001	50960000	0.354508	18	50861466	50957945	96480	+	ENSSSCG00000039535	CAMK2B	protein_coding
18	51220001	51270000	0.370424	18	51220943	51327498	106556	+	ENSSSCG00000027491	NA	protein_coding
18	51230001	51280000	0.416018	18	51220943	51327498	106556	+	ENSSSCG00000027491	NA	protein_coding
18	51240001	51290000	0.417993	18	51220943	51327498	106556	+	ENSSSCG00000027491	NA	protein_coding
18	51250001	51300000	0.39603	18	51220943	51327498	106556	+	ENSSSCG00000027491	NA	protein_coding
18	51260001	51310000	0.37227	18	51220943	51327498	106556	+	ENSSSCG00000027491	NA	protein_coding
18	51830001	51880000	0.362483	18	51829032	51909329	80298	-	ENSSSCG00000025060	NA	protein_coding
18	54240001	54290000	0.35939	18	53639593	54283251	643659	-	ENSSSCG00000035581	SUGCT	protein_coding
18	54250001	54300000	0.37005	18	53639593	54283251	643659	-	ENSSSCG00000035581	SUGCT	protein_coding
18	54260001	54310000	0.357135	18	53639593	54283251	643659	-	ENSSSCG00000035581	SUGCT	protein_coding
18	54270001	54320000	0.394771	18	53639593	54283251	643659	-	ENSSSCG00000035581	SUGCT	protein_coding
18	54280001	54330000	0.378796	18	53639593	54283251	643659	-	ENSSSCG00000035581	SUGCT	protein_coding
18	54240001	54290000	0.35939	18	54282829	54285820	2992	+	ENSSSCG00000016768	NA	protein_coding
18	54250001	54300000	0.37005	18	54282829	54285820	2992	+	ENSSSCG00000016768	NA	protein_coding
18	54260001	54310000	0.357135	18	54282829	54285820	2992	+	ENSSSCG00000016768	NA	protein_coding
18	54270001	54320000	0.394771	18	54282829	54285820	2992	+	ENSSSCG00000016768	NA	protein_coding
18	54280001	54330000	0.378796	18	54282829	54285820	2992	+	ENSSSCG00000016768	NA	protein_coding
18	54260001	54310000	0.357135	18	54308144	54429939	121796	-	ENSSSCG00000016769	CDK13	protein_coding
18	54270001	54320000	0.394771	18	54308144	54429939	121796	-	ENSSSCG00000016769	CDK13	protein_coding
18	54280001	54330000	0.378796	18	54308144	54429939	121796	-	ENSSSCG00000016769	CDK13	protein_coding
18	54290001	54340000	0.36241	18	54308144	54429939	121796	-	ENSSSCG00000016769	CDK13	protein_coding
18	54320001	54370000	0.355856	18	54308144	54429939	121796	-	ENSSSCG00000016769	CDK13	protein_coding
18	54510001	54560000	0.39221	18	54550238	54588980	38743	-	ENSSSCG00000048439	RALA	protein_coding
18	54520001	54570000	0.503966	18	54550238	54588980	38743	-	ENSSSCG00000048439	RALA	protein_coding
18	54530001	54580000	0.490002	18	54550238	54588980	38743	-	ENSSSCG00000048439	RALA	protein_coding
18	54540001	54590000	0.465777	18	54550238	54588980	38743	-	ENSSSCG00000048439	RALA	protein_coding
18	54550001	54600000	0.432659	18	54550238	54588980	38743	-	ENSSSCG00000048439	RALA	protein_coding
18	54560001	54610000	0.405402	18	54550238	54588980	38743	-	ENSSSCG00000048439	RALA	protein_coding
18	54630001	54680000	0.370271	18	54647316	54663201	15886	-	ENSSSCG00000030497	YAE1	protein_coding
18	54640001	54690000	0.388569	18	54647316	54663201	15886	-	ENSSSCG00000030497	YAE1	protein_coding
18	54650001	54700000	0.398568	18	54647316	54663201	15886	-	ENSSSCG00000030497	YAE1	protein_coding
18	54660001	54710000	0.426381	18	54647316	54663201	15886	-	ENSSSCG00000030497	YAE1	protein_coding
18	54700001	54750000	0.37246	18	54748386	55205561	457176	-	ENSSSCG00000037764	POU6F2	protein_coding
18	54710001	54760000	0.381281	18	54748386	55205561	457176	-	ENSSSCG00000037764	POU6F2	protein_coding
18	54720001	54770000	0.361297	18	54748386	55205561	457176	-	ENSSSCG00000037764	POU6F2	protein_coding
18	54730001	54780000	0.378411	18	54748386	55205561	457176	-	ENSSSCG00000037764	POU6F2	protein_coding
18	54740001	54790000	0.42865	18	54748386	55205561	457176	-	ENSSSCG00000037764	POU6F2	protein_coding
18	54750001	54800000	0.422487	18	54748386	55205561	457176	-	ENSSSCG00000037764	POU6F2	protein_coding
18	54760001	54810000	0.370663	18	54748386	55205561	457176	-	ENSSSCG00000037764	POU6F2	protein_coding

18	54770001	54820000	0.378605	18	54748386	55205561	457176	-	ENSSSCG00000037764	POU6F2	protein_coding
18	54950001	55000000	0.354663	18	54748386	55205561	457176	-	ENSSSCG00000037764	POU6F2	protein_coding
18	55120001	55170000	0.352673	18	54748386	55205561	457176	-	ENSSSCG00000037764	POU6F2	protein_coding
18	55130001	55180000	0.355323	18	54748386	55205561	457176	-	ENSSSCG00000037764	POU6F2	protein_coding
2	59850001	59900000	0.357091	2	59893333	59893465	133	-	ENSSSCG00000018685	NA	snoRNA
2	142260001	142310000	0.371357	2	142279273	142279373	101	-	ENSSSCG00000019080	U6	snRNA
2	142270001	142320000	0.406065	2	142279273	142279373	101	-	ENSSSCG00000019080	U6	snRNA
2	75440001	75490000	0.353754	2	75479134	75479230	97	+	ENSSSCG00000022205	U6	snRNA
2	75450001	75500000	0.363226	2	75479134	75479230	97	+	ENSSSCG00000022205	U6	snRNA
2	98740001	98790000	0.383978	2	98787477	98787626	150	-	ENSSSCG00000020474	U1	snRNA
2	51980001	52030000	0.357673	2	52011018	52030629	19612	-	ENSSSCG00000048601	NA	lncRNA
2	51990001	52040000	0.36625	2	52011018	52030629	19612	-	ENSSSCG00000048601	NA	lncRNA
2	52000001	52050000	0.354901	2	52011018	52030629	19612	-	ENSSSCG00000048601	NA	lncRNA
2	56110001	56160000	0.367435	2	56066406	56141066	74661	+	ENSSSCG00000050335	NA	lncRNA
2	56120001	56170000	0.422602	2	56066406	56141066	74661	+	ENSSSCG00000050335	NA	lncRNA
2	56130001	56180000	0.463413	2	56066406	56141066	74661	+	ENSSSCG00000050335	NA	lncRNA
2	56140001	56190000	0.443077	2	56066406	56141066	74661	+	ENSSSCG00000050335	NA	lncRNA
2	57070001	57120000	0.382069	2	57112278	57113874	1597	-	ENSSSCG00000051038	NA	lncRNA
2	57080001	57130000	0.391358	2	57112278	57113874	1597	-	ENSSSCG00000051038	NA	lncRNA
2	57090001	57140000	0.400807	2	57112278	57113874	1597	-	ENSSSCG00000051038	NA	lncRNA
2	57100001	57150000	0.431267	2	57112278	57113874	1597	-	ENSSSCG00000051038	NA	lncRNA
2	57110001	57160000	0.440226	2	57112278	57113874	1597	-	ENSSSCG00000051038	NA	lncRNA
2	57160001	57210000	0.387122	2	57209190	57213966	4777	+	ENSSSCG00000046829	NA	lncRNA
2	57170001	57220000	0.429689	2	57209190	57213966	4777	+	ENSSSCG00000046829	NA	lncRNA
2	57180001	57230000	0.422076	2	57209190	57213966	4777	+	ENSSSCG00000046829	NA	lncRNA
2	57190001	57240000	0.425099	2	57209190	57213966	4777	+	ENSSSCG00000046829	NA	lncRNA
2	57200001	57250000	0.40269	2	57209190	57213966	4777	+	ENSSSCG00000046829	NA	lncRNA
2	57210001	57260000	0.393931	2	57209190	57213966	4777	+	ENSSSCG00000046829	NA	lncRNA
2	57990001	58040000	0.356062	2	58021440	58031477	10038	+	ENSSSCG00000045468	NA	lncRNA
2	58000001	58050000	0.368139	2	58021440	58031477	10038	+	ENSSSCG00000045468	NA	lncRNA
2	64920001	64970000	0.361595	2	64963333	64970001	6669	+	ENSSSCG00000051583	NA	lncRNA
2	64930001	64980000	0.370848	2	64963333	64970001	6669	+	ENSSSCG00000051583	NA	lncRNA
2	64940001	64990000	0.365633	2	64963333	64970001	6669	+	ENSSSCG00000051583	NA	lncRNA
2	64950001	65000000	0.371071	2	64963333	64970001	6669	+	ENSSSCG00000051583	NA	lncRNA
2	64960001	65010000	0.377566	2	64963333	64970001	6669	+	ENSSSCG00000051583	NA	lncRNA
2	64970001	65020000	0.361119	2	64963333	64970001	6669	+	ENSSSCG00000051583	NA	lncRNA
2	73360001	73410000	0.368102	2	73401027	73411574	10548	+	ENSSSCG00000042523	NA	lncRNA
2	73360001	73410000	0.368102	2	73401474	73411582	10109	-	ENSSSCG00000049022	NA	lncRNA
2	79060001	79110000	0.355608	2	79021640	79099938	78299	+	ENSSSCG00000044467	NA	lncRNA
2	79070001	79120000	0.35615	2	79021640	79099938	78299	+	ENSSSCG00000044467	NA	lncRNA
2	79080001	79130000	0.396092	2	79021640	79099938	78299	+	ENSSSCG00000044467	NA	lncRNA
2	79060001	79110000	0.355608	2	79071005	79076570	5566	+	ENSSSCG00000041652	NA	lncRNA
2	79070001	79120000	0.35615	2	79071005	79076570	5566	+	ENSSSCG00000041652	NA	lncRNA
2	91300001	91350000	0.487639	2	91341725	91341860	136	-	ENSSSCG00000018205	NA	snoRNA
2	91310001	91360000	0.517895	2	91341725	91341860	136	-	ENSSSCG00000018205	NA	snoRNA

2	91320001	91370000	0.537275	2	91341725	91341860	136	-	ENSSSCG00000018205	NA	snoRNA
2	91330001	91380000	0.551391	2	91341725	91341860	136	-	ENSSSCG00000018205	NA	snoRNA
2	91340001	91390000	0.515854	2	91341725	91341860	136	-	ENSSSCG00000018205	NA	snoRNA
2	83390001	83440000	0.358731	2	83262737	83425150	162414	+	ENSSSCG00000033215	NA	lncRNA
2	83400001	83450000	0.372915	2	83262737	83425150	162414	+	ENSSSCG00000033215	NA	lncRNA
2	91300001	91350000	0.487639	2	91265719	91358271	92553	+	ENSSSCG00000044447	NA	lncRNA
2	91310001	91360000	0.517895	2	91265719	91358271	92553	+	ENSSSCG00000044447	NA	lncRNA
2	91320001	91370000	0.537275	2	91265719	91358271	92553	+	ENSSSCG00000044447	NA	lncRNA
2	91330001	91380000	0.551391	2	91265719	91358271	92553	+	ENSSSCG00000044447	NA	lncRNA
2	91340001	91390000	0.515854	2	91265719	91358271	92553	+	ENSSSCG00000044447	NA	lncRNA
2	91350001	91400000	0.472062	2	91265719	91358271	92553	+	ENSSSCG00000044447	NA	lncRNA
2	91300001	91350000	0.487639	2	91287931	91319479	31549	-	ENSSSCG00000042308	NA	lncRNA
2	91310001	91360000	0.517895	2	91287931	91319479	31549	-	ENSSSCG00000042308	NA	lncRNA
2	91300001	91350000	0.487639	2	91295887	91301876	5990	+	ENSSSCG00000050488	NA	lncRNA
2	91300001	91350000	0.487639	2	91318551	91322545	3995	+	ENSSSCG00000049180	NA	lncRNA
2	91310001	91360000	0.517895	2	91318551	91322545	3995	+	ENSSSCG00000049180	NA	lncRNA
2	91320001	91370000	0.537275	2	91318551	91322545	3995	+	ENSSSCG00000049180	NA	lncRNA
2	91300001	91350000	0.487639	2	91343556	91347264	3709	+	ENSSSCG00000048050	NA	lncRNA
2	91310001	91360000	0.517895	2	91343556	91347264	3709	+	ENSSSCG00000048050	NA	lncRNA
2	91320001	91370000	0.537275	2	91343556	91347264	3709	+	ENSSSCG00000048050	NA	lncRNA
2	91330001	91380000	0.551391	2	91343556	91347264	3709	+	ENSSSCG00000048050	NA	lncRNA
2	91340001	91390000	0.515854	2	91343556	91347264	3709	+	ENSSSCG00000048050	NA	lncRNA
2	95200001	95250000	0.380304	2	95241744	95375930	134187	+	ENSSSCG00000041003	NA	lncRNA
2	95210001	95260000	0.425299	2	95241744	95375930	134187	+	ENSSSCG00000041003	NA	lncRNA
2	95220001	95270000	0.425867	2	95241744	95375930	134187	+	ENSSSCG00000041003	NA	lncRNA
2	95230001	95280000	0.396459	2	95241744	95375930	134187	+	ENSSSCG00000041003	NA	lncRNA
2	96810001	96860000	0.377449	2	96795581	96826136	30556	+	ENSSSCG00000045004	NA	lncRNA
2	96820001	96870000	0.440782	2	96795581	96826136	30556	+	ENSSSCG00000045004	NA	lncRNA
2	97260001	97310000	0.378022	2	97286797	97436352	149556	-	ENSSSCG00000049927	NA	lncRNA
2	97260001	97310000	0.378022	2	97303085	97306376	3292	-	ENSSSCG00000041572	NA	lncRNA
2	99560001	99610000	0.356735	2	99489398	99680988	191591	-	ENSSSCG00000046494	NA	lncRNA
2	99570001	99620000	0.391089	2	99489398	99680988	191591	-	ENSSSCG00000046494	NA	lncRNA
2	99580001	99630000	0.398054	2	99489398	99680988	191591	-	ENSSSCG00000046494	NA	lncRNA
2	99590001	99640000	0.399273	2	99489398	99680988	191591	-	ENSSSCG00000046494	NA	lncRNA
2	99600001	99650000	0.355119	2	99489398	99680988	191591	-	ENSSSCG00000046494	NA	lncRNA
2	99610001	99660000	0.380325	2	99489398	99680988	191591	-	ENSSSCG00000046494	NA	lncRNA
2	99620001	99670000	0.372394	2	99489398	99680988	191591	-	ENSSSCG00000046494	NA	lncRNA
2	99630001	99680000	0.37689	2	99489398	99680988	191591	-	ENSSSCG00000046494	NA	lncRNA
2	99640001	99690000	0.368347	2	99489398	99680988	191591	-	ENSSSCG00000046494	NA	lncRNA
2	99650001	99700000	0.354021	2	99489398	99680988	191591	-	ENSSSCG00000046494	NA	lncRNA
2	57110001	57160000	0.440226	2	57150125	57150230	106	-	ENSSSCG00000045818	U2	snRNA
2	57120001	57170000	0.422802	2	57150125	57150230	106	-	ENSSSCG00000045818	U2	snRNA
2	57130001	57180000	0.403959	2	57150125	57150230	106	-	ENSSSCG00000045818	U2	snRNA
2	57140001	57190000	0.40155	2	57150125	57150230	106	-	ENSSSCG00000045818	U2	snRNA
2	57150001	57200000	0.410057	2	57150125	57150230	106	-	ENSSSCG00000045818	U2	snRNA

2	57150001	57200000	0.410057	2	57199185	57199291	107	+	ENSSSCG00000034050	U6	snRNA
2	57160001	57210000	0.387122	2	57199185	57199291	107	+	ENSSSCG00000034050	U6	snRNA
2	57170001	57220000	0.429689	2	57199185	57199291	107	+	ENSSSCG00000034050	U6	snRNA
2	57180001	57230000	0.422076	2	57199185	57199291	107	+	ENSSSCG00000034050	U6	snRNA
2	57190001	57240000	0.425099	2	57199185	57199291	107	+	ENSSSCG00000034050	U6	snRNA
2	18730001	18780000	0.363579	2	18747520	18747591	72	-	ENSSSCG00000019175	ssc-mir-670	miRNA
2	18740001	18790000	0.433621	2	18747520	18747591	72	-	ENSSSCG00000019175	ssc-mir-670	miRNA
2	51980001	52030000	0.357673	2	51992121	51993056	936	-	ENSSSCG00000026598	NA	protein_coding
2	51990001	52040000	0.36625	2	51992121	51993056	936	-	ENSSSCG00000026598	NA	protein_coding
2	57990001	58040000	0.356062	2	58005590	58007764	2175	-	ENSSSCG00000045996	NA	processed_pseudogene
2	58000001	58050000	0.368139	2	58005590	58007764	2175	-	ENSSSCG00000045996	NA	processed_pseudogene
2	127160001	127210000	0.377063	2	127202845	127210659	7815	+	ENSSSCG00000042143	NA	lncRNA
2	127170001	127220000	0.392264	2	127202845	127210659	7815	+	ENSSSCG00000042143	NA	lncRNA
2	127180001	127230000	0.391539	2	127202845	127210659	7815	+	ENSSSCG00000042143	NA	lncRNA
2	127190001	127240000	0.380011	2	127202845	127210659	7815	+	ENSSSCG00000042143	NA	lncRNA
2	127200001	127250000	0.359131	2	127202845	127210659	7815	+	ENSSSCG00000042143	NA	lncRNA
2	56240001	56290000	0.469207	2	56286322	56291027	4706	+	ENSSSCG00000048187	NA	protein_coding
2	56250001	56300000	0.463122	2	56286322	56291027	4706	+	ENSSSCG00000048187	NA	protein_coding
2	56260001	56310000	0.444726	2	56286322	56291027	4706	+	ENSSSCG00000048187	NA	protein_coding
2	56270001	56320000	0.470141	2	56286322	56291027	4706	+	ENSSSCG00000048187	NA	protein_coding
2	56280001	56330000	0.470604	2	56286322	56291027	4706	+	ENSSSCG00000048187	NA	protein_coding
2	56290001	56340000	0.473332	2	56286322	56291027	4706	+	ENSSSCG00000048187	NA	protein_coding
2	56110001	56160000	0.367435	2	56141935	56145347	3413	+	ENSSSCG00000013952	NA	protein_coding
2	56120001	56170000	0.422602	2	56141935	56145347	3413	+	ENSSSCG00000013952	NA	protein_coding
2	56130001	56180000	0.463413	2	56141935	56145347	3413	+	ENSSSCG00000013952	NA	protein_coding
2	56140001	56190000	0.443077	2	56141935	56145347	3413	+	ENSSSCG00000013952	NA	protein_coding
2	72100001	72150000	0.381963	2	72142428	72146611	4184	+	ENSSSCG00000050577	NA	protein_coding
2	54910001	54960000	0.429032	2	54951053	54952054	1002	-	ENSSSCG00000044888	NA	protein_coding
2	54920001	54970000	0.438494	2	54951053	54952054	1002	-	ENSSSCG00000044888	NA	protein_coding
2	54930001	54980000	0.439105	2	54951053	54952054	1002	-	ENSSSCG00000044888	NA	protein_coding
2	54940001	54990000	0.410964	2	54951053	54952054	1002	-	ENSSSCG00000044888	NA	protein_coding
2	54930001	54980000	0.439105	2	54972577	54979557	6981	+	ENSSSCG00000042129	NA	protein_coding
2	54940001	54990000	0.410964	2	54972577	54979557	6981	+	ENSSSCG00000042129	NA	protein_coding
2	91300001	91350000	0.487639	2	91230738	91358164	127427	-	ENSSSCG00000048661	NA	lncRNA
2	91310001	91360000	0.517895	2	91230738	91358164	127427	-	ENSSSCG00000048661	NA	lncRNA
2	91320001	91370000	0.537275	2	91230738	91358164	127427	-	ENSSSCG00000048661	NA	lncRNA
2	91330001	91380000	0.551391	2	91230738	91358164	127427	-	ENSSSCG00000048661	NA	lncRNA
2	91340001	91390000	0.515854	2	91230738	91358164	127427	-	ENSSSCG00000048661	NA	lncRNA
2	91350001	91400000	0.472062	2	91230738	91358164	127427	-	ENSSSCG00000048661	NA	lncRNA
2	18730001	18780000	0.363579	2	18726031	18736920	10890	+	ENSSSCG00000048253	NA	lncRNA
2	77450001	77500000	0.380724	2	77490589	77490695	107	-	ENSSSCG00000028716	U6	snRNA
2	72780001	72830000	0.394494	2	72803981	72804092	112	-	ENSSSCG00000021577	U6	snRNA
2	73200001	73250000	0.43344	2	73248723	73248829	107	-	ENSSSCG00000034467	U6	snRNA
2	73210001	73260000	0.415311	2	73248723	73248829	107	-	ENSSSCG00000034467	U6	snRNA
2	73220001	73270000	0.429233	2	73248723	73248829	107	-	ENSSSCG00000034467	U6	snRNA

2	73230001	73280000	0.39343	2	73248723	73248829	107	-	ENSSSCG00000034467	U6	snRNA
2	73240001	73290000	0.385066	2	73248723	73248829	107	-	ENSSSCG00000034467	U6	snRNA
2	75440001	75490000	0.353754	2	75437201	75457092	19892	-	ENSSSCG00000029279	GNA11	protein_coding
2	75450001	75500000	0.363226	2	75437201	75457092	19892	-	ENSSSCG00000029279	GNA11	protein_coding
2	75450001	75500000	0.363226	2	75492207	75501543	9337	+	ENSSSCG00000032265	TLE5	protein_coding
2	56360001	56410000	0.442	2	56402657	56403580	924	+	ENSSSCG00000023700	OR13G1	protein_coding
2	56370001	56420000	0.432098	2	56402657	56403580	924	+	ENSSSCG00000023700	OR13G1	protein_coding
2	56380001	56430000	0.442975	2	56402657	56403580	924	+	ENSSSCG00000023700	OR13G1	protein_coding
2	56390001	56440000	0.443633	2	56402657	56403580	924	+	ENSSSCG00000023700	OR13G1	protein_coding
2	56400001	56450000	0.437399	2	56402657	56403580	924	+	ENSSSCG00000023700	OR13G1	protein_coding
2	56760001	56810000	0.352722	2	56760601	56769516	8916	-	ENSSSCG00000049431	NA	protein_coding
2	56760001	56810000	0.352722	2	56760602	56769511	8910	+	ENSSSCG00000041930	OR2C3	protein_coding
2	56780001	56830000	0.366259	2	56813239	56818351	5113	-	ENSSSCG00000013942	NA	protein_coding
2	56800001	56850000	0.36456	2	56813239	56818351	5113	-	ENSSSCG00000013942	NA	protein_coding
2	56810001	56860000	0.364372	2	56813239	56818351	5113	-	ENSSSCG00000013942	NA	protein_coding
2	56780001	56830000	0.366259	2	56826890	56829562	2673	+	ENSSSCG00000045107	NA	protein_coding
2	56800001	56850000	0.36456	2	56826890	56829562	2673	+	ENSSSCG00000045107	NA	protein_coding
2	56810001	56860000	0.364372	2	56826890	56829562	2673	+	ENSSSCG00000045107	NA	protein_coding
2	56820001	56870000	0.376104	2	56826890	56829562	2673	+	ENSSSCG00000045107	NA	protein_coding
2	56820001	56870000	0.376104	2	56862025	56862978	954	+	ENSSSCG00000013941	OR2B11	protein_coding
2	56830001	56880000	0.370145	2	56862025	56862978	954	+	ENSSSCG00000013941	OR2B11	protein_coding
2	56840001	56890000	0.354202	2	56862025	56862978	954	+	ENSSSCG00000013941	OR2B11	protein_coding
2	142260001	142310000	0.371357	2	142166167	142306102	139936	+	ENSSSCG00000014364	ANKHD1	protein_coding
2	142270001	142320000	0.406065	2	142166167	142306102	139936	+	ENSSSCG00000014364	ANKHD1	protein_coding
2	142260001	142310000	0.371357	2	142307025	142323475	16451	-	ENSSSCG00000014366	APBB3	protein_coding
2	142270001	142320000	0.406065	2	142307025	142323475	16451	-	ENSSSCG00000014366	APBB3	protein_coding
2	77210001	77260000	0.35376	2	77203991	77211870	7880	-	ENSSSCG00000013432	MIDN	protein_coding
2	77210001	77260000	0.35376	2	77216433	77222749	6317	-	ENSSSCG00000035532	ATP5F1D	protein_coding
2	77220001	77270000	0.367497	2	77216433	77222749	6317	-	ENSSSCG00000035532	ATP5F1D	protein_coding
2	77210001	77260000	0.35376	2	77221171	77230056	8886	+	ENSSSCG00000034339	CBARP	protein_coding
2	77220001	77270000	0.367497	2	77221171	77230056	8886	+	ENSSSCG00000034339	CBARP	protein_coding
2	77210001	77260000	0.35376	2	77230372	77249327	18956	-	ENSSSCG00000013429	STK11	protein_coding
2	77220001	77270000	0.367497	2	77230372	77249327	18956	-	ENSSSCG00000013429	STK11	protein_coding
2	77430001	77480000	0.430982	2	77431064	77464628	33565	-	ENSSSCG00000037642	ARID3A	protein_coding
2	77440001	77490000	0.432355	2	77431064	77464628	33565	-	ENSSSCG00000037642	ARID3A	protein_coding
2	77450001	77500000	0.380724	2	77431064	77464628	33565	-	ENSSSCG00000037642	ARID3A	protein_coding
2	77430001	77480000	0.430982	2	77472362	77479204	6843	-	ENSSSCG00000013420	KISS1R	protein_coding
2	77440001	77490000	0.432355	2	77472362	77479204	6843	-	ENSSSCG00000013420	KISS1R	protein_coding
2	77450001	77500000	0.380724	2	77472362	77479204	6843	-	ENSSSCG00000013420	KISS1R	protein_coding
2	77430001	77480000	0.430982	2	77477881	77489105	11225	+	ENSSSCG00000013419	R3HDM4	protein_coding
2	77440001	77490000	0.432355	2	77477881	77489105	11225	+	ENSSSCG00000013419	R3HDM4	protein_coding
2	77450001	77500000	0.380724	2	77477881	77489105	11225	+	ENSSSCG00000013419	R3HDM4	protein_coding
2	77450001	77500000	0.380724	2	77490996	77527659	36664	+	ENSSSCG00000013416	MED16	protein_coding
2	54450001	54500000	0.353563	2	54460667	54461630	964	+	ENSSSCG00000033545	NA	protein_coding
2	54450001	54500000	0.353563	2	54480177	54481184	1008	+	ENSSSCG00000033666	NA	pseudogene

2	55240001	55290000	0.375925	2	55261053	55262083	1031	-	ENSSSCG00000036934	NA	protein_coding
2	55250001	55300000	0.432272	2	55261053	55262083	1031	-	ENSSSCG00000036934	NA	protein_coding
2	55260001	55310000	0.446157	2	55261053	55262083	1031	-	ENSSSCG00000036934	NA	protein_coding
2	55290001	55340000	0.465659	2	55332201	55334066	1866	-	ENSSSCG00000041730	NA	protein_coding
2	55300001	55350000	0.452874	2	55332201	55334066	1866	-	ENSSSCG00000041730	NA	protein_coding
2	55310001	55360000	0.455718	2	55332201	55334066	1866	-	ENSSSCG00000041730	NA	protein_coding
2	55320001	55370000	0.450889	2	55332201	55334066	1866	-	ENSSSCG00000041730	NA	protein_coding
2	55330001	55380000	0.437558	2	55332201	55334066	1866	-	ENSSSCG00000041730	NA	protein_coding
2	77690001	77740000	0.360068	2	77694234	77711630	17397	+	ENSSSCG00000035693	POLRMT	protein_coding
2	77690001	77740000	0.360068	2	77708161	77730881	22721	-	ENSSSCG00000033442	HCN2	protein_coding
2	77720001	77770000	0.352596	2	77708161	77730881	22721	-	ENSSSCG00000033442	HCN2	protein_coding
2	77730001	77780000	0.356334	2	77708161	77730881	22721	-	ENSSSCG00000033442	HCN2	protein_coding
2	77690001	77740000	0.360068	2	77735584	77743403	7820	-	ENSSSCG00000027907	NA	protein_coding
2	77720001	77770000	0.352596	2	77735584	77743403	7820	-	ENSSSCG00000027907	NA	protein_coding
2	77730001	77780000	0.356334	2	77735584	77743403	7820	-	ENSSSCG00000027907	NA	protein_coding
2	77740001	77790000	0.377319	2	77735584	77743403	7820	-	ENSSSCG00000027907	NA	protein_coding
2	77720001	77770000	0.352596	2	77753813	77759983	6171	-	ENSSSCG00000032810	NA	protein_coding
2	77730001	77780000	0.356334	2	77753813	77759983	6171	-	ENSSSCG00000032810	NA	protein_coding
2	77740001	77790000	0.377319	2	77753813	77759983	6171	-	ENSSSCG00000032810	NA	protein_coding
2	77750001	77800000	0.360631	2	77753813	77759983	6171	-	ENSSSCG00000032810	NA	protein_coding
2	77720001	77770000	0.352596	2	77761590	77768396	6807	-	ENSSSCG00000039640	CDC34	protein_coding
2	77730001	77780000	0.356334	2	77761590	77768396	6807	-	ENSSSCG00000039640	CDC34	protein_coding
2	77740001	77790000	0.377319	2	77761590	77768396	6807	-	ENSSSCG00000039640	CDC34	protein_coding
2	77750001	77800000	0.360631	2	77761590	77768396	6807	-	ENSSSCG00000039640	CDC34	protein_coding
2	77720001	77770000	0.352596	2	77764858	77806762	41905	+	ENSSSCG00000036640	NA	protein_coding
2	77730001	77780000	0.356334	2	77764858	77806762	41905	+	ENSSSCG00000036640	NA	protein_coding
2	77740001	77790000	0.377319	2	77764858	77806762	41905	+	ENSSSCG00000036640	NA	protein_coding
2	77750001	77800000	0.360631	2	77764858	77806762	41905	+	ENSSSCG00000036640	NA	protein_coding
2	77730001	77780000	0.356334	2	77775303	77783424	8122	-	ENSSSCG00000036434	TPGS1	protein_coding
2	77740001	77790000	0.377319	2	77775303	77783424	8122	-	ENSSSCG00000036434	TPGS1	protein_coding
2	77750001	77800000	0.360631	2	77775303	77783424	8122	-	ENSSSCG00000036434	TPGS1	protein_coding
2	77740001	77790000	0.377319	2	77784610	77789068	4459	-	ENSSSCG00000039500	NA	protein_coding
2	77750001	77800000	0.360631	2	77784610	77789068	4459	-	ENSSSCG00000039500	NA	protein_coding
2	56210001	56260000	0.493941	2	56251339	56253154	1816	+	ENSSSCG00000043398	NA	protein_coding
2	56220001	56270000	0.482776	2	56251339	56253154	1816	+	ENSSSCG00000043398	NA	protein_coding
2	56230001	56280000	0.473042	2	56251339	56253154	1816	+	ENSSSCG00000043398	NA	protein_coding
2	56240001	56290000	0.469207	2	56251339	56253154	1816	+	ENSSSCG00000043398	NA	protein_coding
2	56250001	56300000	0.463122	2	56251339	56253154	1816	+	ENSSSCG00000043398	NA	protein_coding
2	56890001	56940000	0.384071	2	56892242	56977234	84993	-	ENSSSCG00000013940	NLRP3	protein_coding
2	56900001	56950000	0.399311	2	56892242	56977234	84993	-	ENSSSCG00000013940	NLRP3	protein_coding
2	56910001	56960000	0.40263	2	56892242	56977234	84993	-	ENSSSCG00000013940	NLRP3	protein_coding
2	56920001	56970000	0.408948	2	56892242	56977234	84993	-	ENSSSCG00000013940	NLRP3	protein_coding
2	56930001	56980000	0.385848	2	56892242	56977234	84993	-	ENSSSCG00000013940	NLRP3	protein_coding
2	56900001	56950000	0.399311	2	56945400	56946969	1570	+	ENSSSCG00000036878	NA	protein_coding
2	56910001	56960000	0.40263	2	56945400	56946969	1570	+	ENSSSCG00000036878	NA	protein_coding

2	56920001	56970000	0.408948	2	56945400	56946969	1570	+	ENSSSCG00000036878	NA	protein_coding
2	56930001	56980000	0.385848	2	56945400	56946969	1570	+	ENSSSCG00000036878	NA	protein_coding
2	57030001	57080000	0.372702	2	57053652	57097828	44177	+	ENSSSCG00000036756	ZNF496	protein_coding
2	57040001	57090000	0.384229	2	57053652	57097828	44177	+	ENSSSCG00000036756	ZNF496	protein_coding
2	57060001	57110000	0.361174	2	57053652	57097828	44177	+	ENSSSCG00000036756	ZNF496	protein_coding
2	57070001	57120000	0.382069	2	57053652	57097828	44177	+	ENSSSCG00000036756	ZNF496	protein_coding
2	57080001	57130000	0.391358	2	57053652	57097828	44177	+	ENSSSCG00000036756	ZNF496	protein_coding
2	57090001	57140000	0.400807	2	57053652	57097828	44177	+	ENSSSCG00000036756	ZNF496	protein_coding
2	147110001	147160000	0.365342	2	147109581	147191638	82058	-	ENSSSCG00000014406	PRELID2	protein_coding
2	147120001	147170000	0.365385	2	147109581	147191638	82058	-	ENSSSCG00000014406	PRELID2	protein_coding
2	147110001	147160000	0.365342	2	147157908	147166391	8484	-	ENSSSCG00000041978	NA	protein_coding
2	147120001	147170000	0.365385	2	147157908	147166391	8484	-	ENSSSCG00000041978	NA	protein_coding
2	57150001	57200000	0.410057	2	57194217	57196091	1875	-	ENSSSCG00000044749	NA	protein_coding
2	57160001	57210000	0.387122	2	57194217	57196091	1875	-	ENSSSCG00000044749	NA	protein_coding
2	57170001	57220000	0.429689	2	57194217	57196091	1875	-	ENSSSCG00000044749	NA	protein_coding
2	57180001	57230000	0.422076	2	57194217	57196091	1875	-	ENSSSCG00000044749	NA	protein_coding
2	57190001	57240000	0.425099	2	57194217	57196091	1875	-	ENSSSCG00000044749	NA	protein_coding
2	57160001	57210000	0.387122	2	57205282	57211999	6718	+	ENSSSCG00000043575	NA	pseudogene
2	57170001	57220000	0.429689	2	57205282	57211999	6718	+	ENSSSCG00000043575	NA	pseudogene
2	57180001	57230000	0.422076	2	57205282	57211999	6718	+	ENSSSCG00000043575	NA	pseudogene
2	57190001	57240000	0.425099	2	57205282	57211999	6718	+	ENSSSCG00000043575	NA	pseudogene
2	57200001	57250000	0.40269	2	57205282	57211999	6718	+	ENSSSCG00000043575	NA	pseudogene
2	57210001	57260000	0.393931	2	57205282	57211999	6718	+	ENSSSCG00000043575	NA	pseudogene
2	57180001	57230000	0.422076	2	57226972	57271406	44435	+	ENSSSCG00000035650	NA	protein_coding
2	57190001	57240000	0.425099	2	57226972	57271406	44435	+	ENSSSCG00000035650	NA	protein_coding
2	57200001	57250000	0.40269	2	57226972	57271406	44435	+	ENSSSCG00000035650	NA	protein_coding
2	57210001	57260000	0.393931	2	57226972	57271406	44435	+	ENSSSCG00000035650	NA	protein_coding
2	99820001	99870000	0.355269	2	99863453	99867540	4088	+	ENSSSCG00000043879	NA	protein_coding
2	57990001	58040000	0.356062	2	57999464	58002759	3296	+	ENSSSCG00000050314	NA	pseudogene
2	58000001	58050000	0.368139	2	57999464	58002759	3296	+	ENSSSCG00000050314	NA	pseudogene
2	57130001	57180000	0.403959	2	57177243	57178190	948	+	ENSSSCG00000036813	NA	protein_coding
2	57140001	57190000	0.40155	2	57177243	57178190	948	+	ENSSSCG00000036813	NA	protein_coding
2	57150001	57200000	0.410057	2	57177243	57178190	948	+	ENSSSCG00000036813	NA	protein_coding
2	57160001	57210000	0.387122	2	57177243	57178190	948	+	ENSSSCG00000036813	NA	protein_coding
2	57170001	57220000	0.429689	2	57177243	57178190	948	+	ENSSSCG00000036813	NA	protein_coding
2	55410001	55460000	0.453299	2	55453173	55454108	936	-	ENSSSCG00000034862	NA	protein_coding
2	55420001	55470000	0.458969	2	55453173	55454108	936	-	ENSSSCG00000034862	NA	protein_coding
2	55430001	55480000	0.411824	2	55453173	55454108	936	-	ENSSSCG00000034862	NA	protein_coding
2	55440001	55490000	0.381863	2	55453173	55454108	936	-	ENSSSCG00000034862	NA	protein_coding
2	58950001	59000000	0.37125	2	58930990	58972977	41988	+	ENSSSCG00000027840	NA	protein_coding
2	58960001	59010000	0.364232	2	58930990	58972977	41988	+	ENSSSCG00000027840	NA	protein_coding
2	58970001	59020000	0.379177	2	58930990	58972977	41988	+	ENSSSCG00000027840	NA	protein_coding
2	58950001	59000000	0.37125	2	58951726	58974672	22947	+	ENSSSCG00000013928	GDF1	protein_coding
2	58960001	59010000	0.364232	2	58951726	58974672	22947	+	ENSSSCG00000013928	GDF1	protein_coding
2	58970001	59020000	0.379177	2	58951726	58974672	22947	+	ENSSSCG00000013928	GDF1	protein_coding

2	58950001	59000000	0.37125	2	58975015	59013495	38481	-	ENSSSCG00000039301	UPF1	protein_coding
2	58960001	59010000	0.364232	2	58975015	59013495	38481	-	ENSSSCG00000039301	UPF1	protein_coding
2	58970001	59020000	0.379177	2	58975015	59013495	38481	-	ENSSSCG00000039301	UPF1	protein_coding
2	58980001	59030000	0.352561	2	58975015	59013495	38481	-	ENSSSCG00000039301	UPF1	protein_coding
2	59470001	59520000	0.394342	2	59414008	59485857	71850	-	ENSSSCG00000039684	PGPEP1	protein_coding
2	59480001	59530000	0.432983	2	59414008	59485857	71850	-	ENSSSCG00000039684	PGPEP1	protein_coding
2	59470001	59520000	0.394342	2	59467146	59472777	5632	+	ENSSSCG00000042600	NA	pseudogene
2	59470001	59520000	0.394342	2	59498804	59517145	18342	+	ENSSSCG00000034314	NA	protein_coding
2	59480001	59530000	0.432983	2	59498804	59517145	18342	+	ENSSSCG00000034314	NA	protein_coding
2	59490001	59540000	0.464268	2	59498804	59517145	18342	+	ENSSSCG00000034314	NA	protein_coding
2	59500001	59550000	0.474683	2	59498804	59517145	18342	+	ENSSSCG00000034314	NA	protein_coding
2	59490001	59540000	0.464268	2	59531154	59532196	1043	+	ENSSSCG00000031657	NA	pseudogene
2	59500001	59550000	0.474683	2	59531154	59532196	1043	+	ENSSSCG00000031657	NA	pseudogene
2	59500001	59550000	0.474683	2	59543984	59558456	14473	+	ENSSSCG00000038331	NA	protein_coding
2	59780001	59830000	0.35811	2	59780011	59817059	37049	-	ENSSSCG00000013892	KCNN1	protein_coding
2	59790001	59840000	0.376995	2	59780011	59817059	37049	-	ENSSSCG00000013892	KCNN1	protein_coding
2	59800001	59850000	0.387278	2	59780011	59817059	37049	-	ENSSSCG00000013892	KCNN1	protein_coding
2	59810001	59860000	0.391482	2	59780011	59817059	37049	-	ENSSSCG00000013892	KCNN1	protein_coding
2	59780001	59830000	0.35811	2	59819111	59831482	12372	-	ENSSSCG00000013891	CCDC124	protein_coding
2	59790001	59840000	0.376995	2	59819111	59831482	12372	-	ENSSSCG00000013891	CCDC124	protein_coding
2	59800001	59850000	0.387278	2	59819111	59831482	12372	-	ENSSSCG00000013891	CCDC124	protein_coding
2	59810001	59860000	0.391482	2	59819111	59831482	12372	-	ENSSSCG00000013891	CCDC124	protein_coding
2	59820001	59870000	0.440699	2	59819111	59831482	12372	-	ENSSSCG00000013891	CCDC124	protein_coding
2	59830001	59880000	0.409091	2	59819111	59831482	12372	-	ENSSSCG00000013891	CCDC124	protein_coding
2	59820001	59870000	0.440699	2	59869154	59885265	16112	-	ENSSSCG00000013890	SLC5A5	protein_coding
2	59830001	59880000	0.409091	2	59869154	59885265	16112	-	ENSSSCG00000013890	SLC5A5	protein_coding
2	59840001	59890000	0.408021	2	59869154	59885265	16112	-	ENSSSCG00000013890	SLC5A5	protein_coding
2	59850001	59900000	0.357091	2	59869154	59885265	16112	-	ENSSSCG00000013890	SLC5A5	protein_coding
2	59850001	59900000	0.357091	2	59890796	59897024	6229	-	ENSSSCG00000013889	NA	protein_coding
2	55240001	55290000	0.375925	2	55277835	55278773	939	-	ENSSSCG00000050475	NA	protein_coding
2	55250001	55300000	0.432272	2	55277835	55278773	939	-	ENSSSCG00000050475	NA	protein_coding
2	55260001	55310000	0.446157	2	55277835	55278773	939	-	ENSSSCG00000050475	NA	protein_coding
2	55270001	55320000	0.46367	2	55277835	55278773	939	-	ENSSSCG00000050475	NA	protein_coding
2	56110001	56160000	0.367435	2	56149329	56150261	933	-	ENSSSCG00000013949	NA	protein_coding
2	56120001	56170000	0.422602	2	56149329	56150261	933	-	ENSSSCG00000013949	NA	protein_coding
2	56130001	56180000	0.463413	2	56149329	56150261	933	-	ENSSSCG00000013949	NA	protein_coding
2	56140001	56190000	0.443077	2	56149329	56150261	933	-	ENSSSCG00000013949	NA	protein_coding
2	56150001	56200000	0.429946	2	56149329	56150261	933	-	ENSSSCG00000013949	NA	protein_coding
2	56260001	56310000	0.444726	2	56301803	56302714	912	+	ENSSSCG00000013951	NA	protein_coding
2	56270001	56320000	0.470141	2	56301803	56302714	912	+	ENSSSCG00000013951	NA	protein_coding
2	56280001	56330000	0.470604	2	56301803	56302714	912	+	ENSSSCG00000013951	NA	protein_coding
2	56290001	56340000	0.473332	2	56301803	56302714	912	+	ENSSSCG00000013951	NA	protein_coding
2	56300001	56350000	0.466136	2	56301803	56302714	912	+	ENSSSCG00000013951	NA	protein_coding
2	56440001	56490000	0.434495	2	56486132	56487073	942	-	ENSSSCG00000036591	NA	protein_coding
2	56450001	56500000	0.413851	2	56486132	56487073	942	-	ENSSSCG00000036591	NA	protein_coding

2	56460001	56510000	0.369104	2	56486132	56487073	942	-	ENSSSCG00000036591	NA	protein_coding
2	55340001	55390000	0.431072	2	55380759	55381697	939	-	ENSSSCG00000046662	NA	protein_coding
2	55350001	55400000	0.453446	2	55380759	55381697	939	-	ENSSSCG00000046662	NA	protein_coding
2	55360001	55410000	0.456809	2	55380759	55381697	939	-	ENSSSCG00000046662	NA	protein_coding
2	55370001	55420000	0.45434	2	55380759	55381697	939	-	ENSSSCG00000046662	NA	protein_coding
2	55380001	55430000	0.467666	2	55380759	55381697	939	-	ENSSSCG00000046662	NA	protein_coding
2	63210001	63260000	0.372622	2	63258938	63259987	1050	+	ENSSSCG00000037040	NA	protein_coding
2	57990001	58040000	0.356062	2	58035772	58041631	5860	+	ENSSSCG00000043984	NA	protein_coding
2	58000001	58050000	0.368139	2	58035772	58041631	5860	+	ENSSSCG00000043984	NA	protein_coding
2	56270001	56320000	0.470141	2	56314658	56315587	930	-	ENSSSCG00000036683	OR14K1	protein_coding
2	56280001	56330000	0.470604	2	56314658	56315587	930	-	ENSSSCG00000036683	OR14K1	protein_coding
2	56290001	56340000	0.473332	2	56314658	56315587	930	-	ENSSSCG00000036683	OR14K1	protein_coding
2	56300001	56350000	0.466136	2	56314658	56315587	930	-	ENSSSCG00000036683	OR14K1	protein_coding
2	56310001	56360000	0.456146	2	56314658	56315587	930	-	ENSSSCG00000036683	OR14K1	protein_coding
2	55430001	55480000	0.411824	2	55471670	55472602	933	-	ENSSSCG00000045240	NA	protein_coding
2	55440001	55490000	0.381863	2	55471670	55472602	933	-	ENSSSCG00000045240	NA	protein_coding
2	55260001	55310000	0.446157	2	55309592	55310530	939	-	ENSSSCG00000046179	NA	protein_coding
2	55270001	55320000	0.46367	2	55309592	55310530	939	-	ENSSSCG00000046179	NA	protein_coding
2	55280001	55330000	0.467466	2	55309592	55310530	939	-	ENSSSCG00000046179	NA	protein_coding
2	55290001	55340000	0.465659	2	55309592	55310530	939	-	ENSSSCG00000046179	NA	protein_coding
2	55300001	55350000	0.452874	2	55309592	55310530	939	-	ENSSSCG00000046179	NA	protein_coding
2	55310001	55360000	0.455718	2	55309592	55310530	939	-	ENSSSCG00000046179	NA	protein_coding
2	72480001	72530000	0.357451	2	72500149	72500463	315	-	ENSSSCG00000024275	NA	protein_coding
2	72500001	72550000	0.35829	2	72500149	72500463	315	-	ENSSSCG00000024275	NA	protein_coding
2	65520001	65570000	0.369606	2	65561253	65566129	4877	-	ENSSSCG00000051044	NA	protein_coding
2	63170001	63220000	0.397652	2	63154774	63194328	39555	-	ENSSSCG00000013785	NA	protein_coding
2	63180001	63230000	0.420103	2	63154774	63194328	39555	-	ENSSSCG00000013785	NA	protein_coding
2	63190001	63240000	0.439643	2	63154774	63194328	39555	-	ENSSSCG00000013785	NA	protein_coding
2	18760001	18810000	0.465052	2	18807523	18954650	147128	-	ENSSSCG00000013284	TTC17	protein_coding
2	18770001	18820000	0.454707	2	18807523	18954650	147128	-	ENSSSCG00000013284	TTC17	protein_coding
2	18780001	18830000	0.4488	2	18807523	18954650	147128	-	ENSSSCG00000013284	TTC17	protein_coding
2	18790001	18840000	0.412586	2	18807523	18954650	147128	-	ENSSSCG00000013284	TTC17	protein_coding
2	18800001	18850000	0.401725	2	18807523	18954650	147128	-	ENSSSCG00000013284	TTC17	protein_coding
2	18860001	18910000	0.375068	2	18807523	18954650	147128	-	ENSSSCG00000013284	TTC17	protein_coding
2	18870001	18920000	0.363529	2	18807523	18954650	147128	-	ENSSSCG00000013284	TTC17	protein_coding
2	90430001	90480000	0.35471	2	90436636	90666196	229561	+	ENSSSCG00000025210	ATG10	protein_coding
2	90430001	90480000	0.35471	2	90437033	90739506	302474	+	ENSSSCG00000014132	NA	protein_coding
2	91300001	91350000	0.487639	2	91347153	91351386	4234	+	ENSSSCG00000045510	NA	pseudogene
2	91310001	91360000	0.517895	2	91347153	91351386	4234	+	ENSSSCG00000045510	NA	pseudogene
2	91320001	91370000	0.537275	2	91347153	91351386	4234	+	ENSSSCG00000045510	NA	pseudogene
2	91330001	91380000	0.551391	2	91347153	91351386	4234	+	ENSSSCG00000045510	NA	pseudogene
2	91340001	91390000	0.515854	2	91347153	91351386	4234	+	ENSSSCG00000045510	NA	pseudogene
2	91350001	91400000	0.472062	2	91347153	91351386	4234	+	ENSSSCG00000045510	NA	pseudogene
2	91310001	91360000	0.517895	2	91358399	91811208	452810	+	ENSSSCG00000014136	VCAN	protein_coding
2	91320001	91370000	0.537275	2	91358399	91811208	452810	+	ENSSSCG00000014136	VCAN	protein_coding

2	91330001	91380000	0.551391	2	91358399	91811208	452810	+	ENSSSCG00000014136	VCAN	protein_coding
2	91340001	91390000	0.515854	2	91358399	91811208	452810	+	ENSSSCG00000014136	VCAN	protein_coding
2	91350001	91400000	0.472062	2	91358399	91811208	452810	+	ENSSSCG00000014136	VCAN	protein_coding
2	91360001	91410000	0.432582	2	91358399	91811208	452810	+	ENSSSCG00000014136	VCAN	protein_coding
2	91370001	91420000	0.403906	2	91358399	91811208	452810	+	ENSSSCG00000014136	VCAN	protein_coding
2	91380001	91430000	0.415772	2	91358399	91811208	452810	+	ENSSSCG00000014136	VCAN	protein_coding
2	91390001	91440000	0.459337	2	91358399	91811208	452810	+	ENSSSCG00000014136	VCAN	protein_coding
2	91400001	91450000	0.471502	2	91358399	91811208	452810	+	ENSSSCG00000014136	VCAN	protein_coding
2	91410001	91460000	0.483371	2	91358399	91811208	452810	+	ENSSSCG00000014136	VCAN	protein_coding
2	91420001	91470000	0.464406	2	91358399	91811208	452810	+	ENSSSCG00000014136	VCAN	protein_coding
2	91430001	91480000	0.391105	2	91358399	91811208	452810	+	ENSSSCG00000014136	VCAN	protein_coding
2	92290001	92340000	0.361183	2	92123345	92554262	430918	-	ENSSSCG00000026780	EDIL3	protein_coding
2	64780001	64830000	0.356391	2	64780442	64798783	18342	+	ENSSSCG00000032842	GIPC1	protein_coding
2	64780001	64830000	0.356391	2	64797220	64800459	3240	+	ENSSSCG00000013782	PTGER1	protein_coding
2	64780001	64830000	0.356391	2	64800720	64824957	24238	-	ENSSSCG00000013779	PKN1	protein_coding
2	64810001	64860000	0.393344	2	64800720	64824957	24238	-	ENSSSCG00000013779	PKN1	protein_coding
2	64820001	64870000	0.448728	2	64800720	64824957	24238	-	ENSSSCG00000013779	PKN1	protein_coding
2	64810001	64860000	0.393344	2	64836062	64844994	8933	+	ENSSSCG00000013776	DDX39A	protein_coding
2	64820001	64870000	0.448728	2	64836062	64844994	8933	+	ENSSSCG00000013776	DDX39A	protein_coding
2	64830001	64880000	0.442272	2	64836062	64844994	8933	+	ENSSSCG00000013776	DDX39A	protein_coding
2	64840001	64890000	0.427482	2	64836062	64844994	8933	+	ENSSSCG00000013776	DDX39A	protein_coding
2	64810001	64860000	0.393344	2	64844792	64890939	46148	-	ENSSSCG00000013775	ADGRE5	protein_coding
2	64820001	64870000	0.448728	2	64844792	64890939	46148	-	ENSSSCG00000013775	ADGRE5	protein_coding
2	64830001	64880000	0.442272	2	64844792	64890939	46148	-	ENSSSCG00000013775	ADGRE5	protein_coding
2	64840001	64890000	0.427482	2	64844792	64890939	46148	-	ENSSSCG00000013775	ADGRE5	protein_coding
2	64850001	64900000	0.418934	2	64844792	64890939	46148	-	ENSSSCG00000013775	ADGRE5	protein_coding
2	64860001	64910000	0.374164	2	64844792	64890939	46148	-	ENSSSCG00000013775	ADGRE5	protein_coding
2	64950001	65000000	0.371071	2	64997159	65044331	47173	+	ENSSSCG00000013773	ADGRL1	protein_coding
2	64960001	65010000	0.377566	2	64997159	65044331	47173	+	ENSSSCG00000013773	ADGRL1	protein_coding
2	64970001	65020000	0.361119	2	64997159	65044331	47173	+	ENSSSCG00000013773	ADGRL1	protein_coding
2	98980001	99030000	0.38476	2	99013669	99014978	1310	+	ENSSSCG00000046245	NA	protein_coding
2	98990001	99040000	0.452278	2	99013669	99014978	1310	+	ENSSSCG00000046245	NA	protein_coding
2	99000001	99050000	0.461111	2	99013669	99014978	1310	+	ENSSSCG00000046245	NA	protein_coding
2	99010001	99060000	0.433994	2	99013669	99014978	1310	+	ENSSSCG00000046245	NA	protein_coding
2	29650001	29700000	0.365174	2	29365195	29792983	427789	+	ENSSSCG00000013326	DCDC1	protein_coding
2	29660001	29710000	0.372957	2	29365195	29792983	427789	+	ENSSSCG00000013326	DCDC1	protein_coding
2	29670001	29720000	0.367534	2	29365195	29792983	427789	+	ENSSSCG00000013326	DCDC1	protein_coding
2	66430001	66480000	0.356422	2	66396668	66466187	69520	-	ENSSSCG00000029347	NA	protein_coding
2	66430001	66480000	0.356422	2	66445267	66445719	453	+	ENSSSCG00000046939	NA	protein_coding
2	66430001	66480000	0.356422	2	66449010	66499957	50948	-	ENSSSCG00000033388	NA	protein_coding
2	66430001	66480000	0.356422	2	66478900	66513477	34578	-	ENSSSCG00000022634	NA	protein_coding
2	124190001	124240000	0.389128	2	124199508	124463887	264380	+	ENSSSCG00000039760	PRR16	protein_coding
2	124210001	124260000	0.361827	2	124199508	124463887	264380	+	ENSSSCG00000039760	PRR16	protein_coding
2	124790001	124840000	0.381051	2	124720326	125153557	433232	+	ENSSSCG00000041876	NA	protein_coding
2	124810001	124860000	0.363048	2	124720326	125153557	433232	+	ENSSSCG00000041876	NA	protein_coding

2	126370001	126420000	0.353154	2	126349973	126552304	202332	+	ENSSSCG00000039373	SNX24	protein_coding
2	127060001	127110000	0.356059	2	127017005	127180627	163623	+	ENSSSCG00000014240	CSNK1G3	protein_coding
2	127160001	127210000	0.377063	2	127017005	127180627	163623	+	ENSSSCG00000014240	CSNK1G3	protein_coding
2	127170001	127220000	0.392264	2	127017005	127180627	163623	+	ENSSSCG00000014240	CSNK1G3	protein_coding
2	127180001	127230000	0.391539	2	127017005	127180627	163623	+	ENSSSCG00000014240	CSNK1G3	protein_coding
2	70990001	71040000	0.354368	2	71004417	71048250	43834	-	ENSSSCG00000013595	CERS4	protein_coding
2	71000001	71050000	0.388441	2	71004417	71048250	43834	-	ENSSSCG00000013595	CERS4	protein_coding
2	71020001	71070000	0.355644	2	71004417	71048250	43834	-	ENSSSCG00000013595	CERS4	protein_coding
2	71630001	71680000	0.361408	2	71624266	71631850	7585	-	ENSSSCG00000013571	TEX45	protein_coding
2	71630001	71680000	0.361408	2	71635487	71652903	17417	+	ENSSSCG00000013569	PEX11G	protein_coding
2	71640001	71690000	0.375545	2	71635487	71652903	17417	+	ENSSSCG00000013569	PEX11G	protein_coding
2	71650001	71700000	0.364076	2	71635487	71652903	17417	+	ENSSSCG00000013569	PEX11G	protein_coding
2	71630001	71680000	0.361408	2	71651100	71747410	96311	-	ENSSSCG00000013568	ARHGEF18	protein_coding
2	71640001	71690000	0.375545	2	71651100	71747410	96311	-	ENSSSCG00000013568	ARHGEF18	protein_coding
2	71650001	71700000	0.364076	2	71651100	71747410	96311	-	ENSSSCG00000013568	ARHGEF18	protein_coding
2	71670001	71720000	0.366492	2	71651100	71747410	96311	-	ENSSSCG00000013568	ARHGEF18	protein_coding
2	71730001	71780000	0.354154	2	71651100	71747410	96311	-	ENSSSCG00000013568	ARHGEF18	protein_coding
2	71740001	71790000	0.414482	2	71651100	71747410	96311	-	ENSSSCG00000013568	ARHGEF18	protein_coding
2	71750001	71800000	0.47283	2	71797204	71936139	138936	+	ENSSSCG00000013566	INSR	protein_coding
2	71760001	71810000	0.4777	2	71797204	71936139	138936	+	ENSSSCG00000013566	INSR	protein_coding
2	71770001	71820000	0.447072	2	71797204	71936139	138936	+	ENSSSCG00000013566	INSR	protein_coding
2	71780001	71830000	0.44424	2	71797204	71936139	138936	+	ENSSSCG00000013566	INSR	protein_coding
2	71790001	71840000	0.407985	2	71797204	71936139	138936	+	ENSSSCG00000013566	INSR	protein_coding
2	71800001	71850000	0.390749	2	71797204	71936139	138936	+	ENSSSCG00000013566	INSR	protein_coding
2	71810001	71860000	0.387204	2	71797204	71936139	138936	+	ENSSSCG00000013566	INSR	protein_coding
2	71820001	71870000	0.366557	2	71797204	71936139	138936	+	ENSSSCG00000013566	INSR	protein_coding
2	71830001	71880000	0.367839	2	71797204	71936139	138936	+	ENSSSCG00000013566	INSR	protein_coding
2	71840001	71890000	0.381869	2	71797204	71936139	138936	+	ENSSSCG00000013566	INSR	protein_coding
2	71850001	71900000	0.37847	2	71797204	71936139	138936	+	ENSSSCG00000013566	INSR	protein_coding
2	71860001	71910000	0.369142	2	71797204	71936139	138936	+	ENSSSCG00000013566	INSR	protein_coding
2	71870001	71920000	0.373033	2	71797204	71936139	138936	+	ENSSSCG00000013566	INSR	protein_coding
2	71930001	71980000	0.378884	2	71797204	71936139	138936	+	ENSSSCG00000013566	INSR	protein_coding
2	71930001	71980000	0.378884	2	71957473	72002371	44899	+	ENSSSCG00000033635	NA	protein_coding
2	71930001	71980000	0.378884	2	71963816	71980296	16481	-	ENSSSCG00000041386	NA	protein_coding
2	71930001	71980000	0.378884	2	71966568	72013697	47130	+	ENSSSCG00000013559	NA	protein_coding
2	72100001	72150000	0.381963	2	72073414	72112948	39535	-	ENSSSCG00000026402	NA	protein_coding
2	72100001	72150000	0.381963	2	72142732	72146550	3819	-	ENSSSCG00000050480	NA	protein_coding
2	72200001	72250000	0.44665	2	72155536	72206415	50880	-	ENSSSCG00000040016	NA	protein_coding
2	72200001	72250000	0.44665	2	72202614	72305886	103273	-	ENSSSCG00000013556	NA	protein_coding
2	72210001	72260000	0.464241	2	72202614	72305886	103273	-	ENSSSCG00000013556	NA	protein_coding
2	72220001	72270000	0.4957	2	72202614	72305886	103273	-	ENSSSCG00000013556	NA	protein_coding
2	72230001	72280000	0.508949	2	72202614	72305886	103273	-	ENSSSCG00000013556	NA	protein_coding
2	72240001	72290000	0.552691	2	72202614	72305886	103273	-	ENSSSCG00000013556	NA	protein_coding
2	72250001	72300000	0.46678	2	72202614	72305886	103273	-	ENSSSCG00000013556	NA	protein_coding
2	72280001	72330000	0.366951	2	72202614	72305886	103273	-	ENSSSCG00000013556	NA	protein_coding

2	72290001	72340000	0.380118	2	72202614	72305886	103273	-	ENSSSCG00000013556	NA	protein_coding
2	72300001	72350000	0.382101	2	72202614	72305886	103273	-	ENSSSCG00000013556	NA	protein_coding
2	72280001	72330000	0.366951	2	72326714	72391648	64935	-	ENSSSCG00000013553	VAV1	protein_coding
2	72290001	72340000	0.380118	2	72326714	72391648	64935	-	ENSSSCG00000013553	VAV1	protein_coding
2	72300001	72350000	0.382101	2	72326714	72391648	64935	-	ENSSSCG00000013553	VAV1	protein_coding
2	72310001	72360000	0.416364	2	72326714	72391648	64935	-	ENSSSCG00000013553	VAV1	protein_coding
2	72320001	72370000	0.416522	2	72326714	72391648	64935	-	ENSSSCG00000013553	VAV1	protein_coding
2	72330001	72380000	0.406416	2	72326714	72391648	64935	-	ENSSSCG00000013553	VAV1	protein_coding
2	72340001	72390000	0.392952	2	72326714	72391648	64935	-	ENSSSCG00000013553	VAV1	protein_coding
2	72350001	72400000	0.364801	2	72326714	72391648	64935	-	ENSSSCG00000013553	VAV1	protein_coding
2	72350001	72400000	0.364801	2	72394263	72406235	11973	+	ENSSSCG00000022380	SH2D3A	protein_coding
2	72480001	72530000	0.357451	2	72431499	72528112	96614	+	ENSSSCG00000013551	C3	protein_coding
2	72500001	72550000	0.35829	2	72431499	72528112	96614	+	ENSSSCG00000013551	C3	protein_coding
2	72480001	72530000	0.357451	2	72473477	72484017	10541	+	ENSSSCG00000026695	TNFSF14	protein_coding
2	72480001	72530000	0.357451	2	72528527	72533717	5191	+	ENSSSCG00000031115	CD70	protein_coding
2	72500001	72550000	0.35829	2	72528527	72533717	5191	+	ENSSSCG00000031115	CD70	protein_coding
2	72650001	72700000	0.370133	2	72647095	72655473	8379	+	ENSSSCG00000032785	SLC25A41	protein_coding
2	72650001	72700000	0.370133	2	72656951	72668964	12014	+	ENSSSCG00000013538	KHSRP	protein_coding
2	72660001	72710000	0.394335	2	72656951	72668964	12014	+	ENSSSCG00000013538	KHSRP	protein_coding
2	72650001	72700000	0.370133	2	72687790	72701971	14182	+	ENSSSCG00000013539	GTF2F1	protein_coding
2	72660001	72710000	0.394335	2	72687790	72701971	14182	+	ENSSSCG00000013539	GTF2F1	protein_coding
2	72670001	72720000	0.405296	2	72687790	72701971	14182	+	ENSSSCG00000013539	GTF2F1	protein_coding
2	72680001	72730000	0.362717	2	72687790	72701971	14182	+	ENSSSCG00000013539	GTF2F1	protein_coding
2	72650001	72700000	0.370133	2	72698527	72703864	5338	-	ENSSSCG00000024254	ALKBH7	protein_coding
2	72660001	72710000	0.394335	2	72698527	72703864	5338	-	ENSSSCG00000024254	ALKBH7	protein_coding
2	72670001	72720000	0.405296	2	72698527	72703864	5338	-	ENSSSCG00000024254	ALKBH7	protein_coding
2	72680001	72730000	0.362717	2	72698527	72703864	5338	-	ENSSSCG00000024254	ALKBH7	protein_coding
2	72660001	72710000	0.394335	2	72701298	72701840	543	+	ENSSSCG00000013540	PSPN	protein_coding
2	72670001	72720000	0.405296	2	72701298	72701840	543	+	ENSSSCG00000013540	PSPN	protein_coding
2	72680001	72730000	0.362717	2	72701298	72701840	543	+	ENSSSCG00000013540	PSPN	protein_coding
2	72660001	72710000	0.394335	2	72707158	72713475	6318	-	ENSSSCG00000038079	CLPP	protein_coding
2	72670001	72720000	0.405296	2	72707158	72713475	6318	-	ENSSSCG00000038079	CLPP	protein_coding
2	72680001	72730000	0.362717	2	72707158	72713475	6318	-	ENSSSCG00000038079	CLPP	protein_coding
2	72780001	72830000	0.394494	2	72780947	72847691	66745	+	ENSSSCG00000013534	MLLT1	protein_coding
2	73000001	73050000	0.380033	2	72950056	73051181	101126	+	ENSSSCG00000035403	RFX2	protein_coding
2	73010001	73060000	0.353996	2	72950056	73051181	101126	+	ENSSSCG00000035403	RFX2	protein_coding
2	73020001	73070000	0.36919	2	72950056	73051181	101126	+	ENSSSCG00000035403	RFX2	protein_coding
2	73020001	73070000	0.36919	2	73062896	73123716	60821	+	ENSSSCG00000013533	RANBP3	protein_coding
2	73180001	73230000	0.400027	2	73173170	73188395	15226	-	ENSSSCG00000033413	NRTN	protein_coding
2	73180001	73230000	0.400027	2	73179161	73204979	25819	+	ENSSSCG00000040968	NA	protein_coding
2	73190001	73240000	0.415278	2	73179161	73204979	25819	+	ENSSSCG00000040968	NA	protein_coding
2	73200001	73250000	0.43344	2	73179161	73204979	25819	+	ENSSSCG00000040968	NA	protein_coding
2	73180001	73230000	0.400027	2	73207892	73266302	58411	-	ENSSSCG00000013527	CATSPERD	protein_coding
2	73190001	73240000	0.415278	2	73207892	73266302	58411	-	ENSSSCG00000013527	CATSPERD	protein_coding
2	73200001	73250000	0.43344	2	73207892	73266302	58411	-	ENSSSCG00000013527	CATSPERD	protein_coding

2	73210001	73260000	0.415311	2	73207892	73266302	58411	-	ENSSSCG00000013527	CATSPERD	protein_coding
2	73220001	73270000	0.429233	2	73207892	73266302	58411	-	ENSSSCG00000013527	CATSPERD	protein_coding
2	73230001	73280000	0.39343	2	73207892	73266302	58411	-	ENSSSCG00000013527	CATSPERD	protein_coding
2	73240001	73290000	0.385066	2	73207892	73266302	58411	-	ENSSSCG00000013527	CATSPERD	protein_coding
2	73250001	73300000	0.363587	2	73207892	73266302	58411	-	ENSSSCG00000013527	CATSPERD	protein_coding
2	73260001	73310000	0.354535	2	73207892	73266302	58411	-	ENSSSCG00000013527	CATSPERD	protein_coding
2	73220001	73270000	0.429233	2	73266258	73286774	20517	+	ENSSSCG00000027128	LONP1	protein_coding
2	73230001	73280000	0.39343	2	73266258	73286774	20517	+	ENSSSCG00000027128	LONP1	protein_coding
2	73240001	73290000	0.385066	2	73266258	73286774	20517	+	ENSSSCG00000027128	LONP1	protein_coding
2	73250001	73300000	0.363587	2	73266258	73286774	20517	+	ENSSSCG00000027128	LONP1	protein_coding
2	73260001	73310000	0.354535	2	73266258	73286774	20517	+	ENSSSCG00000027128	LONP1	protein_coding
2	73240001	73290000	0.385066	2	73286903	73294713	7811	-	ENSSSCG00000030546	HSD11B1L	protein_coding
2	73250001	73300000	0.363587	2	73286903	73294713	7811	-	ENSSSCG00000030546	HSD11B1L	protein_coding
2	73260001	73310000	0.354535	2	73286903	73294713	7811	-	ENSSSCG00000030546	HSD11B1L	protein_coding
2	73250001	73300000	0.363587	2	73294842	73296836	1995	+	ENSSSCG00000013524	MICOS13	protein_coding
2	73260001	73310000	0.354535	2	73294842	73296836	1995	+	ENSSSCG00000013524	MICOS13	protein_coding
2	73260001	73310000	0.354535	2	73300632	73334494	33863	-	ENSSSCG00000013522	SAFB	protein_coding
2	73360001	73410000	0.368102	2	73334588	73396881	62294	+	ENSSSCG00000013523	SAFB2	protein_coding
9	99710001	99760000	0.353585	9	99754075	99754257	183	-	ENSSSCG00000049030	U2	snRNA
9	94740001	94790000	0.396288	9	94787934	94788036	103	+	ENSSSCG00000033088	U6	snRNA
9	94750001	94800000	0.398643	9	94787934	94788036	103	+	ENSSSCG00000033088	U6	snRNA
9	94760001	94810000	0.399396	9	94787934	94788036	103	+	ENSSSCG00000033088	U6	snRNA
9	94770001	94820000	0.395156	9	94787934	94788036	103	+	ENSSSCG00000033088	U6	snRNA
9	94780001	94830000	0.364807	9	94787934	94788036	103	+	ENSSSCG00000033088	U6	snRNA
9	118080001	118130000	0.441678	9	118123508	118128024	4517	-	ENSSSCG00000046238	NA	lncRNA
9	118090001	118140000	0.442722	9	118123508	118128024	4517	-	ENSSSCG00000046238	NA	lncRNA
9	118100001	118150000	0.390358	9	118123508	118128024	4517	-	ENSSSCG00000046238	NA	lncRNA
9	118100001	118150000	0.390358	9	118141640	118147530	5891	-	ENSSSCG00000044431	NA	lncRNA
9	118300001	118350000	0.370056	9	118318181	118361953	43773	-	ENSSSCG00000049556	NA	lncRNA
9	118310001	118360000	0.383822	9	118318181	118361953	43773	-	ENSSSCG00000049556	NA	lncRNA
9	118320001	118370000	0.365228	9	118318181	118361953	43773	-	ENSSSCG00000049556	NA	lncRNA
9	48320001	48370000	0.360476	9	48322235	48357024	34790	-	ENSSSCG00000043623	NA	lncRNA
9	48330001	48380000	0.370607	9	48322235	48357024	34790	-	ENSSSCG00000043623	NA	lncRNA
9	91600001	91650000	0.39321	9	91600207	91600280	74	+	ENSSSCG00000019224	NA	snoRNA
9	92990001	93040000	0.386518	9	93022091	93022197	107	-	ENSSSCG00000039043	U6	snRNA
9	93000001	93050000	0.398409	9	93022091	93022197	107	-	ENSSSCG00000039043	U6	snRNA
9	93010001	93060000	0.419361	9	93022091	93022197	107	-	ENSSSCG00000039043	U6	snRNA
9	93020001	93070000	0.462337	9	93022091	93022197	107	-	ENSSSCG00000039043	U6	snRNA
9	92780001	92830000	0.455804	9	92825762	92825861	100	+	ENSSSCG00000039822	U6	snRNA
9	92790001	92840000	0.423511	9	92825762	92825861	100	+	ENSSSCG00000039822	U6	snRNA
9	92800001	92850000	0.3787	9	92825762	92825861	100	+	ENSSSCG00000039822	U6	snRNA
9	35940001	35990000	0.35456	9	35953395	35958907	5513	-	ENSSSCG00000048220	NA	lncRNA
9	35950001	36000000	0.354185	9	35953395	35958907	5513	-	ENSSSCG00000048220	NA	lncRNA
9	48320001	48370000	0.360476	9	48334312	48337826	3515	-	ENSSSCG00000043249	NA	lncRNA
9	48330001	48380000	0.370607	9	48334312	48337826	3515	-	ENSSSCG00000043249	NA	lncRNA

9	48320001	48370000	0.360476	9	48342252	48348764	6513	-	ENSSSCG00000049329	NA	lncRNA
9	48330001	48380000	0.370607	9	48342252	48348764	6513	-	ENSSSCG00000049329	NA	lncRNA
9	71570001	71620000	0.357414	9	71557146	71574974	17829	+	ENSSSCG00000046139	NA	lncRNA
9	118820001	118870000	0.356282	9	118825791	118825873	83	-	ENSSSCG00000018556	NA	miRNA
9	91600001	91650000	0.39321	9	91597913	91611939	14027	+	ENSSSCG00000045695	NA	lncRNA
9	91610001	91660000	0.405458	9	91597913	91611939	14027	+	ENSSSCG00000045695	NA	lncRNA
9	91600001	91650000	0.39321	9	91647871	91651626	3756	-	ENSSSCG00000050033	NA	lncRNA
9	91610001	91660000	0.405458	9	91647871	91651626	3756	-	ENSSSCG00000050033	NA	lncRNA
9	91620001	91670000	0.44458	9	91647871	91651626	3756	-	ENSSSCG00000050033	NA	lncRNA
9	91630001	91680000	0.406338	9	91647871	91651626	3756	-	ENSSSCG00000050033	NA	lncRNA
9	91640001	91690000	0.359629	9	91647871	91651626	3756	-	ENSSSCG00000050033	NA	lncRNA
9	91630001	91680000	0.406338	9	91670090	91682622	12533	-	ENSSSCG00000051736	NA	lncRNA
9	91640001	91690000	0.359629	9	91670090	91682622	12533	-	ENSSSCG00000051736	NA	lncRNA
9	92960001	93010000	0.358642	9	92962320	92964229	1910	-	ENSSSCG00000045626	NA	lncRNA
9	92960001	93010000	0.358642	9	93003053	93005954	2902	+	ENSSSCG00000045732	NA	lncRNA
9	92970001	93020000	0.362625	9	93003053	93005954	2902	+	ENSSSCG00000045732	NA	lncRNA
9	92990001	93040000	0.386518	9	93003053	93005954	2902	+	ENSSSCG00000045732	NA	lncRNA
9	93000001	93050000	0.398409	9	93003053	93005954	2902	+	ENSSSCG00000045732	NA	lncRNA
9	94850001	94900000	0.372465	9	94847496	94859258	11763	-	ENSSSCG00000047975	NA	lncRNA
9	117940001	117990000	0.365226	9	117915441	118114259	198819	-	ENSSSCG00000015509	COP1	protein_coding
9	117950001	118000000	0.375224	9	117915441	118114259	198819	-	ENSSSCG00000015509	COP1	protein_coding
9	117970001	118020000	0.357351	9	117915441	118114259	198819	-	ENSSSCG00000015509	COP1	protein_coding
9	117980001	118030000	0.395346	9	117915441	118114259	198819	-	ENSSSCG00000015509	COP1	protein_coding
9	117990001	118040000	0.415762	9	117915441	118114259	198819	-	ENSSSCG00000015509	COP1	protein_coding
9	118000001	118050000	0.425548	9	117915441	118114259	198819	-	ENSSSCG00000015509	COP1	protein_coding
9	118010001	118060000	0.490573	9	117915441	118114259	198819	-	ENSSSCG00000015509	COP1	protein_coding
9	118020001	118070000	0.480799	9	117915441	118114259	198819	-	ENSSSCG00000015509	COP1	protein_coding
9	118030001	118080000	0.461743	9	117915441	118114259	198819	-	ENSSSCG00000015509	COP1	protein_coding
9	118040001	118090000	0.410418	9	117915441	118114259	198819	-	ENSSSCG00000015509	COP1	protein_coding
9	118050001	118100000	0.419982	9	117915441	118114259	198819	-	ENSSSCG00000015509	COP1	protein_coding
9	118060001	118110000	0.438302	9	117915441	118114259	198819	-	ENSSSCG00000015509	COP1	protein_coding
9	118070001	118120000	0.425184	9	117915441	118114259	198819	-	ENSSSCG00000015509	COP1	protein_coding
9	118080001	118130000	0.441678	9	117915441	118114259	198819	-	ENSSSCG00000015509	COP1	protein_coding
9	118090001	118140000	0.442722	9	117915441	118114259	198819	-	ENSSSCG00000015509	COP1	protein_coding
9	118100001	118150000	0.390358	9	117915441	118114259	198819	-	ENSSSCG00000015509	COP1	protein_coding
9	118320001	118370000	0.365228	9	118364592	118635969	271378	+	ENSSSCG00000015512	PAPPA2	protein_coding
9	118820001	118870000	0.356282	9	118646910	118968970	322061	-	ENSSSCG00000015513	ASTN1	protein_coding
9	118830001	118880000	0.357278	9	118646910	118968970	322061	-	ENSSSCG00000015513	ASTN1	protein_coding
9	48320001	48370000	0.360476	9	48357145	48372388	15244	+	ENSSSCG00000028517	SC5D	protein_coding
9	48330001	48380000	0.370607	9	48357145	48372388	15244	+	ENSSSCG00000028517	SC5D	protein_coding
9	48320001	48370000	0.360476	9	48357165	48365513	8349	+	ENSSSCG00000051213	NA	protein_coding
9	48330001	48380000	0.370607	9	48357165	48365513	8349	+	ENSSSCG00000051213	NA	protein_coding
9	48320001	48370000	0.360476	9	48361054	48365541	4488	-	ENSSSCG00000050836	NA	pseudogene
9	48330001	48380000	0.370607	9	48361054	48365541	4488	-	ENSSSCG00000050836	NA	pseudogene
9	77550001	77600000	0.372067	9	77566197	77570689	4493	-	ENSSSCG00000049770	NA	protein_coding

9	77560001	77610000	0.397102	9	77566197	77570689	4493	-	ENSSSCG00000049770	NA	protein_coding
9	77570001	77620000	0.380272	9	77566197	77570689	4493	-	ENSSSCG00000049770	NA	protein_coding
9	102980001	103030000	0.369248	9	102989945	102996482	6538	+	ENSSSCG00000043224	NA	protein_coding
9	102990001	103040000	0.43787	9	102989945	102996482	6538	+	ENSSSCG00000043224	NA	protein_coding
9	75580001	75630000	0.355271	9	75336441	75760523	424083	+	ENSSSCG00000015335	DYNC1I1	protein_coding
9	76030001	76080000	0.387299	9	75754392	76349242	594851	-	ENSSSCG00000015336	SLC25A13	protein_coding
9	76040001	76090000	0.399962	9	75754392	76349242	594851	-	ENSSSCG00000015336	SLC25A13	protein_coding
9	76050001	76100000	0.419026	9	75754392	76349242	594851	-	ENSSSCG00000015336	SLC25A13	protein_coding
9	76060001	76110000	0.424838	9	75754392	76349242	594851	-	ENSSSCG00000015336	SLC25A13	protein_coding
9	76070001	76120000	0.395318	9	75754392	76349242	594851	-	ENSSSCG00000015336	SLC25A13	protein_coding
9	77620001	77670000	0.413813	9	77666260	77833806	167547	-	ENSSSCG00000015342	COL28A1	protein_coding
9	77630001	77680000	0.437129	9	77666260	77833806	167547	-	ENSSSCG00000015342	COL28A1	protein_coding
9	77640001	77690000	0.403327	9	77666260	77833806	167547	-	ENSSSCG00000015342	COL28A1	protein_coding
9	77650001	77700000	0.369219	9	77666260	77833806	167547	-	ENSSSCG00000015342	COL28A1	protein_coding
9	77940001	77990000	0.396827	9	77914958	78216736	301779	+	ENSSSCG00000036169	UMAD1	protein_coding
9	77950001	78000000	0.371891	9	77914958	78216736	301779	+	ENSSSCG00000036169	UMAD1	protein_coding
9	77940001	77990000	0.396827	9	77915022	78334085	419064	+	ENSSSCG00000015345	GLCCI1	protein_coding
9	77950001	78000000	0.371891	9	77915022	78334085	419064	+	ENSSSCG00000015345	GLCCI1	protein_coding
9	78310001	78360000	0.364108	9	77915022	78334085	419064	+	ENSSSCG00000015345	GLCCI1	protein_coding
9	78310001	78360000	0.364108	9	78332374	78515087	182714	-	ENSSSCG00000015346	ICA1	protein_coding
9	90750001	90800000	0.363224	9	90423077	90767487	344411	+	ENSSSCG00000015379	DNAH11	protein_coding
9	90760001	90810000	0.364438	9	90423077	90767487	344411	+	ENSSSCG00000015379	DNAH11	protein_coding
9	90750001	90800000	0.363224	9	90766767	90806865	40099	-	ENSSSCG00000015380	CDCA7L	protein_coding
9	90760001	90810000	0.364438	9	90766767	90806865	40099	-	ENSSSCG00000015380	CDCA7L	protein_coding
9	90770001	90820000	0.374967	9	90766767	90806865	40099	-	ENSSSCG00000015380	CDCA7L	protein_coding
9	90780001	90830000	0.370221	9	90766767	90806865	40099	-	ENSSSCG00000015380	CDCA7L	protein_coding
9	91790001	91840000	0.403259	9	91729054	91821619	92566	-	ENSSSCG00000015386	FAM126A	protein_coding
9	91800001	91850000	0.398636	9	91729054	91821619	92566	-	ENSSSCG00000015386	FAM126A	protein_coding
9	91810001	91860000	0.408221	9	91729054	91821619	92566	-	ENSSSCG00000015386	FAM126A	protein_coding
9	91820001	91870000	0.419364	9	91729054	91821619	92566	-	ENSSSCG00000015386	FAM126A	protein_coding
9	91860001	91910000	0.451002	9	91903823	91971695	67873	+	ENSSSCG00000038469	KLHL7	protein_coding
9	91870001	91920000	0.441411	9	91903823	91971695	67873	+	ENSSSCG00000038469	KLHL7	protein_coding
9	91880001	91930000	0.383916	9	91903823	91971695	67873	+	ENSSSCG00000038469	KLHL7	protein_coding
9	92070001	92120000	0.362462	9	92037423	92118040	80618	+	ENSSSCG00000032241	GPNMB	protein_coding
9	92070001	92120000	0.362462	9	92088419	92109293	20875	+	ENSSSCG00000036722	MALSU1	protein_coding
9	92070001	92120000	0.362462	9	92104847	92272211	167365	-	ENSSSCG00000036695	IGF2BP3	protein_coding
9	92420001	92470000	0.362073	9	92372069	92450367	78299	+	ENSSSCG00000029174	CCDC126	protein_coding
9	92430001	92480000	0.38288	9	92372069	92450367	78299	+	ENSSSCG00000029174	CCDC126	protein_coding
9	92440001	92490000	0.378965	9	92372069	92450367	78299	+	ENSSSCG00000029174	CCDC126	protein_coding
9	92420001	92470000	0.362073	9	92448731	92481379	32649	-	ENSSSCG00000020870	NA	protein_coding
9	92430001	92480000	0.38288	9	92448731	92481379	32649	-	ENSSSCG00000020870	NA	protein_coding
9	92440001	92490000	0.378965	9	92448731	92481379	32649	-	ENSSSCG00000020870	NA	protein_coding
9	92440001	92490000	0.378965	9	92481564	92528186	46623	+	ENSSSCG00000026177	SLC25A40	protein_coding
9	92690001	92740000	0.357443	9	92528660	92823561	294902	-	ENSSSCG00000024485	RUNDC3B	protein_coding
9	92710001	92760000	0.358957	9	92528660	92823561	294902	-	ENSSSCG00000024485	RUNDC3B	protein_coding

9	92720001	92770000	0.358269	9	92528660	92823561	294902	-	ENSSSCG00000024485	RUNDC3B	protein_coding
9	92750001	92800000	0.390037	9	92528660	92823561	294902	-	ENSSSCG00000024485	RUNDC3B	protein_coding
9	92760001	92810000	0.412417	9	92528660	92823561	294902	-	ENSSSCG00000024485	RUNDC3B	protein_coding
9	92770001	92820000	0.447074	9	92528660	92823561	294902	-	ENSSSCG00000024485	RUNDC3B	protein_coding
9	92780001	92830000	0.455804	9	92528660	92823561	294902	-	ENSSSCG00000024485	RUNDC3B	protein_coding
9	92790001	92840000	0.423511	9	92528660	92823561	294902	-	ENSSSCG00000024485	RUNDC3B	protein_coding
9	92800001	92850000	0.3787	9	92528660	92823561	294902	-	ENSSSCG00000024485	RUNDC3B	protein_coding
9	92690001	92740000	0.357443	9	92718185	92800791	82607	+	ENSSSCG00000032456	NA	protein_coding
9	92710001	92760000	0.358957	9	92718185	92800791	82607	+	ENSSSCG00000032456	NA	protein_coding
9	92720001	92770000	0.358269	9	92718185	92800791	82607	+	ENSSSCG00000032456	NA	protein_coding
9	92750001	92800000	0.390037	9	92718185	92800791	82607	+	ENSSSCG00000032456	NA	protein_coding
9	92760001	92810000	0.412417	9	92718185	92800791	82607	+	ENSSSCG00000032456	NA	protein_coding
9	92770001	92820000	0.447074	9	92718185	92800791	82607	+	ENSSSCG00000032456	NA	protein_coding
9	92780001	92830000	0.455804	9	92718185	92800791	82607	+	ENSSSCG00000032456	NA	protein_coding
9	92790001	92840000	0.423511	9	92718185	92800791	82607	+	ENSSSCG00000032456	NA	protein_coding
9	92800001	92850000	0.3787	9	92718185	92800791	82607	+	ENSSSCG00000032456	NA	protein_coding
9	92800001	92850000	0.3787	9	92844889	92925766	80878	+	ENSSSCG00000035073	NA	protein_coding
9	93010001	93060000	0.419361	9	93050121	93146467	96347	+	ENSSSCG00000015390	NA	protein_coding
9	93020001	93070000	0.462337	9	93050121	93146467	96347	+	ENSSSCG00000015390	NA	protein_coding
9	93030001	93080000	0.502038	9	93050121	93146467	96347	+	ENSSSCG00000015390	NA	protein_coding
9	93040001	93090000	0.485327	9	93050121	93146467	96347	+	ENSSSCG00000015390	NA	protein_coding
9	93050001	93100000	0.449687	9	93050121	93146467	96347	+	ENSSSCG00000015390	NA	protein_coding
9	93060001	93110000	0.406424	9	93050121	93146467	96347	+	ENSSSCG00000015390	NA	protein_coding
9	93070001	93120000	0.356412	9	93050121	93146467	96347	+	ENSSSCG00000015390	NA	protein_coding
9	93050001	93100000	0.449687	9	93098776	93261882	163107	+	ENSSSCG00000024520	NA	protein_coding
9	93060001	93110000	0.406424	9	93098776	93261882	163107	+	ENSSSCG00000024520	NA	protein_coding
9	93070001	93120000	0.356412	9	93098776	93261882	163107	+	ENSSSCG00000024520	NA	protein_coding
9	93390001	93440000	0.379076	9	93393730	93416627	22898	+	ENSSSCG00000025326	TMEM243	protein_coding
9	93390001	93440000	0.379076	9	93416454	93463042	46589	-	ENSSSCG00000015393	DMTF1	protein_coding
9	93700001	93750000	0.383561	9	93540277	93723890	183614	+	ENSSSCG00000015394	ELAPOR2	protein_coding
9	93710001	93760000	0.435321	9	93540277	93723890	183614	+	ENSSSCG00000015394	ELAPOR2	protein_coding
9	93720001	93770000	0.393793	9	93540277	93723890	183614	+	ENSSSCG00000015394	ELAPOR2	protein_coding
9	93700001	93750000	0.383561	9	93731210	93973729	242520	-	ENSSSCG00000015395	GRM3	protein_coding
9	93710001	93760000	0.435321	9	93731210	93973729	242520	-	ENSSSCG00000015395	GRM3	protein_coding
9	93720001	93770000	0.393793	9	93731210	93973729	242520	-	ENSSSCG00000015395	GRM3	protein_coding
9	93730001	93780000	0.379105	9	93731210	93973729	242520	-	ENSSSCG00000015395	GRM3	protein_coding
9	93740001	93790000	0.378298	9	93731210	93973729	242520	-	ENSSSCG00000015395	GRM3	protein_coding
9	93790001	93840000	0.353804	9	93731210	93973729	242520	-	ENSSSCG00000015395	GRM3	protein_coding
9	98260001	98310000	0.355019	9	97780742	98266573	485832	+	ENSSSCG00000015402	CACNA2D1	protein_coding
9	99250001	99300000	0.374144	9	99251714	99433230	181517	+	ENSSSCG00000023784	SEMA3C	protein_coding
9	99260001	99310000	0.374282	9	99251714	99433230	181517	+	ENSSSCG00000023784	SEMA3C	protein_coding
9	99520001	99570000	0.35313	9	99535642	99537277	1636	-	ENSSSCG00000037311	NA	protein_coding
9	99520001	99570000	0.35313	9	99552915	99554486	1572	-	ENSSSCG00000034602	NA	protein_coding
9	99560001	99610000	0.353865	9	99570656	99572292	1637	-	ENSSSCG00000037410	NA	protein_coding
9	99560001	99610000	0.353865	9	99605181	99782105	176925	-	ENSSSCG00000015405	CD36	protein_coding

9	99630001	99680000	0.356517	9	99605181	99782105	176925	-	ENSSSCG00000015405	CD36	protein_coding
9	99640001	99690000	0.381139	9	99605181	99782105	176925	-	ENSSSCG00000015405	CD36	protein_coding
9	99650001	99700000	0.393527	9	99605181	99782105	176925	-	ENSSSCG00000015405	CD36	protein_coding
9	99660001	99710000	0.389793	9	99605181	99782105	176925	-	ENSSSCG00000015405	CD36	protein_coding
9	99670001	99720000	0.369168	9	99605181	99782105	176925	-	ENSSSCG00000015405	CD36	protein_coding
9	99710001	99760000	0.353585	9	99605181	99782105	176925	-	ENSSSCG00000015405	CD36	protein_coding
9	99710001	99760000	0.353585	9	99723594	99727412	3819	+	ENSSSCG00000045337	NA	protein_coding
9	99820001	99870000	0.359083	9	99859832	99911761	51930	+	ENSSSCG00000015406	GNAT3	protein_coding
9	101750001	101800000	0.366719	9	100847212	102169003	1321792	+	ENSSSCG00000031780	MAGI2	protein_coding
9	101760001	101810000	0.376483	9	100847212	102169003	1321792	+	ENSSSCG00000031780	MAGI2	protein_coding
9	101770001	101820000	0.369321	9	100847212	102169003	1321792	+	ENSSSCG00000031780	MAGI2	protein_coding
9	101750001	101800000	0.366719	9	101770034	101772557	2524	-	ENSSSCG00000041553	NA	pseudogene
9	101760001	101810000	0.376483	9	101770034	101772557	2524	-	ENSSSCG00000041553	NA	pseudogene
9	101770001	101820000	0.369321	9	101770034	101772557	2524	-	ENSSSCG00000041553	NA	pseudogene
9	102810001	102860000	0.366335	9	102787548	102919688	132141	-	ENSSSCG00000015412	CCDC146	protein_coding
9	102820001	102870000	0.384323	9	102787548	102919688	132141	-	ENSSSCG00000015412	CCDC146	protein_coding
9	102830001	102880000	0.395202	9	102787548	102919688	132141	-	ENSSSCG00000015412	CCDC146	protein_coding
9	102840001	102890000	0.371546	9	102787548	102919688	132141	-	ENSSSCG00000015412	CCDC146	protein_coding
9	102820001	102870000	0.384323	9	102868836	102875577	6742	+	ENSSSCG00000015413	FGL2	protein_coding
9	102830001	102880000	0.395202	9	102868836	102875577	6742	+	ENSSSCG00000015413	FGL2	protein_coding
9	102840001	102890000	0.371546	9	102868836	102875577	6742	+	ENSSSCG00000015413	FGL2	protein_coding
9	102980001	103030000	0.369248	9	102977129	103152572	175444	-	ENSSSCG00000037822	FBXL13	protein_coding
9	102990001	103040000	0.43787	9	102977129	103152572	175444	-	ENSSSCG00000037822	FBXL13	protein_coding
9	103000001	103050000	0.49217	9	102977129	103152572	175444	-	ENSSSCG00000037822	FBXL13	protein_coding
9	103010001	103060000	0.531166	9	102977129	103152572	175444	-	ENSSSCG00000037822	FBXL13	protein_coding
9	103020001	103070000	0.468946	9	102977129	103152572	175444	-	ENSSSCG00000037822	FBXL13	protein_coding
9	103030001	103080000	0.400999	9	102977129	103152572	175444	-	ENSSSCG00000037822	FBXL13	protein_coding
9	103150001	103200000	0.445574	9	102977129	103152572	175444	-	ENSSSCG00000037822	FBXL13	protein_coding
9	103020001	103070000	0.468946	9	103062867	103122481	59615	+	ENSSSCG00000036452	LRRC17	protein_coding
9	103030001	103080000	0.400999	9	103062867	103122481	59615	+	ENSSSCG00000036452	LRRC17	protein_coding
9	103150001	103200000	0.445574	9	103179503	103292109	112607	+	ENSSSCG00000028444	ARMC10	protein_coding
9	103160001	103210000	0.537503	9	103179503	103292109	112607	+	ENSSSCG00000028444	ARMC10	protein_coding
9	103170001	103220000	0.510329	9	103179503	103292109	112607	+	ENSSSCG00000028444	ARMC10	protein_coding
9	103180001	103230000	0.494597	9	103179503	103292109	112607	+	ENSSSCG00000028444	ARMC10	protein_coding
9	103190001	103240000	0.4985	9	103179503	103292109	112607	+	ENSSSCG00000028444	ARMC10	protein_coding
9	103200001	103250000	0.431912	9	103179503	103292109	112607	+	ENSSSCG00000028444	ARMC10	protein_coding
9	103210001	103260000	0.43722	9	103179503	103292109	112607	+	ENSSSCG00000028444	ARMC10	protein_coding
9	103220001	103270000	0.438675	9	103179503	103292109	112607	+	ENSSSCG00000028444	ARMC10	protein_coding
9	103230001	103280000	0.413894	9	103179503	103292109	112607	+	ENSSSCG00000028444	ARMC10	protein_coding
9	103240001	103290000	0.406995	9	103179503	103292109	112607	+	ENSSSCG00000028444	ARMC10	protein_coding
9	103250001	103300000	0.401029	9	103179503	103292109	112607	+	ENSSSCG00000028444	ARMC10	protein_coding
9	103170001	103220000	0.510329	9	103210748	103262454	51707	-	ENSSSCG00000022903	NAPEPLD	protein_coding
9	103180001	103230000	0.494597	9	103210748	103262454	51707	-	ENSSSCG00000022903	NAPEPLD	protein_coding
9	103190001	103240000	0.4985	9	103210748	103262454	51707	-	ENSSSCG00000022903	NAPEPLD	protein_coding
9	103200001	103250000	0.431912	9	103210748	103262454	51707	-	ENSSSCG00000022903	NAPEPLD	protein_coding

9	103210001	103260000	0.43722	9	103210748	103262454	51707	-	ENSSSCG00000022903	NAPEPLD	protein_coding
9	103220001	103270000	0.438675	9	103210748	103262454	51707	-	ENSSSCG00000022903	NAPEPLD	protein_coding
9	103230001	103280000	0.413894	9	103210748	103262454	51707	-	ENSSSCG00000022903	NAPEPLD	protein_coding
9	103240001	103290000	0.406995	9	103210748	103262454	51707	-	ENSSSCG00000022903	NAPEPLD	protein_coding
9	103250001	103300000	0.401029	9	103210748	103262454	51707	-	ENSSSCG00000022903	NAPEPLD	protein_coding
9	103410001	103460000	0.397303	9	103378776	103453730	74955	-	ENSSSCG00000015425	SLC26A5	protein_coding
9	103420001	103470000	0.363472	9	103378776	103453730	74955	-	ENSSSCG00000015425	SLC26A5	protein_coding
9	103430001	103480000	0.408617	9	103378776	103453730	74955	-	ENSSSCG00000015425	SLC26A5	protein_coding
9	103440001	103490000	0.361453	9	103378776	103453730	74955	-	ENSSSCG00000015425	SLC26A5	protein_coding
9	103420001	103470000	0.363472	9	103460857	103963965	503109	-	ENSSSCG00000015426	RELN	protein_coding
9	103430001	103480000	0.408617	9	103460857	103963965	503109	-	ENSSSCG00000015426	RELN	protein_coding
9	103440001	103490000	0.361453	9	103460857	103963965	503109	-	ENSSSCG00000015426	RELN	protein_coding
9	35940001	35990000	0.35456	9	35904029	35948575	44547	+	ENSSSCG00000014998	AASDHPPT	protein_coding
9	35940001	35990000	0.35456	9	35967044	36059749	92706	-	ENSSSCG00000028201	CWF19L2	protein_coding
9	35950001	36000000	0.354185	9	35967044	36059749	92706	-	ENSSSCG00000028201	CWF19L2	protein_coding
9	35960001	36010000	0.357739	9	35967044	36059749	92706	-	ENSSSCG00000028201	CWF19L2	protein_coding
9	35970001	36020000	0.365118	9	35967044	36059749	92706	-	ENSSSCG00000028201	CWF19L2	protein_coding
5	82820001	82870000	0.388667	5	82813714	82913155	99442	-	ENSSSCG00000000869	UTP20	protein_coding
5	97000001	97050000	0.357274	5	97047949	97049000	1052	+	ENSSSCG00000041909	NA	processed_pseudogene
5	97010001	97060000	0.356341	5	97047949	97049000	1052	+	ENSSSCG00000041909	NA	processed_pseudogene
5	51670001	51720000	0.423403	5	51711017	51711123	107	-	ENSSSCG00000034143	U6	snRNA
5	51680001	51730000	0.466222	5	51711017	51711123	107	-	ENSSSCG00000034143	U6	snRNA
5	51690001	51740000	0.466825	5	51711017	51711123	107	-	ENSSSCG00000034143	U6	snRNA
5	51700001	51750000	0.4566	5	51711017	51711123	107	-	ENSSSCG00000034143	U6	snRNA
5	51710001	51760000	0.466905	5	51711017	51711123	107	-	ENSSSCG00000034143	U6	snRNA
5	83920001	83970000	0.360223	5	83908876	83929054	20179	-	ENSSSCG00000025682	ACTR6	protein_coding
5	83920001	83970000	0.360223	5	83935703	84041756	106054	+	ENSSSCG00000026697	UHRF1BP1L	protein_coding
5	83930001	83980000	0.445187	5	83935703	84041756	106054	+	ENSSSCG00000026697	UHRF1BP1L	protein_coding
5	83940001	83990000	0.442496	5	83935703	84041756	106054	+	ENSSSCG00000026697	UHRF1BP1L	protein_coding
5	83950001	84000000	0.437637	5	83935703	84041756	106054	+	ENSSSCG00000026697	UHRF1BP1L	protein_coding
5	83960001	84010000	0.417696	5	83935703	84041756	106054	+	ENSSSCG00000026697	UHRF1BP1L	protein_coding
5	42300001	42350000	0.373057	5	42299653	42310144	10492	+	ENSSSCG00000046199	NA	lncRNA
5	45520001	45570000	0.373017	5	45545283	45548262	2980	-	ENSSSCG00000033817	NA	protein_coding
5	45530001	45580000	0.370781	5	45545283	45548262	2980	-	ENSSSCG00000033817	NA	protein_coding
5	45540001	45590000	0.363206	5	45545283	45548262	2980	-	ENSSSCG00000033817	NA	protein_coding
5	45000001	45050000	0.369361	5	45048223	45053726	5504	-	ENSSSCG00000044976	NA	protein_coding
5	45010001	45060000	0.365671	5	45048223	45053726	5504	-	ENSSSCG00000044976	NA	protein_coding
5	96800001	96850000	0.354705	5	96801326	97093718	292393	-	ENSSSCG00000000934	LRRIQ1	protein_coding
5	96810001	96860000	0.369849	5	96801326	97093718	292393	-	ENSSSCG00000000934	LRRIQ1	protein_coding
5	96990001	97040000	0.360427	5	96801326	97093718	292393	-	ENSSSCG00000000934	LRRIQ1	protein_coding
5	97000001	97050000	0.357274	5	96801326	97093718	292393	-	ENSSSCG00000000934	LRRIQ1	protein_coding
5	97010001	97060000	0.356341	5	96801326	97093718	292393	-	ENSSSCG00000000934	LRRIQ1	protein_coding
5	96990001	97040000	0.360427	5	96993189	97021369	28181	+	ENSSSCG00000040399	TSPAN19	protein_coding
5	97000001	97050000	0.357274	5	96993189	97021369	28181	+	ENSSSCG00000040399	TSPAN19	protein_coding
5	97010001	97060000	0.356341	5	96993189	97021369	28181	+	ENSSSCG00000040399	TSPAN19	protein_coding

5	41670001	41720000	0.369612	5	41570282	41879062	308781	-	ENSSSCG00000000530	NA	protein_coding
5	41740001	41790000	0.38311	5	41570282	41879062	308781	-	ENSSSCG00000000530	NA	protein_coding
5	41750001	41800000	0.357371	5	41570282	41879062	308781	-	ENSSSCG00000000530	NA	protein_coding
5	41770001	41820000	0.361494	5	41570282	41879062	308781	-	ENSSSCG00000000530	NA	protein_coding
5	43180001	43230000	0.371215	5	43099749	43182803	83055	+	ENSSSCG00000000534	IPO8	protein_coding
5	43180001	43230000	0.371215	5	43223105	43239359	16255	+	ENSSSCG00000040992	NA	protein_coding
5	43960001	44010000	0.356887	5	43913655	44218049	304395	+	ENSSSCG00000027447	TMTC1	protein_coding
5	43970001	44020000	0.386137	5	43913655	44218049	304395	+	ENSSSCG00000027447	TMTC1	protein_coding
5	43980001	44030000	0.382205	5	43913655	44218049	304395	+	ENSSSCG00000027447	TMTC1	protein_coding
5	43990001	44040000	0.368716	5	43913655	44218049	304395	+	ENSSSCG00000027447	TMTC1	protein_coding
5	45240001	45290000	0.357453	5	45138035	45525650	387616	-	ENSSSCG00000024232	CCDC91	protein_coding
5	45250001	45300000	0.379696	5	45138035	45525650	387616	-	ENSSSCG00000024232	CCDC91	protein_coding
5	45260001	45310000	0.397048	5	45138035	45525650	387616	-	ENSSSCG00000024232	CCDC91	protein_coding
5	45270001	45320000	0.421395	5	45138035	45525650	387616	-	ENSSSCG00000024232	CCDC91	protein_coding
5	45280001	45330000	0.40646	5	45138035	45525650	387616	-	ENSSSCG00000024232	CCDC91	protein_coding
5	45310001	45360000	0.367998	5	45138035	45525650	387616	-	ENSSSCG00000024232	CCDC91	protein_coding
5	45320001	45370000	0.388648	5	45138035	45525650	387616	-	ENSSSCG00000024232	CCDC91	protein_coding
5	45330001	45380000	0.423144	5	45138035	45525650	387616	-	ENSSSCG00000024232	CCDC91	protein_coding
5	45340001	45390000	0.439463	5	45138035	45525650	387616	-	ENSSSCG00000024232	CCDC91	protein_coding
5	45350001	45400000	0.451479	5	45138035	45525650	387616	-	ENSSSCG00000024232	CCDC91	protein_coding
5	45360001	45410000	0.412911	5	45138035	45525650	387616	-	ENSSSCG00000024232	CCDC91	protein_coding
5	45370001	45420000	0.400407	5	45138035	45525650	387616	-	ENSSSCG00000024232	CCDC91	protein_coding
5	45380001	45430000	0.384667	5	45138035	45525650	387616	-	ENSSSCG00000024232	CCDC91	protein_coding
5	45390001	45440000	0.378357	5	45138035	45525650	387616	-	ENSSSCG00000024232	CCDC91	protein_coding
5	45400001	45450000	0.365519	5	45138035	45525650	387616	-	ENSSSCG00000024232	CCDC91	protein_coding
5	45410001	45460000	0.365529	5	45138035	45525650	387616	-	ENSSSCG00000024232	CCDC91	protein_coding
5	45420001	45470000	0.377731	5	45138035	45525650	387616	-	ENSSSCG00000024232	CCDC91	protein_coding
5	45430001	45480000	0.377433	5	45138035	45525650	387616	-	ENSSSCG00000024232	CCDC91	protein_coding
5	45440001	45490000	0.370142	5	45138035	45525650	387616	-	ENSSSCG00000024232	CCDC91	protein_coding
5	45450001	45500000	0.36286	5	45138035	45525650	387616	-	ENSSSCG00000024232	CCDC91	protein_coding
5	45520001	45570000	0.373017	5	45138035	45525650	387616	-	ENSSSCG00000024232	CCDC91	protein_coding
5	45310001	45360000	0.367998	5	45351322	45356341	5020	+	ENSSSCG00000046415	NA	pseudogene
5	45320001	45370000	0.388648	5	45351322	45356341	5020	+	ENSSSCG00000046415	NA	pseudogene
5	45330001	45380000	0.423144	5	45351322	45356341	5020	+	ENSSSCG00000046415	NA	pseudogene
5	45340001	45390000	0.439463	5	45351322	45356341	5020	+	ENSSSCG00000046415	NA	pseudogene
5	45350001	45400000	0.451479	5	45351322	45356341	5020	+	ENSSSCG00000046415	NA	pseudogene
5	46350001	46400000	0.353998	5	46290005	46482280	192276	-	ENSSSCG00000000551	ARNTL2	protein_coding
5	46360001	46410000	0.374272	5	46290005	46482280	192276	-	ENSSSCG00000000551	ARNTL2	protein_coding
5	46370001	46420000	0.412618	5	46290005	46482280	192276	-	ENSSSCG00000000551	ARNTL2	protein_coding
5	46380001	46430000	0.432126	5	46290005	46482280	192276	-	ENSSSCG00000000551	ARNTL2	protein_coding
5	46390001	46440000	0.453824	5	46290005	46482280	192276	-	ENSSSCG00000000551	ARNTL2	protein_coding
5	46400001	46450000	0.442827	5	46290005	46482280	192276	-	ENSSSCG00000000551	ARNTL2	protein_coding
5	46410001	46460000	0.417189	5	46290005	46482280	192276	-	ENSSSCG00000000551	ARNTL2	protein_coding
5	49500001	49550000	0.399444	5	49160723	50161570	1000848	+	ENSSSCG00000000567	SOX5	protein_coding
5	49510001	49560000	0.427564	5	49160723	50161570	1000848	+	ENSSSCG00000000567	SOX5	protein_coding

5	49520001	49570000	0.429688	5	49160723	50161570	1000848	+	ENSSSCG00000000567	SOX5	protein_coding
5	49530001	49580000	0.40114	5	49160723	50161570	1000848	+	ENSSSCG00000000567	SOX5	protein_coding
5	51490001	51540000	0.47174	5	51536526	51673583	137058	+	ENSSSCG00000000571	ABCC9	protein_coding
5	51500001	51550000	0.364269	5	51536526	51673583	137058	+	ENSSSCG00000000571	ABCC9	protein_coding
5	51660001	51710000	0.380917	5	51536526	51673583	137058	+	ENSSSCG00000000571	ABCC9	protein_coding
5	51670001	51720000	0.423403	5	51536526	51673583	137058	+	ENSSSCG00000000571	ABCC9	protein_coding
5	51490001	51540000	0.47174	5	51537774	51703817	166044	+	ENSSSCG00000000573	KCNJ8	protein_coding
5	51500001	51550000	0.364269	5	51537774	51703817	166044	+	ENSSSCG00000000573	KCNJ8	protein_coding
5	51660001	51710000	0.380917	5	51537774	51703817	166044	+	ENSSSCG00000000573	KCNJ8	protein_coding
5	51670001	51720000	0.423403	5	51537774	51703817	166044	+	ENSSSCG00000000573	KCNJ8	protein_coding
5	51680001	51730000	0.466222	5	51537774	51703817	166044	+	ENSSSCG00000000573	KCNJ8	protein_coding
5	51690001	51740000	0.466825	5	51537774	51703817	166044	+	ENSSSCG00000000573	KCNJ8	protein_coding
5	51700001	51750000	0.4566	5	51537774	51703817	166044	+	ENSSSCG00000000573	KCNJ8	protein_coding
5	51760001	51810000	0.464227	5	51801251	51801960	710	+	ENSSSCG00000038537	NA	protein_coding
5	51770001	51820000	0.415437	5	51801251	51801960	710	+	ENSSSCG00000038537	NA	protein_coding
5	51780001	51830000	0.410338	5	51801251	51801960	710	+	ENSSSCG00000038537	NA	protein_coding
5	51790001	51840000	0.380924	5	51801251	51801960	710	+	ENSSSCG00000038537	NA	protein_coding
5	51770001	51820000	0.415437	5	51810943	51831217	20275	+	ENSSSCG00000000576	NA	protein_coding
5	51780001	51830000	0.410338	5	51810943	51831217	20275	+	ENSSSCG00000000576	NA	protein_coding
5	51790001	51840000	0.380924	5	51810943	51831217	20275	+	ENSSSCG00000000576	NA	protein_coding
5	51940001	51990000	0.384282	5	51920398	51953316	32919	-	ENSSSCG00000000579	GOLT1B	protein_coding
5	51950001	52000000	0.39624	5	51920398	51953316	32919	-	ENSSSCG00000000579	GOLT1B	protein_coding
5	51940001	51990000	0.384282	5	51953184	52003742	50559	+	ENSSSCG00000000580	RECQL	protein_coding
5	51950001	52000000	0.39624	5	51953184	52003742	50559	+	ENSSSCG00000000580	RECQL	protein_coding
5	51960001	52010000	0.419126	5	51953184	52003742	50559	+	ENSSSCG00000000580	RECQL	protein_coding
5	51970001	52020000	0.432199	5	51953184	52003742	50559	+	ENSSSCG00000000580	RECQL	protein_coding
5	51980001	52030000	0.418492	5	51953184	52003742	50559	+	ENSSSCG00000000580	RECQL	protein_coding
5	51990001	52040000	0.369049	5	51953184	52003742	50559	+	ENSSSCG00000000580	RECQL	protein_coding
5	52000001	52050000	0.359695	5	51953184	52003742	50559	+	ENSSSCG00000000580	RECQL	protein_coding
5	51950001	52000000	0.39624	5	51992437	52020816	28380	-	ENSSSCG00000000583	PYROXD1	protein_coding
5	51960001	52010000	0.419126	5	51992437	52020816	28380	-	ENSSSCG00000000583	PYROXD1	protein_coding
5	51970001	52020000	0.432199	5	51992437	52020816	28380	-	ENSSSCG00000000583	PYROXD1	protein_coding
5	51980001	52030000	0.418492	5	51992437	52020816	28380	-	ENSSSCG00000000583	PYROXD1	protein_coding
5	51990001	52040000	0.369049	5	51992437	52020816	28380	-	ENSSSCG00000000583	PYROXD1	protein_coding
5	52000001	52050000	0.359695	5	51992437	52020816	28380	-	ENSSSCG00000000583	PYROXD1	protein_coding
5	51990001	52040000	0.369049	5	52037218	52131789	94572	+	ENSSSCG00000000584	SLCO1A2	protein_coding
5	52000001	52050000	0.359695	5	52037218	52131789	94572	+	ENSSSCG00000000584	SLCO1A2	protein_coding
5	52040001	52090000	0.371205	5	52037218	52131789	94572	+	ENSSSCG00000000584	SLCO1A2	protein_coding
5	52050001	52100000	0.38237	5	52037218	52131789	94572	+	ENSSSCG00000000584	SLCO1A2	protein_coding
5	52060001	52110000	0.466734	5	52037218	52131789	94572	+	ENSSSCG00000000584	SLCO1A2	protein_coding
5	52070001	52120000	0.476638	5	52037218	52131789	94572	+	ENSSSCG00000000584	SLCO1A2	protein_coding
5	52080001	52130000	0.487996	5	52037218	52131789	94572	+	ENSSSCG00000000584	SLCO1A2	protein_coding
5	52090001	52140000	0.502118	5	52037218	52131789	94572	+	ENSSSCG00000000584	SLCO1A2	protein_coding
5	52100001	52150000	0.524682	5	52037218	52131789	94572	+	ENSSSCG00000000584	SLCO1A2	protein_coding
5	52110001	52160000	0.491783	5	52037218	52131789	94572	+	ENSSSCG00000000584	SLCO1A2	protein_coding

5	52120001	52170000	0.4621	5	52037218	52131789	94572	+	ENSSSCG00000000584	SLCO1A2	protein_coding
5	52130001	52180000	0.394237	5	52037218	52131789	94572	+	ENSSSCG00000000584	SLCO1A2	protein_coding
5	52000001	52050000	0.359695	5	52046573	52050446	3874	-	ENSSSCG00000000582	IAPP	protein_coding
5	52040001	52090000	0.371205	5	52046573	52050446	3874	-	ENSSSCG00000000582	IAPP	protein_coding
5	52050001	52100000	0.38237	5	52046573	52050446	3874	-	ENSSSCG00000000582	IAPP	protein_coding
5	52090001	52140000	0.502118	5	52137454	52217448	79995	-	ENSSSCG00000021998	NA	protein_coding
5	52100001	52150000	0.524682	5	52137454	52217448	79995	-	ENSSSCG00000021998	NA	protein_coding
5	52110001	52160000	0.491783	5	52137454	52217448	79995	-	ENSSSCG00000021998	NA	protein_coding
5	52120001	52170000	0.4621	5	52137454	52217448	79995	-	ENSSSCG00000021998	NA	protein_coding
5	52130001	52180000	0.394237	5	52137454	52217448	79995	-	ENSSSCG00000021998	NA	protein_coding
5	52640001	52690000	0.370795	5	52413105	52738829	325725	-	ENSSSCG00000028662	PDE3A	protein_coding
5	52650001	52700000	0.366532	5	52413105	52738829	325725	-	ENSSSCG00000028662	PDE3A	protein_coding
5	52660001	52710000	0.407385	5	52413105	52738829	325725	-	ENSSSCG00000028662	PDE3A	protein_coding
5	52670001	52720000	0.381058	5	52413105	52738829	325725	-	ENSSSCG00000028662	PDE3A	protein_coding
5	52680001	52730000	0.368354	5	52413105	52738829	325725	-	ENSSSCG00000028662	PDE3A	protein_coding
5	52690001	52740000	0.359464	5	52413105	52738829	325725	-	ENSSSCG00000028662	PDE3A	protein_coding
5	52730001	52780000	0.396246	5	52413105	52738829	325725	-	ENSSSCG00000028662	PDE3A	protein_coding
1	90800001	90850000	0.36098	1	90800927	90801030	104	+	ENSSSCG00000031534	U6	snRNA
1	109760001	109810000	0.367548	1	109760159	109760265	107	-	ENSSSCG00000039479	U6	snRNA
1	101710001	101760000	0.353464	1	101679349	101845647	166299	+	ENSSSCG00000051201	NA	lncRNA
1	101720001	101770000	0.364442	1	101679349	101845647	166299	+	ENSSSCG00000051201	NA	lncRNA
1	101730001	101780000	0.363941	1	101679349	101845647	166299	+	ENSSSCG00000051201	NA	lncRNA
1	101740001	101790000	0.356159	1	101679349	101845647	166299	+	ENSSSCG00000051201	NA	lncRNA
1	110330001	110380000	0.355334	1	110145303	110647352	502050	+	ENSSSCG00000046421	NA	lncRNA
1	110510001	110560000	0.369206	1	110145303	110647352	502050	+	ENSSSCG00000046421	NA	lncRNA
1	110520001	110570000	0.381644	1	110145303	110647352	502050	+	ENSSSCG00000046421	NA	lncRNA
1	137030001	137080000	0.36851	1	137076324	137084238	7915	-	ENSSSCG00000050629	NA	lncRNA
1	137040001	137090000	0.352997	1	137076324	137084238	7915	-	ENSSSCG00000050629	NA	lncRNA
1	137820001	137870000	0.3582	1	137801653	137827081	25429	-	ENSSSCG00000039118	NA	lncRNA
1	137820001	137870000	0.3582	1	137824912	137833719	8808	+	ENSSSCG00000041648	NA	lncRNA
1	137830001	137880000	0.371014	1	137824912	137833719	8808	+	ENSSSCG00000041648	NA	lncRNA
1	142510001	142560000	0.364916	1	142554641	142561486	6846	+	ENSSSCG00000046956	NA	lncRNA
1	142520001	142570000	0.375101	1	142554641	142561486	6846	+	ENSSSCG00000046956	NA	lncRNA
1	142530001	142580000	0.383006	1	142554641	142561486	6846	+	ENSSSCG00000046956	NA	lncRNA
1	142540001	142590000	0.361126	1	142554641	142561486	6846	+	ENSSSCG00000046956	NA	lncRNA
1	142550001	142600000	0.369246	1	142554641	142561486	6846	+	ENSSSCG00000046956	NA	lncRNA
1	145220001	145270000	0.35353	1	145239670	145279898	40229	-	ENSSSCG00000050846	NA	lncRNA
1	145230001	145280000	0.357731	1	145239670	145279898	40229	-	ENSSSCG00000050846	NA	lncRNA
1	137000001	137050000	0.352891	1	136704090	137008721	304632	-	ENSSSCG00000044690	FMN1	protein_coding
1	180840001	180890000	0.382814	1	180882260	180894353	12094	-	ENSSSCG00000050763	NA	lncRNA
1	137820001	137870000	0.3582	1	137855658	137880694	25037	+	ENSSSCG00000035223	NA	protein_coding
1	137830001	137880000	0.371014	1	137855658	137880694	25037	+	ENSSSCG00000035223	NA	protein_coding
1	137840001	137890000	0.360164	1	137855658	137880694	25037	+	ENSSSCG00000035223	NA	protein_coding
1	137830001	137880000	0.371014	1	137874491	137997131	122641	-	ENSSSCG00000004813	TTC23	protein_coding
1	137840001	137890000	0.360164	1	137874491	137997131	122641	-	ENSSSCG00000004813	TTC23	protein_coding

1	266340001	266390000	0.356593	1	266381882	266382274	393	-	ENSSSCG00000031440	NA	protein_coding
1	142400001	142450000	0.443227	1	142448689	142454697	6009	+	ENSSSCG00000004835	MAGEL2	protein_coding
1	142410001	142460000	0.428266	1	142448689	142454697	6009	+	ENSSSCG00000004835	MAGEL2	protein_coding
1	142420001	142470000	0.415215	1	142448689	142454697	6009	+	ENSSSCG00000004835	MAGEL2	protein_coding
1	142430001	142480000	0.40251	1	142448689	142454697	6009	+	ENSSSCG00000004835	MAGEL2	protein_coding
1	142440001	142490000	0.410622	1	142448689	142454697	6009	+	ENSSSCG00000004835	MAGEL2	protein_coding
1	142450001	142500000	0.394694	1	142448689	142454697	6009	+	ENSSSCG00000004835	MAGEL2	protein_coding
1	142450001	142500000	0.394694	1	142496735	142500982	4248	-	ENSSSCG00000004836	MKRN3	protein_coding
1	142460001	142510000	0.383118	1	142496735	142500982	4248	-	ENSSSCG00000004836	MKRN3	protein_coding
1	142470001	142520000	0.405516	1	142496735	142500982	4248	-	ENSSSCG00000004836	MKRN3	protein_coding
1	142480001	142530000	0.403759	1	142496735	142500982	4248	-	ENSSSCG00000004836	MKRN3	protein_coding
1	142490001	142540000	0.396008	1	142496735	142500982	4248	-	ENSSSCG00000004836	MKRN3	protein_coding
1	142500001	142550000	0.380527	1	142496735	142500982	4248	-	ENSSSCG00000004836	MKRN3	protein_coding
1	144090001	144140000	0.366389	1	144071859	144315154	243296	+	ENSSSCG00000004849	APBA2	protein_coding
1	144100001	144150000	0.371475	1	144071859	144315154	243296	+	ENSSSCG00000004849	APBA2	protein_coding
1	142370001	142420000	0.462341	1	142412565	142414240	1676	+	ENSSSCG00000004834	NDN	protein_coding
1	142380001	142430000	0.460362	1	142412565	142414240	1676	+	ENSSSCG00000004834	NDN	protein_coding
1	142390001	142440000	0.452038	1	142412565	142414240	1676	+	ENSSSCG00000004834	NDN	protein_coding
1	142400001	142450000	0.443227	1	142412565	142414240	1676	+	ENSSSCG00000004834	NDN	protein_coding
1	142410001	142460000	0.428266	1	142412565	142414240	1676	+	ENSSSCG00000004834	NDN	protein_coding
1	109780001	109830000	0.37808	1	109826052	109826405	354	-	ENSSSCG00000035016	NA	protein_coding
1	109790001	109840000	0.372216	1	109826052	109826405	354	-	ENSSSCG00000035016	NA	protein_coding
1	142620001	142670000	0.361649	1	142619171	142621668	2498	+	ENSSSCG00000047528	NA	protein_coding
1	157780001	157830000	0.358194	1	157820645	157836904	16260	-	ENSSSCG00000004888	SERPINB8	protein_coding
1	157810001	157860000	0.366221	1	157820645	157836904	16260	-	ENSSSCG00000004888	SERPINB8	protein_coding
1	157810001	157860000	0.366221	1	157841510	157866913	25404	-	ENSSSCG00000004889	SERPINB10	protein_coding
1	159100001	159150000	0.36123	1	159128038	159187481	59444	-	ENSSSCG00000004898	TNFRSF11A	protein_coding
1	159110001	159160000	0.368172	1	159128038	159187481	59444	-	ENSSSCG00000004898	TNFRSF11A	protein_coding
1	159120001	159170000	0.35577	1	159128038	159187481	59444	-	ENSSSCG00000004898	TNFRSF11A	protein_coding
1	160640001	160690000	0.356649	1	160629220	160749478	120259	-	ENSSSCG00000036234	NA	protein_coding
1	83090001	83140000	0.375141	1	83026494	83137427	110934	-	ENSSSCG00000004457	DOP1A	protein_coding
1	83100001	83150000	0.370536	1	83026494	83137427	110934	-	ENSSSCG00000004457	DOP1A	protein_coding
1	180830001	180880000	0.428167	1	180878428	180878805	378	+	ENSSSCG00000046029	NA	protein_coding
1	180840001	180890000	0.382814	1	180878428	180878805	378	+	ENSSSCG00000046029	NA	protein_coding
1	83100001	83150000	0.370536	1	83140474	83366841	226368	+	ENSSSCG00000026346	UBE3D	protein_coding
1	90800001	90850000	0.36098	1	90744429	90875118	130690	+	ENSSSCG00000004484	COL12A1	protein_coding
1	90810001	90860000	0.39343	1	90744429	90875118	130690	+	ENSSSCG00000004484	COL12A1	protein_coding
1	99050001	99100000	0.362913	1	99081938	99110876	28939	+	ENSSSCG00000004509	LIPG	protein_coding
1	99060001	99110000	0.375927	1	99081938	99110876	28939	+	ENSSSCG00000004509	LIPG	protein_coding
1	260820001	260870000	0.353452	1	260825454	260837682	12229	-	ENSSSCG00000005508	NA	protein_coding
1	260830001	260880000	0.391904	1	260825454	260837682	12229	-	ENSSSCG00000005508	NA	protein_coding
1	260820001	260870000	0.353452	1	260846562	260868681	22120	-	ENSSSCG00000005507	PSMD5	protein_coding
1	260830001	260880000	0.391904	1	260846562	260868681	22120	-	ENSSSCG00000005507	PSMD5	protein_coding
1	260840001	260890000	0.389799	1	260846562	260868681	22120	-	ENSSSCG00000005507	PSMD5	protein_coding
1	260820001	260870000	0.353452	1	260868877	260897735	28859	+	ENSSSCG00000039972	NA	protein_coding

1	260830001	260880000	0.391904	1	260868877	260897735	28859	+	ENSSSCG00000039972	NA	protein_coding
1	260840001	260890000	0.389799	1	260868877	260897735	28859	+	ENSSSCG00000039972	NA	protein_coding
1	99870001	99920000	0.375907	1	99848184	99917328	69145	-	ENSSSCG00000004516	MBD1	protein_coding
1	180810001	180860000	0.380153	1	180723287	180830243	106957	-	ENSSSCG00000005025	TRIM9	protein_coding
1	180820001	180870000	0.432513	1	180723287	180830243	106957	-	ENSSSCG00000005025	TRIM9	protein_coding
1	180830001	180880000	0.428167	1	180723287	180830243	106957	-	ENSSSCG00000005025	TRIM9	protein_coding
1	180810001	180860000	0.380153	1	180835465	180840389	4925	+	ENSSSCG00000048400	NA	pseudogene
1	180820001	180870000	0.432513	1	180835465	180840389	4925	+	ENSSSCG00000048400	NA	pseudogene
1	180830001	180880000	0.428167	1	180835465	180840389	4925	+	ENSSSCG00000048400	NA	pseudogene
1	180840001	180890000	0.382814	1	180835465	180840389	4925	+	ENSSSCG00000048400	NA	pseudogene
1	265300001	265350000	0.357498	1	265284493	265311417	26925	-	ENSSSCG00000005588	NR5A1	protein_coding
1	265300001	265350000	0.357498	1	265322015	265570887	248873	-	ENSSSCG00000005589	NR6A1	protein_coding
1	265370001	265420000	0.357799	1	265322015	265570887	248873	-	ENSSSCG00000005589	NR6A1	protein_coding
1	265380001	265430000	0.39856	1	265322015	265570887	248873	-	ENSSSCG00000005589	NR6A1	protein_coding
1	265390001	265440000	0.387842	1	265322015	265570887	248873	-	ENSSSCG00000005589	NR6A1	protein_coding
1	265400001	265450000	0.361849	1	265322015	265570887	248873	-	ENSSSCG00000005589	NR6A1	protein_coding
1	265510001	265560000	0.427637	1	265322015	265570887	248873	-	ENSSSCG00000005589	NR6A1	protein_coding
1	265520001	265570000	0.459796	1	265322015	265570887	248873	-	ENSSSCG00000005589	NR6A1	protein_coding
1	265530001	265580000	0.452322	1	265322015	265570887	248873	-	ENSSSCG00000005589	NR6A1	protein_coding
1	265540001	265590000	0.418356	1	265322015	265570887	248873	-	ENSSSCG00000005589	NR6A1	protein_coding
1	265550001	265600000	0.379001	1	265322015	265570887	248873	-	ENSSSCG00000005589	NR6A1	protein_coding
1	265530001	265580000	0.452322	1	265577114	265604502	27389	+	ENSSSCG00000005593	OLFML2A	protein_coding
1	265540001	265590000	0.418356	1	265577114	265604502	27389	+	ENSSSCG00000005593	OLFML2A	protein_coding
1	265550001	265600000	0.379001	1	265577114	265604502	27389	+	ENSSSCG00000005593	OLFML2A	protein_coding
1	265700001	265750000	0.356362	1	265652128	265705895	53768	-	ENSSSCG00000005597	GOLGA1	protein_coding
1	265700001	265750000	0.356362	1	265710770	265867067	156298	-	ENSSSCG00000005598	SCAI	protein_coding
1	265710001	265760000	0.36672	1	265710770	265867067	156298	-	ENSSSCG00000005598	SCAI	protein_coding
1	265720001	265770000	0.356947	1	265710770	265867067	156298	-	ENSSSCG00000005598	SCAI	protein_coding
1	266280001	266330000	0.366187	1	266096193	266346210	250018	-	ENSSSCG00000029169	MAPKAP1	protein_coding
1	266290001	266340000	0.395013	1	266096193	266346210	250018	-	ENSSSCG00000029169	MAPKAP1	protein_coding
1	266300001	266350000	0.423199	1	266096193	266346210	250018	-	ENSSSCG00000029169	MAPKAP1	protein_coding
1	266310001	266360000	0.457021	1	266096193	266346210	250018	-	ENSSSCG00000029169	MAPKAP1	protein_coding
1	266320001	266370000	0.440613	1	266096193	266346210	250018	-	ENSSSCG00000029169	MAPKAP1	protein_coding
1	266330001	266380000	0.374062	1	266096193	266346210	250018	-	ENSSSCG00000029169	MAPKAP1	protein_coding
1	266340001	266390000	0.356593	1	266096193	266346210	250018	-	ENSSSCG00000029169	MAPKAP1	protein_coding
1	266290001	266340000	0.395013	1	266334979	266337484	2506	+	ENSSSCG00000047153	NA	pseudogene
1	266300001	266350000	0.423199	1	266334979	266337484	2506	+	ENSSSCG00000047153	NA	pseudogene
1	266310001	266360000	0.457021	1	266334979	266337484	2506	+	ENSSSCG00000047153	NA	pseudogene
1	266320001	266370000	0.440613	1	266334979	266337484	2506	+	ENSSSCG00000047153	NA	pseudogene
1	266330001	266380000	0.374062	1	266334979	266337484	2506	+	ENSSSCG00000047153	NA	pseudogene
1	266340001	266390000	0.356593	1	266382640	266610646	228007	+	ENSSSCG00000005604	PBX3	protein_coding
1	266530001	266580000	0.361094	1	266382640	266610646	228007	+	ENSSSCG00000005604	PBX3	protein_coding
1	266540001	266590000	0.354595	1	266382640	266610646	228007	+	ENSSSCG00000005604	PBX3	protein_coding
1	266550001	266600000	0.367767	1	266382640	266610646	228007	+	ENSSSCG00000005604	PBX3	protein_coding
1	266560001	266610000	0.358459	1	266382640	266610646	228007	+	ENSSSCG00000005604	PBX3	protein_coding

1	109780001	109830000	0.37808	1	109825438	109827177	1740	+	ENSSSCG00000004572	NA	protein_coding
1	109790001	109840000	0.372216	1	109825438	109827177	1740	+	ENSSSCG00000004572	NA	protein_coding
1	109940001	109990000	0.375192	1	109931264	110104241	172978	+	ENSSSCG00000036431	VPS13C	protein_coding
1	109950001	110000000	0.389572	1	109931264	110104241	172978	+	ENSSSCG00000036431	VPS13C	protein_coding
1	109960001	110010000	0.413984	1	109931264	110104241	172978	+	ENSSSCG00000036431	VPS13C	protein_coding
1	109970001	110020000	0.469688	1	109931264	110104241	172978	+	ENSSSCG00000036431	VPS13C	protein_coding
1	109980001	110030000	0.500338	1	109931264	110104241	172978	+	ENSSSCG00000036431	VPS13C	protein_coding
1	109990001	110040000	0.513187	1	109931264	110104241	172978	+	ENSSSCG00000036431	VPS13C	protein_coding
1	110000001	110050000	0.467584	1	109931264	110104241	172978	+	ENSSSCG00000036431	VPS13C	protein_coding
1	110010001	110060000	0.461957	1	109931264	110104241	172978	+	ENSSSCG00000036431	VPS13C	protein_coding
1	110020001	110070000	0.475873	1	109931264	110104241	172978	+	ENSSSCG00000036431	VPS13C	protein_coding
1	110030001	110080000	0.474686	1	109931264	110104241	172978	+	ENSSSCG00000036431	VPS13C	protein_coding
1	110040001	110090000	0.463719	1	109931264	110104241	172978	+	ENSSSCG00000036431	VPS13C	protein_coding
1	110050001	110100000	0.46247	1	109931264	110104241	172978	+	ENSSSCG00000036431	VPS13C	protein_coding
1	110060001	110110000	0.448908	1	109931264	110104241	172978	+	ENSSSCG00000036431	VPS13C	protein_coding
1	110070001	110120000	0.416114	1	109931264	110104241	172978	+	ENSSSCG00000036431	VPS13C	protein_coding
1	110080001	110130000	0.368952	1	109931264	110104241	172978	+	ENSSSCG00000036431	VPS13C	protein_coding
1	110980001	111030000	0.412009	1	110719190	111477703	758514	+	ENSSSCG00000004576	RORA	protein_coding
1	110990001	111040000	0.458074	1	110719190	111477703	758514	+	ENSSSCG00000004576	RORA	protein_coding
1	111000001	111050000	0.4756	1	110719190	111477703	758514	+	ENSSSCG00000004576	RORA	protein_coding
1	111010001	111060000	0.501781	1	110719190	111477703	758514	+	ENSSSCG00000004576	RORA	protein_coding
1	111020001	111070000	0.520538	1	110719190	111477703	758514	+	ENSSSCG00000004576	RORA	protein_coding
1	111030001	111080000	0.490594	1	110719190	111477703	758514	+	ENSSSCG00000004576	RORA	protein_coding
1	111040001	111090000	0.392556	1	110719190	111477703	758514	+	ENSSSCG00000004576	RORA	protein_coding
1	111050001	111100000	0.363653	1	110719190	111477703	758514	+	ENSSSCG00000004576	RORA	protein_coding
1	111320001	111370000	0.393006	1	110719190	111477703	758514	+	ENSSSCG00000004576	RORA	protein_coding
1	111330001	111380000	0.398043	1	110719190	111477703	758514	+	ENSSSCG00000004576	RORA	protein_coding
1	111340001	111390000	0.386418	1	110719190	111477703	758514	+	ENSSSCG00000004576	RORA	protein_coding
1	111350001	111400000	0.368303	1	110719190	111477703	758514	+	ENSSSCG00000004576	RORA	protein_coding
1	115700001	115750000	0.358336	1	115526738	115734194	207457	-	ENSSSCG00000004602	TEX9	protein_coding
1	115700001	115750000	0.358336	1	115734344	115871086	136743	+	ENSSSCG00000004603	RFX7	protein_coding
1	117580001	117630000	0.371185	1	117166283	117764668	598386	-	ENSSSCG00000004614	UNC13C	protein_coding
1	117590001	117640000	0.524107	1	117166283	117764668	598386	-	ENSSSCG00000004614	UNC13C	protein_coding
1	117600001	117650000	0.512515	1	117166283	117764668	598386	-	ENSSSCG00000004614	UNC13C	protein_coding
1	117610001	117660000	0.46543	1	117166283	117764668	598386	-	ENSSSCG00000004614	UNC13C	protein_coding
1	117620001	117670000	0.385422	1	117166283	117764668	598386	-	ENSSSCG00000004614	UNC13C	protein_coding
1	119190001	119240000	0.353536	1	119134313	119394725	260413	+	ENSSSCG00000004620	MYO5A	protein_coding
1	119200001	119250000	0.368706	1	119134313	119394725	260413	+	ENSSSCG00000004620	MYO5A	protein_coding
1	119210001	119260000	0.354376	1	119134313	119394725	260413	+	ENSSSCG00000004620	MYO5A	protein_coding
1	119220001	119270000	0.385481	1	119134313	119394725	260413	+	ENSSSCG00000004620	MYO5A	protein_coding
1	119230001	119280000	0.354731	1	119134313	119394725	260413	+	ENSSSCG00000004620	MYO5A	protein_coding
1	120280001	120330000	0.352876	1	120138334	120298711	160378	+	ENSSSCG00000032517	DMXL2	protein_coding
1	120290001	120340000	0.392262	1	120138334	120298711	160378	+	ENSSSCG00000032517	DMXL2	protein_coding
1	120280001	120330000	0.352876	1	120322432	120399041	76610	-	ENSSSCG00000004632	GLDN	protein_coding
1	120290001	120340000	0.392262	1	120322432	120399041	76610	-	ENSSSCG00000004632	GLDN	protein_coding

1	120300001	120350000	0.412912	1	120322432	120399041	76610	-	ENSSSCG00000004632	GLDN	protein_coding
1	120310001	120360000	0.423097	1	120322432	120399041	76610	-	ENSSSCG00000004632	GLDN	protein_coding
1	120320001	120370000	0.409268	1	120322432	120399041	76610	-	ENSSSCG00000004632	GLDN	protein_coding
1	120330001	120380000	0.384818	1	120322432	120399041	76610	-	ENSSSCG00000004632	GLDN	protein_coding
1	120340001	120390000	0.364779	1	120322432	120399041	76610	-	ENSSSCG00000004632	GLDN	protein_coding
1	120320001	120370000	0.409268	1	120367667	120695899	328233	+	ENSSSCG00000030168	CYP19A1	protein_coding
1	120330001	120380000	0.384818	1	120367667	120695899	328233	+	ENSSSCG00000030168	CYP19A1	protein_coding
1	120340001	120390000	0.364779	1	120367667	120695899	328233	+	ENSSSCG00000030168	CYP19A1	protein_coding
1	120460001	120510000	0.411645	1	120367667	120695899	328233	+	ENSSSCG00000030168	CYP19A1	protein_coding
1	120470001	120520000	0.422759	1	120367667	120695899	328233	+	ENSSSCG00000030168	CYP19A1	protein_coding
6	380001	430000	0.357319	6	412582	412662	81	-	ENSSSCG00000022137	NA	snoRNA
6	390001	440000	0.369735	6	412582	412662	81	-	ENSSSCG00000022137	NA	snoRNA
6	105590001	105640000	0.409322	6	105634666	105634772	107	-	ENSSSCG00000035688	U6	snRNA
6	105600001	105650000	0.412298	6	105634666	105634772	107	-	ENSSSCG00000035688	U6	snRNA
6	105610001	105660000	0.409878	6	105634666	105634772	107	-	ENSSSCG00000035688	U6	snRNA
6	105620001	105670000	0.356084	6	105634666	105634772	107	-	ENSSSCG00000035688	U6	snRNA
6	113310001	113360000	0.36675	6	113332743	113332849	107	+	ENSSSCG00000019533	U6	snRNA
6	85500001	85550000	0.355595	6	85533589	85538276	4688	+	ENSSSCG00000041401	NA	lncRNA
6	85500001	85550000	0.355595	6	85535474	85535677	204	+	ENSSSCG00000034185	SNORA73	snoRNA
6	85500001	85550000	0.355595	6	85534254	85534457	204	+	ENSSSCG00000037630	SNORA73	snoRNA
6	380001	430000	0.357319	6	394764	400206	5443	-	ENSSSCG00000049264	NA	lncRNA
6	390001	440000	0.369735	6	394764	400206	5443	-	ENSSSCG00000049264	NA	lncRNA
6	160760001	160810000	0.352902	6	160774741	160774870	130	+	ENSSSCG00000051480	U2	snRNA
6	160770001	160820000	0.360965	6	160774741	160774870	130	+	ENSSSCG00000051480	U2	snRNA
6	161200001	161250000	0.37045	6	161246086	161246189	104	-	ENSSSCG00000018668	U6	snRNA
6	161210001	161260000	0.357402	6	161246086	161246189	104	-	ENSSSCG00000018668	U6	snRNA
6	85240001	85290000	0.383019	6	85260043	85260206	164	-	ENSSSCG00000033799	U1	snRNA
6	85250001	85300000	0.388873	6	85260043	85260206	164	-	ENSSSCG00000033799	U1	snRNA
6	161630001	161680000	0.36034	6	161622795	161678402	55608	+	ENSSSCG00000048380	NA	lncRNA
6	159910001	159960000	0.371798	6	159874026	160074413	200388	-	ENSSSCG00000003863	ZFYVE9	protein_coding
6	160380001	160430000	0.357525	6	160299123	160388763	89641	+	ENSSSCG00000003870	NRDC	protein_coding
6	160380001	160430000	0.357525	6	160388416	160566370	177955	-	ENSSSCG00000021292	OSBPL9	protein_coding
6	160390001	160440000	0.375395	6	160388416	160566370	177955	-	ENSSSCG00000021292	OSBPL9	protein_coding
6	160400001	160450000	0.403956	6	160388416	160566370	177955	-	ENSSSCG00000021292	OSBPL9	protein_coding
6	160410001	160460000	0.390526	6	160388416	160566370	177955	-	ENSSSCG00000021292	OSBPL9	protein_coding
6	85240001	85290000	0.383019	6	85235390	85272333	36944	-	ENSSSCG00000031788	PTAFR	protein_coding
6	85250001	85300000	0.388873	6	85235390	85272333	36944	-	ENSSSCG00000031788	PTAFR	protein_coding
6	85270001	85320000	0.374135	6	85235390	85272333	36944	-	ENSSSCG00000031788	PTAFR	protein_coding
6	85240001	85290000	0.383019	6	85278151	85307608	29458	-	ENSSSCG00000038907	DNAJC8	protein_coding
6	85250001	85300000	0.388873	6	85278151	85307608	29458	-	ENSSSCG00000038907	DNAJC8	protein_coding
6	85270001	85320000	0.374135	6	85278151	85307608	29458	-	ENSSSCG00000038907	DNAJC8	protein_coding
6	85280001	85330000	0.414548	6	85278151	85307608	29458	-	ENSSSCG00000038907	DNAJC8	protein_coding
6	85270001	85320000	0.374135	6	85307189	85321350	14162	+	ENSSSCG00000027072	ATP5IF1	protein_coding
6	85280001	85330000	0.414548	6	85307189	85321350	14162	+	ENSSSCG00000027072	ATP5IF1	protein_coding
6	85280001	85330000	0.414548	6	85324228	85348044	23817	+	ENSSSCG00000029438	SESN2	protein_coding

6	85500001	85550000	0.355595	6	85412180	85528746	116567	+	ENSSSCG00000028973	PHACTR4	protein_coding
6	85500001	85550000	0.355595	6	85546024	85564877	18854	+	ENSSSCG00000029097	RCC1	protein_coding
6	160760001	160810000	0.352902	6	160653890	160801288	147399	+	ENSSSCG00000003872	EPS15	protein_coding
6	160770001	160820000	0.360965	6	160653890	160801288	147399	+	ENSSSCG00000003872	EPS15	protein_coding
6	160800001	160850000	0.355078	6	160653890	160801288	147399	+	ENSSSCG00000003872	EPS15	protein_coding
6	160760001	160810000	0.352902	6	160806629	160854490	47862	+	ENSSSCG00000003875	TTC39A	protein_coding
6	160770001	160820000	0.360965	6	160806629	160854490	47862	+	ENSSSCG00000003875	TTC39A	protein_coding
6	160800001	160850000	0.355078	6	160806629	160854490	47862	+	ENSSSCG00000003875	TTC39A	protein_coding
6	160840001	160890000	0.362487	6	160806629	160854490	47862	+	ENSSSCG00000003875	TTC39A	protein_coding
6	160850001	160900000	0.367774	6	160806629	160854490	47862	+	ENSSSCG00000003875	TTC39A	protein_coding
6	160840001	160890000	0.362487	6	160875766	160911630	35865	-	ENSSSCG00000031172	RNF11	protein_coding
6	160850001	160900000	0.367774	6	160875766	160911630	35865	-	ENSSSCG00000031172	RNF11	protein_coding
6	160860001	160910000	0.384027	6	160875766	160911630	35865	-	ENSSSCG00000031172	RNF11	protein_coding
6	160940001	160990000	0.366097	6	160986081	161031140	45060	-	ENSSSCG00000031188	C1orf185	protein_coding
6	160950001	161000000	0.41606	6	160986081	161031140	45060	-	ENSSSCG00000031188	C1orf185	protein_coding
6	160960001	161010000	0.446377	6	160986081	161031140	45060	-	ENSSSCG00000031188	C1orf185	protein_coding
6	160970001	161020000	0.463075	6	160986081	161031140	45060	-	ENSSSCG00000031188	C1orf185	protein_coding
6	160980001	161030000	0.446067	6	160986081	161031140	45060	-	ENSSSCG00000031188	C1orf185	protein_coding
6	160990001	161040000	0.410612	6	160986081	161031140	45060	-	ENSSSCG00000031188	C1orf185	protein_coding
6	161000001	161050000	0.410437	6	160986081	161031140	45060	-	ENSSSCG00000031188	C1orf185	protein_coding
6	161010001	161060000	0.377094	6	160986081	161031140	45060	-	ENSSSCG00000031188	C1orf185	protein_coding
6	161200001	161250000	0.37045	6	161166996	161603725	436730	+	ENSSSCG00000003877	FAF1	protein_coding
6	161210001	161260000	0.357402	6	161166996	161603725	436730	+	ENSSSCG00000003877	FAF1	protein_coding
6	161850001	161900000	0.358787	6	161786352	161928910	142559	-	ENSSSCG00000037291	ELAVL4	protein_coding
6	161860001	161910000	0.352789	6	161786352	161928910	142559	-	ENSSSCG00000037291	ELAVL4	protein_coding
6	88580001	88630000	0.385761	6	88585290	88606910	21621	+	ENSSSCG00000032563	TMEM39B	protein_coding
6	88590001	88640000	0.387554	6	88585290	88606910	21621	+	ENSSSCG00000032563	TMEM39B	protein_coding
6	88600001	88650000	0.416657	6	88585290	88606910	21621	+	ENSSSCG00000032563	TMEM39B	protein_coding
6	88580001	88630000	0.385761	6	88611462	88656278	44817	+	ENSSSCG00000034017	KPNA6	protein_coding
6	88590001	88640000	0.387554	6	88611462	88656278	44817	+	ENSSSCG00000034017	KPNA6	protein_coding
6	88600001	88650000	0.416657	6	88611462	88656278	44817	+	ENSSSCG00000034017	KPNA6	protein_coding
6	88610001	88660000	0.406731	6	88611462	88656278	44817	+	ENSSSCG00000034017	KPNA6	protein_coding
6	88620001	88670000	0.395336	6	88611462	88656278	44817	+	ENSSSCG00000034017	KPNA6	protein_coding
6	88630001	88680000	0.39436	6	88611462	88656278	44817	+	ENSSSCG00000034017	KPNA6	protein_coding
6	88640001	88690000	0.393165	6	88611462	88656278	44817	+	ENSSSCG00000034017	KPNA6	protein_coding
6	88650001	88700000	0.375951	6	88611462	88656278	44817	+	ENSSSCG00000034017	KPNA6	protein_coding
6	88610001	88660000	0.406731	6	88659708	88676354	16647	+	ENSSSCG00000003617	TXLNA	protein_coding
6	88620001	88670000	0.395336	6	88659708	88676354	16647	+	ENSSSCG00000003617	TXLNA	protein_coding
6	88630001	88680000	0.39436	6	88659708	88676354	16647	+	ENSSSCG00000003617	TXLNA	protein_coding
6	88640001	88690000	0.393165	6	88659708	88676354	16647	+	ENSSSCG00000003617	TXLNA	protein_coding
6	88650001	88700000	0.375951	6	88659708	88676354	16647	+	ENSSSCG00000003617	TXLNA	protein_coding
6	88630001	88680000	0.39436	6	88678230	88682820	4591	+	ENSSSCG00000031514	CCDC28B	protein_coding
6	88640001	88690000	0.393165	6	88678230	88682820	4591	+	ENSSSCG00000031514	CCDC28B	protein_coding
6	88650001	88700000	0.375951	6	88678230	88682820	4591	+	ENSSSCG00000031514	CCDC28B	protein_coding
6	88640001	88690000	0.393165	6	88683119	88685796	2678	+	ENSSSCG00000003610	IQCC	protein_coding

6	88650001	88700000	0.375951	6	88683119	88685796	2678	+	ENSSSCG00000003610	IQCC	protein_coding
6	88640001	88690000	0.393165	6	88686295	88691866	5572	+	ENSSSCG00000022014	DCDC2B	protein_coding
6	88650001	88700000	0.375951	6	88686295	88691866	5572	+	ENSSSCG00000022014	DCDC2B	protein_coding
6	88650001	88700000	0.375951	6	88691869	88696057	4189	-	ENSSSCG00000037312	NA	protein_coding
6	88650001	88700000	0.375951	6	88695947	88705078	9132	+	ENSSSCG00000003612	EIF3I	protein_coding
6	162070001	162120000	0.364956	6	161952983	163216257	1263275	+	ENSSSCG00000031300	NA	protein_coding
6	162080001	162130000	0.420129	6	161952983	163216257	1263275	+	ENSSSCG00000031300	NA	protein_coding
6	162090001	162140000	0.447745	6	161952983	163216257	1263275	+	ENSSSCG00000031300	NA	protein_coding
6	162100001	162150000	0.468849	6	161952983	163216257	1263275	+	ENSSSCG00000031300	NA	protein_coding
6	162110001	162160000	0.478002	6	161952983	163216257	1263275	+	ENSSSCG00000031300	NA	protein_coding
6	162120001	162170000	0.463896	6	161952983	163216257	1263275	+	ENSSSCG00000031300	NA	protein_coding
6	162130001	162180000	0.480132	6	161952983	163216257	1263275	+	ENSSSCG00000031300	NA	protein_coding
6	162140001	162190000	0.436038	6	161952983	163216257	1263275	+	ENSSSCG00000031300	NA	protein_coding
6	162150001	162200000	0.387525	6	161952983	163216257	1263275	+	ENSSSCG00000031300	NA	protein_coding
6	162160001	162210000	0.361887	6	161952983	163216257	1263275	+	ENSSSCG00000031300	NA	protein_coding
6	162240001	162290000	0.375166	6	161952983	163216257	1263275	+	ENSSSCG00000031300	NA	protein_coding
6	162250001	162300000	0.451276	6	161952983	163216257	1263275	+	ENSSSCG00000031300	NA	protein_coding
6	162260001	162310000	0.511	6	161952983	163216257	1263275	+	ENSSSCG00000031300	NA	protein_coding
6	162270001	162320000	0.508933	6	161952983	163216257	1263275	+	ENSSSCG00000031300	NA	protein_coding
6	162280001	162330000	0.478215	6	161952983	163216257	1263275	+	ENSSSCG00000031300	NA	protein_coding
6	162290001	162340000	0.44243	6	161952983	163216257	1263275	+	ENSSSCG00000031300	NA	protein_coding
6	162300001	162350000	0.385918	6	161952983	163216257	1263275	+	ENSSSCG00000031300	NA	protein_coding
6	162560001	162610000	0.374958	6	161952983	163216257	1263275	+	ENSSSCG00000031300	NA	protein_coding
6	163110001	163160000	0.393167	6	161952983	163216257	1263275	+	ENSSSCG00000031300	NA	protein_coding
6	163120001	163170000	0.429845	6	161952983	163216257	1263275	+	ENSSSCG00000031300	NA	protein_coding
6	163130001	163180000	0.413972	6	161952983	163216257	1263275	+	ENSSSCG00000031300	NA	protein_coding
6	162240001	162290000	0.375166	6	162263574	162267392	3819	-	ENSSSCG00000050179	NA	protein_coding
6	162250001	162300000	0.451276	6	162263574	162267392	3819	-	ENSSSCG00000050179	NA	protein_coding
6	162260001	162310000	0.511	6	162263574	162267392	3819	-	ENSSSCG00000050179	NA	protein_coding
6	88910001	88960000	0.370272	6	88922795	88960600	37806	+	ENSSSCG00000021457	ZBTB8A	protein_coding
6	89920001	89970000	0.36791	6	89878021	90048944	170924	-	ENSSSCG00000003622	NA	protein_coding
6	89930001	89980000	0.376871	6	89878021	90048944	170924	-	ENSSSCG00000003622	NA	protein_coding
6	89940001	89990000	0.357988	6	89878021	90048944	170924	-	ENSSSCG00000003622	NA	protein_coding
6	91610001	91660000	0.355313	6	91455778	91623819	168042	+	ENSSSCG00000003627	ZMYM4	protein_coding
6	91610001	91660000	0.355313	6	91605502	91747773	142272	-	ENSSSCG00000037121	KIAA0319L	protein_coding
6	380001	430000	0.357319	6	393685	394344	660	-	ENSSSCG00000051285	NA	protein_coding
6	390001	440000	0.369735	6	393685	394344	660	-	ENSSSCG00000051285	NA	protein_coding
6	380001	430000	0.357319	6	404075	406099	2025	-	ENSSSCG00000031104	NA	protein_coding
6	390001	440000	0.369735	6	404075	406099	2025	-	ENSSSCG00000031104	NA	protein_coding
6	380001	430000	0.357319	6	410309	413336	3028	-	ENSSSCG00000024974	NA	protein_coding
6	390001	440000	0.369735	6	410309	413336	3028	-	ENSSSCG00000024974	NA	protein_coding
6	380001	430000	0.357319	6	414930	439212	24283	-	ENSSSCG00000024756	SPG7	protein_coding
6	390001	440000	0.369735	6	414930	439212	24283	-	ENSSSCG00000024756	SPG7	protein_coding
6	93630001	93680000	0.36423	6	93619453	93645960	26508	-	ENSSSCG00000031631	GNL2	protein_coding
6	93630001	93680000	0.36423	6	93665138	93685197	20060	-	ENSSSCG00000032240	RSPO1	protein_coding

6	93760001	93810000	0.364014	6	93750687	93765483	14797	+	ENSSSCG00000032909	CDCA8	protein_coding
6	93760001	93810000	0.364014	6	93765128	93803894	38767	-	ENSSSCG00000038675	EPHA10	protein_coding
6	93780001	93830000	0.358095	6	93765128	93803894	38767	-	ENSSSCG00000038675	EPHA10	protein_coding
6	93790001	93840000	0.361927	6	93765128	93803894	38767	-	ENSSSCG00000038675	EPHA10	protein_coding
6	93800001	93850000	0.385665	6	93765128	93803894	38767	-	ENSSSCG00000038675	EPHA10	protein_coding
6	93780001	93830000	0.358095	6	93821593	93829456	7864	+	ENSSSCG00000032960	MANEAL	protein_coding
6	93790001	93840000	0.361927	6	93821593	93829456	7864	+	ENSSSCG00000032960	MANEAL	protein_coding
6	93800001	93850000	0.385665	6	93821593	93829456	7864	+	ENSSSCG00000032960	MANEAL	protein_coding
6	93810001	93860000	0.43124	6	93821593	93829456	7864	+	ENSSSCG00000032960	MANEAL	protein_coding
6	93820001	93870000	0.4743	6	93821593	93829456	7864	+	ENSSSCG00000032960	MANEAL	protein_coding
6	93780001	93830000	0.358095	6	93829319	93833667	4349	-	ENSSSCG00000040425	YRDC	protein_coding
6	93790001	93840000	0.361927	6	93829319	93833667	4349	-	ENSSSCG00000040425	YRDC	protein_coding
6	93800001	93850000	0.385665	6	93829319	93833667	4349	-	ENSSSCG00000040425	YRDC	protein_coding
6	93810001	93860000	0.43124	6	93829319	93833667	4349	-	ENSSSCG00000040425	YRDC	protein_coding
6	93820001	93870000	0.4743	6	93829319	93833667	4349	-	ENSSSCG00000040425	YRDC	protein_coding
6	93830001	93880000	0.485704	6	93829319	93833667	4349	-	ENSSSCG00000040425	YRDC	protein_coding
6	93790001	93840000	0.361927	6	93833759	93834920	1162	+	ENSSSCG00000038888	C1orf122	protein_coding
6	93800001	93850000	0.385665	6	93833759	93834920	1162	+	ENSSSCG00000038888	C1orf122	protein_coding
6	93810001	93860000	0.43124	6	93833759	93834920	1162	+	ENSSSCG00000038888	C1orf122	protein_coding
6	93820001	93870000	0.4743	6	93833759	93834920	1162	+	ENSSSCG00000038888	C1orf122	protein_coding
6	93830001	93880000	0.485704	6	93833759	93834920	1162	+	ENSSSCG00000038888	C1orf122	protein_coding
6	93790001	93840000	0.361927	6	93839174	93884405	45232	-	ENSSSCG00000037516	MTF1	protein_coding
6	93800001	93850000	0.385665	6	93839174	93884405	45232	-	ENSSSCG00000037516	MTF1	protein_coding
6	93810001	93860000	0.43124	6	93839174	93884405	45232	-	ENSSSCG00000037516	MTF1	protein_coding
6	93820001	93870000	0.4743	6	93839174	93884405	45232	-	ENSSSCG00000037516	MTF1	protein_coding
6	93830001	93880000	0.485704	6	93839174	93884405	45232	-	ENSSSCG00000037516	MTF1	protein_coding
6	93840001	93890000	0.456236	6	93839174	93884405	45232	-	ENSSSCG00000037516	MTF1	protein_coding
6	93850001	93900000	0.462088	6	93839174	93884405	45232	-	ENSSSCG00000037516	MTF1	protein_coding
6	93860001	93910000	0.457376	6	93839174	93884405	45232	-	ENSSSCG00000037516	MTF1	protein_coding
6	93870001	93920000	0.43889	6	93839174	93884405	45232	-	ENSSSCG00000037516	MTF1	protein_coding
6	93880001	93930000	0.390951	6	93839174	93884405	45232	-	ENSSSCG00000037516	MTF1	protein_coding
6	93840001	93890000	0.456236	6	93885460	93944333	58874	-	ENSSSCG00000003642	INPP5B	protein_coding
6	93850001	93900000	0.462088	6	93885460	93944333	58874	-	ENSSSCG00000003642	INPP5B	protein_coding
6	93860001	93910000	0.457376	6	93885460	93944333	58874	-	ENSSSCG00000003642	INPP5B	protein_coding
6	93870001	93920000	0.43889	6	93885460	93944333	58874	-	ENSSSCG00000003642	INPP5B	protein_coding
6	93880001	93930000	0.390951	6	93885460	93944333	58874	-	ENSSSCG00000003642	INPP5B	protein_coding
6	105570001	105620000	0.370217	6	105505479	105600687	95209	+	ENSSSCG00000031942	YES1	protein_coding
6	105580001	105630000	0.404587	6	105505479	105600687	95209	+	ENSSSCG00000031942	YES1	protein_coding
6	105590001	105640000	0.409322	6	105505479	105600687	95209	+	ENSSSCG00000031942	YES1	protein_coding
6	105600001	105650000	0.412298	6	105505479	105600687	95209	+	ENSSSCG00000031942	YES1	protein_coding
6	105570001	105620000	0.370217	6	105613273	105645113	31841	+	ENSSSCG00000027169	ENOSF1	protein_coding
6	105580001	105630000	0.404587	6	105613273	105645113	31841	+	ENSSSCG00000027169	ENOSF1	protein_coding
6	105590001	105640000	0.409322	6	105613273	105645113	31841	+	ENSSSCG00000027169	ENOSF1	protein_coding
6	105600001	105650000	0.412298	6	105613273	105645113	31841	+	ENSSSCG00000027169	ENOSF1	protein_coding
6	105610001	105660000	0.409878	6	105613273	105645113	31841	+	ENSSSCG00000027169	ENOSF1	protein_coding

6	105620001	105670000	0.356084	6	105613273	105645113	31841	+	ENSSSCG00000027169	ENOSF1	protein_coding
6	105600001	105650000	0.412298	6	105640031	105660601	20571	-	ENSSSCG00000040678	TYMS	protein_coding
6	105610001	105660000	0.409878	6	105640031	105660601	20571	-	ENSSSCG00000040678	TYMS	protein_coding
6	105620001	105670000	0.356084	6	105640031	105660601	20571	-	ENSSSCG00000040678	TYMS	protein_coding
6	105620001	105670000	0.356084	6	105667288	105707335	40048	-	ENSSSCG00000039398	CLUL1	protein_coding
6	106190001	106240000	0.362077	6	106163903	106314944	151042	-	ENSSSCG00000021893	ROCK1	protein_coding
6	106200001	106250000	0.353944	6	106163903	106314944	151042	-	ENSSSCG00000021893	ROCK1	protein_coding
6	106590001	106640000	0.401067	6	106419943	106691646	271704	+	ENSSSCG00000003699	GREB1L	protein_coding
6	106600001	106650000	0.404416	6	106419943	106691646	271704	+	ENSSSCG00000003699	GREB1L	protein_coding
6	106620001	106670000	0.355458	6	106419943	106691646	271704	+	ENSSSCG00000003699	GREB1L	protein_coding
3	58630001	58680000	0.366994	3	58636448	58636582	135	-	ENSSSCG00000019715	NA	snoRNA
3	58050001	58100000	0.354989	3	58037766	58096721	58956	-	ENSSSCG00000035478	RMND5A	protein_coding
3	71410001	71460000	0.354363	3	71452538	71499500	46963	+	ENSSSCG00000038394	NA	lncRNA
3	71450001	71500000	0.372002	3	71452538	71499500	46963	+	ENSSSCG00000038394	NA	lncRNA
3	67810001	67860000	0.390328	3	67820941	67821076	136	-	ENSSSCG00000036714	NA	snoRNA
3	67820001	67870000	0.432033	3	67820941	67821076	136	-	ENSSSCG00000036714	NA	snoRNA
3	37300001	37350000	0.359521	3	37282882	37310589	27708	-	ENSSSCG00000046716	NA	lncRNA
3	38020001	38070000	0.357969	3	38027673	38036108	8436	+	ENSSSCG00000046837	NA	lncRNA
3	38020001	38070000	0.357969	3	38027723	38031201	3479	-	ENSSSCG00000050892	NA	lncRNA
3	39180001	39230000	0.367204	3	39177778	39180788	3011	-	ENSSSCG00000041682	NA	lncRNA
3	39220001	39270000	0.37913	3	39261903	39265455	3553	-	ENSSSCG00000036096	NA	lncRNA
3	39230001	39280000	0.354968	3	39261903	39265455	3553	-	ENSSSCG00000036096	NA	lncRNA
3	46770001	46820000	0.491156	3	46815839	46818825	2987	-	ENSSSCG00000049775	NA	processed_pseudogene
3	46780001	46830000	0.499089	3	46815839	46818825	2987	-	ENSSSCG00000049775	NA	processed_pseudogene
3	46790001	46840000	0.44138	3	46815839	46818825	2987	-	ENSSSCG00000049775	NA	processed_pseudogene
3	46800001	46850000	0.38324	3	46815839	46818825	2987	-	ENSSSCG00000049775	NA	processed_pseudogene
3	46690001	46740000	0.419121	3	46729621	46733323	3703	-	ENSSSCG00000047838	NA	lncRNA
3	46700001	46750000	0.490432	3	46729621	46733323	3703	-	ENSSSCG00000047838	NA	lncRNA
3	46710001	46760000	0.496001	3	46729621	46733323	3703	-	ENSSSCG00000047838	NA	lncRNA
3	46720001	46770000	0.489808	3	46729621	46733323	3703	-	ENSSSCG00000047838	NA	lncRNA
3	46730001	46780000	0.472526	3	46729621	46733323	3703	-	ENSSSCG00000047838	NA	lncRNA
3	58430001	58480000	0.387327	3	58435510	58558939	123430	+	ENSSSCG00000023710	REEP1	protein_coding
3	58440001	58490000	0.362592	3	58435510	58558939	123430	+	ENSSSCG00000023710	REEP1	protein_coding
3	60190001	60240000	0.410255	3	60234416	60238965	4550	-	ENSSSCG00000051308	NA	lncRNA
3	60200001	60250000	0.395402	3	60234416	60238965	4550	-	ENSSSCG00000051308	NA	lncRNA
3	60210001	60260000	0.387769	3	60234416	60238965	4550	-	ENSSSCG00000051308	NA	lncRNA
3	71410001	71460000	0.354363	3	71455036	71460208	5173	+	ENSSSCG00000045542	NA	lncRNA
3	71450001	71500000	0.372002	3	71455036	71460208	5173	+	ENSSSCG00000045542	NA	lncRNA
3	72340001	72390000	0.39049	3	72324859	72359204	34346	+	ENSSSCG00000041317	NA	lncRNA
3	72350001	72400000	0.394626	3	72324859	72359204	34346	+	ENSSSCG00000041317	NA	lncRNA
3	74210001	74260000	0.370637	3	74232219	74300092	67874	+	ENSSSCG00000047894	NA	lncRNA
3	74220001	74270000	0.402834	3	74232219	74300092	67874	+	ENSSSCG00000047894	NA	lncRNA
3	74230001	74280000	0.401823	3	74232219	74300092	67874	+	ENSSSCG00000047894	NA	lncRNA
3	74240001	74290000	0.40118	3	74232219	74300092	67874	+	ENSSSCG00000047894	NA	lncRNA
3	58630001	58680000	0.366994	3	58575183	58633749	58567	+	ENSSSCG00000024564	IMMT	protein_coding

3	46760001	46810000	0.486754	3	46809045	46809145	101	+	ENSSSCG00000018129	U6	snRNA
3	46770001	46820000	0.491156	3	46809045	46809145	101	+	ENSSSCG00000018129	U6	snRNA
3	46780001	46830000	0.499089	3	46809045	46809145	101	+	ENSSSCG00000018129	U6	snRNA
3	46790001	46840000	0.44138	3	46809045	46809145	101	+	ENSSSCG00000018129	U6	snRNA
3	46800001	46850000	0.38324	3	46809045	46809145	101	+	ENSSSCG00000018129	U6	snRNA
3	58630001	58680000	0.366994	3	58628614	58670788	42175	-	ENSSSCG00000008225	PTCD3	protein_coding
3	58640001	58690000	0.380878	3	58628614	58670788	42175	-	ENSSSCG00000008225	PTCD3	protein_coding
3	58650001	58700000	0.373984	3	58628614	58670788	42175	-	ENSSSCG00000008225	PTCD3	protein_coding
3	41970001	42020000	0.368384	3	42018978	42019109	132	-	ENSSSCG00000034789	NA	snoRNA
3	41980001	42030000	0.367684	3	42018978	42019109	132	-	ENSSSCG00000034789	NA	snoRNA
3	99750001	99800000	0.366631	3	99793401	99824533	31133	-	ENSSSCG00000045020	NA	lncRNA
3	74210001	74260000	0.370637	3	74216042	74216140	99	-	ENSSSCG00000019420	SNORD16	snoRNA
3	58630001	58680000	0.366994	3	58667971	58750421	82451	+	ENSSSCG00000008226	POLR1A	protein_coding
3	58640001	58690000	0.380878	3	58667971	58750421	82451	+	ENSSSCG00000008226	POLR1A	protein_coding
3	58650001	58700000	0.373984	3	58667971	58750421	82451	+	ENSSSCG00000008226	POLR1A	protein_coding
3	72340001	72390000	0.39049	3	72378648	72380362	1715	-	ENSSSCG00000027224	PCBP1	protein_coding
3	72350001	72400000	0.394626	3	72378648	72380362	1715	-	ENSSSCG00000027224	PCBP1	protein_coding
3	72360001	72410000	0.369679	3	72378648	72380362	1715	-	ENSSSCG00000027224	PCBP1	protein_coding
3	60160001	60210000	0.370371	3	60141884	60195051	53168	+	ENSSSCG00000008247	SUCLG1	protein_coding
3	60170001	60220000	0.39753	3	60141884	60195051	53168	+	ENSSSCG00000008247	SUCLG1	protein_coding
3	60180001	60230000	0.401783	3	60141884	60195051	53168	+	ENSSSCG00000008247	SUCLG1	protein_coding
3	60190001	60240000	0.410255	3	60141884	60195051	53168	+	ENSSSCG00000008247	SUCLG1	protein_coding
3	67810001	67860000	0.390328	3	67839411	68021728	182318	+	ENSSSCG00000036008	TACR1	protein_coding
3	67820001	67870000	0.432033	3	67839411	68021728	182318	+	ENSSSCG00000036008	TACR1	protein_coding
3	67830001	67880000	0.436894	3	67839411	68021728	182318	+	ENSSSCG00000036008	TACR1	protein_coding
3	67840001	67890000	0.409546	3	67839411	68021728	182318	+	ENSSSCG00000036008	TACR1	protein_coding
3	70010001	70060000	0.355915	3	69939173	70546702	607530	+	ENSSSCG00000008310	EXOC6B	protein_coding
3	70020001	70070000	0.353379	3	69939173	70546702	607530	+	ENSSSCG00000008310	EXOC6B	protein_coding
3	71220001	71270000	0.390111	3	71020141	71246967	226827	-	ENSSSCG00000008312	DYSF	protein_coding
3	71230001	71280000	0.417766	3	71020141	71246967	226827	-	ENSSSCG00000008312	DYSF	protein_coding
3	71240001	71290000	0.442681	3	71020141	71246967	226827	-	ENSSSCG00000008312	DYSF	protein_coding
3	71220001	71270000	0.390111	3	71265898	71424230	158333	-	ENSSSCG00000022708	ZNF638	protein_coding
3	71230001	71280000	0.417766	3	71265898	71424230	158333	-	ENSSSCG00000022708	ZNF638	protein_coding
3	71240001	71290000	0.442681	3	71265898	71424230	158333	-	ENSSSCG00000022708	ZNF638	protein_coding
3	71250001	71300000	0.466346	3	71265898	71424230	158333	-	ENSSSCG00000022708	ZNF638	protein_coding
3	71260001	71310000	0.458217	3	71265898	71424230	158333	-	ENSSSCG00000022708	ZNF638	protein_coding
3	71270001	71320000	0.46347	3	71265898	71424230	158333	-	ENSSSCG00000022708	ZNF638	protein_coding
3	71280001	71330000	0.502936	3	71265898	71424230	158333	-	ENSSSCG00000022708	ZNF638	protein_coding
3	71290001	71340000	0.504214	3	71265898	71424230	158333	-	ENSSSCG00000022708	ZNF638	protein_coding
3	71300001	71350000	0.499293	3	71265898	71424230	158333	-	ENSSSCG00000022708	ZNF638	protein_coding
3	71310001	71360000	0.494376	3	71265898	71424230	158333	-	ENSSSCG00000022708	ZNF638	protein_coding
3	71320001	71370000	0.475491	3	71265898	71424230	158333	-	ENSSSCG00000022708	ZNF638	protein_coding
3	71330001	71380000	0.446191	3	71265898	71424230	158333	-	ENSSSCG00000022708	ZNF638	protein_coding
3	71340001	71390000	0.429958	3	71265898	71424230	158333	-	ENSSSCG00000022708	ZNF638	protein_coding
3	71350001	71400000	0.429415	3	71265898	71424230	158333	-	ENSSSCG00000022708	ZNF638	protein_coding

3	71360001	71410000	0.418348	3	71265898	71424230	158333	-	ENSSSCG00000022708	ZNF638	protein_coding
3	71370001	71420000	0.417195	3	71265898	71424230	158333	-	ENSSSCG00000022708	ZNF638	protein_coding
3	71380001	71430000	0.39648	3	71265898	71424230	158333	-	ENSSSCG00000022708	ZNF638	protein_coding
3	71390001	71440000	0.38703	3	71265898	71424230	158333	-	ENSSSCG00000022708	ZNF638	protein_coding
3	71400001	71450000	0.369181	3	71265898	71424230	158333	-	ENSSSCG00000022708	ZNF638	protein_coding
3	71410001	71460000	0.354363	3	71265898	71424230	158333	-	ENSSSCG00000022708	ZNF638	protein_coding
3	72340001	72390000	0.39049	3	72381330	72494507	113178	+	ENSSSCG00000033099	ASPRV1	protein_coding
3	72350001	72400000	0.394626	3	72381330	72494507	113178	+	ENSSSCG00000033099	ASPRV1	protein_coding
3	72360001	72410000	0.369679	3	72381330	72494507	113178	+	ENSSSCG00000033099	ASPRV1	protein_coding
3	72430001	72480000	0.364196	3	72381330	72494507	113178	+	ENSSSCG00000033099	ASPRV1	protein_coding
3	72440001	72490000	0.366923	3	72381330	72494507	113178	+	ENSSSCG00000033099	ASPRV1	protein_coding
3	72450001	72500000	0.363793	3	72381330	72494507	113178	+	ENSSSCG00000033099	ASPRV1	protein_coding
3	72460001	72510000	0.376377	3	72381330	72494507	113178	+	ENSSSCG00000033099	ASPRV1	protein_coding
3	76540001	76590000	0.369671	3	76523589	76649650	126062	+	ENSSSCG00000008357	SPRED2	protein_coding
3	76550001	76600000	0.374407	3	76523589	76649650	126062	+	ENSSSCG00000008357	SPRED2	protein_coding
3	76560001	76610000	0.360148	3	76523589	76649650	126062	+	ENSSSCG00000008357	SPRED2	protein_coding
3	88540001	88590000	0.354084	3	88580472	88584929	4458	-	ENSSSCG00000042900	NA	protein_coding
3	88540001	88590000	0.354084	3	88580475	88584529	4055	+	ENSSSCG00000044149	NA	protein_coding
3	77610001	77660000	0.421274	3	77658664	77796916	138253	+	ENSSSCG00000028947	PELI1	protein_coding
3	77620001	77670000	0.366649	3	77658664	77796916	138253	+	ENSSSCG00000028947	PELI1	protein_coding
3	99740001	99790000	0.36596	3	99769716	99775240	5525	+	ENSSSCG00000043111	NA	protein_coding
3	99750001	99800000	0.366631	3	99769716	99775240	5525	+	ENSSSCG00000043111	NA	protein_coding
3	99740001	99790000	0.36596	3	99769733	99775183	5451	-	ENSSSCG00000048793	NA	protein_coding
3	99750001	99800000	0.366631	3	99769733	99775183	5451	-	ENSSSCG00000048793	NA	protein_coding
3	37300001	37350000	0.359521	3	37310820	37324709	13890	+	ENSSSCG00000007920	NA	protein_coding
3	37300001	37350000	0.359521	3	37323723	37340401	16679	-	ENSSSCG00000007919	NA	protein_coding
3	37300001	37350000	0.359521	3	37346530	37365360	18831	+	ENSSSCG00000025072	C16orf89	protein_coding
3	37600001	37650000	0.362735	3	37591815	37602112	10298	+	ENSSSCG00000007932	ROGDI	protein_coding
3	37600001	37650000	0.362735	3	37606277	37628920	22644	+	ENSSSCG00000007933	SEPTIN12	protein_coding
3	37610001	37660000	0.352647	3	37606277	37628920	22644	+	ENSSSCG00000007933	SEPTIN12	protein_coding
3	37600001	37650000	0.362735	3	37639811	37689501	49691	+	ENSSSCG00000007939	ANKS3	protein_coding
3	37610001	37660000	0.352647	3	37639811	37689501	49691	+	ENSSSCG00000007939	ANKS3	protein_coding
3	37600001	37650000	0.362735	3	37642516	37649779	7264	+	ENSSSCG00000048144	NA	protein_coding
3	37610001	37660000	0.352647	3	37642516	37649779	7264	+	ENSSSCG00000048144	NA	protein_coding
3	37610001	37660000	0.352647	3	37652538	37665474	12937	-	ENSSSCG00000007935	NA	protein_coding
3	37740001	37790000	0.377921	3	37684207	37748039	63833	-	ENSSSCG00000035999	NUDT16L1	protein_coding
3	37740001	37790000	0.377921	3	37689708	37748068	58361	-	ENSSSCG00000007930	MGRN1	protein_coding
3	37740001	37790000	0.377921	3	37753280	37758870	5591	+	ENSSSCG00000023603	UBALD1	protein_coding
3	37750001	37800000	0.358062	3	37753280	37758870	5591	+	ENSSSCG00000023603	UBALD1	protein_coding
3	37740001	37790000	0.377921	3	37764206	37814314	50109	-	ENSSSCG00000007940	NA	protein_coding
3	37750001	37800000	0.358062	3	37764206	37814314	50109	-	ENSSSCG00000007940	NA	protein_coding
3	37760001	37810000	0.367224	3	37764206	37814314	50109	-	ENSSSCG00000007940	NA	protein_coding
3	37800001	37850000	0.360387	3	37764206	37814314	50109	-	ENSSSCG00000007940	NA	protein_coding
3	37800001	37850000	0.360387	3	37815370	37844608	29239	+	ENSSSCG00000007941	CDIP1	protein_coding
3	37800001	37850000	0.360387	3	37818658	37881116	62459	+	ENSSSCG00000007943	NMRAL1	protein_coding

3	37800001	37850000	0.360387	3	37831434	37868128	36695	-	ENSSSCG00000007942	HMOX2	protein_coding
3	38020001	38070000	0.357969	3	38048128	38060819	12692	+	ENSSSCG00000040215	TFAP4	protein_coding
3	39180001	39230000	0.367204	3	39184388	39213292	28905	-	ENSSSCG00000026248	NA	protein_coding
3	39190001	39240000	0.391923	3	39184388	39213292	28905	-	ENSSSCG00000026248	NA	protein_coding
3	39200001	39250000	0.372362	3	39184388	39213292	28905	-	ENSSSCG00000026248	NA	protein_coding
3	39210001	39260000	0.381802	3	39184388	39213292	28905	-	ENSSSCG00000026248	NA	protein_coding
3	89880001	89930000	0.359206	3	89799543	90914205	1114663	+	ENSSSCG00000026180	NRXN1	protein_coding
3	39180001	39230000	0.367204	3	39201878	39209263	7386	+	ENSSSCG00000029264	PKMYT1	protein_coding
3	39190001	39240000	0.391923	3	39201878	39209263	7386	+	ENSSSCG00000029264	PKMYT1	protein_coding
3	39200001	39250000	0.372362	3	39201878	39209263	7386	+	ENSSSCG00000029264	PKMYT1	protein_coding
3	39180001	39230000	0.367204	3	39214248	39218776	4529	-	ENSSSCG00000035052	KREMEN2	protein_coding
3	39190001	39240000	0.391923	3	39214248	39218776	4529	-	ENSSSCG00000035052	KREMEN2	protein_coding
3	39200001	39250000	0.372362	3	39214248	39218776	4529	-	ENSSSCG00000035052	KREMEN2	protein_coding
3	39210001	39260000	0.381802	3	39214248	39218776	4529	-	ENSSSCG00000035052	KREMEN2	protein_coding
3	39180001	39230000	0.367204	3	39225365	39244753	19389	-	ENSSSCG00000028381	FLYWCH1	protein_coding
3	39190001	39240000	0.391923	3	39225365	39244753	19389	-	ENSSSCG00000028381	FLYWCH1	protein_coding
3	39200001	39250000	0.372362	3	39225365	39244753	19389	-	ENSSSCG00000028381	FLYWCH1	protein_coding
3	39210001	39260000	0.381802	3	39225365	39244753	19389	-	ENSSSCG00000028381	FLYWCH1	protein_coding
3	39220001	39270000	0.37913	3	39225365	39244753	19389	-	ENSSSCG00000028381	FLYWCH1	protein_coding
3	39230001	39280000	0.354968	3	39225365	39244753	19389	-	ENSSSCG00000028381	FLYWCH1	protein_coding
3	39210001	39260000	0.381802	3	39251494	39261173	9680	-	ENSSSCG00000022630	FLYWCH2	protein_coding
3	39220001	39270000	0.37913	3	39251494	39261173	9680	-	ENSSSCG00000022630	FLYWCH2	protein_coding
3	39230001	39280000	0.354968	3	39251494	39261173	9680	-	ENSSSCG00000022630	FLYWCH2	protein_coding
3	39230001	39280000	0.354968	3	39275288	39293738	18451	+	ENSSSCG00000023304	SRRM2	protein_coding
3	39360001	39410000	0.358448	3	39344117	39379982	35866	-	ENSSSCG00000037112	NA	protein_coding
3	39370001	39420000	0.361784	3	39344117	39379982	35866	-	ENSSSCG00000037112	NA	protein_coding
3	39360001	39410000	0.358448	3	39365810	39380489	14680	-	ENSSSCG00000035594	PRSS22	protein_coding
3	39370001	39420000	0.361784	3	39365810	39380489	14680	-	ENSSSCG00000035594	PRSS22	protein_coding
3	39380001	39430000	0.367777	3	39365810	39380489	14680	-	ENSSSCG00000035594	PRSS22	protein_coding
3	39360001	39410000	0.358448	3	39395274	39399970	4697	+	ENSSSCG00000008062	PRSS27	protein_coding
3	39370001	39420000	0.361784	3	39395274	39399970	4697	+	ENSSSCG00000008062	PRSS27	protein_coding
3	39380001	39430000	0.367777	3	39395274	39399970	4697	+	ENSSSCG00000008062	PRSS27	protein_coding
3	39390001	39440000	0.398266	3	39395274	39399970	4697	+	ENSSSCG00000008062	PRSS27	protein_coding
3	39360001	39410000	0.358448	3	39403985	39429588	25604	-	ENSSSCG00000008058	NA	protein_coding
3	39370001	39420000	0.361784	3	39403985	39429588	25604	-	ENSSSCG00000008058	NA	protein_coding
3	39380001	39430000	0.367777	3	39403985	39429588	25604	-	ENSSSCG00000008058	NA	protein_coding
3	39390001	39440000	0.398266	3	39403985	39429588	25604	-	ENSSSCG00000008058	NA	protein_coding
3	39400001	39450000	0.394052	3	39403985	39429588	25604	-	ENSSSCG00000008058	NA	protein_coding
3	39410001	39460000	0.389689	3	39403985	39429588	25604	-	ENSSSCG00000008058	NA	protein_coding
3	39420001	39470000	0.397175	3	39403985	39429588	25604	-	ENSSSCG00000008058	NA	protein_coding
3	39380001	39430000	0.367777	3	39429931	39532583	102653	-	ENSSSCG00000037719	PDPK1	protein_coding
3	39390001	39440000	0.398266	3	39429931	39532583	102653	-	ENSSSCG00000037719	PDPK1	protein_coding
3	39400001	39450000	0.394052	3	39429931	39532583	102653	-	ENSSSCG00000037719	PDPK1	protein_coding
3	39410001	39460000	0.389689	3	39429931	39532583	102653	-	ENSSSCG00000037719	PDPK1	protein_coding
3	39420001	39470000	0.397175	3	39429931	39532583	102653	-	ENSSSCG00000037719	PDPK1	protein_coding

3	39430001	39480000	0.418644	3	39429931	39532583	102653	-	ENSSSCG00000037719	PDPK1	protein_coding
3	39440001	39490000	0.394006	3	39429931	39532583	102653	-	ENSSSCG00000037719	PDPK1	protein_coding
3	39450001	39500000	0.404282	3	39429931	39532583	102653	-	ENSSSCG00000037719	PDPK1	protein_coding
3	39460001	39510000	0.405175	3	39429931	39532583	102653	-	ENSSSCG00000037719	PDPK1	protein_coding
3	39470001	39520000	0.38292	3	39429931	39532583	102653	-	ENSSSCG00000037719	PDPK1	protein_coding
3	39480001	39530000	0.374188	3	39429931	39532583	102653	-	ENSSSCG00000037719	PDPK1	protein_coding
3	39490001	39540000	0.381704	3	39429931	39532583	102653	-	ENSSSCG00000037719	PDPK1	protein_coding
3	39500001	39550000	0.379508	3	39429931	39532583	102653	-	ENSSSCG00000037719	PDPK1	protein_coding
3	39510001	39560000	0.371859	3	39429931	39532583	102653	-	ENSSSCG00000037719	PDPK1	protein_coding
3	39520001	39570000	0.392615	3	39429931	39532583	102653	-	ENSSSCG00000037719	PDPK1	protein_coding
3	39530001	39580000	0.38971	3	39429931	39532583	102653	-	ENSSSCG00000037719	PDPK1	protein_coding
3	39480001	39530000	0.374188	3	39522160	39533741	11582	-	ENSSSCG00000008057	NA	protein_coding
3	39490001	39540000	0.381704	3	39522160	39533741	11582	-	ENSSSCG00000008057	NA	protein_coding
3	39500001	39550000	0.379508	3	39522160	39533741	11582	-	ENSSSCG00000008057	NA	protein_coding
3	39510001	39560000	0.371859	3	39522160	39533741	11582	-	ENSSSCG00000008057	NA	protein_coding
3	39520001	39570000	0.392615	3	39522160	39533741	11582	-	ENSSSCG00000008057	NA	protein_coding
3	39530001	39580000	0.38971	3	39522160	39533741	11582	-	ENSSSCG00000008057	NA	protein_coding
3	39490001	39540000	0.381704	3	39532714	39538681	5968	-	ENSSSCG00000008056	NA	protein_coding
3	39500001	39550000	0.379508	3	39532714	39538681	5968	-	ENSSSCG00000008056	NA	protein_coding
3	39510001	39560000	0.371859	3	39532714	39538681	5968	-	ENSSSCG00000008056	NA	protein_coding
3	39520001	39570000	0.392615	3	39532714	39538681	5968	-	ENSSSCG00000008056	NA	protein_coding
3	39530001	39580000	0.38971	3	39532714	39538681	5968	-	ENSSSCG00000008056	NA	protein_coding
3	39500001	39550000	0.379508	3	39545187	39571298	26112	-	ENSSSCG00000027098	NA	protein_coding
3	39510001	39560000	0.371859	3	39545187	39571298	26112	-	ENSSSCG00000027098	NA	protein_coding
3	39520001	39570000	0.392615	3	39545187	39571298	26112	-	ENSSSCG00000027098	NA	protein_coding
3	39530001	39580000	0.38971	3	39545187	39571298	26112	-	ENSSSCG00000027098	NA	protein_coding
3	39540001	39590000	0.367888	3	39545187	39571298	26112	-	ENSSSCG00000027098	NA	protein_coding
3	39550001	39600000	0.372348	3	39545187	39571298	26112	-	ENSSSCG00000027098	NA	protein_coding
3	39560001	39610000	0.365563	3	39545187	39571298	26112	-	ENSSSCG00000027098	NA	protein_coding
3	39530001	39580000	0.38971	3	39572531	39574971	2441	-	ENSSSCG00000008054	NTN3	protein_coding
3	39540001	39590000	0.367888	3	39572531	39574971	2441	-	ENSSSCG00000008054	NTN3	protein_coding
3	39550001	39600000	0.372348	3	39572531	39574971	2441	-	ENSSSCG00000008054	NTN3	protein_coding
3	39560001	39610000	0.365563	3	39572531	39574971	2441	-	ENSSSCG00000008054	NTN3	protein_coding
3	39540001	39590000	0.367888	3	39580200	39584386	4187	-	ENSSSCG00000008055	TEDC2	protein_coding
3	39550001	39600000	0.372348	3	39580200	39584386	4187	-	ENSSSCG00000008055	TEDC2	protein_coding
3	39560001	39610000	0.365563	3	39580200	39584386	4187	-	ENSSSCG00000008055	TEDC2	protein_coding
3	39540001	39590000	0.367888	3	39585602	39603083	17482	-	ENSSSCG00000026326	CCNF	protein_coding
3	39550001	39600000	0.372348	3	39585602	39603083	17482	-	ENSSSCG00000026326	CCNF	protein_coding
3	39560001	39610000	0.365563	3	39585602	39603083	17482	-	ENSSSCG00000026326	CCNF	protein_coding
3	39630001	39680000	0.363994	3	39648130	39648552	423	+	ENSSSCG00000031982	NA	pseudogene
3	39640001	39690000	0.365825	3	39648130	39648552	423	+	ENSSSCG00000031982	NA	pseudogene
3	39630001	39680000	0.363994	3	39666440	39667494	1055	-	ENSSSCG00000008052	NA	protein_coding
3	39640001	39690000	0.365825	3	39666440	39667494	1055	-	ENSSSCG00000008052	NA	protein_coding
3	39650001	39700000	0.37404	3	39666440	39667494	1055	-	ENSSSCG00000008052	NA	protein_coding
3	39660001	39710000	0.367099	3	39666440	39667494	1055	-	ENSSSCG00000008052	NA	protein_coding

3	39640001	39690000	0.365825	3	39689202	39732979	43778	+	ENSSSCG00000008051	ABCA3	protein_coding
3	39650001	39700000	0.37404	3	39689202	39732979	43778	+	ENSSSCG00000008051	ABCA3	protein_coding
3	39660001	39710000	0.367099	3	39689202	39732979	43778	+	ENSSSCG00000008051	ABCA3	protein_coding
3	39670001	39720000	0.38664	3	39689202	39732979	43778	+	ENSSSCG00000008051	ABCA3	protein_coding
3	39680001	39730000	0.401364	3	39689202	39732979	43778	+	ENSSSCG00000008051	ABCA3	protein_coding
3	39690001	39740000	0.390867	3	39689202	39732979	43778	+	ENSSSCG00000008051	ABCA3	protein_coding
3	39700001	39750000	0.369549	3	39689202	39732979	43778	+	ENSSSCG00000008051	ABCA3	protein_coding
3	39710001	39760000	0.370655	3	39689202	39732979	43778	+	ENSSSCG00000008051	ABCA3	protein_coding
3	39690001	39740000	0.390867	3	39739308	39758376	19069	+	ENSSSCG00000008048	RNPS1	protein_coding
3	39700001	39750000	0.369549	3	39739308	39758376	19069	+	ENSSSCG00000008048	RNPS1	protein_coding
3	39710001	39760000	0.370655	3	39739308	39758376	19069	+	ENSSSCG00000008048	RNPS1	protein_coding
3	39710001	39760000	0.370655	3	39757290	39772208	14919	-	ENSSSCG00000008047	E4F1	protein_coding
3	39710001	39760000	0.370655	3	39757296	39772192	14897	-	ENSSSCG00000024587	NA	pseudogene
3	41960001	42010000	0.358542	3	41945843	42052920	107078	-	ENSSSCG00000008068	IARS1	protein_coding
3	41970001	42020000	0.368384	3	41945843	42052920	107078	-	ENSSSCG00000008068	IARS1	protein_coding
3	41980001	42030000	0.367684	3	41945843	42052920	107078	-	ENSSSCG00000008068	IARS1	protein_coding
3	41980001	42030000	0.367684	3	42026278	42052773	26496	-	ENSSSCG00000008069	NOL8	protein_coding
3	42270001	42320000	0.354429	3	42053058	42308381	255324	+	ENSSSCG00000008070	CENPP	protein_coding
3	42280001	42330000	0.372014	3	42053058	42308381	255324	+	ENSSSCG00000008070	CENPP	protein_coding
3	42290001	42340000	0.387629	3	42053058	42308381	255324	+	ENSSSCG00000008070	CENPP	protein_coding
3	42300001	42350000	0.394395	3	42053058	42308381	255324	+	ENSSSCG00000008070	CENPP	protein_coding
3	42270001	42320000	0.354429	3	42308005	42368224	60220	-	ENSSSCG00000030204	IPPK	protein_coding
3	42280001	42330000	0.372014	3	42308005	42368224	60220	-	ENSSSCG00000030204	IPPK	protein_coding
3	42290001	42340000	0.387629	3	42308005	42368224	60220	-	ENSSSCG00000030204	IPPK	protein_coding
3	42300001	42350000	0.394395	3	42308005	42368224	60220	-	ENSSSCG00000030204	IPPK	protein_coding
3	42310001	42360000	0.397593	3	42308005	42368224	60220	-	ENSSSCG00000030204	IPPK	protein_coding
3	42320001	42370000	0.414524	3	42308005	42368224	60220	-	ENSSSCG00000030204	IPPK	protein_coding
3	42330001	42380000	0.42314	3	42308005	42368224	60220	-	ENSSSCG00000030204	IPPK	protein_coding
3	42340001	42390000	0.419927	3	42308005	42368224	60220	-	ENSSSCG00000030204	IPPK	protein_coding
3	42350001	42400000	0.413653	3	42308005	42368224	60220	-	ENSSSCG00000030204	IPPK	protein_coding
3	42360001	42410000	0.394129	3	42308005	42368224	60220	-	ENSSSCG00000030204	IPPK	protein_coding
3	42320001	42370000	0.414524	3	42368445	42456791	88347	-	ENSSSCG00000027166	BICD2	protein_coding
3	42330001	42380000	0.42314	3	42368445	42456791	88347	-	ENSSSCG00000027166	BICD2	protein_coding
3	42340001	42390000	0.419927	3	42368445	42456791	88347	-	ENSSSCG00000027166	BICD2	protein_coding
3	42350001	42400000	0.413653	3	42368445	42456791	88347	-	ENSSSCG00000027166	BICD2	protein_coding
3	42360001	42410000	0.394129	3	42368445	42456791	88347	-	ENSSSCG00000027166	BICD2	protein_coding
3	109520001	109570000	0.385258	3	109411252	110067977	656726	+	ENSSSCG00000008533	NA	protein_coding
3	109550001	109600000	0.367002	3	109411252	110067977	656726	+	ENSSSCG00000008533	NA	protein_coding
3	109560001	109610000	0.360186	3	109411252	110067977	656726	+	ENSSSCG00000008533	NA	protein_coding
3	109520001	109570000	0.385258	3	109559170	109563210	4041	-	ENSSSCG00000045172	NA	pseudogene
3	109550001	109600000	0.367002	3	109559170	109563210	4041	-	ENSSSCG00000045172	NA	pseudogene
3	109560001	109610000	0.360186	3	109559170	109563210	4041	-	ENSSSCG00000045172	NA	pseudogene
3	46690001	46740000	0.419121	3	46701630	46714122	12493	-	ENSSSCG00000008123	ARID5A	protein_coding
3	46700001	46750000	0.490432	3	46701630	46714122	12493	-	ENSSSCG00000008123	ARID5A	protein_coding
3	46710001	46760000	0.496001	3	46701630	46714122	12493	-	ENSSSCG00000008123	ARID5A	protein_coding

3	46700001	46750000	0.490432	3	46742971	46751547	8577	+	ENSSSCG00000008124	NEURL3	protein_coding
3	46710001	46760000	0.496001	3	46742971	46751547	8577	+	ENSSSCG00000008124	NEURL3	protein_coding
3	46720001	46770000	0.489808	3	46742971	46751547	8577	+	ENSSSCG00000008124	NEURL3	protein_coding
3	46730001	46780000	0.472526	3	46742971	46751547	8577	+	ENSSSCG00000008124	NEURL3	protein_coding
3	46740001	46790000	0.481475	3	46742971	46751547	8577	+	ENSSSCG00000008124	NEURL3	protein_coding
3	46750001	46800000	0.484569	3	46742971	46751547	8577	+	ENSSSCG00000008124	NEURL3	protein_coding
3	51820001	51870000	0.358269	3	51803165	51951383	148219	-	ENSSSCG00000032795	IL1RL1	protein_coding
3	51830001	51880000	0.374513	3	51803165	51951383	148219	-	ENSSSCG00000032795	IL1RL1	protein_coding
3	51890001	51940000	0.363089	3	51803165	51951383	148219	-	ENSSSCG00000032795	IL1RL1	protein_coding
3	51900001	51950000	0.388272	3	51803165	51951383	148219	-	ENSSSCG00000032795	IL1RL1	protein_coding
3	51910001	51960000	0.391264	3	51803165	51951383	148219	-	ENSSSCG00000032795	IL1RL1	protein_coding
3	51920001	51970000	0.405137	3	51803165	51951383	148219	-	ENSSSCG00000032795	IL1RL1	protein_coding
3	51930001	51980000	0.37601	3	51803165	51951383	148219	-	ENSSSCG00000032795	IL1RL1	protein_coding
3	51940001	51990000	0.370248	3	51803165	51951383	148219	-	ENSSSCG00000032795	IL1RL1	protein_coding
3	51820001	51870000	0.358269	3	51803404	51902079	98676	-	ENSSSCG00000008159	IL18R1	protein_coding
3	51830001	51880000	0.374513	3	51803404	51902079	98676	-	ENSSSCG00000008159	IL18R1	protein_coding
3	51890001	51940000	0.363089	3	51803404	51902079	98676	-	ENSSSCG00000008159	IL18R1	protein_coding
3	51900001	51950000	0.388272	3	51803404	51902079	98676	-	ENSSSCG00000008159	IL18R1	protein_coding
3	51940001	51990000	0.370248	3	51980026	52011602	31577	-	ENSSSCG00000023550	IL1RL2	protein_coding
3	51930001	51980000	0.37601	3	51974443	51977019	2577	+	ENSSSCG00000042975	NA	protein_coding
3	51940001	51990000	0.370248	3	51974443	51977019	2577	+	ENSSSCG00000042975	NA	protein_coding
3	54230001	54280000	0.360856	3	54135532	54862224	726693	+	ENSSSCG00000008179	REV1	protein_coding
3	54240001	54290000	0.402421	3	54135532	54862224	726693	+	ENSSSCG00000008179	REV1	protein_coding
3	54250001	54300000	0.426123	3	54135532	54862224	726693	+	ENSSSCG00000008179	REV1	protein_coding
3	54260001	54310000	0.40682	3	54135532	54862224	726693	+	ENSSSCG00000008179	REV1	protein_coding
3	54270001	54320000	0.433278	3	54135532	54862224	726693	+	ENSSSCG00000008179	REV1	protein_coding
3	54280001	54330000	0.479026	3	54135532	54862224	726693	+	ENSSSCG00000008179	REV1	protein_coding
3	54290001	54340000	0.483259	3	54135532	54862224	726693	+	ENSSSCG00000008179	REV1	protein_coding
3	54300001	54350000	0.465978	3	54135532	54862224	726693	+	ENSSSCG00000008179	REV1	protein_coding
3	54310001	54360000	0.45588	3	54135532	54862224	726693	+	ENSSSCG00000008179	REV1	protein_coding
3	54320001	54370000	0.413577	3	54135532	54862224	726693	+	ENSSSCG00000008179	REV1	protein_coding
3	54560001	54610000	0.381072	3	54135532	54862224	726693	+	ENSSSCG00000008179	REV1	protein_coding
3	54570001	54620000	0.374591	3	54135532	54862224	726693	+	ENSSSCG00000008179	REV1	protein_coding
3	54580001	54630000	0.376869	3	54135532	54862224	726693	+	ENSSSCG00000008179	REV1	protein_coding
3	54560001	54610000	0.381072	3	54574132	54579715	5584	-	ENSSSCG00000041855	NA	pseudogene
3	54570001	54620000	0.374591	3	54574132	54579715	5584	-	ENSSSCG00000041855	NA	pseudogene
3	55030001	55080000	0.35382	3	55017572	55032344	14773	+	ENSSSCG00000034542	LYG2	protein_coding
3	55030001	55080000	0.35382	3	55032996	55108355	75360	-	ENSSSCG00000008183	NA	protein_coding
3	55030001	55080000	0.35382	3	55054516	55069867	15352	+	ENSSSCG00000008185	MITD1	protein_coding
3	55030001	55080000	0.35382	3	55054516	55061640	7125	+	ENSSSCG00000045084	NA	protein_coding
3	55030001	55080000	0.35382	3	55056723	55064155	7433	-	ENSSSCG00000045892	NA	protein_coding
7	58300001	58350000	0.394569	7	58343532	58345481	1950	+	ENSSSCG00000041349	NA	processed_pseudogene
7	58310001	58360000	0.3603	7	58343532	58345481	1950	+	ENSSSCG00000041349	NA	processed_pseudogene
7	58260001	58310000	0.372751	7	58280545	58280648	104	-	ENSSSCG00000020055	U6	snRNA
7	58270001	58320000	0.430683	7	58280545	58280648	104	-	ENSSSCG00000020055	U6	snRNA

7	58280001	58330000	0.471373	7	58280545	58280648	104	-	ENSSSCG00000020055	U6	snRNA
7	57980001	58030000	0.396912	7	58023368	58024496	1129	+	ENSSSCG00000042572	IMP3	protein_coding
7	57990001	58040000	0.433919	7	58023368	58024496	1129	+	ENSSSCG00000042572	IMP3	protein_coding
7	58000001	58050000	0.441851	7	58023368	58024496	1129	+	ENSSSCG00000042572	IMP3	protein_coding
7	58010001	58060000	0.430268	7	58023368	58024496	1129	+	ENSSSCG00000042572	IMP3	protein_coding
7	58020001	58070000	0.425452	7	58023368	58024496	1129	+	ENSSSCG00000042572	IMP3	protein_coding
7	93310001	93360000	0.363408	7	93253757	93344187	90431	+	ENSSSCG00000002311	SUSD6	protein_coding
7	97170001	97220000	0.3817	7	97174219	97195597	21379	+	ENSSSCG00000002351	NA	protein_coding
7	97180001	97230000	0.399576	7	97174219	97195597	21379	+	ENSSSCG00000002351	NA	protein_coding
7	97190001	97240000	0.375463	7	97174219	97195597	21379	+	ENSSSCG00000002351	NA	protein_coding
7	97170001	97220000	0.3817	7	97185797	97270560	84764	+	ENSSSCG00000002352	ZNF410	protein_coding
7	97180001	97230000	0.399576	7	97185797	97270560	84764	+	ENSSSCG00000002352	ZNF410	protein_coding
7	97190001	97240000	0.375463	7	97185797	97270560	84764	+	ENSSSCG00000002352	ZNF410	protein_coding
7	97200001	97250000	0.383483	7	97185797	97270560	84764	+	ENSSSCG00000002352	ZNF410	protein_coding
7	97200001	97250000	0.383483	7	97244894	97258408	13515	-	ENSSSCG00000002353	FAM161B	protein_coding
7	3270001	3320000	0.364607	7	3192894	3593896	401003	+	ENSSSCG00000038717	NA	protein_coding
7	3280001	3330000	0.423443	7	3192894	3593896	401003	+	ENSSSCG00000038717	NA	protein_coding
7	3290001	3340000	0.416451	7	3192894	3593896	401003	+	ENSSSCG00000038717	NA	protein_coding
7	3300001	3350000	0.394978	7	3192894	3593896	401003	+	ENSSSCG00000038717	NA	protein_coding
7	101910001	101960000	0.366074	7	101132830	102777822	1644993	+	ENSSSCG00000028942	NRXN3	protein_coding
7	55360001	55410000	0.368509	7	55351083	55373881	22799	-	ENSSSCG00000001849	ANPEP	protein_coding
7	55370001	55420000	0.372483	7	55351083	55373881	22799	-	ENSSSCG00000001849	ANPEP	protein_coding
7	55360001	55410000	0.368509	7	55391913	55462823	70911	-	ENSSSCG00000030174	AP3S2	protein_coding
7	55370001	55420000	0.372483	7	55391913	55462823	70911	-	ENSSSCG00000030174	AP3S2	protein_coding
7	55380001	55430000	0.367166	7	55391913	55462823	70911	-	ENSSSCG00000030174	AP3S2	protein_coding
7	55390001	55440000	0.36733	7	55391913	55462823	70911	-	ENSSSCG00000030174	AP3S2	protein_coding
7	55400001	55450000	0.367234	7	55391913	55462823	70911	-	ENSSSCG00000030174	AP3S2	protein_coding
7	55410001	55460000	0.367625	7	55391913	55462823	70911	-	ENSSSCG00000030174	AP3S2	protein_coding
7	55420001	55470000	0.374677	7	55391913	55462823	70911	-	ENSSSCG00000030174	AP3S2	protein_coding
7	55430001	55480000	0.406271	7	55391913	55462823	70911	-	ENSSSCG00000030174	AP3S2	protein_coding
7	55440001	55490000	0.434304	7	55391913	55462823	70911	-	ENSSSCG00000030174	AP3S2	protein_coding
7	55450001	55500000	0.445134	7	55391913	55462823	70911	-	ENSSSCG00000030174	AP3S2	protein_coding
7	55460001	55510000	0.45632	7	55391913	55462823	70911	-	ENSSSCG00000030174	AP3S2	protein_coding
7	55360001	55410000	0.368509	7	55394189	55483995	89807	-	ENSSSCG00000001850	NA	protein_coding
7	55370001	55420000	0.372483	7	55394189	55483995	89807	-	ENSSSCG00000001850	NA	protein_coding
7	55380001	55430000	0.367166	7	55394189	55483995	89807	-	ENSSSCG00000001850	NA	protein_coding
7	55390001	55440000	0.36733	7	55394189	55483995	89807	-	ENSSSCG00000001850	NA	protein_coding
7	55400001	55450000	0.367234	7	55394189	55483995	89807	-	ENSSSCG00000001850	NA	protein_coding
7	55410001	55460000	0.367625	7	55394189	55483995	89807	-	ENSSSCG00000001850	NA	protein_coding
7	55420001	55470000	0.374677	7	55394189	55483995	89807	-	ENSSSCG00000001850	NA	protein_coding
7	55430001	55480000	0.406271	7	55394189	55483995	89807	-	ENSSSCG00000001850	NA	protein_coding
7	55440001	55490000	0.434304	7	55394189	55483995	89807	-	ENSSSCG00000001850	NA	protein_coding
7	55450001	55500000	0.445134	7	55394189	55483995	89807	-	ENSSSCG00000001850	NA	protein_coding
7	55460001	55510000	0.45632	7	55394189	55483995	89807	-	ENSSSCG00000001850	NA	protein_coding
7	55470001	55520000	0.499776	7	55394189	55483995	89807	-	ENSSSCG00000001850	NA	protein_coding

7	55480001	55530000	0.483878	7	55394189	55483995	89807	-	ENSSSCG00000001850	NA	protein_coding
7	55440001	55490000	0.434304	7	55482387	55652934	170548	+	ENSSSCG00000021361	ZNF710	protein_coding
7	55450001	55500000	0.445134	7	55482387	55652934	170548	+	ENSSSCG00000021361	ZNF710	protein_coding
7	55460001	55510000	0.45632	7	55482387	55652934	170548	+	ENSSSCG00000021361	ZNF710	protein_coding
7	55470001	55520000	0.499776	7	55482387	55652934	170548	+	ENSSSCG00000021361	ZNF710	protein_coding
7	55480001	55530000	0.483878	7	55482387	55652934	170548	+	ENSSSCG00000021361	ZNF710	protein_coding
7	55490001	55540000	0.428835	7	55482387	55652934	170548	+	ENSSSCG00000021361	ZNF710	protein_coding
7	55500001	55550000	0.377054	7	55482387	55652934	170548	+	ENSSSCG00000021361	ZNF710	protein_coding
7	57040001	57090000	0.365539	7	57041003	57059331	18329	+	ENSSSCG00000001866	RCN2	protein_coding
7	57050001	57100000	0.368535	7	57041003	57059331	18329	+	ENSSSCG00000001866	RCN2	protein_coding
7	57060001	57110000	0.36007	7	57102756	57146754	43999	+	ENSSSCG00000001867	PSTPIP1	protein_coding
7	57070001	57120000	0.366093	7	57102756	57146754	43999	+	ENSSSCG00000001867	PSTPIP1	protein_coding
7	57080001	57130000	0.362523	7	57102756	57146754	43999	+	ENSSSCG00000001867	PSTPIP1	protein_coding
7	57860001	57910000	0.357646	7	57684589	57890126	205538	-	ENSSSCG00000001872	LINGO1	protein_coding
7	57870001	57920000	0.358752	7	57684589	57890126	205538	-	ENSSSCG00000001872	LINGO1	protein_coding
7	57980001	58030000	0.396912	7	57949455	57988337	38883	+	ENSSSCG00000001873	CSPG4	protein_coding
7	57980001	58030000	0.396912	7	57991790	58014972	23183	-	ENSSSCG00000001875	SNX33	protein_coding
7	57990001	58040000	0.433919	7	57991790	58014972	23183	-	ENSSSCG00000001875	SNX33	protein_coding
7	58000001	58050000	0.441851	7	57991790	58014972	23183	-	ENSSSCG00000001875	SNX33	protein_coding
7	58010001	58060000	0.430268	7	57991790	58014972	23183	-	ENSSSCG00000001875	SNX33	protein_coding
7	57990001	58040000	0.433919	7	58038953	58071277	32325	+	ENSSSCG00000001877	SNUPN	protein_coding
7	58000001	58050000	0.441851	7	58038953	58071277	32325	+	ENSSSCG00000001877	SNUPN	protein_coding
7	58010001	58060000	0.430268	7	58038953	58071277	32325	+	ENSSSCG00000001877	SNUPN	protein_coding
7	58020001	58070000	0.425452	7	58038953	58071277	32325	+	ENSSSCG00000001877	SNUPN	protein_coding
7	58030001	58080000	0.400961	7	58038953	58071277	32325	+	ENSSSCG00000001877	SNUPN	protein_coding
7	58030001	58080000	0.400961	7	58078389	58164405	86017	+	ENSSSCG00000001878	PTPN9	protein_coding
7	58090001	58140000	0.361516	7	58078389	58164405	86017	+	ENSSSCG00000001878	PTPN9	protein_coding
7	58100001	58150000	0.357171	7	58078389	58164405	86017	+	ENSSSCG00000001878	PTPN9	protein_coding
7	58110001	58160000	0.353945	7	58078389	58164405	86017	+	ENSSSCG00000001878	PTPN9	protein_coding
7	58120001	58170000	0.358471	7	58078389	58164405	86017	+	ENSSSCG00000001878	PTPN9	protein_coding
7	58090001	58140000	0.361516	7	58131067	58131839	773	-	ENSSSCG00000032951	NA	protein_coding
7	58100001	58150000	0.357171	7	58131067	58131839	773	-	ENSSSCG00000032951	NA	protein_coding
7	58110001	58160000	0.353945	7	58131067	58131839	773	-	ENSSSCG00000032951	NA	protein_coding
7	58120001	58170000	0.358471	7	58131067	58131839	773	-	ENSSSCG00000032951	NA	protein_coding
7	58260001	58310000	0.372751	7	58248384	58261203	12820	+	ENSSSCG00000001881	MAN2C1	protein_coding
7	58260001	58310000	0.372751	7	58261761	58283092	21332	-	ENSSSCG00000001882	NEIL1	protein_coding
7	58270001	58320000	0.430683	7	58261761	58283092	21332	-	ENSSSCG00000001882	NEIL1	protein_coding
7	58280001	58330000	0.471373	7	58261761	58283092	21332	-	ENSSSCG00000001882	NEIL1	protein_coding
7	58260001	58310000	0.372751	7	58276954	58283114	6161	-	ENSSSCG00000001884	COMMD4	protein_coding
7	58270001	58320000	0.430683	7	58276954	58283114	6161	-	ENSSSCG00000001884	COMMD4	protein_coding
7	58280001	58330000	0.471373	7	58276954	58283114	6161	-	ENSSSCG00000001884	COMMD4	protein_coding
7	58280001	58330000	0.471373	7	58321053	58330491	9439	+	ENSSSCG00000041453	NA	protein_coding
7	58290001	58340000	0.449755	7	58321053	58330491	9439	+	ENSSSCG00000041453	NA	protein_coding
7	58300001	58350000	0.394569	7	58321053	58330491	9439	+	ENSSSCG00000041453	NA	protein_coding
7	58310001	58360000	0.3603	7	58321053	58330491	9439	+	ENSSSCG00000041453	NA	protein_coding

7	59230001	59280000	0.35783	7	59070709	59243740	173032	+	ENSSSCG00000001907	UBL7	protein_coding
7	59230001	59280000	0.35783	7	59190179	59291021	100843	-	ENSSSCG00000001908	CCDC33	protein_coding
7	59490001	59540000	0.357625	7	59481412	59505149	23738	-	ENSSSCG00000001914	LOXL1	protein_coding
7	59490001	59540000	0.357625	7	59503316	59677107	173792	+	ENSSSCG00000001916	INSYN1	protein_coding
7	59510001	59560000	0.357159	7	59503316	59677107	173792	+	ENSSSCG00000001916	INSYN1	protein_coding
7	59520001	59570000	0.389859	7	59503316	59677107	173792	+	ENSSSCG00000001916	INSYN1	protein_coding
7	59530001	59580000	0.397152	7	59503316	59677107	173792	+	ENSSSCG00000001916	INSYN1	protein_coding
7	59540001	59590000	0.361163	7	59503316	59677107	173792	+	ENSSSCG00000001916	INSYN1	protein_coding
7	59490001	59540000	0.357625	7	59535614	59548897	13284	-	ENSSSCG00000001915	TBC1D21	protein_coding
7	59510001	59560000	0.357159	7	59535614	59548897	13284	-	ENSSSCG00000001915	TBC1D21	protein_coding
7	59520001	59570000	0.389859	7	59535614	59548897	13284	-	ENSSSCG00000001915	TBC1D21	protein_coding
7	59530001	59580000	0.397152	7	59535614	59548897	13284	-	ENSSSCG00000001915	TBC1D21	protein_coding
7	59540001	59590000	0.361163	7	59535614	59548897	13284	-	ENSSSCG00000001915	TBC1D21	protein_coding
13	198690001	198740000	0.380146	13	198730179	198750676	20498	+	ENSSSCG00000041276	NA	lncRNA
13	198700001	198750000	0.370663	13	198730179	198750676	20498	+	ENSSSCG00000041276	NA	lncRNA
13	199340001	199390000	0.353853	13	199388349	199393385	5037	+	ENSSSCG00000048579	NA	lncRNA
13	204330001	204380000	0.353319	13	204365638	204405040	39403	+	ENSSSCG00000044620	NA	lncRNA
13	204340001	204390000	0.375634	13	204365638	204405040	39403	+	ENSSSCG00000044620	NA	lncRNA
13	204350001	204400000	0.372592	13	204365638	204405040	39403	+	ENSSSCG00000044620	NA	lncRNA
13	204360001	204410000	0.375802	13	204365638	204405040	39403	+	ENSSSCG00000044620	NA	lncRNA
13	204370001	204420000	0.35992	13	204365638	204405040	39403	+	ENSSSCG00000044620	NA	lncRNA
13	206040001	206090000	0.386032	13	206046629	206048785	2157	-	ENSSSCG00000041152	NA	lncRNA
13	206040001	206090000	0.386032	13	206063945	206071041	7097	+	ENSSSCG00000051023	NA	lncRNA
13	206050001	206100000	0.403508	13	206063945	206071041	7097	+	ENSSSCG00000051023	NA	lncRNA
13	206060001	206110000	0.439773	13	206063945	206071041	7097	+	ENSSSCG00000051023	NA	lncRNA
13	206070001	206120000	0.467261	13	206063945	206071041	7097	+	ENSSSCG00000051023	NA	lncRNA
13	143390001	143440000	0.363889	13	143406780	143414408	7629	+	ENSSSCG00000047219	NA	lncRNA
13	143400001	143450000	0.36867	13	143406780	143414408	7629	+	ENSSSCG00000047219	NA	lncRNA
13	143410001	143460000	0.370864	13	143406780	143414408	7629	+	ENSSSCG00000047219	NA	lncRNA
13	143430001	143480000	0.489657	13	143475449	143482780	7332	-	ENSSSCG00000048427	NA	lncRNA
13	143440001	143490000	0.496223	13	143475449	143482780	7332	-	ENSSSCG00000048427	NA	lncRNA
13	143450001	143500000	0.451803	13	143475449	143482780	7332	-	ENSSSCG00000048427	NA	lncRNA
13	143460001	143510000	0.418406	13	143475449	143482780	7332	-	ENSSSCG00000048427	NA	lncRNA
13	136390001	136440000	0.398531	13	136395668	136430739	35072	+	ENSSSCG00000028717	NA	protein_coding
13	136400001	136450000	0.396084	13	136395668	136430739	35072	+	ENSSSCG00000028717	NA	protein_coding
13	136410001	136460000	0.393438	13	136395668	136430739	35072	+	ENSSSCG00000028717	NA	protein_coding
13	136420001	136470000	0.374607	13	136395668	136430739	35072	+	ENSSSCG00000028717	NA	protein_coding
13	136390001	136440000	0.398531	13	136438962	136501872	62911	+	ENSSSCG00000025349	CCDC14	protein_coding
13	136400001	136450000	0.396084	13	136438962	136501872	62911	+	ENSSSCG00000025349	CCDC14	protein_coding
13	136410001	136460000	0.393438	13	136438962	136501872	62911	+	ENSSSCG00000025349	CCDC14	protein_coding
13	136420001	136470000	0.374607	13	136438962	136501872	62911	+	ENSSSCG00000025349	CCDC14	protein_coding
13	143410001	143460000	0.370864	13	143456980	143459010	2031	-	ENSSSCG00000045503	NA	protein_coding
13	143420001	143470000	0.453563	13	143456980	143459010	2031	-	ENSSSCG00000045503	NA	protein_coding
13	143430001	143480000	0.489657	13	143456980	143459010	2031	-	ENSSSCG00000045503	NA	protein_coding
13	143440001	143490000	0.496223	13	143456980	143459010	2031	-	ENSSSCG00000045503	NA	protein_coding

13	143450001	143500000	0.451803	13	143456980	143459010	2031	-	ENSSSCG00000045503	NA	protein_coding
13	143670001	143720000	0.353031	13	143676005	144304151	628147	+	ENSSSCG00000027941	LSAMP	protein_coding
13	143410001	143460000	0.370864	13	143459468	143464937	5470	-	ENSSSCG00000046077	NA	protein_coding
13	143420001	143470000	0.453563	13	143459468	143464937	5470	-	ENSSSCG00000046077	NA	protein_coding
13	143430001	143480000	0.489657	13	143459468	143464937	5470	-	ENSSSCG00000046077	NA	protein_coding
13	143440001	143490000	0.496223	13	143459468	143464937	5470	-	ENSSSCG00000046077	NA	protein_coding
13	143450001	143500000	0.451803	13	143459468	143464937	5470	-	ENSSSCG00000046077	NA	protein_coding
13	143460001	143510000	0.418406	13	143459468	143464937	5470	-	ENSSSCG00000046077	NA	protein_coding
13	207370001	207420000	0.359825	13	207385108	207385863	756	+	ENSSSCG00000039836	NA	protein_coding
13	207370001	207420000	0.359825	13	207393407	207393880	474	+	ENSSSCG00000037222	NA	protein_coding
13	207370001	207420000	0.359825	13	207403602	207404237	636	+	ENSSSCG00000025793	NA	protein_coding
13	197710001	197760000	0.361006	13	197687783	197756787	69005	+	ENSSSCG00000026981	NA	protein_coding
13	197710001	197760000	0.361006	13	197688631	197718696	30066	+	ENSSSCG00000034167	SLC5A3	protein_coding
13	198470001	198520000	0.354987	13	198371835	198638189	266355	-	ENSSSCG00000035537	RUNX1	protein_coding
13	203850001	203900000	0.358853	13	203567129	204313713	746585	-	ENSSSCG00000012072	DSCAM	protein_coding
13	203860001	203910000	0.378996	13	203567129	204313713	746585	-	ENSSSCG00000012072	DSCAM	protein_coding
13	204120001	204170000	0.367709	13	203567129	204313713	746585	-	ENSSSCG00000012072	DSCAM	protein_coding
13	110140001	110190000	0.35599	13	110087051	110376319	289269	-	ENSSSCG00000011750	PLD1	protein_coding
13	110150001	110200000	0.375121	13	110087051	110376319	289269	-	ENSSSCG00000011750	PLD1	protein_coding
13	110160001	110210000	0.359185	13	110087051	110376319	289269	-	ENSSSCG00000011750	PLD1	protein_coding
13	206040001	206090000	0.386032	13	206074943	206094297	19355	-	ENSSSCG00000023078	WDR4	protein_coding
13	206050001	206100000	0.403508	13	206074943	206094297	19355	-	ENSSSCG00000023078	WDR4	protein_coding
13	206060001	206110000	0.439773	13	206074943	206094297	19355	-	ENSSSCG00000023078	WDR4	protein_coding
13	206070001	206120000	0.467261	13	206074943	206094297	19355	-	ENSSSCG00000023078	WDR4	protein_coding
13	206080001	206130000	0.491221	13	206074943	206094297	19355	-	ENSSSCG00000023078	WDR4	protein_coding
13	206090001	206140000	0.45131	13	206074943	206094297	19355	-	ENSSSCG00000023078	WDR4	protein_coding
13	206070001	206120000	0.467261	13	206111408	206127732	16325	+	ENSSSCG00000021991	NA	protein_coding
13	206080001	206130000	0.491221	13	206111408	206127732	16325	+	ENSSSCG00000021991	NA	protein_coding
13	206090001	206140000	0.45131	13	206111408	206127732	16325	+	ENSSSCG00000021991	NA	protein_coding
13	206100001	206150000	0.42317	13	206111408	206127732	16325	+	ENSSSCG00000021991	NA	protein_coding
13	206110001	206160000	0.364662	13	206111408	206127732	16325	+	ENSSSCG00000021991	NA	protein_coding
13	206110001	206160000	0.364662	13	206151647	206195470	43824	+	ENSSSCG00000037663	PKNOX1	protein_coding
13	206210001	206260000	0.359991	13	206205946	206230243	24298	-	ENSSSCG00000040779	NA	protein_coding
13	112070001	112120000	0.371744	13	112036053	112725618	689566	+	ENSSSCG00000011756	NLGN1	protein_coding
13	112080001	112130000	0.359303	13	112036053	112725618	689566	+	ENSSSCG00000011756	NLGN1	protein_coding
13	206210001	206260000	0.359991	13	206244772	206258222	13451	-	ENSSSCG00000033653	NA	protein_coding
13	207370001	207420000	0.359825	13	207285881	207412512	126632	-	ENSSSCG00000022988	TSPEAR	protein_coding
13	207370001	207420000	0.359825	13	207377502	207378437	936	+	ENSSSCG00000047876	NA	pseudogene
13	207370001	207420000	0.359825	13	207398974	207400627	1654	+	ENSSSCG00000029038	NA	protein_coding
17	36260001	36310000	0.353563	17	36072122	36325074	252953	-	ENSSSCG00000007249	NOL4L	protein_coding
17	44120001	44170000	0.362876	17	44047803	44265431	217629	-	ENSSSCG00000007360	CHD6	protein_coding
10	51450001	51500000	0.367578	10	51485895	51580194	94300	-	ENSSSCG00000048214	NA	lncRNA
10	51460001	51510000	0.38695	10	51485895	51580194	94300	-	ENSSSCG00000048214	NA	lncRNA
10	51470001	51520000	0.375878	10	51485895	51580194	94300	-	ENSSSCG00000048214	NA	lncRNA
10	26090001	26140000	0.353333	10	26112032	26264175	152144	-	ENSSSCG00000010941	ERCC6L2	protein_coding

10	26100001	26150000	0.363637	10	26112032	26264175	152144	-	ENSSSCG00000010941	ERCC6L2	protein_coding
10	26110001	26160000	0.389712	10	26112032	26264175	152144	-	ENSSSCG00000010941	ERCC6L2	protein_coding
10	26190001	26240000	0.359684	10	26112032	26264175	152144	-	ENSSSCG00000010941	ERCC6L2	protein_coding
10	26200001	26250000	0.380719	10	26112032	26264175	152144	-	ENSSSCG00000010941	ERCC6L2	protein_coding
10	31180001	31230000	0.379573	10	31141811	31248224	106414	+	ENSSSCG00000026087	GKAP1	protein_coding
10	31190001	31240000	0.393033	10	31141811	31248224	106414	+	ENSSSCG00000026087	GKAP1	protein_coding
10	31200001	31250000	0.410484	10	31141811	31248224	106414	+	ENSSSCG00000026087	GKAP1	protein_coding
10	31210001	31260000	0.379905	10	31141811	31248224	106414	+	ENSSSCG00000026087	GKAP1	protein_coding
10	31180001	31230000	0.379573	10	31226412	31232316	5905	+	ENSSSCG00000049051	NA	protein_coding
10	31190001	31240000	0.393033	10	31226412	31232316	5905	+	ENSSSCG00000049051	NA	protein_coding
10	31200001	31250000	0.410484	10	31226412	31232316	5905	+	ENSSSCG00000049051	NA	protein_coding
10	31210001	31260000	0.379905	10	31226412	31232316	5905	+	ENSSSCG00000049051	NA	protein_coding
11	9880001	9930000	0.359154	11	9814030	10029559	215530	-	ENSSSCG00000046743	NA	lncRNA
11	9890001	9940000	0.396977	11	9814030	10029559	215530	-	ENSSSCG00000046743	NA	lncRNA
11	9900001	9950000	0.361538	11	9814030	10029559	215530	-	ENSSSCG00000046743	NA	lncRNA
11	9880001	9930000	0.359154	11	9913075	10135147	222073	+	ENSSSCG00000038414	NA	lncRNA
11	9890001	9940000	0.396977	11	9913075	10135147	222073	+	ENSSSCG00000038414	NA	lncRNA
11	9900001	9950000	0.361538	11	9913075	10135147	222073	+	ENSSSCG00000038414	NA	lncRNA
11	10110001	10160000	0.352954	11	9913075	10135147	222073	+	ENSSSCG00000038414	NA	lncRNA
11	10130001	10180000	0.358057	11	9913075	10135147	222073	+	ENSSSCG00000038414	NA	lncRNA
11	9880001	9930000	0.359154	11	9923839	9928161	4323	-	ENSSSCG00000049420	NA	lncRNA
11	9890001	9940000	0.396977	11	9923839	9928161	4323	-	ENSSSCG00000049420	NA	lncRNA
11	9900001	9950000	0.361538	11	9923839	9928161	4323	-	ENSSSCG00000049420	NA	lncRNA
11	10110001	10160000	0.352954	11	10145974	10527268	381295	+	ENSSSCG00000009349	RFC3	protein_coding
11	10130001	10180000	0.358057	11	10145974	10527268	381295	+	ENSSSCG00000009349	RFC3	protein_coding
11	10140001	10190000	0.366616	11	10145974	10527268	381295	+	ENSSSCG00000009349	RFC3	protein_coding
11	10150001	10200000	0.360282	11	10145974	10527268	381295	+	ENSSSCG00000009349	RFC3	protein_coding
11	10160001	10210000	0.362355	11	10145974	10527268	381295	+	ENSSSCG00000009349	RFC3	protein_coding
11	10170001	10220000	0.383123	11	10145974	10527268	381295	+	ENSSSCG00000009349	RFC3	protein_coding
11	10150001	10200000	0.360282	11	10195775	10198499	2725	-	ENSSSCG00000051664	NA	pseudogene
11	10160001	10210000	0.362355	11	10195775	10198499	2725	-	ENSSSCG00000051664	NA	pseudogene
11	10170001	10220000	0.383123	11	10195775	10198499	2725	-	ENSSSCG00000051664	NA	pseudogene
11	42110001	42160000	0.363273	11	42033842	42419770	385929	-	ENSSSCG00000009456	KLHL1	protein_coding
14	114050001	114100000	0.358289	14	113890149	114053352	163204	+	ENSSSCG00000010593	CNNM2	protein_coding
14	114050001	114100000	0.358289	14	114062457	114168204	105748	-	ENSSSCG00000010594	NT5C2	protein_coding
14	114060001	114110000	0.386583	14	114062457	114168204	105748	-	ENSSSCG00000010594	NT5C2	protein_coding
14	114070001	114120000	0.395114	14	114062457	114168204	105748	-	ENSSSCG00000010594	NT5C2	protein_coding
14	114080001	114130000	0.39137	14	114062457	114168204	105748	-	ENSSSCG00000010594	NT5C2	protein_coding
14	114090001	114140000	0.384147	14	114062457	114168204	105748	-	ENSSSCG00000010594	NT5C2	protein_coding
14	114100001	114150000	0.358148	14	114062457	114168204	105748	-	ENSSSCG00000010594	NT5C2	protein_coding

Table S8: 2,930 genes for adipose tissues predicted from SNPs using FarmGTEx TWAS-server.

Genes_prediction	LU_1	LU_2	LU_3	LU_4	LU_5	LU_6	LU_7	LU_8	LW_1	LW_2	LW_3	LW_4	LW_5	LW_6	LW_7	LW_8	pvalue	FC	log2(FC)	Significant	CHR
GTSE1	0.37	-0.26	0.09	0.22	0.23	0.00	-0.13	0.17	-0.06	0.20	-0.16	-0.14	0.11	-0.21	-0.24	0.05	0.31	-0.55	-0.87	Stable	5.00
TTC38	0.32	0.31	0.38	0.61	0.27	0.55	-0.03	0.17	0.19	0.61	0.39	-0.03	0.24	-0.13	0.25	0.38	0.07	0.48	-1.06	Stable	5.00
CDPF1	0.02	0.00	0.00	0.19	0.00	0.00	0.36	0.39	0.19	0.00	0.00	0.17	0.19	0.37	0.00	0.17	0.33	1.82	0.87	Stable	5.00
SCUBE1	0.47	0.09	0.25	0.94	0.67	0.82	0.68	0.27	0.42	0.78	0.28	0.55	0.57	0.35	0.06	0.95	0.00	0.47	-1.10	Down	5.00
MCAT	-0.89	-0.67	-0.64	-0.81	-0.46	-0.56	-1.09	-0.74	-0.67	-1.06	-0.89	-0.68	-0.64	-0.92	-0.52	-0.42	0.46	1.11	0.15	Stable	5.00
TSPO	0.13	-0.27	-0.43	-0.16	-0.13	-0.16	0.14	0.17	0.16	-0.15	-0.22	-0.36	-0.14	0.14	-0.73	-0.39	0.35	0.41	-1.28	Stable	5.00
TTLL12	-0.28	-0.80	-0.70	-0.03	-0.37	-0.31	-0.04	0.11	0.25	0.14	-0.20	-0.23	-0.10	-0.06	-0.32	-0.21	0.66	1.40	0.49	Stable	5.00
TTLL1	0.21	0.28	0.48	0.37	0.33	0.27	0.32	0.42	0.56	0.44	0.44	0.42	0.39	0.24	0.67	0.23	0.58	1.10	0.14	Stable	5.00
NDUFA6	-0.14	-0.28	-0.28	-0.28	-0.28	-0.28	-0.14	-0.11	-0.28	-0.28	-0.28	-0.28	-0.14	-0.14	-0.28	-0.28	0.12	0.79	-0.35	Stable	5.00
PMM1	0.46	0.00	0.00	0.01	0.00	0.00	0.88	0.46	0.00	0.01	0.00	0.02	0.00	0.51	0.01	0.05	0.05	25.45	4.67	Stable	5.00
POLR3H	-0.35	0.22	-0.36	-0.22	-0.44	0.00	0.19	-0.28	-0.39	0.06	-0.35	-0.01	-0.35	0.28	-0.48	-0.12	0.81	0.82	-0.29	Stable	5.00
ACO2	0.09	0.13	-0.12	-0.13	-0.16	-0.13	0.27	0.26	0.23	0.15	-0.13	-0.05	-0.16	0.27	-0.02	0.23	0.03	-2.95	1.56	Up	5.00
ENSSSCG00000000073	0.18	0.15	0.12	0.27	0.30	0.32	0.17	0.17	0.19	0.25	0.33	0.04	0.33	0.17	0.14	0.15	0.05	0.65	-0.63	Stable	5.00
CACNA1I	-0.52	-0.74	-0.32	-0.55	0.44	0.06	-0.90	-1.12	-1.05	-1.32	-0.06	0.04	0.13	-0.92	-0.42	-0.02	0.02	4.65	2.22	Up	5.00
TAB1	0.00	-0.09	0.12	0.32	0.19	0.20	-0.07	0.08	0.12	0.20	0.34	-0.13	0.43	-0.14	-0.11	-0.08	0.03	-0.07	-3.76	Down	5.00
DMC1	0.88	0.34	0.62	0.71	0.97	0.51	0.75	0.40	0.47	0.71	0.53	1.11	0.80	0.87	0.33	1.10	0.08	0.73	-0.46	Stable	5.00
PLA2G6	-0.25	-0.08	-0.35	-0.22	-0.34	-0.44	-0.12	-0.12	-0.04	-0.34	-0.30	-0.26	-0.22	-0.16	-0.05	-0.23	0.01	0.51	-0.99	Stable	5.00
BAIAP2L2	0.18	0.18	0.04	0.04	0.14	0.08	0.02	0.15	0.25	0.18	0.04	0.11	0.12	0.06	0.11	0.13	0.62	1.16	0.22	Stable	5.00
EIF3L	0.16	-0.66	-0.53	-0.11	-0.26	-0.29	-0.12	0.24	-0.03	-0.11	-0.20	-0.17	-0.18	0.13	-0.27	-0.22	0.65	0.71	-0.50	Stable	5.00
TEX33	0.31	0.11	0.48	0.18	0.07	0.24	-0.55	0.44	0.20	0.01	0.74	-0.15	0.19	0.38	0.07	-0.35	0.70	1.55	0.63	Stable	5.00
TST	-0.08	-0.01	0.52	0.21	-0.37	-0.22	-0.85	-0.08	-0.02	-0.16	0.25	-0.12	0.04	0.04	0.04	-0.78	0.62	0.38	-1.41	Stable	5.00
KCTD17	-0.07	-0.14	-0.39	-0.46	-0.34	-0.30	0.02	0.19	0.06	-0.39	0.29	-0.12	-0.05	0.10	-0.17	0.19	0.41	0.33	-1.59	Stable	5.00
EIF3D	-0.26	0.34	0.40	0.08	-0.20	-0.36	0.06	1.13	0.42	0.08	0.22	-0.09	0.27	0.55	-0.11	0.20	0.12	12.93	3.69	Stable	5.00
FOXRED2	-0.57	-0.08	-0.25	-0.43	-0.28	0.04	-0.48	-0.63	-0.34	-0.46	-0.18	-0.17	-0.12	-0.31	-0.33	-0.06	0.08	1.80	0.85	Stable	5.00
FBXO7	-0.56	-0.55	-0.60	-0.60	-0.36	-0.39	-0.54	-1.00	-0.64	-0.60	0.00	-0.40	-0.27	-0.49	-0.37	-0.18	0.02	1.70	0.76	Stable	5.00
BTBD11	0.00	0.18	0.12	-0.31	0.16	0.23	0.00	0.02	-0.05	-0.31	0.18	0.14	0.20	-0.14	-0.02	0.01	0.79	0.37	-1.42	Stable	5.00
ADCY6	-0.33	0.10	-0.34	-0.44	-0.55	-0.12	-0.33	0.44	0.28	-0.47	-0.13	0.03	-0.04	0.35	-0.13	-0.32	0.09	-0.02	-6.03	Stable	5.00
TUBA1B	-1.17	-0.43	-0.64	-0.42	-1.12	-0.69	-1.17	-0.51	-0.34	-0.55	-0.61	-0.74	-0.94	-0.26	-0.74	-0.84	0.59	0.89	-0.17	Stable	5.00
ENSSSCG00000000194	0.60	0.40	0.57	0.07	0.42	0.34	0.60	0.15	0.11	0.18	0.24	0.25	0.29	0.23	0.06	0.35	0.45	1.27	0.34	Stable	5.00
SMARCD1	0.59	-0.06	0.18	0.59	0.18	-0.06	0.59	0.21	0.21	0.27	0.44	0.27	-0.06	-0.06	-0.06	0.59	0.57	0.73	-0.46	Stable	5.00
ASIC1	-0.28	0.20	-0.07	-0.28	0.04	0.00	-0.28	0.34	-0.06	-0.08	-0.06	-0.31	0.19	0.00	-0.20	-0.20	0.65	0.48	-1.06	Stable	5.00
SCN8A	0.13	0.43	0.34	0.13	0.13	0.37	0.13	0.29	0.45	0.13	0.01	0.14	0.13	-0.05	0.02	0.13	0.38	1.45	0.54	Stable	5.00
KRT18	0.05	0.60	0.20	0.21	-0.11	0.19	-0.15	0.09	0.18	0.41	0.29	0.21	0.30	0.35	0.39	0.30	0.90	0.94	-0.08	Stable	5.00
SOAT2	-0.12	0.42	0.27	-0.05	0.16	0.51	0.01	-0.07	-0.11	-0.37	-0.57	-0.41	0.04	-0.22	-0.05	-0.18	0.41	-0.15	-2.71	Stable	5.00
ESPL1	0.00	-0.08	-0.08	-0.13	-0.15	-0.04	-0.04	0.00	-0.04	-0.24	-0.43	-0.12	-0.13	0.00	-0.08	-0.16	0.01	0.22	-2.18	Down	5.00
AMHR2	-0.29	-0.10	-0.14	-0.40	-0.21	-0.15	-0.27	0.06	-0.27	-0.55	-0.45	-0.55	-0.41	-0.24	-0.28	-0.44	0.01	0.48	-1.06	Down	5.00
PCBP2	0.51	0.03	0.25	0.24	0.72	0.09	0.74	0.60	0.29	0.32	0.07	0.32	0.28	0.47	0.01	0.61	0.81	1.09	0.12	Stable	5.00
COPZ1	-0.35	-0.02	-0.19	-0.35	-0.17	-0.10	-0.12	0.10	-0.25	-0.11	-0.47	-0.21	-0.33	0.03	-0.48	-0.13	0.40	0.69	-0.54	Stable	5.00
RDH5	-0.52	-0.72	-0.29	-0.95	-0.56	-0.24	-0.44	-0.53	-0.24	-0.84	-0.62	-0.81	-0.83	-0.74	0.60	-0.71	0.08	0.52	-0.95	Stable	5.00
SMARCC2	0.07	0.07	0.04	0.06	0.07	0.08	0.05	0.08	0.04	0.06	0.03	0.06	0.07	0.06	0.00	0.02	0.64	0.90	-0.15	Stable	5.00
ENSSSCG00000000394	-0.24	-0.08	-0.02	-0.04	-0.25	-0.46	-0.20	-0.03	-0.09	-0.05	-0.01	-0.05	-0.04	-0.04	0.04	-0.03	0.62	0.71	-0.49	Stable	5.00
TIMELESS	0.15	1.11	0.04	0.42	0.25	-0.06	0.00	-0.01	0.79	0.59	0.60	0.59	0.42	0.29	0.95	0.37	0.93	1.04	0.05	Stable	5.00
RDH16	0.96	0.43	-0.56	0.56	0.82	0.34	0.50	-0.56	0.64	1.58	0.92	1.58	0.34	0.18	1.27	1.21	0.08	0.39	-1.37	Stable	5.00

USP15	-0.23	0.00	0.02	-0.23	-0.23	-0.23	-0.09	-0.23	0.12	-0.23	-0.23	-0.23	-0.23	-0.23	0.05	0.03	0.06	0.37	-1.42	Stable	5.00
IRAK3	0.01	0.01	0.01	0.00	0.33	0.28	0.01	0.83	0.68	0.89	0.00	0.00	0.00	0.29	1.02	0.00	0.38	1.92	0.94	Stable	5.00
NUP107	0.01	0.19	0.13	-0.05	0.10	0.13	0.01	0.39	0.15	0.02	0.08	-0.03	-0.05	0.04	0.16	-0.03	0.05	6.16	2.62	Up	5.00
SLC35E3	0.28	0.84	0.93	0.21	0.53	0.56	0.31	1.77	0.07	0.14	0.27	0.21	0.21	0.94	0.78	0.13	0.05	2.62	1.39	Up	5.00
PTPRB	0.48	0.26	0.82	0.89	0.53	0.25	1.03	-0.41	0.30	0.89	0.53	0.94	0.94	0.21	-0.11	0.84	0.05	0.44	-1.18	Stable	5.00
PKP2	0.41	0.00	0.00	0.39	0.41	0.00	0.41	0.48	0.41	0.24	0.00	0.00	0.24	0.24	0.48	0.00	0.16	1.89	0.92	Stable	5.00
BICD1	0.06	0.38	0.35	0.76	0.68	0.28	0.45	0.22	0.52	0.66	0.55	0.75	0.70	0.01	0.22	0.67	0.00	0.44	-1.20	Down	5.00
KLHL42	0.42	0.23	0.23	0.50	0.50	0.27	0.47	0.51	0.49	0.53	0.50	0.53	0.50	0.46	0.51	0.53	0.21	0.86	-0.22	Stable	5.00
ARNTL2	-0.20	-0.42	-0.45	-0.15	0.63	0.13	-0.09	-0.10	-0.06	0.45	0.45	0.66	-0.15	-0.08	-0.18	0.53	0.00	-0.62	-0.70	Stable	5.00
FGFR1OP2	0.00	0.00	0.00	0.00	0.25	0.03	0.00	0.00	0.00	0.25	0.27	0.25	0.00	0.00	0.00	0.25	0.01	0.00	#NAME?	Down	5.00
ENSSSCG00000000576	-0.47	-0.23	-0.22	-0.37	-0.37	-0.32	-0.47	-0.08	-0.02	-0.26	-0.32	-0.32	-0.37	-0.14	0.20	-0.36	0.09	0.53	-0.90	Stable	5.00
GYS2	-0.40	-0.34	-0.20	0.13	0.13	0.01	-0.40	-0.27	0.04	0.19	0.06	-0.04	0.13	-0.23	0.21	-0.04	0.01	-2.79	1.48	Up	5.00
SLCO1A2	1.57	0.83	0.63	-0.71	-0.62	-0.08	1.65	0.75	-0.39	-0.56	-0.56	0.51	-0.52	0.92	0.00	0.45	0.01	-2.86	1.52	Up	5.00
PIK3C2G	-0.38	-0.14	-0.14	-0.40	-0.40	-0.21	-0.34	-0.14	-0.20	-0.40	-0.36	-0.32	-0.40	-0.14	0.00	-0.34	0.01	0.52	-0.93	Stable	5.00
ERP27	0.34	-0.17	-0.17	-0.23	-0.35	0.00	0.45	0.00	0.22	0.45	0.45	0.22	-0.23	0.00	0.00	0.00	0.74	2.24	1.16	Stable	5.00
YBX3	0.09	0.03	0.03	0.20	0.20	0.32	0.09	0.36	0.45	0.20	0.29	0.10	0.20	0.15	0.35	0.11	0.89	0.95	-0.07	Stable	5.00
GABARAPL1	0.74	0.71	0.68	0.13	0.23	0.51	0.79	0.63	0.55	0.33	0.40	0.50	0.23	0.74	0.34	0.49	0.00	1.84	0.88	Stable	5.00
OLR1	1.24	1.00	1.00	0.19	0.25	0.60	0.95	1.02	0.58	0.37	0.83	0.79	0.16	1.06	0.53	1.14	0.03	1.71	0.77	Stable	5.00
CLEC7A	0.65	0.19	0.17	-0.12	0.06	0.61	0.69	0.39	0.33	0.25	0.38	0.29	0.00	0.55	0.25	0.78	0.37	1.43	0.51	Stable	5.00
CLEC1A	-0.19	-0.45	-0.45	-0.12	-0.07	-0.10	-0.19	-0.41	-0.41	-0.01	-0.09	-0.13	-0.11	-0.37	-0.52	-0.13	0.00	3.85	1.94	Up	5.00
ENSSSCG00000000659	0.34	0.27	0.27	-0.16	-0.08	0.11	0.38	0.26	0.10	0.00	-0.09	0.09	-0.07	0.04	-0.31	0.15	0.08	-25.21	4.66	Stable	5.00
A2M	0.07	0.37	0.14	0.07	0.07	-0.08	0.37	0.14	-0.06	0.07	-0.16	0.07	0.07	0.49	0.06	0.12	0.05	7.14	2.84	Up	5.00
P3H3	-0.16	-0.62	-0.60	-0.08	-0.08	0.04	-0.11	-0.41	-0.24	-0.08	0.05	-0.27	-0.08	-0.37	-0.47	-0.21	0.00	4.12	2.04	Up	5.00
GNB3	0.25	-0.13	-0.13	0.32	0.32	0.31	-0.06	-0.05	0.17	0.32	0.17	0.10	0.32	-0.05	0.00	0.10	0.00	0.01	-7.52	Down	5.00
TPI1	0.52	0.58	0.58	0.49	0.49	0.49	0.52	0.64	0.24	0.49	0.55	0.58	0.49	0.06	0.28	0.58	0.26	0.83	-0.27	Stable	5.00
CD4	-0.03	-0.03	-0.03	0.19	0.19	0.25	0.07	-0.05	0.17	0.19	0.03	-0.01	0.19	-0.03	0.21	-0.06	0.13	0.30	-1.74	Stable	5.00
IFFO1	-0.31	-0.33	-0.18	0.32	0.32	0.22	-0.25	0.20	0.03	0.32	0.40	0.11	0.32	0.12	0.12	0.52	0.00	-0.23	-2.10	Down	5.00
CD27	0.24	0.51	0.39	0.29	0.29	0.42	0.34	0.31	0.14	0.29	0.14	0.38	0.29	0.09	0.00	0.38	0.42	0.82	-0.29	Stable	5.00
AKAP3	0.10	0.19	0.17	0.13	0.21	0.21	0.10	0.06	0.35	0.08	0.19	0.27	0.27	0.19	0.00	0.27	0.22	0.72	-0.48	Stable	5.00
DYRK4	-0.13	-0.03	-0.03	-0.41	-0.37	-0.37	-0.19	-0.21	-0.24	-0.37	-0.37	-0.17	-0.37	-0.01	0.00	-0.23	0.00	0.31	-1.68	Down	5.00
RHNO1	0.01	-0.46	-0.26	-0.28	0.43	0.30	-0.22	0.09	0.37	0.42	1.16	-0.12	0.36	-0.29	-0.49	0.46	0.02	-0.45	-1.14	Down	5.00
NRIP2	0.25	0.24	0.26	0.10	-0.02	-0.04	0.30	-0.02	-0.04	-0.02	0.04	0.30	-0.06	0.67	0.71	0.18	0.05	4.99	2.32	Stable	5.00
DDX11	0.00	-0.05	-0.05	0.00	-0.01	-0.01	0.00	0.00	-0.01	-0.01	0.66	0.00	-0.01	0.00	-0.02	0.32	0.17	-0.13	-2.90	Stable	5.00
WASHC1	-0.05	-0.47	-0.26	-0.04	-0.53	-0.65	0.06	-0.29	-0.78	-0.65	0.08	-0.02	-0.68	-0.09	-0.31	-0.17	0.69	0.82	-0.29	Stable	5.00
CNTN1	0.67	0.10	0.40	1.01	0.82	0.82	0.64	0.82	0.82	0.82	0.99	0.70	0.82	0.58	0.69	0.67	0.03	0.71	-0.50	Stable	5.00
ADAMTS20	0.10	1.46	0.33	0.83	0.94	1.44	1.36	0.95	1.24	1.35	1.18	1.42	1.48	1.29	1.47	0.96	0.42	0.85	-0.23	Stable	5.00
IRAK4	0.51	0.51	0.33	0.34	1.29	0.79	0.84	0.73	0.10	1.00	0.96	0.14	0.90	-0.25	1.26	0.51	0.28	0.68	-0.55	Stable	5.00
PUS7L	0.17	0.26	0.00	0.35	0.25	0.60	0.11	0.17	0.21	0.51	0.65	0.22	0.60	0.00	0.52	0.13	0.03	0.44	-1.19	Down	5.00
NELL2	-0.60	-0.30	-0.54	-0.37	0.19	-0.66	-0.16	-0.47	-0.32	-0.11	-0.16	-0.49	-0.51	-0.66	-0.30	-0.39	0.36	1.35	0.43	Stable	5.00
ANO6	0.20	0.80	0.31	1.23	0.35	0.67	0.75	0.20	1.19	0.85	1.31	1.24	0.43	0.95	1.53	0.77	0.60	0.86	-0.21	Stable	5.00
AMIGO2	-1.40	-0.90	-0.88	-0.02	-0.07	0.19	-0.93	-0.87	0.06	0.07	0.88	-0.27	0.02	-1.35	-0.85	-1.03	0.00	31.63	4.98	Up	5.00
SENP1	0.26	0.53	0.50	0.04	0.21	0.25	0.43	0.42	0.21	0.15	0.12	0.18	0.50	0.66	0.57	1.83	0.86	1.10	0.13	Stable	5.00
SLC41A2	0.33	0.16	0.44	0.28	0.16	0.06	0.40	0.04	-0.04	0.06	0.21	0.38	0.06	0.36	0.24	0.72	1.00	1.00	0.00	Stable	5.00
WASHC4	0.64	0.28	0.33	0.25	0.38	0.32	0.54	0.19	-0.09	0.32	0.62	0.84	0.32	0.49	0.71	0.40	0.70	0.90	-0.16	Stable	5.00
NFYB	-0.23	-0.22	-0.23	-0.24	-0.12	-0.12	-0.23	-0.25	-0.12	-0.12	-0.25	-0.23	-0.12	-0.23	0.25	-0.23	0.75	0.88	-0.18	Stable	5.00

HCFC2	0.09	0.10	0.09	0.01	0.02	0.02	0.00	0.00	0.00	0.02	0.03	0.11	0.02	0.00	0.28	0.21	0.71	1.30	0.38	Stable	5.00
CHPT1	0.00	0.00	0.01	0.01	0.14	0.14	0.00	0.00	0.14	0.14	0.00	0.00	0.14	0.00	0.50	0.23	0.80	0.82	-0.29	Stable	5.00
SPIC	-0.23	-0.81	-0.62	-1.09	-1.39	-1.46	-0.70	-0.43	-0.84	-0.43	-0.67	-1.29	-1.42	-0.33	-1.10	-0.56	0.04	0.61	-0.71	Stable	5.00
ANO4	0.11	-0.06	-0.11	0.20	0.20	0.26	0.22	0.24	0.30	0.70	0.25	0.11	0.17	0.03	0.35	-0.22	0.49	0.64	-0.64	Stable	5.00
APAF1	-0.25	0.51	0.28	-0.08	-0.08	-0.43	0.14	0.75	0.14	-0.52	0.30	-0.08	-0.08	0.15	0.79	-0.78	0.01	-1.45	0.54	Stable	5.00
NTN4	0.12	0.01	0.46	0.22	0.38	0.25	0.31	0.12	0.12	0.12	0.06	0.25	0.02	0.46	0.06	0.07	0.64	1.22	0.28	Stable	5.00
FGD6	0.18	0.06	-0.07	-0.03	-0.11	0.15	-0.01	0.09	0.27	0.25	0.05	-0.07	0.02	-0.07	-0.01	0.30	0.83	0.79	-0.35	Stable	5.00
ENSSSCG00000000905	0.12	-0.11	-0.16	-0.46	-0.14	-0.47	-0.17	0.57	-0.30	-0.48	-0.40	-0.78	-0.07	-0.31	-0.18	-0.49	0.02	0.17	-2.59	Down	5.00
EPYC	-0.58	-0.49	0.03	-0.22	-0.18	-0.58	-0.58	-0.58	-0.19	-0.40	-0.54	-0.42	-0.16	-0.52	-0.58	-0.20	0.35	1.29	0.37	Stable	5.00
SBF1	0.08	0.17	0.08	0.00	0.00	0.08	0.00	0.17	0.08	-0.09	0.00	0.08	0.00	0.17	0.00	0.00	0.02	10.14	3.34	Up	5.00
ALG12	1.71	1.58	1.70	1.33	0.83	0.81	0.33	1.58	0.81	1.40	1.02	0.33	0.50	0.50	1.36	1.12	0.26	1.31	0.38	Stable	5.00
ENSSSCG00000000982	-0.59	-0.97	-0.77	-0.26	-1.49	-0.53	-1.10	-0.98	-0.74	-1.13	-0.66	-0.93	-0.56	-0.65	0.21	-1.04	0.54	0.85	-0.24	Stable	5.00
EXOC2	0.00	-0.66	-0.12	-0.25	-0.46	-0.74	0.00	-0.34	-0.17	0.00	-0.46	-0.55	-0.25	-0.67	0.00	-0.70	0.20	0.57	-0.80	Stable	7.00
RPP40	-0.76	-1.21	-1.20	-0.87	-0.63	-0.54	-0.30	-1.00	-1.17	-1.01	-0.36	-0.95	-0.89	-1.01	0.74	-1.07	0.85	0.94	-0.09	Stable	7.00
ECI2	0.27	-0.20	0.04	-0.68	0.28	-0.28	0.42	-0.21	0.15	-0.20	-0.01	-0.15	-0.21	-0.52	-0.25	-0.44	0.26	0.17	-2.52	Stable	7.00
PRPF4B	-0.11	0.00	0.00	0.00	-0.04	0.00	-0.11	-0.06	-0.06	-0.06	-0.06	-0.11	-0.06	-0.06	-0.11	-0.06	0.46	1.34	0.42	Stable	7.00
BPHL	0.29	0.09	0.11	0.05	-0.15	0.20	0.41	-0.40	0.04	0.02	0.09	-0.28	0.01	-0.38	0.46	-0.22	0.41	-2.27	1.18	Stable	7.00
RIOK1	-0.13	-0.34	0.65	0.16	0.12	-0.43	-0.14	-0.67	-0.34	0.34	-0.21	0.23	0.70	-0.18	-0.06	-0.03	0.17	-1.36	0.45	Stable	7.00
ENSSSCG00000001038	-0.34	0.18	0.05	0.00	-0.28	0.10	-0.32	-0.28	0.04	-0.07	0.06	0.20	-0.18	0.00	-0.33	0.06	0.25	10.89	3.44	Stable	7.00
PAK1IP1	0.48	0.25	0.81	0.25	0.00	0.25	0.47	-0.27	0.39	0.11	0.25	0.58	0.24	0.19	0.00	0.25	0.71	1.21	0.27	Stable	7.00
MAK	0.33	0.00	0.26	0.08	0.00	0.08	0.33	0.08	0.08	0.00	0.08	0.26	0.15	0.08	0.08	0.00	0.22	1.92	0.94	Stable	7.00
TBC1D7	0.10	0.00	0.00	0.38	0.34	0.34	0.19	0.19	0.00	0.19	0.31	0.34	0.34	0.30	0.50	0.21	0.05	0.52	-0.94	Stable	7.00
ENSSSCG00000001088	0.19	0.08	0.07	0.30	0.30	-0.07	-0.13	0.40	0.28	0.58	-0.13	0.59	0.31	0.49	0.31	0.33	0.57	0.76	-0.40	Stable	7.00
GPLD1	0.21	0.89	0.38	0.64	0.93	-0.24	0.76	0.96	0.55	0.60	0.51	0.23	0.50	0.15	0.55	0.58	0.61	1.18	0.24	Stable	7.00
ALDH5A1	-0.76	-0.85	-0.82	-0.53	-0.55	-0.52	-1.37	-0.56	-0.50	-0.17	-0.68	-0.12	-0.47	0.03	-0.22	-0.21	0.21	1.55	0.64	Stable	7.00
TDP2	0.00	-0.04	0.00	-0.04	-0.04	-0.04	0.00	-0.08	-0.04	0.00	-0.08	0.00	-0.04	0.00	-0.04	-0.04	0.48	0.71	-0.49	Stable	7.00
BTN2A2	-0.07	-0.48	-0.54	-0.67	-0.27	-0.68	-0.12	-0.27	-0.16	-0.55	-0.49	-0.13	-0.33	-0.63	-0.33	-0.35	0.30	0.75	-0.42	Stable	7.00
ZNF184	0.01	-0.11	-0.04	0.01	0.11	0.10	-0.11	0.06	-0.07	0.02	0.05	0.12	0.03	0.14	0.11	0.05	0.12	-0.06	-4.00	Stable	7.00
ZSCAN9	0.50	0.56	0.67	0.32	0.25	0.40	0.87	-0.10	0.44	-0.02	0.32	0.15	0.19	0.28	0.24	0.31	0.13	1.80	0.85	Stable	7.00
ENSSSCG00000001229	0.20	-0.36	0.14	-0.27	-0.02	-0.39	-0.38	-0.44	-0.52	0.13	-0.55	-0.05	-0.28	0.17	-0.26	-0.55	0.64	0.73	-0.46	Stable	7.00
DHX16	0.09	-0.21	-0.14	-0.13	-0.01	0.12	-0.29	0.02	-0.20	0.19	-0.04	0.16	0.09	0.21	0.02	0.18	0.10	-0.94	-0.09	Stable	7.00
VARS2	-0.70	-0.22	-0.32	-0.28	-0.32	-0.06	-0.67	0.14	-0.09	-0.02	0.05	-0.33	-0.11	-0.78	-0.25	-0.02	0.11	2.65	1.41	Stable	7.00
ENSSSCG00000001395	0.08	0.00	-0.26	-0.29	-0.02	-0.03	0.06	0.13	0.17	0.23	-0.31	0.20	0.11	0.13	0.01	0.06	0.60	-5.37	2.43	Stable	7.00
ENSSSCG00000001396	0.09	0.27	-0.29	0.40	0.52	0.47	-0.02	0.02	-0.28	-0.49	0.33	-0.11	0.58	0.52	0.52	0.70	0.29	0.34	-1.55	Stable	7.00
ENSSSCG00000001398	0.61	0.30	0.06	0.27	0.65	0.19	0.25	0.45	0.59	0.90	0.21	0.93	0.36	0.75	0.65	0.39	0.83	0.94	-0.09	Stable	7.00
ATP6V1G2-DDX39B	-0.21	-0.09	0.07	-0.23	-0.37	-0.12	0.08	-0.09	0.04	-0.31	-0.16	-0.28	-0.06	-0.30	-0.45	-0.17	0.26	0.57	-0.82	Stable	7.00
LTA	0.07	0.10	0.08	0.03	0.07	0.09	0.13	0.11	0.14	0.09	0.09	0.11	0.12	0.07	0.07	0.11	0.62	1.08	0.11	Stable	7.00
SLC44A4	-0.06	0.13	-0.06	0.22	-0.13	-0.21	0.29	-0.08	-0.01	-0.12	-0.11	-0.27	-0.05	-0.04	-0.19	-0.06	0.24	0.01	-6.40	Stable	7.00
ZBTB12	0.13	0.65	0.77	1.62	1.00	1.29	-0.40	1.00	-0.27	0.63	1.42	0.56	1.47	0.94	0.99	1.66	0.01	0.39	-1.34	Down	7.00
C2	-0.18	-0.30	-0.09	-0.25	-0.25	-0.11	-0.23	0.03	-0.06	-0.04	0.01	0.05	-0.32	-0.11	-0.12	-0.25	0.84	0.91	-0.13	Stable	7.00
SKIV2L	-0.39	-0.15	0.09	-0.38	-0.17	-0.42	-0.06	-0.29	-0.19	-0.15	-0.30	-0.37	-0.39	-0.39	-0.29	-0.39	0.14	0.65	-0.61	Stable	7.00
DXO	-0.33	0.04	0.12	-0.03	0.52	-0.18	-0.35	0.80	-0.07	0.44	0.66	-0.04	0.14	0.42	0.39	0.23	0.61	0.58	-0.79	Stable	7.00
ATF6B	0.03	0.28	0.14	0.39	0.28	0.22	0.30	0.06	0.18	0.01	0.23	0.05	-0.01	0.06	0.15	0.10	0.92	0.96	-0.06	Stable	7.00
ENSSSCG00000001433	-0.11	0.20	0.23	0.39	0.21	-0.11	-0.11	0.92	0.39	0.23	0.37	0.46	0.44	0.25	0.60	0.24	0.91	1.06	0.08	Stable	7.00
RNF5	-1.29	-1.48	-1.25	-1.34	-1.09	-1.27	-1.47	-1.11	-1.29	-1.00	-0.79	-1.04	-0.90	-1.09	-1.34	-1.19	0.02	1.20	0.26	Stable	7.00

ENSSSCG00000001455	-1.26	-1.30	-1.51	-0.53	-0.88	-1.29	-1.30	-0.67	-1.18	-1.01	-1.31	-1.14	-1.23	-1.16	-0.88	-0.98	0.41	1.11	0.14	Stable	7.00
ENSSSCG00000001456	-0.93	-1.29	-1.44	-0.76	-1.30	-1.55	-1.01	-1.48	-1.01	-0.98	-1.54	-0.66	-1.81	-0.93	-1.46	-1.54	0.68	0.94	-0.09	Stable	7.00
SLA-DQB1	-0.44	-0.56	-0.93	-0.51	-0.58	-0.96	-0.43	-0.54	-0.43	-0.40	-0.60	-0.44	-0.93	-0.58	-0.63	-0.94	0.33	0.85	-0.24	Stable	7.00
ENSSSCG00000001458	-0.62	-0.15	-0.85	-0.25	0.39	-0.44	-0.47	-0.25	-0.62	-0.87	-0.02	-0.53	0.03	-0.12	-0.13	0.22	0.24	2.18	1.12	Stable	7.00
HLA-DOB	-0.17	0.17	0.05	0.14	-0.10	0.12	0.15	0.21	0.23	0.36	0.06	-0.01	0.16	0.05	-0.09	0.10	0.72	0.75	-0.41	Stable	7.00
SLA-DMA	0.35	0.14	-0.11	-0.30	-0.09	-0.44	0.43	0.05	0.43	-0.09	-0.38	0.43	-0.52	-0.01	-0.39	-0.52	0.04	-0.47	-1.10	Down	7.00
SLC39A7	0.02	-0.02	-0.02	-0.03	0.09	-0.03	0.00	-0.02	0.00	-0.03	-0.08	0.30	0.04	0.06	0.04	0.08	0.45	0.18	-2.51	Stable	7.00
MLIP	-0.14	-0.14	-0.07	-0.14	-0.14	-0.07	-0.14	-0.14	-0.14	-0.14	-0.14	-0.14	-0.07	-0.14	-0.14	-0.07	0.28	1.15	0.21	Stable	7.00
LRRC1	0.00	0.00	0.00	0.00	0.12	0.00	0.00	0.00	0.00	0.00	0.00	0.00	0.00	0.12	0.12	0.00	0.55	2.00	1.00	Stable	7.00
GCLC	0.37	0.35	0.17	0.36	0.53	0.17	0.35	0.35	0.35	0.35	0.35	0.36	0.14	0.54	0.53	0.03	0.22	1.31	0.39	Stable	7.00
VPS52	0.17	0.07	-0.08	-0.46	0.13	-0.52	0.23	-0.03	-0.17	0.05	-0.67	0.57	-0.63	0.12	0.00	-0.61	0.11	-0.14	-2.80	Stable	7.00
RPS18	0.51	0.14	0.26	0.20	0.23	0.19	0.20	0.09	0.49	0.07	0.30	0.27	0.21	0.53	0.04	0.21	0.36	1.35	0.43	Stable	7.00
ITPR3	0.20	-0.09	0.07	-0.14	0.15	0.15	-0.14	-0.05	0.02	-0.23	0.08	0.16	-0.10	0.24	0.14	-0.12	0.44	-6.30	2.66	Stable	7.00
TCP11	-0.31	-0.08	0.52	0.10	-0.12	0.47	-0.04	-0.16	-0.33	-0.43	0.61	-0.07	0.15	-0.52	-0.23	-0.03	0.18	-1.66	0.73	Stable	7.00
RPL10A	-0.22	-0.41	-0.50	-0.44	-0.32	-0.20	-0.38	-0.45	-0.29	-0.11	-0.35	-0.28	-0.17	-0.21	-0.01	-0.16	0.45	1.21	0.28	Stable	7.00
CLPS	0.09	-0.08	-0.04	0.11	0.19	0.11	-0.23	-0.10	-0.18	-0.21	0.08	0.22	0.07	0.16	0.07	0.11	0.08	-0.46	-1.13	Stable	7.00
LHFPL5	-0.45	-0.17	-0.17	0.00	-0.17	0.00	-0.35	-0.17	-0.47	-0.17	0.00	-0.35	0.00	-0.45	0.00	0.00	0.03	3.21	1.68	Up	7.00
BNIP5	-0.23	-0.31	-0.08	-0.31	0.04	-0.16	-0.31	-0.31	-0.27	-0.12	-0.12	-0.12	-0.31	-0.08	-0.31	-0.31	0.29	1.36	0.44	Stable	7.00
ETV7	0.11	0.16	-0.06	0.22	0.22	0.04	0.20	0.15	0.15	0.11	-0.04	0.18	0.21	0.16	0.31	0.23	0.97	1.01	0.02	Stable	7.00
CPNE5	-0.25	-0.05	-0.14	-0.03	0.08	-0.36	0.50	-0.02	0.01	0.04	-0.03	-0.07	0.46	-0.26	0.03	0.24	0.61	-0.53	-0.93	Stable	7.00
ENSSSCG00000001612	-0.45	-0.90	-0.85	-0.42	-0.18	-0.71	-0.62	-0.82	-0.99	-0.79	-0.49	-0.42	-0.45	-0.61	-0.24	-0.47	0.11	1.39	0.47	Stable	7.00
MDFI	-0.02	-0.16	-0.18	0.16	0.07	-0.17	-0.29	-0.07	-0.20	-0.18	0.06	-0.17	0.01	-0.02	0.08	-0.04	0.25	3.33	1.74	Stable	7.00
GUCA1A	0.05	0.05	0.15	0.02	0.00	0.07	0.07	0.07	0.10	0.07	0.07	0.10	0.00	0.05	0.00	0.00	0.27	1.57	0.65	Stable	7.00
PEX6	-0.49	-0.25	-0.52	-0.12	-0.24	-1.01	-1.19	-1.16	-0.85	-0.60	-0.56	-0.47	-0.50	-0.60	-0.95	-0.66	0.15	1.44	0.53	Stable	7.00
RRP36	-0.27	-0.41	-0.43	-0.22	-0.08	-0.45	-0.81	-0.78	-0.41	-0.15	-0.59	-0.37	0.00	-0.13	-0.46	-0.16	0.07	1.85	0.88	Stable	7.00
TJAP1	-0.50	-0.51	-0.45	-0.56	-0.55	-0.27	-0.25	-0.17	-0.40	-0.37	-0.48	-0.50	-0.39	-0.51	-0.19	-0.28	0.44	0.88	-0.19	Stable	7.00
CAPN11	0.09	-0.09	-0.02	-0.05	0.05	-0.39	-0.05	-0.01	0.02	0.06	-0.34	0.02	0.02	0.05	-0.70	0.02	0.92	1.16	0.21	Stable	7.00
SLC29A1	0.05	0.12	0.05	0.17	0.10	-0.04	-0.12	-0.10	0.04	0.01	0.12	0.05	0.10	0.10	0.10	0.10	0.27	0.40	-1.31	Stable	7.00
AARS2	-0.27	-0.39	-0.42	-0.57	-0.57	-0.57	-0.27	-0.43	-0.33	-0.17	-0.57	-0.45	-0.57	-0.51	-0.58	-0.50	0.15	0.81	-0.31	Stable	7.00
CYP39A1	-0.20	-0.25	-0.30	-0.25	-0.31	-0.34	-0.20	-0.05	-0.18	-0.21	-0.30	-0.20	-0.35	-0.24	-0.28	-0.35	0.05	0.73	-0.45	Stable	7.00
SLC25A27	-0.23	-0.50	0.15	-0.50	-0.56	-0.37	-0.17	0.04	-0.12	-0.06	-0.59	-0.19	-0.44	-0.56	-0.31	-0.31	0.15	0.56	-0.83	Stable	7.00
PLA2G7	-0.26	-0.39	0.19	-0.45	-0.46	0.11	-0.38	0.03	-0.04	-0.45	-0.21	-0.22	-0.40	-0.26	-0.56	-0.42	0.36	0.66	-0.59	Stable	7.00
ENSSSCG00000001724	-0.15	-0.73	0.93	-0.62	-0.80	-0.65	0.49	0.67	0.92	0.81	-0.66	0.23	-1.10	-0.71	-0.80	-0.98	0.14	-0.16	-2.61	Stable	7.00
CENPQ	-0.39	0.03	-0.87	0.20	-0.47	0.19	-1.06	-0.23	0.06	-0.64	0.28	-0.41	0.05	0.23	0.03	0.05	0.40	2.91	1.54	Stable	7.00
MMUT	-1.16	-0.19	-1.28	-0.55	-1.28	-1.00	-1.32	-1.10	-0.53	-0.63	-0.91	-1.25	-1.29	-0.50	-0.19	-1.29	0.26	0.77	-0.38	Stable	7.00
RHAG	-0.36	0.10	-0.24	-0.12	-0.54	-0.54	-0.31	-0.41	-0.06	-0.23	-0.54	-0.40	-0.49	-0.16	0.10	-0.49	0.02	0.40	-1.31	Down	7.00
FAH	-0.07	0.06	-0.12	0.06	0.02	0.02	-0.09	0.21	0.01	0.02	0.02	0.00	0.05	-0.17	0.08	0.05	0.39	-0.33	-1.59	Stable	7.00
CFAP161	0.24	0.07	0.23	0.05	0.15	0.12	0.23	0.07	0.09	-0.07	0.12	0.11	0.12	0.27	0.27	0.08	0.03	2.14	1.10	Up	7.00
STARD5	-0.05	-0.08	0.22	-0.10	-0.10	-0.16	0.18	0.06	0.12	-0.06	-0.15	-0.07	-0.15	0.09	-0.17	-0.16	0.01	-0.40	-1.34	Down	7.00
FSD2	0.20	-0.26	0.46	-0.24	0.13	-0.50	0.05	-0.20	-0.34	-0.39	-0.30	-0.17	-0.27	0.19	0.14	-0.34	0.03	-0.12	-3.11	Down	7.00
BLM	0.00	0.40	0.07	0.26	0.19	0.52	0.17	0.18	0.48	0.16	0.32	-0.01	0.15	0.00	0.33	0.18	0.84	0.92	-0.11	Stable	7.00
HDDC3	0.00	0.29	-0.25	0.41	0.12	0.48	-0.17	-0.30	-0.23	0.20	0.88	0.19	0.59	0.00	0.25	0.24	0.00	-0.13	-2.92	Down	7.00
WDR93	0.24	0.05	-0.27	-0.07	0.00	-0.55	-0.25	0.36	-0.20	-0.39	-0.74	0.10	0.06	0.18	0.40	-0.13	0.08	-0.31	-1.70	Stable	7.00
MESP1	0.00	-0.11	0.00	-0.13	-0.14	-0.27	-0.10	0.00	-0.10	-0.15	-0.25	-0.03	-0.19	0.00	-0.10	-0.13	0.00	0.31	-1.68	Down	7.00
ETFA	0.00	-0.07	-0.18	-0.07	0.04	-0.03	-0.10	-0.01	-0.09	0.00	-0.03	-0.08	0.06	0.02	-0.06	-0.06	0.18	3.04	1.60	Stable	7.00

RCN2	-0.09	-0.35	-0.19	-0.29	-0.12	-0.26	-0.20	0.01	-0.16	-0.17	-0.26	-0.19	-0.15	-0.08	-0.34	-0.25	0.49	0.83	-0.27	Stable	7.00
LINGO1	0.21	-0.53	0.04	-0.26	0.49	-0.84	-0.29	0.04	0.06	-0.90	-0.54	0.12	0.09	0.25	0.42	-0.28	0.18	-0.09	-3.47	Stable	7.00
NEIL1	-0.08	0.16	-0.12	0.30	0.27	0.54	0.04	-0.05	0.01	0.18	0.39	0.10	0.16	-0.07	0.12	0.37	0.00	0.00	-7.83	Down	7.00
ULK3	-0.13	-0.37	-0.20	-0.43	-0.27	0.28	-0.39	-0.09	-0.30	-0.17	0.26	-0.49	-0.34	-0.20	-0.31	-0.60	0.82	1.13	0.17	Stable	7.00
CYP1A1	0.00	-0.08	0.00	0.00	0.00	-0.17	0.00	0.00	0.00	0.00	-0.17	0.00	0.00	0.00	0.00	0.00	0.30	0.23	-2.13	Stable	7.00
STOML1	-0.16	-0.03	-0.32	-0.04	0.00	-0.25	-0.16	-0.08	-0.10	-0.18	-0.23	0.04	0.00	-0.16	-0.04	-0.13	0.53	1.33	0.41	Stable	7.00
INSYN1	0.00	-0.03	0.00	-0.33	-0.47	-0.06	-0.20	0.00	-0.20	-0.14	-0.06	-0.10	-0.44	0.00	-0.03	-0.17	0.04	0.27	-1.91	Down	7.00
ENSSSCG00000001927	0.00	0.06	0.05	0.17	-0.01	-0.05	0.00	0.00	-0.02	-0.01	-0.14	0.08	0.08	-0.02	-0.09	-0.17	0.97	0.66	-0.59	Stable	7.00
ENSSSCG00000001930	0.74	1.19	0.26	-0.15	1.61	1.10	0.50	0.36	0.84	0.81	0.67	-0.04	0.69	0.82	1.21	1.64	0.86	0.94	-0.09	Stable	7.00
ENSSSCG00000001931	-0.40	0.14	-0.26	0.14	0.14	0.14	-0.22	0.14	-0.04	-0.34	0.14	0.14	0.14	-0.36	0.14	0.14	0.09	-1.30	0.38	Stable	7.00
BAZ1A	-0.16	-0.31	-0.37	-0.27	-0.16	-0.21	-0.21	-0.09	-0.10	-0.18	-0.11	-0.12	-0.25	-0.14	-0.18	-0.16	0.76	1.07	0.10	Stable	7.00
AP4S1	-0.03	0.14	-0.03	-0.11	-0.30	-0.01	0.16	0.01	-0.09	0.05	-0.38	-0.14	-0.30	-0.03	-0.02	-0.04	0.02	-0.09	-3.54	Down	7.00
PRKD1	0.00	0.12	0.26	0.72	0.76	0.25	-0.07	-0.07	0.35	0.37	0.62	0.61	0.77	0.00	0.37	0.69	0.00	0.20	-2.30	Down	7.00
ENSSSCG00000001982	0.02	1.36	0.24	1.42	1.66	1.40	0.09	-0.05	-0.28	0.62	1.83	0.04	1.63	-0.09	0.92	1.58	0.00	0.22	-2.20	Down	7.00
SDR39U1	0.21	-0.19	0.11	0.00	-0.22	0.08	0.11	0.11	0.03	0.00	0.00	0.00	-0.22	0.21	-0.10	-0.17	0.08	-0.89	-0.17	Stable	7.00
KHNYN	0.00	-0.12	-0.04	-0.13	-0.13	-0.10	0.00	0.00	-0.01	-0.06	-0.08	-0.04	-0.04	0.00	-0.06	-0.12	0.01	0.34	-1.58	Down	7.00
RIPK3	-0.08	0.09	-0.14	-0.20	0.16	-0.25	-0.08	0.00	0.13	-0.18	-0.19	-0.08	0.07	-0.03	0.14	-0.03	0.17	-0.05	-4.39	Stable	7.00
IRF9	-0.07	-0.13	-0.11	-0.11	-0.16	0.00	-0.07	0.00	0.00	-0.21	-0.11	0.01	-0.16	-0.15	-0.06	-0.13	0.32	0.68	-0.56	Stable	7.00
DHRS4	0.08	0.65	0.57	0.22	0.88	0.29	0.66	0.36	-0.03	0.26	0.84	-0.33	1.17	-0.03	0.94	0.88	0.58	0.77	-0.39	Stable	7.00
MRPL52	-0.05	0.60	0.05	0.31	0.20	0.34	0.03	0.08	0.00	0.10	0.18	0.15	0.20	-0.04	0.32	0.25	0.29	0.57	-0.81	Stable	7.00
SLC7A7	0.01	-0.50	0.27	-0.55	-0.04	-0.15	-0.10	-0.09	0.41	-0.26	0.20	-0.34	-0.01	0.12	0.05	-0.42	0.12	-0.11	-3.14	Stable	7.00
ENSSSCG00000002045	0.00	0.22	0.02	0.22	0.22	0.20	0.19	0.28	0.00	0.11	0.22	0.11	0.22	0.00	0.20	0.22	0.13	0.60	-0.74	Stable	7.00
ABHD4	0.00	0.18	0.01	0.33	-0.05	0.33	0.43	0.44	0.00	0.16	0.33	0.16	-0.05	0.00	0.02	0.18	0.68	0.78	-0.36	Stable	7.00
RPGRIP1	-0.31	0.23	0.13	0.17	0.68	0.38	0.16	0.18	-0.31	-0.09	0.17	-0.01	0.42	-0.31	-0.04	0.25	0.04	-0.14	-2.85	Down	7.00
KLHL33	0.68	-0.07	0.19	-0.43	-0.55	-0.46	0.06	0.08	0.68	0.24	-0.43	0.27	-0.55	0.68	0.42	-0.55	0.00	-1.10	0.14	Stable	7.00
LPCAT4	0.26	-0.17	0.04	0.04	0.46	-0.07	-0.29	-0.23	0.26	0.16	0.12	0.18	0.24	0.26	-0.13	0.20	0.12	-0.01	-6.47	Stable	7.00
AKAP5	-0.19	-0.26	-0.24	-0.29	-0.31	-0.07	-0.46	0.16	0.46	0.00	-0.31	-0.48	-0.29	0.01	-0.02	-0.08	0.19	0.29	-1.76	Stable	7.00
PPP1R36	-0.09	-0.09	-0.09	-0.09	-0.11	-0.09	-0.11	-0.09	0.33	-0.11	-0.09	-0.11	-0.09	-0.11	0.11	-0.10	0.19	0.18	-2.47	Stable	7.00
PLEKHG3	-0.55	-0.21	-0.51	-0.23	-0.63	-0.23	-0.59	-0.04	-0.03	-0.50	-0.31	-0.44	-0.28	-0.59	-0.02	-0.44	0.55	0.83	-0.27	Stable	7.00
MPP5	0.10	0.23	0.18	-0.01	0.17	0.12	0.31	-0.21	-0.21	0.31	0.31	0.17	-0.01	0.15	0.11	0.17	0.39	0.54	-0.89	Stable	7.00
RDH12	0.00	0.06	0.16	0.52	0.31	0.52	-0.06	0.03	0.05	0.00	-0.06	0.19	0.52	0.00	-0.06	0.37	0.01	0.08	-3.71	Down	7.00
GALNT16	0.66	0.35	0.47	-0.07	1.02	-0.08	0.97	-0.19	-0.08	0.95	0.35	0.47	1.02	0.47	0.45	-0.07	0.78	0.86	-0.21	Stable	7.00
ERH	0.13	0.03	0.13	0.13	0.13	0.13	0.07	0.10	0.10	0.03	0.07	0.13	0.13	0.13	0.00	0.13	0.29	0.78	-0.36	Stable	7.00
PLEKHD1	0.64	0.45	0.64	0.64	0.64	0.35	0.44	0.15	0.10	0.48	0.46	0.64	0.64	0.64	0.10	0.64	0.11	0.70	-0.51	Stable	7.00
PAPLN	0.25	-0.35	0.65	-0.17	-0.19	0.04	-0.09	0.58	0.12	-0.10	0.36	0.49	0.47	0.32	-0.33	-0.08	0.81	1.40	0.48	Stable	7.00
ACOT4	-0.45	-0.16	-0.14	-0.07	0.48	0.48	-0.04	-0.39	-0.25	0.32	0.29	-0.08	0.11	-0.13	-0.06	-0.11	0.00	-1.13	0.17	Stable	7.00
COQ6	0.26	-0.03	-0.26	-0.23	0.23	0.16	-0.04	0.08	-0.06	-0.08	0.33	-0.23	-0.04	-0.14	-0.13	-0.10	0.63	-7.58	2.92	Stable	7.00
GSTZ1	0.21	0.51	0.48	0.07	0.69	0.18	0.32	0.06	0.19	0.70	0.45	-0.01	0.44	0.17	0.30	0.30	0.51	0.79	-0.34	Stable	7.00
VIPAS39	0.01	0.32	0.03	0.79	0.36	0.24	-0.01	0.24	0.63	0.17	0.17	0.18	0.06	0.34	0.32	-0.05	0.96	0.97	-0.04	Stable	7.00
ZC3H14	-0.07	-0.12	-0.17	-0.23	-0.40	-0.40	-0.12	-0.29	-0.17	-0.04	-0.16	-0.29	-0.16	-0.42	0.08	-0.31	0.21	0.64	-0.64	Stable	7.00
EML5	-0.38	-0.42	-0.29	-0.60	-0.60	-0.60	-0.42	-0.33	-0.31	-0.24	-0.48	-0.54	-0.48	-0.72	-0.14	-0.72	0.06	0.70	-0.50	Stable	7.00
KCNK13	0.55	0.00	0.10	0.32	0.32	0.37	0.23	0.04	0.34	0.41	0.12	0.37	0.09	0.21	0.27	0.55	0.24	0.68	-0.56	Stable	7.00
PSMC1	-0.51	-0.21	-0.07	-0.53	-0.29	-0.45	-0.20	-0.21	-0.24	-0.50	-0.33	-0.38	-0.33	-0.32	-0.10	-0.53	0.01	0.56	-0.85	Stable	7.00
NRDE2	0.30	0.18	0.46	0.12	0.75	0.62	0.64	0.47	1.07	0.18	-0.08	0.39	-0.08	0.69	0.49	0.18	0.07	2.08	1.05	Stable	7.00
TRIP11	0.28	0.27	0.66	0.23	0.47	0.79	0.68	0.73	-0.01	0.56	0.48	0.14	0.26	0.56	0.76	0.40	0.56	1.18	0.24	Stable	7.00

LGMN	0.27	-0.16	0.10	0.41	0.51	0.15	-0.19	-0.35	0.05	-0.25	0.25	0.25	0.61	-0.23	-0.23	0.53	0.01	-0.30	-1.74	Down	7.00
CHGA	-0.27	-0.86	-1.18	-0.28	-0.20	-0.40	-0.74	-0.33	-1.19	-0.33	-1.40	-0.74	-0.68	-1.52	-0.18	0.06	0.24	1.58	0.66	Stable	7.00
PPP4R4	0.08	0.09	0.05	0.05	-0.04	-0.16	0.09	-0.20	0.09	-0.07	0.00	0.05	-0.04	0.09	-0.06	-0.12	0.13	-0.73	-0.46	Stable	7.00
AK7	-0.97	-0.42	-0.33	-0.07	-0.88	-0.97	-0.74	-0.72	-0.72	-0.88	-0.79	-0.46	-1.07	-0.83	-0.90	-0.97	0.69	0.92	-0.11	Stable	7.00
WDR25	0.11	-0.16	0.00	-0.23	-0.02	0.28	-0.08	0.00	0.00	-0.23	0.20	-0.11	0.00	0.00	0.00	0.09	0.88	3.61	1.85	Stable	7.00
GSTA4	-0.30	0.32	0.22	-0.10	-0.70	-0.41	0.13	0.70	-0.05	0.21	-0.23	-0.77	-0.51	0.14	0.11	-0.51	0.00	-0.42	-1.26	Down	7.00
FBXO9	0.27	0.42	0.80	0.20	0.02	0.19	0.28	0.35	0.47	0.24	0.20	0.06	0.04	0.33	0.24	0.04	0.00	3.21	1.68	Up	7.00
GAS8	0.09	0.05	0.02	0.13	-0.33	0.09	0.09	0.09	0.09	0.09	-0.31	0.11	0.09	-0.07	0.04	0.09	0.45	-6.66	2.74	Stable	6.00
GALNS	0.09	0.02	0.08	-0.04	0.09	0.09	0.09	0.06	0.02	0.02	0.03	0.03	0.09	0.05	0.03	0.09	0.87	1.06	0.09	Stable	6.00
APRT	-0.23	0.12	0.07	0.79	-0.23	-0.31	-0.23	-0.23	-0.23	-0.23	0.00	0.24	-0.31	-0.24	0.06	-0.31	0.65	2.53	1.34	Stable	6.00
KLHDC4	-0.97	-0.47	-0.58	0.23	-1.09	-0.82	-0.97	-0.55	-0.59	-0.58	-0.38	-0.27	-0.82	-0.33	-0.40	-0.82	0.81	1.07	0.10	Stable	6.00
ENSSSCG00000002653	-0.73	-0.64	-0.64	-0.28	-0.31	-0.73	-0.78	-0.11	-0.40	-0.37	0.03	-0.50	-0.73	-0.52	-0.32	-0.73	0.62	1.14	0.19	Stable	6.00
MTHFSD	0.75	0.29	0.51	-0.10	0.39	-0.03	0.98	0.19	0.45	0.88	1.00	0.00	0.34	0.44	-0.37	0.34	0.80	1.15	0.20	Stable	6.00
GSE1	0.25	0.24	0.24	0.12	0.20	0.10	0.25	0.32	0.24	0.25	0.20	0.14	0.24	0.20	0.25	0.24	0.03	1.33	0.41	Stable	6.00
ZDHHC7	0.09	0.05	0.05	0.03	0.10	0.04	0.04	0.06	0.05	0.04	0.18	0.04	0.05	0.10	0.03	0.05	0.71	0.88	-0.18	Stable	6.00
CRISPLD2	0.61	0.52	0.52	0.97	0.51	0.83	0.51	0.61	0.52	0.57	0.66	0.66	0.52	0.46	0.52	0.52	0.08	0.82	-0.29	Stable	6.00
TAF1C	-0.09	-0.04	-0.04	-0.44	-0.21	-0.24	-0.09	0.00	-0.04	-0.07	-0.30	-0.24	-0.04	-0.03	0.09	-0.04	0.01	0.15	-2.74	Down	6.00
GAN	0.27	0.00	0.00	0.60	0.60	0.60	0.30	0.43	0.27	0.60	0.60	0.27	0.60	0.30	0.43	0.60	0.00	0.45	-1.16	Down	6.00
NUDT7	0.51	0.51	0.51	0.51	0.51	0.51	0.23	0.19	0.72	0.86	0.52	0.53	0.51	0.22	0.06	0.51	0.06	0.66	-0.59	Stable	6.00
ENSSSCG00000002700	-0.65	-0.65	-0.65	-0.65	-0.65	-0.65	-0.82	-1.15	-0.92	-0.86	-0.19	-0.06	-0.65	-0.32	0.09	-0.65	0.60	1.17	0.22	Stable	6.00
LRRC36	0.11	0.06	0.00	0.04	0.00	0.00	0.06	0.00	0.00	0.11	0.00	0.04	0.00	0.06	0.04	0.00	0.43	1.66	0.73	Stable	6.00
KCTD19	0.52	0.45	0.38	0.09	0.34	0.38	0.35	0.13	0.09	0.52	0.13	0.35	0.13	0.74	0.54	0.24	0.20	1.46	0.55	Stable	6.00
ELMO3	0.00	0.00	0.00	-0.08	-0.07	-0.07	0.00	-0.07	0.00	0.00	-0.08	0.00	-0.07	0.00	0.00	-0.07	0.01	0.14	-2.82	Down	6.00
GINS3	-0.01	0.88	0.69	0.73	0.55	0.62	1.40	1.23	0.80	0.39	0.76	0.10	-0.01	0.40	0.80	0.48	0.11	1.71	0.78	Stable	6.00
PLLP	0.30	0.05	0.11	0.13	0.13	0.13	0.76	0.34	0.74	0.06	0.07	0.29	0.48	0.11	0.27	0.13	0.19	1.86	0.90	Stable	6.00
ARL2BP	0.02	-0.04	0.04	-0.10	-0.10	-0.10	0.32	0.07	0.32	-0.21	-0.21	-0.05	0.11	-0.05	-0.03	-0.10	0.02	-0.86	-0.21	Stable	6.00
ENSSSCG00000002825	0.99	0.69	0.26	0.52	0.73	0.73	0.59	0.30	-0.07	0.99	0.99	0.75	0.62	0.59	0.60	0.66	0.07	0.66	-0.61	Stable	6.00
SLC6A2	-0.24	-0.12	0.00	0.00	-0.23	-0.24	-0.08	-0.06	-0.13	-0.24	0.00	-0.12	0.00	-0.08	-0.12	-0.10	0.79	0.89	-0.17	Stable	6.00
POP4	0.00	-0.08	-0.09	0.00	0.00	0.00	0.00	0.00	0.00	0.00	0.00	0.00	0.00	0.00	0.00	0.00	0.17	#NAME?	Inf	Stable	6.00
TDRD12	-0.25	-0.11	-0.62	-0.01	0.68	0.66	-0.93	0.15	0.17	0.62	0.10	-0.96	-0.08	-0.25	-0.96	-0.88	0.22	-21.91	4.45	Stable	6.00
ANKRD27	-0.06	-0.12	-0.22	-0.22	0.12	-0.01	-0.35	-0.27	-0.27	-0.17	0.00	-0.35	-0.17	0.03	-0.35	-0.42	0.54	1.33	0.41	Stable	6.00
ENSSSCG00000002875	0.27	0.26	0.22	0.02	0.18	0.04	0.13	0.33	0.20	0.36	-0.01	0.13	0.17	0.25	0.13	0.05	0.05	1.95	0.96	Stable	6.00
ENSSSCG00000002877	0.11	-0.01	-0.11	-0.36	0.21	-0.30	-0.66	-0.27	-0.20	0.13	-0.32	-0.66	0.06	0.32	-0.66	-0.25	0.99	0.99	-0.01	Stable	6.00
USF2	1.18	0.22	0.59	0.31	0.61	0.55	1.41	0.70	0.49	0.64	0.44	1.41	0.65	0.84	1.41	0.71	0.35	1.28	0.36	Stable	6.00
HAUS5	-0.09	-0.07	-0.20	-0.06	-0.30	0.01	-0.15	-0.14	-0.18	-0.19	-0.09	-0.15	-0.12	-0.15	-0.15	-0.07	0.59	1.17	0.22	Stable	6.00
APLP1	-0.09	0.00	0.15	-0.31	-0.13	-0.27	-0.04	0.10	0.08	0.10	-0.38	-0.04	-0.02	0.10	-0.04	-0.02	0.03	-0.25	-1.97	Down	6.00
ZNF260	-0.03	0.49	0.69	-0.12	-0.12	-0.13	0.28	-0.31	0.17	-0.08	-0.15	0.18	0.30	-0.09	0.18	0.38	0.33	5.17	2.37	Stable	6.00
ZNF565	-0.12	0.19	-0.05	-0.19	-0.24	-0.22	-0.28	0.19	0.19	0.04	-0.20	-0.28	-0.11	0.04	-0.28	-0.20	0.07	0.09	-3.47	Stable	6.00
ZNF567	0.29	0.10	0.17	0.05	0.24	0.27	0.15	0.76	0.42	0.48	0.17	0.15	0.63	0.34	0.15	0.54	0.87	0.94	-0.09	Stable	6.00
ZNF382	-0.02	0.36	0.43	-0.12	-0.08	-0.12	-0.05	-0.14	-0.05	-0.06	-0.16	-0.05	0.36	0.30	-0.05	0.12	0.28	-6.47	2.69	Stable	6.00
ENSSSCG00000002937	0.46	0.24	1.10	-0.13	0.50	-0.06	1.01	0.09	0.39	0.40	-0.24	0.97	0.57	0.55	1.01	0.78	0.24	1.74	0.80	Stable	6.00
SPINT2	0.00	0.31	0.11	-0.05	-0.05	-0.05	0.00	0.23	0.31	0.11	-0.05	0.00	-0.05	0.06	0.00	-0.05	0.02	-5.77	2.53	Up	6.00
FAM98C	0.26	-0.10	-0.06	0.55	0.14	0.55	0.20	-0.47	-0.16	-0.05	0.23	0.20	0.55	0.04	0.20	0.42	0.01	-0.04	-4.80	Down	6.00
ENSSSCG00000002963	-0.39	-0.12	-0.24	-0.38	0.03	-0.39	-0.38	-0.10	-0.12	-0.27	-0.22	-0.38	-0.03	-0.35	-0.38	-0.02	0.52	1.24	0.32	Stable	6.00
ACTN4	0.12	-0.01	0.12	0.00	0.02	0.02	0.25	-0.01	-0.01	-0.01	0.00	0.25	0.02	-0.01	0.25	0.18	0.63	1.43	0.51	Stable	6.00

ENSSSCG00000002980	-0.17	0.09	-0.20	-1.00	-0.72	-0.27	-0.52	-0.39	0.14	-0.36	-0.32	-0.32	-0.80	-0.27	-0.28	-0.23	0.04	0.40	-1.33	Down	14.00
LGALS13	-0.60	0.18	-0.32	-0.61	-0.73	-0.72	-0.47	0.63	0.27	-0.13	-0.60	-0.47	-0.82	-0.13	-0.47	-0.68	0.02	0.19	-2.40	Down	6.00
DLL3	1.04	1.04	0.40	0.82	0.72	0.67	1.28	0.72	1.12	0.73	0.86	1.28	0.63	0.30	1.28	0.94	0.67	1.08	0.11	Stable	6.00
ENSSSCG00000002997	0.54	0.97	0.65	0.71	0.13	0.62	0.54	0.93	0.91	0.53	0.81	0.54	0.34	0.83	0.54	0.09	0.04	1.56	0.64	Stable	6.00
CYP2B22	0.67	0.97	0.85	0.20	0.83	0.27	0.52	1.32	0.92	1.08	-0.21	0.52	0.87	0.91	0.52	0.68	0.11	1.57	0.65	Stable	6.00
B9D2	-0.27	-0.12	-0.28	-0.24	-0.07	-0.24	-0.28	-0.30	-0.12	-0.27	-0.27	-0.28	-0.06	-0.27	-0.28	0.09	0.21	1.44	0.52	Stable	6.00
EXOSC5	-0.03	0.00	-0.02	-0.02	-0.03	-0.02	-0.03	0.00	0.00	0.00	-0.02	-0.03	-0.03	-0.02	-0.03	-0.03	0.35	0.71	-0.49	Stable	6.00
LIPE	-0.06	-0.03	-0.08	-0.18	0.17	-0.21	-0.05	0.35	0.17	0.16	-0.19	-0.05	0.18	-0.10	-0.05	0.08	0.81	-4.13	2.05	Stable	6.00
MEGF8	-0.31	0.30	-0.20	0.04	-0.49	-0.01	-0.55	-0.50	0.02	-0.36	-0.04	-0.55	-0.32	0.04	-0.55	-0.49	0.69	0.80	-0.33	Stable	6.00
LYPD4	-0.29	-0.62	-0.65	-0.26	0.27	-0.21	-1.18	-0.49	-0.48	-0.29	-0.12	-1.18	0.48	-0.45	-1.18	-0.25	0.04	3.42	1.77	Up	6.00
ENSSSCG00000003047	-0.17	0.02	-0.17	0.02	-0.07	0.02	-0.32	-0.19	-0.09	-0.06	-0.10	-0.32	-0.02	0.02	-0.32	-0.06	0.21	2.05	1.03	Stable	6.00
XRCC1	-0.33	-0.42	-0.25	-0.13	-0.12	-0.42	-0.16	-0.29	-0.45	-0.35	-0.31	-0.16	-0.03	-0.31	-0.16	0.14	0.13	1.72	0.78	Stable	6.00
CEACAM16	-0.08	0.03	-0.01	-0.21	-0.21	-0.21	-0.27	0.00	0.02	-0.14	-0.27	-0.27	-0.07	-0.21	-0.27	0.04	0.28	0.59	-0.75	Stable	6.00
CLPTM1	0.40	-0.05	0.11	0.18	0.39	0.05	0.51	0.18	0.09	0.31	0.14	0.51	0.45	0.07	0.51	0.40	0.45	0.76	-0.40	Stable	6.00
ENSSSCG00000003090	-0.06	0.04	0.04	0.14	0.22	-0.16	0.09	0.09	0.09	0.01	0.15	0.09	-0.11	-0.11	0.09	0.05	0.77	0.66	-0.60	Stable	6.00
IRF2BP1	-0.12	0.02	0.10	0.03	0.45	0.01	0.25	0.00	0.00	0.02	0.24	0.25	-0.19	-0.21	0.25	0.17	0.36	0.31	-1.70	Stable	6.00
TMEM160	0.36	0.09	0.00	0.48	0.37	0.44	-0.24	0.09	0.09	0.52	-0.08	-0.24	0.38	0.48	-0.24	0.11	0.22	0.31	-1.68	Stable	6.00
ZNF541	-0.08	-0.57	-0.77	-0.09	-0.49	-0.15	-0.80	-0.85	-0.63	-0.13	-0.37	-0.80	-0.05	-0.19	-0.80	-0.69	0.12	1.69	0.75	Stable	6.00
FGF21	0.16	-0.17	0.19	0.02	0.13	0.03	0.33	-0.17	-0.12	0.02	0.10	0.33	0.02	-0.01	0.33	0.03	0.83	0.78	-0.35	Stable	6.00
DHDH	0.43	0.85	0.27	0.38	0.80	0.15	0.55	0.59	0.84	0.25	0.53	0.55	0.28	0.27	0.55	0.84	0.55	1.16	0.21	Stable	6.00
HRC	0.15	0.19	0.15	0.19	0.19	0.15	0.08	0.08	0.22	0.22	0.12	0.08	0.19	0.18	0.08	0.06	0.77	0.94	-0.08	Stable	6.00
PIH1D1	0.01	0.34	0.18	0.20	0.07	0.02	0.02	0.16	0.36	0.18	0.20	0.02	0.05	0.01	0.02	0.07	0.56	1.35	0.43	Stable	6.00
IRF3	0.00	-0.27	0.01	-0.25	-0.44	-0.17	0.26	-0.13	-0.28	-0.19	0.02	0.26	-0.55	-0.19	0.26	-0.43	0.17	0.19	-2.38	Stable	6.00
FCGRT	0.15	-0.17	-0.09	-0.06	0.19	-0.13	0.25	0.21	-0.07	-0.01	-0.01	0.25	0.12	-0.16	0.25	0.22	0.76	0.64	-0.63	Stable	6.00
PRMT1	0.22	-0.05	0.00	0.38	-0.06	0.29	0.00	0.00	-0.05	0.26	0.00	0.00	0.20	0.25	0.00	-0.06	0.31	0.37	-1.45	Stable	6.00
TSKS	-0.01	0.18	0.03	0.11	-0.01	0.15	-0.12	0.03	0.18	0.15	0.00	-0.12	0.11	0.14	-0.12	-0.01	0.88	0.83	-0.27	Stable	6.00
TBC1D17	0.16	-0.08	-0.05	-0.08	-0.73	0.09	-0.03	0.12	0.03	0.19	-0.18	-0.03	-0.41	0.04	-0.03	-0.48	0.09	-0.10	-3.34	Stable	6.00
IZUMO2	0.52	-0.29	-0.02	0.06	-0.48	0.50	-0.20	-0.94	-0.31	-0.42	-0.53	-0.20	0.15	0.01	-0.20	0.00	0.75	1.55	0.63	Stable	6.00
NAPSA	0.09	-0.69	-0.34	0.76	0.10	0.49	-0.26	-0.37	-0.78	-0.12	-0.13	-0.26	0.44	0.11	-0.26	-0.20	0.02	-2.27	1.18	Up	6.00
MYBPC2	0.25	0.01	0.09	-0.03	0.20	-0.08	0.19	0.15	0.02	0.12	-0.08	0.19	0.31	0.08	0.19	0.22	0.80	1.15	0.20	Stable	6.00
SYT3	-0.10	0.30	0.06	0.20	-0.14	0.12	-0.17	0.07	0.30	0.10	0.10	-0.17	-0.26	0.11	-0.17	-0.26	0.37	-1.21	0.28	Stable	6.00
VSIG10L	-0.14	-0.17	-0.29	-0.02	-0.20	-0.02	-0.40	0.12	-0.17	-0.23	-0.21	-0.40	0.03	-0.05	-0.40	-0.21	0.70	1.20	0.26	Stable	6.00
ENSSSCG00000003235	-0.42	-0.42	-0.42	-0.42	-0.42	-0.42	-0.41	-0.21	-0.42	-0.42	-0.42	-0.41	-0.42	-0.42	-0.41	-0.41	0.35	0.94	-0.09	Stable	6.00
ENSSSCG00000003240	0.30	-0.22	0.03	0.00	0.12	-0.09	0.20	0.15	-0.22	-0.01	-0.05	0.20	0.16	0.15	0.20	0.09	0.81	1.36	0.45	Stable	6.00
NLRP12	0.05	-0.35	-0.10	-0.12	-0.33	-0.13	0.15	-0.35	-0.35	-0.28	-0.14	0.15	-0.33	-0.30	0.15	-0.07	0.86	0.88	-0.18	Stable	6.00
PRKCG	0.17	0.23	0.19	0.10	0.28	0.11	0.10	0.23	0.23	0.23	0.09	0.10	0.26	0.23	0.10	0.24	0.87	1.03	0.05	Stable	6.00
ENSSSCG00000003278	0.21	0.31	0.80	0.01	0.30	-0.08	1.44	0.39	0.27	0.09	0.34	1.44	0.29	0.07	1.44	1.10	0.52	1.41	0.50	Stable	6.00
ENSSSCG00000003302	0.00	-0.01	-0.01	0.00	0.00	0.00	0.00	-0.18	0.00	0.00	-0.14	0.00	0.00	-0.07	0.00	0.00	0.59	1.88	0.91	Stable	6.00
GALP	0.40	0.18	0.39	0.10	0.62	0.09	0.61	0.58	0.18	0.18	0.09	0.61	0.58	0.18	0.61	0.83	0.97	1.01	0.02	Stable	6.00
ZNF471	-0.50	-0.57	-0.36	-0.42	-0.46	-0.48	-0.16	-0.42	-0.57	-0.67	-0.08	-0.16	-0.63	-0.71	-0.16	-0.24	0.71	1.10	0.14	Stable	6.00
PUSL1	-0.37	-1.73	-0.64	-0.30	-1.23	-0.29	-0.22	-1.71	-1.77	-1.13	-0.52	-0.22	-1.08	-1.08	-0.22	-1.15	0.45	1.31	0.39	Stable	6.00
ESPN	-0.05	0.09	0.03	-0.08	-0.05	0.08	-0.02	0.11	-0.01	-0.04	0.00	-0.02	-0.09	0.08	-0.02	-0.06	0.05	-0.83	-0.28	Stable	6.00
RBP7	0.04	0.01	0.07	0.17	0.29	0.16	0.05	0.05	0.00	0.02	0.17	0.05	0.17	0.06	0.04	0.17	0.01	0.27	-1.88	Down	6.00
NMNAT1	-0.17	0.00	-0.12	-0.49	-1.18	-0.39	-0.34	0.08	-0.22	-0.11	-0.47	-0.34	-0.63	0.24	-0.06	-0.87	0.00	0.13	-2.93	Down	6.00
CENPS	0.02	0.02	0.05	0.44	0.37	0.37	-0.10	0.00	0.00	0.03	0.14	-0.10	0.06	0.06	-0.01	-0.02	0.07	0.03	-5.01	Stable	6.00

DFFA	0.42	0.10	0.42	0.88	1.03	0.85	0.52	0.27	0.06	0.34	0.87	0.52	0.93	0.12	0.43	0.73	0.00	0.38	-1.40	Down	6.00
MAD2L2	0.76	0.03	0.10	0.67	0.65	0.62	0.65	0.28	0.13	0.54	0.67	0.65	0.86	-0.01	0.76	0.66	0.03	0.51	-0.98	Stable	6.00
FBXO6	0.27	-0.42	-0.56	-0.47	-0.21	-0.12	0.11	-0.16	-0.06	-0.19	-0.22	-0.31	-0.35	-0.18	0.26	0.11	0.32	0.42	-1.25	Stable	6.00
MIIP	0.37	0.35	0.96	0.69	0.24	0.68	1.40	-0.07	0.03	0.28	0.62	1.40	0.24	0.65	1.24	0.65	0.94	1.03	0.04	Stable	6.00
PDPN	-0.06	-0.09	0.26	0.53	0.14	0.58	0.32	0.17	0.09	0.45	0.52	0.32	0.21	0.01	-0.15	0.24	0.00	0.18	-2.44	Down	6.00
CTRC	0.12	0.00	0.00	0.13	0.00	0.03	0.00	0.21	0.11	0.22	0.11	0.00	0.13	0.04	0.00	0.00	0.68	0.78	-0.35	Stable	6.00
FBLIM1	-0.33	-0.28	-0.04	-0.15	0.02	-0.16	-0.30	-0.04	-0.32	-0.19	-0.16	-0.08	-0.10	-0.15	0.10	-0.04	0.34	1.58	0.66	Stable	6.00
TMEM82	-0.14	-0.04	0.00	-0.20	-0.10	-0.20	-0.04	0.00	-0.04	-0.10	-0.20	0.00	-0.10	-0.10	0.00	0.00	0.07	0.40	-1.34	Stable	6.00
CROCC	0.01	-0.18	0.08	0.23	0.03	0.36	0.30	-0.02	0.02	0.14	0.32	0.49	0.04	-0.13	0.04	0.13	0.02	0.07	-3.87	Down	6.00
ARHGEF10L	1.16	1.87	1.03	0.78	1.19	0.83	1.09	-0.06	0.86	0.41	0.95	0.37	0.96	1.53	0.71	0.59	0.27	1.35	0.43	Stable	6.00
SLC66A1	-0.13	-0.01	0.00	-0.17	-0.02	-0.26	-0.10	0.09	-0.09	-0.30	-0.26	-0.10	-0.21	-0.12	0.09	-0.04	0.02	0.20	-2.33	Down	6.00
GRHL3	-0.87	-0.85	-0.83	-0.21	-0.46	-0.21	-0.80	-0.90	-0.84	-0.50	-0.21	-0.80	-0.07	-0.94	-0.44	-0.34	0.00	2.30	1.20	Up	6.00
STPG1	-0.61	-1.23	-1.13	-0.63	-0.83	-0.63	-1.20	-0.90	-1.29	-0.91	-0.65	-1.20	-0.62	-0.61	-0.93	-0.77	0.11	1.26	0.34	Stable	6.00
NIPAL3	0.32	0.36	0.56	-0.24	-0.20	-0.24	0.10	0.45	0.01	-0.17	-0.09	0.10	-0.32	0.65	-0.32	-0.28	0.01	-1.48	0.57	Stable	6.00
LDLRAP1	-0.28	-0.11	-0.12	-0.22	-0.17	-0.22	-0.06	0.15	0.12	-0.13	0.02	-0.06	-0.29	-0.36	-0.26	-0.15	0.60	0.75	-0.42	Stable	6.00
PAQR7	-0.23	-0.24	-0.24	-0.08	-0.12	-0.08	-0.19	-0.18	-0.17	-0.12	-0.09	-0.19	-0.10	-0.29	-0.15	-0.11	0.00	1.86	0.90	Stable	6.00
SELENON	0.06	0.27	0.24	1.28	1.52	1.28	-0.10	-0.23	0.35	1.13	0.64	-0.10	1.61	0.38	-0.26	0.79	0.00	0.09	-3.52	Down	6.00
EYA3	0.00	0.05	0.05	0.00	-0.08	0.00	0.03	0.08	-0.13	-0.14	-0.11	0.03	0.00	0.04	0.00	0.02	0.13	-0.46	-1.14	Stable	6.00
SMPDL3B	-0.02	-0.19	-0.19	0.13	0.20	0.13	-0.13	-0.09	-0.01	0.29	0.31	-0.13	0.13	-0.05	-0.10	0.00	0.00	-0.75	-0.42	Stable	6.00
EPB41	0.61	0.88	0.88	0.79	0.27	0.79	0.91	0.43	0.33	0.38	0.36	0.91	0.79	0.48	0.42	0.86	0.84	0.96	-0.06	Stable	6.00
SPOCD1	0.10	0.02	0.07	0.18	0.21	0.91	0.22	0.26	0.25	0.27	0.35	0.22	0.18	0.02	0.18	0.21	0.10	0.45	-1.16	Stable	6.00
TRIM62	-0.01	0.11	0.16	0.06	0.06	0.16	0.08	0.09	0.06	0.06	0.06	0.08	0.06	0.09	0.18	0.07	0.42	1.26	0.33	Stable	6.00
GJA9	-0.53	-0.46	-0.04	-0.37	-0.45	-0.25	-0.53	-0.53	-0.53	-0.45	-0.45	-0.53	-0.37	-0.07	-0.67	-0.45	0.96	1.01	0.01	Stable	6.00
RHBDL2	-0.28	-0.10	-0.14	0.05	-0.21	-0.44	-0.40	-0.40	-0.38	-0.16	-0.10	-0.40	0.05	0.27	-0.32	-0.17	0.66	1.27	0.35	Stable	6.00
PPIE	-0.42	0.17	-0.05	-0.14	-0.15	-0.07	-0.45	0.05	-0.29	-0.08	0.18	-0.45	-0.14	-0.07	-0.05	-0.36	0.90	0.91	-0.13	Stable	6.00
ANKRD29	-0.01	0.11	0.22	0.09	0.14	0.18	0.06	-0.03	-0.03	0.15	0.06	0.12	0.09	0.07	0.01	0.19	0.05	0.40	-1.32	Stable	6.00
CABYR	-0.01	-0.11	-0.10	0.00	0.00	-0.11	-0.21	0.00	-0.22	0.00	0.00	-0.10	0.00	-0.01	0.00	-0.10	0.29	2.07	1.05	Stable	6.00
GALNT1	0.11	0.05	0.05	-0.09	-0.09	0.00	-0.09	0.10	0.16	0.00	-0.09	0.00	-0.09	-0.05	0.18	-0.05	0.01	-1.16	0.22	Stable	6.00
MOCOS	0.35	0.23	0.18	-0.22	0.33	0.09	0.10	-0.37	0.16	-0.07	-0.22	0.09	-0.04	0.21	0.32	0.23	0.26	6.00	2.59	Stable	6.00
KIAA1328	0.00	0.00	0.00	0.00	0.00	0.00	0.00	0.14	0.00	0.00	0.00	0.00	0.00	0.00	0.07	0.00	0.20	Inf	Inf	Stable	6.00
ENSSSCG00000003753	-0.21	-0.28	-0.12	-0.28	-0.30	-0.15	-0.15	-0.30	-0.28	-0.13	-0.30	0.00	-0.30	-0.28	-0.15	0.00	0.49	1.21	0.27	Stable	6.00
CTBS	0.04	0.15	0.19	1.30	-0.59	0.12	-0.06	0.18	0.40	0.89	1.55	0.22	0.68	0.06	0.07	-0.25	0.22	0.27	-1.92	Stable	6.00
AK5	-0.06	0.00	0.14	-0.33	-0.11	0.46	0.01	-0.11	0.20	-0.19	-0.54	0.08	-0.40	-0.02	0.06	0.07	0.25	-0.24	-2.08	Stable	6.00
TYW3	0.82	0.38	0.35	0.07	0.10	0.10	0.82	0.13	0.48	0.31	0.00	0.82	0.07	0.36	0.47	0.36	0.07	2.09	1.06	Stable	6.00
CRYZ	-0.08	0.19	0.18	0.21	0.21	0.31	-0.08	0.18	-0.20	0.14	0.23	-0.08	0.21	0.29	-0.07	-0.03	0.24	0.35	-1.51	Stable	6.00
LEPROT	0.02	0.07	-0.15	-0.24	-0.36	0.03	-0.06	-0.21	0.27	-0.24	-0.22	-0.27	-0.10	-0.15	-0.20	-0.28	0.05	0.24	-2.05	Down	6.00
FGGY	-0.03	-0.18	-0.18	-0.18	-0.07	-0.28	-0.10	-0.02	0.00	-0.07	0.08	-0.02	-0.02	-0.18	0.15	-0.02	0.93	0.93	-0.10	Stable	6.00
TACSTD2	0.62	0.13	-0.03	0.22	-0.04	0.22	0.56	0.38	0.43	-0.04	-0.04	0.38	0.38	0.13	0.62	0.38	0.15	1.95	0.96	Stable	6.00
TTC4	-0.03	0.04	0.13	0.01	0.11	0.18	-0.05	0.02	0.01	0.24	-0.09	-0.05	-0.05	-0.02	-0.20	0.04	0.28	-0.23	-2.14	Stable	6.00
CZIB	0.12	-0.25	-0.05	-0.28	-0.11	-0.43	-0.39	-0.50	-0.50	0.35	-0.27	0.31	-0.24	-0.21	-0.14	0.24	0.19	4.48	2.16	Stable	6.00
CPT2	-0.07	0.14	0.01	0.10	0.24	0.22	0.10	0.10	0.10	0.18	-0.09	0.11	0.10	-0.02	0.06	0.18	0.11	0.40	-1.31	Stable	6.00
ECHDC2	0.09	-0.14	-0.52	-0.76	-0.47	-0.34	-0.26	-0.15	-0.29	-0.23	-0.27	-0.24	-0.63	0.02	-0.74	-0.99	0.10	0.50	-0.99	Stable	6.00
ORC1	-0.29	0.06	0.02	0.10	0.02	-0.03	0.10	0.10	0.10	-0.04	0.10	0.02	0.10	0.02	0.07	0.10	0.61	0.44	-1.18	Stable	6.00
TXNDC12	-0.24	-0.11	-0.23	-0.25	-0.25	-0.35	-0.25	-0.19	-0.19	-0.23	-0.19	-0.25	-0.25	-0.19	-0.30	-0.28	0.10	0.83	-0.28	Stable	6.00
TTC39A	0.15	-0.85	-0.50	-0.32	0.23	0.06	-0.29	-0.22	-0.17	-0.59	-0.05	-0.72	-0.49	0.10	-0.75	-0.27	0.79	1.18	0.24	Stable	6.00

SLC5A9	-0.16	-0.10	-0.02	-0.16	-0.30	-0.29	-0.12	-0.06	-0.08	-0.19	-0.12	-0.19	-0.12	-0.10	-0.06	-0.49	0.01	0.37	-1.42	Down	6.00
CYP4B1	0.85	-0.10	-0.21	0.20	-0.24	-0.16	0.37	-0.23	0.07	0.55	0.37	0.10	0.37	-0.09	0.44	0.24	0.81	0.77	-0.38	Stable	6.00
FAAH	0.37	1.08	1.12	0.52	1.01	0.99	1.22	0.69	0.95	0.57	0.82	1.20	1.11	0.72	0.43	0.86	0.67	0.93	-0.11	Stable	6.00
TSPAN1	-0.15	-0.12	-0.12	-0.11	-0.12	-0.12	-0.12	-0.12	-0.12	-0.13	-0.12	-0.12	-0.12	-0.14	-0.13	-0.05	0.14	1.14	0.19	Stable	6.00
MAST2	-1.52	-1.82	-1.34	-1.35	-1.62	-1.79	-1.60	-1.78	-2.06	-1.77	-1.84	-1.60	-1.48	-1.67	-1.73	-0.62	0.27	1.12	0.17	Stable	6.00
PRDX1	-0.22	-0.24	-0.18	0.15	-0.10	-0.07	-0.17	0.01	-0.02	0.05	0.12	-0.24	0.02	-0.34	-0.14	0.05	0.02	72.85	6.19	Up	6.00
TESK2	-0.79	-0.48	-0.47	0.01	-0.16	-0.19	0.07	-0.66	-0.36	-0.90	-0.36	-0.17	0.04	-0.72	-1.34	0.04	0.05	2.81	1.49	Stable	6.00
ENSSSCG00000003919	0.42	-0.24	-0.45	0.48	-0.64	-0.39	-0.38	-0.22	-0.39	0.09	-0.22	-0.33	-0.56	-0.30	-0.18	-0.43	0.84	0.86	-0.21	Stable	6.00
HECTD3	0.27	0.33	0.34	0.18	0.28	0.28	0.28	0.28	0.28	0.23	0.28	0.28	0.28	0.34	0.24	0.22	0.05	1.16	0.22	Stable	6.00
MED8	-0.41	-0.16	-0.10	-0.31	-0.21	-0.46	-0.32	-0.27	0.01	0.07	-0.27	-0.32	-0.19	-0.20	-0.26	-0.12	0.87	0.95	-0.08	Stable	6.00
TIE1	0.03	0.00	-0.02	-0.28	-0.04	-0.56	0.03	-0.59	-0.32	0.02	-0.59	0.03	-0.26	-0.01	-0.31	0.02	0.62	0.71	-0.50	Stable	6.00
PPCS	-0.11	0.01	0.01	0.03	-0.08	-0.16	0.00	0.00	-0.21	-0.51	0.00	0.02	0.01	0.01	-0.07	-0.01	0.55	0.50	-1.00	Stable	6.00
ZMYND12	0.16	0.05	0.04	-0.04	0.42	0.25	0.09	-0.08	-0.14	0.18	-0.08	0.09	-0.03	0.10	0.20	0.18	0.36	0.42	-1.25	Stable	6.00
ZFP69B	0.08	-0.08	0.26	0.19	0.23	0.23	0.39	0.42	0.27	-0.16	0.77	0.39	0.68	-0.11	0.13	0.73	0.14	0.44	-1.18	Stable	6.00
ENSSSCG00000003986	0.41	0.44	1.34	0.08	0.69	0.08	0.79	0.34	0.01	0.22	-0.11	0.79	0.47	0.56	0.79	1.13	0.43	1.40	0.48	Stable	6.00
ENSSSCG00000003987	-0.03	0.80	0.86	0.00	-0.06	0.00	-0.06	-0.26	-0.30	-0.18	-0.10	-0.06	-0.03	0.85	-0.06	-0.06	0.16	-3.66	1.87	Stable	6.00
ZNF584	0.00	0.20	0.20	0.00	-0.18	0.00	0.00	-0.20	-0.20	-0.10	0.05	0.00	-0.12	0.27	0.00	-0.18	0.19	-0.52	-0.95	Stable	6.00
WDR27	0.28	0.15	-0.04	0.20	0.25	0.32	0.31	0.24	0.34	0.49	0.03	0.23	0.32	0.24	0.28	0.22	0.62	0.88	-0.19	Stable	1.00
ERMARD	-0.23	0.09	0.33	-0.03	0.01	-0.01	-0.09	-0.28	-0.25	-0.33	-0.21	0.05	-0.26	-0.13	-0.13	-0.09	0.81	0.80	-0.32	Stable	1.00
THBS2	-0.11	-0.51	-0.87	0.76	-0.70	-0.32	-0.28	-0.38	-0.77	-0.44	-0.78	-0.11	-0.19	0.00	-0.75	-0.17	0.31	1.89	0.92	Stable	1.00
ENSSSCG00000004016	0.56	0.63	0.43	0.04	0.48	0.39	0.64	0.51	0.63	0.72	-0.05	0.61	0.52	0.72	0.56	0.33	0.07	1.54	0.62	Stable	1.00
SFT2D1	-0.38	-0.09	-0.17	-0.06	-0.45	0.03	-0.37	-0.08	-0.26	-0.13	-0.28	-0.37	-0.24	-0.35	-0.29	-0.30	0.76	1.10	0.14	Stable	1.00
QKI	0.15	0.05	0.16	0.18	0.13	-0.17	0.15	-0.55	-0.08	-0.29	0.15	0.13	-0.06	0.15	-0.04	0.12	0.81	-0.05	-4.46	Stable	1.00
TCP1	0.39	0.17	0.30	-0.34	-0.36	0.04	-0.06	-0.28	-0.43	-0.45	0.08	0.00	-0.11	0.47	-0.42	-0.36	0.20	-0.08	-3.57	Stable	1.00
ARID1B	0.04	-0.12	0.02	0.18	0.04	0.09	-0.12	0.00	-0.01	-0.14	-0.17	0.00	0.22	0.21	0.04	0.04	0.68	0.25	-2.01	Stable	1.00
CCDC170	-0.03	-0.15	-0.49	-0.22	-0.22	-0.22	0.13	0.01	-0.01	-0.05	-0.09	-0.29	-0.22	-0.28	-0.15	-0.22	0.37	0.63	-0.66	Stable	1.00
ARMT1	0.20	-0.09	0.04	0.16	0.16	0.16	0.18	0.16	0.14	0.12	0.22	0.17	0.16	0.16	0.08	0.16	0.15	0.66	-0.61	Stable	1.00
RMND1	0.01	-0.01	-0.06	-0.06	-0.06	-0.06	0.36	0.25	0.15	0.00	0.05	-0.06	-0.06	-0.06	0.06	-0.06	0.05	-2.15	1.10	Stable	1.00
PCMT1	0.00	-0.26	0.00	-0.07	-0.56	-0.56	-0.41	-0.25	-0.24	-0.29	-0.22	0.00	-0.07	-0.27	-0.22	-0.56	0.41	0.71	-0.49	Stable	1.00
KATNA1	0.06	-0.53	0.21	-0.09	-0.57	-0.51	0.19	0.21	-0.36	0.77	-0.40	0.93	0.23	0.19	0.14	-0.57	0.87	-0.56	-0.84	Stable	1.00
NUP43	-0.21	-0.05	-0.25	-0.29	-0.10	-0.10	-0.51	-0.48	-0.01	-0.76	0.03	-0.53	-0.08	-0.37	-0.48	-0.10	0.64	1.24	0.31	Stable	1.00
FUCA2	0.01	0.01	0.01	0.13	-0.52	-0.51	0.01	0.03	0.01	0.14	-0.17	0.01	0.01	0.01	0.01	-0.53	0.11	-0.06	-3.99	Stable	1.00
PEX3	-0.11	-0.11	-0.11	0.05	0.32	0.41	-0.10	-0.45	-0.11	-0.47	-0.11	-0.12	-0.11	-0.10	-0.11	0.32	0.14	-4.31	2.11	Stable	1.00
AIG1	-0.20	-0.09	-0.14	-0.30	-0.82	-0.61	-0.14	-0.13	-0.20	-0.31	-0.63	-0.20	-0.20	-0.14	-0.20	-0.85	0.01	0.31	-1.67	Down	1.00
NHSL1	-0.42	-0.56	-0.40	-0.30	-0.80	-0.84	-0.60	-0.64	-0.72	-0.18	-0.23	-0.42	0.00	-0.70	-0.70	-0.80	0.26	1.33	0.41	Stable	1.00
ENSSSCG00000004150	-0.22	-0.47	-0.22	-0.29	-0.79	-0.71	-0.47	-0.18	-0.34	0.03	-0.22	-0.22	-0.09	-0.69	-0.43	-0.94	0.85	0.93	-0.10	Stable	1.00
PEX7	0.08	0.68	0.25	0.07	-0.05	0.17	0.03	0.16	0.26	0.15	-0.07	0.07	0.11	-0.04	0.22	0.00	0.10	3.85	1.95	Stable	1.00
AHI1	0.74	0.42	0.55	0.54	0.60	1.10	0.65	1.04	0.66	0.65	0.85	0.85	0.62	1.18	0.74	0.65	0.90	1.02	0.03	Stable	1.00
SLC18B1	-0.84	-0.44	-0.96	-1.31	-1.66	-0.89	0.06	-0.65	-0.63	-0.34	-1.09	-0.84	-0.97	0.14	-0.10	-1.45	0.01	0.40	-1.32	Down	1.00
MOXD1	0.19	0.10	0.20	-0.34	-0.27	-0.14	0.19	-0.58	-0.43	-0.37	0.20	0.19	0.17	0.05	-0.32	-0.43	0.76	0.62	-0.68	Stable	1.00
ENPP1	0.03	-0.54	-0.11	0.13	0.14	-0.41	-0.63	0.22	-0.09	-0.23	-0.19	0.03	-0.49	-0.02	0.13	0.38	0.75	1.61	0.69	Stable	1.00
HINT3	0.00	-1.62	-0.50	-0.33	-0.33	-0.22	-0.12	-0.33	-0.20	-0.21	-0.01	-0.74	-0.33	-0.12	-0.09	-0.39	0.80	1.17	0.22	Stable	1.00
NCOA7	0.29	-0.24	0.04	-0.07	-0.07	0.07	0.07	-0.07	0.15	0.08	-0.08	-0.12	-0.07	0.07	0.18	-0.01	0.17	-1.69	0.76	Stable	1.00
SMPDL3A	0.00	-0.92	-0.34	-1.87	-1.19	-1.19	-0.67	-1.18	-0.90	-1.25	-1.37	-0.36	-0.95	-0.41	-1.65	-1.53	0.08	0.63	-0.68	Stable	1.00
FABP7	0.00	0.43	0.14	-0.37	0.32	0.50	0.47	0.29	0.30	-0.13	0.27	0.36	0.04	0.44	-0.21	0.25	0.57	1.49	0.58	Stable	1.00

SERINC1	0.00	-0.35	-0.68	-0.17	-0.25	-0.11	-0.33	-0.21	0.01	-0.40	-0.32	0.03	-0.43	-0.11	-0.25	-0.25	0.95	1.02	0.03	Stable	1.00
TBC1D32	0.00	-0.25	-0.25	-0.25	0.00	0.00	0.00	0.00	0.00	0.00	-0.24	0.00	-0.51	0.00	0.00	0.00	0.45	0.51	-0.99	Stable	1.00
RIMS1	-0.34	0.09	-0.10	-0.10	-0.13	0.00	-0.05	-0.13	0.27	0.00	-0.12	-0.22	-0.10	-0.22	-0.08	-0.13	0.68	0.71	-0.50	Stable	1.00
KCNQ5	0.01	0.23	-0.09	-0.13	0.01	-0.13	0.01	-0.25	-0.02	0.00	0.01	-0.09	-0.25	-0.13	-0.22	0.01	0.82	0.80	-0.33	Stable	1.00
NT5E	0.00	0.00	0.00	0.06	0.06	0.06	0.06	0.00	0.00	0.00	0.12	0.06	0.06	0.00	0.00	0.00	0.01	0.14	-2.81	Down	1.00
SNX14	1.01	1.54	1.47	0.25	0.75	1.56	0.24	0.80	0.34	0.62	0.60	0.76	0.90	1.10	0.76	0.82	0.57	1.16	0.21	Stable	1.00
SLC35A1	-0.52	-0.29	-0.36	-0.06	-0.24	-0.16	0.05	-0.35	-0.18	0.23	-0.14	-0.54	-0.02	-0.44	-0.04	-0.24	0.27	1.82	0.86	Stable	1.00
SPACA1	-0.50	-0.09	-0.03	0.50	0.48	0.05	0.45	0.31	-0.02	0.25	0.69	0.07	0.20	-0.05	0.18	0.48	0.03	0.09	-3.47	Down	1.00
PM20D2	-0.08	0.11	0.11	0.36	0.19	0.15	0.00	-0.09	-0.81	-0.11	0.47	0.07	0.44	0.11	-0.38	0.22	0.02	-0.57	-0.80	Stable	1.00
RRAGD	0.01	-0.06	0.01	0.01	-0.10	-0.03	-0.06	-0.24	-0.25	0.01	-0.10	-0.10	-0.10	0.01	-0.24	-0.10	0.41	1.63	0.70	Stable	1.00
MMS22L	0.00	-0.01	0.08	-0.10	-0.21	-0.03	0.00	-0.05	-0.29	-0.30	0.03	0.03	0.00	0.03	0.10	-0.21	0.22	0.17	-2.52	Stable	1.00
FAXC	0.00	0.00	-0.11	-0.49	-0.32	-0.33	0.00	-0.42	-0.65	-0.27	-0.30	-0.08	-0.29	-0.16	-0.47	-0.38	0.42	0.73	-0.45	Stable	1.00
USP45	0.04	0.04	-0.29	-0.61	-0.63	-0.01	0.04	-0.41	-0.61	-0.22	-0.58	-0.35	-0.60	-0.27	-0.23	-0.54	0.07	0.48	-1.06	Stable	1.00
ENSSSCG00000004355	0.23	0.23	0.30	-0.08	-0.27	0.46	0.23	-0.26	0.48	0.17	-0.57	-0.17	-0.32	-0.17	0.53	-0.62	0.04	-1.13	0.18	Stable	1.00
POPDC3	-0.65	-0.04	-0.31	-0.10	-0.13	-0.12	-0.15	0.15	0.20	-0.17	0.34	-0.18	0.34	-0.30	-0.04	-0.06	0.31	14.73	3.88	Stable	1.00
PREP	0.50	0.17	0.38	0.30	0.47	0.24	0.28	0.21	0.17	0.25	0.29	0.50	0.27	0.26	0.17	0.47	0.18	0.77	-0.38	Stable	1.00
QRSL1	0.52	-0.91	0.52	0.43	0.43	-0.08	-0.18	-0.24	-0.42	-0.24	0.43	0.48	0.24	-0.43	-0.91	0.43	0.04	-0.95	-0.08	Stable	1.00
OSTM1	-0.12	0.02	0.31	1.05	0.20	0.50	-0.03	0.35	0.11	0.66	0.77	0.20	0.77	0.37	0.17	0.72	0.00	0.24	-2.04	Down	1.00
NR2E1	0.02	0.02	0.02	0.36	0.16	0.32	0.01	0.14	0.12	0.17	0.32	0.17	0.32	0.16	0.00	0.36	0.00	0.23	-2.12	Down	1.00
GTF3C6	0.00	-0.18	-0.10	-0.10	-0.09	-0.27	0.16	-0.31	-0.44	0.01	-0.04	-0.14	-0.24	0.04	-0.10	-0.17	0.87	0.90	-0.14	Stable	1.00
SLC22A16	0.00	0.06	0.22	1.11	0.21	0.47	0.00	0.28	0.62	0.51	0.59	0.23	0.56	0.21	0.03	0.95	0.01	0.30	-1.72	Down	1.00
DDO	0.00	-0.74	-0.25	-0.72	-0.19	-0.64	0.03	-0.88	-1.08	-0.23	-0.67	-0.17	-0.61	-0.22	-0.81	-0.63	0.95	1.02	0.03	Stable	1.00
METTL24	-0.23	0.26	0.25	1.01	0.40	0.66	-0.05	0.72	0.78	0.48	0.74	0.11	0.85	0.40	0.41	0.82	0.07	0.50	-1.00	Stable	1.00
FIG4	0.00	0.16	-0.04	-0.38	-0.12	-0.27	-0.02	-0.24	-0.11	-0.23	-0.29	-0.08	-0.33	-0.16	-0.03	-0.31	0.00	0.23	-2.13	Down	1.00
MICAL1	-0.05	-0.01	-0.03	-0.05	-0.02	-0.04	-0.01	-0.02	-0.01	-0.02	-0.04	-0.04	-0.05	-0.02	0.00	-0.04	0.04	0.56	-0.85	Stable	1.00
PPIL6	-0.22	0.28	-0.07	-0.34	0.07	0.02	-0.16	0.19	0.21	-0.12	-0.16	-0.10	-0.16	-0.05	0.21	0.00	0.09	-0.50	-1.01	Stable	1.00
ENSSSCG00000004415	-1.02	-0.28	-0.45	-0.22	-0.12	0.30	-0.81	-0.15	-0.40	-0.52	-0.22	-0.41	-0.08	-0.17	-0.74	0.33	0.03	4.32	2.11	Up	1.00
SLC16A10	0.00	-0.36	-0.24	-0.55	-0.28	-0.40	-0.14	-0.44	-0.56	-0.43	-0.40	-0.21	-0.40	-0.36	-0.40	-0.48	0.27	0.78	-0.35	Stable	1.00
TRAF3IP2	0.00	-0.10	0.10	0.18	-0.10	0.21	-0.10	0.14	0.04	0.00	0.17	0.10	0.17	-0.10	-0.07	0.21	0.03	-0.11	-3.24	Down	1.00
FYN	0.29	0.07	0.14	-0.06	0.05	-0.08	0.29	-0.09	-0.06	0.12	-0.08	0.10	-0.08	0.05	0.08	-0.06	0.10	-8.18	3.03	Stable	1.00
NT5DC1	-0.46	-0.20	-0.48	-0.64	-0.10	-0.54	-0.13	-0.77	-0.67	0.21	-0.91	-0.69	-1.02	0.20	-0.30	-0.51	0.36	0.67	-0.58	Stable	1.00
SNAP91	0.00	-0.04	0.14	0.27	-0.04	0.15	-0.04	0.00	0.13	0.10	0.08	0.00	0.13	0.10	0.00	0.08	0.18	0.37	-1.43	Stable	1.00
IBTK	-0.96	-0.44	-0.75	-0.56	-0.29	-0.98	-0.91	0.03	-0.42	-0.14	-0.54	-0.28	-0.48	-0.50	-0.16	0.11	0.49	1.30	0.38	Stable	1.00
BCKDHB	-0.65	0.37	-0.44	-0.03	-0.06	-0.11	-0.35	0.15	0.23	0.00	-0.13	-0.31	-0.11	-0.08	0.23	-0.17	0.72	0.56	-0.83	Stable	1.00
IRAK1BP1	-0.50	-0.25	-0.12	-0.67	-0.28	-0.10	-0.33	-0.28	-0.11	-0.62	-0.22	-0.10	-0.34	-0.22	-0.45	-0.32	0.59	0.85	-0.24	Stable	1.00
HTR1B	0.51	0.00	0.00	0.00	0.00	0.00	0.25	0.00	0.00	0.00	0.00	0.00	0.00	0.00	0.00	0.00	0.20	Inf	Inf	Stable	1.00
SETBP1	0.20	0.36	0.00	-0.03	0.32	-0.10	0.46	0.42	0.03	0.24	-0.10	0.24	-0.10	0.33	0.71	-0.04	0.03	5.80	2.54	Up	1.00
HAUS1	0.05	0.20	0.14	0.44	0.59	0.30	0.38	0.38	0.35	0.54	0.55	0.33	0.67	0.26	0.31	0.44	0.00	0.54	-0.90	Stable	1.00
CCDC68	-0.01	0.25	-0.39	0.03	0.03	-0.13	-0.13	-0.37	-0.03	0.03	0.03	0.01	0.03	0.02	0.04	-0.08	0.37	21.53	4.43	Stable	1.00
HERC1	-0.26	-0.26	-0.26	-0.26	-0.26	-0.26	-0.26	-0.26	-0.26	-0.26	-0.26	-0.26	-0.26	-0.26	-0.13	-0.26	0.35	0.94	-0.09	Stable	1.00
LACTB	-0.29	-0.19	-0.23	-0.18	-0.14	-0.09	-0.14	-0.19	-0.15	-0.12	-0.18	-0.14	0.02	-0.30	-0.29	-0.09	0.01	1.96	0.97	Stable	1.00
ICE2	0.00	0.10	0.18	0.68	0.00	0.11	0.00	0.00	0.09	0.00	0.23	0.00	0.00	0.11	0.00	0.51	0.21	0.31	-1.68	Stable	1.00
ANXA2	0.00	-0.14	-0.28	-0.28	-0.14	-0.28	0.00	-0.14	-0.14	-0.14	-0.28	-0.14	-0.28	-0.14	0.00	-0.28	0.01	0.46	-1.11	Down	1.00
BNIP2	0.00	-0.28	-0.20	-0.04	0.03	-0.04	-0.08	0.01	-0.22	-0.02	-0.04	0.14	-0.04	-0.20	-0.07	-0.04	0.02	14.53	3.86	Up	1.00
GCNT3	0.00	-0.86	-0.55	-0.34	-0.07	-0.47	-0.45	-0.69	-0.68	-0.24	-0.41	-0.10	-0.47	-0.41	-0.45	-0.47	0.11	1.58	0.66	Stable	1.00

RNF111	0.00	0.00	0.00	0.00	0.01	0.00	0.00	0.01	0.00	0.01	0.01	0.01	0.01	0.00	0.00	0.00	0.18	0.33	-1.58	Stable	1.00
LIPC	-0.09	0.73	0.36	1.22	0.53	0.77	0.48	0.72	0.75	0.58	1.81	0.35	1.34	0.17	-0.08	0.57	0.03	0.42	-1.24	Down	1.00
RAB27A	0.00	-0.17	0.00	-0.08	-0.08	-0.17	-0.08	-0.17	-0.17	-0.08	-0.17	-0.08	-0.17	-0.08	-0.08	-0.08	0.49	0.82	-0.29	Stable	1.00
SLC12A1	0.62	0.00	0.31	0.21	0.25	0.00	0.18	0.00	0.01	0.30	0.00	0.31	0.00	0.24	0.13	0.31	0.88	1.08	0.11	Stable	1.00
GATM	0.36	0.21	0.58	0.24	0.27	0.51	0.36	0.48	0.02	0.29	0.27	0.33	0.54	0.42	0.54	0.48	0.95	1.01	0.02	Stable	1.00
SLC28A2	0.03	-0.25	-0.06	-0.10	-0.18	0.00	0.03	0.07	-0.19	-0.36	-0.11	0.06	0.02	0.07	-0.12	0.02	0.64	0.63	-0.68	Stable	1.00
SHF	0.21	-0.10	-0.01	0.00	0.03	0.00	0.21	0.00	0.10	0.10	0.00	0.10	0.00	0.04	0.08	0.00	0.39	2.27	1.18	Stable	1.00
DUOXA2	0.01	-0.17	-0.17	-0.26	0.15	0.12	0.01	0.09	0.04	0.19	-0.03	0.00	0.02	-0.14	0.24	-0.22	0.94	2.62	1.39	Stable	1.00
DUOX2	0.08	-0.07	-0.05	-0.47	0.05	-0.19	0.08	0.05	-0.02	0.06	-0.23	-0.12	-0.23	-0.01	0.11	-0.47	0.02	-0.12	-3.11	Down	1.00
SERINC4	-0.06	0.33	-0.02	-0.30	0.21	0.10	-0.06	0.11	0.08	0.09	0.10	0.16	0.30	0.35	0.37	-0.41	0.34	4.79	2.26	Stable	1.00
STRC	0.00	0.00	0.00	-0.19	-0.12	-0.36	0.00	-0.12	0.00	-0.12	-0.12	0.00	-0.25	0.00	-0.12	-0.62	0.03	0.14	-2.85	Down	1.00
TMEM62	0.00	0.01	0.00	0.26	0.05	0.58	-0.01	0.16	0.28	0.28	0.27	0.05	0.28	0.00	0.43	0.36	0.08	0.41	-1.27	Stable	1.00
LRRC57	0.00	-0.19	-0.19	0.00	-0.02	0.00	0.00	-0.09	0.00	0.00	-0.02	-0.02	-0.09	-0.19	0.00	0.00	0.11	4.23	2.08	Stable	1.00
VPS39	0.05	-0.03	-0.03	-0.15	-0.14	-0.29	0.05	-0.14	-0.04	-0.02	-0.27	-0.19	-0.25	-0.03	-0.14	-0.19	0.00	0.20	-2.32	Down	1.00
PLA2G4F	0.71	0.59	0.58	0.14	0.60	0.11	0.71	0.76	0.50	0.63	0.47	0.76	0.67	0.58	0.57	0.27	0.11	1.37	0.45	Stable	1.00
SPTBN5	-0.22	-0.13	-0.10	-1.01	-0.16	-0.75	-0.22	-0.18	-0.21	-0.06	-0.61	-0.20	-0.08	-0.13	-0.05	-0.66	0.06	0.35	-1.51	Stable	1.00
MAPKBP1	-0.12	0.14	0.15	0.02	-0.02	0.11	-0.12	-0.06	-0.08	-0.02	-0.02	-0.06	0.07	0.08	-0.07	0.08	0.54	-0.48	-1.05	Stable	1.00
EXD1	0.00	0.00	0.00	0.33	-0.43	0.33	0.00	-0.43	-0.03	0.11	-0.13	-0.43	-0.67	0.00	0.16	0.33	0.85	0.57	-0.80	Stable	1.00
GPR176	0.00	0.00	0.00	0.39	0.17	0.39	0.00	0.16	0.06	0.12	0.52	0.19	0.38	0.00	0.19	0.39	0.00	0.16	-2.63	Down	1.00
LINS1	0.43	0.50	0.58	0.35	0.16	0.56	0.45	0.66	0.45	0.51	0.33	0.43	0.70	0.50	0.32	0.56	0.61	1.08	0.11	Stable	1.00
CNDP2	0.45	0.13	0.22	-0.11	-0.07	-0.34	0.44	0.17	0.17	-0.03	0.45	-0.29	0.16	0.12	0.00	0.16	0.06	-22.49	4.49	Stable	1.00
CYB5A	-0.43	0.32	-0.20	-0.21	-0.21	-0.18	-0.21	-0.27	0.32	-0.42	-0.43	-0.29	-0.23	-0.25	0.00	-0.18	0.12	0.34	-1.58	Stable	1.00
ENSSSCG00000004911	-0.84	0.11	-0.36	-0.62	-1.13	-0.87	-0.86	-0.90	-0.61	-0.76	-1.05	-1.32	-0.70	-0.41	-0.84	-1.07	0.04	0.63	-0.67	Stable	1.00
PDCD7	-0.20	-0.20	-0.20	0.00	-0.15	0.05	-0.20	-0.05	-0.20	0.05	0.00	-0.10	0.01	-0.20	-0.20	0.01	0.00	11.16	3.48	Up	1.00
TIPIN	0.00	0.00	0.00	0.15	-0.03	0.10	0.00	0.03	0.00	0.29	-0.05	-0.02	-0.07	0.00	0.00	-0.05	0.45	0.09	-3.41	Stable	1.00
ZWILCH	0.09	0.00	0.04	0.51	-0.01	0.13	0.09	0.27	0.04	0.44	0.26	-0.04	-0.09	0.00	0.09	-0.11	0.54	0.58	-0.80	Stable	1.00
ITGA11	0.38	0.08	0.45	-0.26	0.08	-0.26	0.42	0.29	0.21	-0.26	-0.26	0.08	-0.26	0.24	0.42	-0.26	0.00	-1.80	0.85	Stable	1.00
NOX5	-0.14	-0.15	-0.13	0.29	-0.03	0.19	-0.14	-0.14	-0.14	0.19	0.39	0.07	0.19	-0.14	-0.14	0.29	0.00	-0.70	-0.52	Stable	1.00
UACA	-0.29	-0.39	-0.29	-0.61	-0.45	-0.61	-0.29	-0.29	-0.29	-0.21	-0.59	-0.38	-0.61	-0.29	-0.29	-0.59	0.01	0.60	-0.73	Stable	1.00
GEMIN2	-0.54	-0.54	-0.54	-0.54	-0.54	-0.54	-0.54	-0.54	-0.54	-0.27	-0.54	-0.54	-0.54	-0.54	-0.54	-0.54	0.39	1.06	0.09	Stable	1.00
LRR1	-0.22	-0.22	-0.11	0.00	-0.17	0.00	-0.22	-0.22	-0.22	0.00	0.00	-0.11	0.00	-0.22	-0.22	0.00	0.00	5.92	2.56	Up	1.00
NEMF	0.57	0.44	0.28	0.22	0.40	0.47	0.57	0.51	0.57	-0.15	0.68	0.71	0.74	0.51	0.57	1.01	0.96	0.99	-0.02	Stable	1.00
SAV1	-1.13	-0.86	-1.22	-1.28	-0.71	-0.86	-1.13	-0.96	-1.13	-0.28	0.06	-0.48	-0.73	-1.01	-1.13	0.17	0.01	2.08	1.06	Up	1.00
ABHD12B	-0.40	-0.29	-0.25	0.55	0.03	0.19	-0.40	-0.35	-0.40	0.49	0.18	-0.29	0.32	-0.35	-0.40	-0.18	0.00	-2.23	1.16	Up	1.00
RTRAF	-0.13	-0.55	-0.85	-0.19	-0.44	-0.50	-0.13	-0.27	0.04	-0.50	0.47	-0.13	-0.28	-0.65	-0.13	0.17	0.35	1.91	0.94	Stable	1.00
TMEM260	0.00	-0.06	0.00	0.18	0.09	0.18	0.00	-0.11	0.00	0.18	0.18	0.09	0.18	-0.11	0.04	0.18	0.00	-0.20	-2.33	Down	1.00
PCNX4	-0.76	-0.70	-0.76	-0.69	-0.14	-0.41	-0.76	-0.70	-0.76	-0.35	-1.07	-0.35	-0.46	-0.47	0.14	-0.33	0.43	1.26	0.33	Stable	1.00
DHRS7	0.10	0.10	0.10	0.40	0.29	0.42	0.10	0.10	0.10	0.29	0.29	0.31	0.21	0.10	0.15	0.42	0.00	0.32	-1.66	Down	1.00
ENSSSCG00000005103	0.31	0.40	0.20	0.02	0.79	-0.06	0.31	0.40	0.31	0.95	-0.06	0.14	0.14	0.40	1.05	-0.06	0.29	1.80	0.85	Stable	1.00
ESR2	0.24	0.18	0.17	0.11	0.15	0.12	0.24	0.06	0.24	0.56	-0.16	-0.13	0.11	0.06	0.24	0.14	0.43	1.61	0.69	Stable	1.00
IFN-DELTA-1	0.00	-0.03	0.00	0.90	0.54	0.53	0.00	0.59	0.00	-0.16	0.44	0.16	0.66	0.16	-0.20	0.32	0.02	0.16	-2.69	Down	1.00
SNAPC3	-0.14	-0.14	-0.14	0.06	0.50	0.13	-0.14	-0.14	-0.14	0.50	0.13	-0.01	0.06	-0.14	-0.14	0.13	0.00	-0.77	-0.38	Stable	1.00
TTC39B	-0.62	-0.60	-0.62	-0.22	-0.10	-0.22	-0.62	-0.62	-0.56	-0.10	-0.23	-0.42	-0.22	-0.62	-0.30	-0.22	0.00	2.65	1.41	Up	1.00
IL33	0.00	0.00	0.00	-0.39	0.00	0.00	0.00	0.00	0.00	0.00	0.00	0.00	-0.36	0.00	0.00	0.00	0.17	0.00	#NAME?	Stable	1.00
ENSSSCG00000005216	-0.14	0.38	0.11	-0.02	-0.12	0.25	0.38	-0.15	-0.09	0.24	-0.12	0.28	0.12	0.38	-0.31	0.04	0.92	0.86	-0.22	Stable	1.00

SPATA6L	-0.09	0.54	0.29	0.77	0.62	-0.35	-0.63	0.79	-0.26	-0.67	1.17	0.33	1.22	0.07	0.17	0.22	0.31	0.27	-1.91	Stable	1.00
SLC1A1	-0.20	-0.57	-0.39	-0.47	-0.47	-0.47	-0.57	-0.04	-0.57	-0.52	-0.47	-0.49	-0.47	-0.57	-0.23	-0.47	0.28	0.82	-0.29	Stable	1.00
NMRK1	-0.17	-0.24	-0.17	0.23	0.01	-0.06	-0.11	-0.24	-0.17	-0.03	0.15	0.06	0.23	-0.17	-0.17	0.15	0.00	-1.96	0.97	Stable	1.00
CCDC107	-0.03	-0.01	-0.01	0.42	-0.02	-0.14	-0.19	-0.03	-0.19	0.27	0.38	0.00	0.50	-0.10	-0.03	0.11	0.02	-0.38	-1.40	Down	1.00
RGP1	0.17	0.20	0.20	-0.34	-0.11	-0.07	-0.24	-0.08	-0.24	-0.08	-0.04	-0.25	-0.53	-0.08	0.12	-0.38	0.02	-0.03	-5.00	Down	1.00
GRHPR	-0.13	-0.04	0.07	-0.31	-0.50	-0.50	0.17	-0.35	0.18	0.00	-0.50	-0.18	-0.31	-0.06	-0.19	-0.31	0.01	0.14	-2.89	Down	1.00
ALDH1B1	-0.14	-0.16	-0.30	-0.18	-0.10	-0.09	-0.41	-0.27	-0.54	-0.35	-0.32	-0.48	-0.49	-0.01	-0.20	-0.10	0.90	0.96	-0.06	Stable	1.00
TSTD2	0.05	0.40	0.27	0.58	0.58	0.76	0.28	0.14	0.56	0.57	0.33	0.47	0.80	0.02	0.03	0.28	0.00	0.41	-1.30	Down	1.00
XPA	0.26	0.16	0.37	0.28	0.00	-0.17	0.37	0.08	0.63	0.15	0.09	0.13	0.31	-0.04	0.30	0.29	0.18	1.96	0.97	Stable	1.00
TRMO	0.47	0.00	0.49	0.63	0.68	1.13	0.16	-0.02	0.33	0.64	0.82	0.34	0.40	0.00	0.56	0.40	0.01	0.39	-1.35	Down	1.00
ALG2	0.64	0.78	0.88	0.02	0.51	0.37	-0.12	0.18	-0.11	0.45	0.38	0.49	0.19	0.68	0.76	0.45	0.52	1.29	0.37	Stable	1.00
SMC2	0.21	0.19	0.11	0.10	0.06	0.14	-0.22	0.01	0.17	0.25	0.19	-0.15	0.13	-0.14	0.26	-0.01	0.85	0.84	-0.25	Stable	1.00
ENSSSCG00000005440	-1.39	0.03	-0.08	-0.16	-0.71	-1.13	-0.09	-0.02	-0.01	-1.07	-0.11	-0.68	-0.11	-0.05	-1.39	-0.64	0.46	0.65	-0.62	Stable	1.00
ELP1	-0.07	-0.18	0.02	-0.02	0.06	0.20	-0.38	0.06	0.04	0.04	0.05	-0.39	0.05	-0.19	-0.06	-0.07	0.32	10.33	3.37	Stable	1.00
C9orf152	0.00	-0.01	-0.13	0.25	0.10	0.03	0.02	0.11	0.27	0.02	0.11	0.00	0.24	0.14	0.00	0.25	0.22	0.40	-1.30	Stable	1.00
ENSSSCG00000005453	0.13	0.16	0.10	0.07	-0.06	-0.07	0.00	0.14	0.10	0.00	0.07	0.00	-0.02	0.07	0.08	0.07	0.00	14.19	3.83	Up	1.00
SHOC1	0.43	0.56	0.20	0.53	0.49	0.26	0.24	0.41	0.35	0.04	0.46	0.07	0.40	0.41	0.43	0.41	0.55	1.14	0.19	Stable	1.00
SLC46A2	0.07	0.13	0.07	0.07	0.02	0.01	0.01	0.21	0.18	0.00	0.05	0.03	0.07	0.04	0.07	0.03	0.04	2.68	1.42	Up	1.00
SAL1	-0.14	0.12	0.04	0.61	0.34	0.09	-0.24	0.10	0.08	-0.08	0.15	-0.02	0.15	-0.05	-0.05	0.57	0.04	-0.08	-3.67	Down	1.00
ENSSSCG00000005475	-0.69	-0.35	-0.56	-0.38	-0.53	-0.54	-0.94	-0.12	-0.45	-0.68	-0.47	-0.63	-0.45	-0.46	-0.58	-0.19	0.74	1.07	0.10	Stable	1.00
RGS3	0.03	0.30	-0.11	0.18	-0.05	-0.14	-0.11	0.39	0.33	-0.04	-0.28	-0.12	-0.23	0.21	-0.14	0.34	0.17	-2.60	1.38	Stable	1.00
ASTN2	0.34	0.40	-0.15	0.57	0.51	0.02	0.51	0.38	0.00	0.25	0.51	0.17	0.00	0.51	-0.16	0.00	0.85	0.90	-0.15	Stable	1.00
TRAF1	-0.14	0.10	0.33	0.31	0.14	0.10	-0.04	0.01	-0.07	-0.18	-0.08	0.36	0.14	0.36	-0.06	-0.03	0.72	0.64	-0.65	Stable	1.00
MRRF	0.00	0.01	0.00	0.02	0.02	0.02	-0.18	0.01	0.00	0.00	0.01	0.02	0.02	0.02	0.00	0.00	0.24	-1.50	0.58	Stable	1.00
OLFML2A	0.09	-0.10	-0.43	-0.22	-0.10	-0.10	-0.44	0.12	0.08	0.12	0.11	-0.17	-0.13	0.12	0.05	0.03	0.95	1.12	0.16	Stable	1.00
PPP6C	-0.15	0.02	-0.26	0.49	0.06	0.08	0.48	0.31	-0.10	0.14	-0.09	0.27	0.20	0.44	0.06	-0.08	0.78	0.74	-0.43	Stable	1.00
PBX3	0.15	-0.03	-0.08	0.43	0.03	-0.13	0.10	-0.47	-0.19	-0.32	0.67	0.63	0.15	-0.28	0.05	0.31	0.05	-0.42	-1.25	Stable	1.00
LRSAM1	-0.35	-0.43	-0.27	-0.19	-0.36	-0.29	-0.25	-0.45	0.18	-0.41	-0.39	-0.41	-0.24	-0.49	-0.37	-0.36	0.75	0.92	-0.12	Stable	1.00
TTC16	0.74	1.08	1.99	1.82	1.89	1.74	0.64	1.46	1.28	1.44	1.94	0.99	1.87	1.41	0.54	1.78	0.02	0.68	-0.56	Stable	1.00
TOR2A	0.08	0.02	0.06	0.36	0.46	0.49	0.04	0.22	0.21	0.22	0.51	0.25	0.49	0.14	0.20	0.36	0.00	0.31	-1.70	Down	1.00
ENSSSCG00000005623	0.28	0.64	-0.23	0.37	0.44	0.72	-0.01	0.97	0.76	0.48	0.61	0.36	0.85	0.94	0.20	0.12	0.79	0.90	-0.15	Stable	1.00
CIZ1	1.05	0.32	1.08	1.09	0.46	1.11	0.67	0.72	0.86	1.16	0.64	1.09	1.00	1.59	0.51	0.64	0.77	0.94	-0.08	Stable	1.00
TRUB2	0.30	0.19	0.09	0.26	0.40	0.44	-0.21	-0.06	0.40	-0.05	0.45	0.07	0.30	-0.20	0.14	0.32	0.08	0.30	-1.73	Stable	1.00
URM1	0.94	0.72	1.12	0.71	0.92	1.12	0.55	0.65	1.15	0.71	0.50	0.66	0.89	0.28	1.05	0.70	0.80	1.04	0.06	Stable	1.00
GLE1	1.12	1.25	1.69	1.81	1.31	1.69	0.83	0.12	0.71	1.05	1.82	1.45	2.45	0.80	0.85	1.11	0.01	0.58	-0.78	Stable	1.00
PKN3	-0.07	-0.20	-0.09	-0.04	-0.05	-0.04	-0.05	-0.07	-0.05	-0.05	-0.06	-0.05	-0.02	-0.10	-0.43	-0.32	0.36	1.69	0.76	Stable	1.00
TBC1D13	-0.42	0.07	0.38	0.31	0.30	-0.08	0.04	0.25	0.06	0.18	0.73	0.13	0.26	-0.35	-0.19	-0.07	0.10	-0.09	-3.40	Stable	1.00
SPOUT1	-0.18	0.08	0.05	0.05	0.09	0.02	0.07	0.08	0.10	-0.02	0.06	0.05	0.07	0.03	0.09	0.04	0.84	0.85	-0.24	Stable	1.00
PHYHD1	0.59	0.54	0.00	0.26	0.42	0.31	0.08	0.53	0.55	0.40	0.25	0.43	0.29	0.44	0.45	0.46	0.62	1.12	0.17	Stable	1.00
NUP188	-0.31	-0.13	-0.03	-0.12	-0.06	-0.32	-0.24	-0.23	-0.06	-0.03	-0.04	-0.24	-0.23	-0.07	-0.24	-0.25	0.95	1.02	0.03	Stable	1.00
CRAT	0.14	0.33	-0.03	-0.17	0.16	0.37	-0.01	0.22	0.10	0.14	0.11	0.01	0.16	0.46	0.23	0.07	0.36	1.70	0.77	Stable	1.00
FNBP1	-0.37	-0.41	-0.36	0.08	-0.14	-0.26	-0.33	-0.30	-0.23	-0.04	-0.08	-0.11	-0.06	-0.33	-0.40	-0.37	0.00	2.74	1.45	Up	1.00
LAMC3	0.09	0.21	0.04	-0.01	-0.03	-0.03	-0.03	0.07	-0.02	0.11	-0.05	-0.04	0.02	0.09	0.20	0.20	0.21	3.59	1.84	Stable	1.00
NUP214	-0.22	-0.22	-0.42	-0.21	-0.13	0.17	-0.21	-0.25	-0.06	-0.30	-0.48	-0.34	-0.37	-0.20	-0.27	-0.26	0.90	0.96	-0.06	Stable	1.00
PRRC2B	0.99	1.08	0.73	0.25	0.63	0.96	0.63	0.52	0.74	0.34	0.11	0.51	-0.27	0.76	1.34	1.03	0.05	1.90	0.93	Stable	1.00

POMT1	0.29	-0.22	-0.49	-0.68	-0.57	-0.35	-0.68	-0.78	-0.65	-0.18	-0.48	-0.01	-0.46	-0.48	0.31	0.24	0.89	1.08	0.11	Stable	1.00
SARDH	-0.47	0.17	-0.83	-0.44	-0.45	-0.44	-0.33	-0.33	-0.38	-0.51	-0.33	-0.68	-0.33	-0.33	-0.70	-0.57	0.56	0.86	-0.22	Stable	1.00
HDHD2	-0.77	-0.17	-0.23	0.12	0.10	0.27	-0.37	-0.08	-0.04	0.09	-0.23	-0.55	-0.13	0.38	0.04	0.10	0.41	5.39	2.43	Stable	1.00
HSBP1L1	0.04	-0.03	0.00	-0.33	-0.33	-0.30	0.02	-0.33	-0.03	-0.16	-0.33	0.00	-0.33	0.02	-0.16	-0.09	0.02	0.25	-1.98	Down	6.00
ZNF7	0.12	0.15	0.24	0.18	0.13	0.04	0.09	0.33	0.26	-0.14	0.21	0.17	0.09	0.10	0.07	0.15	0.24	1.60	0.68	Stable	4.00
ZNF34	1.09	1.83	2.00	3.08	0.92	2.07	1.35	0.60	0.58	2.09	0.15	1.94	1.91	0.64	2.11	1.72	0.25	0.74	-0.44	Stable	4.00
ARHGAP39	0.42	0.39	0.21	0.23	0.16	0.21	0.57	0.31	0.09	0.28	0.17	0.33	0.28	0.42	0.36	0.20	0.08	1.47	0.56	Stable	4.00
MFSD3	0.30	1.00	0.29	0.82	0.01	0.68	0.44	-0.21	0.02	0.37	-0.32	0.94	0.33	-0.10	0.17	0.35	0.44	0.60	-0.73	Stable	4.00
FOXH1	-0.17	-0.45	-0.20	-0.39	-0.04	-0.13	-0.23	-0.04	-0.04	-0.33	-0.09	-0.42	-0.36	-0.04	-0.13	-0.20	0.26	0.66	-0.60	Stable	4.00
SLC39A4	0.26	0.50	0.63	1.01	0.71	0.67	0.22	0.69	0.22	0.48	0.81	0.56	0.45	0.65	0.67	0.75	0.07	0.71	-0.50	Stable	4.00
TMEM249	0.16	0.06	0.05	0.02	0.42	0.21	0.15	0.19	0.06	0.09	0.34	0.10	0.07	0.12	-0.02	0.11	0.22	0.57	-0.81	Stable	4.00
OPLAH	-0.34	-0.54	-0.64	-0.79	-0.61	-0.66	-0.33	-0.47	-0.34	-0.72	-0.68	-0.54	-0.72	-0.52	-0.47	-0.66	0.00	0.68	-0.56	Stable	4.00
GPAA1	-0.26	-0.46	-0.67	-0.55	-0.39	-0.47	-0.15	-0.29	-0.08	-0.49	-0.41	-0.46	-0.38	-0.47	-0.29	-0.45	0.13	0.74	-0.43	Stable	4.00
MROH1	-0.30	-0.34	-0.14	-0.26	-0.13	-0.08	-0.30	-0.15	-0.23	-0.26	-0.38	-0.38	-0.24	-0.21	-0.16	-0.30	0.61	0.91	-0.14	Stable	4.00
KCNQ3	-0.87	-0.19	-0.33	-0.56	-0.27	-0.06	-0.93	-0.42	-0.31	-0.34	-0.08	-0.35	-0.11	-0.27	-0.41	-0.12	0.07	1.96	0.97	Stable	4.00
EFR3A	-0.13	0.02	-0.01	-0.26	-0.10	-0.06	-0.12	-0.05	-0.16	-0.19	-0.06	-0.03	-0.06	-0.07	-0.14	-0.06	0.59	0.80	-0.33	Stable	4.00
ENSSSCG00000005959	0.16	0.20	0.00	0.36	0.04	0.40	0.32	0.20	0.00	0.40	0.40	0.24	0.40	0.00	0.00	0.32	0.00	0.34	-1.55	Down	4.00
WASHC5	-0.37	-0.33	-0.32	-0.62	-0.46	-0.57	-0.57	-0.62	-0.29	-0.65	-0.57	-0.57	-0.57	-0.23	-0.45	-0.41	0.02	0.72	-0.47	Stable	4.00
ENSSSCG00000005982	-1.55	-0.77	-0.62	-1.50	-1.84	-2.06	-2.06	-1.00	-1.07	-1.66	-1.96	-2.06	-2.06	-0.83	-1.43	-0.62	0.04	0.68	-0.56	Stable	4.00
ZHX1	0.32	0.26	0.21	0.23	0.32	0.32	0.32	0.29	0.26	0.32	0.32	0.32	0.32	0.26	0.32	0.20	0.63	0.96	-0.05	Stable	4.00
FAM83A	0.13	0.16	0.39	0.13	0.13	0.13	0.13	0.16	0.26	0.13	0.13	0.13	0.13	0.26	0.13	0.07	0.05	1.64	0.72	Stable	4.00
ENPP2	0.09	-0.03	-0.07	0.09	0.18	0.06	0.18	-0.07	-0.07	0.06	0.18	0.18	0.18	0.09	-0.07	-0.07	0.05	0.05	-4.31	Stable	4.00
AARD	0.49	0.01	-0.02	0.54	1.05	0.78	0.92	-0.58	-0.06	0.37	1.05	1.05	1.05	0.71	-0.06	-0.47	0.07	0.26	-1.94	Stable	4.00
OXR1	0.83	0.00	0.12	0.12	0.00	0.00	0.12	0.00	0.00	0.12	0.12	0.24	0.24	0.12	0.00	0.00	0.69	1.42	0.50	Stable	4.00
ENSSSCG00000006049	0.53	1.34	0.78	0.07	0.61	0.99	0.78	1.26	0.91	0.78	0.07	0.19	0.07	0.78	1.18	0.07	0.00	2.65	1.40	Up	4.00
BAALC	0.09	0.13	0.00	0.09	0.09	0.00	0.13	0.09	0.13	0.25	0.25	0.25	0.25	0.13	0.00	0.25	0.05	0.48	-1.07	Down	4.00
RGS22	0.31	0.14	0.22	0.48	0.36	0.14	0.35	0.11	0.36	0.58	0.58	0.58	0.58	0.26	0.14	0.58	0.00	0.49	-1.03	Down	4.00
RIDA	-0.85	-0.45	-0.44	-0.93	-0.26	-0.13	-0.62	-0.11	-0.85	-0.46	-0.48	-0.45	-0.57	-0.22	-0.03	-0.48	0.88	0.95	-0.07	Stable	4.00
MTERF3	0.80	0.36	0.60	0.29	-0.02	-0.18	0.46	0.68	0.61	0.32	0.32	0.32	0.32	0.46	0.49	0.32	0.00	2.63	1.39	Up	4.00
NDUFAF6	-0.25	-0.18	-0.18	-0.09	-0.09	-0.06	0.09	-0.18	0.25	-0.09	-0.01	-0.09	-0.01	-0.18	0.00	-0.01	0.77	1.33	0.41	Stable	4.00
INTS8	-0.21	-0.20	-0.20	-0.11	-0.01	-0.10	-0.19	-0.20	-0.10	-0.11	-0.01	-0.11	-0.01	-0.20	-0.20	-0.01	0.00	3.19	1.68	Up	4.00
PIP4P2	-1.49	-0.62	-0.69	-1.23	-0.36	-0.19	0.06	-0.71	0.06	-0.20	-0.20	-0.20	-0.20	-0.96	-0.78	-0.20	0.20	1.87	0.90	Stable	4.00
DECR1	-0.06	0.10	0.09	0.10	-0.01	-0.08	0.27	0.10	0.27	0.07	0.07	0.07	0.07	0.10	0.24	0.07	0.07	2.98	1.58	Stable	4.00
CPNE3	-0.53	0.00	0.00	0.03	-0.16	0.00	-0.18	0.12	0.14	-0.08	-0.25	-0.37	-0.06	0.00	-0.18	-0.06	0.66	0.66	-0.59	Stable	4.00
RMDN1	0.05	0.20	0.20	0.20	0.05	0.00	0.10	0.20	0.10	0.20	0.20	0.20	0.20	0.20	0.10	0.20	0.72	0.91	-0.13	Stable	4.00
CA2	-0.26	0.33	0.09	-0.02	0.48	0.19	0.19	-0.14	0.12	0.36	0.79	0.19	-0.10	-0.41	-0.31	-0.10	0.08	-0.22	-2.19	Stable	4.00
CA13	-0.18	-0.43	-0.73	-0.76	-0.17	0.00	-0.06	-0.53	0.11	-0.57	-0.78	-1.02	-1.00	-0.70	-0.48	-0.91	0.13	0.58	-0.80	Stable	4.00
E2F5	0.31	0.06	0.39	0.51	0.11	0.00	0.29	0.06	-0.21	0.32	0.24	0.49	0.30	0.61	0.27	0.30	0.59	0.79	-0.34	Stable	4.00
PKIA	0.00	0.00	0.00	0.00	0.00	0.00	0.00	0.00	0.14	0.36	0.38	0.00	0.00	0.00	0.00	0.00	0.27	0.19	-2.39	Stable	4.00
SEC31B	1.47	0.63	0.99	1.28	0.93	1.25	0.52	1.32	0.99	1.60	0.63	1.53	1.94	1.53	1.78	1.29	0.48	0.88	-0.18	Stable	14.00
PEX2	-0.55	-0.71	-0.78	-0.12	-1.24	-0.49	-1.27	-0.23	-0.82	-1.50	-0.83	-0.45	-0.07	-0.54	-0.81	-0.07	0.60	1.20	0.27	Stable	4.00
TERF1	0.16	-0.04	0.14	0.14	-0.02	0.00	-0.02	0.01	-0.01	-0.04	-0.04	-0.02	-0.04	0.14	0.14	-0.04	0.08	-11.09	3.47	Stable	4.00
MCMDC2	0.07	-0.09	0.15	-0.15	0.04	-0.16	0.02	0.05	-0.57	-0.17	0.05	0.02	0.04	0.13	0.07	0.04	0.87	0.58	-0.80	Stable	4.00
SDCBP	-0.07	0.00	-0.07	-0.04	-0.07	-0.07	0.37	0.00	-0.04	0.01	0.01	-0.04	-0.07	0.00	-0.07	-0.07	0.32	-0.35	-1.49	Stable	4.00
FAM110B	0.96	0.03	0.27	0.10	0.52	0.26	0.13	0.54	0.45	0.32	0.85	0.47	0.52	0.06	0.52	0.52	0.60	0.84	-0.26	Stable	4.00

PENK	0.10	0.26	-0.10	-0.22	0.22	0.12	0.22	0.04	0.20	-0.10	0.18	0.28	0.22	-0.13	0.22	0.22	0.87	0.88	-0.18	Stable	4.00
PLAG1	0.01	0.05	0.11	-0.21	0.01	0.01	0.01	-0.17	-0.38	-0.03	0.22	-0.33	0.01	0.19	0.01	0.01	0.83	0.52	-0.94	Stable	4.00
TGS1	-1.18	-0.88	-0.93	-0.96	-1.13	-0.57	-1.13	-0.56	-0.70	-1.13	-1.24	-0.70	-1.13	-0.97	-1.13	-1.13	0.59	0.94	-0.09	Stable	4.00
SOX17	0.00	0.03	0.05	0.06	0.00	0.02	0.08	0.09	0.12	0.05	0.06	0.04	0.03	0.03	0.05	0.00	0.18	1.72	0.78	Stable	4.00
SPIDR	0.21	0.08	0.07	0.20	0.27	0.24	0.23	0.33	0.31	0.12	0.16	0.37	0.33	0.01	0.12	0.27	0.16	0.69	-0.53	Stable	4.00
METTL11B	0.25	0.33	0.66	0.09	-0.11	0.31	0.36	0.58	0.18	0.10	0.21	0.43	0.20	0.23	0.08	0.00	0.07	2.17	1.12	Stable	4.00
METTL18	-0.06	-0.05	-0.20	-0.05	0.13	0.09	0.00	0.37	0.70	-0.01	-0.05	0.40	-0.03	-0.07	0.01	0.13	0.92	1.16	0.22	Stable	4.00
ENSSSCG00000006286	-0.16	-0.04	-0.30	0.00	0.00	-0.17	-0.16	-0.28	-0.15	-0.15	-0.15	-0.31	-0.15	-0.15	-0.26	0.00	0.17	1.61	0.68	Stable	4.00
F5	0.17	0.13	0.34	0.13	0.00	0.08	0.12	0.35	0.11	0.16	0.08	0.31	0.15	0.08	0.13	0.00	0.24	1.56	0.65	Stable	4.00
SLC19A2	0.13	0.26	0.12	0.27	0.36	0.55	0.17	0.28	0.43	0.21	0.22	0.36	0.22	0.04	0.20	0.36	0.08	0.64	-0.64	Stable	4.00
NME7	0.06	-0.12	-0.18	0.06	0.02	0.02	0.11	-0.15	0.02	-0.01	0.00	0.01	-0.04	-0.02	-0.01	0.02	0.24	-3.88	1.95	Stable	4.00
MPC2	-0.07	0.00	-0.14	-0.68	-0.68	-0.56	-0.07	0.04	0.12	-0.32	-0.30	0.04	-0.33	-0.14	-0.07	-0.68	0.00	0.10	-3.36	Down	4.00
CD247	0.05	-0.05	-0.01	0.23	0.11	0.13	-0.08	-0.13	-0.08	0.08	0.10	0.09	0.08	0.05	-0.05	0.11	0.00	-0.32	-1.66	Down	4.00
FCER1G	-0.04	-0.02	0.00	0.01	0.00	-0.05	-0.04	-0.03	-0.02	0.00	0.00	-0.02	0.00	0.01	-0.02	0.00	0.20	2.64	1.40	Stable	4.00
PPOX	0.00	0.00	0.00	0.12	0.26	0.25	0.00	0.00	0.00	0.26	0.26	0.00	0.26	0.00	0.00	0.26	0.00	0.00	#NAME?	Down	4.00
UFC1	0.34	0.23	0.31	-0.13	0.26	-0.21	0.31	0.34	0.13	0.26	0.26	0.35	0.26	0.54	0.32	0.26	0.09	1.95	0.96	Stable	4.00
F11R	-0.58	-0.74	-0.83	-0.54	-0.64	-0.37	-0.61	-0.74	-0.32	-0.64	-0.64	-0.64	-0.64	-0.64	-0.64	-0.64	0.54	1.07	0.09	Stable	4.00
CFAP45	0.01	1.03	0.68	1.15	1.75	1.45	-0.24	0.71	0.05	1.75	1.75	-0.25	1.75	0.37	0.07	1.75	0.00	0.24	-2.05	Down	4.00
ENSSSCG00000006418	-0.27	-0.30	-0.31	-0.21	-1.00	-0.68	-0.24	0.45	-0.61	-1.00	-1.00	-0.66	-1.00	0.60	-0.72	-1.00	0.01	0.21	-2.22	Down	4.00
OR6N2	0.05	0.28	0.20	0.36	0.30	0.26	0.24	0.74	0.24	0.30	0.30	0.22	0.30	0.61	0.30	0.30	0.63	1.14	0.19	Stable	4.00
CD1E	-0.12	-0.34	-0.24	-0.34	-0.24	-0.24	-0.26	-0.52	-0.26	-0.24	-0.24	-0.38	-0.24	-0.38	-0.38	-0.24	0.38	1.16	0.22	Stable	4.00
KIRREL1	-0.09	-0.38	-0.15	-0.48	-0.35	-0.33	-0.47	-0.63	-0.24	-0.35	-0.35	-0.52	-0.35	-0.47	-0.50	-0.35	0.80	0.95	-0.07	Stable	4.00
GPATCH4	-0.14	0.01	-0.03	0.15	-0.17	-0.13	-0.23	-0.07	-0.06	-0.17	-0.17	-0.17	-0.17	-0.31	-0.22	-0.17	0.84	1.09	0.13	Stable	4.00
HCN3	0.34	-0.16	0.22	0.10	0.04	0.04	0.13	-0.22	-0.14	0.04	0.04	0.18	0.04	0.16	0.13	0.04	0.97	0.95	-0.07	Stable	4.00
GBA	-0.09	-0.28	-0.29	-0.96	-1.00	-0.48	-0.12	0.04	-0.12	-0.98	-1.00	0.02	-1.00	-0.09	0.08	-1.00	0.00	0.13	-2.91	Down	4.00
MTX1	0.49	0.14	0.20	0.17	-0.05	0.13	0.21	0.20	-0.22	-0.05	-0.05	0.34	-0.05	0.24	0.32	-0.05	0.12	4.04	2.01	Stable	4.00
SLC50A1	-0.07	-0.08	-0.03	0.16	0.21	0.34	0.00	-0.10	-0.04	0.21	0.21	-0.07	0.21	-0.07	-0.06	0.21	0.00	-0.30	-1.75	Down	4.00
PMVK	0.00	0.01	-0.32	-0.38	-0.61	-0.21	-0.21	0.37	0.14	-0.61	-0.61	0.06	-0.61	0.02	0.04	-0.61	0.00	-0.01	-6.40	Down	4.00
UBE2Q1	0.04	0.04	0.04	0.02	0.04	0.04	0.04	0.04	0.02	0.04	0.04	0.04	0.04	0.04	0.04	0.04	1.00	1.00	0.00	Stable	4.00
AQP10	-0.11	-0.10	-0.10	0.01	-0.03	-0.07	-0.09	-0.11	-0.05	-0.03	-0.03	-0.11	-0.03	-0.11	-0.11	-0.03	0.00	2.44	1.29	Up	4.00
ENSSSCG00000006564	-0.48	-0.65	-0.52	0.12	0.35	-0.11	-0.66	-0.48	-0.24	0.35	0.35	-0.47	0.35	-0.48	-0.40	0.35	0.00	-3.09	1.63	Up	4.00
PGLYRP4	0.03	0.00	0.03	0.00	0.01	0.03	0.13	0.07	0.00	0.01	0.01	0.03	0.01	0.06	0.03	0.01	0.07	3.95	1.98	Stable	4.00
S100A11	0.66	1.19	1.32	0.71	0.72	1.13	0.33	0.27	0.05	0.64	0.64	0.74	0.64	0.35	0.79	0.64	0.53	0.85	-0.24	Stable	4.00
THEM5	0.60	0.98	0.54	0.60	0.68	0.17	0.12	0.34	0.33	0.91	0.91	0.31	0.91	0.29	0.60	0.91	0.17	0.70	-0.51	Stable	4.00
TUFT1	-0.55	-0.32	-0.36	-0.31	-0.28	-0.51	-0.38	-0.07	-0.29	-0.16	-0.16	-0.54	-0.16	0.00	-0.47	-0.16	0.80	1.08	0.11	Stable	4.00
SETDB1	-0.56	0.01	-0.44	-0.40	-0.30	-0.47	-0.63	-0.61	-0.34	-0.32	-0.32	-0.62	-0.32	-0.60	-0.19	-0.32	0.72	1.09	0.12	Stable	4.00
OTUD7B	0.08	0.01	0.03	0.12	0.18	-0.02	0.02	0.10	0.06	0.22	0.23	0.06	0.23	0.03	-0.01	0.23	0.01	0.27	-1.87	Down	4.00
FCGR1A	-1.43	-0.41	-0.94	-0.37	-0.45	-1.35	-1.60	-1.00	-0.48	-0.03	-0.02	-1.40	0.03	-1.39	-1.46	-0.02	0.03	2.41	1.27	Up	4.00
VTCN1	-0.28	-0.12	-0.60	-0.84	-1.01	-0.83	-0.71	-0.50	-1.02	-1.13	-1.01	-0.59	-1.08	-0.34	-0.29	-1.26	0.00	0.50	-1.00	Down	4.00
IGSF3	-0.14	0.13	-0.16	-0.41	-0.38	-0.21	-0.14	-0.29	0.14	-0.32	-0.29	-0.14	-0.50	-0.20	-0.09	-0.29	0.01	0.30	-1.75	Down	4.00
DCLRE1B	0.00	0.07	0.10	0.00	0.10	0.21	0.00	0.00	0.00	0.00	0.13	0.00	0.16	0.00	0.00	0.10	0.06	0.24	-2.05	Stable	4.00
AP4B1	-0.22	-0.32	-0.13	-0.36	-0.50	-0.53	-0.22	-0.32	-0.22	-0.32	0.10	-0.22	-0.48	-0.25	-0.22	-0.49	0.18	0.67	-0.57	Stable	4.00
PHTF1	0.08	-0.06	-0.01	-0.59	-0.51	-0.31	0.08	-0.33	0.01	-0.20	-0.12	0.08	-0.03	-0.20	0.08	-0.47	0.05	0.16	-2.66	Down	4.00
RHOC	0.00	0.01	0.01	0.01	0.03	0.01	0.00	0.00	0.00	0.01	0.03	0.00	0.01	0.01	0.00	0.03	0.02	0.30	-1.72	Down	4.00
ST7L	-0.38	0.00	-0.19	-0.19	-0.19	-0.21	-0.38	-0.23	-0.21	-0.26	-0.28	-0.38	-0.19	-0.30	-0.38	-0.19	0.66	1.10	0.14	Stable	4.00

WDR77	0.00	0.00	0.00	0.00	-0.01	-0.01	0.00	0.00	0.00	0.00	0.00	0.00	0.00	0.00	0.00	-0.01	0.07	0.25	-2.00	Stable	4.00
ALX3	-0.29	-0.15	-0.18	-0.21	-0.14	-0.22	-0.32	-0.26	-0.19	-0.26	-0.16	-0.22	-0.28	-0.25	-0.29	-0.14	0.23	1.18	0.24	Stable	4.00
GSTM3	-0.03	0.21	0.26	0.18	0.07	0.02	-0.04	-0.02	-0.01	-0.02	0.12	0.01	-0.05	-0.04	-0.04	0.07	0.80	0.74	-0.44	Stable	4.00
GNAT2	0.16	0.16	0.00	0.19	0.33	0.32	-0.28	0.32	-0.09	0.32	-0.30	0.12	0.43	0.16	0.00	0.33	0.15	0.25	-1.98	Stable	4.00
GNAI3	-0.39	-0.18	-0.16	0.20	0.49	0.37	-0.16	-0.19	-0.18	-0.19	-0.35	0.11	0.02	-0.16	-0.04	0.49	0.02	-1.24	0.31	Stable	4.00
CYB561D1	0.02	0.02	0.00	0.00	0.04	0.04	0.00	0.04	0.02	0.04	0.00	0.02	0.02	0.02	0.02	0.04	0.35	0.70	-0.51	Stable	4.00
SORT1	0.33	0.28	-0.17	0.05	0.49	0.74	-0.37	0.78	0.34	0.78	-0.03	0.02	0.49	0.24	0.19	0.49	0.32	0.54	-0.89	Stable	4.00
MYBPHL	-0.02	-0.04	-0.03	-0.04	-0.04	-0.04	-0.02	-0.04	-0.03	-0.04	-0.05	-0.04	-0.12	-0.04	-0.02	-0.04	0.06	0.59	-0.77	Stable	4.00
STXBP3	-0.55	-0.07	0.45	0.37	-0.50	-0.82	0.09	-1.14	-0.28	-1.14	0.87	-0.08	-0.39	-0.76	-0.37	-0.50	0.85	1.21	0.27	Stable	4.00
SLC25A24	0.36	1.07	1.62	1.73	0.42	0.19	0.94	0.08	0.58	0.08	1.27	0.45	1.39	0.11	0.19	0.33	0.71	0.85	-0.24	Stable	4.00
FAM102B	-0.71	-0.62	-0.50	-0.58	-0.64	-0.75	-0.80	-0.83	-0.46	-0.83	-0.60	-0.54	-0.84	-0.70	-0.84	-0.64	0.95	1.01	0.01	Stable	4.00
DPH5	0.10	0.15	0.11	-0.08	-0.08	0.31	0.15	0.25	0.18	0.35	0.14	0.14	-0.02	0.21	0.55	0.33	0.37	1.56	0.64	Stable	4.00
SLC30A7	-0.13	-0.08	0.01	-0.08	-0.19	-0.27	-0.05	-0.09	-0.21	-0.12	-0.08	-0.21	-0.11	-0.09	-0.33	-0.20	0.45	0.78	-0.37	Stable	4.00
RTCA	-0.80	-0.82	-0.54	-0.59	-0.86	-0.63	-0.25	-0.23	0.09	-0.40	-0.88	-0.09	-0.60	-0.39	-0.02	-0.26	0.29	0.69	-0.54	Stable	4.00
DBT	0.08	0.09	0.13	0.17	0.13	0.10	0.26	0.11	0.25	0.26	0.13	0.18	0.24	0.07	0.26	0.13	0.78	0.94	-0.09	Stable	4.00
MFSD14A	-0.50	-0.35	-0.37	-0.48	0.35	-0.07	-0.18	-0.39	-0.22	-0.16	-0.60	-0.48	-0.53	-0.46	-0.19	0.35	0.39	1.65	0.72	Stable	4.00
AGL	-0.44	-0.18	-0.08	-0.35	-0.32	-0.36	-0.45	-0.38	-0.10	-0.32	-0.21	-0.15	-0.32	-0.36	-0.21	-0.37	0.65	0.91	-0.14	Stable	4.00
SLC44A3	-0.09	-0.21	-0.10	-0.30	-0.24	-0.33	-0.69	-0.63	-0.84	-0.62	-0.13	0.07	-0.73	-0.93	-0.16	-0.62	0.56	1.26	0.33	Stable	4.00
RPL5	0.11	0.13	0.18	0.15	0.11	0.06	0.06	0.06	0.05	0.11	0.10	0.09	0.11	0.01	0.11	0.06	0.63	0.89	-0.17	Stable	4.00
RPAP2	0.47	0.42	0.59	0.42	0.19	0.09	0.12	0.18	0.09	0.35	0.27	0.34	0.35	0.08	0.35	0.25	0.94	1.02	0.03	Stable	4.00
GLMN	0.32	0.24	0.44	0.24	0.28	0.08	0.04	0.14	0.07	0.16	0.05	0.28	0.16	0.07	0.16	0.22	1.00	1.00	0.00	Stable	4.00
HFM1	-0.05	0.01	-0.85	-0.92	-0.13	-0.18	-0.88	0.84	0.01	-1.07	-1.19	-0.55	-1.07	0.30	-1.07	-0.35	0.12	0.31	-1.68	Stable	4.00
ENSSSCG00000006928	0.09	0.11	0.37	0.26	0.41	0.49	-0.08	0.18	0.14	-0.19	0.35	0.35	0.69	-0.11	-0.19	0.31	0.03	0.19	-2.38	Down	4.00
CLCA2	-0.14	0.52	0.53	-0.17	-0.17	0.17	-0.29	-0.22	-0.28	-0.29	-0.11	-0.12	-0.06	-0.21	-0.29	0.28	0.93	0.79	-0.33	Stable	4.00
DNAI3	0.87	0.38	0.30	0.22	0.14	0.27	-0.24	0.19	-0.24	-0.24	0.30	0.28	0.87	0.17	-0.24	0.24	0.54	0.58	-0.80	Stable	4.00
GSDMD	0.22	0.33	0.08	0.09	0.29	0.05	0.35	0.13	0.13	0.20	0.32	0.43	0.23	0.16	0.03	0.27	0.37	0.76	-0.39	Stable	4.00
RHPN1	0.79	0.88	1.07	1.32	0.21	0.59	0.86	0.73	0.14	1.04	0.15	0.97	1.04	0.72	1.03	0.61	0.84	1.05	0.07	Stable	4.00
ZDHHC2	0.12	0.01	-0.03	-0.08	0.14	-0.27	0.21	0.19	0.26	0.00	-0.08	-0.07	0.10	0.14	0.01	-0.07	0.02	-2.80	1.48	Up	17.00
SLC7A2	0.51	0.17	-0.14	-0.22	0.28	-0.06	0.40	0.38	0.51	0.44	-0.13	-0.11	-0.07	0.16	0.09	0.04	0.06	11.28	3.50	Stable	17.00
ENSSSCG00000007003	0.47	0.36	0.56	0.30	0.26	0.39	0.08	0.37	0.40	0.57	0.45	0.75	0.47	0.14	0.51	0.44	0.28	0.80	-0.33	Stable	17.00
POLB	-0.34	-0.46	-0.24	0.10	-0.44	0.01	-0.39	-0.07	-0.02	-0.11	0.19	-0.24	-0.41	-0.05	0.14	-0.16	0.68	1.35	0.43	Stable	17.00
ENSSSCG00000007047	-0.90	0.21	0.77	0.20	-0.21	-0.82	-0.29	0.29	-0.16	0.20	-0.81	-0.50	0.01	-0.82	-0.72	-0.31	0.77	0.73	-0.46	Stable	17.00
ANKEF1	0.35	0.11	0.00	0.31	0.25	0.03	0.25	0.31	0.07	0.21	0.22	0.43	0.32	0.29	0.01	0.25	0.23	0.68	-0.55	Stable	17.00
ENSSSCG00000007082	0.14	0.12	0.12	0.14	0.26	0.12	0.26	-0.02	0.00	0.28	0.19	0.15	0.06	0.08	0.12	0.26	0.08	0.57	-0.82	Stable	17.00
ENSSSCG00000007088	0.64	0.70	0.51	0.65	0.40	-0.09	0.92	0.11	0.00	0.11	0.22	0.25	0.22	0.52	0.19	0.49	0.25	1.59	0.66	Stable	17.00
MGME1	0.86	0.88	0.79	1.17	1.01	0.99	0.92	0.43	0.05	0.40	1.27	1.04	1.04	0.42	0.75	1.04	0.03	0.64	-0.64	Stable	17.00
KAT14	0.09	0.04	0.00	0.04	0.08	0.00	0.15	0.00	0.00	0.03	0.00	0.00	0.00	0.08	0.04	0.12	0.52	1.47	0.56	Stable	17.00
ZNF133	-2.24	-2.25	-2.27	-2.25	-2.28	-2.28	-2.24	-2.22	-2.10	-2.18	-2.27	-2.26	-2.28	-2.16	-0.78	-2.25	0.25	0.90	-0.15	Stable	17.00
DZANK1	0.22	0.29	0.21	0.38	0.44	0.18	0.44	0.07	-0.01	-0.01	0.40	0.22	0.14	0.30	0.25	0.44	0.50	0.81	-0.31	Stable	17.00
ENSSSCG00000007117	-0.42	-0.46	-0.33	-0.43	-0.48	-0.44	-0.37	-0.32	-0.43	-0.43	-0.46	-0.37	-0.37	-0.43	-0.57	-0.48	0.66	0.97	-0.05	Stable	17.00
ACSS1	-0.24	0.04	0.09	0.03	-0.25	-0.04	-0.22	0.12	-0.10	-0.10	-0.20	-0.24	-0.49	-0.10	0.02	-0.25	0.08	0.26	-1.95	Stable	17.00
NINL	0.08	0.16	0.23	0.10	0.24	0.12	0.24	0.21	0.25	0.10	0.22	0.22	0.22	0.25	0.07	0.24	0.91	1.02	0.03	Stable	17.00
ADAM33	0.04	-0.02	-0.31	-0.32	-0.64	0.10	-0.20	-0.99	-0.97	-0.35	-0.38	-0.56	-0.58	-1.00	0.18	-0.64	0.94	0.97	-0.05	Stable	17.00
CDC25B	0.42	0.22	0.47	1.07	0.87	0.35	0.94	0.72	1.21	0.49	0.51	0.88	0.96	1.35	0.02	0.87	0.67	0.89	-0.17	Stable	17.00
UBOX5	-0.18	-0.71	-0.83	-0.66	-0.88	-0.34	-0.76	-0.70	-0.56	-0.40	-0.48	-0.50	-0.47	-0.62	-0.31	-0.88	0.94	1.02	0.02	Stable	17.00

MRPS26	-0.01	-0.02	0.08	-0.15	0.67	0.28	0.08	-0.07	0.15	-0.14	0.32	0.46	-0.04	0.26	0.18	0.67	0.19	0.31	-1.69	Stable	17.00
VPS16	-0.07	0.16	0.05	0.06	-0.01	-0.13	0.03	0.17	0.08	0.04	0.03	0.01	0.05	0.03	-0.21	-0.01	0.61	6.49	2.70	Stable	17.00
SIRPB2	-0.10	0.06	-0.32	-0.59	-0.34	-0.19	-0.15	0.03	-0.13	-0.07	-0.12	-0.31	-0.16	-0.07	-0.38	-0.34	0.12	0.50	-0.99	Stable	17.00
ENSSSCG00000007188	-0.16	-0.38	-0.24	-0.15	-0.38	-0.19	-0.38	-0.38	-0.38	-0.35	-0.38	-0.16	-0.16	-0.38	-0.16	-0.38	0.47	1.15	0.21	Stable	17.00
SDCBP2	-0.07	-0.14	-0.07	-0.07	-0.14	-0.07	-0.14	-0.14	-0.14	-0.14	-0.14	-0.07	-0.07	-0.14	-0.07	-0.14	0.64	1.08	0.12	Stable	17.00
TPX2	0.01	0.11	0.25	0.23	0.21	0.08	0.12	0.48	0.45	0.11	0.12	0.09	0.21	0.41	0.13	0.21	0.22	1.57	0.65	Stable	17.00
CCM2L	0.03	0.07	0.16	0.13	0.13	0.06	0.05	0.28	0.28	0.16	0.10	0.03	0.07	0.41	0.10	0.13	0.18	1.74	0.80	Stable	17.00
POFUT1	0.03	-0.33	-0.16	0.00	0.00	0.00	0.03	-0.30	0.03	0.00	0.03	0.00	-0.23	0.03	0.00	0.00	0.36	3.36	1.75	Stable	17.00
KIF3B	0.06	0.11	0.20	0.03	0.18	-0.08	0.08	0.01	0.02	-0.13	0.23	0.13	-0.08	0.05	0.06	0.14	0.72	1.37	0.46	Stable	17.00
NOL4L	-0.01	-0.05	-0.13	-0.28	-0.31	-0.10	-0.11	-0.16	-0.31	-0.22	-0.18	-0.16	-0.18	-0.31	-0.17	-0.31	0.21	0.72	-0.48	Stable	17.00
ACSS2	0.00	0.20	0.22	0.05	0.22	0.13	0.11	0.36	0.30	0.11	0.15	0.03	0.13	0.37	0.08	0.22	0.19	1.57	0.65	Stable	17.00
RBL1	-0.02	-0.30	-0.14	-0.56	-0.32	-0.08	-0.16	-0.36	-0.27	-0.06	-0.29	-0.16	-0.17	-0.16	-0.27	-0.32	0.62	0.85	-0.23	Stable	17.00
RPRD1B	0.62	0.41	0.53	0.74	0.10	0.36	0.20	0.85	0.79	0.85	0.05	0.47	0.44	0.62	0.81	0.10	0.13	1.55	0.64	Stable	17.00
KIAA1755	-0.06	0.27	0.19	-0.02	0.27	0.14	0.22	0.10	-0.04	-0.02	0.27	0.10	-0.02	-0.06	0.19	0.27	0.76	0.83	-0.27	Stable	17.00
ENSSSCG00000007346	0.16	-1.22	-0.60	0.22	-1.09	-0.65	-0.04	-0.59	0.12	-0.02	-1.23	-0.19	0.49	0.08	-0.07	-1.09	0.56	0.61	-0.72	Stable	17.00
ACTR5	-0.23	-0.18	-0.16	0.14	-0.24	0.24	-0.02	-0.25	-0.23	-0.06	-0.24	-0.20	-0.19	-0.25	-0.02	-0.24	0.38	1.68	0.75	Stable	17.00
EMILIN3	0.26	0.19	0.30	0.07	0.28	0.38	0.22	0.35	0.30	0.13	0.11	0.27	0.09	0.28	0.19	0.26	0.19	1.31	0.39	Stable	17.00
LPIN3	0.65	0.23	0.18	0.24	0.73	0.74	0.08	0.54	0.28	0.49	0.40	0.66	0.45	0.32	-0.11	0.78	0.02	0.48	-1.05	Down	17.00
R3HDML	-0.17	-0.39	-0.42	-0.41	-0.03	-0.25	-0.03	-0.60	-0.14	-0.11	-0.19	0.31	-0.43	0.26	-0.07	-0.03	0.67	1.39	0.47	Stable	17.00
SNX21	0.00	0.07	0.05	0.03	0.05	0.01	0.00	0.17	0.23	0.28	0.05	0.14	0.03	0.05	0.03	0.05	0.90	0.93	-0.11	Stable	17.00
ACOT8	0.12	0.42	0.49	-0.20	-0.45	0.05	-0.06	0.80	0.06	-0.05	0.50	-0.12	-0.20	-0.17	0.61	-0.33	0.03	-2.82	1.50	Up	17.00
NEURL2	-0.10	-0.27	-0.80	-0.24	0.37	0.23	-0.52	-1.26	-0.53	0.22	0.30	-0.02	-0.03	-0.02	-0.39	0.37	0.00	-3.27	1.71	Up	17.00
CDH22	-0.41	-0.19	-0.59	-0.48	-0.20	-0.42	-0.43	-0.57	-0.04	-0.05	-0.54	-0.21	-0.06	-0.11	-0.62	-0.20	0.36	1.37	0.45	Stable	17.00
SLC2A10	0.05	-0.01	-0.01	-0.02	-0.04	-0.05	0.02	0.07	0.04	-0.02	-0.01	-0.02	-0.08	-0.02	-0.02	-0.07	0.00	-0.39	-1.36	Down	17.00
KCNB1	0.24	0.17	0.03	0.16	0.35	0.15	0.07	0.20	0.19	0.27	0.29	0.20	0.25	0.08	0.17	0.38	0.01	0.56	-0.84	Stable	17.00
PTGIS	0.34	-0.17	-0.08	-0.08	-0.35	-0.25	0.39	-0.10	-0.04	-0.26	0.01	-0.53	-0.19	-0.04	0.25	-0.33	0.01	-0.28	-1.86	Down	17.00
ENSSSCG00000007464	0.03	-0.11	-0.12	-0.25	-0.11	0.03	0.19	-0.10	0.02	-0.12	0.04	0.04	-0.21	-0.10	0.09	-0.12	0.23	0.17	-2.57	Stable	17.00
SLC9A8	1.04	0.61	0.50	-0.36	-0.09	1.10	0.57	0.67	0.28	-0.12	-0.13	-0.08	-0.26	0.13	0.31	-0.22	0.02	-26.74	4.74	Up	17.00
BCAS4	-0.05	0.06	0.06	0.08	0.00	0.18	0.14	-0.18	-0.07	0.02	0.15	0.05	-0.15	0.05	0.13	0.00	0.65	0.41	-1.29	Stable	17.00
DOK5	0.14	0.49	0.08	0.68	0.36	0.23	0.24	0.46	0.51	0.47	0.73	0.46	0.47	0.12	0.24	0.40	0.04	0.60	-0.74	Stable	17.00
FAM210B	0.00	-0.19	-0.06	0.29	0.53	-0.33	0.20	0.07	-0.26	0.33	0.47	0.53	0.31	0.21	0.00	0.37	0.02	-0.01	-6.56	Down	17.00
RAE1	-0.06	-0.40	-0.30	-0.25	-0.38	0.30	-0.53	-0.34	-0.46	0.23	0.13	-0.50	0.12	-0.62	-0.68	-0.42	0.03	4.42	2.14	Up	17.00
CTCFL	-0.62	-0.58	-0.47	-0.54	-0.70	-0.74	-0.62	-0.41	-0.70	-0.61	-0.76	-0.86	-0.64	-0.57	-0.91	-0.77	0.18	0.87	-0.20	Stable	17.00
VAPB	-0.03	-0.10	-0.22	0.03	-0.07	-0.16	0.05	-0.21	-0.14	-0.32	-0.19	-0.07	-0.22	0.08	-0.04	-0.07	0.31	0.56	-0.83	Stable	17.00
ENSSSCG00000007552	0.00	0.04	0.06	0.09	0.00	0.08	0.00	0.00	0.00	0.00	0.16	0.08	0.16	0.00	0.00	0.02	0.03	0.17	-2.59	Down	3.00
AMZ1	-0.18	0.10	-0.03	0.41	0.21	0.10	-0.18	-0.02	-0.18	-0.18	0.59	0.10	0.51	-0.18	-0.18	0.23	0.00	-0.43	-1.22	Down	3.00
CHST12	0.23	0.17	0.03	-0.01	0.19	0.16	0.21	0.14	0.23	0.18	0.15	0.23	0.15	0.23	0.23	0.16	0.39	1.21	0.27	Stable	3.00
GRIFIN	0.00	-0.04	0.00	-0.38	0.04	-0.23	-0.04	0.00	0.00	-0.08	-0.03	-0.19	-0.14	0.00	0.00	0.00	0.05	0.08	-3.56	Stable	3.00
AP5Z1	-0.22	0.07	-0.30	0.21	0.51	-0.01	-0.11	-0.21	-0.21	-0.16	0.68	-0.39	0.28	-0.69	-0.19	0.63	0.01	-1.06	0.09	Stable	3.00
PMS2	0.11	0.28	0.16	0.13	0.00	0.30	0.08	0.11	0.14	0.12	0.00	0.31	0.14	0.16	0.09	0.00	0.77	1.12	0.16	Stable	3.00
EIF2AK1	-0.13	-0.17	-0.10	-0.30	-0.32	-0.25	-0.09	-0.24	-0.29	-0.42	-0.37	-0.36	-0.31	-0.28	-0.42	-0.30	0.03	0.65	-0.62	Stable	3.00
KPNA7	0.13	-0.26	-0.07	0.06	-0.02	-0.13	0.21	0.02	0.18	0.15	0.02	0.05	0.01	0.19	0.18	-0.11	0.33	19.82	4.31	Stable	3.00
ZNF789	-0.55	0.47	-0.24	-0.03	0.32	-0.21	-0.21	-0.26	-0.36	-0.29	-0.12	-0.05	0.20	-0.35	-0.50	0.15	0.09	56.94	5.83	Stable	3.00
ENSSSCG00000007622	0.08	-0.25	-0.11	-0.07	-0.24	-0.18	-0.03	-0.16	0.10	0.06	-0.07	-0.13	-0.18	0.05	0.12	-0.19	0.12	0.22	-2.17	Stable	3.00
ARPC1B	0.92	0.45	0.37	0.72	0.11	0.33	0.30	1.29	1.76	1.58	-0.02	0.38	0.12	1.34	1.14	0.18	0.07	2.23	1.16	Stable	3.00

GJC3	-0.10	0.24	0.53	-0.06	0.43	0.63	0.12	0.23	0.09	0.34	0.14	0.18	0.40	0.15	0.22	0.25	0.30	0.64	-0.65	Stable	3.00
TRIM4	-0.13	0.09	0.11	-0.02	0.33	0.43	0.01	0.17	0.07	0.33	0.06	0.02	0.36	0.20	0.15	0.34	0.07	0.37	-1.45	Stable	3.00
ENSSSCG00000007642	-0.32	-0.03	0.63	-0.11	0.59	1.17	0.14	0.90	0.90	0.71	0.14	-0.02	0.63	0.65	0.71	0.07	0.83	1.12	0.17	Stable	3.00
ENSSSCG00000007644	-0.24	-0.03	-0.17	-0.01	0.05	0.09	-0.24	0.06	-0.17	0.02	-0.13	-0.08	0.04	-0.12	-0.18	0.02	0.01	-85.81	6.42	Up	3.00
ENSSSCG00000007665	0.00	-0.03	0.00	0.00	-0.13	0.00	0.00	0.00	0.00	0.00	0.00	-0.21	-0.19	0.00	0.00	-0.21	0.04	0.05	-4.47	Down	3.00
TFR2	-0.75	-0.86	-0.87	-0.44	-0.35	-1.06	-0.75	-0.80	-0.75	-0.95	-0.77	-0.80	-0.97	-0.82	-0.83	-0.53	0.48	1.10	0.13	Stable	3.00
POP7	-0.64	-0.48	-0.62	-0.65	-0.49	-0.63	-0.64	-0.29	-0.35	-0.52	-0.73	-0.40	-0.63	-0.55	-0.42	-0.48	0.28	0.88	-0.18	Stable	3.00
COL26A1	0.23	0.07	0.18	0.00	0.08	0.17	0.23	0.14	0.12	0.02	0.11	0.19	0.02	0.23	0.23	0.08	0.01	2.18	1.12	Up	3.00
HIP1	0.14	-0.14	0.05	0.05	0.04	-0.05	0.07	0.06	0.08	-0.03	0.05	0.00	0.10	-0.02	0.00	0.10	0.94	0.92	-0.12	Stable	3.00
TBL2	0.00	0.00	0.00	0.00	0.00	0.05	0.00	0.00	0.00	0.05	0.00	0.00	0.00	0.00	0.05	0.00	0.59	0.53	-0.91	Stable	3.00
BUD23	0.46	0.10	0.46	0.02	0.28	0.32	0.42	0.26	0.23	0.40	0.28	0.30	0.24	0.07	0.32	0.21	0.62	1.13	0.18	Stable	3.00
METTL27	-0.40	-0.33	-0.39	-0.01	-0.37	-0.27	-0.42	-0.07	-0.28	-0.38	-0.14	-0.37	-0.25	-0.29	-0.47	-0.37	0.35	1.23	0.30	Stable	3.00
PHKG1	0.10	0.01	0.07	-0.08	-0.17	0.01	0.14	-0.31	0.07	-0.18	-0.35	0.05	-0.19	0.15	-0.04	-0.01	0.07	-0.20	-2.36	Stable	3.00
ENSSSCG00000007749	-0.25	-0.12	-0.10	0.47	0.62	0.11	0.23	0.15	0.19	0.42	0.25	-0.30	0.41	-0.04	0.07	0.42	0.04	0.06	-4.01	Down	3.00
RUSF1	-0.09	0.01	0.30	0.13	-0.09	0.54	0.06	0.05	0.19	-0.01	0.03	-0.04	-0.01	0.29	-0.09	0.09	0.91	1.12	0.17	Stable	3.00
BCKDK	1.64	0.82	0.68	0.53	1.84	0.44	1.50	0.15	0.77	1.14	0.86	1.42	1.76	0.68	1.89	1.38	0.59	0.87	-0.20	Stable	3.00
RNF40	-0.08	-0.03	-0.09	-0.13	-0.07	-0.06	-0.19	-0.01	-0.09	-0.09	-0.01	-0.10	-0.08	-0.11	-0.03	-0.16	0.77	0.91	-0.14	Stable	3.00
ATP2A1	0.17	0.03	0.09	0.00	-0.09	0.28	0.21	0.14	0.06	0.28	0.17	0.23	-0.08	0.28	0.18	0.21	0.74	1.17	0.23	Stable	3.00
GTF3C1	-0.08	-0.05	-0.03	-0.07	0.03	-0.12	-0.04	-0.25	-0.14	-0.25	-0.31	-0.07	-0.01	-0.19	-0.11	-0.04	0.88	1.08	0.11	Stable	3.00
KDM8	0.05	0.18	-0.05	-0.26	-0.04	-0.25	-0.44	-0.15	-0.20	-0.50	-0.50	0.08	-0.05	-0.25	0.35	-0.44	0.16	0.27	-1.91	Stable	3.00
ENSSSCG00000007826	0.10	-0.15	-0.07	0.18	0.18	0.10	0.18	0.01	0.18	0.18	0.18	0.02	0.18	0.08	-0.14	0.18	0.04	0.16	-2.64	Down	3.00
SDR42E2	-0.02	0.79	0.73	0.08	0.06	0.13	0.17	0.00	0.44	0.22	0.08	0.17	0.14	0.13	-0.02	0.15	0.24	2.19	1.13	Stable	3.00
CRYM	0.41	0.16	0.23	0.16	0.35	0.31	0.16	0.42	0.16	0.28	0.32	0.26	0.11	0.14	0.29	0.02	0.76	1.08	0.11	Stable	3.00
ANKS4B	0.23	0.04	0.01	0.09	0.23	0.23	0.11	0.23	0.11	0.23	0.23	0.08	0.11	0.12	0.23	0.12	0.47	0.82	-0.28	Stable	3.00
ENSSSCG00000007858	0.01	-0.03	0.04	0.01	0.12	0.04	0.08	0.14	0.02	0.07	0.01	0.08	0.01	0.11	0.17	0.02	0.43	1.50	0.59	Stable	3.00
ABCC1	-0.92	-0.31	-0.21	-1.24	-0.47	-1.19	-1.61	-0.68	-1.15	-0.49	-0.87	-1.27	-1.42	-0.27	-0.62	-0.94	0.24	0.73	-0.45	Stable	3.00
ENSSSCG00000007899	-0.24	-0.07	-0.30	-0.21	0.01	0.12	0.31	-0.20	-0.44	0.16	-0.36	0.11	-0.05	-0.11	-0.17	0.32	0.15	-12.03	3.59	Stable	3.00
METTL22	-0.59	-0.73	-0.21	-0.68	-0.21	-0.21	-0.20	-0.06	-0.89	-0.16	-0.21	-0.59	-0.21	-0.85	-1.07	-0.20	0.11	1.86	0.89	Stable	3.00
ENSSSCG00000007920	0.12	0.50	0.54	0.10	0.11	0.11	0.11	0.16	0.18	0.11	0.11	0.07	0.11	0.15	0.15	0.11	0.06	2.38	1.25	Stable	3.00
ENSSSCG00000007944	-0.11	-0.04	-0.09	0.00	0.00	-0.05	-0.05	-0.15	0.05	-0.05	-0.05	0.00	0.00	-0.05	0.02	-0.05	0.36	1.90	0.93	Stable	3.00
TRAP1	-0.17	-0.40	-0.20	-0.19	-0.20	-0.20	-0.20	0.04	-0.60	-0.20	-0.20	-0.17	-0.20	-0.04	-0.29	-0.20	0.62	1.19	0.25	Stable	3.00
CIAO3	-0.23	-0.41	-0.23	-0.01	0.34	0.11	0.49	0.18	0.30	0.49	0.43	-0.34	0.34	-0.25	-0.18	0.49	0.10	-0.18	-2.49	Stable	3.00
LMF1	-0.02	0.00	-0.02	0.00	-0.02	-0.03	-0.03	0.00	-0.02	-0.02	-0.03	-0.02	-0.03	0.00	0.00	-0.03	0.06	0.45	-1.14	Stable	3.00
BAIAP3	-0.14	0.26	-0.03	-0.90	-0.28	-0.03	0.00	0.19	-0.13	-0.09	-0.15	-0.32	-0.27	-0.34	-0.30	0.24	0.25	0.27	-1.91	Stable	3.00
NOL8	-0.20	-0.01	-0.20	-0.07	-0.15	-0.20	-0.20	-0.07	0.01	-0.20	-0.18	-0.10	-0.15	0.01	0.08	-0.20	0.08	0.47	-1.10	Stable	3.00
CHCHD5	0.57	0.24	0.31	0.15	-0.10	0.65	0.87	0.05	-0.23	0.57	0.29	0.34	-0.10	-0.06	0.00	0.87	0.52	0.65	-0.63	Stable	3.00
ENSSSCG00000008097	0.00	0.00	-0.42	-0.14	-1.07	0.00	0.00	-0.24	-0.40	-0.43	-0.50	0.00	-1.07	0.00	0.00	0.00	0.16	0.33	-1.60	Stable	3.00
CIAO1	0.25	0.22	0.15	0.29	0.03	0.05	0.21	0.12	0.21	0.15	0.16	0.15	0.03	0.13	0.27	0.28	0.27	1.33	0.41	Stable	3.00
ENSSSCG00000008137	0.10	0.22	0.31	-0.03	0.24	-0.14	0.87	0.20	0.97	0.20	0.67	-0.03	0.37	-0.57	-0.10	0.87	0.93	0.93	-0.11	Stable	3.00
ST6GAL2	-0.75	0.00	-0.64	-0.30	-0.31	-0.48	-0.89	-0.27	-0.55	-0.70	-0.81	-0.62	-0.62	-0.57	-0.14	-0.54	0.59	0.87	-0.20	Stable	3.00
ENSSSCG00000008170	-0.10	-0.20	-0.19	-0.20	-0.20	-0.20	-0.20	-0.20	-0.05	-0.20	-0.20	-0.10	-0.20	0.07	-0.03	-0.20	0.08	0.59	-0.75	Stable	3.00
CHST10	-1.36	-0.58	-0.78	-0.77	-0.82	-0.68	-0.41	-0.54	-0.03	-1.25	-1.50	-0.02	-0.48	-0.63	-0.16	-0.49	0.40	0.75	-0.42	Stable	3.00
ENSSSCG00000008183	-0.14	-0.48	-0.59	0.00	0.00	0.00	0.00	-0.30	0.00	0.00	0.00	0.00	0.00	-0.07	0.00	0.00	0.05	#NAME?	Inf	Up	3.00
MGAT4A	0.10	0.16	0.16	0.26	0.13	0.26	0.16	0.16	0.26	0.16	0.26	0.06	0.13	0.20	0.23	0.16	0.94	1.01	0.02	Stable	3.00
ELMOD3	0.03	0.21	0.14	0.15	0.19	0.17	0.17	0.28	0.10	0.17	0.30	0.05	0.21	-0.18	0.07	0.17	0.21	0.59	-0.77	Stable	3.00

ENSSSCG00000008240	0.37	0.46	0.31	0.06	0.47	-0.13	0.31	0.39	0.25	0.31	0.21	0.44	0.33	0.26	0.28	0.31	0.32	1.32	0.40	Stable	3.00
CCDC142	0.36	0.29	0.44	0.34	0.18	0.29	0.27	0.34	-0.02	0.10	0.16	0.11	0.13	0.36	0.06	0.46	0.57	1.19	0.25	Stable	3.00
SFXN5	0.14	-0.20	-0.07	0.01	-0.16	-0.09	0.14	-0.06	0.06	0.07	0.03	-0.11	0.02	0.14	-0.08	0.07	0.64	-0.34	-1.56	Stable	3.00
ATP6V1B1	0.02	1.07	0.61	1.23	1.29	1.38	0.02	0.79	0.28	0.58	0.63	0.73	1.23	0.02	0.42	0.49	0.01	0.43	-1.23	Down	3.00
NAGK	-0.22	-0.52	-0.48	-0.58	-0.60	-0.59	-0.22	-0.42	-0.45	-0.31	-0.60	-0.43	-0.54	-0.22	-0.23	-0.33	0.04	0.70	-0.52	Stable	3.00
AAK1	0.00	0.30	0.23	-0.08	-0.07	-0.03	0.00	-0.03	0.00	-0.04	-0.08	0.00	-0.08	0.00	-0.03	-0.04	0.05	-1.13	0.18	Stable	3.00
ARHGAP25	0.00	-0.24	0.00	0.14	-0.12	-0.07	0.00	0.02	-0.06	0.02	0.61	0.01	-0.05	0.00	-0.43	0.05	0.13	-1.20	0.27	Stable	3.00
APLF	0.00	0.02	-0.35	-0.10	0.20	0.03	0.00	0.00	0.13	0.08	0.25	0.18	0.08	0.00	0.18	0.00	0.20	-0.04	-4.65	Stable	3.00
FBXO48	0.01	-0.30	-0.18	-0.27	-0.22	-0.31	-0.15	-0.29	-0.21	-0.19	-0.29	-0.21	-0.29	0.01	-0.16	-0.23	0.06	0.63	-0.67	Stable	3.00
PNO1	0.16	0.31	0.49	0.18	0.17	0.17	0.08	0.31	0.08	0.09	0.17	0.09	0.18	0.16	0.24	0.09	0.13	1.61	0.69	Stable	3.00
WDR92	0.00	-0.30	-0.32	0.00	0.00	-0.24	0.00	-0.11	-0.20	0.00	0.00	-0.31	0.00	0.00	-0.47	0.00	0.19	2.56	1.36	Stable	3.00
KIAA1841	-0.28	0.23	-0.13	0.23	0.24	0.23	0.16	0.23	0.42	0.61	0.23	0.41	0.24	-0.05	-0.01	0.42	0.02	0.22	-2.21	Down	3.00
CFAP36	0.00	0.08	-0.08	0.18	0.04	0.18	-0.13	-0.14	0.04	-0.04	0.04	0.09	0.04	-0.07	0.08	-0.12	0.12	-0.56	-0.83	Stable	3.00
CLHC1	0.00	-0.12	-0.15	-0.08	-0.30	-0.05	0.18	-0.30	-0.20	0.07	-0.20	0.30	-0.17	-0.15	0.00	0.15	0.53	2.62	1.39	Stable	3.00
ERLEC1	-0.44	-0.14	-0.44	0.17	0.37	0.17	-0.44	-0.44	-0.04	0.06	0.17	0.05	0.26	-0.44	0.09	-0.23	0.00	-2.31	1.21	Up	3.00
SLC3A1	-1.31	-0.55	-0.45	-1.58	-2.00	-1.60	-0.84	-0.63	-0.43	-0.18	-1.22	-1.23	-1.38	-1.13	-0.62	-0.85	0.04	0.59	-0.75	Stable	3.00
ENSSSCG00000008451	-0.26	-0.34	-0.68	-0.17	-0.17	-0.24	-0.15	-0.07	-0.10	-0.30	-0.44	-0.10	-0.29	-0.34	-0.21	-0.26	0.77	1.09	0.13	Stable	3.00
DHX57	-0.47	-0.36	-0.10	-0.41	-0.31	-0.51	-0.34	-0.24	0.07	-0.50	-0.40	-0.37	-0.41	-0.16	-0.28	-0.47	0.02	0.55	-0.86	Stable	3.00
MORN2	0.03	0.06	0.11	-0.42	-0.42	-0.41	-0.34	-0.26	-0.27	0.03	-0.27	-0.36	-0.42	0.21	-0.52	-0.13	0.13	0.41	-1.29	Stable	3.00
GEMIN6	0.26	0.24	0.31	0.13	0.19	-0.22	-0.15	0.24	-0.35	0.28	-0.21	-0.10	0.13	0.31	-0.34	0.12	0.84	1.65	0.72	Stable	3.00
GALM	-0.23	-0.07	-0.06	-0.30	-0.28	-0.46	-0.34	0.21	0.32	-0.23	-0.62	-0.31	-0.47	-0.05	-0.29	-0.52	0.00	0.16	-2.65	Down	3.00
FEZ2	0.05	-0.07	0.05	-0.19	-0.18	0.00	0.04	-0.07	-0.20	0.05	-0.19	0.04	-0.17	0.10	-0.11	0.03	0.37	0.34	-1.56	Stable	3.00
DPY30	0.00	0.15	0.46	0.34	0.07	0.42	0.13	0.23	-0.57	-0.57	0.37	0.16	0.46	0.34	0.09	0.10	0.69	0.62	-0.69	Stable	3.00
SLC4A1AP	0.13	0.01	0.02	0.14	0.17	-0.32	-0.07	0.29	0.47	0.24	0.23	-0.08	0.26	0.07	0.03	-0.01	0.68	1.50	0.59	Stable	3.00
SLC5A6	-1.08	-1.26	-1.41	-0.48	-0.59	-0.70	-0.97	-1.80	-1.18	-1.17	-1.21	-1.09	-0.57	-1.35	-0.56	-0.65	0.03	1.49	0.57	Stable	3.00
PREB	-0.05	-0.11	-0.06	-0.08	-0.08	-0.06	-0.01	0.00	-0.04	-0.05	-0.04	-0.01	-0.08	-0.03	-0.10	-0.05	0.71	0.88	-0.18	Stable	3.00
ENSSSCG00000008555	-0.35	-0.11	-0.20	-0.03	-0.02	0.10	-0.41	-0.31	-0.35	-0.38	-0.17	-0.41	-0.02	-0.28	-0.01	-0.31	0.26	1.64	0.71	Stable	3.00
DPYSL5	0.05	0.09	-0.08	-0.11	-0.17	-0.04	0.03	-0.08	0.17	0.11	-0.18	0.05	-0.22	-0.03	0.06	-0.02	0.08	-0.34	-1.54	Stable	3.00
SLC35F6	-0.02	-0.26	0.08	-0.21	-0.15	-0.03	0.11	-0.07	-0.04	-0.06	0.23	-0.01	-0.10	-0.25	-0.23	-0.31	0.94	1.07	0.10	Stable	3.00
DRC1	0.28	-0.03	0.11	0.29	0.32	0.34	0.13	0.12	-0.03	0.03	0.11	0.13	0.28	-0.05	0.09	0.10	0.05	0.38	-1.38	Stable	3.00
HADHA	0.70	0.06	0.27	0.36	0.44	1.08	0.44	0.28	0.19	0.66	0.04	0.45	0.65	0.17	0.62	0.61	0.17	0.64	-0.65	Stable	3.00
GAREM2	-0.02	0.02	-0.18	-0.20	-0.20	-0.20	-0.13	0.05	0.14	-0.10	-0.08	-0.05	-0.20	-0.05	0.00	-0.04	0.02	0.15	-2.70	Down	3.00
KIF3C	0.47	0.22	0.67	0.16	0.16	0.20	0.13	0.87	0.06	0.35	0.16	0.18	0.13	0.02	0.39	0.32	0.23	1.70	0.77	Stable	3.00
TP53I3	0.00	0.00	0.00	-0.16	-0.16	-0.08	0.06	0.00	0.00	0.00	0.00	0.08	-0.14	0.00	0.00	0.00	0.09	-0.12	-3.11	Stable	3.00
SF3B6	-0.01	0.04	-0.01	0.10	0.09	0.11	-0.01	0.05	-0.01	0.04	-0.01	0.00	0.10	-0.01	0.00	0.04	0.02	0.10	-3.38	Down	3.00
ENSSSCG00000008599	-0.27	-0.42	-0.24	-0.26	-0.33	-0.59	-0.22	-0.36	-0.04	-0.25	0.27	-0.71	-0.17	-0.31	-0.20	-0.86	0.45	0.71	-0.49	Stable	3.00
WDR35	0.35	-0.60	0.05	-0.06	-0.02	-0.12	-0.05	0.07	-0.22	0.29	0.73	-0.24	0.27	-0.21	-0.06	-0.37	0.38	-1.38	0.47	Stable	3.00
NBAS	-0.01	0.02	0.03	0.00	0.00	0.06	-0.01	0.09	0.01	0.02	0.00	0.00	0.00	0.00	0.01	0.22	0.50	0.46	-1.12	Stable	3.00
TAF1B	0.19	-0.12	0.15	0.23	0.28	0.03	0.21	0.15	0.09	0.15	0.03	0.08	0.09	0.17	-0.16	0.10	0.55	0.70	-0.51	Stable	3.00
IAH1	0.90	0.22	-0.03	-1.46	1.16	0.13	0.04	-0.03	0.56	0.41	-1.77	-0.74	0.11	0.69	0.68	0.47	0.16	-1.80	0.85	Stable	3.00
RNF144A	-0.67	-1.08	-1.16	-0.53	-1.00	-1.00	-1.14	-1.03	-0.42	-0.84	-1.01	-0.53	-1.14	-1.30	-0.78	-0.84	0.53	1.10	0.14	Stable	3.00
CMPK2	0.03	0.74	0.84	1.45	1.26	0.24	0.43	0.40	1.08	0.22	0.89	1.34	0.43	0.30	0.62	0.48	0.30	0.70	-0.51	Stable	3.00
POLN	0.40	0.14	-0.04	0.09	0.43	0.07	0.53	0.38	0.39	0.40	0.19	0.25	0.17	0.37	0.24	0.19	0.35	1.34	0.42	Stable	8.00
ENSSSCG00000008686	-0.96	-0.40	-0.40	-0.67	-0.79	-0.81	-1.03	-0.36	-0.79	-0.62	-0.74	-0.88	-0.85	-0.62	-0.57	-0.88	0.20	0.82	-0.28	Stable	8.00
ZFYVE28	0.00	0.13	0.09	0.06	0.11	0.12	0.22	0.00	0.10	0.12	0.04	0.02	0.06	0.02	0.15	0.09	0.72	1.15	0.20	Stable	8.00

RNF4	0.55	0.50	-0.10	-0.15	0.18	-0.01	0.47	0.27	0.37	0.31	-0.19	-0.01	-0.13	0.21	-0.28	0.18	0.09	11.45	3.52	Stable	8.00
GRK4	-0.26	-0.59	-0.63	0.61	0.13	-0.34	-0.34	-0.87	-0.73	-0.64	-0.10	0.46	0.37	-0.14	-0.77	0.04	0.00	-8.10	3.02	Up	8.00
LRPAP1	-0.01	-0.07	0.02	-0.16	-0.14	-0.03	0.04	-0.02	-0.06	-0.14	-0.38	-0.57	-0.03	-0.26	-0.37	-0.41	0.12	0.39	-1.37	Stable	8.00
DOK7	-0.29	-0.19	0.20	-0.11	-0.42	0.06	-0.63	-0.02	-0.13	-0.09	-0.29	-0.23	-0.30	-0.18	-0.18	-0.22	0.82	0.88	-0.18	Stable	8.00
ABLIM2	0.04	0.02	-0.08	0.00	0.02	-0.10	0.04	0.04	-0.02	-0.06	-0.11	-0.06	-0.02	0.00	-0.04	-0.11	0.04	0.02	-5.77	Down	8.00
ACOX3	-0.01	0.02	-0.03	-0.02	0.02	0.15	0.04	-0.11	-0.14	-0.03	0.23	0.18	-0.03	-0.12	0.01	0.22	0.02	-0.49	-1.03	Down	8.00
FBXL5	0.39	-0.06	-0.14	0.35	0.09	0.20	0.33	-0.20	0.10	0.22	0.01	-0.08	0.29	-0.24	-0.02	0.49	0.12	0.11	-3.15	Stable	8.00
PROM1	0.09	-0.19	0.11	-0.03	-0.01	-0.01	-0.01	-0.15	-0.01	-0.01	-0.04	0.00	0.11	0.00	0.05	-0.06	0.88	2.00	1.00	Stable	8.00
PACRGL	0.00	-0.22	-0.22	0.00	0.00	0.00	0.00	0.00	0.00	0.00	0.00	0.00	0.00	0.00	0.00	0.00	0.17	#NAME?	Inf	Stable	8.00
RELL1	-0.34	-0.17	0.00	0.00	0.00	0.00	-0.17	-0.34	-0.17	-0.34	-0.17	-0.17	-0.17	-0.34	-0.17	0.00	0.11	2.00	1.00	Stable	8.00
WDR19	-0.18	-0.27	-0.49	0.31	-0.13	-0.22	-0.13	-0.30	-0.51	-0.27	-0.28	-0.31	-0.18	-0.18	-0.27	-0.23	0.17	1.78	0.83	Stable	8.00
RBM47	-0.51	-0.27	0.07	-0.03	0.07	-0.23	-0.18	-0.22	-0.27	-0.21	-0.27	-0.14	-0.51	-0.51	-0.14	-0.09	0.41	1.44	0.53	Stable	8.00
LIMCH1	0.00	0.00	0.10	0.00	-0.06	-0.02	0.00	0.00	0.00	0.00	-0.03	-0.03	0.00	0.00	-0.03	0.00	0.13	-0.50	-1.01	Stable	8.00
GNPDA2	0.00	0.00	0.00	0.00	0.36	0.00	0.00	0.00	0.00	0.00	0.00	0.00	0.00	0.00	0.00	0.00	0.35	0.00	#NAME?	Stable	8.00
KIT	0.18	0.25	0.60	0.30	0.31	-0.22	0.18	-0.56	-0.19	0.16	0.26	-0.15	0.18	-0.19	0.11	-0.19	0.83	0.59	-0.77	Stable	8.00
ENSSSCG00000008845	0.56	0.48	0.67	0.75	0.15	-0.01	0.56	-0.44	0.09	0.64	0.32	0.20	-0.07	0.03	0.59	-0.38	0.55	1.57	0.65	Stable	8.00
GUCY1A1	0.10	-0.12	-0.37	-0.50	-0.24	-0.51	0.10	-0.51	-0.19	-0.50	-0.51	-0.50	-0.51	-0.22	-0.50	-0.51	0.02	0.45	-1.15	Down	8.00
TMEM144	-0.18	-0.49	-1.07	-0.56	-0.19	-0.28	-0.18	-0.58	-0.42	-1.04	-0.44	-0.64	-0.40	-0.20	-0.65	-0.28	0.96	0.99	-0.02	Stable	8.00
ARL9	-0.19	0.01	-0.21	0.09	-0.11	-0.02	-0.19	0.20	-0.02	0.24	0.16	0.16	0.20	-0.05	0.20	-0.17	0.23	-0.43	-1.22	Stable	8.00
GRSF1	0.00	0.05	0.10	0.10	0.10	0.10	0.00	0.10	0.05	0.10	0.10	0.10	0.10	0.05	0.10	0.10	0.02	0.56	-0.83	Stable	8.00
NAAA	0.03	0.17	0.07	-0.16	0.16	-0.10	0.07	0.21	0.06	-0.22	-0.10	-0.21	-0.20	0.06	-0.20	-0.10	0.01	-0.48	-1.06	Down	8.00
CCDC158	0.12	0.20	-0.02	0.06	0.31	0.67	0.12	0.23	-0.01	0.20	0.82	0.35	0.70	0.16	0.30	0.78	0.01	0.28	-1.83	Down	8.00
ENSSSCG00000008990	-0.41	-0.13	-0.18	-0.23	-0.13	-0.27	-0.23	-0.19	-0.13	-0.47	-0.27	-0.29	-0.27	-0.08	-0.37	-0.27	0.28	0.78	-0.35	Stable	8.00
FRAS1	0.00	0.00	0.00	0.00	0.00	0.06	0.00	0.13	0.06	0.06	0.06	0.06	0.13	0.06	0.06	0.06	0.48	0.71	-0.49	Stable	8.00
RBM46	0.32	0.62	0.52	0.63	0.46	0.43	0.50	0.90	0.57	0.76	0.65	0.90	0.90	0.44	0.99	0.51	0.67	0.93	-0.10	Stable	8.00
TMEM131L	0.80	1.01	0.38	0.27	1.36	0.62	0.60	1.09	0.75	0.57	0.93	0.68	1.09	0.82	0.69	1.20	0.64	0.91	-0.13	Stable	8.00
GATB	-0.30	-0.17	0.06	0.10	-0.43	-0.60	-0.36	-0.43	-0.24	0.06	-0.51	-0.15	-0.43	-0.32	-0.15	-0.51	0.55	0.78	-0.37	Stable	8.00
INPP4B	0.37	0.34	0.17	0.38	0.34	0.32	0.34	0.26	0.34	0.23	0.30	0.30	0.32	0.31	0.27	0.32	0.64	0.96	-0.06	Stable	8.00
RNF150	0.43	0.00	-0.22	0.08	-0.11	-0.42	-0.20	-0.49	-0.26	-0.02	-0.32	0.01	-0.23	0.08	-0.30	-0.20	0.78	0.78	-0.35	Stable	8.00
C4orf33	-1.06	-0.63	-0.23	-0.55	-0.62	-1.06	-1.06	-0.64	-1.06	-0.61	-0.26	-0.61	-0.26	-1.06	-0.26	-0.55	0.26	1.33	0.41	Stable	8.00
ENSSSCG00000009072	-0.23	-0.28	0.20	-0.18	-0.16	-0.23	-0.23	-0.32	-0.23	-0.12	-0.35	-0.23	-0.35	-0.23	0.00	-0.47	0.23	0.64	-0.65	Stable	8.00
INTU	0.02	-0.26	0.45	-0.30	-0.24	0.02	0.02	-0.19	0.02	-0.16	-0.48	-0.27	-0.48	0.02	-0.48	-0.23	0.08	0.18	-2.46	Stable	8.00
ANXA5	0.73	0.45	0.67	0.42	0.61	0.40	0.58	0.30	0.55	0.18	0.17	0.45	0.30	0.49	0.35	0.30	0.05	1.45	0.54	Stable	8.00
METTL14	-0.46	-0.29	-0.52	-0.40	-0.26	-0.06	-0.28	-0.06	-0.26	-0.23	-0.15	-0.26	-0.11	-0.46	-0.26	-0.08	0.07	1.67	0.74	Stable	8.00
ENSSSCG00000009138	-0.21	-0.14	-0.15	-0.48	-0.14	-0.14	0.06	0.13	0.19	0.25	-0.14	-0.35	0.01	0.22	-0.25	0.10	0.39	0.16	-2.61	Stable	8.00
CASP6	-0.14	0.08	-0.04	-0.02	0.08	0.08	0.07	0.07	0.06	-0.06	0.08	-0.02	0.01	0.15	0.04	0.09	0.84	1.28	0.36	Stable	8.00
ETNPPL	0.76	0.45	0.20	0.99	0.45	0.45	0.14	0.35	0.42	0.20	0.45	0.51	0.22	0.13	0.55	0.14	0.68	0.88	-0.18	Stable	8.00
HADH	-0.95	-0.45	-0.24	-0.52	-0.45	-0.45	0.04	-0.04	-0.26	-0.17	-0.45	-0.45	-0.13	-0.44	-0.19	-0.01	0.93	0.96	-0.05	Stable	8.00
CYP2U1	0.30	0.10	0.21	0.08	0.10	0.10	0.05	0.24	0.22	0.11	0.10	0.10	0.05	0.05	0.36	0.08	0.04	2.08	1.05	Up	8.00
SLC9B1	0.15	0.20	0.17	0.21	0.33	0.23	0.20	0.23	0.23	0.18	0.26	0.23	0.33	0.09	0.23	0.32	0.02	0.73	-0.46	Stable	8.00
TRMT10A	-0.10	0.05	0.18	-0.20	0.05	-0.30	-0.20	-0.05	-0.10	0.08	0.10	-0.10	-0.05	0.18	-0.05	0.05	0.63	0.25	-1.99	Stable	8.00
ENSSSCG00000009182	-0.05	-0.17	0.34	0.38	0.32	0.70	0.32	-0.09	0.25	1.04	-0.09	0.20	0.34	0.15	0.85	0.39	0.23	0.49	-1.02	Stable	8.00
ENSSSCG00000009208	-0.17	-0.23	-0.05	-0.14	-0.13	-0.16	-0.17	-0.23	-0.12	-0.13	-0.21	-0.21	-0.13	-0.05	-0.13	-0.21	0.50	0.88	-0.18	Stable	8.00
ENSSSCG00000009211	-0.84	-0.89	-0.01	-0.69	-0.38	-0.79	-0.89	-0.96	-0.39	-0.39	-0.65	-0.66	-0.37	-0.03	-0.39	-0.63	0.90	0.97	-0.05	Stable	8.00
KLHL8	0.89	0.66	-0.18	0.76	0.54	0.47	0.43	0.73	0.84	0.76	0.76	0.80	0.76	0.47	0.76	0.53	0.48	0.86	-0.22	Stable	8.00

CDS1	0.23	0.34	0.30	0.51	0.37	0.02	0.23	0.34	0.48	0.23	0.27	0.30	0.51	0.38	0.22	0.37	0.90	0.97	-0.04	Stable	8.00
GPAT3	0.22	0.45	0.37	0.23	0.21	-0.05	0.22	0.44	0.04	0.13	-0.03	-0.06	0.20	0.26	0.20	-0.09	0.01	4.11	2.04	Up	8.00
ENSSSCG00000009240	0.91	0.23	0.39	-0.43	0.27	-0.25	0.91	0.41	0.20	0.21	-0.35	0.69	-0.25	0.41	0.32	-0.10	0.01	-18.57	4.21	Up	8.00
ANTXR2	0.38	0.75	0.11	0.09	0.24	0.52	0.38	1.00	0.38	0.19	0.57	0.00	0.05	0.19	0.14	0.21	0.19	1.79	0.84	Stable	8.00
ENSSSCG00000009281	-0.45	-0.62	-0.28	-0.61	-0.52	-0.54	-0.48	-0.26	-0.76	-0.39	-0.37	-0.49	-0.43	-0.16	-0.22	-0.27	0.57	0.89	-0.17	Stable	11.00
GTF3A	0.46	0.59	0.19	0.41	0.48	0.04	0.24	0.18	0.58	0.35	0.37	0.66	0.44	0.04	0.39	0.40	0.53	0.85	-0.24	Stable	11.00
LNX2	0.25	0.16	1.00	0.12	0.39	0.39	0.31	1.37	1.20	0.78	0.62	0.91	0.23	1.55	0.98	-0.05	0.08	2.01	1.01	Stable	11.00
POLR1D	0.61	-0.23	0.29	-0.09	0.50	-0.17	0.63	-0.20	-0.34	0.73	-1.48	0.85	-0.23	0.44	1.08	0.72	0.59	2.73	1.45	Stable	11.00
SLC46A3	0.00	-0.09	0.00	0.00	0.00	-0.09	0.00	0.00	0.00	0.00	0.00	0.00	0.00	0.00	0.00	0.00	1.00	1.00	0.00	Stable	11.00
RXFP2	-0.53	-0.21	-0.53	-0.53	-0.53	-0.33	-0.53	-0.53	-0.60	-0.53	-0.29	-0.53	-0.53	-0.27	-0.06	-0.53	0.40	0.85	-0.23	Stable	11.00
FRY	0.15	-0.10	-0.15	-0.15	0.01	0.14	0.02	-0.14	0.07	0.02	0.13	0.05	-0.06	-0.11	0.06	0.06	0.35	-1.01	0.02	Stable	11.00
ENSSSCG00000009348	0.19	-0.46	-0.78	-0.78	-0.79	-0.07	0.38	-0.73	1.25	0.36	-0.42	0.52	-0.64	-0.90	-0.32	-0.71	0.66	0.54	-0.89	Stable	11.00
RFC3	0.35	0.47	0.32	0.32	0.41	0.62	0.54	0.32	0.91	0.41	0.36	0.54	0.41	0.41	0.33	0.50	0.91	1.02	0.03	Stable	11.00
ALG5	-0.07	-0.07	-0.07	-0.07	-0.07	0.05	-0.07	-0.07	-0.07	-0.07	0.05	-0.07	-0.07	-0.07	-0.07	-0.07	0.17	1.80	0.85	Stable	11.00
TRPC4	0.25	0.44	0.38	0.30	0.35	-0.04	0.25	0.37	0.42	0.35	0.05	0.27	0.08	0.21	0.37	0.25	0.05	1.68	0.75	Stable	11.00
THSD1	0.65	0.45	0.52	0.08	0.58	0.18	0.81	0.34	0.39	0.84	0.00	0.31	0.03	0.17	0.59	0.14	0.11	1.81	0.86	Stable	11.00
NEK3	-0.60	-0.12	0.17	0.82	-0.03	0.41	-0.48	0.21	0.30	-0.19	0.69	-0.15	0.14	0.07	-0.03	0.50	0.09	-0.21	-2.22	Stable	11.00
SPRYD7	-0.61	-0.72	-0.73	-0.54	-0.49	-0.27	-0.61	-0.75	-0.35	-0.69	-0.27	-0.36	-0.54	-0.69	-0.33	-0.10	0.05	1.47	0.55	Stable	11.00
EBPL	-0.16	0.02	-0.07	0.20	0.02	0.19	-0.16	0.02	0.38	-0.07	0.20	0.20	0.21	-0.16	0.38	0.11	0.28	0.25	-2.00	Stable	11.00
SETDB2	-0.29	-0.27	-0.26	-0.14	-0.25	-0.06	-0.29	-0.27	0.09	-0.27	-0.03	-0.01	-0.10	-0.27	0.09	0.08	0.26	1.87	0.90	Stable	11.00
SUCLA2	-0.75	-0.87	-0.68	-0.86	-0.87	-0.51	-0.75	-0.87	-0.72	-0.79	-0.45	-0.75	-0.62	-0.71	-0.39	-0.78	0.87	1.02	0.03	Stable	11.00
ESD	0.63	1.00	1.00	-0.42	1.00	-0.05	0.54	1.00	0.02	1.12	-0.61	0.03	0.80	0.68	-0.37	-0.54	0.22	3.38	1.76	Stable	11.00
AKAP11	0.14	0.04	-0.15	0.39	0.51	0.65	0.14	0.08	0.65	-0.15	0.75	0.05	0.61	0.80	1.04	0.29	0.83	0.89	-0.16	Stable	11.00
DGKD	0.29	0.10	0.22	0.11	0.23	-0.08	0.22	0.22	0.22	0.22	0.13	0.23	0.12	0.18	0.10	0.30	0.45	1.24	0.30	Stable	15.00
RGCC	-0.12	-0.75	-0.22	-0.55	-0.96	-0.46	-0.26	-0.67	-0.44	-0.17	-0.42	-0.65	-0.38	-0.98	-0.70	-0.52	0.97	1.01	0.01	Stable	11.00
COMMD6	-0.30	-0.07	-0.19	-0.41	-0.11	-0.11	0.13	-0.06	0.07	-0.01	-0.39	-0.41	-0.08	-0.52	-0.30	-0.08	0.66	0.78	-0.36	Stable	11.00
EDNRB	-0.01	-0.01	-0.86	-0.29	-0.10	0.19	0.69	-0.01	0.56	-0.01	0.45	-0.56	-0.42	-0.01	0.41	0.04	0.39	-1.13	0.18	Stable	11.00
TGDS	0.00	0.00	-0.38	0.00	0.00	-0.03	0.00	0.00	0.00	0.00	0.00	0.00	0.00	0.00	0.00	0.00	0.38	14.08	3.82	Stable	11.00
ENSSSCG00000009498	-0.31	-0.42	-0.13	-0.19	-0.31	0.07	-0.25	-0.42	-0.31	-0.19	-0.19	-0.25	-0.24	-0.31	-0.25	-0.25	0.06	1.56	0.65	Stable	11.00
ENSSSCG00000009501	0.08	0.08	0.07	0.27	0.08	0.23	0.08	0.08	0.08	0.08	0.08	0.08	0.46	0.08	0.08	0.08	0.11	0.46	-1.11	Stable	11.00
UGGT2	0.21	0.25	-0.02	0.18	0.21	-0.06	0.21	0.21	0.18	0.25	0.25	0.25	0.08	0.25	0.18	0.25	0.86	1.05	0.07	Stable	11.00
UBAC2	-1.09	-0.58	-1.12	-0.62	-0.25	-0.64	-1.09	-1.09	-0.45	-1.21	-0.83	-0.68	-0.64	-1.35	-1.05	-1.03	0.13	1.33	0.41	Stable	11.00
GPR183	0.21	0.21	0.21	0.00	0.21	0.00	0.21	0.21	0.21	0.21	0.21	0.21	0.00	0.21	0.21	0.21	0.09	1.57	0.65	Stable	11.00
ENSSSCG00000009523	-0.48	-0.74	-0.73	-0.33	-0.08	0.01	-0.39	-0.52	0.05	-0.55	-0.16	-0.11	-0.28	-0.30	-0.49	0.01	0.04	2.40	1.26	Up	11.00
POGLUT2	-0.21	-0.17	-0.25	-0.23	-0.19	-0.43	0.06	-0.21	-0.21	-0.30	0.32	-0.11	0.07	-0.23	-0.25	0.07	0.40	1.81	0.85	Stable	11.00
ENSSSCG00000009534	0.01	-0.33	-0.21	-0.32	-0.42	-0.46	-0.12	-0.12	0.02	-0.19	-0.20	-0.25	-0.34	-0.13	-0.20	-0.29	0.01	0.43	-1.21	Down	11.00
ARHGEF7	-0.22	-0.18	-0.46	-0.13	-0.03	-0.04	-0.04	-0.04	-0.15	-0.13	-0.54	-0.41	-0.19	-0.12	-0.04	-0.19	0.54	0.75	-0.41	Stable	11.00
GRTP1	0.18	0.21	0.13	-0.43	-0.25	-0.27	0.08	0.26	0.10	0.20	0.02	-0.03	-0.09	0.08	-0.03	-0.16	0.01	-0.99	-0.02	Stable	11.00
CUL4A	-0.01	-0.17	0.00	-0.21	-0.10	-0.06	-0.08	-0.10	-0.08	-0.16	-0.12	-0.01	-0.17	-0.08	-0.03	-0.18	0.08	0.55	-0.86	Stable	11.00
TMCO3	-0.04	-0.27	0.11	0.00	0.00	0.00	-0.09	-0.23	0.04	-0.26	0.01	0.00	-0.13	-0.14	0.03	-0.10	0.84	1.20	0.26	Stable	11.00
RASA3	0.00	-0.09	0.00	-0.32	-0.16	-0.16	0.01	0.16	0.00	-0.01	-0.20	-0.02	-0.06	-0.04	0.09	-0.15	0.01	-0.12	-3.08	Down	11.00
PSPC1	-0.43	-0.13	-0.65	0.46	-0.81	-0.75	-0.86	-0.60	-1.98	-1.47	-0.89	-1.86	-1.81	-0.69	-1.19	-0.92	0.58	0.81	-0.30	Stable	11.00
RNF17	0.64	0.21	0.64	-0.40	-0.24	0.06	0.25	0.67	0.39	0.68	0.34	0.36	-0.11	0.61	0.15	-0.24	0.03	7.75	2.95	Up	11.00
AUH	-0.24	-0.47	-0.49	-0.40	-0.40	-0.40	-0.17	-0.24	-0.49	-0.17	-0.40	-0.40	-0.47	-0.17	-0.49	-0.47	0.49	0.88	-0.18	Stable	14.00
SPTLC1	0.71	0.73	0.63	0.71	0.63	0.27	0.62	0.69	0.69	0.17	0.77	0.70	0.71	0.37	0.89	0.13	0.18	1.30	0.38	Stable	14.00

ENSSSCG00000009595	0.34	0.39	0.25	0.41	0.60	0.32	0.38	0.15	0.25	0.65	0.39	0.51	0.44	0.48	0.25	0.38	0.01	0.67	-0.57	Stable	14.00
GFRA2	-0.12	-0.23	0.76	0.24	0.24	0.10	0.34	0.52	0.59	0.49	-0.21	-0.07	0.49	0.47	-0.16	0.31	0.66	1.37	0.46	Stable	14.00
DMTN	-0.84	-0.65	-0.35	-0.64	-0.51	-0.46	-1.07	-0.74	-0.86	-0.98	0.34	-0.67	-0.82	-1.02	-0.10	-0.33	0.31	1.38	0.46	Stable	14.00
HR	0.05	0.07	0.08	0.05	0.05	-0.08	0.05	0.14	0.14	0.05	0.09	0.05	0.11	0.05	0.12	0.05	0.12	1.89	0.92	Stable	14.00
REEP4	-0.45	0.06	-0.25	0.67	0.14	0.01	-0.06	0.21	0.48	0.53	-0.23	0.08	0.38	0.57	-0.02	0.53	0.25	0.25	-2.00	Stable	14.00
SFTPC	0.11	0.03	0.45	0.18	0.03	0.16	0.09	0.32	0.28	0.09	0.21	0.13	0.05	0.24	0.05	0.13	0.23	1.62	0.69	Stable	14.00
POLR3D	-0.18	-0.18	-0.08	-0.16	-0.17	-0.08	-0.16	-0.18	-0.18	-0.16	-0.18	-0.18	-0.18	-0.15	-0.18	-0.17	0.98	1.00	0.00	Stable	14.00
CCAR2	0.74	0.03	0.39	0.08	-0.02	0.26	0.56	0.19	0.02	0.31	0.36	0.71	-0.02	0.31	-0.17	-0.03	0.74	1.23	0.30	Stable	14.00
DOCK5	-0.72	-0.36	-0.23	-0.55	-0.72	-0.72	-0.36	-0.63	-0.88	-0.72	-0.50	-0.37	-0.82	-0.54	-0.38	-0.72	0.21	0.80	-0.31	Stable	14.00
STMN4	-0.15	-0.27	-0.13	-0.03	-0.03	-0.14	-0.15	0.08	0.64	0.08	-0.07	-0.15	-0.03	-0.10	-0.27	-0.02	0.98	0.93	-0.10	Stable	14.00
EPHX2	0.54	0.03	0.53	0.94	0.82	0.84	0.72	1.14	2.00	1.20	0.89	0.68	0.89	0.35	0.21	0.62	0.48	0.80	-0.32	Stable	14.00
CLU	0.57	-0.21	0.50	0.14	0.04	0.08	0.53	0.68	1.00	0.16	0.27	0.31	0.16	0.65	-0.12	0.21	0.10	2.61	1.39	Stable	14.00
PRSS55	0.41	0.35	0.09	0.05	0.40	0.58	0.43	-0.49	-0.64	-0.26	0.58	0.41	-0.05	-0.07	0.40	0.40	0.30	0.23	-2.10	Stable	14.00
HPF1	-0.44	-0.23	0.05	-0.40	-0.40	-0.40	-0.23	-0.03	-0.13	-0.44	-0.29	-0.40	-0.40	-0.44	-0.12	-0.44	0.01	0.49	-1.02	Down	14.00
NEK1	-0.07	-0.07	-0.06	-0.22	-0.09	-0.10	-0.03	-0.02	0.00	-0.31	-0.07	-0.10	-0.28	-0.25	-0.04	-0.07	0.07	0.44	-1.19	Stable	14.00
SH3RF1	-0.23	-0.09	0.07	-0.22	-0.12	-0.23	-0.12	0.04	0.00	-0.22	0.05	-0.23	-0.23	-0.23	-0.14	-0.12	0.19	0.54	-0.89	Stable	14.00
CBR4	0.11	-0.14	-1.77	-0.17	-0.38	-0.02	0.58	-0.50	0.41	0.04	-1.29	-0.29	-0.25	-0.09	-0.73	0.25	1.00	1.01	0.01	Stable	14.00
STX2	0.06	0.13	0.00	0.06	0.05	0.05	-0.08	0.11	0.21	0.21	0.03	0.06	0.06	0.06	0.13	0.05	0.86	1.09	0.13	Stable	14.00
CCDC92	0.37	0.34	-0.50	0.15	0.17	0.43	0.35	-0.36	-0.17	-0.06	0.47	0.25	0.51	0.43	0.07	0.24	0.19	0.25	-2.02	Stable	14.00
DNAH10	0.02	-0.03	-0.69	0.03	-0.17	-0.24	0.02	-0.79	-0.51	-0.25	0.04	0.03	0.03	-0.05	-0.27	-0.24	0.16	3.02	1.59	Stable	14.00
TCTN2	0.13	0.78	0.74	0.87	0.83	0.70	0.08	1.28	1.33	0.72	1.22	0.85	1.03	0.38	0.39	0.62	0.27	0.75	-0.42	Stable	14.00
GTF2H3	-0.10	-0.49	-1.02	-0.62	-0.21	-0.52	-0.12	-1.25	-1.22	-0.69	-0.77	-0.47	-0.83	-0.83	-0.51	-0.64	0.59	1.17	0.22	Stable	14.00
EIF2B1	-0.32	-1.61	-2.18	-1.68	-1.71	-1.39	-0.50	-1.96	-1.82	-1.30	-2.09	-1.20	-1.37	-0.99	-0.97	-1.16	0.49	0.87	-0.20	Stable	14.00
DDX55	-0.23	-0.47	-0.21	-0.38	-0.42	-0.40	-0.09	0.07	0.15	-0.08	-0.38	-0.26	-0.36	-0.24	-0.37	-0.50	0.07	0.50	-1.00	Stable	14.00
C12orf65	-0.02	-0.17	-0.60	-0.31	-0.57	-0.83	-0.02	-0.88	-0.84	-0.45	-0.29	-0.16	-0.10	-0.49	-0.78	-0.88	0.89	1.05	0.07	Stable	14.00
ABCB9	-0.67	0.02	0.16	0.89	-0.40	-0.81	0.06	0.34	0.16	-0.11	0.59	0.33	0.05	-0.48	-0.35	-1.12	0.93	1.33	0.41	Stable	14.00
HCAR1	-0.12	0.10	0.01	0.38	0.52	0.23	0.02	-0.16	-0.05	-0.03	0.39	0.14	0.06	0.07	0.13	0.40	0.01	0.00	-7.79	Down	14.00
MLXIP	0.28	0.37	0.34	0.32	0.31	0.41	0.23	0.14	0.00	0.00	0.32	0.26	0.37	0.38	0.40	0.43	0.60	0.88	-0.19	Stable	14.00
B3GNT4	-0.11	-0.14	-0.25	-0.17	-0.11	-0.15	0.03	-0.19	-0.04	0.24	-0.21	-0.07	-0.25	0.03	-0.24	-0.03	0.76	1.22	0.29	Stable	14.00
MORN3	-0.01	-0.31	-0.04	-0.17	-0.19	-0.26	-0.01	-0.01	-0.01	-0.05	-0.24	-0.12	-0.26	-0.21	-0.41	-0.26	0.32	0.65	-0.62	Stable	14.00
IFT81	0.22	0.26	0.46	0.16	0.20	0.29	0.19	0.33	0.00	0.00	0.16	0.08	0.29	0.17	0.36	0.28	0.32	1.35	0.43	Stable	14.00
RAD9B	0.21	0.55	0.34	0.50	0.32	0.45	0.19	0.00	-0.02	0.03	0.39	0.12	0.55	0.34	0.66	0.44	0.55	0.81	-0.30	Stable	14.00
TCTN1	0.43	-0.04	0.56	-0.34	-0.38	0.18	0.39	0.24	-0.18	-0.10	-0.60	-0.19	-0.33	0.31	0.63	-0.09	0.00	-1.27	0.35	Stable	14.00
HVCN1	-0.05	-0.35	-0.27	-0.18	-0.11	-0.17	-0.02	-0.20	0.00	0.00	-0.18	-0.05	-0.28	-0.27	-0.32	-0.31	0.71	1.15	0.20	Stable	14.00
CUX2	0.27	-0.07	0.08	0.02	0.01	0.15	0.20	0.10	0.17	0.01	-0.11	0.05	-0.06	0.08	0.02	-0.06	0.05	25.23	4.66	Up	14.00
PHETA1	-0.24	-0.69	-0.36	-0.45	-0.64	-0.62	-0.38	-0.08	0.00	-0.17	-0.50	-0.38	-0.51	-0.14	-0.53	-0.57	0.09	0.63	-0.67	Stable	14.00
ACAD10	-0.16	-0.16	0.18	-0.51	-0.50	-0.22	0.01	0.06	-0.19	-0.05	-0.35	-0.10	0.14	0.30	0.28	-0.30	0.02	-0.17	-2.54	Down	14.00
ENSSSCG00000009848	0.00	0.14	0.00	0.00	0.00	0.00	-0.04	-0.04	0.00	-0.04	0.00	-0.04	0.18	0.00	0.36	0.18	0.77	1.50	0.58	Stable	14.00
TESC	-0.08	0.05	0.20	-0.16	-0.14	-0.10	-0.06	0.04	-0.08	-0.06	-0.16	-0.08	-0.14	-0.04	-0.06	-0.10	0.01	0.02	-5.63	Down	14.00
SDSL	-0.25	-0.10	-0.09	0.10	-0.15	-0.21	0.01	-0.33	0.04	0.06	0.10	-0.18	-0.24	-0.30	0.30	-0.24	0.97	0.96	-0.06	Stable	14.00
DTX1	-0.22	-0.33	-0.02	-0.23	-0.22	-0.20	-0.28	-0.22	-0.28	-0.29	-0.23	-0.31	-0.08	0.05	-0.33	-0.16	0.82	0.94	-0.09	Stable	14.00
RASAL1	0.86	0.54	0.34	0.65	0.87	0.57	0.87	0.93	0.85	0.59	0.77	0.47	0.43	0.74	0.57	0.60	0.34	1.14	0.19	Stable	14.00
IQCD	-0.48	-0.22	-0.83	-0.06	0.15	0.27	-0.74	-0.54	-0.65	-0.80	-0.31	-0.55	-0.36	-0.20	-0.05	-0.07	0.15	2.13	1.09	Stable	14.00
SLC8B1	-0.20	0.05	0.21	0.30	0.15	0.06	0.10	-0.06	0.12	0.16	0.24	-0.01	0.23	0.04	-0.17	0.21	0.03	0.07	-3.89	Down	14.00
RPH3A	0.59	0.01	0.20	0.19	0.33	0.62	0.66	0.50	0.59	0.54	0.23	0.43	0.58	0.59	-0.06	0.63	0.62	0.86	-0.21	Stable	14.00

ALDH2	-0.10	0.08	0.17	-0.08	-0.25	-0.07	-0.10	0.07	0.05	-0.14	-0.21	0.25	0.13	0.01	0.27	-0.02	0.18	-1.13	0.18	Stable	14.00
TMEM116	-0.14	-0.14	-0.16	-0.07	-0.07	-0.14	0.12	-0.14	-0.13	-0.14	-0.07	-0.14	-0.14	-0.14	-0.14	-0.14	0.92	0.97	-0.05	Stable	14.00
BICDL1	-0.79	-0.34	-0.74	-0.68	-0.60	-0.42	-0.90	-0.74	-0.92	-1.04	-0.58	-0.58	-0.64	-0.55	-0.18	-0.56	0.95	1.01	0.02	Stable	14.00
RNF10	-0.76	-1.11	-1.14	0.66	1.00	-0.58	-2.12	-1.82	-1.36	-2.15	0.54	-0.28	0.26	-0.73	-1.31	0.08	0.01	21.87	4.45	Up	14.00
TRPV4	-0.19	0.51	0.54	0.54	0.51	0.56	0.04	-0.39	0.40	0.46	0.58	0.21	0.57	0.51	0.24	0.55	0.06	0.42	-1.25	Stable	14.00
MVK	-0.32	-0.31	-0.29	-0.24	-0.20	-0.46	-0.42	-0.23	-0.42	-0.55	-0.29	-0.27	-0.53	-0.29	-0.38	-0.43	0.47	0.89	-0.17	Stable	14.00
MMAB	0.61	0.49	0.82	0.48	0.45	0.71	0.81	0.63	0.78	0.67	0.29	0.64	0.98	0.44	0.03	0.58	0.85	0.96	-0.06	Stable	14.00
UBE3B	-0.72	-0.39	-0.14	0.10	0.10	-0.07	-0.20	-0.83	-0.15	-0.24	0.12	-0.37	0.19	-0.24	-0.33	0.03	0.01	19.45	4.28	Up	14.00
SART3	-0.02	0.20	-0.21	-0.40	-0.40	-0.41	0.10	0.15	0.31	0.15	-0.56	0.02	-0.01	0.39	0.41	-0.21	0.00	-0.73	-0.45	Stable	14.00
SGSM1	0.19	-0.02	0.60	0.77	0.72	0.68	-0.26	0.36	-0.17	-0.24	0.73	0.66	0.17	-0.16	-0.70	0.57	0.02	-0.04	-4.65	Down	14.00
ASPHD2	0.10	0.24	0.53	0.08	0.08	0.10	0.44	0.10	-0.15	0.06	0.08	0.10	-0.01	-0.04	0.02	0.13	0.38	2.02	1.01	Stable	14.00
TPST2	-0.27	0.75	0.55	0.47	0.30	0.38	-0.30	-0.39	0.74	-0.06	0.37	0.13	0.59	0.38	1.00	0.11	0.93	1.07	0.09	Stable	14.00
EMID1	0.07	-0.38	-0.19	-0.11	-0.20	-0.29	-0.13	0.15	-0.25	0.09	-0.20	-0.13	-0.18	-0.11	-0.36	-0.29	0.89	0.93	-0.11	Stable	14.00
MTMR3	-0.10	0.38	0.37	0.21	0.31	0.23	-0.02	-0.10	-0.10	0.01	0.23	-0.05	-0.03	-0.10	0.04	0.26	0.30	0.33	-1.59	Stable	14.00
SF3A1	-0.38	-0.06	0.16	-0.30	-0.30	-0.07	-0.31	-0.38	-0.37	-0.10	-0.30	-0.33	-0.33	-0.38	-0.33	-0.07	0.71	1.14	0.19	Stable	14.00
PES1	0.49	0.01	-0.04	0.16	0.00	0.08	0.18	0.48	0.05	0.18	0.08	0.41	0.10	0.41	0.17	0.08	0.37	1.60	0.68	Stable	14.00
TCN2	0.19	-0.55	-0.57	-0.45	-0.28	-0.42	-0.35	0.17	0.52	-0.35	-0.52	-0.09	0.10	0.30	-0.12	-0.39	0.16	0.17	-2.54	Stable	14.00
SLC35E4	0.44	-0.48	-0.13	0.31	0.31	0.15	0.15	0.30	-0.25	-0.02	0.31	0.58	-0.03	0.19	-0.32	0.27	0.09	-0.05	-4.38	Stable	14.00
LIMK2	0.00	-0.66	-0.56	-0.11	-0.10	-0.42	-0.23	-0.01	0.03	-0.49	-0.11	0.05	-0.10	0.06	-0.27	-0.42	0.94	0.95	-0.07	Stable	14.00
SLC5A4	-0.37	0.16	0.30	-0.03	-0.26	-0.08	0.15	-0.24	-0.13	0.09	0.06	-0.24	-0.05	-0.39	-0.09	0.00	0.91	1.19	0.25	Stable	14.00
GNAZ	0.08	-0.09	-0.03	-0.03	-0.02	0.03	-0.03	0.08	0.92	-0.03	-0.03	0.08	0.38	0.41	0.13	-0.02	0.30	4.09	2.03	Stable	14.00
GGT5	0.11	0.57	0.49	0.24	0.38	0.25	0.16	0.45	-0.24	0.16	0.15	0.55	-0.04	0.49	0.41	0.32	0.63	1.22	0.29	Stable	14.00
LRRC75B	0.20	0.41	0.16	0.01	0.23	0.30	0.01	0.35	-1.48	0.19	0.01	0.32	-0.56	-0.41	0.36	-0.01	0.66	-0.82	-0.29	Stable	14.00
GUCD1	0.67	-0.02	0.50	0.65	0.70	0.73	0.66	0.72	1.11	0.62	0.64	0.66	0.86	0.93	0.50	0.57	0.72	0.93	-0.10	Stable	14.00
AIFM3	0.13	0.00	0.20	0.22	0.04	0.11	0.22	0.15	-0.89	0.11	0.22	0.33	-0.28	-0.14	0.00	0.04	0.35	-0.44	-1.18	Stable	14.00
P2RX6	-0.02	-0.60	-0.13	-0.01	0.07	0.01	0.01	-0.05	-0.05	0.05	-0.06	-0.17	-0.15	-0.09	-0.42	0.06	0.12	6.84	2.77	Stable	14.00
LGALS8	0.50	0.05	0.25	0.12	0.21	0.32	0.42	0.52	0.00	0.35	0.30	0.50	0.23	0.23	0.22	0.28	0.87	0.95	-0.07	Stable	14.00
ENSSSCG00000010161	-0.12	0.34	0.23	-0.02	0.06	-0.44	-0.12	-0.02	-0.17	-0.25	-0.17	-0.02	-0.31	-0.11	-0.12	0.03	0.19	0.07	-3.77	Stable	14.00
ENSSSCG00000010165	-0.65	-0.22	-0.63	-0.12	-0.06	-0.56	-0.57	-0.65	-0.31	-0.79	-0.06	-0.45	-0.06	-0.55	-0.24	0.10	0.10	1.91	0.93	Stable	14.00
NTPCR	-0.11	0.01	0.46	0.00	-0.60	-0.02	0.42	0.04	-0.14	0.58	-0.24	0.10	-0.09	-0.22	-0.49	-0.46	0.61	0.04	-4.83	Stable	14.00
GNPAT	-0.26	-0.06	-0.09	-0.48	-0.28	-0.33	-0.40	-0.29	-0.19	-0.63	-0.74	-0.30	-0.62	-0.47	-0.02	0.00	0.08	0.53	-0.92	Stable	14.00
C1orf131	-0.04	0.00	-0.10	0.13	-0.14	-0.26	0.03	-0.02	-0.17	-0.20	-0.08	0.17	-0.10	0.10	-0.04	-0.13	0.47	0.41	-1.27	Stable	14.00
GALNT2	0.24	0.41	0.37	0.06	0.05	0.28	-0.04	0.23	0.09	-0.12	0.01	0.35	-0.14	0.09	0.27	-0.08	0.08	3.94	1.98	Stable	14.00
ABCB10	-0.10	-0.04	-0.17	-0.02	0.01	0.01	-0.02	-0.06	-0.06	0.04	0.07	-0.16	0.07	-0.06	0.04	0.04	0.08	-7.97	2.99	Stable	14.00
ZNF25	0.12	0.10	0.13	0.06	0.02	-0.01	0.12	0.08	0.07	-0.15	-0.07	0.19	-0.09	0.10	0.33	-0.19	0.01	-4.05	2.02	Up	14.00
CCDC6	0.08	0.11	0.16	-0.06	0.01	-0.06	-0.16	-0.05	0.13	-0.35	0.01	-0.07	-0.12	-0.11	-0.12	0.09	0.27	-0.06	-4.08	Stable	14.00
VPS26A	-0.19	-0.15	-0.32	-0.17	-0.17	-0.30	0.10	-0.13	0.09	0.02	-0.17	-0.32	-0.17	0.00	-0.11	-0.22	0.14	0.48	-1.06	Stable	14.00
SUPV3L1	-0.41	-0.25	-0.35	-0.13	-0.13	-0.19	-0.13	-0.37	-0.16	-0.10	-0.26	-0.37	-0.26	-0.35	-0.38	-0.13	0.05	1.54	0.63	Stable	14.00
HKDC1	0.70	0.24	0.17	0.10	0.11	0.55	0.66	0.76	0.53	0.35	0.14	0.15	0.10	0.72	0.17	0.11	0.02	2.47	1.30	Up	14.00
AIFM2	0.63	-0.28	0.06	0.00	0.02	-0.03	-0.11	0.54	0.40	0.05	0.13	0.42	-0.15	0.55	0.10	-0.36	0.14	36.63	5.19	Stable	14.00
PCBD1	0.40	0.32	0.40	0.43	0.33	0.27	0.31	0.40	0.36	0.39	0.31	0.41	0.43	0.39	0.30	0.24	0.85	1.02	0.02	Stable	14.00
CDH23	-0.68	-1.01	-0.82	-0.06	-0.23	-0.41	0.17	-0.51	-0.50	-0.15	0.03	-0.81	0.04	-0.31	-0.92	-0.23	0.06	2.51	1.33	Stable	14.00
PSAP	-0.25	-0.92	-1.14	-0.29	-0.60	-0.47	0.17	-0.36	-0.31	-0.01	-0.18	-1.20	-0.10	-0.48	-0.26	-0.77	0.97	0.98	-0.02	Stable	14.00
MICU1	-0.01	-0.11	-0.09	-0.04	0.09	-0.10	-0.05	0.04	-0.07	-0.08	-0.01	0.20	-0.04	0.01	-0.06	0.05	0.23	-5.10	2.35	Stable	14.00
ANXA11	-0.01	-0.08	-0.28	-0.34	-0.90	-0.50	-0.29	-0.36	-0.02	-0.30	-0.44	-0.46	-0.01	-0.05	-0.25	-0.90	0.02	0.34	-1.54	Down	14.00

PLAC9	-0.10	-0.08	-0.35	-0.45	-1.11	-0.43	-0.46	-1.01	-0.12	-0.78	-0.34	-0.87	0.07	-0.08	-0.30	-1.11	0.11	0.50	-1.00	Stable	14.00
TSPAN14	0.20	-0.01	-0.04	-0.01	0.06	-0.04	-0.09	0.36	-0.02	-0.04	-0.01	0.33	-0.02	0.26	0.02	0.06	0.56	2.06	1.05	Stable	14.00
GHITM	0.12	0.45	0.00	0.15	0.14	-0.17	0.07	0.23	0.19	0.07	0.05	0.23	0.07	0.20	0.55	0.14	0.09	2.64	1.40	Stable	14.00
CDHR1	-0.25	0.00	0.00	-0.30	-0.47	-0.47	-0.22	-0.47	0.00	-0.22	-0.24	-0.47	-0.26	-0.25	-0.21	-0.47	0.02	0.49	-1.04	Down	14.00
GRID1	-0.02	0.37	-0.02	0.04	0.16	0.11	0.48	-0.02	0.37	0.04	0.07	0.13	-0.02	-0.02	0.46	0.16	0.23	2.31	1.21	Stable	14.00
BMPR1A	0.07	0.38	0.13	0.12	0.04	0.59	0.35	0.29	0.23	0.01	0.13	-0.08	0.21	0.07	0.29	0.04	0.28	1.72	0.78	Stable	14.00
ENSSSCG00000010369	0.00	-0.29	-0.01	-0.31	-0.61	-0.27	-0.04	-0.15	-0.21	-0.31	-0.31	-0.31	-0.14	0.00	-0.01	-0.61	0.00	0.25	-1.99	Down	14.00
ENSSSCG00000010370	-0.21	0.11	-0.23	-0.28	-0.36	-0.35	-0.24	-0.12	0.19	-0.22	-0.28	-0.31	-0.18	-0.21	0.10	-0.36	0.01	0.26	-1.97	Down	14.00
NCOA4	-0.03	-0.21	-0.14	0.01	0.04	-0.04	-0.04	-0.04	-0.21	0.02	0.04	0.01	-0.18	-0.03	-0.16	0.04	0.02	18.76	4.23	Up	14.00
MARCHF8	0.12	-0.11	-0.05	0.00	0.04	-0.09	-0.17	-0.34	-0.11	0.05	-0.02	0.08	0.08	0.12	-0.04	0.04	0.13	-3.17	1.66	Stable	14.00
ALOX5	0.00	-0.06	0.03	-0.05	-0.26	-0.17	-0.18	-0.09	-0.06	-0.13	-0.26	-0.13	-0.24	0.00	-0.11	-0.26	0.00	0.32	-1.63	Down	14.00
MINPP1	-0.09	-0.12	-0.29	-0.23	-0.22	-0.15	-0.17	-0.24	-0.18	-0.29	-0.23	-0.09	-0.23	-0.13	-0.08	-0.22	0.18	0.78	-0.36	Stable	14.00
LIPM	-0.56	0.21	0.33	0.05	0.24	0.32	-0.15	0.08	-0.08	0.12	0.29	0.12	0.53	-0.37	-0.17	0.40	0.01	-0.34	-1.54	Down	14.00
STAMBPL1	-0.48	-0.28	-0.13	0.03	-0.27	-0.13	-0.53	-0.35	0.01	-0.24	-0.16	-0.34	-0.13	-0.14	-0.48	-0.33	0.24	1.52	0.60	Stable	14.00
LIPA	0.33	0.33	0.23	0.13	0.28	0.28	0.24	0.22	0.22	0.33	0.11	0.33	0.22	0.13	0.20	0.28	0.87	0.97	-0.04	Stable	14.00
PPP1R3C	-1.09	-0.61	-0.40	-0.39	-0.39	-0.39	-0.75	-0.74	-0.59	-0.75	-0.39	-0.73	-0.39	-0.68	-0.26	-0.39	0.14	1.35	0.43	Stable	14.00
IDE	0.01	0.23	-0.04	-0.48	-0.29	-0.45	-0.13	-0.06	0.36	-0.21	-0.37	-0.27	-0.55	-0.11	0.42	-0.34	0.00	-0.23	-2.13	Down	14.00
PDE6C	0.43	0.22	-0.08	0.27	-0.01	0.27	0.30	0.83	0.96	0.00	-0.01	0.40	-0.03	0.66	0.18	-0.01	0.04	4.00	2.00	Up	14.00
NOC3L	0.73	0.79	0.12	0.67	0.62	0.70	0.23	0.61	0.73	0.95	0.48	0.83	0.91	0.74	0.20	0.61	0.11	0.72	-0.47	Stable	14.00
ALDH18A1	-0.12	-0.09	-0.23	-0.45	-0.29	-0.45	0.16	-0.33	-0.15	-0.13	-0.39	-0.54	-0.57	-0.43	-0.05	-0.29	0.01	0.40	-1.32	Down	14.00
TCTN3	-0.11	-0.07	-0.09	-0.10	0.26	-0.08	0.11	-0.09	-0.08	-0.12	0.08	-0.12	-0.13	-0.11	-0.11	0.08	0.38	4.24	2.08	Stable	14.00
TLL2	-0.06	-0.39	0.13	-0.56	-0.31	-0.63	0.02	-0.30	-0.32	-0.14	-0.22	-0.22	-0.49	-0.41	0.21	-0.38	0.05	0.38	-1.39	Stable	14.00
SLIT1	-0.58	0.06	0.06	0.28	-0.09	0.33	-0.65	0.16	-0.30	-0.52	-0.03	-0.17	0.10	0.12	-0.32	-0.15	0.33	5.89	2.56	Stable	14.00
LCOR	0.05	0.51	-0.31	-0.96	-0.40	-0.90	0.31	-0.41	-0.19	-0.67	-0.62	-0.42	-1.22	0.04	-0.02	-0.20	0.00	0.00	-7.72	Down	14.00
HOGA1	0.00	-0.04	-0.18	-0.20	-0.08	-0.20	-0.07	-0.16	0.00	-0.06	-0.08	-0.04	-0.08	-0.16	-0.01	-0.08	0.48	0.76	-0.40	Stable	14.00
ZFYVE27	0.00	0.19	0.38	0.10	0.39	0.15	0.20	-0.06	0.00	0.20	0.39	0.17	0.39	-0.01	0.06	0.39	0.02	0.34	-1.54	Down	14.00
PYROXD2	0.30	-0.09	-0.18	-0.31	-0.29	-0.34	0.09	-0.05	-0.08	0.11	-0.31	-0.10	-0.31	-0.20	0.18	-0.36	0.01	0.02	-5.63	Down	14.00
PKD2L1	0.77	-0.38	-0.09	0.64	-0.19	0.10	0.44	0.46	-0.65	0.81	-0.29	0.27	0.01	0.45	0.29	-0.10	0.99	1.02	0.03	Stable	14.00
DPCD	-0.05	-0.18	-0.18	0.03	0.01	-0.18	0.07	-0.18	-0.18	0.06	-0.18	-0.18	-0.18	0.03	-0.18	0.03	0.57	1.43	0.52	Stable	14.00
NPM3	0.30	0.22	0.04	0.11	0.10	0.07	0.10	0.48	0.49	0.09	0.04	0.19	0.07	0.35	0.27	0.10	0.01	2.94	1.56	Up	14.00
ARMH3	0.03	0.05	-0.01	-0.05	-0.03	0.00	-0.02	0.10	0.10	-0.02	0.00	0.07	0.00	0.05	0.05	-0.02	0.03	-7.89	2.98	Up	14.00
MFSD13A	-0.26	-0.28	-0.23	-0.05	-0.18	-0.06	-0.17	-0.33	-0.33	-0.09	-0.13	-0.19	-0.06	-0.12	-0.12	-0.18	0.01	1.95	0.96	Stable	14.00
PDCD11	0.02	-0.02	-0.02	0.02	-0.02	-0.02	0.00	-0.02	-0.02	-0.02	-0.02	-0.02	-0.02	0.01	-0.02	-0.02	0.40	0.48	-1.06	Stable	14.00
XPNPEP1	0.00	0.02	0.03	0.02	0.03	0.03	0.02	0.00	0.02	0.02	0.03	0.00	0.03	0.00	0.02	0.03	0.06	0.50	-1.00	Stable	14.00
ZDHHC6	-0.49	-0.38	-0.32	-0.32	-0.28	-0.33	-0.34	-0.49	-0.32	-0.32	-0.33	-0.27	-0.31	-0.38	-0.08	-0.28	0.36	1.15	0.20	Stable	14.00
ABLIM1	0.00	0.01	-0.01	0.00	0.00	0.00	0.04	0.00	-0.07	0.00	0.16	0.00	0.22	-0.02	0.20	-0.11	0.74	0.53	-0.90	Stable	14.00
TRUB1	0.00	0.03	0.02	0.00	0.00	0.06	0.00	0.00	0.12	0.00	0.10	0.06	0.03	0.06	0.01	0.03	0.84	0.88	-0.18	Stable	14.00
FAM204A	0.03	-0.91	-0.26	-0.11	-0.08	-0.04	-0.10	0.00	-0.04	-0.76	-0.08	-0.24	-0.16	-0.12	-0.07	-0.16	0.90	0.92	-0.13	Stable	14.00
EIF3A	0.01	0.02	0.02	-0.32	-0.63	-0.36	0.00	-0.09	-0.35	-0.24	-0.19	-0.37	-0.59	-0.03	-0.12	-0.53	0.00	0.16	-2.61	Down	14.00
DENND10	0.00	0.41	0.18	0.07	0.14	0.07	0.00	0.00	0.07	0.29	0.16	0.16	0.14	0.00	0.00	0.14	0.30	0.57	-0.81	Stable	14.00
ATE1	0.35	0.85	-1.41	-0.24	-0.19	-0.32	-0.77	-0.04	-1.38	0.08	-0.38	-0.59	-0.15	-1.23	0.05	-0.58	0.65	1.51	0.59	Stable	14.00
ACADSB	-0.02	-0.10	0.12	0.13	0.61	-0.03	-0.12	0.00	-0.17	0.04	0.13	0.27	0.59	0.28	0.29	0.21	0.07	0.15	-2.72	Stable	14.00
ENSSSCG00000010734	-0.47	-0.67	-0.13	-0.79	-1.46	-0.69	-0.43	-0.44	-0.07	-0.52	-0.96	-0.68	-1.35	-0.38	-0.11	-0.72	0.00	0.38	-1.41	Down	14.00
LHPP	0.12	0.12	0.22	0.10	0.17	0.10	0.09	0.16	0.01	0.01	0.21	0.20	0.21	0.23	0.03	0.00	0.91	0.96	-0.06	Stable	14.00
ABRAXAS2	0.81	0.43	1.07	0.65	0.84	0.43	0.62	0.57	0.53	0.37	0.46	0.68	0.71	1.19	0.82	0.50	0.15	1.30	0.38	Stable	14.00

UROS	0.35	1.18	0.47	-0.07	0.84	0.76	0.35	0.31	0.11	0.14	0.62	0.92	1.03	0.35	0.35	0.44	0.41	0.74	-0.43	Stable	14.00
BCCIP	0.19	0.66	0.01	0.39	0.51	0.24	0.08	-0.11	0.51	0.47	0.21	0.10	0.48	0.19	0.30	0.65	0.20	0.60	-0.73	Stable	14.00
FANK1	-0.21	-0.19	-0.24	0.26	-0.12	-0.13	-0.34	-0.31	-0.34	0.04	-0.33	-0.47	-0.32	-0.26	0.15	0.06	0.39	1.74	0.80	Stable	14.00
LRRC27	0.33	0.98	0.36	1.07	0.30	0.23	0.32	0.32	0.36	0.33	0.38	0.41	0.64	0.33	0.73	0.30	0.96	1.01	0.02	Stable	14.00
INPP5A	0.40	0.51	0.31	0.75	0.25	0.16	0.60	0.56	0.31	0.32	0.43	0.02	0.02	0.32	0.65	0.32	0.10	1.62	0.70	Stable	14.00
CFAP46	0.00	-0.43	0.00	-0.45	-0.08	-0.24	-0.50	-0.55	0.00	0.00	-0.10	-0.15	-0.15	-0.08	-0.62	0.00	0.28	1.87	0.90	Stable	14.00
KNDC1	0.16	-0.23	0.00	0.05	0.12	0.31	-0.08	0.15	0.00	0.14	-0.04	0.14	0.14	-0.05	-0.05	0.27	0.02	-0.09	-3.52	Down	14.00
TUBGCP2	-0.01	-1.09	-0.19	-0.43	-0.58	-0.28	-0.80	-0.92	-0.19	-0.49	-0.03	-0.17	0.06	-0.17	-0.80	-1.01	0.44	1.42	0.51	Stable	14.00
ZNF511	-1.26	-0.43	-0.46	-0.45	-0.74	-0.59	-0.46	-0.43	-0.46	-0.32	-1.15	-1.29	-1.29	-0.20	-0.90	-0.19	0.38	0.76	-0.39	Stable	14.00
CYP2E1	0.43	0.42	0.38	0.42	0.65	0.33	0.47	0.50	0.38	0.53	0.39	0.65	0.63	0.52	0.48	0.63	0.14	0.85	-0.24	Stable	14.00
FUOM	0.93	0.72	0.57	0.91	1.14	0.75	0.89	0.89	0.57	0.86	0.92	1.11	0.93	0.93	1.44	1.15	0.37	0.89	-0.16	Stable	14.00
COG7	0.69	0.47	-0.39	0.30	-0.03	0.58	0.48	0.58	0.23	0.59	0.84	0.16	0.71	-0.03	0.63	0.30	0.57	0.77	-0.37	Stable	3.00
ESRRG	-0.09	-0.08	-0.09	-0.46	-0.04	-0.64	-0.27	-0.46	-0.64	-0.27	-0.64	0.10	-0.08	-0.27	-0.14	-0.64	0.54	0.76	-0.40	Stable	10.00
MARK1	0.22	0.13	0.24	0.17	0.24	0.16	0.22	0.46	0.26	0.25	0.38	0.11	0.38	0.11	0.22	0.26	0.83	0.95	-0.07	Stable	10.00
MTARC2	0.19	0.17	0.23	0.09	0.06	0.04	0.04	-0.09	0.16	0.06	0.17	0.14	0.08	-0.06	0.10	0.16	0.87	0.92	-0.12	Stable	10.00
ENSSSCG00000010829	-0.40	0.29	-0.69	0.26	0.08	0.32	-0.22	-0.17	0.43	-0.47	0.13	-0.53	0.29	-0.38	-0.26	0.43	0.22	-2.83	1.50	Stable	10.00
EPHX1	-0.47	0.03	-0.29	0.13	-0.97	0.13	-0.49	0.11	0.11	0.11	-0.33	-1.02	0.07	-0.40	-0.91	-0.32	0.95	1.05	0.07	Stable	10.00
ENSSSCG00000010855	0.30	0.03	-0.12	-0.48	-0.10	-0.52	0.09	0.02	0.09	-0.01	0.17	-0.10	-0.41	-0.42	0.10	-0.28	0.07	-0.05	-4.46	Stable	10.00
SDE2	1.17	0.77	1.35	0.37	1.65	0.31	1.19	0.53	0.93	1.37	0.84	1.65	0.05	1.00	1.05	0.90	0.67	1.12	0.16	Stable	10.00
ENSSSCG00000010860	-0.18	0.47	0.01	0.36	-0.73	0.34	-0.26	0.09	0.47	0.50	-0.17	-0.73	0.26	-0.17	-0.26	-0.24	0.73	-0.42	-1.25	Stable	10.00
COQ8A	0.23	0.46	0.22	0.62	0.31	0.83	0.31	0.03	-0.09	0.10	0.10	0.31	0.10	0.13	0.11	0.37	0.16	0.51	-0.96	Stable	10.00
SCCPDH	-0.20	-0.09	-0.22	-0.11	-0.25	-0.16	-0.30	-0.04	-0.29	-0.29	-0.27	-0.25	-0.11	-0.22	-0.19	-0.21	0.79	0.95	-0.08	Stable	10.00
DESI2	-0.54	-0.36	-0.48	-0.37	-0.13	-0.37	-0.44	-0.44	-0.83	-0.11	-0.19	-0.21	-0.30	-0.25	0.04	-0.19	0.09	1.78	0.83	Stable	10.00
ASPM	-0.28	-0.52	-0.57	-0.19	-0.62	-0.30	-0.37	-0.01	-0.05	-0.28	-0.51	-0.52	-0.73	-0.52	-0.38	-0.41	0.30	0.76	-0.39	Stable	10.00
SYT2	0.16	0.15	0.16	0.15	0.08	0.15	0.00	0.22	0.15	0.07	0.15	0.14	0.19	0.15	0.08	0.02	0.63	1.13	0.17	Stable	10.00
AOPEP	0.07	0.14	0.14	0.07	0.07	0.07	0.07	0.07	0.00	0.00	0.07	0.14	0.14	0.14	0.14	0.07	0.48	1.22	0.29	Stable	10.00
CTSL	0.54	0.56	0.53	0.33	0.21	0.31	0.54	0.58	0.58	0.62	0.55	0.60	0.53	0.56	0.26	0.36	0.25	1.18	0.24	Stable	10.00
DAPK1	0.22	0.34	0.37	0.07	0.10	-0.37	0.15	0.28	0.23	0.26	0.60	0.32	0.36	0.34	0.17	0.04	0.42	1.52	0.60	Stable	10.00
ENSSSCG00000010954	-0.22	-0.10	-0.05	-0.13	-0.05	-0.13	-0.21	0.00	-0.05	-0.14	-0.23	-0.05	0.00	-0.10	-0.29	-0.05	0.51	1.31	0.39	Stable	10.00
GOLM1	-0.41	-0.25	-0.28	-0.38	0.18	-0.43	-0.33	-0.19	-0.53	-0.70	-0.54	-0.22	-0.19	-0.25	-0.41	-0.07	0.73	1.13	0.17	Stable	10.00
NUDT2	-0.39	-0.88	-0.22	-0.37	-0.38	0.13	-0.33	0.12	-0.23	-0.46	-0.20	-0.88	-0.30	-0.87	-0.42	-0.24	0.69	1.19	0.25	Stable	10.00
BAG1	-0.30	-0.57	-0.10	-0.12	-0.19	0.23	0.11	0.25	0.26	0.26	0.35	-0.44	-0.20	-0.44	-0.16	-0.29	0.65	2.40	1.26	Stable	10.00
SMU1	0.00	0.00	-0.03	-0.11	-0.05	-0.11	-0.10	-0.05	-0.05	-0.12	-0.12	0.00	-0.03	0.00	-0.04	-0.10	0.05	0.43	-1.21	Down	10.00
TMEM215	-0.40	-0.81	-0.27	-0.58	-0.64	-0.21	-0.56	-0.13	0.10	-0.19	-0.20	-0.75	-0.47	-0.81	-0.57	-0.47	0.95	0.98	-0.03	Stable	10.00
WAC	0.07	0.13	0.00	-0.01	-0.09	0.07	-0.08	0.00	0.13	-0.01	0.00	0.05	0.00	0.13	0.07	0.05	0.16	7.06	2.82	Stable	10.00
ZNF438	0.11	0.33	0.10	0.26	0.10	0.08	0.12	0.11	0.09	0.33	0.11	0.13	0.33	0.12	0.08	0.07	0.38	0.75	-0.42	Stable	10.00
CUBN	-0.08	-0.04	-0.08	0.24	-0.26	-0.08	-0.08	-0.08	-0.04	0.18	0.14	-0.08	0.18	-0.08	-0.08	-0.26	0.33	-8.55	3.10	Stable	10.00
VIM	-0.26	0.06	0.02	0.18	0.16	-0.03	-0.82	0.05	0.12	0.17	0.21	-0.39	0.07	-0.42	-0.36	0.13	0.07	-3.23	1.69	Stable	10.00
STAM	0.00	0.08	0.00	0.00	-0.11	-0.02	0.00	0.00	0.08	0.16	-0.02	0.00	0.08	0.00	0.00	-0.04	0.67	3.23	1.69	Stable	10.00
TMEM236	0.35	0.44	0.00	0.33	0.62	0.60	0.77	0.20	0.33	0.84	0.55	0.39	0.66	0.53	0.24	0.20	0.15	0.68	-0.55	Stable	10.00
NSUN6	-0.24	-0.12	-0.24	-0.24	-0.12	-0.24	-0.27	-0.24	-0.24	-0.15	-0.24	-0.27	-0.27	-0.27	-0.27	-0.08	0.29	1.17	0.23	Stable	10.00
ACBD5	-0.49	-0.37	-0.36	0.00	-0.39	-0.28	-0.56	-0.52	-0.45	-0.38	-0.32	-0.46	-0.12	-0.48	-0.48	-0.28	0.01	1.65	0.72	Stable	10.00
ENSSSCG00000011068	-0.15	-0.04	-0.16	-0.23	-0.20	-0.25	-0.16	-0.24	-0.16	-0.18	-0.36	-0.29	-0.35	-0.34	-0.19	-0.08	0.17	0.74	-0.44	Stable	10.00
MSRB2	-0.80	-0.24	-0.11	-0.64	-0.24	-0.62	-1.19	-0.13	0.18	0.21	-0.84	-1.21	-1.01	-0.97	-1.33	-0.28	0.98	0.99	-0.02	Stable	10.00
NRP1	-0.19	-0.19	-0.19	-0.39	-0.36	0.00	-0.39	-0.42	-0.22	-0.39	-0.39	-0.19	-0.39	0.00	0.00	-0.36	0.16	0.65	-0.62	Stable	10.00

PARD3	-0.24	0.10	-0.22	-0.32	-0.56	-0.27	-0.46	-0.35	-0.23	-0.37	-0.34	-0.31	-0.22	-0.08	-0.18	-0.63	0.05	0.55	-0.86	Stable	10.00
NUDT5	-0.04	0.00	-0.18	0.00	0.40	0.29	0.02	-0.21	0.39	0.59	0.00	-0.01	0.02	-0.49	-0.19	0.44	0.03	-0.41	-1.27	Down	10.00
ECHDC3	0.33	0.03	0.40	-0.05	0.42	0.39	-0.03	0.89	0.31	0.64	-0.01	-0.17	0.14	0.44	0.57	0.15	0.23	1.93	0.95	Stable	10.00
ENSSSCG00000011121	-0.11	-0.08	-0.23	-0.08	-0.19	0.16	-0.21	-0.37	-0.45	-0.24	-0.08	0.04	-0.08	-0.21	-0.08	-0.38	0.16	2.08	1.05	Stable	10.00
ITIH5	-0.25	-0.18	0.36	-0.03	0.14	-0.19	0.10	-0.46	-0.88	-1.08	-0.12	0.41	-0.22	-0.48	-0.34	-0.32	0.66	1.51	0.60	Stable	10.00
TASOR2	0.28	-0.04	-0.26	-0.30	-0.34	0.00	0.03	0.40	0.06	0.16	-0.23	0.00	-0.24	-0.10	0.09	-0.09	0.08	-0.44	-1.19	Stable	10.00
GDI2	-0.41	0.06	-0.25	-0.28	0.25	-0.32	-0.06	0.10	-0.08	-0.27	-0.44	-0.06	-0.23	-0.31	-0.37	0.16	0.90	1.10	0.13	Stable	10.00
CALML5	-0.52	-1.15	-0.59	0.47	0.36	0.21	0.58	-0.93	-0.46	-0.13	-0.09	0.29	-0.06	-0.18	-1.67	0.18	0.01	-4.02	2.01	Up	10.00
ASB13	-0.96	-1.10	-0.62	-0.78	-0.44	0.03	-0.19	-1.29	-0.76	-0.32	-0.60	0.00	-0.85	-0.16	-1.61	-0.74	0.11	1.82	0.86	Stable	10.00
PITRM1	-0.99	-1.30	-1.21	-0.94	-0.97	-1.48	-0.62	-0.39	-1.51	-1.42	-1.15	-0.88	-0.83	-0.92	-0.13	-0.97	0.32	0.82	-0.29	Stable	10.00
ZNF782	0.42	0.21	0.15	0.21	0.14	0.21	0.03	0.14	0.28	0.01	0.21	0.34	0.47	0.21	0.07	0.10	0.75	0.90	-0.16	Stable	10.00
PRXL2C	-0.58	-0.23	-0.26	-0.23	-0.13	-0.23	-0.11	-0.27	-0.26	-0.15	-0.22	-0.31	-0.28	-0.23	-0.13	-0.13	0.42	1.22	0.29	Stable	10.00
NEK11	-0.78	-0.62	-0.80	-0.50	-0.28	-0.36	-0.84	-0.49	-0.66	-0.28	-0.28	-0.28	-0.28	-0.49	-0.30	-0.48	0.00	1.82	0.86	Stable	13.00
ASTE1	0.07	0.20	0.05	-1.05	-0.38	-0.06	0.20	0.03	-0.12	-0.38	-0.38	-0.38	-0.38	0.36	-0.31	-0.27	0.00	-0.14	-2.80	Down	13.00
COL6A5	0.11	0.42	0.46	0.58	0.59	0.34	0.41	0.56	0.71	0.65	0.59	0.59	0.53	0.37	0.54	0.52	0.17	0.81	-0.30	Stable	13.00
METTL6	0.22	0.09	0.09	-0.34	0.09	-0.02	0.22	0.06	0.36	-0.22	0.09	0.09	-0.28	-0.08	-0.17	0.09	0.09	-1.51	0.59	Stable	13.00
HACL1	-0.33	-0.30	-0.20	0.46	-0.15	0.06	-0.47	-0.89	-0.73	-0.15	-0.15	-0.15	0.09	-0.59	0.27	-0.31	0.03	11.07	3.47	Up	13.00
BTD	-0.21	0.00	0.00	-0.67	0.00	-0.31	-0.21	-0.34	-0.18	-0.04	0.00	0.00	-0.29	-0.48	-0.25	0.00	0.67	1.28	0.35	Stable	13.00
OXNAD1	0.16	0.22	0.20	-0.01	0.20	0.09	0.19	0.44	0.46	0.36	0.20	0.20	0.17	0.01	-0.07	0.20	0.76	1.13	0.18	Stable	13.00
DCLK3	-0.06	0.12	0.08	0.15	-0.12	-0.36	-0.06	0.32	-0.12	0.01	0.33	0.19	0.30	-0.13	-0.06	-0.13	0.75	0.29	-1.79	Stable	13.00
OXSR1	0.07	0.14	0.18	0.01	0.01	0.17	0.07	0.22	0.10	0.03	0.35	0.18	0.19	0.22	0.07	0.21	0.86	0.94	-0.09	Stable	13.00
XYLB	0.04	-0.28	-0.60	-0.36	0.03	-0.08	0.07	-0.65	-0.35	-0.20	-0.67	-0.24	-0.53	-0.08	0.07	-0.37	0.54	0.73	-0.45	Stable	13.00
ACVR2B	0.10	-0.12	-0.14	-0.14	-0.17	-0.30	0.10	-0.18	-0.25	-0.10	-0.23	-0.04	-0.15	0.09	0.10	-0.42	0.04	0.19	-2.39	Down	13.00
WDR48	0.03	0.12	0.03	-0.15	-0.16	-0.24	0.03	0.08	0.24	-0.15	0.05	0.04	0.05	-0.32	0.03	0.16	0.31	-0.61	-0.71	Stable	13.00
TTC21A	0.63	0.21	0.12	-0.44	0.32	0.02	0.64	-0.21	-0.83	-0.27	-0.64	-0.14	-0.01	0.10	0.64	-0.54	0.10	-0.76	-0.40	Stable	13.00
CCK	-0.27	-0.05	-0.08	0.17	0.29	0.65	-0.27	-0.05	-0.08	-0.05	-0.09	0.01	0.22	-0.06	-0.27	0.08	0.01	-0.90	-0.16	Stable	13.00
KLHL40	0.04	0.21	0.58	0.75	0.14	0.33	0.04	0.00	0.61	0.35	0.35	0.40	0.74	0.40	0.04	1.34	0.08	0.44	-1.19	Stable	13.00
ZNF662	-0.56	-0.20	-0.10	-0.07	-0.58	-0.49	-0.56	-0.23	-0.26	-0.40	-0.17	-0.35	-0.37	-0.40	-0.56	-0.24	0.79	1.07	0.10	Stable	13.00
GASK1A	-0.05	0.10	-0.05	0.43	-0.07	-0.09	-0.05	0.05	0.24	-0.03	0.14	0.40	0.50	0.13	-0.05	0.39	0.11	0.18	-2.47	Stable	13.00
ANO10	-0.54	0.00	-0.39	0.12	-0.18	0.09	-0.53	-0.12	-0.24	-0.37	-0.27	-0.19	-0.15	-0.31	-0.54	0.01	0.04	2.84	1.51	Up	13.00
ZNF502	-0.03	-0.11	-0.10	-0.11	-0.03	-0.07	-0.03	-0.11	-0.11	-0.11	-0.07	-0.03	-0.11	-0.03	-0.03	-0.06	0.85	0.95	-0.07	Stable	13.00
CCDC12	0.00	0.21	-0.11	-0.21	0.06	0.23	0.00	0.21	0.00	-0.11	0.00	-0.11	-0.23	0.00	0.00	-0.25	0.13	-0.52	-0.96	Stable	13.00
NBEAL2	0.00	0.00	0.00	0.00	-0.01	-0.01	0.00	0.00	0.00	0.00	0.00	0.00	0.00	0.00	0.00	0.00	0.17	0.00	#NAME?	Stable	13.00
DHX30	0.00	-0.19	-0.03	-0.20	0.00	-0.19	0.00	-0.12	0.00	-0.13	-0.03	-0.09	-0.11	0.00	0.00	-0.05	0.12	0.41	-1.27	Stable	13.00
NME6	0.15	0.48	0.46	0.77	0.26	0.58	0.15	0.21	0.10	0.55	0.47	0.34	0.66	0.15	0.15	0.99	0.00	0.40	-1.31	Down	13.00
ATRIP	0.62	0.18	0.10	0.31	0.15	0.28	0.62	0.08	0.00	0.04	0.00	0.49	0.42	0.62	0.62	0.23	0.36	1.47	0.56	Stable	13.00
BSN	-0.04	-0.17	-0.19	-0.19	0.00	-0.09	-0.04	-0.17	0.00	-0.14	-0.12	-0.10	-0.25	-0.04	-0.04	-0.35	0.15	0.54	-0.88	Stable	13.00
MST1	-0.14	-0.24	-0.14	-0.13	-0.07	-0.14	-0.14	-0.36	-0.45	-0.16	-0.52	-0.20	-0.20	-0.14	-0.14	-0.06	0.61	1.19	0.25	Stable	13.00
RNF123	0.08	0.08	-0.09	-0.17	0.08	-0.01	0.08	0.07	0.00	-0.29	-0.09	0.05	-0.04	0.08	0.08	-0.18	0.03	-0.58	-0.78	Stable	13.00
IFRD2	-0.39	0.06	-0.30	-0.26	0.01	-0.05	-0.39	-0.01	-0.12	-0.27	-0.36	-0.08	-0.25	-0.39	-0.39	-0.54	0.85	1.08	0.11	Stable	13.00
HYAL1	-0.11	-0.07	-0.28	0.10	0.04	0.16	-0.11	-0.13	-0.29	-0.25	-0.06	0.13	-0.12	-0.11	-0.11	-0.40	0.21	3.01	1.59	Stable	13.00
DOCK3	-0.35	-0.21	-0.07	0.12	-0.15	0.01	-0.35	-0.18	-0.35	-0.09	-0.10	-0.14	0.00	-0.35	-0.35	0.03	0.00	6.97	2.80	Up	13.00
PHF7	0.00	0.00	0.00	0.00	0.00	0.00	0.00	0.00	0.00	0.00	0.00	0.00	0.00	0.00	0.00	0.00	0.35	0.00	#NAME?	Stable	13.00
NEK4	0.40	0.19	0.20	0.15	0.28	0.19	0.40	0.19	0.40	0.29	0.29	0.17	0.15	0.40	0.40	0.17	0.03	1.54	0.62	Stable	13.00
GLT8D1	0.97	-0.28	-0.13	0.25	0.30	-0.07	0.97	-0.35	1.20	0.73	0.52	0.51	-0.21	0.97	0.97	0.28	0.36	1.87	0.90	Stable	13.00

SLC25A26	0.00	-0.01	0.00	-0.12	0.00	-0.06	0.00	-0.06	-0.01	-0.01	0.00	-0.11	-0.12	-0.06	-0.01	-0.01	0.14	0.32	-1.63	Stable	13.00
SHQ1	-0.66	0.08	0.03	0.20	0.34	0.29	-0.56	-0.39	0.37	0.44	0.50	-0.62	0.30	0.29	-0.57	0.36	0.06	-0.78	-0.35	Stable	13.00
CNTN3	-0.54	-0.25	-0.18	-0.43	-0.43	-0.37	-0.51	-0.18	-0.43	-0.43	-0.37	-0.47	-0.37	-0.43	-0.47	-0.43	0.53	0.91	-0.13	Stable	13.00
TTLL3	0.75	-0.07	0.56	0.45	0.17	0.34	0.88	0.51	0.58	0.44	0.28	0.54	0.38	0.60	0.60	0.45	0.15	1.44	0.53	Stable	13.00
RPUSD3	-0.51	0.02	0.11	0.01	0.36	0.16	-0.29	-0.04	0.15	0.09	0.26	-0.47	0.10	-0.52	-0.62	0.39	0.04	-1.89	0.92	Stable	13.00
CIDEC	0.37	-0.15	0.48	0.50	0.15	0.53	0.40	0.05	0.27	0.31	0.27	0.67	0.61	0.79	0.52	0.66	0.34	0.73	-0.45	Stable	13.00
CRELD1	0.60	0.19	0.40	0.45	0.95	0.79	0.70	0.46	0.36	0.41	0.53	0.75	0.39	0.79	0.62	0.74	0.30	0.82	-0.28	Stable	13.00
FANCD2	0.07	-0.08	0.10	0.03	-0.18	0.01	0.07	0.03	0.07	0.13	0.07	0.14	0.18	0.16	0.23	0.07	0.65	1.41	0.50	Stable	13.00
BRK1	1.06	0.67	0.60	0.59	1.01	0.75	0.50	0.73	0.76	0.77	0.84	0.57	0.63	0.13	0.72	0.70	0.43	0.88	-0.18	Stable	13.00
VHL	-0.64	-0.38	-0.24	-0.28	-0.82	-0.52	-0.60	-0.51	-0.88	-0.99	-0.88	-0.24	-0.29	-0.35	-0.85	-0.60	0.87	0.96	-0.06	Stable	13.00
ENSSSCG00000011569	-0.49	-0.49	-0.49	-0.49	-0.25	-0.49	-0.49	-0.49	-0.25	-0.25	-0.25	-0.49	-0.25	-0.49	-0.25	-0.25	0.15	1.27	0.34	Stable	13.00
SEC61A1	-0.05	-0.25	-0.05	0.07	-0.19	-0.15	0.00	-0.13	0.07	0.08	-0.02	0.20	0.26	-0.23	0.18	0.00	0.27	-1.80	0.85	Stable	13.00
CHST13	0.04	-0.65	-0.15	-0.38	-0.82	-0.30	-0.08	-0.68	-0.93	-0.72	-0.52	0.54	-0.04	0.31	1.14	-0.45	0.46	0.37	-1.42	Stable	13.00
TF	0.28	0.62	0.37	0.45	0.23	0.80	0.47	-0.06	0.13	0.17	0.52	0.48	0.27	-0.01	0.16	0.07	0.29	0.66	-0.61	Stable	13.00
AMOTL2	0.00	0.00	0.00	0.00	0.00	0.00	0.00	0.00	0.00	0.00	0.00	0.00	0.00	-0.17	0.00	0.00	0.35	#NAME?	Inf	Stable	13.00
RBP2	0.00	0.03	-0.17	-0.04	-0.01	-0.28	0.00	-0.12	-0.03	-0.14	-0.03	-0.01	-0.28	0.02	-0.01	-0.28	0.09	0.27	-1.90	Stable	13.00
GRK7	0.17	-0.68	-0.33	-0.37	-0.37	-0.50	0.13	-0.55	-0.47	-0.41	-0.43	-0.42	-1.06	-0.12	0.26	-0.41	0.07	0.40	-1.31	Stable	13.00
P2RY14	0.30	0.44	0.32	0.21	0.17	0.05	0.22	0.27	-0.04	-0.04	0.14	0.39	0.25	0.25	0.10	0.00	0.24	1.60	0.68	Stable	13.00
PTX3	0.18	0.00	0.13	0.00	0.00	0.16	0.13	0.00	0.00	0.00	0.00	0.00	0.00	0.00	0.14	0.00	0.16	3.56	1.83	Stable	13.00
ENSSSCG00000011764	0.00	0.15	0.14	0.00	0.14	0.00	0.00	0.00	0.14	0.00	0.14	0.00	0.00	0.00	0.00	0.27	0.74	0.78	-0.36	Stable	13.00
KLHL24	0.18	0.13	0.13	0.04	0.21	0.22	0.11	0.13	0.45	0.33	0.03	0.54	0.32	0.28	0.16	0.41	0.39	0.75	-0.41	Stable	13.00
YEATS2	-0.17	-0.12	-0.09	-0.08	-0.26	-0.27	0.04	-0.12	-0.25	-0.24	-0.36	-0.26	-0.11	-0.42	-0.37	-0.43	0.35	0.74	-0.43	Stable	13.00
EIF2B5	-0.23	0.04	0.05	0.19	0.05	0.04	-0.55	0.04	0.15	0.08	0.08	0.22	0.28	0.11	0.14	0.08	0.12	-0.24	-2.06	Stable	13.00
RFC4	-0.41	-0.24	-0.10	0.48	0.34	-0.11	-0.74	-0.07	0.21	0.36	0.22	-0.12	0.38	-0.14	-0.14	0.05	0.01	-1.01	0.02	Stable	13.00
TMEM44	-0.28	-0.37	-0.37	0.08	0.07	-0.15	-0.21	0.02	-0.04	0.02	0.17	0.00	0.10	-0.37	-0.36	-0.36	0.01	22.89	4.52	Up	13.00
LSG1	-0.39	-0.70	-0.51	0.10	-0.11	-0.48	-0.36	0.26	0.46	0.46	0.23	-0.17	0.34	-0.80	0.49	-1.09	0.69	2.16	1.11	Stable	13.00
TNK2	0.07	0.07	0.03	0.07	0.13	0.03	0.07	0.00	0.00	0.00	0.07	0.07	0.03	0.07	0.13	0.07	0.84	0.93	-0.11	Stable	13.00
ENSSSCG00000011859	0.28	0.14	0.14	0.10	0.82	0.10	0.27	0.33	0.17	0.10	0.41	0.11	0.30	0.14	0.11	0.14	0.52	0.75	-0.41	Stable	13.00
UMPS	0.20	0.24	0.24	-0.04	0.17	0.31	0.14	0.03	0.31	0.28	0.14	0.12	0.19	0.24	0.12	0.24	0.80	1.07	0.10	Stable	13.00
DRD3	0.17	0.14	-0.24	0.19	0.02	0.32	0.17	-0.29	0.25	0.12	-0.19	0.04	0.00	-0.23	-0.21	0.46	0.18	-0.26	-1.94	Stable	13.00
CCDC191	-0.41	-0.04	-0.46	-0.25	-0.12	-0.09	-0.41	-0.16	-0.33	-0.50	-0.12	-0.51	-0.28	-0.40	-0.53	-0.08	0.26	1.41	0.50	Stable	13.00
ZDHHC23	0.54	-0.02	0.03	0.10	-0.35	-0.15	0.54	-0.17	-0.28	-0.02	-0.56	-0.43	-0.19	0.32	0.27	-0.25	0.01	-0.67	-0.58	Stable	13.00
GRAMD1C	-0.04	0.15	0.87	-0.09	-0.31	-0.18	-0.04	-0.14	0.39	0.24	0.04	0.01	-0.16	0.72	0.58	-0.01	0.03	-5.37	2.42	Up	13.00
BTG3	0.04	0.04	-0.23	0.41	0.01	0.21	0.04	0.04	0.04	0.30	-0.04	-0.02	0.21	0.03	-0.30	0.21	0.02	-0.22	-2.18	Down	13.00
ATP5PO	0.01	-0.78	-0.53	0.55	-0.13	0.50	0.08	0.30	0.41	0.72	0.44	0.17	0.48	0.44	-0.01	0.17	0.07	-0.03	-5.27	Stable	13.00
ENSSSCG00000012061	0.00	0.00	0.00	0.00	0.00	0.00	0.00	-0.05	-0.05	-0.05	0.00	0.00	0.00	0.05	0.00	0.00	0.97	1.07	0.10	Stable	13.00
VPS26C	-0.13	-0.13	-0.13	-0.12	-0.25	-0.25	-0.13	-0.02	-0.12	-0.02	-0.25	-0.25	-0.13	-0.09	-0.25	-0.25	0.10	0.65	-0.63	Stable	13.00
ENSSSCG00000012071	0.70	1.01	1.07	0.82	1.00	0.55	0.74	0.46	0.39	0.74	0.95	1.00	0.95	1.00	0.92	0.81	0.55	0.92	-0.12	Stable	13.00
DSCAM	-0.34	-0.57	-0.17	-0.39	-0.22	-0.31	0.09	-0.35	-0.44	-0.32	-0.32	0.08	-0.16	0.14	-0.19	0.04	0.79	1.15	0.20	Stable	13.00
ADARB1	-0.13	-0.05	-0.11	-0.24	0.06	-0.14	-0.12	-0.14	-0.16	-0.12	-0.05	-0.24	-0.24	-0.24	-0.05	-0.24	0.58	0.83	-0.27	Stable	13.00
SERAC1	-0.07	-0.14	-0.48	-0.21	-0.17	-0.15	0.15	-0.31	-0.07	0.12	-0.11	-0.26	-0.14	-0.28	-0.13	-0.16	0.68	1.25	0.33	Stable	1.00
SYNJ2	-0.05	-0.56	-0.07	-0.31	-0.06	-0.23	-0.56	-0.25	-0.51	-0.47	-0.09	-0.15	-0.38	-0.11	-0.34	-0.41	0.64	1.17	0.22	Stable	1.00
AP2A2	-0.14	0.03	-0.02	0.11	-0.14	0.02	0.08	-0.26	-0.13	0.15	0.31	0.11	0.00	-0.02	0.00	-0.08	0.09	-0.98	-0.02	Stable	2.00
IRF7	-0.17	0.17	0.14	0.10	0.31	0.28	0.15	0.20	0.15	0.10	0.10	0.16	0.09	0.00	0.09	0.13	0.23	0.57	-0.80	Stable	2.00
ENSSSCG00000012878	0.00	-0.03	-0.02	-0.01	-0.02	-0.02	-0.02	-0.06	-0.06	-0.02	-0.01	-0.02	-0.05	-0.01	-0.04	-0.03	0.34	1.42	0.51	Stable	2.00

ENSSSCG00000012879	-0.02	-0.05	-0.42	-0.49	-0.31	0.11	0.10	-0.02	-0.04	-0.02	0.15	-0.36	-0.25	0.14	-0.05	-0.30	0.19	0.24	-2.05	Stable	2.00
TESMIN	-0.10	-0.12	0.29	0.26	0.33	-0.06	-0.06	-0.25	-0.11	-0.10	-0.13	0.29	0.26	-0.12	-0.13	0.39	0.03	-0.48	-1.04	Down	2.00
CHKA	-0.38	-0.39	-0.02	-0.02	-0.06	0.32	0.16	-0.19	-0.11	-0.05	-0.31	0.27	-0.17	0.00	0.12	0.06	0.33	-19.83	4.31	Stable	2.00
TMEM134	0.02	0.06	-0.69	-0.25	-0.03	-0.02	0.03	-0.12	0.29	-0.08	0.14	-0.09	-0.03	0.13	0.14	-0.20	0.65	0.25	-1.98	Stable	2.00
PPP1CA	0.20	0.20	0.22	0.26	0.14	0.26	0.31	0.00	0.21	0.17	0.35	0.44	0.38	0.30	0.07	0.16	0.16	0.70	-0.51	Stable	2.00
PC	-0.29	-0.21	-0.07	-0.47	-0.28	-0.01	0.20	-0.38	-0.38	0.15	-0.27	-0.14	-0.16	-0.18	-0.11	-0.21	0.96	1.03	0.04	Stable	2.00
PELI3	-0.18	-0.14	0.04	-0.15	0.00	-0.16	0.00	0.00	-0.18	0.00	-0.23	0.00	-0.19	-0.08	-0.14	0.00	0.91	0.94	-0.09	Stable	2.00
PACS1	0.51	0.49	0.02	1.19	0.00	0.74	0.23	-0.01	-0.22	0.23	1.25	0.08	0.12	0.64	-0.07	-0.03	0.28	0.44	-1.17	Stable	2.00
EFEMP2	-0.59	-0.59	-0.29	-0.59	0.00	0.00	-0.59	0.00	0.00	-0.59	0.00	-0.29	-0.29	-0.59	0.00	-0.59	0.80	1.13	0.17	Stable	2.00
AP5B1	0.06	0.12	-0.09	0.19	-0.21	-0.15	-0.06	-0.25	0.08	-0.06	0.05	-0.07	-0.13	0.14	-0.09	-0.06	0.53	0.22	-2.21	Stable	2.00
FRMD8	0.01	0.00	0.00	0.37	-0.38	-0.68	-0.73	-0.09	-0.28	-0.73	0.42	-0.34	-0.76	0.05	0.23	-0.41	0.30	0.32	-1.62	Stable	2.00
SLC25A45	-0.35	-0.36	-0.49	-0.52	0.12	-0.15	-0.26	0.09	0.19	-0.26	-0.40	-0.07	0.02	-0.41	0.30	-0.07	0.97	0.97	-0.05	Stable	2.00
TIGD3	0.18	0.18	0.01	0.33	-0.01	0.01	0.04	-0.01	0.06	0.04	0.18	0.03	0.01	0.18	0.33	-0.01	0.43	1.67	0.74	Stable	2.00
CAPN1	0.50	0.48	0.05	-0.34	0.09	0.96	0.92	-0.57	0.04	0.92	0.35	0.22	0.39	0.36	0.08	0.76	0.41	0.55	-0.85	Stable	2.00
MRPL49	-0.02	-0.17	-0.28	0.23	-0.18	-0.16	-0.19	-0.15	-0.12	-0.19	0.04	-0.16	-0.21	-0.25	-0.11	-0.18	0.36	1.58	0.66	Stable	2.00
NAALADL1	0.65	0.30	0.57	0.09	0.63	0.92	0.59	0.45	0.37	0.59	0.10	0.66	0.86	0.06	0.35	0.62	0.30	0.75	-0.42	Stable	2.00
SAC3D1	0.09	0.04	0.11	0.33	0.27	0.15	-0.26	0.30	0.44	-0.26	0.33	0.16	0.02	-0.07	0.42	0.02	0.96	1.04	0.06	Stable	2.00
ENSSSCG00000013038	-0.05	0.21	-0.04	-0.18	-0.07	0.01	0.37	-0.15	0.10	0.37	0.11	-0.12	-0.03	0.09	-0.43	0.00	1.00	1.01	0.02	Stable	2.00
NUDT22	0.62	0.31	0.75	0.53	0.03	-0.10	0.57	-0.06	-0.17	0.57	-0.10	-0.04	0.00	0.61	0.00	0.02	0.20	2.88	1.52	Stable	2.00
TRPT1	1.18	1.05	1.15	1.18	0.05	0.52	1.19	1.47	1.19	1.19	1.34	0.65	0.39	1.27	0.57	0.40	0.05	1.58	0.66	Stable	2.00
LGALS12	0.90	0.59	0.72	0.41	0.23	0.19	1.24	1.24	0.69	1.24	-0.24	0.97	0.71	0.90	0.03	0.10	0.15	1.75	0.81	Stable	2.00
FADS2	-0.61	0.27	-0.30	1.15	1.17	0.68	-0.52	-0.55	-0.54	-0.52	0.54	-0.22	-0.23	0.02	0.38	0.44	0.04	-0.62	-0.70	Stable	2.00
CYB561A3	0.84	0.00	0.76	-0.30	-0.34	0.40	0.84	0.79	0.79	0.84	-0.16	0.38	0.66	0.19	0.37	0.24	0.09	2.66	1.41	Stable	2.00
MS4A7	0.29	-0.23	-0.87	-0.73	-0.31	-0.44	0.16	-1.21	-1.18	1.37	-0.13	-0.14	-0.52	0.43	0.06	0.11	0.51	3.21	1.68	Stable	2.00
OSBP	0.37	0.24	0.65	0.51	0.24	0.48	0.52	0.80	0.71	0.01	0.22	0.60	0.48	0.49	0.36	0.50	0.19	1.36	0.44	Stable	2.00
GLYATL2	-1.13	-0.15	-0.56	-0.37	0.00	-0.90	-1.09	-0.97	-0.55	-1.78	-0.54	-1.17	-0.81	-1.44	-0.67	-0.67	0.87	1.05	0.07	Stable	2.00
ENSSSCG00000013176	-0.09	-0.18	-0.30	-0.60	-0.31	-0.03	-0.11	-0.11	-0.21	0.20	-0.04	-0.05	-0.04	0.05	-0.05	-0.13	0.99	1.00	0.01	Stable	2.00
SERPING1	-0.13	-0.13	-0.13	-0.13	-0.13	-0.12	-0.13	-0.13	-0.13	0.12	-0.13	-0.12	-0.13	-0.13	-0.13	-0.12	0.26	1.40	0.48	Stable	2.00
PTPMT1	0.10	0.46	0.00	0.78	1.41	1.41	-0.01	-0.05	-0.11	0.16	0.42	0.03	0.60	-0.08	-0.08	1.41	0.01	0.04	-4.81	Down	2.00
PACSIN3	-0.08	0.12	0.07	-0.04	0.00	-0.04	-0.03	0.08	-0.03	-0.22	0.12	-0.14	-0.12	0.05	-0.08	0.00	0.16	-0.25	-1.98	Stable	2.00
C11orf49	-0.09	0.00	0.00	0.00	0.00	0.00	-0.10	-0.21	-0.01	-0.20	0.00	0.00	0.00	0.00	0.00	0.00	0.47	2.11	1.08	Stable	2.00
ATG13	0.27	0.00	0.05	-0.25	-0.24	-0.24	0.13	0.22	0.03	-0.02	-0.11	0.04	-0.10	0.05	0.07	-0.24	0.00	-0.70	-0.52	Stable	2.00
CREB3L1	-0.21	-0.16	-0.19	-0.13	0.06	0.17	-0.22	-0.13	-0.21	-0.02	0.03	-0.33	-0.13	-0.17	-0.17	0.06	0.03	5.05	2.34	Up	2.00
MAPK8IP1	0.19	0.00	0.40	0.03	0.00	0.00	0.13	0.04	0.23	-0.04	0.06	0.34	0.17	0.23	0.17	0.00	0.12	2.47	1.30	Stable	2.00
TSPAN18	0.07	0.11	-0.10	-0.19	-0.10	-0.10	-0.13	0.12	0.12	-0.01	-0.14	-0.11	-0.24	0.02	0.06	0.06	0.01	-0.33	-1.61	Down	2.00
ACCS	-0.33	-0.24	-0.22	-0.52	-0.52	-0.57	-0.46	-0.02	-0.63	-0.78	-0.52	-0.57	-0.57	-0.14	-0.05	-0.03	0.03	0.51	-0.97	Stable	2.00
COMMD9	0.01	-0.18	-0.18	0.01	0.01	0.01	-0.26	0.00	0.01	0.01	0.01	-0.13	-0.25	-0.18	-0.31	0.01	0.11	3.28	1.71	Stable	2.00
SLC1A2	0.19	0.00	0.28	0.00	0.00	0.00	0.19	0.36	0.00	0.00	0.00	0.12	0.00	0.28	0.00	0.27	0.09	3.36	1.75	Stable	2.00
APIP	0.00	0.01	0.00	-0.15	-0.36	-0.22	0.15	-0.42	-0.27	-0.32	-0.40	-0.33	-0.29	-0.06	0.11	-0.23	0.01	0.21	-2.28	Down	2.00
CAT	-0.11	-0.06	-0.07	-0.09	-0.31	-0.40	-0.02	0.39	-0.41	-0.69	-0.48	0.20	-0.40	-0.08	-0.12	0.09	0.15	0.23	-2.14	Stable	2.00
NAT10	-0.26	-0.59	-0.12	-0.61	-0.60	-0.59	-0.26	0.00	-0.59	-0.57	-0.57	0.00	-0.57	-0.26	-0.14	-0.19	0.12	0.61	-0.72	Stable	2.00
LMO2	-0.77	-0.47	0.73	0.02	-0.14	-0.32	-0.48	1.10	0.35	0.01	0.14	0.99	-0.25	0.62	-0.56	0.72	0.79	0.44	-1.19	Stable	2.00
KIAA1549L	-0.11	-0.25	0.00	-0.25	-0.28	-0.28	-0.10	0.00	-0.12	-0.15	-0.11	0.00	-0.28	0.00	-0.11	-0.05	0.09	0.49	-1.04	Stable	2.00
PRRG4	-0.30	-0.74	0.11	-0.74	-0.13	-0.29	-0.20	0.11	-0.34	0.11	-0.71	0.11	-0.16	0.19	-0.08	-0.25	0.54	0.61	-0.71	Stable	2.00
EIF3M	0.19	0.35	-0.30	0.35	0.19	0.10	0.20	-0.30	0.04	-0.35	0.47	-0.30	0.20	0.06	0.12	0.05	0.74	0.50	-0.99	Stable	2.00

WT1	-0.05	-0.12	0.00	-0.12	-0.14	-0.14	0.00	0.00	0.00	-0.08	0.18	0.00	-0.16	-0.02	-0.06	-0.06	0.42	0.45	-1.14	Stable	2.00
ENSSSCG00000013318	-0.23	-0.50	0.00	-0.50	-0.69	-0.50	-0.25	0.00	-0.12	-0.02	-0.40	0.00	-0.50	-0.12	-0.13	-0.25	0.09	0.47	-1.09	Stable	2.00
LGR4	-0.11	-0.26	-0.13	-0.24	-0.26	-0.26	-0.07	-0.26	-0.13	-0.11	-0.26	-0.13	-0.26	-0.26	-0.09	-0.26	0.13	0.74	-0.43	Stable	2.00
ANO3	-0.40	-0.93	0.35	-0.55	-0.49	-0.44	0.14	-0.44	-0.38	-0.47	-0.74	-0.39	-0.68	-0.55	-0.38	-0.44	0.21	0.61	-0.70	Stable	2.00
PRMT3	0.21	0.00	0.48	0.17	0.12	0.19	0.24	0.00	0.48	0.15	0.00	0.22	0.00	0.20	-0.02	0.48	0.73	1.19	0.25	Stable	2.00
HTATIP2	-0.74	-1.20	-0.34	-0.78	-0.47	-0.66	-0.74	-0.12	-0.34	-0.70	-0.86	-0.31	-1.12	-0.65	-0.41	-0.34	0.58	0.87	-0.20	Stable	2.00
UEVLD	-0.32	-0.24	-0.08	-0.15	-0.24	-0.46	-0.29	-0.08	-0.15	-0.26	-0.16	0.04	-0.28	-0.16	-0.16	-0.12	0.78	0.91	-0.13	Stable	2.00
PLEKHA7	0.07	0.13	0.05	0.05	0.06	-0.01	0.06	-0.10	-0.11	0.01	0.01	-0.11	0.06	0.05	0.02	-0.05	0.64	5.76	2.53	Stable	2.00
SBF2	0.27	0.54	0.58	0.47	0.46	-0.46	0.48	0.37	0.41	0.58	0.01	0.41	0.53	0.45	0.43	0.44	0.32	1.45	0.53	Stable	2.00
MED16	0.36	0.36	0.31	0.31	0.39	0.47	0.26	0.52	0.47	0.47	0.52	0.52	0.32	0.44	0.44	0.36	0.56	0.94	-0.09	Stable	2.00
IZUMO4	-0.14	0.00	-0.15	0.11	0.07	-0.16	-0.14	-0.11	0.03	-0.06	-0.01	0.09	0.20	-0.06	-0.09	-0.19	0.13	-13.33	3.74	Stable	2.00
DOT1L	0.25	0.25	0.34	0.25	0.33	0.33	0.13	0.25	0.25	0.25	0.25	0.25	0.25	0.25	0.25	0.25	0.34	0.91	-0.14	Stable	2.00
OAZ1	0.43	0.43	0.40	0.43	0.40	0.40	0.07	0.43	0.43	0.43	0.43	0.43	0.40	0.43	0.43	0.43	0.45	0.91	-0.13	Stable	2.00
ENSSSCG00000013471	0.34	0.05	0.02	0.30	0.02	0.33	0.32	0.76	0.76	0.70	0.34	0.91	0.03	0.33	0.28	0.30	0.96	0.98	-0.03	Stable	2.00
DOHH	0.65	0.49	0.28	0.54	0.29	0.45	0.29	0.58	0.60	0.52	0.55	0.65	0.28	0.53	0.51	0.51	0.80	1.04	0.05	Stable	2.00
ANKRD24	-0.01	0.13	0.07	0.07	0.07	0.07	-0.08	-0.16	-0.16	0.07	-0.01	-0.36	-0.01	-0.08	-0.08	-0.01	0.63	2.78	1.48	Stable	2.00
MPND	0.07	-0.02	0.18	-0.05	0.09	-0.10	-0.04	-0.04	0.15	-0.02	0.16	-0.05	-0.03	-0.03	-0.03	-0.02	0.46	-24.91	4.64	Stable	2.00
SH3GL1	-0.29	-0.39	0.05	-0.43	-0.09	-0.13	-0.18	-0.35	-0.32	-0.29	-0.20	-0.61	-0.22	-0.37	-0.38	-0.40	0.81	0.93	-0.10	Stable	2.00
C3	0.00	0.00	0.00	0.00	0.00	0.00	-0.27	-0.27	0.00	0.00	0.00	-0.27	0.00	0.00	0.00	-0.14	0.79	1.30	0.38	Stable	2.00
PEX11G	0.02	0.00	0.02	0.04	-0.01	-0.07	0.41	0.00	0.01	0.08	-0.03	0.07	0.09	0.00	0.00	0.00	0.52	2.72	1.45	Stable	2.00
PNPLA6	-0.07	-0.24	-0.24	-0.21	-0.24	0.09	-0.25	-0.24	-0.05	-0.18	-0.07	-0.21	-0.21	-0.24	-0.24	-0.24	0.47	1.24	0.31	Stable	2.00
RPS28	0.17	0.11	0.21	-0.24	0.21	0.32	0.12	0.11	0.00	-0.55	0.21	0.04	0.18	0.11	0.11	0.11	0.46	3.35	1.74	Stable	2.00
ENSSSCG00000013610	-0.26	-0.51	-0.20	-0.26	-0.41	0.00	-0.05	-0.07	0.11	0.22	-0.22	-0.87	-0.19	-0.21	-0.18	-0.18	0.61	0.72	-0.48	Stable	2.00
DNM2	0.15	0.29	0.30	-0.02	0.32	-0.02	0.34	0.11	-0.14	0.00	0.00	0.38	0.54	0.22	0.11	0.07	0.87	1.10	0.14	Stable	2.00
ICAM4	0.01	0.03	0.01	0.03	0.01	0.00	0.01	0.03	0.01	0.03	0.00	0.00	0.03	0.03	0.03	0.03	0.37	1.33	0.42	Stable	2.00
ENSSSCG00000013665	-0.51	-0.40	0.08	-0.68	-0.43	-0.56	0.61	0.29	-0.74	-0.02	-0.19	0.15	-0.85	-0.37	-0.28	0.17	0.53	0.55	-0.86	Stable	2.00
RDH8	0.18	0.46	0.26	0.46	0.25	0.00	-0.49	-0.21	0.20	0.60	0.17	-0.05	0.28	0.10	0.17	0.12	0.29	0.36	-1.46	Stable	2.00
FBXL12	0.13	0.37	0.16	0.35	0.19	0.00	0.36	0.37	0.18	0.39	0.16	0.23	0.35	0.36	0.38	0.33	0.54	1.15	0.21	Stable	2.00
ENSSSCG00000013715	0.15	0.15	0.01	0.09	-0.01	0.00	0.16	0.16	0.02	0.18	0.03	0.28	0.15	0.20	0.16	0.10	0.61	1.22	0.29	Stable	2.00
WDR83	0.46	1.18	0.52	1.18	0.59	0.00	0.26	1.18	0.59	0.02	0.42	0.34	1.18	1.18	1.18	1.18	0.38	1.33	0.42	Stable	2.00
ENSSSCG00000013723	0.02	0.04	0.02	0.02	0.02	0.00	0.11	0.00	0.00	0.11	0.02	0.21	0.01	0.04	0.02	0.01	0.50	0.61	-0.71	Stable	2.00
ENSSSCG00000013724	0.00	0.00	0.04	0.00	0.05	0.00	0.00	0.00	0.00	0.11	0.00	-0.26	0.00	0.00	0.00	0.00	0.66	-0.33	-1.60	Stable	2.00
BEST2	-0.32	-0.59	-0.55	-0.59	-0.37	-0.06	-0.45	-0.70	-0.32	-0.19	-0.32	-0.65	-0.59	-0.64	-0.64	-0.64	0.31	1.24	0.31	Stable	2.00
CCDC130	-0.42	-0.29	-0.40	-0.29	-0.44	-0.57	-0.24	-0.29	-0.49	-0.36	-0.42	-0.11	-0.52	-0.29	-0.29	-0.29	0.57	0.91	-0.14	Stable	2.00
RFX1	0.00	0.00	0.00	0.00	0.00	0.00	0.00	0.00	0.00	0.00	0.00	0.00	0.00	0.00	0.00	0.00	0.66	1.07	0.10	Stable	2.00
RLN3	0.14	0.16	-0.07	0.16	-0.06	0.17	0.28	0.16	0.19	0.16	0.17	0.23	0.07	0.16	0.16	0.16	0.74	1.12	0.16	Stable	2.00
ENSSSCG00000013785	-0.09	0.11	0.15	0.01	0.01	-0.40	0.15	-0.13	-0.34	-0.13	-0.16	-0.09	-0.03	-0.11	-0.01	-0.01	0.41	0.33	-1.60	Stable	2.00
ZNF333	-0.56	-0.56	-0.32	-0.56	-0.42	-0.49	-0.36	-0.56	-0.49	-0.56	-0.59	-0.47	-0.31	-0.56	-0.56	-0.56	0.98	1.00	0.00	Stable	2.00
CYP4F3	-0.04	-0.03	0.16	0.15	0.19	0.00	0.19	0.23	0.12	0.09	-0.02	0.30	-0.05	0.13	-0.05	0.00	0.93	1.07	0.09	Stable	2.00
CALR3	0.70	0.72	0.69	0.37	0.69	0.56	-0.10	0.00	0.13	0.04	0.44	-0.10	0.36	0.42	0.44	0.24	0.75	1.15	0.20	Stable	2.00
SMIM7	0.37	0.43	0.34	0.23	0.25	0.54	0.26	0.06	-0.25	0.13	0.49	-0.64	0.27	0.29	0.27	0.27	0.87	1.13	0.18	Stable	2.00
SIN3B	0.00	0.00	0.00	0.00	0.00	0.00	0.00	0.13	0.00	0.13	0.00	0.07	0.00	0.07	0.00	0.07	0.74	0.75	-0.41	Stable	2.00
SLC27A1	-0.76	-0.29	-0.17	-0.41	-0.18	-1.23	-0.53	-0.28	-0.42	-0.45	-0.86	-0.26	-0.28	-0.28	-0.28	-0.28	0.43	0.76	-0.40	Stable	2.00
MPV17L2	-0.78	-0.35	-0.43	-0.64	-0.29	-0.14	0.17	-0.61	-0.07	-0.88	-0.17	0.37	-0.69	-0.13	-0.31	-0.80	0.63	0.77	-0.37	Stable	2.00
LPAR2	0.01	0.12	0.05	0.10	0.05	0.00	0.05	0.10	0.09	0.06	0.07	0.04	0.12	0.11	0.10	0.10	0.56	1.17	0.23	Stable	2.00

ZNF672	-0.01	0.53	0.33	0.68	0.14	-0.50	0.14	0.81	-0.20	0.44	0.20	-0.20	0.59	0.89	0.75	0.52	0.42	1.72	0.78	Stable	2.00
COL23A1	0.09	0.10	0.06	0.06	0.06	0.06	0.03	0.09	0.06	0.06	0.09	0.02	0.06	0.06	0.09	0.13	0.86	1.04	0.05	Stable	2.00
B4GALT7	0.13	0.00	-0.31	0.03	0.00	0.08	0.13	0.29	0.16	0.11	0.13	-0.27	0.02	0.14	0.13	0.15	0.51	2.71	1.44	Stable	2.00
TMED9	-0.05	0.10	-0.16	0.14	0.03	0.02	0.03	0.05	0.15	0.18	-0.08	-0.09	0.35	0.07	0.08	0.08	0.46	0.41	-1.30	Stable	2.00
LMAN2	-0.15	0.00	0.00	-0.09	0.00	0.00	-0.24	-0.46	-0.33	-0.01	-0.23	0.00	-0.18	-0.22	-0.09	-0.22	0.19	2.04	1.03	Stable	2.00
FGFR4	-0.32	-0.12	-0.06	-0.12	-0.12	-0.08	-0.23	-0.53	-0.22	-0.13	-0.23	-0.12	-0.38	-0.32	-0.29	-0.25	0.20	1.46	0.55	Stable	2.00
RNF44	0.05	0.19	0.19	0.28	0.05	0.08	0.00	0.00	0.21	0.29	0.09	0.09	0.09	0.10	0.06	0.09	0.45	0.74	-0.44	Stable	2.00
SFXN1	-0.42	-0.38	-0.16	-0.38	-0.34	-0.38	-0.23	-0.46	-0.42	-0.38	-0.42	-0.15	-0.38	-0.38	-0.38	-0.42	0.96	0.99	-0.01	Stable	2.00
UTP15	-0.23	-0.79	-0.42	-1.02	-0.54	-0.68	0.19	-0.64	-1.02	-0.94	-0.73	-0.13	-0.86	-0.77	-0.81	-0.57	0.49	0.82	-0.28	Stable	2.00
FAM169A	0.12	0.14	0.42	0.12	0.11	0.11	0.10	0.12	0.12	0.05	0.11	0.08	0.11	0.12	0.12	0.12	0.18	1.56	0.64	Stable	2.00
IQGAP2	-0.44	-0.44	-0.26	-0.52	-0.22	-0.44	-0.06	-0.53	-0.52	-0.43	-0.62	-0.07	-0.62	-0.51	-0.49	-0.43	0.88	0.97	-0.05	Stable	2.00
F2RL1	0.68	0.33	0.18	0.71	0.09	0.33	0.24	0.48	0.49	0.26	0.83	0.25	0.83	0.50	0.64	0.74	0.63	0.88	-0.19	Stable	2.00
ENSSSCG00000014097	0.28	0.36	0.15	-0.13	0.36	0.36	0.00	0.20	-0.07	0.15	0.14	0.27	0.14	0.28	0.25	0.32	0.80	0.90	-0.15	Stable	2.00
MTX3	-0.10	-0.12	0.10	0.00	0.23	-0.12	0.20	-0.18	-0.01	0.05	-0.09	0.08	-0.20	-0.21	-0.09	-0.17	0.72	1.97	0.98	Stable	2.00
ZFYVE16	-0.31	-0.38	-0.22	-0.45	-0.16	-0.38	-0.21	-0.09	-0.09	0.06	-0.32	-0.20	-0.51	-0.26	-0.27	-0.19	0.60	0.85	-0.24	Stable	2.00
FAM151B	0.09	0.00	-0.08	0.10	-0.09	0.00	0.12	0.12	0.25	0.25	-0.08	-0.06	-0.18	0.08	0.03	0.05	0.22	-56.83	5.83	Stable	2.00
ANKRD34B	-0.04	0.00	-0.01	0.08	-0.18	0.00	0.27	0.06	-0.07	0.10	0.00	0.50	0.20	0.00	-0.03	0.11	0.33	0.21	-2.25	Stable	2.00
RASGRF2	0.23	0.11	0.17	0.44	0.17	0.11	0.52	0.19	0.64	0.58	0.02	0.01	-0.07	0.14	0.19	0.19	0.40	1.50	0.59	Stable	2.00
ENSSSCG00000014132	0.05	-0.20	0.04	-0.23	0.27	-0.20	0.02	-0.13	0.22	0.15	-0.43	-0.26	-0.76	-0.04	-0.16	-0.09	0.20	0.12	-3.06	Stable	2.00
VCAN	0.00	0.00	0.00	-0.15	0.00	0.00	0.00	0.00	0.00	0.00	-0.15	-0.15	-0.30	0.00	0.00	0.00	0.05	0.00	#NAME?	Down	2.00
ARSK	-0.11	0.12	0.11	0.00	0.02	0.12	0.05	0.12	0.10	0.05	0.02	0.02	-0.08	0.12	0.12	0.21	0.42	1.76	0.82	Stable	2.00
RFESD	0.08	-0.25	-0.09	-0.22	-0.38	-0.25	-0.27	-0.59	0.29	0.03	-0.40	-0.37	-0.88	-0.25	-0.63	0.16	0.63	0.74	-0.44	Stable	2.00
MBLAC2	-0.28	-0.47	-0.41	-0.30	-0.21	-0.47	-0.12	-0.30	-0.12	-0.30	-0.50	-0.59	-0.71	-0.47	-0.26	-0.26	0.18	0.73	-0.45	Stable	2.00
NR2F1	-0.34	-0.34	-0.21	-0.36	-0.34	-0.34	-0.34	-0.34	-0.34	-0.34	-0.31	-0.53	-0.44	-0.34	-0.34	-0.34	0.13	0.87	-0.20	Stable	2.00
CAST	0.11	0.46	0.20	0.00	0.34	0.46	0.22	0.20	0.11	0.46	0.03	-0.03	-0.42	0.46	0.23	0.46	0.51	1.52	0.60	Stable	2.00
ERAP1	-0.51	-0.32	0.42	0.43	0.06	-0.32	0.27	0.36	-0.40	-0.32	-0.06	0.22	0.72	-0.32	0.09	-0.32	0.60	-1.11	0.15	Stable	2.00
SLCO6A1	0.03	0.07	0.09	-0.03	0.04	0.07	0.23	-0.02	0.03	0.07	-0.03	-0.15	-0.12	0.07	0.03	0.07	0.09	-6.54	2.71	Stable	2.00
PAM	0.42	-0.02	0.49	0.18	0.55	-0.02	0.28	0.14	0.26	-0.02	0.53	0.54	1.10	-0.02	0.24	-0.02	0.42	0.63	-0.67	Stable	2.00
ENSSSCG00000014198	-0.05	0.07	0.07	0.05	0.07	0.04	-0.08	0.07	-0.13	0.04	0.11	0.07	0.16	0.07	0.04	0.00	0.11	0.13	-2.95	Stable	2.00
APC	0.28	-0.02	-0.01	0.00	-0.02	-0.02	0.19	-0.02	0.03	-0.02	-0.02	-0.02	-0.02	-0.02	-0.02	-0.01	0.15	-3.23	1.69	Stable	2.00
PGGT1B	-0.11	0.03	-0.74	-0.60	-0.18	0.03	-1.12	0.03	0.20	-0.69	-0.05	-0.24	-0.14	0.03	-0.67	-0.70	0.90	0.92	-0.13	Stable	2.00
FEM1C	-0.19	-0.10	-0.24	-0.26	-0.17	-0.10	-0.26	-0.10	-0.05	-0.16	-0.18	-0.17	-0.26	-0.10	-0.18	-0.18	0.34	0.83	-0.27	Stable	2.00
CDO1	0.52	0.65	-0.08	0.03	0.54	0.65	-0.04	0.65	0.32	0.36	0.56	0.54	0.20	0.65	0.23	0.39	0.74	0.89	-0.17	Stable	2.00
ARL14EPL	1.04	0.92	0.70	0.72	0.68	0.92	0.55	0.92	1.19	0.88	0.69	0.21	0.75	0.92	0.98	0.89	0.10	1.26	0.33	Stable	2.00
COMMD10	0.06	0.18	0.14	0.00	0.08	0.18	-0.15	0.18	0.15	0.16	0.13	0.08	0.07	0.18	0.10	0.08	0.91	1.05	0.07	Stable	2.00
ENSSSCG00000014233	0.41	0.41	0.41	0.14	0.41	0.41	0.09	0.41	0.41	0.41	0.09	0.41	-0.13	0.41	0.41	0.41	0.26	1.38	0.46	Stable	2.00
ALDH7A1	-0.33	-1.12	-1.14	-0.42	-0.56	-0.41	-0.29	-1.06	-0.80	0.21	-0.43	-1.06	-0.58	-1.06	-0.10	-0.70	0.24	1.49	0.58	Stable	2.00
MARCHF3	-0.12	-0.16	-0.16	0.06	-0.10	-0.11	-0.08	-0.11	-0.07	-0.10	-0.05	-0.11	-0.03	-0.11	-0.13	-0.09	0.05	1.77	0.82	Stable	2.00
C5orf63	0.44	-0.06	0.06	-0.12	-0.20	-0.63	-1.23	0.13	-0.79	-1.56	0.64	0.13	0.59	0.13	-0.93	-0.08	0.71	1.81	0.86	Stable	2.00
KIF3A	0.00	0.00	0.00	0.00	-0.18	0.02	0.02	-0.20	0.02	0.00	0.05	-0.24	0.00	0.00	0.02	0.02	0.62	0.42	-1.27	Stable	2.00
CDKL3	-0.24	-0.49	-0.39	-0.34	-0.34	-0.38	-0.36	-0.17	-0.29	-0.31	-0.41	-0.24	-0.39	-0.24	-0.21	-0.19	0.58	0.92	-0.12	Stable	2.00
JADE2	-0.06	-0.01	0.00	0.00	0.00	0.00	-0.13	0.00	0.00	0.00	0.00	0.00	0.00	0.00	0.00	0.00	0.18	105.55	6.72	Stable	2.00
SAR1B	-0.83	-0.57	-0.56	-0.53	-0.55	-0.53	-0.53	-0.30	-0.42	-0.55	-0.23	-0.23	-0.14	-0.23	-0.41	-0.47	0.41	1.19	0.25	Stable	2.00
ENSSSCG00000014314	-0.12	-0.13	-0.13	-0.60	0.06	-0.10	-0.10	-0.13	-0.12	-0.12	-0.12	-0.63	-0.64	-0.13	-0.12	-0.12	0.15	0.43	-1.23	Stable	2.00
TGFBI	0.35	0.34	0.41	-0.04	-0.08	-0.07	0.16	0.20	0.29	0.21	0.10	0.17	-0.02	0.41	0.20	0.30	0.00	4.16	2.06	Up	2.00

SMAD5	0.25	0.09	0.01	0.62	0.41	0.19	0.33	0.19	0.12	0.03	0.31	0.46	0.83	-0.01	0.39	0.26	0.06	0.44	-1.19	Stable	2.00
STING1	0.40	-0.30	0.49	-0.33	0.25	0.30	-0.13	-0.09	0.38	0.28	-0.44	0.75	0.36	0.28	0.47	0.19	0.91	1.12	0.16	Stable	2.00
DNAJC18	0.22	0.44	0.04	0.25	0.29	0.26	0.35	0.23	0.16	0.36	0.26	-0.06	0.13	0.11	0.07	0.26	0.82	0.93	-0.11	Stable	2.00
ENSSSCG00000014361	0.18	0.32	0.47	0.19	0.35	0.19	0.33	0.32	0.26	0.13	0.17	0.22	0.27	0.48	0.12	0.21	0.09	1.44	0.52	Stable	2.00
DIAPH1	0.01	-0.19	0.27	-0.11	-0.51	-0.32	-0.18	-0.04	-0.14	0.01	-0.08	-0.20	-0.29	0.17	-0.21	-0.29	0.05	0.18	-2.50	Stable	2.00
DELE1	0.07	0.25	0.03	-0.03	0.21	0.11	0.21	0.25	0.21	0.17	-0.02	0.11	0.15	-0.16	0.06	0.07	0.78	1.17	0.23	Stable	2.00
ARHGAP26	-0.09	-0.09	-0.18	-0.13	-0.06	-0.09	0.00	-0.09	-0.09	-0.18	-0.09	-0.18	-0.09	-0.04	-0.18	0.02	0.88	0.95	-0.07	Stable	2.00
LARS1	0.03	-0.11	0.06	0.25	0.07	0.05	0.08	-0.11	-0.15	0.02	0.26	0.26	0.25	0.03	-0.10	-0.03	0.01	-0.23	-2.09	Down	2.00
STK32A	0.19	0.17	0.10	0.41	0.15	0.11	0.19	0.17	0.01	0.11	0.28	0.28	0.34	0.24	0.11	0.07	0.17	0.66	-0.60	Stable	2.00
SPINK5	-0.07	0.01	-0.06	-0.07	-0.07	0.00	-0.06	0.00	0.01	-0.01	0.00	-0.06	0.00	0.00	-0.03	0.00	0.92	0.93	-0.10	Stable	2.00
SPINK9	0.24	0.18	0.30	0.78	0.89	0.22	0.45	0.33	-0.18	0.21	0.69	0.96	0.65	0.57	0.35	0.20	0.05	0.49	-1.04	Stable	2.00
FBXO38	0.04	0.12	0.06	0.12	0.09	0.12	0.06	0.12	0.12	0.12	0.06	0.06	0.16	0.16	0.04	0.12	0.41	0.84	-0.26	Stable	2.00
HTR4	0.15	0.72	-0.11	0.42	0.46	0.57	-0.02	0.61	-0.08	0.65	0.42	0.85	0.90	0.07	0.04	0.46	0.01	0.29	-1.78	Down	2.00
GRPEL2	-0.12	0.44	0.08	-0.12	-0.48	0.00	0.10	0.16	0.72	0.00	-0.13	-0.29	-0.45	0.22	0.40	-0.26	0.00	-1.15	0.20	Stable	2.00
PCYOX1L	-0.20	-0.20	-0.13	-0.38	-0.47	-0.40	-0.04	-0.21	0.20	-0.31	-0.32	-0.43	-0.47	-0.12	0.19	-0.30	0.00	0.16	-2.62	Down	2.00
IL17B	-0.11	0.72	0.03	-0.42	-0.78	0.24	0.55	0.04	1.02	0.27	0.16	-0.48	-0.78	-0.03	0.31	0.03	0.03	-1.44	0.52	Stable	2.00
CSNK1A1	0.02	0.38	0.15	0.12	-0.29	0.30	0.12	0.10	0.44	0.24	0.45	-0.32	-0.03	0.14	0.31	0.23	0.31	2.39	1.25	Stable	2.00
ARHGEF37	0.03	-0.42	-0.03	0.20	0.09	-0.79	-0.45	-0.22	-0.38	-0.26	-0.22	0.21	-0.56	-0.33	-0.25	-0.62	0.93	1.06	0.08	Stable	2.00
PDE6A	0.56	0.41	0.00	-0.14	-0.11	-0.01	0.26	0.35	1.28	-0.03	0.05	-0.13	-0.18	0.42	0.36	-0.03	0.00	-6.29	2.65	Up	2.00
CAMK2A	0.06	0.21	-0.05	-0.28	-0.26	-0.17	0.05	0.17	0.50	-0.17	-0.20	-0.21	-0.25	0.07	-0.05	-0.19	0.00	-0.55	-0.85	Stable	2.00
CDX1	0.15	-0.35	-0.48	-0.08	-0.40	-0.40	0.14	-0.11	0.34	-0.37	0.22	-0.29	-0.49	0.01	-0.29	-0.31	0.16	0.28	-1.83	Stable	2.00
RIC8A	-0.41	-0.35	-0.03	-0.23	-0.59	-0.25	0.37	-1.00	-0.40	-0.08	0.23	0.05	-0.53	0.53	-0.53	-0.27	0.93	1.09	0.13	Stable	2.00
SIRT3	-0.44	-0.41	-0.57	-0.02	-0.48	0.13	-0.03	-0.96	-0.43	0.14	0.07	0.23	0.11	-0.38	0.11	-0.21	0.02	167.53	7.39	Up	2.00
ENSSSCG00000014565	0.00	0.00	0.02	0.00	0.12	0.09	0.00	0.00	0.00	0.00	0.01	0.08	0.00	0.08	0.00	0.01	0.21	0.34	-1.57	Stable	2.00
RPL27A	0.00	0.11	0.00	0.04	-0.15	-0.25	0.00	0.00	0.09	-0.08	-0.16	0.00	-0.15	0.11	0.11	-0.15	0.00	-0.46	-1.12	Down	9.00
NRIP3	-0.60	0.31	0.46	-0.22	-0.06	0.04	-0.58	-0.48	-0.11	-0.46	0.33	-0.48	-0.06	0.11	0.39	-0.06	0.76	0.52	-0.94	Stable	9.00
ENSSSCG00000014575	-0.79	-0.20	-0.04	-0.21	-0.36	-0.27	-0.85	-0.90	-0.35	-0.62	-0.35	-0.90	-0.36	-0.37	-0.35	-0.36	0.71	1.13	0.17	Stable	9.00
TMEM9B	-0.50	-0.04	0.33	-0.18	-0.26	-0.37	-0.50	-0.50	-0.10	-0.39	-0.48	-0.50	-0.26	-0.04	0.25	-0.26	0.15	0.41	-1.29	Stable	9.00
TUB	-0.11	-0.73	-0.43	-0.69	-0.27	-0.51	-0.11	-0.11	-0.59	-0.17	-0.43	-0.11	-0.27	-0.74	-0.76	-0.27	0.41	1.31	0.39	Stable	9.00
SYT9	-0.89	0.18	0.46	-0.17	-0.13	-0.14	-0.89	-0.84	-0.41	-0.18	-0.14	-0.84	-0.11	-0.15	-0.07	-0.11	0.64	1.44	0.52	Stable	9.00
HPX	-0.15	-0.13	-0.11	0.00	-0.10	0.09	-0.15	-0.17	-0.19	0.11	-0.17	-0.15	-0.24	-0.13	-0.17	-0.09	0.11	2.18	1.12	Stable	9.00
SMPD1	0.00	0.00	0.00	0.12	0.26	0.07	0.00	-0.01	-0.01	0.07	-0.12	0.00	0.08	0.00	-0.01	0.08	0.09	-0.07	-3.94	Stable	9.00
APBB1	-0.27	-0.32	-0.19	-0.05	0.07	-0.47	-0.27	-0.27	-0.22	-0.20	-0.48	-0.27	-0.09	-0.32	-0.29	-0.42	0.70	1.13	0.17	Stable	9.00
CNGA4	-0.11	-0.21	-0.18	-0.02	0.03	0.00	-0.11	-0.11	-0.10	0.00	0.07	-0.11	0.00	-0.13	-0.29	0.02	0.00	113.61	6.83	Up	9.00
FAM160A2	0.23	0.22	0.22	0.26	0.22	0.16	0.23	0.23	0.11	0.16	0.15	0.23	0.20	0.17	0.16	0.13	0.72	1.04	0.06	Stable	9.00
CAVIN3	0.73	-0.20	-0.77	-0.50	-0.59	-0.83	0.73	0.78	0.36	-0.75	-0.66	0.73	-0.76	-0.08	0.41	-0.76	0.01	-0.47	-1.08	Down	9.00
TRIM68	-0.36	-0.03	0.10	-0.01	-0.13	0.05	-0.36	-0.12	0.01	0.01	0.00	-0.36	0.02	-0.18	-0.36	0.10	0.16	4.06	2.02	Stable	9.00
ENSSSCG00000014769	-0.67	-0.15	0.39	-0.21	-0.12	0.07	-0.67	0.05	0.06	-0.11	0.36	-0.67	-0.02	-0.27	-0.67	0.39	0.30	6.12	2.61	Stable	9.00
TRIM21	0.00	-0.10	-0.21	-0.10	-0.10	-0.10	0.00	0.00	0.00	-0.10	-0.10	0.00	0.00	-0.10	0.00	-0.21	0.30	0.57	-0.81	Stable	9.00
RRM1	0.26	0.20	0.20	0.19	0.20	-0.02	0.21	0.21	0.09	-0.02	0.19	0.21	0.00	0.24	0.21	0.20	0.09	1.67	0.74	Stable	9.00
ENSSSCG00000014799	-0.05	-0.05	-0.08	-0.03	-0.05	-0.03	-0.01	-0.06	-0.06	-0.03	0.01	-0.01	-0.03	-0.08	-0.01	-0.08	0.22	1.58	0.66	Stable	9.00
LAMTOR1	-0.05	-0.08	-0.03	-0.01	-0.05	-0.06	-0.13	-0.09	-0.03	-0.08	-0.14	-0.13	-0.10	0.00	-0.09	-0.03	0.65	0.85	-0.23	Stable	9.00
ATG16L2	0.32	-0.11	-0.01	0.50	-0.13	0.01	-0.03	-0.15	-0.14	0.07	0.26	-0.03	-0.08	0.27	-0.03	0.02	0.54	0.22	-2.19	Stable	9.00
MRPL48	0.37	0.12	-0.39	-0.08	-0.51	-1.05	0.12	-0.28	-0.32	-0.81	-0.69	0.14	-0.78	0.34	0.04	-0.83	0.01	0.00	-10.08	Down	9.00
COA4	0.36	0.37	0.86	1.09	0.66	1.16	0.75	1.12	1.08	1.32	0.60	0.75	1.41	0.40	0.77	0.65	0.15	0.75	-0.42	Stable	9.00

PAAF1	-0.32	-0.38	-0.55	-0.59	-0.35	-0.34	-0.23	-0.24	-0.24	-0.36	-0.55	-0.23	-0.26	-0.68	-0.23	-0.52	0.62	0.90	-0.15	Stable	9.00
DNAJB13	0.05	0.69	0.95	0.17	0.56	0.45	0.32	0.33	0.29	0.35	0.10	0.32	0.35	0.28	0.50	0.77	0.74	1.11	0.15	Stable	9.00
UCP2	0.02	-0.03	-0.09	-0.04	-0.01	-0.07	0.08	-0.01	0.04	-0.07	-0.06	0.08	-0.04	-0.07	0.14	-0.09	0.19	-0.27	-1.87	Stable	9.00
ARRB1	-0.75	-1.21	-1.09	-1.05	-0.99	-0.91	-1.21	-1.08	-0.99	-0.95	-1.04	-1.21	-0.95	-0.57	-1.20	-0.97	0.97	1.00	0.00	Stable	9.00
MAP6	0.05	-0.59	-0.58	-0.36	-0.30	0.18	-0.46	-0.15	0.06	0.38	-0.26	-0.49	0.38	0.01	-0.54	-0.25	0.27	3.13	1.65	Stable	9.00
MOGAT2	-0.74	-0.65	-0.72	-0.98	-1.23	-1.07	-1.12	-0.65	-0.79	-0.87	-1.12	-0.81	-0.87	-0.09	-1.13	-0.87	0.08	0.75	-0.41	Stable	9.00
TSKU	-0.18	0.04	0.00	0.00	0.00	-0.06	-0.07	0.06	-0.15	0.00	0.00	-0.09	0.00	0.13	0.04	0.00	1.00	1.00	-0.01	Stable	9.00
B3GNT6	-0.14	-0.07	-0.21	0.28	0.01	0.20	-0.07	0.19	0.25	0.23	0.08	0.16	0.23	0.00	-0.07	0.02	0.03	-0.09	-3.53	Down	9.00
CAPN5	0.26	0.67	0.50	0.19	0.27	0.07	0.37	0.42	-0.13	0.10	0.12	0.02	0.10	0.59	0.67	0.30	0.02	2.85	1.51	Up	9.00
ALG8	-0.93	0.73	0.97	-0.31	-1.22	-0.63	0.30	-0.93	-0.88	-1.02	-0.93	0.01	-1.06	-0.73	0.54	-0.55	0.10	0.16	-2.60	Stable	9.00
PRCP	-0.20	-0.30	0.00	-0.06	0.11	0.09	-0.33	0.02	0.02	0.19	0.08	-0.38	0.32	-0.33	-0.31	0.05	0.03	-3.56	1.83	Up	9.00
CCDC90B	-0.32	-0.37	-0.11	-0.47	-0.29	-0.35	-0.92	0.00	0.00	-0.19	-0.30	-0.54	-0.33	-0.92	-0.43	-0.08	0.64	1.21	0.28	Stable	9.00
DLG2	-0.37	-0.04	-0.15	-0.25	-0.28	-0.19	-0.27	-0.28	-0.18	-0.29	-0.27	-0.18	-0.31	-0.26	-0.18	-0.37	0.23	0.81	-0.31	Stable	9.00
TMEM126B	0.21	-0.24	-0.28	-0.12	0.11	0.12	-0.22	0.59	-0.07	-0.28	0.28	-0.02	-0.78	0.40	0.52	-0.42	0.18	-0.83	-0.28	Stable	9.00
CCDC89	0.17	0.00	0.13	0.13	0.01	-0.08	0.01	-0.17	-0.04	-0.13	0.05	-0.14	0.13	0.01	-0.29	0.17	0.58	-1.31	0.39	Stable	9.00
CTSC	0.02	-0.23	-0.24	0.00	0.01	0.01	0.02	0.03	0.02	0.03	0.01	0.02	0.02	0.02	0.03	0.02	0.23	-2.44	1.28	Stable	9.00
NAALAD2	-0.44	0.83	0.00	0.29	0.46	0.25	-0.09	1.05	-0.29	0.81	0.07	0.38	0.33	0.42	0.60	-0.37	0.94	0.94	-0.09	Stable	9.00
DEUP1	-0.18	0.42	-0.14	0.11	0.12	-0.07	-0.17	-0.20	0.83	-0.20	-0.20	0.39	0.23	1.01	0.18	-0.23	0.32	10.81	3.43	Stable	9.00
C11orf54	-0.85	-0.78	-0.03	-1.20	-0.87	-1.19	-0.48	-1.07	-1.33	-1.06	-1.05	-1.30	-1.22	-1.06	-0.81	-1.27	0.05	0.70	-0.52	Stable	9.00
ANKRD49	-0.21	-0.03	-0.02	0.04	0.05	-0.06	-0.17	-0.24	0.05	-0.15	-0.01	0.04	-0.09	0.05	-0.18	-0.38	0.73	1.33	0.41	Stable	9.00
PIWIL4	-1.32	-1.00	-1.44	-0.70	-1.03	-0.33	-1.40	-0.86	-1.16	-0.83	-0.86	-0.82	-0.71	-1.03	-0.90	-0.18	0.00	1.67	0.74	Stable	9.00
AMOTL1	-0.07	-0.28	-0.01	-0.34	-0.12	-0.33	-0.07	-0.13	-0.06	-0.13	-0.13	-0.55	-0.34	-0.22	-0.30	-0.12	0.11	0.55	-0.87	Stable	9.00
CCDC82	0.16	0.17	0.54	0.28	0.28	0.16	0.38	0.31	0.07	0.78	0.61	-0.07	-0.20	-0.07	0.26	0.02	0.98	0.98	-0.03	Stable	9.00
ENSSSCG00000014996	-1.60	-1.79	-2.36	-0.88	-1.54	-1.38	-1.68	-0.72	-0.61	-0.81	-0.21	-0.64	-0.91	-0.50	-1.18	-1.04	0.19	1.41	0.50	Stable	9.00
ENSSSCG00000014997	0.16	-0.36	-0.83	-0.29	-0.54	0.08	-0.27	0.01	-0.22	-0.18	0.43	0.08	-0.41	0.26	-0.37	0.26	0.46	2.74	1.46	Stable	9.00
SLC35F2	0.62	0.25	0.94	0.10	0.29	-0.02	0.49	0.50	-0.47	0.51	0.14	0.32	0.10	0.53	0.08	0.14	0.32	1.85	0.89	Stable	9.00
POGLUT3	-1.82	-0.87	-1.66	-0.91	-1.42	-0.44	-1.82	-1.20	0.28	-0.34	-1.27	-0.38	-0.40	0.08	0.00	-1.58	0.93	1.04	0.06	Stable	9.00
ENSSSCG00000015023	-0.47	0.06	-0.45	-0.20	-0.29	-0.09	-0.47	-0.45	-0.45	-0.47	0.33	-0.21	-0.20	-0.07	-0.12	-0.19	0.24	1.83	0.87	Stable	9.00
BCO2	-0.43	-0.12	-0.33	-0.35	-0.42	0.02	-0.43	-0.24	-0.21	-0.24	-0.12	-0.22	-0.27	-0.29	-0.15	-0.19	0.44	1.22	0.29	Stable	9.00
PCSK7	-0.09	-0.40	-0.23	-0.18	-0.22	-0.37	-0.20	-0.08	-0.11	-0.21	-0.21	-0.22	-0.22	-0.02	-0.25	-0.32	0.16	0.70	-0.51	Stable	9.00
BACE1	-0.24	0.18	-0.19	0.08	-0.27	-0.38	-0.23	-0.06	-0.45	-0.20	-0.49	0.17	-0.05	-0.03	-0.41	-0.20	0.90	1.08	0.12	Stable	9.00
ENSSSCG00000015083	0.40	-0.95	-0.09	-0.31	-0.14	-0.41	-0.35	-0.25	-0.47	-0.42	0.34	-0.23	-0.29	-0.01	-0.43	-0.98	0.85	0.88	-0.19	Stable	9.00
MPZL2	0.00	0.00	0.00	0.03	0.00	0.00	0.00	0.00	0.00	0.00	0.00	0.00	0.00	0.00	0.00	0.00	0.35	0.00	#NAME?	Stable	9.00
SLC37A4	0.00	-0.28	-0.08	-0.31	-0.59	-0.80	-0.39	-0.05	-0.53	0.33	-0.15	-0.37	-0.28	-0.49	0.04	0.53	0.93	1.08	0.11	Stable	9.00
MFRP	-0.02	-0.17	-0.05	-0.49	-0.40	-0.17	-0.21	0.18	-0.14	0.07	0.17	-0.10	-0.34	-0.08	0.09	-0.01	0.28	0.33	-1.62	Stable	9.00
NECTIN1	-0.17	-0.15	-0.26	-0.32	0.04	-0.24	-0.13	-0.16	-0.05	-0.27	-0.46	-0.25	-0.11	-0.08	-0.33	-0.27	0.29	0.71	-0.50	Stable	9.00
GRIK4	-0.09	0.12	0.11	-0.04	0.00	-0.21	0.04	0.11	-0.03	-0.05	0.00	-0.01	-0.26	0.11	-0.07	0.17	0.13	-0.79	-0.34	Stable	9.00
SORL1	0.13	0.01	0.14	0.14	0.28	0.14	0.28	0.00	0.28	0.00	0.00	0.14	0.14	0.14	0.14	0.28	1.00	1.00	0.00	Stable	9.00
ZNF202	0.10	0.10	0.10	0.19	0.20	0.01	0.14	-0.07	0.14	0.02	0.00	0.02	-0.04	0.04	0.04	-0.14	0.39	2.31	1.21	Stable	9.00
SIAE	0.11	-0.01	0.09	0.19	0.48	0.29	0.00	-0.07	0.29	0.36	0.00	0.21	0.09	0.00	0.20	0.16	0.05	0.35	-1.52	Stable	9.00
SPA17	-0.02	-0.19	0.18	0.12	-0.36	0.18	-0.13	0.22	-0.11	0.16	0.00	0.13	0.01	0.21	-0.16	0.40	0.43	0.00	-8.99	Stable	9.00
VSIG2	0.17	0.35	0.35	0.34	0.38	0.21	0.35	0.09	0.35	0.05	0.00	0.08	0.27	0.16	0.12	0.12	0.35	1.35	0.43	Stable	9.00
ROBO3	0.57	0.78	0.58	0.74	1.02	0.69	0.63	0.00	0.62	0.49	0.00	0.44	0.74	0.14	0.12	0.47	0.34	0.74	-0.43	Stable	9.00
STT3A	0.09	-0.05	-0.20	0.02	0.63	0.42	0.58	-0.02	0.52	0.27	-0.21	0.30	0.29	0.17	-0.11	0.15	0.42	0.52	-0.94	Stable	9.00
RPUSD4	0.27	0.36	0.33	0.49	0.49	0.49	0.27	0.12	0.21	0.44	0.24	0.19	0.49	0.00	0.12	0.49	0.01	0.51	-0.98	Stable	9.00

APLP2	0.20	0.05	0.09	0.39	0.39	0.61	0.02	-0.08	0.29	0.02	0.18	-0.08	0.23	0.00	0.11	0.39	0.07	0.31	-1.67	Stable	9.00
JAM3	-0.01	-0.05	0.00	-0.10	-0.06	-0.01	-0.01	0.00	-0.10	0.00	-0.06	0.00	-0.10	0.00	0.00	-0.10	0.15	0.40	-1.32	Stable	9.00
FMO4	-0.13	-0.18	-0.25	-0.29	-0.31	-0.18	-0.14	-0.11	-0.35	-0.17	-0.47	0.00	-0.58	0.00	-0.29	-0.46	0.13	0.58	-0.77	Stable	9.00
FMO1	-0.30	-0.38	-0.22	-0.58	-0.26	-0.15	-0.22	-0.31	-0.55	-0.27	-0.28	-0.31	-0.57	-0.31	-0.44	-0.54	0.68	0.92	-0.12	Stable	9.00
ATP2B4	0.20	0.22	0.21	0.24	0.52	0.39	0.20	0.25	0.18	0.30	0.36	0.25	0.43	0.25	0.22	0.37	0.00	0.61	-0.71	Stable	9.00
CDK18	-0.60	0.17	-0.23	-0.01	-0.29	-0.08	-0.19	0.17	0.14	0.35	-0.02	0.33	0.09	0.06	-0.01	0.17	0.31	-0.93	-0.10	Stable	9.00
STEAP1	0.85	0.22	-0.06	0.94	0.94	0.67	0.38	0.17	0.33	0.03	0.84	-0.29	0.70	0.35	0.81	0.87	0.31	0.65	-0.63	Stable	9.00
CFAP69	0.02	-0.18	-0.12	0.00	0.03	-0.20	-0.40	-0.38	-0.33	-0.26	-0.12	-0.36	-0.01	-0.32	-0.29	-0.09	0.09	1.99	1.00	Stable	9.00
SGCE	0.21	0.30	-0.02	-0.16	0.23	0.25	0.24	0.19	0.12	0.04	0.19	0.23	0.58	0.39	0.40	0.29	0.81	1.10	0.14	Stable	9.00
PON2	-0.12	0.04	0.15	0.11	-0.13	0.43	0.04	0.40	0.03	-0.03	0.08	-0.15	-0.15	-0.24	0.11	-0.08	0.67	5.48	2.45	Stable	9.00
PON1	-0.90	-0.56	-0.38	-0.39	-0.61	-0.24	-0.48	-0.20	0.11	-0.54	-0.54	-0.49	-0.55	-0.58	-0.54	-0.58	0.67	0.90	-0.15	Stable	9.00
ASB4	-0.50	-0.55	-0.48	-0.70	-0.72	-0.52	-0.56	-0.52	-0.49	-0.60	-0.65	-0.67	-0.45	-0.68	-0.60	-0.62	0.10	0.89	-0.17	Stable	9.00
DYNC1I1	0.33	0.76	0.95	0.68	0.36	0.60	0.95	1.25	0.50	0.89	0.61	1.21	0.35	0.96	0.67	0.55	0.34	1.21	0.28	Stable	9.00
COL28A1	-0.38	-0.34	-0.62	-0.09	-0.22	-0.32	-0.36	-0.85	0.03	-0.76	-0.13	-0.85	-0.08	-0.51	-0.40	-0.25	0.53	1.27	0.34	Stable	9.00
ICA1	-0.45	-0.46	-0.50	-0.42	-0.54	-0.50	-0.46	-0.58	-0.45	-0.58	-0.45	-0.58	-0.26	-0.52	-0.45	-0.45	0.73	1.03	0.04	Stable	9.00
AGMO	0.50	0.68	0.60	0.65	0.75	0.70	0.49	0.52	0.75	0.42	0.75	0.40	0.52	0.94	0.39	0.76	0.92	0.99	-0.02	Stable	9.00
CRPPA	-0.28	-0.36	-0.14	-0.52	-0.45	-0.25	-0.19	-0.16	-0.53	-0.26	-0.45	-0.16	-0.62	-0.11	-0.19	-0.48	0.06	0.61	-0.70	Stable	9.00
CCDC146	0.27	0.27	0.26	0.26	0.02	0.04	0.04	0.28	0.26	0.04	0.26	0.05	0.26	-0.20	0.10	0.26	0.88	1.08	0.11	Stable	9.00
FGL2	-0.24	-0.16	-0.19	0.38	0.10	0.17	0.17	0.03	-0.19	-0.15	-0.19	-0.03	-0.10	0.16	0.02	-0.19	0.61	17.38	4.12	Stable	9.00
NRCAM	0.58	0.95	0.13	0.09	0.31	0.12	0.31	0.89	0.53	0.64	0.12	0.95	0.17	0.59	0.52	0.44	0.17	1.59	0.67	Stable	9.00
ZNF786	-0.23	-0.39	-0.12	-0.22	-0.36	-0.30	-0.13	-0.39	-0.32	-0.39	-0.29	-0.39	-0.33	-0.33	-0.36	-0.33	0.36	0.87	-0.20	Stable	9.00
TMEM183A	0.00	0.00	0.00	0.00	0.00	0.00	0.00	0.00	0.00	0.00	0.00	0.00	0.01	0.00	0.00	0.00	0.41	0.33	-1.58	Stable	9.00
CHI3L1	0.00	-0.04	-0.19	-0.57	-0.32	-0.08	-0.10	0.19	-0.58	-0.02	-0.59	-0.07	-0.40	-0.26	-0.11	-0.47	0.14	0.43	-1.22	Stable	9.00
ENSSSCG00000015491	0.99	0.80	1.63	0.27	1.12	1.04	1.01	0.88	0.20	0.59	0.52	0.39	1.15	0.95	1.89	0.98	0.22	1.37	0.46	Stable	9.00
TNR	0.09	0.24	0.08	0.31	-0.12	-0.12	0.46	0.14	0.36	0.23	-0.12	-0.06	-0.12	-0.06	0.22	-0.12	0.03	-11.12	3.48	Up	9.00
FAM20B	-0.03	-0.15	0.08	-0.02	0.22	0.02	0.07	0.32	0.11	0.28	0.25	0.15	0.16	0.37	0.13	0.18	0.58	0.74	-0.44	Stable	9.00
TOR3A	-0.06	-0.48	0.00	0.10	0.10	-0.18	-0.21	-0.02	-0.32	0.11	0.00	0.11	0.04	0.21	0.08	0.00	0.15	-2.84	1.50	Stable	9.00
AXDND1	-0.12	0.01	0.08	-0.07	0.16	0.11	-0.06	0.03	0.13	0.17	0.10	-0.06	0.24	0.01	-0.01	0.32	0.07	0.09	-3.43	Stable	9.00
ACBD6	-0.47	0.42	0.23	-0.08	-0.02	-0.39	0.66	0.19	0.55	0.48	-0.42	0.24	-0.02	-0.24	-0.51	0.47	0.73	3.26	1.70	Stable	9.00
MR1	-0.42	-0.16	-0.26	-0.28	-0.15	-0.24	-0.20	-0.12	-0.15	-0.07	-0.16	-0.17	-0.15	-0.19	-0.16	-0.13	0.38	1.22	0.28	Stable	9.00
NMNAT2	-0.27	-0.25	-0.42	-0.40	-0.90	-0.52	-0.39	-0.07	-0.25	-0.10	-0.79	-0.47	-0.52	-0.65	-0.52	-0.65	0.10	0.65	-0.63	Stable	9.00
SWT1	0.28	0.31	0.19	0.27	0.28	0.09	0.31	0.33	0.33	0.28	0.28	0.31	0.28	0.31	0.28	0.28	0.28	1.12	0.17	Stable	9.00
TATDN3	0.26	0.51	0.08	0.43	0.43	0.43	0.18	0.29	0.15	0.24	0.41	0.71	0.23	0.45	0.71	0.43	0.37	0.79	-0.33	Stable	9.00
NSL1	0.03	0.23	0.08	0.15	0.19	0.10	0.03	0.11	0.05	0.05	0.20	0.30	0.04	0.19	0.16	0.15	0.40	0.76	-0.39	Stable	9.00
PPP2R5A	0.05	0.28	0.09	0.02	-0.01	0.14	0.09	0.06	0.17	-0.03	0.19	0.18	0.00	-0.06	0.66	0.05	0.27	2.50	1.32	Stable	9.00
SYT14	1.08	0.24	0.24	0.28	0.19	0.24	1.08	0.24	0.58	0.59	0.29	0.59	0.28	0.71	0.47	0.38	0.14	1.63	0.70	Stable	9.00
C1orf74	0.77	0.66	0.55	0.44	0.03	0.46	0.77	0.46	0.17	0.36	0.23	0.66	0.29	0.72	0.33	0.73	0.19	1.38	0.47	Stable	9.00
G0S2	-0.59	-0.30	-0.11	-0.20	-0.36	-0.31	-0.59	-0.20	-0.30	-0.56	-0.24	-0.40	-0.21	-0.41	-0.23	-0.44	0.97	1.01	0.01	Stable	9.00
CAMK1G	0.49	0.07	0.15	0.15	0.19	0.18	0.49	0.14	0.17	0.33	0.30	0.31	0.14	0.37	0.31	0.26	0.53	1.18	0.24	Stable	9.00
PFKFB2	0.11	0.09	0.05	0.29	0.14	0.01	0.17	0.17	0.39	0.07	0.42	0.03	0.39	-0.01	0.45	0.38	0.66	0.83	-0.27	Stable	9.00
UBXN4	0.00	-0.02	-0.08	-0.32	-0.43	-0.02	-0.11	-0.14	0.00	-0.02	-0.14	-0.07	-0.17	-0.25	-0.01	0.00	0.31	0.54	-0.90	Stable	15.00
RAB3GAP1	0.02	0.15	0.12	0.30	0.29	-0.16	0.14	0.15	-0.05	-0.16	0.04	0.07	0.10	0.15	0.11	0.15	0.79	1.23	0.30	Stable	15.00
TMEM163	0.07	0.12	-0.02	0.16	0.03	0.00	0.02	0.12	0.04	0.00	-0.03	-0.02	-0.07	0.01	0.02	-0.05	0.19	15.61	3.96	Stable	15.00
CCDC93	0.31	1.35	0.51	0.91	1.04	2.04	0.27	1.02	0.67	1.37	1.14	0.43	0.78	0.30	0.31	1.48	0.03	0.52	-0.95	Stable	15.00
ERCC3	0.00	0.00	0.00	0.00	0.00	0.00	0.00	0.00	0.00	0.00	0.00	0.00	0.23	0.00	0.00	0.00	0.35	0.00	#NAME?	Stable	15.00

IMP4	0.02	0.31	0.13	-0.10	0.18	0.23	0.11	0.23	0.23	0.04	0.27	0.02	0.06	0.15	-0.22	0.25	0.97	1.02	0.03	Stable	15.00
TMEM177	0.22	0.23	0.23	0.00	0.37	0.07	0.36	0.06	0.02	0.13	0.23	0.36	0.02	0.04	0.25	0.09	0.78	1.12	0.16	Stable	15.00
PTPN4	-0.22	-0.03	-0.26	-0.57	-0.18	0.21	0.21	-0.23	-0.18	-0.18	-0.22	-0.17	-0.32	-0.10	0.39	-0.57	0.13	0.22	-2.20	Stable	15.00
DLGAP2	-0.87	-1.33	-1.45	-1.27	-1.48	-1.31	-1.16	-1.02	-0.77	-1.21	-1.71	-1.50	-0.85	-0.71	-1.20	-1.43	0.05	0.79	-0.34	Stable	15.00
AGA	0.00	0.00	0.47	0.00	0.00	0.00	0.00	0.00	0.00	0.00	0.00	0.00	0.00	0.00	0.00	0.00	0.35	Inf	Inf	Stable	15.00
TRAPPC11	0.00	0.00	0.00	0.00	0.00	0.00	0.00	0.00	0.00	0.00	-0.01	0.00	-0.01	0.00	0.00	0.00	0.17	0.00	#NAME?	Stable	15.00
CDKN2AIP	0.11	0.18	0.11	0.05	0.18	0.11	0.11	0.18	0.11	0.18	0.10	0.11	0.25	0.11	0.11	0.26	0.34	0.81	-0.30	Stable	15.00
ACSL1	-0.06	0.00	-0.06	0.08	0.05	-0.06	-0.06	0.00	-0.06	0.10	-0.07	-0.06	0.11	-0.06	-0.06	0.37	0.06	-0.75	-0.42	Stable	15.00
PRIMPOL	-0.12	-0.20	-0.23	-0.17	-0.15	-0.17	-0.23	-0.09	-0.10	-0.19	0.12	-0.19	0.11	-0.23	-0.21	-0.26	0.25	1.60	0.68	Stable	15.00
LRP2BP	-0.04	-0.04	-0.04	-0.04	-0.04	-0.04	-0.04	-0.04	-0.04	-0.04	0.31	-0.04	0.50	-0.04	-0.04	-0.04	0.18	-0.52	-0.93	Stable	15.00
UFSP2	-0.10	-0.10	-0.10	-0.34	-0.10	-0.09	-0.10	-0.10	-0.10	0.06	0.26	-0.10	0.08	-0.10	-0.10	-0.26	0.60	1.59	0.67	Stable	15.00
C4orf47	-0.20	-0.15	-0.20	-0.19	-0.15	-0.19	-0.20	-0.15	-0.20	-0.15	-0.09	-0.20	-0.16	-0.20	-0.20	-0.10	0.07	1.21	0.28	Stable	15.00
KLKB1	-0.55	-0.66	-0.39	-0.33	-0.51	-0.23	-0.39	-0.67	-0.70	-0.31	-0.62	-0.55	-0.55	-0.39	-0.55	-0.20	0.12	1.30	0.38	Stable	15.00
ENSSSCG00000015802	-0.30	-0.30	-0.25	-0.10	-0.30	-0.10	-0.25	-0.36	-0.37	-0.10	-0.14	-0.31	0.16	-0.25	-0.30	-0.09	0.01	2.43	1.28	Up	15.00
ADAM32	-0.10	-0.25	-0.24	-0.11	-0.25	-0.34	-0.24	-0.10	0.14	-0.18	-0.06	-0.04	-0.58	-0.24	-0.12	-0.45	0.23	0.58	-0.79	Stable	15.00
ENSSSCG00000015814	0.43	0.60	0.45	-0.19	0.45	-0.12	0.45	0.40	0.46	-0.31	0.06	0.46	-0.57	0.45	0.13	-0.07	0.01	-11.53	3.53	Up	15.00
DDHD2	-0.25	0.01	0.15	-0.38	0.15	-0.20	0.15	-0.34	-1.07	-0.31	0.30	-0.48	0.64	0.15	-0.31	0.23	0.38	29.36	4.88	Stable	15.00
WRN	-0.18	0.01	-0.10	0.15	0.00	0.10	-0.10	-0.04	0.01	0.04	0.05	-0.14	0.02	-0.10	-0.10	0.20	0.01	-1.42	0.51	Stable	15.00
SFT2D3	-0.32	-0.32	-0.38	-0.32	-0.38	-0.31	-0.38	-0.32	-0.10	-0.32	-0.26	-0.32	-0.26	-0.38	-0.32	-0.38	0.95	0.99	-0.01	Stable	15.00
GPD2	-0.06	-0.07	0.00	-0.30	-0.15	-0.30	0.00	-0.13	-0.15	-0.07	-0.30	-0.02	-0.30	0.00	-0.13	-0.30	0.01	0.31	-1.67	Down	15.00
METTL8	-0.31	-0.41	-0.02	-0.42	-0.22	-0.43	-0.02	-0.43	-0.21	-0.18	-0.41	-0.19	-0.41	-0.02	-0.34	-0.43	0.15	0.66	-0.61	Stable	15.00
METAP1D	0.02	0.15	-0.03	0.06	0.00	0.07	-0.03	0.15	0.00	0.02	0.08	0.00	0.08	-0.03	0.03	0.05	0.69	0.74	-0.44	Stable	15.00
PDK1	-0.32	-0.35	-0.18	-0.49	-0.35	-0.42	-0.18	-0.35	-0.37	-0.35	-0.52	-0.35	-0.52	-0.18	-0.19	-0.53	0.00	0.60	-0.74	Stable	15.00
MAP3K20	-0.48	-0.78	-1.29	-0.61	-1.07	-1.07	-1.29	-0.84	-0.93	-0.77	-0.33	-0.90	-0.33	-1.29	-0.87	-0.84	0.14	1.31	0.39	Stable	15.00
NFE2L2	-0.83	-0.75	-0.83	-0.48	-0.51	-0.71	-0.75	-0.75	-0.41	-0.70	-1.09	-0.56	-0.63	-0.83	-0.49	-0.34	0.43	1.13	0.17	Stable	15.00
RBM45	0.36	0.24	0.48	0.38	0.40	0.33	0.24	0.24	0.20	0.69	0.20	0.23	0.64	0.48	0.54	0.38	0.47	0.86	-0.22	Stable	15.00
FKBP7	-0.25	-0.25	-0.25	-0.25	-0.25	-0.25	-0.25	-0.25	-0.05	-0.25	-0.25	-0.25	-0.25	-0.25	-0.25	-0.25	0.44	0.92	-0.13	Stable	15.00
COL5A2	-0.35	-0.35	-0.35	-0.33	-0.28	-0.33	-0.35	-0.35	-0.40	-0.34	-0.28	-0.35	-0.33	-0.35	-0.34	-0.33	0.02	1.09	0.13	Stable	15.00
HIBCH	0.20	0.28	0.13	-0.61	-0.46	-0.83	0.28	0.28	0.24	0.03	0.73	-0.42	-0.60	0.13	-0.15	-0.98	0.02	-0.44	-1.18	Down	15.00
NABP1	0.01	0.01	0.01	-0.54	-0.13	-0.42	0.01	0.01	-0.67	-0.49	-0.18	-0.18	-0.21	0.01	-0.30	-0.26	0.10	0.38	-1.40	Stable	15.00
PGAP1	-0.53	-0.26	-0.53	-0.09	-0.57	-0.64	-0.53	-0.34	0.33	-0.36	0.55	-0.61	0.47	-0.53	-0.87	-0.64	0.44	1.73	0.79	Stable	15.00
WDR12	-0.17	0.47	-0.10	-0.08	0.10	0.10	-0.06	0.37	0.00	-0.14	0.19	0.10	-0.17	-0.10	0.10	0.10	0.71	2.37	1.25	Stable	15.00
PIKFYVE	-0.10	0.07	-0.02	-0.10	-0.19	-0.10	-0.19	0.07	-0.10	-0.06	-0.16	-0.23	-0.23	-0.10	-0.10	-0.10	0.05	0.40	-1.33	Down	15.00
MREG	0.00	0.11	0.02	-0.61	0.12	-0.09	0.13	0.24	0.24	-0.28	0.11	-0.29	-0.39	0.00	0.02	0.00	0.02	-0.53	-0.92	Stable	15.00
CATIP	0.22	0.20	0.42	-0.26	0.30	0.68	0.12	-0.01	0.48	0.16	0.27	0.32	-0.14	0.13	0.50	0.13	0.56	1.41	0.49	Stable	15.00
CYP27A1	-1.03	-1.11	-1.07	-1.02	-1.09	-0.94	-1.05	-0.28	-0.81	-0.87	-0.39	-0.88	-0.90	-1.11	-0.78	-1.35	0.86	0.97	-0.04	Stable	15.00
PRKAG3	-0.03	-0.03	-0.03	-0.14	-0.03	0.10	-0.03	-0.07	-0.14	-0.14	0.00	-0.14	0.07	0.07	0.00	0.07	0.84	1.35	0.44	Stable	15.00
TTLL4	-0.98	-0.88	-0.84	-0.75	-0.96	-0.61	-0.73	0.08	-0.25	-0.50	-0.23	-0.50	-0.79	-1.25	-0.35	-1.28	0.79	0.92	-0.11	Stable	15.00
ENSSSCG00000016215	0.74	0.84	1.08	1.03	1.05	0.78	0.89	0.03	0.88	0.85	0.48	0.97	0.84	1.24	0.80	1.16	0.59	0.91	-0.14	Stable	15.00
TUBA4A	0.37	0.15	0.29	0.19	0.35	0.30	0.34	0.04	0.32	0.24	0.11	0.18	0.38	0.41	0.16	0.41	0.84	0.96	-0.06	Stable	15.00
DNPEP	-0.10	0.04	0.04	-0.14	0.04	-0.10	0.04	0.03	0.01	-0.02	-0.28	-0.07	0.10	0.08	0.14	0.08	0.12	-0.78	-0.36	Stable	15.00
SERPINE2	0.19	0.45	0.20	0.37	0.04	0.38	0.11	0.14	0.19	0.18	0.37	0.17	0.37	0.19	-0.08	0.45	0.13	0.60	-0.74	Stable	15.00
COL4A4	-0.16	-0.21	-0.14	-0.09	-0.14	-0.19	-0.09	0.00	-0.27	-0.19	-0.15	-0.24	-0.16	-0.14	-0.16	-0.14	0.64	0.90	-0.14	Stable	15.00
COL4A3	-0.07	-0.08	-0.08	-0.04	-0.06	-0.08	-0.06	0.01	-0.09	-0.08	-0.05	-0.08	-0.06	-0.06	-0.06	-0.07	1.00	1.00	0.00	Stable	15.00
TM4SF20	0.00	-0.06	0.22	0.35	0.23	0.10	0.12	0.17	-0.01	0.10	0.21	-0.16	0.14	0.16	0.04	0.16	0.35	0.56	-0.83	Stable	15.00

CAB39	0.24	-0.01	0.12	0.40	0.35	0.33	0.30	0.27	0.21	0.28	0.38	0.38	0.38	-0.03	0.28	0.42	0.00	0.47	-1.08	Down	15.00
EFHD1	0.31	0.31	0.31	0.30	0.30	0.28	0.31	0.15	0.31	0.30	0.28	0.31	0.30	0.31	0.31	0.30	0.76	0.98	-0.03	Stable	15.00
RBM44	0.31	0.36	0.16	0.57	0.41	0.21	0.11	0.30	0.57	0.39	0.58	0.53	0.22	0.09	0.47	0.41	0.15	0.71	-0.49	Stable	15.00
SCLY	0.56	0.49	0.54	0.60	0.62	0.30	0.31	0.17	0.64	0.62	0.69	0.49	0.35	0.30	0.46	0.61	0.20	0.81	-0.31	Stable	15.00
ESPNL	0.57	0.58	0.65	0.38	0.59	0.24	0.14	0.28	0.51	0.59	0.53	0.59	0.27	0.29	0.51	0.58	0.75	0.94	-0.09	Stable	15.00
ASB1	0.02	0.06	0.03	0.09	0.06	-0.07	0.05	0.34	0.05	0.03	0.05	0.03	0.06	0.03	0.05	0.00	0.26	2.58	1.37	Stable	15.00
HES6	0.20	0.08	-0.22	0.58	0.65	0.24	0.24	0.24	0.58	0.21	0.89	0.35	0.66	0.14	0.54	0.45	0.04	0.45	-1.17	Down	15.00
NDUFA10	-0.08	-0.08	-0.14	0.00	-0.14	-0.19	-0.03	-0.12	-0.12	-0.08	-0.14	-0.14	-0.17	-0.03	-0.14	-0.14	0.24	0.74	-0.43	Stable	15.00
GPC1	-0.12	-0.02	-0.09	0.14	-0.09	-0.01	0.24	0.00	0.07	0.00	-0.09	-0.09	-0.04	0.25	0.00	-0.09	0.21	-1.29	0.36	Stable	15.00
ESYT2	-0.10	-0.27	0.00	-0.02	-0.08	-0.41	-0.10	-0.02	-0.16	-0.11	-0.15	-0.24	-0.41	-0.05	-0.09	-0.10	0.17	0.52	-0.93	Stable	18.00
ENSSSCG00000016411	-0.84	-0.34	-0.25	-0.25	-0.34	-0.60	-0.84	-0.88	-0.40	-0.50	-0.38	-0.45	-0.38	-0.48	-0.75	-0.44	0.10	1.43	0.52	Stable	18.00
RNF32	0.23	0.42	0.33	0.36	0.56	-0.01	0.23	0.41	0.17	0.77	0.30	0.46	0.12	0.58	0.24	0.38	0.68	0.89	-0.17	Stable	18.00
GALNT11	-0.13	-0.13	-0.13	-0.50	-0.13	-0.58	-0.13	-0.45	-0.35	-0.46	-0.23	-0.13	-0.13	-0.13	-0.37	-0.13	0.50	0.80	-0.33	Stable	18.00
GALNTL5	0.07	0.02	0.55	0.20	0.30	0.03	0.07	0.79	0.17	0.32	0.51	0.54	0.51	0.22	0.88	0.54	0.88	0.94	-0.09	Stable	18.00
ENSSSCG00000016444	-0.02	0.00	-0.04	-0.01	-0.04	0.00	0.00	-0.02	0.00	0.00	0.01	-0.04	-0.02	-0.02	-0.04	0.00	0.57	1.45	0.53	Stable	18.00
NOS3	0.07	0.03	0.02	0.18	0.03	-0.07	0.07	0.35	0.43	0.13	-0.32	0.02	-0.12	-0.01	0.30	-0.03	0.05	-7.55	2.92	Up	18.00
ENSSSCG00000016451	0.16	0.16	0.16	0.16	0.16	-0.01	0.16	0.07	0.07	0.08	0.08	0.16	0.16	0.16	0.08	0.16	0.73	1.08	0.11	Stable	18.00
CLCN1	-0.58	-0.25	-0.52	-0.71	-0.73	-0.80	-0.62	-0.76	-0.50	-0.52	-0.53	-0.73	-0.66	-0.31	-0.81	-0.68	0.13	0.81	-0.30	Stable	18.00
TRPV5	-0.23	-0.02	0.04	-0.03	-0.17	0.04	-0.17	0.07	-0.06	0.15	-0.21	-0.05	0.54	-0.07	0.34	-0.28	0.92	10.50	3.39	Stable	18.00
EPHB6	0.00	0.00	-0.09	0.03	0.00	0.03	0.00	0.01	0.00	0.00	0.00	0.00	0.09	-0.09	0.01	0.00	0.07	-1.06	0.08	Stable	18.00
SSBP1	0.14	0.29	0.08	0.02	-0.03	0.01	0.18	0.26	0.00	0.28	0.14	-0.07	-0.21	0.12	0.08	0.12	0.10	4.27	2.09	Stable	18.00
DENND11	0.07	0.01	0.12	0.29	0.27	0.14	0.08	0.31	0.14	0.12	0.18	0.29	0.31	0.15	0.02	0.23	0.02	0.50	-1.01	Down	18.00
TMEM178B	-0.07	-0.10	-0.07	-0.05	-0.01	-0.03	-0.07	-0.03	-0.05	-0.11	-0.03	-0.05	-0.10	-0.01	-0.13	-0.13	0.96	1.01	0.02	Stable	18.00
PARP12	-0.15	0.02	-0.07	-0.24	-0.15	-0.08	-0.15	-0.22	-0.14	-0.24	-0.07	-0.19	-0.33	0.01	-0.07	-0.16	0.06	0.53	-0.92	Stable	18.00
ZC3HAV1	-0.08	0.22	0.16	-0.06	-0.07	0.21	-0.07	0.05	0.04	-0.01	0.43	-0.07	0.06	0.13	0.37	-0.03	0.60	1.77	0.83	Stable	18.00
ATP6V0A4	-0.65	-0.50	-0.68	-1.10	-1.10	-0.62	-1.04	-1.37	-0.97	-0.89	-0.76	-1.10	-0.79	-0.42	-0.90	-0.93	0.47	0.89	-0.16	Stable	18.00
SVOPL	0.08	-0.08	-0.25	-0.76	-0.76	0.07	-0.34	-1.04	-0.69	-1.25	-0.95	-0.76	-0.17	1.16	-0.32	-0.30	0.15	0.30	-1.72	Stable	18.00
ENSSSCG00000016527	-0.19	-1.04	-1.30	-1.31	-1.31	-0.32	-1.18	-1.17	-1.04	-1.35	-1.32	-1.31	-1.33	-1.18	-1.33	-0.36	0.93	0.98	-0.03	Stable	18.00
MKLN1	-0.40	-0.37	-0.33	-0.37	-0.53	-0.26	0.09	0.17	0.28	-0.16	-0.40	-0.33	-0.06	-0.35	-0.27	-0.41	0.16	0.47	-1.09	Stable	18.00
MEST	0.45	0.80	0.15	0.27	0.21	0.93	0.04	0.15	0.75	0.43	0.37	0.20	0.13	0.89	0.43	0.48	0.60	1.21	0.27	Stable	18.00
CPA1	0.30	0.02	-0.19	0.31	0.25	0.35	0.02	-0.16	-0.07	-0.03	0.30	0.09	0.21	0.14	-0.21	0.33	0.01	-0.08	-3.61	Down	18.00
CCDC136	-0.78	-1.38	-1.12	-0.84	-0.84	-0.72	-1.06	-1.45	-1.49	-0.36	-0.79	-1.19	-0.54	-1.40	-1.17	-0.78	0.00	1.62	0.70	Stable	18.00
FAM71F2	0.14	0.43	0.21	0.06	0.09	0.13	0.50	0.63	0.39	0.46	0.17	0.01	0.23	0.50	-0.22	0.16	0.17	1.97	0.98	Stable	18.00
CADPS2	0.22	0.14	0.06	0.38	0.55	0.15	0.17	0.13	0.47	0.06	0.16	0.23	0.24	0.14	0.21	0.55	0.22	0.65	-0.61	Stable	18.00
AASS	0.21	-0.07	0.02	0.34	0.24	0.24	0.27	0.02	0.44	0.05	0.34	0.18	0.29	0.02	0.18	0.24	0.16	0.57	-0.82	Stable	18.00
PTPRZ1	-0.20	0.00	-0.13	-0.28	-0.01	-0.15	-0.16	-0.04	-0.28	-0.03	-0.28	-0.18	-0.14	-0.06	-0.11	-0.01	0.78	0.90	-0.16	Stable	18.00
FAM3C	-0.23	0.02	-0.26	-0.30	-0.14	-0.08	-0.25	0.03	-0.43	-0.02	-0.28	-0.21	-0.32	-0.07	-0.19	-0.14	0.84	0.93	-0.11	Stable	18.00
TSPAN12	0.18	0.09	0.07	0.24	-0.22	-0.19	0.24	-0.01	0.30	0.05	0.16	0.09	0.22	0.02	0.24	-0.22	0.16	7.97	2.99	Stable	18.00
SEPTIN7	0.20	0.07	0.23	0.33	0.53	0.53	0.16	-0.02	0.19	0.09	0.47	0.14	0.16	0.00	0.13	0.53	0.01	0.35	-1.53	Down	18.00
MINDY4	-0.43	-0.95	-0.82	-0.49	-0.65	-0.63	-0.31	-0.50	-0.19	-0.17	-0.21	-0.51	-0.10	-0.67	-0.81	-0.65	0.23	1.37	0.45	Stable	18.00
PLEKHA8	0.09	0.06	0.04	0.07	0.07	0.07	0.06	0.06	0.07	0.07	0.07	0.04	0.07	0.11	0.00	0.07	0.61	0.90	-0.14	Stable	18.00
TAX1BP1	0.03	0.03	0.00	-0.31	-0.31	-0.19	0.03	0.03	-0.15	0.03	0.03	0.00	0.03	-0.01	0.00	-0.31	0.08	0.03	-5.18	Stable	18.00
HIBADH	0.07	0.14	0.15	0.27	0.27	0.27	0.02	0.06	0.07	0.09	0.27	0.00	0.16	0.14	0.00	0.27	0.02	0.42	-1.25	Down	18.00
CBX3	-0.08	0.00	-0.08	-0.15	0.00	-0.15	-0.08	-0.08	-0.08	0.00	0.00	-0.15	-0.08	-0.08	-0.08	0.00	1.00	1.00	0.00	Stable	18.00
OSBPL3	0.00	0.00	0.07	0.00	0.00	0.00	0.00	0.00	0.00	0.00	0.00	0.00	0.00	0.00	0.16	0.00	0.20	Inf	Inf	Stable	18.00

DBNL	-0.40	-0.08	-0.09	-0.40	-0.65	-0.83	-0.31	-0.66	-0.25	-0.91	-0.56	-0.21	-0.63	-0.77	-0.36	-0.35	0.12	0.64	-0.64	Stable	18.00
TNS3	-0.02	-0.66	-0.64	-0.49	-0.38	-0.28	-0.28	-0.66	-0.56	-0.74	-0.43	-0.54	-0.37	-0.35	-0.69	-0.33	0.72	1.08	0.11	Stable	18.00
VPS41	0.32	-0.30	0.22	0.18	0.12	0.12	0.02	-0.50	-0.02	0.06	-0.06	0.30	0.30	-0.02	-0.64	0.25	0.06	-0.72	-0.46	Stable	18.00
ZNF282	0.00	-0.34	0.00	0.00	0.01	0.01	0.00	0.00	-0.35	0.00	0.01	0.00	0.00	-0.32	-0.38	0.00	0.03	-40.67	5.35	Up	18.00
ZNF622	0.51	0.28	1.35	0.43	1.34	0.01	0.68	-0.09	-0.09	0.61	1.12	0.02	1.35	-0.09	0.20	0.42	0.24	0.52	-0.95	Stable	16.00
C5orf22	0.04	-0.03	0.01	0.06	0.07	0.01	-0.03	-0.03	-0.03	-0.04	0.01	0.01	-0.01	-0.09	-0.10	0.04	0.03	-1.54	0.62	Stable	16.00
DROSHA	0.37	0.09	0.23	0.07	0.34	-0.06	0.31	0.10	0.13	0.24	0.10	0.24	0.23	0.13	0.19	0.22	0.73	1.12	0.16	Stable	16.00
ENSSSCG00000016810	0.58	0.74	0.83	0.67	0.40	0.92	0.92	0.92	1.02	1.03	0.80	0.86	1.13	1.28	1.19	-0.03	0.20	1.29	0.37	Stable	16.00
ADAMTS12	-0.05	0.00	-0.05	-0.20	-0.11	-0.12	-0.04	-0.04	-0.07	0.01	-0.01	-0.12	-0.08	-0.05	0.00	-0.49	0.11	0.27	-1.91	Stable	16.00
TARS1	0.11	-0.04	0.38	0.41	0.40	0.36	0.01	-0.13	-0.11	0.10	0.07	0.16	0.13	0.14	-0.04	0.11	0.04	0.19	-2.41	Down	16.00
RAD1	-0.65	-1.46	-1.34	-1.68	-1.03	-0.25	-0.86	-0.89	-0.90	-1.30	-1.42	-1.21	-1.36	-1.35	-1.21	-0.31	0.97	1.01	0.01	Stable	16.00
AGXT2	-0.56	-0.08	0.02	0.10	-0.10	-0.08	-0.40	-0.42	-0.44	-0.33	-0.56	-0.26	-0.60	-0.28	-0.56	-0.48	0.66	1.18	0.24	Stable	16.00
PRLR	-0.15	-0.25	-0.08	0.22	-0.07	-0.52	-0.07	-0.03	-0.09	-0.54	-0.78	-0.32	-0.50	-0.26	-0.30	-0.61	0.08	0.39	-1.36	Stable	16.00
C9	0.00	0.00	-0.02	-0.24	-0.46	0.02	0.00	-0.02	0.00	-0.07	-0.05	-0.30	-0.07	-0.05	-0.09	0.02	0.09	0.17	-2.60	Stable	16.00
C7	0.15	0.19	-0.19	0.54	0.19	0.20	0.13	0.78	0.27	-0.24	-0.22	0.51	0.27	0.51	0.33	0.32	0.62	1.37	0.45	Stable	16.00
OXCT1	-0.13	-0.84	-0.63	-0.31	-0.53	-0.52	-0.13	0.15	-0.58	-0.57	-1.28	0.08	-0.83	-0.13	-0.72	-0.59	0.32	0.66	-0.60	Stable	16.00
ENSSSCG00000016868	-0.33	0.22	-0.21	-0.32	0.36	-0.26	-0.48	-0.29	0.29	0.07	0.45	-0.05	-0.50	-0.17	0.14	0.20	0.54	19.54	4.29	Stable	16.00
ENSSSCG00000016869	0.10	0.54	-0.14	0.06	0.60	0.02	0.04	0.41	0.94	0.22	0.70	0.19	0.14	0.10	0.61	0.66	0.99	1.01	0.01	Stable	16.00
MOCS2	0.13	0.01	0.14	-0.15	-0.32	-0.28	0.04	-0.05	-0.33	0.78	0.84	0.15	-0.52	0.44	0.14	-0.31	0.85	2.42	1.28	Stable	16.00
GZMA	0.02	0.02	0.02	-0.10	0.02	0.00	0.02	0.02	0.00	0.02	0.02	0.04	0.00	0.04	0.02	0.00	0.23	70.51	6.14	Stable	16.00
PLPP1	0.00	0.01	-0.23	-0.07	-0.12	-0.07	-0.15	-0.07	-0.16	-0.03	-0.01	-0.04	-0.06	-0.04	-0.12	-0.20	0.60	1.27	0.34	Stable	16.00
SETD9	0.12	-0.28	-0.34	-0.15	-0.20	-0.34	0.26	0.21	0.10	-0.14	-0.14	-0.14	-0.15	-0.14	-0.14	-0.25	0.09	0.14	-2.85	Stable	16.00
ERCC8	0.12	0.50	0.25	0.52	0.14	0.00	0.26	0.49	0.50	0.25	0.16	0.52	0.17	0.52	0.24	0.00	0.14	1.65	0.72	Stable	16.00
RNF180	-0.71	-1.51	-0.90	-0.71	-0.96	-0.47	-1.05	-1.16	-1.27	-1.45	-0.67	-1.30	-1.24	-1.45	-1.46	-0.64	0.14	1.27	0.35	Stable	16.00
CWC27	-0.17	-0.45	-0.05	-0.01	-0.17	0.05	-0.17	-0.10	-0.10	-0.33	-0.01	-0.16	-0.20	-0.33	-0.33	0.05	0.12	2.20	1.14	Stable	16.00
MAST4	-0.57	-0.61	-0.55	-0.31	-0.58	-0.18	-0.55	-0.61	-0.50	-0.59	-0.57	-0.30	-0.30	-0.61	-0.61	-0.14	0.01	1.56	0.64	Stable	16.00
PIK3R1	0.02	-0.26	0.04	-0.46	0.00	-0.11	0.04	0.04	-0.13	0.02	-0.28	-0.14	0.04	0.04	-0.17	-0.10	0.29	0.36	-1.47	Stable	16.00
MARVELD2	-0.13	-0.30	-0.08	-0.27	-0.07	-0.04	-0.26	-0.34	-0.24	-0.36	-0.10	-0.23	-0.34	-0.26	-0.26	-0.10	0.41	1.25	0.32	Stable	16.00
RAD17	-0.05	0.33	-0.04	0.46	0.18	-0.03	-0.09	-0.09	0.09	0.01	0.15	-0.07	0.12	-0.09	0.07	0.09	0.23	0.14	-2.84	Stable	16.00
CCDC125	0.43	0.43	0.25	0.73	0.13	0.69	0.43	0.86	1.30	0.70	0.64	0.90	0.38	0.43	0.88	0.76	0.95	1.02	0.02	Stable	16.00
BDP1	0.20	1.90	0.92	2.02	1.99	2.10	1.38	0.55	1.72	1.36	0.20	1.35	1.52	1.38	1.47	0.65	0.52	0.85	-0.24	Stable	16.00
SERF1A	0.03	-0.08	0.04	-0.11	0.08	-0.04	-0.11	-0.03	-0.06	0.00	0.15	-0.12	-0.08	-0.11	-0.17	0.07	0.22	9.92	3.31	Stable	16.00
MCCC2	0.32	0.40	0.23	0.36	0.08	0.10	0.49	0.11	0.15	0.25	0.24	0.44	0.39	0.49	0.43	0.26	0.41	1.22	0.29	Stable	16.00
RANBP17	0.41	-0.21	0.40	0.68	0.50	0.17	0.14	-0.39	0.48	0.45	0.23	0.00	0.11	-0.07	0.18	0.36	0.18	0.37	-1.42	Stable	16.00
HMMR	-0.01	0.09	0.45	0.48	-0.04	0.40	-0.01	-0.03	0.02	-0.06	0.25	-0.01	-0.04	-0.01	-0.01	-0.04	0.59	0.54	-0.88	Stable	16.00
SLU7	1.13	1.08	-0.09	-0.75	0.23	0.09	0.96	0.02	-0.33	-0.15	-0.26	0.96	-0.71	1.13	1.02	0.29	0.05	-17.09	4.10	Up	16.00
PWWP2A	-0.61	-0.63	-0.60	-0.54	-0.42	-0.34	-0.65	-0.43	-0.30	-0.17	-0.60	-0.65	-0.40	-0.61	-0.66	-0.47	0.13	1.25	0.33	Stable	16.00
ADRA1B	1.35	1.35	0.53	-0.40	0.14	-0.19	1.21	0.63	-0.01	-0.03	0.33	1.21	-0.51	1.35	1.21	0.27	0.01	9.28	3.21	Up	16.00
UBLCP1	-0.40	0.20	-0.01	0.05	-0.23	0.16	-0.20	0.43	0.24	0.16	-0.45	0.04	0.50	-0.40	0.04	-0.85	0.72	0.16	-2.64	Stable	16.00
GEMIN5	0.00	-0.07	-0.09	0.00	0.00	0.00	-0.13	-0.09	-0.08	0.00	-0.09	0.00	0.00	0.00	0.00	0.00	0.05	5.06	2.34	Up	16.00
CNOT8	0.00	-0.22	-0.20	-0.46	0.00	-0.20	0.05	-0.16	-0.15	-0.56	0.01	0.00	-0.66	0.00	0.00	0.00	0.20	0.36	-1.48	Stable	16.00
FAXDC2	0.00	0.11	0.44	0.44	0.00	0.21	0.22	0.44	0.44	0.44	0.44	0.00	0.44	0.00	0.00	0.00	0.72	0.84	-0.25	Stable	16.00
GALNT10	0.10	-0.14	-0.23	0.50	0.00	0.10	0.03	-0.29	-0.44	-0.10	-0.24	0.00	-0.08	0.21	0.05	0.00	0.33	-3.89	1.96	Stable	16.00
FAM114A2	0.00	-0.02	0.02	0.10	-0.05	0.05	0.06	0.06	-0.04	0.53	0.04	-0.05	0.50	0.10	0.03	-0.05	0.25	0.21	-2.26	Stable	16.00
SLC36A2	0.63	0.28	0.38	0.22	0.41	0.10	0.65	0.31	0.51	0.15	0.20	0.63	0.16	0.57	0.52	0.44	0.04	1.66	0.73	Stable	16.00

GM2A	-0.51	-0.12	0.13	-0.12	-0.46	-0.36	0.06	-0.03	0.37	0.10	-0.31	-0.63	-0.30	0.17	-0.43	-0.57	0.06	0.14	-2.84	Stable	16.00
C5orf49	0.12	-0.28	-0.20	0.04	-0.10	-0.15	0.04	0.50	0.36	0.48	-0.02	0.04	-0.02	0.44	0.30	-0.15	0.27	11.28	3.50	Stable	16.00
MTRR	-1.35	-0.08	-0.37	-1.12	-0.29	-0.94	-1.05	0.18	-0.31	-1.02	-1.16	-0.74	-2.08	0.15	-0.08	-0.33	0.05	0.38	-1.40	Stable	16.00
NSUN2	0.04	-0.53	-0.47	-0.16	0.23	0.01	0.09	0.01	0.04	0.04	-0.16	0.11	-0.16	-0.04	-0.27	-0.17	0.31	4.44	2.15	Stable	16.00
NDUFS6	-0.13	-0.31	0.02	-0.31	-0.31	-0.29	-0.03	-0.31	-0.31	-0.31	-0.31	-0.16	-0.31	-0.31	-0.31	-0.15	0.34	0.79	-0.34	Stable	16.00
NARF	0.17	-0.07	0.02	0.21	0.17	-0.09	0.17	0.30	0.25	0.17	0.17	0.18	0.17	0.23	0.15	0.04	0.66	1.20	0.26	Stable	12.00
CYBC1	-0.34	-0.28	0.08	0.15	-0.34	-0.23	-0.34	-0.19	0.10	0.02	-0.15	0.36	-0.34	-0.03	-0.02	0.01	0.56	2.03	1.02	Stable	12.00
HEXD	0.08	0.10	0.05	0.06	0.29	-0.22	0.19	1.17	-0.03	0.14	0.21	-0.17	0.31	0.78	-0.17	0.10	0.33	3.06	1.61	Stable	12.00
FN3K	0.35	0.18	0.09	0.23	0.35	0.20	0.35	0.40	0.12	0.23	0.17	0.11	0.35	0.37	0.23	0.23	0.58	1.13	0.17	Stable	12.00
CEP131	0.00	0.00	0.00	0.00	0.00	0.00	0.00	0.00	0.00	0.07	0.07	0.15	0.00	0.00	0.07	0.14	0.09	0.18	-2.49	Stable	12.00
SGSH	-0.01	-0.05	0.35	0.28	-0.06	-0.31	-0.08	0.13	-0.02	0.14	0.16	0.44	-0.04	0.07	0.27	0.18	0.88	0.85	-0.23	Stable	12.00
CARD14	-0.27	-0.23	-0.39	-0.14	-0.44	-0.38	-0.20	-0.33	-0.18	-0.41	-0.14	-0.39	-0.20	-0.38	-0.17	-0.41	0.42	0.85	-0.23	Stable	12.00
EIF4A3	0.06	-0.12	-0.27	-0.02	-0.20	-0.18	-0.03	-0.58	-0.11	-0.13	0.08	0.06	0.04	-0.27	0.13	-0.05	0.28	3.03	1.60	Stable	12.00
GAA	0.59	0.18	0.17	0.22	0.59	0.34	0.56	-0.03	0.16	0.61	0.41	0.57	0.21	0.24	0.50	0.66	0.14	0.65	-0.62	Stable	12.00
ENSSSCG00000017164	0.00	0.11	0.20	0.16	0.00	-0.01	0.13	0.14	0.11	0.00	-0.04	0.00	0.28	0.08	0.00	0.00	0.35	1.92	0.94	Stable	12.00
ENSSSCG00000017165	-0.07	0.11	0.63	0.69	-0.15	-0.34	0.17	0.72	0.43	0.44	0.35	1.14	0.31	0.35	0.86	0.66	0.95	1.03	0.04	Stable	12.00
CYTH1	0.21	0.39	0.52	0.36	0.70	0.62	0.49	0.56	0.45	0.35	0.33	0.26	0.79	0.50	0.25	0.51	0.42	0.86	-0.22	Stable	12.00
SEC14L1	-0.11	-0.13	0.11	-0.15	0.00	0.17	-0.26	0.05	0.04	-0.02	-0.15	0.01	0.00	-0.29	0.03	-0.15	0.62	1.92	0.94	Stable	12.00
EXOC7	-0.14	-0.24	0.04	-0.20	-0.01	-0.28	-0.13	0.04	-0.14	-0.22	0.21	-0.27	-0.38	-0.26	-0.14	-0.19	0.56	0.72	-0.46	Stable	12.00
FBF1	0.55	0.70	0.37	0.49	0.12	0.61	0.68	0.74	0.61	0.22	0.48	0.58	0.31	0.69	0.63	0.49	0.02	1.51	0.60	Stable	12.00
CDR2L	-0.09	0.17	0.10	0.65	0.38	0.30	0.28	-0.19	-0.08	-0.02	0.16	0.30	0.26	0.33	0.00	0.30	0.03	0.22	-2.15	Down	12.00
RPL38	0.03	-0.08	0.43	0.34	0.11	0.11	0.27	0.01	0.11	-0.05	0.15	0.23	0.12	0.15	0.28	0.23	0.96	0.98	-0.03	Stable	12.00
C17orf80	-0.09	0.21	0.10	-0.17	0.19	0.21	0.03	-0.06	-0.14	0.19	-0.07	0.17	0.14	0.03	0.16	0.19	0.26	0.28	-1.82	Stable	12.00
SOX9	0.29	-0.34	-1.56	-0.10	-0.15	-0.45	-0.24	-0.69	-0.70	0.11	-0.59	-0.10	0.00	0.21	-0.10	-0.30	0.41	2.00	1.00	Stable	12.00
ABCA6	-0.14	0.00	-0.06	-0.15	-0.07	0.01	-0.38	-0.21	-0.04	-0.20	-0.12	-0.04	-0.04	-0.34	-0.33	-0.22	0.19	1.82	0.86	Stable	12.00
ABCA9	-0.13	0.31	0.12	0.10	0.36	0.22	0.13	0.35	0.53	0.28	0.43	0.40	0.42	0.21	0.00	0.43	0.13	0.58	-0.78	Stable	12.00
FAM20A	0.15	0.00	0.28	0.18	0.29	0.20	0.18	-0.02	0.00	0.00	0.21	0.18	0.08	0.06	0.15	0.16	0.22	0.61	-0.71	Stable	12.00
ARSG	0.11	0.32	0.38	0.36	0.50	0.25	0.13	0.67	0.41	0.73	0.54	0.14	0.38	0.54	0.35	0.35	0.64	0.89	-0.16	Stable	12.00
AMZ2	-0.89	-0.45	-0.93	-0.68	-1.10	-0.92	-0.97	-0.17	-0.30	-0.22	-0.60	-0.77	-0.83	-0.37	-0.47	-0.62	0.32	0.79	-0.34	Stable	12.00
PSMD12	0.12	0.35	-0.01	0.23	0.15	0.61	0.11	-0.06	0.32	-0.15	0.36	0.47	0.59	0.51	0.27	0.03	0.50	0.71	-0.49	Stable	12.00
TEX2	-0.20	0.90	0.93	0.68	0.72	0.42	0.45	1.11	0.95	0.74	-0.04	0.91	0.86	1.06	0.70	0.83	0.62	1.15	0.20	Stable	12.00
SCN4A	0.25	0.29	-0.05	0.12	0.23	0.02	-0.24	0.10	-0.07	-0.72	-0.02	-0.20	0.07	0.03	-0.07	0.11	0.54	-0.64	-0.64	Stable	12.00
ENSSSCG00000017285	-0.27	0.08	0.18	-0.24	-0.35	0.08	-0.27	0.04	-0.31	-0.10	0.05	-0.25	-0.38	-0.09	0.02	-0.22	0.29	0.44	-1.18	Stable	12.00
STRADA	0.11	0.08	0.30	0.16	0.33	0.07	0.09	0.12	0.01	0.15	0.17	0.23	0.14	0.13	0.21	0.29	0.17	0.68	-0.56	Stable	12.00
ACE	0.10	-0.72	-0.57	-0.27	-0.36	-0.40	0.06	-0.54	-0.68	-0.40	-0.15	-0.82	-0.72	-0.60	-0.74	-0.40	0.89	1.05	0.07	Stable	12.00
TANC2	0.37	-0.01	-0.16	0.21	0.19	0.10	0.27	0.23	0.26	0.47	0.13	0.15	0.24	0.18	-0.25	0.23	0.26	0.51	-0.96	Stable	12.00
MARCHF10	-0.06	-0.02	0.04	0.12	0.01	-0.16	-0.06	0.06	0.02	0.12	-0.11	0.14	-0.14	-0.01	0.06	-0.06	0.77	-0.50	-1.01	Stable	12.00
MAPT	0.53	0.85	0.75	0.65	0.64	0.24	0.77	1.18	1.72	2.14	0.59	1.52	0.51	0.77	1.49	1.01	0.72	1.11	0.14	Stable	12.00
ENSSSCG00000017313	-0.07	-0.18	-0.14	-0.03	-0.18	0.00	-0.17	-0.36	-0.19	-0.16	-0.10	-0.34	-0.18	-0.19	-0.18	-0.35	0.77	1.10	0.13	Stable	12.00
ACBD4	-0.31	-0.56	-0.59	-0.48	-0.15	0.07	-0.14	-0.35	-0.34	-0.18	-0.29	-0.12	-0.20	-0.37	-0.18	-0.41	0.12	1.62	0.70	Stable	12.00
DCAKD	0.02	0.12	0.15	-0.03	-0.06	0.08	0.00	0.34	0.34	0.34	-0.03	0.13	-0.09	0.23	0.38	-0.06	0.05	5.38	2.43	Up	12.00
CCDC43	0.47	0.37	0.22	0.57	0.58	0.02	0.43	0.20	0.49	0.18	0.11	0.35	0.53	0.28	0.11	0.26	0.96	0.99	-0.02	Stable	12.00
ENSSSCG00000017383	0.17	-0.27	0.61	0.64	0.56	0.98	0.22	0.30	-0.15	0.39	0.52	0.35	0.86	0.67	-0.18	0.41	0.02	0.29	-1.78	Down	12.00
CNTNAP1	-0.49	-0.67	-0.13	-0.28	-0.28	-0.56	-0.51	-0.61	-0.83	-0.82	-0.27	-0.73	-0.19	-0.25	-0.45	-0.70	0.92	1.02	0.03	Stable	12.00
CNP	0.16	0.20	0.23	0.37	0.14	0.41	0.17	0.00	0.00	0.27	0.19	0.12	0.14	0.25	0.00	0.11	0.13	0.59	-0.77	Stable	12.00

HAP1	-0.36	0.01	-0.07	-0.06	-0.25	-0.26	-0.21	-0.54	-0.52	-0.43	-0.34	-0.20	0.05	0.07	-0.51	-0.29	0.67	1.20	0.27	Stable	12.00
JUP	-0.10	-0.11	-0.20	-0.26	-0.24	-0.27	-0.01	-0.24	-0.16	-0.18	-0.22	-0.09	-0.17	-0.29	-0.04	-0.24	0.13	0.69	-0.54	Stable	12.00
ENSSSCG00000017446	-0.14	0.24	-0.14	-0.55	0.02	-1.21	-0.04	-0.26	-0.13	-0.64	-0.78	-0.14	-0.22	-0.55	0.11	0.16	0.12	0.27	-1.89	Stable	12.00
TNS4	-0.06	-0.18	-0.13	-0.56	-0.30	-0.22	0.05	-0.10	-0.61	-0.51	-0.06	0.08	0.60	0.71	-0.37	0.10	0.91	0.79	-0.33	Stable	12.00
MED1	0.00	0.23	0.00	0.00	0.00	0.00	0.23	0.00	0.00	0.23	-0.01	0.23	0.00	0.00	0.00	0.00	0.99	1.01	0.01	Stable	12.00
CACNB1	0.10	0.19	0.03	0.10	0.18	0.10	0.10	0.10	0.00	0.10	0.19	0.10	0.55	0.19	0.00	0.28	0.10	0.44	-1.18	Stable	12.00
OSBPL7	0.00	0.04	0.17	0.00	0.15	0.07	-0.01	0.22	0.00	0.00	0.03	-0.01	0.00	0.15	0.00	0.00	0.31	2.31	1.21	Stable	12.00
LRRC46	-0.02	0.26	0.08	0.02	-0.01	0.11	-0.06	0.35	-0.07	0.42	0.12	-0.06	-0.03	0.33	-0.02	-0.07	0.64	1.66	0.73	Stable	12.00
SCRN2	0.09	0.34	0.07	0.27	0.16	0.54	0.00	0.05	-0.17	0.21	0.32	-0.10	0.48	0.17	-0.06	-0.17	0.17	0.29	-1.78	Stable	12.00
SP6	-0.03	0.35	0.20	0.06	-0.03	0.05	-0.10	0.10	-0.10	0.03	0.16	-0.10	0.00	0.25	0.00	-0.09	0.30	9.00	3.17	Stable	12.00
CDK5RAP3	-0.46	-0.29	-0.45	-0.11	-0.70	-0.48	-0.03	-0.15	-0.27	-0.84	-0.37	0.05	-0.50	-0.68	-0.39	-0.74	0.38	0.74	-0.44	Stable	12.00
TTLL6	0.52	0.56	0.59	0.36	0.61	1.16	-0.05	0.49	-0.14	0.94	0.75	0.24	0.59	1.60	0.42	0.77	0.42	0.73	-0.44	Stable	12.00
NGFR	0.09	-0.27	0.03	0.24	-0.31	-0.17	-0.09	0.14	-0.13	-0.97	0.03	0.09	-0.43	-0.02	-0.22	-0.40	0.25	0.25	-2.03	Stable	12.00
NXPH3	-0.30	-0.41	-0.42	-0.06	-0.75	-0.49	-0.62	-0.51	-0.57	-0.80	-0.21	-0.44	-0.69	-0.60	-0.40	-0.60	0.80	0.95	-0.08	Stable	12.00
RSAD1	0.15	-0.14	0.45	0.88	0.35	0.16	0.34	0.35	0.19	0.26	0.45	-0.21	0.59	0.78	0.56	0.51	0.79	0.89	-0.16	Stable	12.00
XYLT2	-0.31	-0.32	-0.18	-0.26	-0.29	-0.34	-0.03	0.30	0.07	-0.53	-0.23	0.04	-0.13	0.01	-0.29	-0.26	0.13	0.37	-1.42	Stable	12.00
SGCA	0.74	1.31	0.55	-0.65	0.16	0.47	0.56	0.48	-0.08	0.79	-0.05	0.81	-0.11	-0.09	-0.05	-0.38	0.26	3.30	1.72	Stable	12.00
ENSSSCG00000017608	0.55	0.82	-0.60	0.57	0.06	0.02	0.53	0.39	0.22	0.67	0.22	0.40	0.62	-0.17	0.32	-0.60	0.95	1.05	0.07	Stable	12.00
COIL	0.00	-0.05	0.00	-0.05	-0.05	-0.05	0.00	-0.05	-0.05	0.00	-0.05	0.00	-0.09	-0.05	-0.05	0.00	0.69	0.83	-0.26	Stable	12.00
TRIM25	0.13	0.07	-0.14	-0.24	-0.16	-0.15	-0.25	-0.25	-0.23	-0.16	0.01	-0.07	-0.31	-0.03	-0.17	0.11	0.84	0.88	-0.19	Stable	12.00
DGKE	0.19	0.13	0.04	-0.11	-0.08	-0.11	0.13	0.03	0.03	0.09	-0.04	0.06	-0.06	0.03	0.03	0.07	0.02	-3.34	1.74	Up	12.00
SCPEP1	0.24	-0.14	-0.45	-0.36	-0.14	0.18	-0.46	0.26	0.33	0.02	-0.38	0.37	-0.97	0.64	0.23	0.15	0.30	-0.56	-0.82	Stable	12.00
CUEDC1	-0.39	-0.28	-0.12	-0.26	-0.21	-0.34	-0.16	-0.16	-0.28	-0.21	-0.38	-0.30	-0.17	-0.10	-0.13	-0.11	0.36	0.82	-0.29	Stable	12.00
TSPOAP1	0.10	0.04	-0.25	-0.10	-0.62	-0.08	0.21	-0.20	0.07	-0.29	-0.09	0.09	-0.02	-0.04	-0.06	-0.50	0.10	0.09	-3.48	Stable	12.00
ENSSSCG00000017640	-0.13	0.00	-0.09	-0.21	0.09	0.53	0.24	-0.13	-0.13	0.09	0.00	-0.16	-0.24	-0.13	-0.09	-0.13	0.61	22.33	4.48	Stable	12.00
DHX40	0.01	0.26	-0.01	0.15	-0.06	-0.13	0.08	-0.06	-0.06	-0.13	0.04	0.43	0.00	0.43	0.00	0.43	0.92	0.89	-0.17	Stable	12.00
RNFT1	-0.09	-0.08	-0.56	0.74	-0.28	-0.30	-0.10	0.33	0.10	-0.03	0.32	-0.87	0.27	-0.63	-0.03	-0.57	0.84	1.49	0.57	Stable	12.00
DHRS11	-0.22	-0.12	-0.23	0.23	-0.07	-0.20	-0.07	0.04	-0.12	-0.03	0.00	-0.08	-0.22	-0.23	-0.19	-0.18	0.26	2.07	1.05	Stable	12.00
UNC45B	-0.02	0.30	0.01	0.99	0.58	0.88	-0.13	0.46	0.52	0.57	1.11	0.03	-0.03	0.14	0.20	0.14	0.08	0.35	-1.53	Stable	12.00
C17orf75	-0.52	-0.47	-0.41	-0.69	-0.56	-0.83	-0.67	-0.65	-0.88	-0.69	-0.69	-0.53	-0.70	-0.06	-0.71	-0.50	0.32	0.84	-0.24	Stable	12.00
RHBDL3	0.42	0.15	0.03	0.11	-0.01	0.40	0.10	0.30	0.44	0.26	0.22	0.24	0.20	-0.01	0.21	-0.01	0.72	1.16	0.22	Stable	12.00
NLK	-0.91	-1.10	-1.15	-0.81	-0.92	-0.88	-1.23	-0.89	-1.14	-0.54	-1.23	-0.78	-1.47	-1.38	-0.35	-1.09	0.71	1.06	0.08	Stable	12.00
UNC119	-0.40	-0.50	-0.11	-0.45	-0.19	-0.43	-0.09	-0.16	-0.24	-0.48	-0.27	-0.44	-0.19	-0.03	-0.12	-0.23	0.11	0.62	-0.69	Stable	12.00
RAB34	-0.02	-0.16	-0.17	-0.02	-0.15	0.10	-0.15	-0.05	-0.08	-0.02	0.10	-0.02	0.14	-0.20	-0.05	-0.17	0.04	28.03	4.81	Up	12.00
SDF2	-0.24	0.22	0.27	-0.23	0.30	-0.25	0.31	-0.19	-0.22	-0.23	-0.25	-0.24	-0.25	0.66	-0.14	0.26	0.20	-0.74	-0.44	Stable	12.00
FLOT2	-0.03	-0.57	0.13	0.18	-0.35	0.14	-0.11	0.24	0.25	0.01	-0.17	0.06	0.16	-0.58	0.17	-0.08	0.69	7.59	2.92	Stable	12.00
ABHD15	-0.72	0.07	0.17	-0.65	-0.06	-0.71	-0.35	-0.13	-0.32	-0.62	-0.68	-0.72	-0.75	0.16	-0.01	-0.08	0.02	0.27	-1.91	Down	12.00
CORO6	0.41	0.52	0.46	0.38	0.43	0.51	0.54	0.55	0.15	0.38	0.47	0.36	-0.11	0.92	0.15	0.53	0.42	1.25	0.33	Stable	12.00
EFCAB5	-0.34	0.21	0.01	-0.35	0.35	-0.26	0.10	0.01	-0.18	-0.33	-0.35	-0.34	-0.29	0.37	0.05	0.29	0.17	-0.18	-2.47	Stable	12.00
BHLHA9	0.30	0.21	-0.04	0.45	0.10	0.18	0.30	0.48	0.38	0.39	0.17	0.36	0.05	0.13	-0.16	0.16	0.72	0.86	-0.23	Stable	12.00
TIMM22	-0.11	-0.11	-0.01	-0.13	-0.10	-0.11	-0.02	-0.07	-0.14	-0.11	-0.08	-0.16	-0.01	-0.02	-0.22	-0.07	0.76	0.90	-0.15	Stable	12.00
GEMIN4	-0.58	-0.52	-0.40	-0.58	-0.40	-0.51	-0.52	-0.41	-0.13	-0.18	0.17	-0.47	-0.46	-0.41	-0.33	-0.48	0.64	1.13	0.18	Stable	12.00
SGSM2	-0.16	-0.10	-0.03	-0.26	0.00	-0.11	-0.01	-0.18	-0.27	-0.91	-0.09	-0.17	-0.08	0.00	-0.20	0.00	0.48	0.59	-0.76	Stable	12.00
ENSSSCG00000017868	-0.14	0.10	-0.48	0.26	0.01	0.18	-0.06	-0.27	0.22	-0.41	0.12	0.07	-0.12	0.01	-0.10	0.01	0.36	-6.63	2.73	Stable	12.00
CAMKK1	0.20	0.06	0.17	-0.22	0.47	-0.01	0.35	0.11	-0.03	-0.09	0.09	0.10	0.40	0.50	0.24	0.47	0.68	1.31	0.39	Stable	12.00

CYB5D2	0.07	-0.28	0.07	0.09	-0.52	-0.22	-0.39	-0.05	-0.01	-0.10	0.32	-0.31	-0.08	-0.40	-0.09	-0.47	0.83	0.83	-0.27	Stable	12.00
WSCD1	-0.04	0.16	-0.12	-0.07	0.33	-0.04	0.34	0.10	0.04	0.11	-0.54	-0.22	0.18	0.75	0.24	0.43	0.28	8.97	3.17	Stable	12.00
ENSSSCG00000017897	-0.14	-0.45	-0.12	0.08	-0.28	0.02	-0.88	-0.20	-0.01	0.19	-0.06	-0.44	-0.10	-0.42	-0.19	-0.32	0.15	2.67	1.42	Stable	12.00
GLTPD2	0.43	0.14	0.48	0.22	0.26	0.42	0.27	0.17	0.49	0.55	0.10	0.29	0.07	0.20	0.15	0.32	0.88	1.04	0.06	Stable	12.00
VMO1	0.21	0.42	0.49	0.29	0.21	0.29	0.62	0.37	0.69	0.91	0.55	0.84	0.12	0.31	0.03	0.53	0.58	0.84	-0.25	Stable	12.00
ENSSSCG00000017920	-0.22	-0.45	-0.27	-0.19	-0.53	-0.30	-0.46	-0.11	-0.34	-0.24	-0.18	-0.19	-0.15	-0.71	-0.12	-0.53	0.62	1.16	0.21	Stable	12.00
ZMYND15	0.02	0.00	0.24	0.00	0.00	0.00	0.02	0.00	0.00	0.00	0.00	0.00	0.00	0.00	0.02	0.00	0.26	Inf	Inf	Stable	12.00
ENSSSCG00000017934	0.48	0.21	-0.10	0.22	0.11	0.35	0.33	0.17	0.32	0.33	0.23	0.35	0.15	0.19	0.24	0.13	0.97	0.99	-0.02	Stable	12.00
CTDNEP1	0.00	0.00	0.19	0.00	0.00	0.00	0.00	0.00	0.00	0.00	0.00	0.00	0.00	0.00	0.00	0.00	0.35	Inf	Inf	Stable	12.00
SAT2	-0.84	-0.39	-0.08	-0.87	-0.18	-0.47	-0.34	-0.78	-0.46	-0.75	-0.85	-0.62	-0.59	-0.02	-0.38	-0.23	0.27	0.72	-0.46	Stable	12.00
ALOX12B	0.18	0.03	0.15	0.01	0.18	0.06	0.18	-0.22	0.06	0.00	-0.09	0.00	0.00	0.14	0.17	0.07	0.33	3.04	1.60	Stable	12.00
CCDC42	-0.27	0.04	0.12	-0.06	0.04	-0.11	-0.04	-0.44	-0.19	-0.35	-0.04	-0.12	-0.27	0.12	-0.19	-0.03	0.90	0.91	-0.14	Stable	12.00
PIK3R5	0.43	0.38	0.24	0.64	0.42	0.74	0.37	-0.09	0.37	0.22	0.79	0.71	0.18	0.19	0.31	0.42	0.04	0.54	-0.90	Stable	12.00
DHRS7C	0.03	0.03	0.03	0.03	0.06	0.06	0.03	0.05	0.09	0.09	0.03	0.03	0.09	0.03	0.03	0.06	0.16	0.72	-0.47	Stable	12.00
GLP2R	0.03	-0.01	0.02	-0.03	0.19	0.19	0.06	-0.03	0.04	0.11	-0.03	0.00	0.14	0.19	0.09	0.19	0.33	0.54	-0.89	Stable	12.00
MYH3	-0.25	-0.20	-0.29	-0.38	-0.37	-0.37	-0.22	-0.38	-0.22	-0.26	-0.25	-0.25	-0.28	-0.40	-0.33	-0.32	0.48	0.92	-0.12	Stable	12.00
DNAH9	-0.21	-0.10	-0.15	-0.22	-0.18	-0.39	-0.25	-0.22	-0.23	-0.25	-0.35	-0.24	-0.20	0.04	-0.18	0.00	0.25	0.71	-0.48	Stable	12.00
ENSSSCG00000018018	0.00	0.00	0.00	-0.04	0.00	-0.04	0.00	0.00	0.00	0.00	-0.07	0.00	-0.04	0.00	0.00	-0.04	0.02	-0.03	-5.27	Down	6.00
ENSSSCG00000018027	-1.23	-1.07	-0.27	-0.84	-1.36	-0.99	-1.11	-1.62	-1.58	-1.27	-0.74	-0.73	-0.58	-0.24	-1.36	-1.27	0.69	1.09	0.13	Stable	12.00
TEKT3	-0.99	-0.92	-0.25	-0.90	-1.00	-0.98	-0.86	-0.89	-0.95	-0.84	-1.06	-0.88	-0.84	-0.16	-0.57	-1.12	0.07	0.73	-0.45	Stable	12.00
UBB	0.10	0.03	0.00	0.09	0.12	0.14	0.03	0.09	0.09	0.16	0.13	0.09	0.07	0.20	0.16	0.19	0.24	0.72	-0.48	Stable	12.00
ALDH3A2	0.17	-0.09	0.83	0.10	0.01	0.24	0.07	0.40	0.31	0.35	0.22	0.12	0.24	0.85	0.23	0.05	0.19	2.07	1.05	Stable	12.00
SLC47A2	0.40	-0.26	0.04	0.31	0.34	0.41	-0.26	0.30	0.25	0.82	0.60	0.15	0.26	0.28	1.15	0.44	0.33	0.57	-0.80	Stable	12.00
ALDH3A1	-0.08	0.70	0.09	1.02	0.77	0.72	0.68	0.28	0.05	0.27	0.69	0.55	0.92	1.11	-0.45	0.57	0.07	0.43	-1.21	Stable	12.00
ULK2	1.17	0.95	-0.45	1.08	1.42	1.45	0.81	1.24	1.29	1.38	0.82	1.28	0.96	-0.48	1.72	1.49	0.17	0.63	-0.66	Stable	12.00
ENSSSCG00000018046	-1.16	-0.62	-0.27	-0.80	-0.81	-0.91	-0.63	-0.87	-1.06	-1.20	-0.81	-0.91	-0.91	-0.85	-0.73	-0.81	0.29	0.86	-0.21	Stable	12.00
ENSSSCG00000020686	-0.42	-0.54	-0.49	-0.54	-0.54	-0.41	-0.21	-0.54	-0.54	-0.54	-0.54	-0.27	-0.54	-0.62	-0.48	-0.27	0.72	1.05	0.07	Stable	16.00
ENSSSCG00000020706	0.04	0.04	0.04	0.04	0.08	0.04	0.00	0.00	-0.02	-0.01	0.08	0.08	0.04	0.04	0.08	0.04	0.21	0.58	-0.79	Stable	13.00
FAM160A1	0.00	-0.11	0.23	0.23	-0.22	-0.22	-0.11	-0.22	-0.08	0.04	-0.22	-0.11	-0.22	-0.11	-0.11	-0.22	0.49	0.54	-0.90	Stable	8.00
DUSP3	-0.23	0.26	0.34	0.49	0.62	-0.02	0.61	0.02	0.19	0.30	0.12	-0.23	0.57	0.27	0.08	0.23	0.64	0.75	-0.42	Stable	12.00
NQO2	-0.29	-0.15	-0.15	-0.15	0.24	0.01	-0.37	0.06	-0.30	-0.15	-0.15	0.00	-0.15	-0.21	-0.29	-0.07	0.03	4.17	2.06	Up	7.00
SLC41A1	0.15	0.09	0.49	-0.08	0.18	0.17	0.28	0.03	0.21	0.46	-0.08	0.39	0.28	0.20	0.01	0.28	0.84	0.91	-0.14	Stable	9.00
BRPF3	0.53	-0.11	-0.28	0.31	0.40	0.04	-1.00	-0.33	-0.25	-0.44	0.14	0.06	0.26	0.47	0.23	0.33	0.28	-0.68	-0.55	Stable	7.00
BSCL2	0.38	0.55	0.20	0.59	0.47	0.37	0.24	0.24	0.43	0.24	0.65	0.27	0.27	0.34	0.92	0.25	0.81	1.06	0.09	Stable	2.00
ENSSSCG00000020837	0.07	0.24	0.15	-0.12	0.48	-0.13	0.13	-0.39	0.02	-0.33	0.25	0.13	0.75	0.18	0.13	1.28	0.29	0.22	-2.16	Stable	6.00
TAF13	-0.24	0.11	0.32	-0.04	-0.03	-0.26	-0.06	-0.36	0.08	-0.36	-0.03	0.01	-0.16	-0.04	-0.13	-0.08	0.40	0.35	-1.53	Stable	4.00
SCARA3	0.40	0.06	0.39	0.31	0.29	0.18	0.38	0.55	0.83	0.29	0.26	0.41	0.21	0.48	-0.05	0.08	0.24	1.51	0.60	Stable	14.00
HECTD2	-1.49	-0.66	-0.77	-0.15	-0.78	-0.34	-0.89	-0.85	-0.98	-0.42	-0.68	-0.60	-0.24	-0.27	-1.43	-0.90	0.04	1.78	0.83	Stable	14.00
EPDR1	-0.12	-0.34	0.00	-0.34	-0.19	-0.24	-0.21	-0.47	-0.52	-0.30	-0.55	-0.45	-0.40	-0.34	-0.25	-0.35	0.36	0.80	-0.32	Stable	9.00
WDR31	0.17	0.48	0.27	-0.09	0.01	0.15	0.27	-0.02	0.03	0.32	-0.06	0.29	-0.10	0.33	0.18	-0.04	0.08	3.58	1.84	Stable	1.00
ENSSSCG00000020988	-0.12	0.55	0.22	0.09	0.04	0.46	-0.12	0.15	0.41	0.26	0.46	-0.12	0.24	0.26	-0.12	-0.04	0.87	0.88	-0.18	Stable	6.00
DNAH12	0.00	0.00	0.00	-0.03	0.00	0.00	0.00	0.00	0.00	0.00	0.00	-0.03	-0.05	0.00	0.00	0.00	0.10	0.00	#NAME?	Stable	13.00
RAB3B	0.02	0.25	0.35	0.61	0.08	0.15	0.43	0.56	0.50	0.15	0.48	0.27	0.58	0.25	0.35	0.33	0.95	1.02	0.03	Stable	6.00
SLC26A11	-0.51	-0.25	0.06	-0.02	-0.50	-0.59	-0.48	0.16	-0.24	-0.11	-0.13	0.37	-0.36	-0.21	0.05	-0.09	1.00	1.00	0.00	Stable	12.00
ENSSSCG00000021041	-0.27	0.06	-0.40	0.54	0.14	0.00	-0.32	-0.30	0.05	-0.57	0.35	-0.83	0.11	-0.33	0.50	0.62	0.44	-2.92	1.54	Stable	9.00

BRWD1	0.01	0.02	0.01	0.04	-0.01	0.04	0.00	-0.15	0.03	-0.13	-0.01	-0.01	-0.01	-0.01	-0.01	-0.01	0.96	0.89	-0.17	Stable	13.00
GIPR	-0.16	-0.27	-0.04	-0.35	-0.11	-0.06	-0.09	-0.91	-0.59	-0.36	0.13	-0.09	-0.33	-0.25	-0.09	-0.48	0.48	1.45	0.54	Stable	6.00
BLVRB	-0.05	0.12	-0.04	0.04	-0.05	0.04	-0.09	0.09	0.09	0.04	0.04	-0.09	-0.05	0.04	-0.09	-0.09	0.58	-0.68	-0.55	Stable	6.00
BSPRY	0.39	0.78	1.39	-0.17	-0.20	-0.28	-0.28	0.08	-0.22	-0.19	-0.21	0.44	0.71	-0.05	0.63	-0.18	0.17	-36.54	5.19	Stable	1.00
DHCR7	-0.89	-1.18	-0.58	-0.85	-0.40	-0.52	-1.51	-0.28	-0.36	-1.35	-1.16	-0.99	-0.44	-1.47	-0.75	-0.85	0.79	1.07	0.10	Stable	2.00
SYNC	-0.11	-0.15	-0.07	-0.01	0.19	0.33	0.29	0.09	0.32	0.18	0.16	0.25	-0.01	-0.44	0.29	0.12	0.26	0.18	-2.50	Stable	6.00
TRDMT1	-0.10	-0.13	-0.06	-0.03	-0.25	-0.03	-0.15	-0.32	0.00	-0.06	0.00	-0.10	-0.03	-0.10	-0.10	-0.32	0.73	1.18	0.24	Stable	10.00
MCFD2	-1.16	0.03	-0.56	0.15	-0.25	0.12	-0.99	0.06	-0.56	-0.94	-0.03	-0.50	-0.11	-0.37	-0.38	-0.47	0.26	1.94	0.96	Stable	3.00
CHD3	-0.17	-0.07	-0.02	-0.26	-0.04	-0.21	-0.11	-0.16	-0.16	-0.09	-0.19	-0.09	-0.16	0.06	-0.33	-0.12	0.62	0.83	-0.27	Stable	12.00
ENSSSCG00000021414	-0.30	-0.71	-0.35	-0.29	0.19	0.88	-0.40	-0.68	0.27	-0.39	0.02	-0.10	-0.56	-0.13	0.39	0.91	0.21	-2.92	1.55	Stable	2.00
DCTN4	-0.58	-0.34	-0.80	-1.04	-0.44	-0.55	-0.58	-0.16	-0.27	-0.34	-0.85	-0.52	-0.41	-0.53	0.03	-0.38	0.23	0.71	-0.49	Stable	2.00
GPSM2	-0.18	-0.13	0.00	-0.07	-0.42	-0.32	-0.09	-0.28	-0.04	-0.28	-0.02	-0.22	-0.23	-0.15	-0.16	-0.42	0.08	0.53	-0.93	Stable	4.00
ENSSSCG00000021448	0.37	0.68	0.21	0.12	0.64	1.33	0.85	0.02	1.37	0.83	0.02	0.81	0.14	0.61	0.06	0.07	0.92	1.05	0.07	Stable	6.00
SNF8	-0.51	-0.06	-0.11	-0.26	-0.14	-0.43	-0.20	-0.65	-0.14	-0.49	-0.16	-0.16	-0.29	-0.48	-0.47	-0.04	0.43	1.32	0.40	Stable	12.00
SLC30A3	-0.15	-0.39	0.02	-0.15	-0.14	-0.08	0.16	-0.24	-0.30	-0.24	-0.39	-0.03	-0.15	0.09	-0.40	0.06	0.89	1.09	0.13	Stable	3.00
KCNJ5	0.22	0.29	-0.05	0.25	0.16	0.12	0.56	0.38	0.17	-0.22	0.43	0.38	0.26	0.04	0.02	0.10	0.86	1.10	0.14	Stable	9.00
ENSSSCG00000021663	-0.06	0.22	0.11	-0.52	-0.17	-0.01	-0.25	0.00	0.33	-0.32	0.22	-0.22	0.34	0.07	-0.05	-0.02	0.28	-0.53	-0.92	Stable	5.00
ZDHHC13	-0.11	-0.04	0.02	0.22	-0.06	-0.09	-0.08	0.28	0.08	0.22	0.23	0.24	0.02	0.11	0.07	0.07	0.33	0.39	-1.36	Stable	2.00
NRSN1	0.91	1.10	0.93	0.45	0.52	0.13	1.72	0.56	0.97	0.44	0.50	0.16	0.13	0.58	0.46	0.37	0.00	2.67	1.42	Up	7.00
XRRA1	0.34	0.59	0.87	0.33	0.25	0.37	0.60	0.48	0.72	0.51	0.59	0.39	0.51	0.16	0.52	0.50	0.28	1.23	0.30	Stable	9.00
ENSSSCG00000021744	-0.42	-0.61	-0.31	-0.23	-0.32	-0.24	-0.24	-0.31	-0.45	-0.32	-0.24	-0.24	-0.24	-0.34	-0.41	-0.25	0.02	1.49	0.58	Stable	4.00
ZNF341	-0.02	0.08	0.01	-0.48	-0.56	-0.39	-0.15	0.39	-0.14	-0.37	-0.25	0.05	0.25	-0.06	0.35	-0.60	0.02	-0.19	-2.40	Down	17.00
TBCK	0.56	0.69	0.53	0.63	0.69	0.69	0.52	0.69	0.69	0.56	0.69	0.69	0.51	0.59	0.69	0.53	0.92	0.99	-0.01	Stable	8.00
ENSSSCG00000021815	-0.03	0.22	0.10	0.01	0.43	0.10	0.20	0.11	0.13	0.13	0.01	0.17	0.05	0.07	0.22	0.48	0.54	0.74	-0.44	Stable	10.00
RPS21	-0.08	-0.12	-0.07	-0.11	-0.12	0.02	-0.16	-0.12	-0.41	-0.07	-0.04	-0.37	-0.26	-0.12	-0.16	-0.40	0.85	0.92	-0.11	Stable	17.00
TAPT1	-0.03	-0.26	-0.91	-0.20	-0.19	-0.04	0.03	-0.22	-0.30	0.13	-0.24	-0.35	-0.52	-0.27	-0.64	-0.11	0.32	1.71	0.78	Stable	8.00
FAM124B	-0.14	-0.20	-0.26	-0.20	-0.26	-0.20	-0.26	-0.04	-0.25	-0.26	-0.20	-0.26	-0.20	-0.25	-0.32	-0.20	0.85	0.97	-0.04	Stable	15.00
EDDM3B	0.72	0.40	0.43	0.14	-0.16	0.18	0.35	0.51	0.72	0.47	0.14	0.41	-0.15	0.72	0.66	0.12	0.00	3.93	1.98	Up	7.00
FDXACB1	-0.35	-0.35	-0.35	-0.35	0.05	0.76	-0.35	-0.35	-0.35	-0.35	-0.13	0.21	-0.35	-0.01	0.60	-0.35	0.51	2.99	1.58	Stable	9.00
NDRG4	0.21	0.22	0.14	0.14	0.19	0.09	0.46	0.87	1.11	0.25	0.19	0.23	-0.18	0.26	0.23	0.10	0.05	3.47	1.79	Up	6.00
LY75	-0.17	-0.14	0.00	-0.34	-0.17	-0.33	0.00	-0.12	-0.17	-0.05	-0.34	-0.17	-0.34	0.00	-0.21	-0.34	0.01	0.39	-1.37	Down	15.00
ENSSSCG00000021998	0.45	0.18	0.18	0.19	0.19	0.09	0.45	0.22	0.00	0.09	0.09	0.22	0.19	0.05	0.01	0.18	0.60	1.23	0.29	Stable	5.00
REEP5	-0.32	0.02	-0.02	-0.09	-0.09	-0.01	-0.43	0.02	-0.34	-0.13	0.01	-0.09	-0.02	0.02	-0.08	-0.04	0.26	2.41	1.27	Stable	2.00
RHOA	0.27	0.14	0.23	0.19	0.09	-0.01	0.27	0.11	0.11	0.24	0.21	0.22	0.25	0.27	0.27	0.27	0.56	1.14	0.19	Stable	13.00
TP53INP2	0.01	0.19	0.39	0.39	0.39	0.19	0.20	0.39	0.39	0.19	0.39	0.19	0.39	0.39	0.34	0.39	0.67	0.91	-0.13	Stable	17.00
SULT1C2	0.04	0.50	-0.27	-0.10	0.48	-0.02	0.34	0.33	0.57	0.18	0.30	0.16	0.49	0.01	0.35	0.50	0.91	0.94	-0.09	Stable	3.00
ZNF251	-0.24	-0.61	-0.69	-1.03	-0.36	-0.66	-0.22	0.18	0.02	-0.04	0.19	-0.74	-0.14	-0.01	-0.42	-0.51	0.38	0.60	-0.73	Stable	4.00
RAB11FIP5	-0.03	-0.08	-0.22	-0.41	-0.17	-0.36	-0.15	-0.21	-0.51	-0.16	-0.30	-0.11	-0.41	0.02	-0.03	-0.29	0.11	0.54	-0.88	Stable	3.00
CISD3	-0.17	0.29	0.28	0.20	0.31	-0.02	1.26	-0.26	0.45	-0.18	-0.10	1.12	-0.31	0.00	0.18	0.69	0.87	1.19	0.25	Stable	12.00
NCAPD3	0.00	0.00	0.00	0.06	0.00	0.00	0.00	0.00	0.12	0.12	0.08	0.00	0.29	0.00	0.00	0.29	0.08	0.14	-2.84	Stable	9.00
CCT6B	0.32	0.05	0.20	0.45	0.52	0.38	0.24	0.25	0.31	0.43	0.35	0.39	0.18	0.02	0.20	0.27	0.01	0.53	-0.92	Stable	12.00
TAF8	-0.37	-0.22	-0.09	-0.30	-0.30	0.02	-0.05	-0.05	-0.22	-0.02	0.04	-0.24	-0.30	-0.35	0.07	-0.26	0.90	0.94	-0.09	Stable	7.00
SPACA9	-0.14	-0.20	-1.21	0.40	0.46	0.24	0.64	0.64	0.18	-0.01	0.64	0.11	0.64	0.46	-1.11	-0.17	0.20	-0.33	-1.61	Stable	1.00
ENSSSCG00000022258	-0.02	0.11	0.07	-0.08	-0.05	-0.27	0.19	0.15	0.25	0.19	-0.27	0.17	-0.02	0.05	0.20	-0.05	0.03	-2.67	1.42	Up	17.00
DACT3	-0.10	0.00	0.00	0.00	0.00	-0.10	0.00	0.00	0.00	-0.10	0.00	0.00	-0.10	-0.10	0.00	0.00	0.62	0.67	-0.58	Stable	6.00

BDH2	0.38	0.40	0.34	0.51	0.53	0.67	0.38	0.51	0.67	0.60	0.47	0.67	0.45	0.21	0.63	0.36	0.19	0.83	-0.27	Stable	8.00
UMODL1	0.40	-0.05	-0.06	0.34	-0.01	-0.03	0.15	0.22	-0.14	0.26	0.15	0.48	0.08	0.40	0.03	-0.17	0.86	0.87	-0.21	Stable	13.00
NPL	0.00	0.00	0.00	0.00	0.00	0.00	0.00	0.00	0.00	0.00	0.18	0.00	0.00	0.00	0.00	0.00	0.35	0.00	#NAME?	Stable	9.00
DNAI4	0.05	-0.16	-0.40	-0.42	-0.37	-0.51	-0.40	-0.53	-0.14	-0.44	-0.49	-0.36	-0.37	-0.14	-0.53	-0.49	0.09	0.65	-0.63	Stable	6.00
ENSSSCG00000022401	0.02	-0.32	-0.29	0.08	0.14	0.11	-0.22	0.00	-0.22	-0.12	0.11	-0.22	-0.02	-0.02	-0.03	0.01	0.05	-15.12	3.92	Stable	6.00
TIRAP	0.22	0.37	0.31	0.89	0.83	0.87	0.47	-0.02	0.50	0.70	0.33	0.43	0.87	0.06	0.16	0.79	0.00	0.36	-1.46	Down	9.00
KAZALD1	0.09	0.15	0.51	0.46	0.40	0.19	0.40	0.26	-0.25	0.18	0.36	-0.04	0.21	0.24	0.13	0.37	0.46	0.72	-0.48	Stable	14.00
SLC30A5	0.63	0.71	0.97	0.37	0.32	0.69	1.25	1.04	0.59	0.76	0.64	0.94	0.93	1.32	1.00	0.17	0.03	1.56	0.64	Stable	16.00
PAF1	0.25	-0.03	0.12	-0.26	-0.33	-0.17	0.05	0.35	0.05	0.23	-0.24	0.05	-0.13	0.23	0.05	-0.25	0.01	-0.97	-0.04	Stable	6.00
TEX12	-1.11	-0.22	-1.38	-0.36	-0.45	-0.08	-1.60	-1.02	-0.40	-0.95	-1.06	0.41	-0.41	0.21	-0.28	-1.02	0.43	1.48	0.57	Stable	9.00
WDR73	0.00	-0.13	-0.03	-0.20	-0.16	-0.14	-0.16	-0.16	-0.32	-0.09	-0.14	-0.14	-0.24	0.00	-0.17	-0.03	0.64	0.85	-0.24	Stable	7.00
STK39	0.21	0.71	0.65	0.43	0.65	-0.23	0.68	0.51	0.65	0.41	0.50	0.35	0.50	0.65	0.25	0.37	0.18	1.44	0.53	Stable	15.00
CDON	0.48	0.03	0.79	0.05	1.01	0.48	1.00	-0.20	0.15	0.82	0.12	0.10	-0.02	0.00	-0.12	0.96	0.44	0.61	-0.72	Stable	9.00
POLR2H	0.13	0.19	0.14	-0.11	0.21	0.16	0.01	0.22	0.00	0.00	0.21	-0.06	-0.18	0.04	0.36	0.01	0.14	4.43	2.15	Stable	13.00
MARK4	-0.64	-0.45	-0.39	-0.40	0.29	-0.23	-0.25	-0.30	-0.49	-0.50	0.02	-0.25	-0.15	-0.29	-0.25	-0.17	0.06	2.21	1.14	Stable	6.00
CAPRIN2	-0.37	-0.22	-0.12	-0.80	-0.49	-0.07	-0.45	0.00	-0.42	-0.71	-0.69	-0.52	-0.50	-0.22	-0.19	-0.51	0.01	0.46	-1.11	Down	5.00
DENND5B	-0.21	-0.04	0.04	-0.09	-0.06	-0.16	-0.20	-0.04	-0.07	0.01	-0.09	-0.10	-0.09	-0.10	-0.02	-0.10	0.94	0.97	-0.05	Stable	5.00
KATNAL2	-0.38	-0.08	-0.25	-0.36	-0.28	-0.31	-0.35	-0.15	-0.06	-0.34	-0.29	-0.34	-0.34	-0.31	-0.14	-0.27	0.06	0.68	-0.56	Stable	1.00
TRPM4	-0.06	0.33	0.16	0.22	0.00	0.22	-0.12	0.11	0.27	0.19	0.07	-0.12	0.12	0.25	-0.12	0.00	0.85	1.16	0.22	Stable	6.00
COCH	0.02	-0.19	-0.07	-0.13	0.01	-0.26	-0.12	-0.16	-0.11	-0.22	-0.23	-0.07	-0.02	-0.05	-0.08	-0.18	0.38	0.72	-0.48	Stable	7.00
RINL	0.37	0.13	0.50	0.02	-0.13	-0.07	0.75	0.52	0.23	0.29	-0.22	0.60	0.01	0.21	0.60	0.03	0.01	6.22	2.64	Up	6.00
SALL2	-0.04	-0.25	0.00	-0.32	-0.18	-0.28	-0.02	0.07	-0.04	-0.08	-0.32	-0.06	-0.11	-0.04	0.04	-0.29	0.01	0.18	-2.51	Down	7.00
FLOT1	0.57	0.18	0.30	-0.13	0.30	0.14	0.54	-0.17	-0.05	-0.04	0.05	-0.03	-0.03	0.52	0.31	0.20	0.07	4.87	2.28	Stable	7.00
ENSSSCG00000022738	-0.35	-0.69	-0.40	-0.69	-0.46	-0.19	-0.59	-0.71	-0.44	-0.62	-0.37	-0.70	-0.67	-0.69	-0.69	-0.70	0.82	1.04	0.05	Stable	2.00
ENSSSCG00000022773	0.84	0.05	0.02	0.05	0.57	0.12	0.84	0.14	0.06	0.27	-0.06	0.49	-0.26	0.65	0.84	0.15	0.15	2.58	1.37	Stable	13.00
S100PBP	-0.39	-0.42	-0.37	-0.97	-0.74	-0.51	-0.46	-0.67	-0.43	-0.68	-0.48	-0.46	-0.97	-0.34	-0.22	-0.71	0.01	0.60	-0.74	Stable	6.00
ENSSSCG00000022820	0.33	0.11	-0.11	0.03	0.19	-0.02	0.09	0.11	0.06	0.17	0.34	0.23	0.17	0.26	0.13	0.20	0.50	0.74	-0.43	Stable	3.00
SLC4A3	0.00	0.12	0.06	-0.03	0.12	-0.09	0.06	0.01	0.12	0.06	-0.30	0.11	0.00	0.00	0.05	0.00	0.21	-3.09	1.63	Stable	15.00
IL2RA	-0.02	-0.17	-0.26	-0.20	-0.34	-0.04	0.00	-0.24	-0.29	0.07	-0.14	-0.08	-0.20	0.01	0.07	-0.31	0.55	0.72	-0.47	Stable	10.00
ENSSSCG00000022906	-0.08	-0.53	-0.20	-0.36	0.03	-0.31	0.11	-0.35	-0.53	-0.22	-0.19	0.11	-0.11	-0.25	0.11	-0.08	0.50	1.53	0.61	Stable	6.00
SNW1	0.73	-0.37	-1.04	-0.53	-0.33	-1.89	-1.66	0.19	-1.29	-1.72	-1.78	-0.64	-1.43	-0.88	-0.17	-2.17	0.07	0.43	-1.22	Stable	7.00
POLR2B	-0.13	-0.14	-0.29	-0.19	-0.06	-0.13	-0.13	-0.14	-0.14	-0.15	-0.15	-0.14	-0.14	-0.13	-0.14	-0.14	0.42	1.14	0.18	Stable	8.00
PARP4	0.28	0.04	-0.05	-1.62	-1.52	-1.74	-0.75	-0.41	-1.60	1.27	-0.57	-0.28	-1.53	-0.20	-0.86	-1.60	0.26	0.47	-1.10	Stable	11.00
CYB5R4	0.01	0.20	0.01	-0.28	0.01	0.01	0.01	-0.06	-0.06	0.01	-0.09	0.01	-0.22	0.01	-0.06	0.01	0.16	-0.11	-3.18	Stable	1.00
ZNF232	-0.04	0.04	-0.15	0.14	-0.03	0.14	-0.20	-0.13	-0.01	-0.16	0.14	0.05	0.00	-0.20	0.07	0.07	0.04	-1.85	0.89	Stable	12.00
CCDC149	-0.15	-0.15	0.00	-0.15	-0.15	-0.34	-0.34	-0.15	-0.04	-0.15	-0.22	-0.34	-0.52	-0.15	-0.52	-0.34	0.26	0.68	-0.55	Stable	8.00
PARP3	-0.35	-0.19	-0.45	-0.21	-0.16	-0.05	-0.40	-0.22	-0.43	-0.22	-0.37	-0.28	-0.19	-0.40	-0.40	-0.29	0.02	1.61	0.68	Stable	13.00
SSUH2	1.05	-0.34	0.09	0.94	0.43	0.23	0.77	0.68	0.48	0.70	0.19	0.75	0.54	0.56	0.18	0.21	0.74	0.88	-0.19	Stable	13.00
ENSSSCG00000023091	0.06	-0.39	-0.06	-0.10	-0.39	-0.39	0.06	-0.09	-0.21	-0.39	-0.39	-0.17	-0.39	0.04	-0.14	-0.39	0.01	0.29	-1.80	Down	4.00
OSBPL6	-0.05	-0.06	-0.03	0.06	0.00	0.04	-0.06	-0.06	-0.02	-0.02	-0.01	-0.03	0.06	-0.03	0.00	0.06	0.00	-2.18	1.12	Up	15.00
METTL23	0.04	0.26	0.01	0.29	0.44	0.53	-0.18	0.11	0.11	0.36	0.24	-0.02	0.11	0.19	0.08	-0.08	0.11	0.33	-1.59	Stable	12.00
SMIM29	-0.22	-0.17	0.05	-0.48	-0.48	-0.19	0.18	-0.15	0.14	-0.17	-0.02	-0.17	-0.48	-0.26	-0.52	-0.48	0.10	0.39	-1.37	Stable	7.00
CA10	-0.40	0.08	-0.01	-0.10	-0.20	-0.23	-0.32	0.17	-0.24	-0.30	-0.13	0.33	0.25	0.27	-0.01	0.00	0.92	1.27	0.34	Stable	12.00
VAV3	0.16	0.19	0.35	0.11	0.02	0.09	0.04	0.07	0.23	0.07	0.04	0.08	0.05	0.21	-0.13	0.02	0.18	2.25	1.17	Stable	4.00
ENSSSCG00000023195	0.33	0.14	0.20	-0.65	-0.06	-0.27	0.31	0.06	0.12	0.19	-0.43	0.31	0.04	0.62	0.31	0.09	0.02	-2.69	1.43	Up	6.00

SYN2	0.46	0.79	0.20	0.27	0.28	0.27	0.39	0.18	0.02	0.14	0.29	0.41	0.19	0.56	0.22	0.26	0.37	1.33	0.41	Stable	13.00
OPTN	0.04	0.11	0.10	-0.08	0.08	-0.18	-0.55	-0.62	-0.15	0.28	-0.21	0.09	-0.17	0.05	-0.21	0.10	0.26	12.95	3.69	Stable	10.00
MRPL45	-0.01	0.04	0.10	0.11	-0.06	0.07	0.31	0.05	-0.08	-0.02	0.17	0.24	-0.16	-0.02	-0.05	-0.16	0.77	1.84	0.88	Stable	12.00
EXOC1	-0.48	-0.48	-0.51	-0.42	-0.11	-0.30	-0.48	-0.30	-0.48	-0.48	-0.39	-0.39	-0.30	-0.30	-0.30	-0.30	0.15	1.24	0.31	Stable	8.00
CENPE	0.45	0.00	0.16	0.15	0.18	-0.15	0.01	-0.15	-0.15	-0.16	-0.15	-0.15	0.31	0.22	0.15	0.10	0.49	4.89	2.29	Stable	8.00
SCRN3	-0.31	-0.08	-0.38	-0.24	-0.31	-0.07	-0.38	-0.12	-0.31	-0.31	-0.24	-0.31	-0.24	-0.38	-0.31	-0.24	0.43	1.17	0.23	Stable	15.00
ENSSSCG00000023305	0.05	0.03	0.06	0.05	0.05	0.06	0.26	0.19	0.33	0.06	0.02	0.07	0.09	0.05	0.11	0.05	0.09	2.34	1.22	Stable	6.00
ELMO2	-0.60	0.10	-0.51	-0.33	-0.63	-0.87	-0.45	-0.30	0.15	0.12	-0.72	-0.06	0.02	0.03	-0.44	-0.64	0.44	0.65	-0.62	Stable	17.00
CYP3A29	1.46	-0.39	0.59	-0.24	0.00	0.06	0.39	0.28	0.46	-0.32	1.03	0.50	0.23	0.72	0.62	0.02	0.16	3.20	1.68	Stable	3.00
ENSSSCG00000023325	-0.11	-0.38	-0.55	-0.45	0.44	0.21	-0.11	-0.50	-0.06	-0.21	-0.42	-0.38	-0.27	-0.11	-0.11	-0.52	0.79	1.19	0.25	Stable	13.00
FCN2	-1.51	-1.21	-0.92	-1.42	-1.40	-0.83	-0.82	-0.87	-0.73	-1.41	-1.23	-0.83	-1.06	-0.87	-1.98	-0.79	0.97	0.99	-0.01	Stable	1.00
REST	-0.28	-0.32	-0.58	-0.53	-0.17	-0.36	-0.33	-0.37	-0.37	-0.37	-0.38	-0.37	-0.37	-0.32	-0.37	-0.40	1.00	1.00	0.00	Stable	8.00
LIX1	0.06	0.12	0.12	0.27	0.31	0.12	0.18	0.12	0.06	0.12	0.31	0.33	0.50	0.12	0.18	0.12	0.02	0.46	-1.12	Down	2.00
PPM1L	-0.12	0.00	0.00	0.00	-0.03	-0.14	-0.06	-0.09	-0.10	-0.09	-0.01	-0.01	-0.04	0.00	-0.28	-0.14	0.59	1.36	0.45	Stable	13.00
KCNK2	0.06	-0.05	-0.10	0.09	0.07	0.05	-0.06	0.01	0.11	-0.17	0.15	-0.01	0.34	0.20	0.16	0.00	0.72	0.64	-0.65	Stable	9.00
SLC46A1	-0.27	-0.78	-0.73	-0.24	-0.60	-0.23	-0.61	-0.51	-0.31	-0.35	-0.19	-0.35	0.01	-0.88	-0.44	-0.70	0.05	1.71	0.77	Stable	12.00
PCDHGB5	-0.08	-0.22	-0.04	-0.01	-0.17	-0.05	-0.22	-0.22	0.10	0.06	-0.08	-0.05	-0.20	-0.08	0.15	-0.06	0.93	1.08	0.11	Stable	2.00
CCKAR	0.00	0.00	0.07	-0.31	0.00	-0.22	-0.13	0.00	0.00	0.00	-0.71	-0.28	-0.73	0.00	-0.30	-0.28	0.03	0.14	-2.84	Down	8.00
TGM2	0.10	0.52	0.06	0.40	0.47	0.30	0.59	0.03	0.08	0.36	0.18	0.26	0.64	0.19	0.19	0.47	0.10	0.57	-0.81	Stable	17.00
IKZF5	0.00	0.00	0.00	-0.13	-0.26	-0.13	-0.04	0.00	0.00	-0.13	0.00	0.00	-0.26	0.00	-0.26	-0.13	0.07	0.29	-1.81	Stable	14.00
SERINC2	-0.04	-0.01	0.02	0.43	0.14	0.08	-0.14	0.12	-0.24	0.04	0.14	-0.14	0.43	0.14	0.01	0.18	0.04	-0.11	-3.23	Down	6.00
AK3	0.05	-0.04	0.03	0.04	0.04	0.04	-0.04	0.05	-0.04	0.00	0.04	0.00	0.04	-0.04	0.05	0.04	0.14	0.05	-4.37	Stable	1.00
ENSSSCG00000023596	0.16	0.27	0.45	0.23	-0.15	0.47	-0.32	0.10	0.18	0.33	0.39	0.54	-0.32	0.11	-0.09	0.16	0.48	0.53	-0.91	Stable	14.00
LAP3	-0.02	-1.05	-1.04	-1.16	-0.01	-0.38	-0.81	-1.30	-1.50	-1.50	-1.03	-0.94	-0.97	-1.09	-0.37	-1.20	1.00	1.00	0.00	Stable	8.00
FBXO25	-0.13	-0.25	-0.19	-0.02	-0.29	0.03	-0.39	-0.17	0.25	0.11	-0.19	-0.37	-0.02	0.07	-0.25	-0.16	0.83	1.18	0.24	Stable	15.00
CHMP1A	0.26	-0.32	-0.32	1.25	0.18	0.24	0.26	0.26	0.26	0.26	0.10	0.71	0.16	-0.09	0.19	0.24	0.07	0.17	-2.59	Stable	6.00
MT1A	0.27	0.46	0.41	-0.10	0.00	0.06	0.54	0.85	0.64	0.49	0.06	0.11	-0.09	0.14	0.98	-0.05	0.00	8.69	3.12	Up	6.00
TTR	0.08	0.00	0.00	-0.48	0.35	0.00	0.00	-0.04	0.00	-0.26	-0.48	0.00	-0.10	0.00	0.06	0.17	0.33	-0.11	-3.15	Stable	6.00
REEP1	0.82	0.61	0.94	0.60	0.40	0.09	0.98	0.36	0.31	0.84	0.86	0.58	0.84	0.84	0.40	0.95	0.94	1.02	0.03	Stable	3.00
TRIM37	0.65	0.56	0.65	0.70	0.76	0.26	0.84	0.25	0.43	0.39	0.40	0.80	0.52	0.27	0.27	0.54	0.62	0.90	-0.15	Stable	12.00
COG3	-0.70	-0.63	-1.24	-0.08	-0.09	-0.27	-0.75	-0.78	-0.53	-0.79	-0.71	-0.29	-0.18	-0.21	-0.27	-0.21	0.06	1.94	0.96	Stable	11.00
MCOLN3	0.94	0.50	0.17	0.39	0.37	0.26	0.01	0.51	0.01	0.01	0.29	0.03	1.08	0.50	0.01	0.24	0.98	0.99	-0.01	Stable	4.00
ENSSSCG00000023749	1.17	0.96	1.13	1.89	1.29	1.29	0.45	0.96	1.28	0.96	1.31	0.45	1.72	0.53	1.10	1.23	0.12	0.75	-0.42	Stable	5.00
ENSSSCG00000023771	-0.45	-0.56	0.01	-0.17	-0.44	-0.45	-0.42	-0.33	-0.22	-0.57	-0.23	-0.35	-0.10	-0.14	-0.55	-0.55	0.80	0.93	-0.10	Stable	11.00
SNRNP70	-0.14	0.00	-0.07	-0.07	-0.25	0.00	-0.14	-0.07	0.00	-0.07	-0.07	-0.14	-0.24	-0.07	-0.14	-0.33	0.17	0.54	-0.88	Stable	6.00
LANCL2	-0.24	-0.48	-0.40	-0.26	-0.44	-0.04	-0.22	-0.42	-0.41	0.12	-0.47	-0.42	-0.28	-0.09	-0.68	-0.25	0.27	1.43	0.52	Stable	18.00
LRRN1	0.00	0.00	0.04	0.03	-0.01	-0.01	0.00	0.08	0.03	0.03	0.08	0.04	0.08	0.16	0.04	0.08	0.92	1.06	0.08	Stable	13.00
ACO1	0.11	0.07	-0.27	-0.13	0.04	-0.48	-0.32	-0.61	-0.21	-0.08	-0.51	-0.06	-0.27	-0.08	-0.17	0.03	1.00	1.00	0.00	Stable	10.00
FAIM	0.05	-0.01	-0.02	0.06	0.01	0.06	0.05	-0.02	0.06	0.06	-0.02	-0.06	0.06	-0.02	0.04	0.06	0.64	0.64	-0.64	Stable	13.00
MRPS27	0.28	0.44	0.21	0.20	-0.07	0.31	0.65	0.24	0.23	0.06	0.34	0.61	0.32	0.65	0.61	0.28	0.14	1.61	0.69	Stable	16.00
CCP110	-0.05	-0.01	0.00	-0.01	-0.01	-0.12	0.00	-0.05	-0.15	-0.01	-0.01	-0.03	-0.01	-0.09	-0.03	-0.01	0.36	1.84	0.88	Stable	3.00
AZIN1	0.25	0.24	0.29	0.41	0.34	0.16	0.39	0.28	0.33	0.45	0.44	0.54	0.44	0.46	0.29	0.44	0.09	0.78	-0.35	Stable	4.00
GRM7	0.06	-0.40	-0.41	0.51	-0.55	-0.56	0.17	-0.21	-0.13	-0.27	-0.43	0.76	0.27	-0.17	-0.03	-0.63	0.90	1.23	0.30	Stable	13.00
TXNRD3	0.52	0.87	0.68	0.23	0.84	0.63	0.12	0.77	-0.20	-0.06	-0.12	-0.15	-0.38	0.13	-0.77	0.63	0.81	1.31	0.39	Stable	13.00
CFAP126	-0.16	0.02	-0.19	-0.05	0.10	0.02	-0.12	0.10	0.08	0.10	0.10	-0.07	0.10	-0.15	-0.11	0.10	0.03	-1.29	0.36	Stable	4.00

H3-3A	0.00	0.04	0.04	0.09	0.00	0.24	0.05	0.04	0.09	0.04	0.04	0.00	0.24	0.04	0.00	0.09	0.16	0.41	-1.27	Stable	10.00
AGPS	0.34	0.39	0.28	0.50	0.44	0.18	0.39	0.39	0.90	0.44	0.58	0.44	0.50	0.28	0.38	0.50	0.74	0.94	-0.09	Stable	15.00
PLXNA1	-0.27	-0.53	-0.22	-0.05	-0.49	0.10	-0.30	-0.43	-0.24	-0.23	0.01	0.06	0.15	-0.10	0.18	-0.11	0.13	3.41	1.77	Stable	13.00
FADS1	-0.43	0.11	-0.33	0.64	0.83	0.45	-0.43	-0.43	-0.45	-0.43	0.13	-0.06	-0.40	-0.12	0.51	0.32	0.08	-1.04	0.06	Stable	2.00
ENSSSCG00000024021	0.10	0.27	0.06	-0.23	0.35	0.13	0.34	0.01	0.49	0.24	-0.36	0.44	-0.19	-0.07	0.65	0.44	0.39	2.22	1.15	Stable	10.00
CASTOR2	0.15	0.10	0.18	0.00	0.23	0.16	0.23	0.00	0.07	0.02	0.00	0.10	0.07	0.16	0.05	0.23	0.72	1.16	0.21	Stable	3.00
MMRN2	0.15	-0.31	0.11	0.12	-0.04	0.10	0.00	0.04	-0.26	0.03	0.09	0.02	-0.03	0.15	-0.42	-0.04	0.26	-2.07	1.05	Stable	14.00
SLC5A2	0.15	0.05	0.06	0.11	0.14	0.13	0.18	0.11	0.15	0.14	0.14	0.14	0.16	0.15	0.10	0.18	0.26	0.85	-0.24	Stable	3.00
MGLL	0.11	-0.11	-0.10	-0.02	-0.16	-0.14	0.02	-0.09	0.16	0.25	0.22	0.13	0.20	0.11	0.44	-0.15	0.78	1.66	0.73	Stable	13.00
AMPH	0.21	0.33	-0.18	-0.05	0.14	0.05	-0.09	0.33	0.28	-0.05	0.17	0.31	0.00	0.14	0.03	0.25	0.75	1.26	0.34	Stable	9.00
ELOVL5	-0.05	-0.29	-0.29	-0.32	-0.35	-0.32	-0.17	-0.04	-0.12	-0.32	-0.32	-0.19	-0.37	-0.13	-0.32	-0.37	0.01	0.55	-0.86	Stable	7.00
ENSSSCG00000024161	-0.05	-0.09	0.07	-0.09	0.26	0.12	-0.05	0.03	-0.21	-0.19	-0.02	0.16	-0.08	-0.23	-0.02	-0.13	0.31	-20.36	4.35	Stable	7.00
ACBD3	0.46	0.03	0.54	0.31	0.75	0.31	0.51	0.29	0.38	0.37	0.66	0.75	0.31	0.42	0.58	0.48	0.34	0.81	-0.30	Stable	10.00
ARHGEF26	0.19	0.25	0.07	0.15	0.08	0.20	0.09	0.02	-0.02	-0.20	0.11	0.08	-0.06	-0.03	0.18	0.20	0.69	1.35	0.44	Stable	13.00
CCDC91	0.03	0.05	-0.10	-0.68	-0.68	-0.03	0.19	-0.21	-0.08	-0.68	-0.25	-0.02	-0.68	0.19	-0.21	-0.16	0.01	0.04	-4.56	Down	5.00
GABRR2	0.55	1.56	0.53	0.30	-0.02	0.43	0.20	1.15	1.22	0.08	0.38	0.41	0.17	0.41	0.76	-0.05	0.01	3.74	1.90	Up	1.00
ZMAT5	-0.01	0.87	0.18	-0.78	-0.92	-0.34	-0.01	-0.46	-0.59	0.25	-0.59	-0.84	-0.67	-0.80	0.56	-0.54	0.05	0.06	-4.10	Stable	14.00
ENSSSCG00000024281	0.38	-0.03	0.08	0.07	0.04	0.08	0.35	-0.30	-0.19	-0.07	0.15	0.38	0.19	0.04	0.35	0.09	0.74	0.71	-0.50	Stable	6.00
NKIRAS1	0.07	-0.10	0.09	0.32	0.23	0.09	0.05	0.07	0.07	0.23	0.26	0.26	0.21	0.25	0.27	0.20	0.02	0.43	-1.23	Down	13.00
TMEM254	0.00	0.56	0.41	0.15	0.53	1.11	0.23	0.09	0.00	0.26	0.28	0.25	-0.03	-0.03	0.69	0.53	0.38	0.63	-0.67	Stable	14.00
MT3	-0.08	0.13	-0.21	0.09	0.07	0.07	0.22	0.47	0.29	0.06	0.55	-0.26	0.25	-0.01	0.42	0.07	0.74	1.36	0.44	Stable	6.00
BNIP3	0.49	0.55	0.36	0.84	-0.69	-0.42	0.63	0.02	0.49	-0.33	0.52	-0.64	-0.52	0.01	0.46	-1.14	0.02	-1.27	0.35	Stable	14.00
ENSSSCG00000024411	-0.37	-0.58	-0.21	-0.18	-0.29	-0.54	-0.29	-0.21	-0.54	-0.37	-0.33	-0.20	-0.31	-0.20	-0.20	-0.22	0.78	1.06	0.09	Stable	12.00
RHOU	-0.34	0.12	-0.04	0.15	0.12	0.68	-0.44	-0.21	-0.36	0.15	0.21	0.23	0.39	-0.35	-0.17	-0.02	0.00	-0.94	-0.09	Stable	14.00
FAM91A1	-0.39	-0.25	-0.36	-0.50	-0.56	-0.72	-0.72	-0.09	-0.36	-0.34	-0.72	-0.72	-0.72	-0.37	-0.42	-0.08	0.11	0.68	-0.56	Stable	4.00
CES3	-0.17	-0.24	-0.49	-0.14	-0.10	-0.12	-0.33	-0.34	-0.49	-0.17	-0.08	-0.34	-0.25	-0.34	-0.33	-0.12	0.00	2.06	1.04	Up	6.00
NOCT	-0.76	-0.35	-0.23	-0.41	-0.47	-0.55	-0.76	-0.35	-0.64	0.01	-0.23	-0.49	-0.23	-0.48	-0.35	-0.18	0.10	1.55	0.63	Stable	8.00
TNFAIP1	0.00	0.00	0.00	0.00	0.00	-0.01	0.00	0.00	0.00	0.00	0.00	0.00	0.00	0.00	0.00	-0.01	0.13	-0.15	-2.77	Stable	12.00
GPRL15	0.19	0.03	0.00	-0.08	-0.17	-0.33	-0.08	-0.02	0.03	-0.09	-0.01	0.11	-0.37	0.15	0.48	-0.17	0.01	-0.69	-0.53	Stable	14.00
PRPF4	-0.33	-0.27	-0.48	-0.03	0.06	-0.06	-0.19	0.07	-0.11	-0.03	0.05	-0.22	-0.32	0.17	-0.20	-0.19	0.42	1.80	0.85	Stable	1.00
TRAPPC12	-0.47	-0.18	-0.11	-0.03	0.02	0.01	-0.55	-0.11	0.00	-0.10	-0.32	0.00	-1.17	-0.40	-0.37	0.11	0.61	1.47	0.55	Stable	3.00
ENSSSCG00000024669	0.05	-0.24	-0.37	-0.21	-0.28	-0.21	-0.12	-0.15	-0.22	-0.21	-0.37	-0.12	-0.09	0.25	0.04	-0.26	0.14	0.44	-1.19	Stable	6.00
ENSSSCG00000024696	-0.72	0.04	-0.34	-0.72	-0.44	-0.73	-0.44	0.03	-0.07	-0.29	-0.40	-0.44	-0.39	-0.73	-0.31	-0.73	0.14	0.62	-0.70	Stable	15.00
ACP4	0.68	0.24	0.70	-0.11	0.02	0.41	0.98	0.31	0.24	-0.01	0.55	0.98	0.54	0.13	0.98	0.59	0.38	1.44	0.52	Stable	6.00
THUMPD1	0.05	-0.09	-0.03	0.01	0.01	0.01	0.01	0.14	-0.09	0.03	0.01	0.19	0.01	0.17	0.18	0.01	0.89	1.17	0.22	Stable	3.00
ALDH4A1	-0.39	-0.16	-0.18	-0.23	-0.02	-0.30	-0.50	-0.20	-0.26	-0.26	-0.05	-0.50	-0.17	-0.14	-0.04	-0.16	0.76	1.11	0.15	Stable	6.00
LIG3	-0.46	-0.27	-0.14	-0.55	-0.40	-0.37	-0.20	-0.20	-0.46	-0.40	-0.44	-0.35	0.10	0.02	-0.72	-0.14	0.88	0.95	-0.08	Stable	12.00
EID2	0.00	0.14	0.00	0.00	0.00	0.00	0.00	0.00	0.17	0.00	0.00	0.00	0.00	0.00	0.00	0.00	0.17	Inf	Inf	Stable	6.00
ZFAND1	-0.58	-1.44	-0.91	-0.30	-0.92	-1.18	-0.22	-1.31	-0.75	-0.87	-0.43	-0.36	-0.06	-1.22	-0.14	-0.06	0.21	1.57	0.65	Stable	4.00
ENSSSCG00000024791	-0.16	-0.30	0.69	0.08	0.03	0.13	-0.45	0.71	0.09	-0.25	0.83	0.43	0.15	0.32	-0.37	0.58	0.39	0.26	-1.93	Stable	2.00
ENSSSCG00000024800	-0.02	-0.12	-0.09	-0.19	-0.43	-0.03	-0.03	-0.27	0.00	-0.13	-0.19	0.00	-0.31	-0.06	-0.27	-0.22	0.22	0.57	-0.81	Stable	6.00
MACC1	-0.72	-1.22	-0.97	-1.01	-1.01	-1.67	-1.69	-2.31	0.12	-1.64	-0.40	-2.31	-1.75	-2.39	-1.12	-0.37	0.97	1.01	0.02	Stable	9.00
ABTB1	-0.10	-0.10	-0.21	-0.26	-0.21	-0.23	-0.29	-0.13	-0.10	-0.21	-0.27	-0.68	-0.48	-0.26	-0.78	-0.20	0.48	0.77	-0.38	Stable	13.00
SYT12	-0.31	-0.32	-0.37	-0.15	-0.10	-0.33	-0.39	0.05	-0.04	-0.39	-0.18	-0.32	-0.19	-0.32	-0.24	-0.29	1.00	1.00	0.00	Stable	2.00
ATXN10	0.17	0.28	0.17	0.28	0.04	0.18	0.19	0.44	0.30	0.17	0.14	0.28	0.15	0.28	0.28	0.02	0.04	1.70	0.76	Stable	5.00

TMEM181	0.25	-0.63	-0.66	-0.06	0.21	0.22	0.08	0.35	0.39	0.03	-0.02	0.22	0.37	-0.32	0.01	0.11	0.24	-0.50	-1.01	Stable	1.00
GGA1	0.09	-0.05	-0.19	-0.19	-0.10	-0.14	-0.12	0.10	0.05	-0.42	-0.14	-0.21	-0.19	-0.11	0.17	-0.25	0.00	0.04	-4.64	Down	5.00
ENSSSCG00000024914	0.14	0.02	0.57	-0.06	0.08	0.00	0.14	0.27	0.35	0.23	0.06	0.35	-0.06	-0.02	0.02	-0.06	0.21	2.72	1.44	Stable	7.00
SLC36A4	-0.33	0.61	-0.04	0.08	-0.02	-0.01	-0.04	-0.15	0.68	-0.15	-0.15	0.49	1.05	0.59	0.64	0.52	0.93	1.08	0.12	Stable	9.00
MPI	0.05	-0.08	-0.01	-0.07	-0.08	0.11	-0.01	0.02	-0.05	-0.01	0.07	-0.03	-0.08	0.05	-0.08	-0.04	0.91	0.79	-0.35	Stable	7.00
B4GALT6	0.18	0.00	-0.13	0.36	0.62	-0.13	-0.03	0.08	0.11	0.10	0.36	-0.27	0.44	0.05	0.16	0.22	0.18	0.24	-2.06	Stable	6.00
NFU1	-0.14	0.53	-0.27	-0.35	0.77	-0.60	-0.18	0.40	0.40	0.52	0.54	0.10	-0.38	-0.28	-0.04	-0.64	0.81	-11.05	3.47	Stable	3.00
PGGHG	0.12	0.35	0.31	0.25	0.48	0.40	0.19	0.20	0.15	0.14	0.51	0.22	0.00	0.43	0.00	0.34	0.39	0.76	-0.40	Stable	2.00
CYBRD1	-0.92	-0.86	-0.94	-0.85	-0.58	-0.91	-0.94	-1.06	-1.09	-1.00	-1.15	-0.29	-1.15	-0.94	-0.82	-0.59	0.27	1.16	0.21	Stable	15.00
THNSL2	-0.83	-0.49	-0.27	-0.87	-0.89	-0.60	-0.67	-0.97	-0.15	-0.45	-1.41	-0.22	-0.83	-0.23	-0.25	-0.81	0.12	0.63	-0.66	Stable	3.00
TONSL	0.44	0.24	0.97	0.85	0.31	0.85	0.34	0.49	0.25	0.64	-0.57	0.30	0.67	-0.10	0.86	0.11	0.85	1.10	0.14	Stable	4.00
ATN1	-0.14	0.23	0.14	-0.03	-0.03	-0.07	-0.12	-0.04	0.21	-0.03	-0.02	0.20	-0.03	0.34	0.95	0.00	0.17	542.38	9.08	Stable	5.00
BORA	0.00	0.25	0.10	0.14	0.02	0.10	0.00	0.30	0.20	0.02	0.02	0.24	0.47	0.27	0.13	0.00	0.68	1.24	0.31	Stable	11.00
CEP89	-0.42	-0.13	-0.30	-0.34	-0.28	-0.20	-0.63	-0.32	-0.32	-0.32	-0.39	-0.63	-0.38	0.14	-0.63	-0.65	0.50	0.81	-0.30	Stable	6.00
NECAP1	0.37	1.19	0.91	0.31	0.31	0.25	0.77	0.34	0.41	0.31	-0.09	0.52	0.31	1.16	1.00	0.60	0.01	2.45	1.29	Up	5.00
ASCC2	1.70	0.84	1.35	2.09	1.67	1.79	1.91	1.71	2.87	2.26	1.56	1.38	2.31	1.73	1.10	1.86	0.41	0.89	-0.18	Stable	14.00
RNF39	0.36	0.84	0.72	0.97	0.65	1.02	0.28	0.91	0.55	0.83	1.22	0.40	1.07	0.16	0.65	1.11	0.02	0.61	-0.70	Stable	7.00
DDIT4L	0.00	0.00	0.00	0.12	0.00	0.24	0.12	0.00	0.00	0.00	0.00	0.00	0.12	0.00	0.00	0.00	0.23	0.25	-2.00	Stable	8.00
VWA3A	-0.09	-0.10	0.31	-0.06	-0.12	-0.08	-0.01	-0.05	0.34	-0.12	-0.19	0.04	-0.01	0.04	-0.07	-0.05	0.11	-0.61	-0.70	Stable	3.00
ENSSSCG00000025271	0.37	0.37	0.78	0.06	0.17	0.23	0.45	0.81	0.66	0.49	0.37	0.21	0.27	0.42	0.48	0.15	0.00	2.23	1.16	Up	3.00
ARL6IP1	0.09	0.09	0.10	0.08	0.04	-0.11	0.09	0.09	-0.07	0.04	0.08	0.10	0.09	0.10	0.09	0.10	0.56	1.37	0.45	Stable	3.00
MCTP1	-0.17	-0.25	-0.04	-0.26	-0.18	-0.25	-0.21	-0.25	-0.17	-0.21	-0.25	-0.34	-0.43	-0.25	-0.25	-0.21	0.10	0.74	-0.43	Stable	2.00
GIN1	-0.05	0.07	-0.04	-0.04	0.26	0.07	0.30	-0.01	-0.13	0.07	0.17	-0.02	0.23	0.07	-0.12	0.07	0.16	0.09	-3.47	Stable	2.00
AIRE	-0.03	0.69	0.74	-0.23	0.28	0.69	-0.14	0.93	-0.22	0.62	-0.19	0.04	-0.32	-0.22	0.81	0.32	0.47	2.14	1.10	Stable	13.00
TMEM243	0.59	0.59	0.59	0.00	0.00	0.00	0.21	0.37	0.27	0.00	0.28	0.31	0.00	0.00	0.18	0.24	0.02	3.35	1.74	Up	9.00
GUCY2D	0.33	0.15	0.32	0.00	-0.08	-0.25	0.28	-0.68	-0.24	-0.42	-0.25	-0.19	-0.47	0.07	-0.22	-0.10	0.14	0.00	-7.74	Stable	12.00
ZNF146	0.03	0.30	0.19	-0.51	0.17	-0.16	0.29	-0.31	0.13	-0.18	-0.09	0.29	0.08	0.18	0.29	0.22	0.20	-5.72	2.52	Stable	6.00
SNX17	-0.14	0.11	-0.23	-0.03	-0.07	0.00	0.11	0.14	0.18	0.27	0.18	0.14	0.02	0.09	0.27	0.14	0.84	0.81	-0.30	Stable	3.00
ENSSSCG00000025403	0.03	0.02	0.03	0.03	0.03	0.03	0.03	0.00	0.00	0.03	0.03	-0.01	0.03	-0.03	0.03	0.00	0.46	0.64	-0.64	Stable	17.00
ULK4	0.00	-0.11	-0.11	-0.05	-0.20	-0.42	0.00	-0.15	-0.16	-0.18	-0.22	0.04	-0.04	-0.51	0.00	-0.35	0.56	0.73	-0.46	Stable	13.00
PRSS16	-0.07	0.27	-0.17	0.27	0.02	0.21	-0.17	0.53	0.29	0.39	0.38	0.27	0.32	-0.07	0.19	0.24	0.14	0.38	-1.39	Stable	7.00
ENSSSCG00000025417	0.14	-0.14	0.00	-0.13	-0.02	-0.13	0.08	0.19	0.11	0.25	-0.02	0.14	-0.17	0.08	0.26	-0.13	0.12	-3.55	1.83	Stable	6.00
PROS1	0.06	0.23	0.19	-0.08	0.20	0.06	0.06	0.46	0.28	0.06	0.13	-0.21	-0.08	0.12	0.46	0.06	0.01	14.04	3.81	Up	13.00
ENSSSCG00000025485	-0.42	0.21	-0.20	-0.16	-0.10	0.39	-0.45	0.28	-0.16	-0.09	-0.33	-0.39	-0.25	0.04	-0.06	-0.02	0.87	0.81	-0.30	Stable	4.00
NUDT13	-0.05	0.15	0.14	0.00	-0.02	0.00	-0.13	-0.13	-0.13	-0.26	0.00	0.08	-0.13	0.08	0.00	-0.02	0.54	0.21	-2.24	Stable	14.00
SSPN	0.00	0.00	0.00	0.00	0.00	0.00	0.00	0.00	0.00	0.00	0.00	0.00	0.00	0.00	0.16	0.00	0.35	Inf	Inf	Stable	5.00
CACNG7	0.31	0.21	0.31	0.04	0.65	0.12	0.33	0.13	0.13	0.21	0.12	0.33	0.69	0.28	0.33	0.58	0.38	0.74	-0.43	Stable	6.00
PGLYRP2	-0.13	-0.30	-0.09	0.28	-0.24	-0.04	0.40	0.96	0.38	0.69	-0.15	0.39	0.29	0.49	0.20	0.19	0.73	1.36	0.44	Stable	2.00
GAS2L1	0.05	-0.91	-0.20	-0.29	0.01	-0.22	-0.18	0.07	-0.26	-0.33	-0.29	-0.25	-0.39	-0.13	-0.90	-0.22	0.68	1.24	0.31	Stable	14.00
ENSSSCG00000025565	-0.21	-0.28	-0.16	-0.13	-0.18	-0.10	-0.02	-0.21	-0.22	-0.20	-0.14	-0.08	-0.01	-0.28	-0.28	0.07	0.03	2.15	1.11	Up	3.00
TEAD2	0.06	-0.37	0.06	0.00	-0.14	0.00	0.12	0.06	-0.15	0.00	0.04	0.12	-0.06	0.00	0.12	-0.05	1.00	1.01	0.01	Stable	6.00
SMIM26	0.20	0.20	0.20	0.20	0.20	0.20	0.20	0.10	0.00	0.00	0.20	0.20	0.20	0.10	0.20	0.20	0.51	0.86	-0.22	Stable	17.00
ENSSSCG00000025631	-1.10	-1.22	-0.72	-0.67	-0.60	-0.82	-0.54	-1.02	-1.00	-1.29	-1.26	-0.87	-1.08	-0.86	-1.12	-0.86	0.89	1.02	0.02	Stable	2.00
ENSSSCG00000025673	0.13	-0.14	0.10	0.02	-0.08	0.06	0.28	-0.03	0.02	-0.15	-0.03	-0.13	0.04	0.14	-0.04	-0.08	0.08	-1.31	0.38	Stable	17.00
SNRNP27	-0.16	0.14	-0.31	0.03	0.14	0.41	-0.16	0.09	0.02	-0.23	-0.09	0.09	-0.03	-0.16	0.43	0.16	0.51	-0.23	-2.10	Stable	3.00

ACTR6	-0.10	0.01	-0.12	-0.44	-0.34	-0.06	-0.19	0.30	0.05	0.07	-0.54	-0.26	-0.35	0.04	0.30	-0.01	0.02	-0.14	-2.79	Down	5.00
DVL2	1.37	0.44	0.25	0.93	0.44	0.50	0.98	0.04	0.11	-0.09	0.33	0.77	0.04	0.55	1.40	0.30	0.31	1.60	0.68	Stable	12.00
DENND2D	-0.11	-0.51	-0.11	-0.22	-0.24	-0.23	-0.12	-0.05	-0.15	-0.09	-0.12	-0.11	-0.16	-0.11	-0.11	-0.24	0.75	0.90	-0.16	Stable	4.00
OLAH	0.54	0.17	0.34	0.43	0.27	0.26	0.55	0.43	0.34	0.41	0.43	0.45	0.17	0.37	0.09	0.28	0.81	1.05	0.07	Stable	10.00
GPR45	0.00	0.00	0.00	0.00	0.01	0.00	0.00	0.00	0.00	0.00	0.00	0.00	0.01	0.00	0.00	0.00	0.17	0.00	#NAME?	Stable	3.00
CDH4	0.01	0.04	0.08	0.18	0.04	0.06	0.01	0.04	0.50	0.11	0.08	0.05	0.08	0.01	0.01	0.04	0.95	1.05	0.07	Stable	17.00
ENPP4	-0.13	-0.01	-0.13	0.05	0.45	0.23	0.07	0.08	-0.21	-0.49	0.47	-0.07	0.33	0.26	0.13	0.51	0.21	0.03	-4.95	Stable	7.00
FBRS	0.06	0.18	-0.09	0.23	0.20	0.06	0.19	0.08	0.29	0.11	0.11	0.11	0.20	0.23	0.03	0.19	0.56	0.81	-0.31	Stable	3.00
NLE1	0.01	0.27	0.00	0.01	0.00	0.00	0.14	0.27	0.14	0.01	0.00	0.00	0.27	0.27	0.14	0.13	0.07	2.98	1.57	Stable	12.00
C2CD2	0.03	-0.38	-0.20	0.09	-0.31	-0.54	-0.34	-0.16	-0.12	-0.23	-0.16	-0.33	-0.34	-0.34	-0.17	-0.25	0.56	0.82	-0.29	Stable	13.00
SFRP1	0.39	0.27	0.40	0.08	0.49	0.40	0.14	0.57	0.69	0.38	0.59	0.17	-0.29	-0.10	0.74	0.24	0.37	1.51	0.60	Stable	17.00
HEPACAM2	-0.23	-0.22	-0.19	-0.33	-0.10	-0.35	-0.22	-0.63	-0.28	-0.22	-0.16	-0.23	-0.35	-0.17	-0.13	-0.13	0.70	1.11	0.15	Stable	9.00
PBLD	-0.59	-0.55	-0.72	-0.25	0.23	-0.73	0.25	-0.49	-0.67	-0.39	-0.21	0.20	-0.43	-0.42	-0.09	0.33	0.17	2.63	1.40	Stable	14.00
MCCC1	-0.15	0.00	0.00	-0.26	-0.24	-0.29	-0.15	0.00	-0.32	-0.32	-0.28	-0.32	-0.07	-0.26	-0.32	-0.57	0.06	0.52	-0.96	Stable	13.00
DNAJC1	0.04	-0.06	-0.03	0.24	-0.34	0.22	-0.04	0.19	0.12	0.34	0.29	0.04	0.29	0.02	0.45	-0.40	0.99	1.01	0.02	Stable	10.00
DPP9	-0.12	-0.30	-0.34	-0.33	-0.22	0.09	-0.06	0.01	-0.05	-0.15	-0.05	-0.31	-0.28	-0.14	-0.10	-0.10	0.65	0.82	-0.29	Stable	2.00
TKTL2	-0.54	-0.68	-0.99	-0.82	-0.49	-0.81	-0.54	-0.81	-0.56	-0.73	-0.81	-0.81	-0.81	-0.52	-0.81	-0.81	0.30	0.90	-0.16	Stable	8.00
TXNDC15	0.00	0.23	0.24	0.09	0.15	0.13	0.16	0.18	0.15	0.02	0.31	0.15	0.06	0.20	0.06	0.15	0.66	1.15	0.20	Stable	2.00
EPHA3	-0.86	-0.12	-0.05	-0.52	-0.50	-0.86	-0.86	-0.11	-0.40	-0.86	-0.08	0.24	-0.53	-0.08	-0.11	-0.86	0.37	0.65	-0.62	Stable	13.00
RAD18	0.02	0.00	0.01	0.00	0.00	0.00	0.01	0.01	0.00	0.00	0.01	0.01	0.00	0.01	0.01	0.02	0.27	1.47	0.55	Stable	13.00
ENSSSCG00000026210	-0.14	-0.63	-0.25	-1.17	-0.82	-0.83	-0.56	-0.72	-0.25	-0.82	-0.82	-0.32	-0.82	-0.74	-0.70	-0.82	0.02	0.62	-0.68	Stable	4.00
SRP19	-0.14	-0.14	-0.10	-0.43	-0.14	-0.18	-0.15	-0.14	-0.14	-0.18	-0.36	-0.14	-0.58	-0.14	-0.14	-0.14	0.06	0.51	-0.96	Stable	2.00
FAM184B	0.00	-0.86	-0.90	-0.71	0.00	-0.51	-0.35	-0.92	-1.06	-1.06	-0.96	-0.93	-0.93	-0.90	-0.40	-0.87	0.69	0.90	-0.15	Stable	8.00
ENSSSCG00000026233	-0.29	-0.58	-0.29	-0.50	-0.25	-0.58	-0.10	-0.34	-0.10	-0.42	-0.67	-0.75	-0.73	-0.58	-0.32	-0.36	0.04	0.61	-0.71	Stable	2.00
B3GAT2	1.06	0.95	0.07	0.32	0.31	0.13	0.35	0.19	1.19	0.48	0.09	0.25	0.05	0.79	0.34	0.40	0.05	2.43	1.28	Stable	1.00
SCAP	0.00	0.04	-0.33	-0.52	0.07	0.07	0.00	0.04	0.00	-0.30	-0.38	-0.28	-0.61	0.00	0.00	-0.71	0.02	0.09	-3.45	Down	13.00
KLB	0.89	0.36	0.34	0.34	0.11	0.53	0.74	0.48	0.59	0.61	0.36	0.40	0.89	0.89	0.43	0.30	0.22	1.33	0.42	Stable	8.00
ENSSSCG00000026339	0.02	0.17	0.00	-0.24	-0.39	-0.24	-0.09	-0.05	0.14	-0.16	-0.12	-0.16	-0.07	0.02	0.08	-0.39	0.00	-0.17	-2.58	Down	14.00
ALDH3B2	0.03	-0.01	0.04	0.10	0.11	0.06	0.06	0.33	-0.05	-0.03	0.06	0.04	-0.14	0.01	-0.03	0.02	0.70	1.69	0.76	Stable	2.00
ENSSSCG00000026354	-0.22	-0.24	-0.46	-0.52	-0.24	-0.73	-0.19	-0.24	-0.09	-0.34	-0.62	-0.15	-0.50	-0.06	-0.14	-0.25	0.03	0.49	-1.04	Down	10.00
BSDC1	-0.27	-0.17	-0.07	-0.53	-0.17	-0.24	-0.21	-0.36	-0.18	-0.27	-0.14	-0.21	-0.53	-0.24	-0.10	-0.27	0.17	0.69	-0.54	Stable	6.00
TARS2	-0.55	-0.87	-0.99	-0.67	-0.73	-0.60	-0.58	-0.59	-0.27	-0.87	-0.87	-0.54	-0.87	-0.58	-0.52	-0.87	0.17	0.82	-0.28	Stable	4.00
ACSM5	0.25	0.22	0.33	0.15	0.26	0.62	0.04	0.20	0.16	-0.05	0.33	0.02	-0.08	0.00	0.24	-0.02	0.79	1.16	0.22	Stable	3.00
ABCA8	-0.26	0.26	-0.11	-0.25	-0.31	-0.01	-0.42	0.14	0.48	0.39	-0.02	0.05	-0.02	-0.08	0.18	-0.19	0.60	-0.54	-0.90	Stable	12.00
R3HDM2	0.02	0.04	0.01	-0.03	0.02	0.07	0.02	0.10	0.11	-0.03	-0.03	-0.03	-0.03	-0.02	-0.03	-0.03	0.07	-1.88	0.91	Stable	5.00
SETD6	-0.24	0.23	0.34	-0.04	-0.05	-0.13	0.23	-0.07	0.22	-0.24	-0.27	-0.24	0.04	0.21	-0.24	0.07	0.07	-0.79	-0.34	Stable	6.00
RALGAPA2	0.38	0.43	0.39	0.23	0.33	0.22	0.22	0.18	0.34	0.45	0.35	0.31	0.42	0.11	0.35	0.23	0.76	0.95	-0.07	Stable	17.00
CALML4	0.12	0.00	0.17	-0.30	-0.14	-0.28	0.12	0.06	0.07	-0.32	-0.26	-0.03	-0.23	0.06	0.12	-0.22	0.00	-0.41	-1.30	Down	1.00
HCN1	0.71	0.10	0.25	0.05	0.05	0.20	0.43	0.53	0.43	0.25	0.25	0.15	0.10	0.20	0.00	0.05	0.06	2.43	1.28	Stable	16.00
DCUN1D2	0.19	0.24	0.00	0.55	0.25	0.16	0.34	0.15	0.08	0.35	0.55	0.26	0.40	0.11	0.25	0.16	0.03	0.51	-0.99	Stable	11.00
ZNF621	0.55	-0.17	0.01	0.30	0.61	0.38	0.55	-0.02	0.54	0.67	0.38	0.38	0.06	0.38	0.55	0.78	0.31	0.68	-0.57	Stable	13.00
ENSSSCG00000026564	0.19	0.33	0.55	0.09	0.07	0.37	0.42	0.38	0.91	1.16	0.20	0.04	0.21	0.53	0.41	0.22	0.28	1.57	0.65	Stable	11.00
HGH1	0.55	0.61	0.29	0.54	0.42	0.12	0.40	0.47	0.38	0.61	0.95	0.61	0.58	0.61	0.47	0.58	0.41	0.86	-0.22	Stable	4.00
BPI	0.24	0.29	0.50	0.87	0.51	0.44	0.45	0.30	0.48	0.41	0.36	0.62	0.51	0.50	0.24	0.51	0.05	0.71	-0.50	Stable	17.00
PAIP2	-0.16	-0.08	-0.01	-0.16	0.66	0.46	-0.08	0.07	0.01	0.78	-0.01	0.24	0.27	-0.05	-0.09	0.28	0.01	-0.16	-2.69	Down	2.00

TIMM50	0.86	0.02	-0.28	1.42	0.11	0.96	0.23	-0.03	-0.19	0.68	0.99	0.08	0.88	0.42	0.13	0.60	0.02	0.20	-2.30	Down	6.00
RER1	-1.15	-0.59	-0.71	-0.78	-0.76	-0.73	-1.33	-0.57	-0.82	-0.74	0.12	-1.33	-1.30	-1.11	-1.33	-1.24	0.61	1.12	0.17	Stable	6.00
DTD2	-0.04	-0.61	-0.48	-0.77	-0.97	-0.69	-0.33	-0.26	0.16	-0.24	-0.78	-0.49	-0.97	-0.12	-0.58	-0.87	0.00	0.39	-1.35	Down	7.00
KCND3	0.05	-0.19	-0.05	0.00	0.30	0.66	0.06	0.41	0.22	0.30	0.07	0.05	0.18	0.43	0.00	0.37	0.28	0.49	-1.04	Stable	4.00
PDZD9	-0.94	-0.93	-0.95	-0.72	-1.29	-1.18	-0.98	-0.82	-0.36	-1.00	-1.14	-0.55	-1.02	-0.82	-0.93	-1.01	0.21	0.85	-0.23	Stable	3.00
ZNF180	0.15	0.22	0.30	0.06	0.24	0.13	0.44	0.30	0.30	0.20	-0.01	0.36	0.03	0.26	0.44	0.32	0.04	1.83	0.87	Stable	6.00
PHF14	0.09	0.09	0.09	0.04	0.04	0.09	0.09	0.09	0.09	0.00	0.09	0.09	0.04	0.09	0.09	0.04	0.02	1.60	0.68	Stable	9.00
PLBD1	0.32	-0.01	0.06	0.30	0.52	0.30	0.25	0.13	0.00	0.24	0.28	0.19	0.20	0.13	0.13	0.12	0.03	0.48	-1.06	Down	5.00
CCT8	1.09	1.48	1.41	0.34	1.39	1.14	0.47	1.65	1.11	1.08	1.30	-0.74	0.33	0.36	0.36	1.54	0.57	1.24	0.31	Stable	13.00
ANKLE2	0.00	-0.10	-0.24	-0.14	-0.06	-0.14	-0.28	-0.28	-0.03	-0.27	-0.02	-0.07	-0.10	-0.05	-0.15	-0.11	0.58	1.24	0.31	Stable	14.00
RHCE	-0.19	-0.27	-0.34	-0.39	-0.40	-0.39	-0.22	-0.22	-0.32	-0.40	-0.39	-0.22	-0.40	-0.29	-0.20	-0.31	0.00	0.71	-0.50	Stable	6.00
B4GALT2	0.11	-0.11	0.19	0.00	0.24	-0.28	0.19	-0.05	-0.20	0.30	-0.05	0.19	0.12	-0.12	0.23	0.34	0.43	0.28	-1.85	Stable	6.00
EMG1	0.58	1.05	1.04	0.06	0.06	0.55	0.49	0.18	0.14	0.06	0.09	0.55	0.06	0.41	0.49	0.59	0.07	2.17	1.12	Stable	5.00
ENSSSCG00000026816	-0.22	-1.05	-0.57	-0.15	-0.47	-0.17	-0.30	-1.05	-1.09	-0.60	-0.38	-0.29	-0.57	-0.60	-0.30	-0.58	0.12	1.61	0.69	Stable	6.00
NID1	0.15	0.22	0.11	0.11	0.04	0.15	0.22	0.15	0.00	0.22	0.00	0.15	0.11	0.04	0.22	0.04	0.37	1.35	0.43	Stable	14.00
RMC1	0.03	0.20	0.05	0.06	-0.03	0.00	-0.01	0.00	0.27	-0.04	0.03	-0.01	0.06	0.07	0.00	-0.04	0.09	41.69	5.38	Stable	6.00
DCHS2	0.09	0.23	0.18	0.92	0.65	0.92	0.50	0.34	0.19	0.55	0.49	0.46	0.34	0.48	0.46	0.77	0.00	0.49	-1.04	Down	8.00
PLAAT3	0.08	0.11	0.08	0.14	0.24	0.28	0.08	0.08	0.10	0.08	0.06	0.28	0.11	0.08	0.14	0.29	0.03	0.50	-1.00	Stable	2.00
GMPR2	-0.14	-0.59	-0.45	-0.36	-0.67	-0.60	-0.19	-0.08	0.06	-0.33	-0.42	-0.18	-0.67	-0.13	-0.68	-0.77	0.08	0.54	-0.88	Stable	7.00
TPGS2	0.82	0.81	0.35	0.53	0.36	0.13	0.40	0.71	0.42	0.16	0.53	0.03	0.37	0.81	0.63	0.19	0.00	2.15	1.10	Up	6.00
LURAP1	0.05	0.22	0.09	0.12	0.19	0.26	0.11	0.24	0.22	0.04	0.25	0.15	0.16	0.22	0.06	-0.03	0.83	1.07	0.10	Stable	6.00
CYP4F8	0.03	0.03	0.03	0.04	0.03	0.03	0.04	0.06	0.04	0.01	0.03	-0.79	0.04	0.04	0.04	0.04	0.32	-0.51	-0.96	Stable	2.00
PEX11A	-0.27	0.00	-0.18	-0.21	-0.04	-0.06	-0.18	-0.31	-0.18	-0.18	-0.06	-0.48	-0.04	-0.31	0.00	-0.21	0.82	1.10	0.14	Stable	7.00
OAZ3	0.27	0.00	0.01	1.29	0.97	0.57	1.34	1.25	0.28	1.13	1.13	0.36	1.13	1.09	0.09	1.13	0.10	0.56	-0.83	Stable	4.00
FOLH1B	-0.27	0.27	-0.03	-0.11	-0.25	0.11	-0.14	0.23	-0.10	0.16	-0.11	0.13	-0.26	0.12	0.24	-0.20	0.27	-0.60	-0.74	Stable	9.00
RPP38	-0.48	-0.33	-0.55	-0.55	-0.26	-0.45	-0.08	-0.55	-0.55	-0.08	-0.55	-0.26	-0.09	-0.29	-0.04	-0.29	0.68	1.13	0.18	Stable	10.00
MAP2K2	-0.25	-0.14	-0.06	-0.27	-0.07	-0.45	-0.16	-0.38	-0.38	-0.35	-0.20	-0.52	-0.25	-0.19	-0.32	-0.20	0.42	0.81	-0.30	Stable	2.00
LMNTD1	0.19	0.34	0.34	0.09	0.51	0.60	0.19	0.25	0.21	0.41	-0.12	0.72	0.09	0.44	0.19	0.57	0.45	0.75	-0.41	Stable	5.00
PPP1R12C	0.52	0.54	0.43	0.30	0.49	0.20	0.47	0.87	0.57	0.66	0.22	0.47	0.63	0.68	0.47	0.54	0.13	1.30	0.38	Stable	6.00
TUBA8	-0.16	-0.16	-0.16	0.14	0.14	0.09	-0.16	0.48	0.14	0.31	-0.05	0.16	0.19	-0.16	-0.16	-0.10	0.14	-0.35	-1.51	Stable	5.00
EPN3	-0.38	-0.31	-0.23	-0.37	-0.17	-0.21	-0.42	-0.33	-0.11	-0.06	-0.18	-0.42	-0.33	-0.20	-0.27	0.00	0.34	1.29	0.37	Stable	12.00
B3GNTL1	-0.20	-0.13	-0.34	-0.20	-0.20	0.00	-0.20	-0.18	-0.21	-0.20	-0.05	-0.20	-0.20	-0.08	-0.20	-0.20	0.38	1.22	0.29	Stable	12.00
SLC25A2	-0.79	-0.92	-0.58	-0.84	-1.00	-0.99	-0.82	-1.01	-0.38	-0.48	-0.81	-0.73	-0.80	-0.73	-0.34	-0.89	0.27	0.85	-0.23	Stable	2.00
PNPLA3	-0.18	-0.60	-0.09	0.36	0.02	0.04	0.15	-0.10	-0.15	0.25	0.24	-0.06	0.28	0.10	-0.03	-0.11	0.03	-0.90	-0.16	Stable	5.00
HHLA2	-0.82	-0.66	-0.76	-0.85	-0.27	-1.00	-0.82	-0.84	-0.80	-0.65	-0.60	-0.68	-0.83	-0.59	-0.93	-1.04	0.69	1.05	0.07	Stable	13.00
ARL6	-0.17	0.00	0.00	0.22	-0.27	-0.17	-0.17	0.00	-0.31	-0.17	0.22	0.48	-0.19	0.45	0.00	-0.17	0.88	5.03	2.33	Stable	13.00
PANK4	0.15	0.37	0.26	0.08	0.24	0.11	0.15	0.32	0.32	0.26	0.07	0.15	0.25	0.31	0.15	0.21	0.08	1.48	0.56	Stable	6.00
ENSSSCG00000027301	-0.43	-0.38	-0.40	-0.15	-0.21	-0.32	-0.43	-0.13	-0.29	-0.15	-0.33	-0.14	-0.14	-0.28	-0.14	-0.27	0.08	1.46	0.55	Stable	8.00
VPS35L	1.39	0.36	0.71	0.50	0.96	0.40	0.97	1.30	1.03	0.72	1.02	1.32	1.01	1.57	1.31	0.99	0.24	1.25	0.32	Stable	3.00
RPS17	-0.02	0.24	0.09	0.32	-0.17	0.46	0.08	0.20	0.36	0.27	0.36	0.15	0.28	0.08	-0.04	0.25	0.18	0.51	-0.96	Stable	7.00
ENSSSCG00000027374	-0.20	-0.13	-0.16	0.09	-0.18	-0.11	-0.09	-0.14	-0.16	-0.27	-0.14	-0.09	0.07	-0.13	-0.40	-0.07	0.12	2.03	1.02	Stable	6.00
GGCX	0.01	-0.10	-0.10	0.11	-0.10	0.04	-0.02	-0.10	0.05	-0.02	0.00	-0.08	-0.02	0.10	0.04	-0.03	0.95	1.19	0.25	Stable	3.00
HERPUD2	0.02	0.14	0.24	0.03	0.78	0.78	0.00	0.19	0.02	0.02	0.29	0.14	0.34	0.00	0.29	0.78	0.05	0.29	-1.80	Stable	18.00
UNC93A	-0.18	0.31	0.42	0.17	-0.31	0.26	-0.05	0.01	0.28	0.67	0.04	0.27	0.00	0.06	0.11	-0.20	0.95	1.07	0.10	Stable	1.00
FBXO22	-0.25	-0.43	-0.47	-0.39	-0.21	-0.47	-0.50	0.02	-0.20	-0.43	-0.45	-0.07	-0.08	-0.23	-0.43	-0.42	0.97	0.99	-0.02	Stable	7.00

RNF13	-0.61	0.43	-0.13	-0.67	-0.53	-1.02	-0.65	-0.13	-0.79	-0.92	-0.09	-0.84	-0.63	-0.62	-0.73	-1.09	0.11	0.56	-0.84	Stable	13.00
ESYT3	0.03	0.05	0.05	0.15	0.03	0.05	0.00	0.03	0.09	0.15	-0.03	-0.03	0.00	0.00	-0.11	0.03	0.44	0.42	-1.26	Stable	13.00
POLD3	0.72	0.62	0.79	0.71	0.16	0.15	0.50	0.22	0.39	0.14	0.42	0.21	0.14	0.97	0.16	0.39	0.06	1.88	0.91	Stable	9.00
DHCR24	-0.98	-0.14	-0.45	-0.38	-0.22	-0.50	-0.45	-0.45	-0.51	-0.39	-0.55	-0.46	-0.45	-0.21	-0.68	-0.36	0.50	1.17	0.22	Stable	6.00
TMEM107	0.04	0.03	0.02	0.14	-0.02	0.07	0.00	0.15	0.11	0.14	0.10	0.08	0.07	-0.05	0.15	-0.02	0.65	0.78	-0.36	Stable	12.00
PLCD1	0.11	0.11	0.54	0.90	0.22	0.26	0.11	0.94	-0.28	0.17	1.83	0.69	1.45	0.36	0.11	1.35	0.04	0.29	-1.78	Down	13.00
SPAG1	0.77	0.01	0.44	0.52	0.72	0.67	0.78	0.10	0.64	0.54	0.54	0.63	0.54	0.19	0.67	0.54	0.26	0.77	-0.38	Stable	4.00
USP16	-0.01	-0.04	-0.01	-0.03	-0.04	-0.01	-0.04	0.56	0.69	-0.03	-0.04	-0.03	-0.04	-0.03	-0.04	-0.04	0.16	-4.28	2.10	Stable	13.00
FAM172A	-0.14	-0.13	0.04	-0.19	-0.13	-0.13	-0.15	-0.14	-0.15	-0.14	-0.16	-0.14	-0.16	-0.13	-0.14	-0.14	0.26	0.81	-0.31	Stable	2.00
IL6R	0.18	0.25	0.53	0.40	0.54	0.37	0.32	0.29	0.14	0.54	0.54	0.40	0.54	0.23	0.48	0.54	0.01	0.63	-0.67	Stable	4.00
CDIPT	-0.17	-0.01	-0.12	-0.56	-0.21	-0.42	-0.52	0.59	0.08	0.22	0.23	-0.06	-0.21	0.38	0.40	-0.52	0.13	-0.42	-1.25	Stable	3.00
FUCA1	0.21	0.26	0.24	0.16	0.18	0.16	0.41	0.27	0.38	0.23	0.17	0.41	0.05	0.06	0.23	0.26	0.31	1.27	0.35	Stable	6.00
ENSSSCG00000027677	-0.11	-0.20	-0.47	-0.40	-0.03	-0.17	-0.05	-0.28	-0.07	-0.51	-0.25	0.09	-0.15	-0.56	-0.01	-0.47	0.86	0.92	-0.12	Stable	12.00
RNF183	0.12	0.36	-0.18	-1.20	-0.28	-0.44	-0.31	0.28	-0.29	-0.72	-0.12	-0.17	1.14	0.68	0.26	-1.09	0.13	-0.32	-1.63	Stable	1.00
ENSSSCG00000027741	-0.31	-0.52	-0.32	-0.34	-0.33	-0.29	-0.18	-0.61	-0.71	-0.57	-0.17	-0.18	-0.47	-0.48	-0.18	-0.35	0.36	1.23	0.30	Stable	6.00
MRFAP1	0.09	0.12	0.41	0.05	0.12	0.12	0.09	0.38	0.66	0.12	0.07	0.12	0.41	0.12	0.09	0.07	0.23	1.82	0.86	Stable	8.00
C12orf45	0.01	0.23	0.06	0.34	0.48	0.39	0.05	0.23	0.32	0.38	0.59	0.28	0.39	-0.02	0.32	0.03	0.01	0.41	-1.28	Down	5.00
CSKMT	-0.93	-0.68	-0.96	-0.66	-0.01	-0.85	-0.82	-0.82	-0.39	-0.82	-0.47	-0.36	-0.52	-0.75	-0.75	-0.91	0.16	1.32	0.40	Stable	2.00
ENSSSCG00000027852	0.38	0.30	0.21	0.51	-0.72	-0.19	0.51	0.34	0.30	0.19	0.51	0.00	0.20	0.54	-0.19	-0.38	0.13	21.09	4.40	Stable	2.00
HSD17B6	0.46	-0.12	-0.12	0.01	0.49	-0.23	-0.12	-0.38	0.05	1.18	0.28	1.17	0.55	0.54	0.21	0.67	0.05	0.12	-3.03	Stable	5.00
ERAP2	-0.08	-0.18	0.06	-0.02	-0.17	-0.18	0.05	0.22	-0.08	-0.18	0.02	0.02	0.14	-0.18	0.10	-0.18	0.41	0.14	-2.88	Stable	2.00
CA14	0.40	-0.01	0.18	0.00	0.00	0.20	0.40	0.20	0.20	0.00	0.00	0.40	0.00	0.20	0.20	0.00	0.06	2.95	1.56	Stable	4.00
FAM76A	0.02	0.06	0.08	0.10	0.25	0.10	0.06	0.02	0.23	0.24	0.10	0.06	0.10	0.04	0.20	0.08	0.32	0.69	-0.54	Stable	6.00
MVP	0.22	-0.03	0.29	0.67	0.04	0.50	0.36	0.36	0.44	0.18	0.18	0.24	0.11	0.19	-0.12	0.36	0.49	0.75	-0.42	Stable	3.00
ENSSSCG00000027970	0.25	0.20	-0.10	-0.20	-0.10	-0.13	0.01	-0.05	-0.21	0.15	0.13	0.10	-0.12	-0.20	0.19	0.01	0.69	-0.52	-0.94	Stable	3.00
ENSSSCG00000027991	-0.06	-0.67	-0.38	-0.07	-0.05	0.00	-0.14	-0.63	-0.88	-0.62	-0.44	-0.14	-0.27	-0.20	-0.14	-0.35	0.29	1.59	0.67	Stable	6.00
ENSSSCG00000028005	-0.66	-1.16	-0.59	-0.56	-0.63	-1.09	-0.37	-0.36	-0.22	-0.04	-0.69	-0.31	-0.90	-0.87	-1.16	-1.15	0.97	1.01	0.01	Stable	14.00
EP400	0.00	-0.08	0.00	-0.08	0.00	0.00	0.01	0.00	0.00	-0.16	0.00	0.00	0.00	0.00	-0.08	0.00	0.65	0.61	-0.71	Stable	14.00
MAP3K10	0.00	-0.60	0.00	0.00	0.00	0.00	0.00	0.00	-0.39	0.00	0.00	0.00	0.00	0.00	0.00	0.00	0.18	-1352.92	10.40	Stable	6.00
ZNF385C	0.13	0.03	0.00	0.58	0.39	0.39	0.04	0.00	0.01	0.13	0.66	0.13	0.28	0.14	0.01	0.13	0.01	0.13	-2.93	Down	12.00
ENSSSCG00000028085	-0.64	0.04	-0.32	-0.70	-0.85	-0.64	-0.64	-0.72	-1.08	-0.67	-0.75	-0.96	-0.85	-0.60	-0.94	-0.53	0.34	0.82	-0.28	Stable	8.00
SLC9B2	-0.26	-0.31	-0.51	-0.43	-0.16	-0.55	-0.56	-0.53	-0.55	-0.72	-0.53	-0.55	-0.46	0.07	-0.28	-0.19	0.43	0.81	-0.30	Stable	8.00
PRTFDC1	0.17	0.21	0.13	0.15	0.21	0.11	0.05	0.18	0.18	0.23	0.12	0.21	0.15	0.21	0.10	0.25	0.36	0.85	-0.23	Stable	10.00
THYN1	-0.02	-0.19	-0.12	-0.44	-0.31	-0.12	-0.22	0.00	-0.44	-0.25	-0.37	0.00	-0.51	0.00	-0.19	-0.51	0.07	0.47	-1.09	Stable	9.00
ENSSSCG00000028166	0.12	-0.34	-0.34	-0.29	-0.06	-0.20	-0.23	-0.32	0.00	0.07	-0.34	-0.23	0.00	-0.34	-0.22	-0.20	0.52	1.34	0.43	Stable	12.00
FH	0.20	0.33	0.12	0.40	0.49	0.41	0.71	0.09	1.05	0.02	0.58	-0.16	0.64	-0.25	0.19	0.45	0.78	0.86	-0.21	Stable	10.00
DNAH1	-0.22	0.58	0.37	0.09	0.26	0.48	-0.22	0.69	-0.12	0.11	-0.30	0.17	0.78	-0.22	-0.22	0.55	0.32	0.29	-1.76	Stable	13.00
FGD3	-0.25	-0.25	-0.47	-0.48	-0.47	-0.47	-0.47	-0.16	-0.16	-0.47	-0.47	-0.16	-0.47	0.00	-0.03	-0.47	0.01	0.52	-0.95	Stable	3.00
DNAJC28	0.04	0.00	-0.06	0.39	0.21	0.32	0.13	0.27	0.10	0.43	0.16	0.24	0.16	0.31	0.00	0.15	0.02	0.39	-1.38	Down	13.00
ENSSSCG00000028203	-0.17	0.00	0.00	0.02	0.00	0.41	-0.05	-0.52	-0.16	-0.10	-0.04	-0.12	-0.24	0.00	-0.21	-0.10	0.23	6.77	2.76	Stable	13.00
TREH	-0.03	-0.01	-0.03	0.01	-0.05	0.01	-0.03	-0.03	-0.06	0.03	-0.07	0.07	0.01	-0.06	-0.15	0.03	0.03	-11.61	3.54	Up	9.00
CEACAM20	0.49	0.53	0.23	0.25	0.00	0.57	0.61	0.74	0.69	0.64	0.42	0.61	0.43	0.55	0.61	0.12	0.10	1.47	0.56	Stable	6.00
RPN2	0.28	0.36	0.32	0.29	0.73	0.60	0.61	0.83	1.47	0.20	0.56	0.39	0.35	1.17	0.56	0.73	0.22	1.46	0.55	Stable	17.00
SKA3	0.29	0.35	0.06	-0.01	0.05	0.08	0.14	0.05	-0.04	0.34	0.03	0.41	0.10	0.14	0.10	0.05	0.94	1.04	0.06	Stable	11.00
AZI2	-0.03	-0.01	-0.01	-0.03	-0.03	-0.01	-0.03	0.00	-0.01	0.00	-0.11	-0.03	-0.01	-0.01	-0.01	-0.03	0.24	0.51	-0.97	Stable	13.00

ENSSSCG00000028277	0.56	0.40	0.00	0.00	0.00	0.84	0.00	0.56	0.00	0.90	0.45	0.00	0.45	0.56	0.05	0.00	0.70	0.80	-0.32	Stable	4.00
DR1	-0.35	-0.38	-0.55	-0.40	-0.33	-0.35	-0.19	-0.19	-0.17	-0.33	-0.09	-0.36	-0.36	-0.15	-0.33	-0.32	0.62	0.91	-0.14	Stable	4.00
MRPL9	-0.85	-0.57	-0.63	-0.96	-0.89	-0.80	-0.73	-0.86	-0.32	-0.97	-0.97	-0.72	-0.97	-0.69	-0.56	-0.97	0.00	0.72	-0.48	Stable	4.00
ACTG1	0.19	-0.19	-0.60	-0.06	-0.28	-0.31	0.30	-0.15	0.34	-0.58	0.37	-0.92	0.42	-0.10	-0.23	-0.55	0.37	0.23	-2.13	Stable	12.00
BST1	-0.10	0.01	0.15	-0.07	-0.28	-0.20	-0.51	0.01	-0.13	-0.51	-0.03	-0.06	0.12	-0.33	0.06	-0.24	0.59	0.65	-0.63	Stable	8.00
TMEM106C	-0.14	-0.08	-0.68	-0.07	0.05	0.28	-0.13	0.03	0.18	0.21	0.50	0.03	0.06	-0.24	-0.09	-0.16	0.04	-1.29	0.36	Stable	5.00
HSD17B4	0.30	0.50	0.37	0.70	0.50	0.50	0.42	0.50	0.56	0.59	0.59	0.61	0.92	0.50	0.50	0.54	0.02	0.74	-0.44	Stable	2.00
ENSSSCG00000028443	0.09	-0.47	-0.12	-0.23	-0.57	-0.21	-0.03	-0.14	-0.41	-0.05	-0.31	-0.07	-0.57	0.06	-0.16	-0.33	0.16	0.50	-1.00	Stable	7.00
ENSSSCG00000028479	0.57	0.84	-0.21	-0.05	0.90	0.38	0.86	0.44	0.34	0.26	0.84	0.10	0.20	0.39	0.02	0.44	0.91	1.05	0.08	Stable	17.00
ENSSSCG00000028501	-0.49	-0.40	-0.39	-0.44	-0.20	-0.18	-0.13	0.29	0.05	-0.32	-0.49	-0.15	-0.11	-0.26	-0.54	-0.25	0.77	0.87	-0.20	Stable	2.00
YIPF1	0.24	0.06	0.00	0.15	-0.53	-0.58	-0.36	0.19	0.19	-0.21	0.34	0.16	-0.46	0.11	0.16	0.05	0.17	-0.53	-0.91	Stable	6.00
HUS1	0.38	1.02	0.43	-0.20	0.23	-0.02	-0.17	1.07	0.97	-0.45	0.95	1.04	0.83	-0.05	1.05	0.29	0.36	1.76	0.81	Stable	18.00
ECM2	0.17	-0.03	-0.03	-0.03	-0.03	-0.03	-0.03	-0.02	-0.03	-0.03	-0.03	0.16	-0.03	0.18	0.34	-0.03	0.21	-9.13	3.19	Stable	3.00
SLC39A9	-0.16	-0.12	-0.17	-0.17	-0.17	-0.20	-0.17	-0.24	-0.30	-0.26	-0.12	-0.17	-0.17	-0.17	-0.35	-0.20	0.43	1.14	0.19	Stable	7.00
ABCD3	0.43	0.27	0.52	-0.11	-0.10	-0.05	-0.05	0.60	0.20	0.03	0.08	0.80	0.01	0.11	0.02	0.09	0.22	2.84	1.50	Stable	4.00
RARRES1	0.43	0.19	0.20	-0.05	0.24	0.39	0.43	-0.06	0.06	0.06	0.24	0.07	0.06	0.25	0.43	0.06	0.22	1.77	0.83	Stable	13.00
ENSSSCG00000028635	-0.03	-0.24	-0.12	-0.05	0.10	-0.19	-0.02	-0.17	-0.06	0.19	-0.18	-0.11	-0.14	-0.12	-0.15	-0.07	0.32	1.97	0.98	Stable	3.00
UTP4	0.00	0.00	0.00	0.00	0.00	0.00	0.00	0.00	0.00	0.00	0.00	0.00	0.00	0.06	0.00	0.00	0.32	8.13	3.02	Stable	6.00
CEL	-0.82	-0.49	-0.68	-0.69	-1.20	-0.92	-1.16	-1.16	-0.92	-0.67	-1.16	-0.93	-1.16	-1.16	-0.88	-0.88	0.72	0.96	-0.07	Stable	1.00
ENSSSCG00000028674	0.14	-0.31	-0.02	0.13	-0.08	0.05	-0.04	-0.47	-0.35	-0.19	-0.31	-0.34	-0.52	-0.07	-0.17	-0.13	0.89	0.92	-0.12	Stable	9.00
CCDC47	0.13	0.39	0.30	0.51	0.60	-0.09	0.31	0.22	0.21	0.29	0.19	0.73	0.58	0.27	0.54	0.47	0.30	0.73	-0.46	Stable	12.00
NEIL2	-1.49	-0.84	-0.24	-0.34	-0.49	-0.57	-1.59	-0.14	-0.83	-0.46	-1.00	-0.96	-0.45	-1.20	-0.27	0.05	0.23	1.57	0.65	Stable	14.00
CCDC102A	-0.09	-0.15	-0.15	-0.18	-0.18	-0.14	0.11	0.01	0.24	-0.31	-0.50	-0.15	-0.22	-0.12	-0.34	-0.05	0.08	0.28	-1.82	Stable	6.00
N6AMT1	-0.49	-0.71	-0.65	-0.28	-0.48	-0.31	-0.29	0.53	0.28	-0.33	-0.70	-0.03	0.08	-0.47	-0.38	-0.56	0.77	0.84	-0.26	Stable	13.00
NDC1	-0.30	0.06	-0.63	-1.20	-0.92	-0.42	-0.77	-0.37	-1.07	0.01	-0.54	-0.83	-1.18	-0.03	-0.82	-1.13	0.19	0.63	-0.66	Stable	6.00
EIPR1	-0.32	-0.33	-0.23	-0.16	-0.20	-0.20	-0.35	-0.15	-0.29	-0.36	-0.16	-0.22	-0.35	-0.25	-0.30	-0.13	0.18	1.25	0.32	Stable	3.00
LBP	-0.02	0.11	0.10	0.45	0.45	0.22	0.21	0.21	-0.02	0.21	0.11	0.11	0.21	-0.02	0.33	0.45	0.04	0.42	-1.26	Down	17.00
ENSSSCG00000028777	-1.15	0.49	0.44	0.84	0.52	0.55	-0.98	-0.25	-0.35	0.53	0.70	-0.03	0.76	0.01	-0.27	0.83	0.00	-0.44	-1.19	Down	7.00
ENSSSCG00000028784	-0.02	-0.37	-0.24	-0.29	0.03	-0.37	-0.47	-0.04	-0.16	-0.13	-0.33	0.14	-0.26	-0.01	0.03	-0.27	0.79	0.86	-0.21	Stable	7.00
ENSSSCG00000028794	0.07	0.34	0.20	0.30	0.07	0.23	0.30	-0.05	0.24	-0.11	0.34	0.30	0.01	0.14	0.30	0.19	0.71	1.17	0.22	Stable	6.00
SOD3	-1.15	-1.05	0.00	-0.41	-1.03	-0.59	-0.87	-1.15	-0.59	-1.15	-0.14	-0.84	-0.39	-1.15	-0.78	-0.68	0.33	1.29	0.37	Stable	8.00
SASS6	0.16	-0.01	-0.03	-0.12	-0.02	-0.23	0.08	-0.10	-0.17	0.07	-0.13	-0.18	-0.13	0.01	0.25	-0.11	0.04	-0.22	-2.18	Down	4.00
FAM83C	0.00	-0.01	-0.04	0.00	-0.06	-0.03	-0.03	-0.13	-0.25	-0.03	-0.03	0.00	0.00	-0.16	0.00	-0.06	0.19	2.78	1.47	Stable	17.00
HAVCR2	0.30	0.12	0.32	0.16	0.31	0.16	0.31	0.16	0.16	0.16	0.32	0.31	0.32	0.30	0.31	0.16	0.77	1.05	0.08	Stable	16.00
RB1CC1	0.00	0.21	0.15	-0.11	0.00	0.24	-0.06	0.12	-0.15	0.02	-0.02	-0.19	0.11	-0.01	-0.06	0.00	0.78	3.60	1.85	Stable	4.00
ECH1	0.07	0.00	-0.03	-0.05	-0.11	0.07	0.06	-0.10	0.00	-0.03	0.08	0.06	0.04	-0.16	0.06	0.05	0.48	-0.89	-0.16	Stable	6.00
ENSSSCG00000028962	-1.19	-1.29	-1.24	-0.82	-0.72	-0.50	-1.65	-0.40	-0.24	-0.66	-0.53	-1.78	-1.34	-0.16	-0.55	-0.10	0.90	1.04	0.06	Stable	12.00
PHACTR4	-0.07	-0.07	-0.07	-0.07	0.37	-0.07	-0.07	-0.07	0.40	0.37	0.18	-0.07	-0.07	-0.07	-0.04	0.12	0.28	-0.09	-3.46	Stable	6.00
CAD	-0.30	-0.13	-0.14	-0.27	-0.25	0.08	-0.05	-0.08	-0.40	-0.25	-0.31	-0.05	-0.22	-0.11	-0.10	-0.07	0.94	0.97	-0.04	Stable	3.00
ZNF367	0.33	0.42	0.12	0.42	0.63	0.42	0.32	0.19	0.41	0.46	0.42	0.53	0.50	0.42	0.20	0.58	0.00	0.61	-0.72	Stable	10.00
ALDH1A1	-0.47	-0.53	-0.16	-0.62	-1.02	-0.87	-0.47	-0.45	-0.57	-0.40	-0.80	-0.66	-0.54	-0.30	-0.47	-0.47	0.02	0.64	-0.65	Stable	1.00
ENSSSCG00000029039	0.36	0.51	0.18	0.18	0.30	0.09	0.20	0.23	-0.03	0.48	0.33	0.33	0.19	0.28	0.06	0.24	0.56	0.84	-0.26	Stable	11.00
IDI1	-0.72	-0.70	-0.77	-0.78	-0.91	-0.92	-0.93	-0.64	-0.94	-0.75	-0.41	-0.86	-0.67	-0.53	-0.59	-0.90	0.57	0.94	-0.09	Stable	10.00
PAFAH1B3	0.01	-0.09	-0.07	0.02	0.04	-0.01	0.00	-0.21	-0.15	-0.01	-0.01	0.00	0.04	-0.01	0.00	0.00	0.04	-8.88	3.15	Up	6.00
CACNB4	-0.20	0.00	-0.08	-0.20	-0.28	-0.28	-0.20	-0.20	-0.33	-0.20	-0.35	-0.28	-0.28	-0.32	-0.33	-0.28	0.23	0.78	-0.36	Stable	15.00

MTG1	0.09	-0.59	0.07	-0.72	-0.32	-0.04	-0.41	-0.38	0.07	-0.33	-0.11	-0.22	-0.22	-0.01	-0.68	-0.64	0.53	0.72	-0.48	Stable	14.00
HRH2	0.13	0.01	0.00	0.30	0.18	-0.10	0.17	0.35	0.23	0.12	0.41	0.01	0.42	0.14	0.22	0.18	0.68	0.83	-0.27	Stable	2.00
ENSSSCG00000029160	0.56	0.45	0.22	0.72	0.70	0.23	0.21	0.53	0.21	0.23	0.45	0.40	0.47	0.41	0.70	0.36	0.72	0.92	-0.11	Stable	7.00
ADI1	-0.52	-0.79	-0.16	-0.12	-0.22	-0.19	0.20	-0.25	-0.79	-0.44	-0.25	-0.08	-0.20	-0.49	-0.61	-0.28	0.15	1.90	0.93	Stable	3.00
DOK4	-0.08	-0.03	0.06	-0.23	-0.21	-0.12	-0.16	-0.29	-0.14	-0.33	-0.31	-0.02	-0.27	-0.26	-0.37	-0.19	0.43	0.75	-0.41	Stable	6.00
FAM200A	-0.30	0.46	-0.03	0.14	0.13	0.25	-0.31	-0.20	-0.40	0.04	0.30	-0.03	0.38	-0.34	-0.50	0.26	0.01	-1.10	0.14	Stable	3.00
SEC11A	-0.01	-0.07	-0.04	-0.13	-0.11	-0.12	-0.10	-0.07	-0.20	-0.09	-0.12	-0.12	-0.20	0.00	-0.04	-0.12	0.03	0.52	-0.94	Stable	7.00
DIP2B	0.35	-0.44	0.04	0.35	0.13	-0.13	0.35	0.01	0.52	0.12	0.09	0.48	-0.06	0.29	0.28	0.35	0.95	1.05	0.06	Stable	5.00
ENSSSCG00000029203	0.00	-0.10	-0.10	0.00	0.00	-0.10	-0.10	-0.10	0.00	0.00	0.00	0.00	0.00	0.00	-0.09	0.00	0.04	4.95	2.31	Up	7.00
ZNF276	0.48	0.13	-0.15	0.56	0.43	0.46	0.48	0.48	0.48	0.48	0.06	0.51	0.40	-0.29	-0.02	0.46	0.11	0.47	-1.08	Stable	6.00
NAV3	-0.40	-0.82	-0.61	-0.61	-0.20	0.00	-0.46	0.01	-0.82	-0.55	-0.41	-0.20	0.00	-0.61	-0.82	-0.41	0.06	1.90	0.93	Stable	5.00
ENSSSCG00000029257	0.16	0.14	0.17	0.28	0.44	0.19	0.16	0.15	0.00	0.23	0.40	0.40	0.31	0.12	0.11	0.19	0.00	0.42	-1.26	Down	2.00
NDNF	0.00	0.00	0.00	0.20	0.00	0.00	0.00	0.00	0.00	0.00	0.00	0.00	0.00	0.00	0.00	0.00	0.35	0.00	#NAME?	Stable	8.00
TIGIT	0.43	0.21	0.43	0.33	0.29	0.29	0.43	0.43	0.29	0.43	0.29	0.33	0.33	0.43	0.43	0.16	0.06	1.26	0.33	Stable	13.00
TOPAZ1	0.76	0.10	0.06	0.06	0.60	0.17	0.76	-0.01	0.27	0.31	-0.12	0.39	-0.02	0.66	0.76	0.08	0.14	2.28	1.19	Stable	13.00
PCDHAC2	0.65	0.45	0.40	0.86	0.76	0.73	0.83	0.29	0.35	0.24	0.58	0.83	0.75	0.42	0.05	0.95	0.03	0.60	-0.73	Stable	2.00
ENSSSCG00000029291	0.00	-0.12	-0.08	0.29	0.30	-0.07	0.14	-0.07	-0.13	-0.13	0.41	0.28	0.30	-0.12	-0.08	-0.12	0.03	-0.36	-1.47	Down	13.00
ALKBH4	0.30	0.15	0.29	0.01	0.16	0.27	0.30	0.28	0.29	0.01	0.16	0.30	0.02	0.12	0.16	0.16	0.06	1.75	0.81	Stable	3.00
STEAP3	0.01	-0.47	0.02	-1.41	0.05	0.08	-0.05	-0.25	-0.54	-0.36	-0.54	-0.76	-0.98	0.39	-0.28	-0.36	0.09	0.27	-1.86	Stable	15.00
FNDC4	0.45	0.01	-0.38	0.07	0.43	0.57	0.15	0.10	0.42	0.56	0.15	0.45	0.19	-0.41	0.33	0.17	0.11	0.26	-1.93	Stable	3.00
ITGB1BP1	0.25	-0.03	0.09	0.11	0.45	0.34	0.16	0.09	0.21	0.13	0.09	0.14	0.46	0.22	0.22	0.55	0.10	0.53	-0.93	Stable	3.00
PALLD	0.00	0.00	0.00	0.00	0.00	0.00	-0.02	0.00	0.00	0.00	0.00	0.00	0.00	0.00	0.00	0.00	0.35	#NAME?	Inf	Stable	14.00
MFSD11	0.27	0.32	1.04	0.33	1.11	1.30	0.39	0.47	0.34	0.53	0.63	0.48	1.03	0.50	0.34	0.23	0.16	0.65	-0.62	Stable	12.00
LIAS	-0.10	-0.10	-0.10	0.22	-0.05	0.04	0.04	-0.01	-0.01	-0.05	-0.10	-0.10	-0.10	-0.10	-0.05	-0.05	0.49	2.13	1.09	Stable	8.00
HUNK	0.07	0.07	-0.04	-0.15	-0.05	-0.06	-0.04	0.07	0.05	-0.04	-0.04	-0.04	-0.04	-0.04	0.07	-0.02	0.01	-0.47	-1.09	Down	13.00
CENPJ	-0.13	-0.34	-0.13	-0.25	-0.49	-0.13	-0.40	-0.13	0.34	0.12	-0.18	-0.46	-0.63	-0.29	-0.46	-0.57	0.32	0.59	-0.76	Stable	11.00
CAPN2	-0.70	-0.61	-0.70	-0.64	-0.55	-0.56	-0.62	-0.77	-0.58	-0.59	-0.62	-0.61	-0.54	-0.61	-0.38	-0.61	0.48	1.05	0.08	Stable	10.00
ZNF569	0.52	0.02	0.29	0.07	0.20	0.21	0.72	0.00	0.02	0.21	0.03	0.71	0.27	0.35	0.72	0.45	0.64	1.23	0.30	Stable	6.00
IPO13	-0.05	-0.05	0.00	-0.05	-0.02	-0.05	-0.02	-0.05	-0.05	0.06	-0.05	-0.02	-0.02	-0.05	-0.02	-0.02	0.36	1.58	0.66	Stable	6.00
KDM5A	0.06	-0.26	-0.23	-0.23	-0.45	-1.14	0.19	-0.72	-0.93	-0.75	1.45	-0.13	-0.72	0.15	-0.34	0.59	0.79	1.52	0.60	Stable	5.00
AVEN	0.00	-0.07	-0.19	-0.07	0.15	-0.03	-0.36	-0.28	0.00	-0.23	-0.15	0.06	0.05	0.00	-0.04	-0.02	0.20	4.00	2.00	Stable	7.00
CHST15	-0.18	-0.10	-0.28	-0.03	-0.16	-0.04	-0.23	-0.19	0.03	0.05	-0.15	-0.17	-0.18	-0.27	-0.15	-0.04	0.11	1.88	0.91	Stable	14.00
PON3	1.58	1.31	1.00	1.50	1.90	1.15	1.33	0.71	1.63	1.34	1.74	1.34	1.35	1.64	1.70	1.74	0.36	0.90	-0.15	Stable	9.00
RAB23	-0.44	-0.15	0.27	-0.37	-0.50	0.15	0.00	-0.09	-0.25	-0.36	0.13	-0.26	-0.37	-0.65	-0.42	-0.37	0.86	0.90	-0.15	Stable	7.00
PSD2	0.74	0.67	0.83	0.71	0.76	0.82	0.74	0.57	0.65	0.43	0.83	0.96	0.94	0.78	0.68	0.73	0.32	0.91	-0.13	Stable	2.00
ZNF239	0.89	0.64	0.21	1.22	-1.26	-0.19	1.19	1.73	1.42	-0.71	0.86	-0.50	1.76	0.89	0.91	-1.26	0.05	-84.63	6.40	Up	14.00
NFATC3	0.01	-0.01	0.02	0.51	0.32	0.17	0.06	0.32	0.10	0.01	0.46	0.06	0.30	-0.12	0.09	0.13	0.04	0.24	-2.05	Down	6.00
FAM124A	0.07	-0.08	-0.08	-0.08	0.06	0.02	0.07	-0.07	-0.09	-0.11	0.18	-0.26	-0.05	-0.05	-0.26	-0.08	0.70	1.59	0.67	Stable	11.00
ENSSSCG00000029600	-0.48	0.08	-0.21	-0.67	-0.59	-0.58	-0.37	-0.24	0.02	-0.31	-0.35	-0.37	-0.05	-0.18	-0.37	-0.02	0.21	0.60	-0.74	Stable	6.00
NECAB3	-0.30	-0.63	-0.61	-0.37	-0.49	-0.29	-0.53	-0.82	-0.74	-0.51	-0.50	-0.30	-0.54	-0.77	-0.23	-0.49	0.12	1.32	0.40	Stable	17.00
ENSSSCG00000029627	0.39	0.26	0.24	0.29	0.58	0.31	0.39	0.23	0.30	0.21	0.57	0.26	0.18	0.29	0.12	0.31	0.35	0.82	-0.29	Stable	13.00
PGAP4	-0.50	-0.31	0.08	-0.40	-0.20	-0.20	-0.14	-0.24	-0.39	-0.20	-0.46	0.02	-0.34	-0.26	-0.54	-0.29	0.73	1.12	0.17	Stable	1.00
ENSSSCG00000029705	0.60	-0.47	0.04	-0.50	0.37	0.28	0.97	0.61	1.18	0.23	-0.20	1.19	-0.50	-0.44	0.14	0.29	0.54	2.27	1.18	Stable	6.00
GCNT4	0.00	0.00	0.14	0.00	0.00	0.00	0.00	0.00	0.00	0.00	0.00	0.00	0.00	0.00	0.00	0.00	0.35	Inf	Inf	Stable	2.00
RNF7	0.01	-0.15	-0.08	0.00	0.06	0.00	0.01	0.06	0.01	-0.07	-0.15	-0.08	-0.08	-0.15	0.01	0.13	0.76	1.59	0.66	Stable	13.00

RPS9	-0.31	-1.13	-0.55	-0.47	-0.40	-0.64	-0.15	-1.25	-1.35	-0.77	-0.87	-0.15	0.02	-0.66	-0.15	0.04	0.20	1.72	0.78	Stable	6.00
PCYOX1	-0.23	0.27	0.75	-0.60	-0.40	-0.57	-0.23	-0.07	-0.47	-0.43	-0.50	-0.57	-0.67	-0.23	-0.54	-0.42	0.02	0.18	-2.47	Down	3.00
RHOBTB3	-0.07	0.03	-0.08	-0.25	-0.12	0.03	-0.03	0.06	-0.12	-0.05	-0.22	-0.18	-0.39	0.03	0.08	-0.10	0.02	0.08	-3.66	Down	2.00
VWA8	0.03	0.05	-0.28	-0.06	0.35	0.29	0.00	0.05	0.27	-0.28	0.31	0.15	0.12	0.31	0.23	-0.02	0.80	0.75	-0.41	Stable	11.00
NPHP4	1.07	0.74	0.51	0.90	1.68	0.60	0.88	0.57	0.45	0.63	1.45	0.88	1.53	0.92	0.88	1.56	0.04	0.65	-0.62	Stable	6.00
GRM5	-0.02	-0.26	-0.23	-0.10	-0.10	-0.20	-0.09	-0.19	-0.09	-0.19	-0.03	-0.19	-0.10	-0.19	-0.19	-0.07	0.34	1.30	0.38	Stable	9.00
DUSP14	0.00	0.00	0.00	0.02	0.32	0.44	0.35	0.01	0.30	0.33	0.07	0.02	0.00	0.00	0.00	0.00	0.43	0.55	-0.87	Stable	12.00
ZNF461	-0.35	0.15	-0.01	-0.27	-0.35	-0.21	-0.35	-0.27	-0.06	-0.27	0.07	-0.43	0.05	-0.03	-0.43	-0.14	0.81	0.88	-0.19	Stable	6.00
SCLT1	0.23	0.25	0.11	0.35	0.27	0.23	0.23	0.23	0.23	0.22	0.31	0.25	0.31	0.23	0.28	0.26	0.05	0.82	-0.29	Stable	8.00
ASIC4	-0.17	-0.17	-0.28	-0.24	-0.27	-0.28	-0.17	0.09	-0.10	-0.17	-0.17	-0.21	-0.24	-0.38	-0.22	-0.38	0.22	0.71	-0.49	Stable	15.00
PCGF3	0.11	0.02	0.00	0.16	0.10	0.07	0.15	-0.14	-0.04	0.09	0.20	0.30	0.24	0.14	-0.12	0.28	0.01	0.09	-3.54	Down	8.00
ARL10	-0.43	-0.15	0.04	-0.80	0.22	-0.98	-0.26	-0.51	-0.83	-1.35	-0.33	-0.07	-0.70	-0.33	-0.31	-0.31	0.36	0.64	-0.64	Stable	2.00
MED28	0.00	0.67	0.37	0.26	0.00	0.13	0.09	0.34	0.18	0.18	0.47	0.44	0.80	0.18	0.45	0.57	0.57	0.80	-0.32	Stable	8.00
C18orf21	0.23	-0.24	-0.33	0.28	0.47	-0.29	-0.34	0.09	-0.07	-0.05	0.28	-0.38	0.32	-0.17	0.32	0.10	0.29	-0.68	-0.55	Stable	6.00
PDE9A	0.22	0.03	-0.54	0.22	-0.21	-0.37	0.37	-0.20	0.16	0.05	0.23	0.07	-0.20	0.22	-0.04	-0.41	0.44	-0.38	-1.40	Stable	13.00
FOXRED1	-0.39	-1.55	-1.42	-0.93	-1.04	-0.87	-0.37	-0.78	-0.23	-1.07	-0.61	-0.33	-0.93	0.05	-0.39	-1.10	0.34	0.74	-0.44	Stable	9.00
PLEKHG2	-0.04	-0.15	-0.06	0.21	0.14	0.19	-0.07	-0.36	-0.33	-0.05	0.15	-0.07	0.19	-0.02	-0.07	0.08	0.00	-1.33	0.42	Stable	6.00
GINM1	0.12	0.06	0.10	0.10	0.00	0.00	0.12	0.10	0.10	0.10	0.04	0.07	0.07	0.04	0.12	0.00	0.03	1.99	0.99	Stable	1.00
SELENOF	-1.54	-0.14	-1.00	-0.09	-0.97	-1.22	0.83	-1.26	0.02	0.77	-1.24	-1.20	-1.08	-0.79	0.77	-0.13	0.54	0.60	-0.73	Stable	4.00
ENSSSCG00000030183	0.08	0.28	0.29	0.24	0.15	0.08	0.08	0.41	0.28	0.08	0.18	0.23	0.15	0.26	0.18	0.25	0.20	1.38	0.46	Stable	12.00
CD2AP	-0.04	-0.73	-0.12	-0.41	-0.54	-0.70	-0.21	-0.13	-0.26	-0.38	-0.26	-0.23	-1.04	-0.62	-0.68	-0.85	0.18	0.63	-0.66	Stable	7.00
MFNG	-0.18	0.09	-0.16	0.25	-0.04	0.77	0.21	-0.39	-0.12	0.43	0.21	0.28	-0.05	0.16	-0.17	0.43	0.01	-0.24	-2.04	Down	5.00
PIWIL2	-0.01	-0.08	0.09	-0.24	-0.14	-0.08	0.18	0.04	0.00	-0.05	0.10	-0.19	0.08	-0.10	-0.01	-0.06	0.12	-0.22	-2.16	Stable	14.00
EPM2AIP1	-0.45	0.05	0.32	0.74	0.53	0.55	-0.45	0.83	-0.42	0.17	0.85	-0.35	-0.17	-0.76	-0.45	-0.03	0.08	-0.58	-0.79	Stable	13.00
ENSSSCG00000030269	0.73	0.34	0.21	0.52	0.54	0.46	0.67	0.42	0.21	0.36	0.22	0.73	0.51	0.54	0.36	0.43	0.69	0.92	-0.11	Stable	12.00
GSTO2	-0.31	-0.21	-0.05	-0.42	0.03	-0.82	-0.24	-0.34	-0.34	-0.50	-0.43	-0.71	-0.82	-0.19	-0.53	-0.14	0.13	0.58	-0.78	Stable	14.00
RPRD2	0.00	0.16	0.16	0.03	0.03	0.02	0.00	0.00	0.00	0.00	0.00	0.00	0.00	0.00	0.00	0.00	0.30	3.96	1.98	Stable	4.00
ENSSSCG00000030300	0.17	0.32	0.33	0.17	0.10	0.16	0.60	0.75	0.84	0.26	0.33	0.12	0.07	0.26	0.58	0.17	0.01	2.77	1.47	Up	6.00
TCP11L1	0.18	-0.02	0.31	-0.12	0.07	0.16	0.15	0.21	0.15	0.30	-0.17	0.21	0.06	0.31	0.17	0.03	0.11	2.78	1.47	Stable	2.00
NDUFB10	-0.15	-1.41	-0.40	0.91	0.04	0.22	0.22	-0.19	0.02	0.13	0.13	-0.71	0.04	-0.11	-0.71	0.31	0.07	-2.55	1.35	Stable	3.00
SARM1	-0.45	-0.86	-1.11	-0.53	-0.64	-0.14	-0.58	-0.65	-0.59	-0.54	-0.49	-0.54	-0.59	-0.89	-1.22	-0.73	0.04	1.52	0.60	Stable	12.00
METTL9	-0.27	0.35	0.01	0.36	0.00	0.20	0.22	-0.13	0.17	-0.05	0.13	-0.30	0.25	-0.25	-0.37	0.23	0.27	-0.32	-1.66	Stable	3.00
USP31	-0.24	-0.03	-0.11	0.01	-0.11	-0.22	-0.03	-0.26	0.06	-0.14	-0.10	-0.20	-0.03	-0.19	-0.26	-0.03	0.56	1.32	0.40	Stable	3.00
C9orf78	-0.17	0.31	0.12	0.11	-0.08	0.21	-0.25	-0.43	-0.19	0.03	-0.50	-0.18	-0.17	-0.06	0.55	-0.09	0.63	0.18	-2.48	Stable	1.00
RAB42	0.07	0.11	-0.01	0.40	0.05	0.40	0.40	0.17	0.06	0.10	0.37	0.40	0.40	-0.28	0.42	0.40	0.06	0.37	-1.43	Stable	6.00
ENSSSCG00000030433	-0.32	0.28	-0.27	-0.96	-1.12	-0.85	-0.32	0.44	0.28	0.21	-0.41	-0.32	-1.05	0.15	-0.32	-0.81	0.01	0.02	-6.02	Down	6.00
YAE1	0.00	-0.40	0.00	-0.39	-0.81	-0.63	0.00	-0.36	-0.40	0.00	-0.40	0.00	0.00	-0.05	-0.82	-0.06	0.83	0.88	-0.18	Stable	18.00
FBL	0.06	0.57	0.13	0.99	0.51	0.74	-0.45	0.34	0.57	0.25	0.19	-0.45	0.52	0.37	-0.45	0.33	0.26	0.37	-1.45	Stable	6.00
CDCP2	-0.18	-0.10	0.09	-0.31	0.09	0.07	-0.33	-0.33	-0.28	0.29	-0.26	-0.38	-0.31	0.01	-0.13	-0.29	0.88	1.12	0.16	Stable	6.00
TMEM143	0.14	-0.10	0.05	0.20	-0.19	0.02	-0.05	-0.10	-0.10	0.02	-0.03	-0.05	-0.07	0.06	-0.05	-0.16	0.84	0.63	-0.68	Stable	6.00
HOXC6	-0.02	-0.09	0.01	0.51	0.43	0.08	0.16	0.41	0.28	0.92	0.73	0.32	0.40	0.16	0.24	0.45	0.01	0.30	-1.74	Down	5.00
TATDN1	-0.34	-0.17	0.44	-0.44	-0.44	-0.23	-0.23	-0.01	-0.11	-0.27	-0.29	-0.29	-0.29	-0.23	-0.01	-0.10	0.05	0.28	-1.85	Down	4.00
FNBP4	-0.01	-0.07	0.00	-0.02	-0.13	-0.13	-0.01	0.00	-0.01	-0.01	-0.07	-0.02	-0.08	-0.01	-0.01	-0.13	0.02	0.20	-2.36	Down	2.00
C7H6orf125	-0.11	0.02	0.29	-0.04	0.01	0.23	0.19	0.00	0.07	0.02	0.20	-0.05	-0.06	-0.03	-0.17	-0.06	0.99	1.02	0.03	Stable	7.00
GART	0.35	0.57	0.48	-0.54	-0.20	0.09	-0.70	0.50	0.02	0.03	0.20	-0.45	-0.07	-0.42	0.31	0.29	0.28	-1.72	0.78	Stable	13.00

MYBPH	0.15	0.02	0.41	0.44	0.18	-0.06	-0.40	-0.29	-0.26	0.10	-0.35	-0.16	0.71	-0.40	0.09	0.38	0.16	-0.56	-0.85	Stable	9.00
MACO1	0.15	-0.33	-0.36	-0.39	-0.03	-0.39	0.02	-0.13	0.49	0.46	-0.63	0.02	-0.58	-0.46	-0.35	-0.04	0.68	0.62	-0.68	Stable	6.00
ARID2	-1.01	-0.56	-0.67	-0.44	-0.64	-1.04	-0.60	-0.27	-0.51	-0.44	-0.74	-0.63	-0.90	-0.47	-0.40	-0.62	0.27	0.82	-0.28	Stable	5.00
FAM43B	0.33	-0.27	0.18	0.01	0.70	0.37	-0.19	-0.17	0.06	-0.02	-0.21	-0.19	0.64	0.26	-0.06	0.20	0.28	0.11	-3.19	Stable	6.00
ENSSSCG00000030709	0.55	0.35	0.33	0.00	0.08	0.51	0.55	0.29	0.53	0.19	0.34	0.38	0.09	0.55	0.55	0.03	0.01	2.27	1.18	Up	3.00
MPL	0.00	0.00	0.00	0.00	0.00	0.00	0.00	0.00	0.00	0.00	0.00	0.00	0.00	0.00	0.00	0.00	0.35	#NAME?	Inf	Stable	6.00
S100A1	-0.20	-0.25	-0.01	0.00	0.03	0.04	0.05	-0.44	-0.24	0.03	0.03	-0.27	0.03	-0.10	-0.20	0.03	0.03	19.31	4.27	Up	4.00
ENSSSCG00000031075	0.00	-0.20	0.00	-0.49	-0.25	-0.18	0.00	-0.20	0.00	-0.25	-0.49	-0.25	-0.49	-0.25	0.00	-0.49	0.00	0.22	-2.16	Down	1.00
ENSSSCG00000031091	-0.39	0.09	-0.94	-0.18	-0.57	0.29	-0.20	0.56	-0.42	-0.71	-0.32	-0.37	-0.15	-0.17	-0.73	-0.34	0.92	0.93	-0.10	Stable	9.00
METTL21C	0.05	0.04	-0.02	0.07	0.03	0.13	0.15	0.15	0.06	-0.04	-0.04	-0.03	0.05	-0.06	0.06	-0.04	0.28	3.49	1.80	Stable	11.00
C9orf85	0.52	0.01	-0.07	0.34	-0.04	0.38	0.50	-0.20	0.13	0.65	-0.57	-0.33	-0.11	-0.07	0.32	0.48	0.82	1.43	0.52	Stable	1.00
HILPDA	0.18	0.27	0.11	0.24	0.18	0.30	0.24	0.24	0.13	0.15	0.24	0.22	-0.02	0.26	0.09	0.18	0.97	1.01	0.01	Stable	18.00
ENSSSCG00000031117	0.13	0.17	0.26	0.12	0.24	0.17	0.65	0.15	0.28	0.29	0.09	0.36	0.00	0.15	0.15	0.15	0.41	1.36	0.44	Stable	2.00
TSEN15	0.18	0.09	0.25	0.10	0.10	0.15	0.01	0.18	0.37	0.17	0.12	0.25	0.10	0.37	0.04	0.10	0.38	1.35	0.44	Stable	9.00
PPIC	0.07	-0.51	-0.44	-0.26	-0.51	-0.51	-0.41	-0.51	-0.10	-0.38	-0.37	-0.38	-0.27	-0.51	-0.49	-0.37	0.82	0.95	-0.08	Stable	2.00
ENSSSCG00000031141	0.27	-0.41	-0.14	-0.20	-0.08	-0.07	0.13	-0.28	0.38	0.13	0.03	-0.05	-0.07	0.19	0.13	0.10	0.59	-1.25	0.32	Stable	9.00
RAMP3	0.06	-0.69	-0.68	-0.94	-0.01	-0.09	0.26	-0.57	0.02	-0.77	-0.90	-0.70	0.76	-0.53	-0.74	0.23	0.84	1.18	0.24	Stable	18.00
ENSSSCG00000031163	0.23	-0.31	-0.13	-0.15	-0.03	0.07	0.23	0.23	0.23	-0.19	0.28	0.11	0.07	0.02	-0.30	0.05	1.00	1.00	0.00	Stable	2.00
ENSSSCG00000031169	0.83	0.33	0.40	0.68	0.81	0.56	0.81	0.93	0.80	0.86	0.71	0.81	0.36	0.49	0.81	0.42	0.82	1.04	0.05	Stable	6.00
KCNMB4	0.01	-0.45	-0.38	0.94	0.59	-0.32	-0.33	0.01	0.35	0.94	0.53	0.04	0.94	-0.65	0.45	0.06	0.02	-0.27	-1.90	Down	5.00
FBP1	0.03	-0.16	-0.12	-0.14	-0.06	-0.24	-0.06	-0.26	-0.30	-0.08	0.01	-0.20	-0.18	-0.16	-0.19	-0.08	0.51	1.26	0.34	Stable	10.00
CHML	0.07	-0.01	-0.01	-0.27	0.05	0.12	-0.05	-0.17	0.31	-0.41	-0.05	-0.34	-0.05	0.03	-0.10	0.70	0.77	-0.31	-1.70	Stable	10.00
ENSSSCG00000031269	-0.01	0.00	0.03	0.05	0.10	0.00	-0.04	0.19	0.19	0.03	0.03	0.03	0.03	0.11	-0.03	0.05	0.67	1.40	0.48	Stable	7.00
MRPL57	-0.41	-0.21	-0.23	-0.03	0.05	-0.16	-0.24	-0.18	-0.21	-0.16	-0.06	-0.26	0.13	0.00	0.23	0.17	0.20	3.97	1.99	Stable	11.00
ENSSSCG00000031288	0.26	0.24	-0.49	-0.21	0.17	0.21	0.48	0.24	0.34	0.24	-0.03	-0.18	0.16	0.22	-0.14	0.42	0.73	1.48	0.56	Stable	11.00
ENSSSCG00000031292	-0.28	0.07	-0.14	0.08	0.27	0.44	0.25	0.42	0.25	0.55	0.36	0.02	0.05	0.21	-0.04	0.19	0.17	0.37	-1.44	Stable	12.00
ENSSSCG00000031342	-0.16	-0.33	-0.26	0.00	-0.26	0.00	-0.31	-0.31	-0.31	-0.09	-0.20	-0.31	-0.15	-0.16	-0.31	-0.30	0.06	1.63	0.71	Stable	6.00
CMKLR1	0.51	0.24	0.51	0.48	0.38	0.31	0.41	0.28	0.26	0.18	0.42	0.17	0.37	0.57	0.40	0.50	0.45	1.14	0.19	Stable	14.00
DYNLT5	-0.15	0.00	0.00	-0.13	-0.13	-0.58	-0.06	-0.32	0.00	-0.21	-0.12	-0.21	-0.13	-0.07	-0.32	-0.12	0.24	0.56	-0.83	Stable	6.00
DMAC2L	-0.51	-0.16	-0.51	-0.40	-0.45	-0.49	-0.51	-0.34	-0.51	-0.46	-0.18	-0.41	-0.47	-0.34	-0.51	-0.30	0.64	1.07	0.10	Stable	1.00
CELSR1	0.02	0.28	-0.08	-0.04	-0.10	-0.08	-0.05	-0.14	-0.11	-0.08	-0.06	-0.05	-0.05	-0.12	0.26	-0.06	0.28	-0.09	-3.43	Stable	5.00
ENSSSCG00000031365	0.25	0.44	0.28	0.25	0.14	0.12	0.14	0.18	0.18	0.36	0.21	0.36	0.28	0.16	0.21	0.16	0.95	0.99	-0.02	Stable	3.00
AADAC	-0.21	0.00	-0.13	0.00	-0.15	-0.35	-0.21	-0.08	-0.21	-0.21	-0.07	0.00	0.00	0.00	-0.42	-0.29	0.75	1.17	0.22	Stable	13.00
STKLD1	-1.03	-0.51	-1.36	-0.98	-1.21	-0.82	-1.04	-1.04	-0.92	-0.74	-0.91	-1.14	-0.91	-0.94	-1.13	-1.13	0.89	1.01	0.02	Stable	1.00
ZBTB34	0.19	0.68	0.66	0.12	0.10	-0.17	0.20	0.31	0.14	-0.27	0.01	0.28	0.35	0.23	0.41	0.37	0.04	3.59	1.84	Up	1.00
KDELR2	-0.11	-0.26	0.01	-0.05	0.08	-0.26	-0.02	0.01	-0.18	-0.06	0.08	-0.25	-0.13	-0.20	-0.15	0.08	0.43	1.82	0.86	Stable	3.00
MRPS14	-0.08	-0.07	-0.16	0.00	-0.22	-0.22	0.01	0.00	0.06	-0.01	-0.12	-0.08	-0.22	-0.11	-0.10	-0.22	0.08	0.41	-1.28	Stable	9.00
GRAMD4	-0.24	0.00	-0.12	-0.24	-0.24	-0.12	-0.24	-0.24	-0.12	-0.12	-0.12	-0.12	-0.12	-0.24	0.00	-0.24	0.74	0.91	-0.14	Stable	5.00
CLP1	-0.67	-0.31	-0.30	-0.67	-0.67	-0.51	-0.67	-0.67	-0.67	-0.18	-0.52	-0.53	-0.55	-0.47	-0.21	-0.47	0.85	0.97	-0.05	Stable	2.00
PTK6	0.63	0.08	0.60	0.81	0.88	0.52	0.07	0.19	0.81	0.76	0.68	0.82	0.67	0.30	0.66	0.73	0.02	0.57	-0.81	Stable	17.00
ENSSSCG00000031483	-0.27	0.02	-0.28	-0.17	-0.09	-0.17	-0.03	0.02	0.03	-0.01	-0.02	-0.56	-0.11	-0.02	0.01	-0.02	0.34	0.45	-1.15	Stable	10.00
ENSSSCG00000031485	-0.02	-0.32	-0.04	0.28	0.28	0.25	0.07	0.02	0.04	0.30	0.48	0.13	0.31	0.06	0.07	0.31	0.00	-0.05	-4.37	Down	3.00
FNTA	-0.23	-0.29	-0.09	-0.14	-0.43	-0.09	-0.35	-0.27	-0.31	-0.28	-0.18	-0.33	-0.46	-0.46	-0.19	-0.34	0.92	0.98	-0.03	Stable	17.00
CCDC28B	-0.19	-0.24	-0.10	-0.40	-0.45	-0.66	-0.41	-0.41	-0.46	-0.45	-0.39	-0.41	-0.40	-0.02	-0.19	-0.41	0.01	0.56	-0.83	Stable	6.00
DPPA2	-0.58	-0.27	-0.76	-0.58	-0.56	-0.58	-0.58	-0.54	-0.56	-0.29	-0.56	-0.81	-0.58	-0.52	-0.58	-0.58	0.80	0.97	-0.04	Stable	13.00

CHCHD6	0.40	0.00	0.23	0.18	0.00	0.23	0.17	0.00	0.06	0.06	0.06	0.38	0.24	0.15	0.46	0.29	0.96	1.02	0.03	Stable	13.00
ZNF462	0.11	0.11	0.15	-0.01	-0.01	0.15	-0.47	0.07	-0.56	0.15	0.04	0.07	0.04	0.07	0.11	-0.09	0.40	-1.21	0.27	Stable	1.00
ZNF570	0.56	0.36	0.51	-0.11	0.12	-0.08	0.67	-0.06	0.16	0.11	0.04	0.63	0.42	0.49	0.63	0.38	0.10	2.20	1.14	Stable	6.00
SFT2D2	0.03	0.04	-0.31	0.66	0.86	0.14	0.33	-0.12	0.45	0.38	0.13	-0.03	0.13	-0.09	0.15	0.86	0.05	0.15	-2.71	Stable	4.00
RNASE1	0.07	-0.10	-0.06	-0.20	-0.20	-0.20	-0.05	0.00	0.07	0.06	-0.20	-0.07	-0.20	0.07	0.02	-0.20	0.00	-0.02	-5.67	Down	7.00
SRP9	-0.36	-0.35	-0.20	0.21	-0.22	0.17	-0.05	-0.02	-0.21	-0.46	-0.26	-0.16	-0.19	-0.19	-0.04	-0.03	0.52	1.53	0.61	Stable	10.00
DOLPP1	0.48	0.76	0.19	0.62	1.04	0.59	0.52	0.36	0.67	0.88	0.86	0.14	0.66	0.40	0.83	0.54	0.27	0.79	-0.34	Stable	1.00
GFUS	0.13	-0.09	-0.13	-0.22	0.79	0.63	-0.24	0.19	-0.42	-0.36	-0.27	-0.25	-0.37	0.14	-0.10	-0.20	0.86	2.06	1.04	Stable	4.00
MACROH2A2	0.29	-0.54	0.18	0.04	-0.13	0.33	-0.17	0.36	0.16	-0.11	-0.18	0.25	-0.06	0.33	0.24	-0.12	0.43	76.07	6.25	Stable	14.00
RAB39A	0.16	0.02	0.01	-0.01	-0.07	0.17	0.04	0.05	0.08	0.05	0.07	0.00	-0.11	0.24	0.08	0.22	0.38	2.11	1.07	Stable	9.00
LYNX1	-1.28	-1.95	-1.40	-1.85	-1.20	-1.11	-1.11	-0.63	-0.55	-1.48	-1.53	-1.55	-1.66	-0.89	-1.41	-1.38	0.11	0.78	-0.36	Stable	4.00
AKAP12	0.20	-0.15	0.24	-0.29	-0.29	-0.09	0.04	0.09	0.09	0.11	0.03	0.31	-0.29	0.11	0.14	-0.29	0.06	-0.95	-0.07	Stable	1.00
MGST2	-0.03	-0.13	0.11	0.09	-0.02	0.28	0.13	0.13	0.13	0.12	-0.02	-0.23	-0.02	-0.02	0.02	0.15	0.99	0.99	-0.02	Stable	8.00
ENSSSCG00000031751	0.05	0.08	0.05	0.05	0.03	0.00	0.05	0.10	0.08	0.10	0.00	0.10	0.10	0.10	0.08	0.10	0.50	1.22	0.28	Stable	7.00
CTTN	-0.22	-0.04	0.00	-0.06	-0.21	-0.25	-0.06	-0.35	-0.24	-0.02	0.00	-0.11	-0.12	-0.13	-0.02	-0.02	0.53	1.35	0.43	Stable	2.00
C4orf3	-0.49	-0.49	-0.46	-0.32	-0.49	-0.82	-0.71	-0.73	-0.76	-0.60	-0.48	-0.49	-0.64	-0.49	-0.52	-0.80	0.97	1.00	0.01	Stable	8.00
ACSL5	-0.22	0.00	0.00	-0.22	-0.22	-0.22	-0.11	-0.22	-0.22	-0.22	-0.11	-0.10	0.00	-0.22	-0.09	0.00	0.98	0.99	-0.01	Stable	14.00
TMEM139	-0.33	-0.12	-0.24	-0.01	-0.41	-0.32	-0.43	-0.22	-0.40	-0.49	-0.23	-0.41	-0.09	-0.27	-0.31	-0.22	0.77	1.07	0.10	Stable	18.00
ZKSCAN5	0.59	-0.07	0.12	0.17	0.24	0.34	0.25	0.00	0.58	0.48	0.50	-0.03	0.18	0.30	0.28	0.27	0.91	0.95	-0.07	Stable	3.00
ENSSSCG00000031810	0.12	0.95	0.19	0.69	0.76	0.32	-0.02	0.83	0.75	0.66	0.47	0.85	0.76	0.38	0.87	0.54	0.44	0.81	-0.31	Stable	7.00
IFN-OMEGA-3	0.00	0.08	0.00	0.16	0.08	0.16	0.00	0.07	0.00	0.08	0.16	0.15	0.16	0.07	0.00	0.16	0.00	0.20	-2.34	Down	1.00
REM2	-0.18	0.04	-0.09	0.06	0.01	-0.16	-0.12	-0.09	-0.18	-0.06	0.08	-0.06	0.01	-0.18	0.01	0.04	0.05	10.60	3.41	Up	7.00
PAPSS1	-0.31	0.03	0.18	0.05	0.03	0.03	0.02	0.22	0.28	0.20	0.03	0.03	0.04	0.16	0.32	0.08	0.51	1.82	0.87	Stable	8.00
ENSSSCG00000031916	-0.01	-0.36	-0.27	-0.17	-0.21	-0.14	-0.21	0.05	-0.36	-0.22	-0.30	-0.21	-0.06	-0.16	-0.21	-0.21	0.99	1.00	-0.01	Stable	6.00
RIMS2	-0.05	0.04	0.04	-0.04	0.01	0.03	-0.03	0.01	-0.03	-0.01	-0.06	-0.06	-0.06	-0.05	-0.03	-0.06	0.33	0.40	-1.33	Stable	4.00
ENSSSCG00000031979	-0.37	-0.98	-0.56	-0.55	0.07	-0.40	-1.15	-0.45	-0.23	-0.61	-0.67	-0.64	-0.61	-0.29	-1.12	-0.53	0.36	1.31	0.38	Stable	9.00
ENSSSCG00000031998	0.23	0.89	0.68	0.70	0.69	0.39	0.46	0.95	0.45	1.01	0.36	0.91	1.01	0.87	0.81	0.61	0.75	0.94	-0.09	Stable	9.00
ENSSSCG00000032003	0.11	0.07	-0.32	-0.32	-0.55	-0.01	0.04	-0.46	-0.33	0.18	-0.05	-0.37	-0.37	-0.13	0.09	-0.22	0.42	0.55	-0.87	Stable	2.00
SH3BGRL2	-0.27	0.38	-0.49	-0.26	0.20	-0.24	-0.18	-0.10	-0.14	0.05	0.04	-0.25	0.03	0.10	0.37	-0.28	0.71	0.47	-1.09	Stable	1.00
GCHFR	0.00	0.23	0.15	-0.22	-0.08	-0.75	0.00	-0.33	-0.03	-0.05	0.42	-0.26	-0.05	0.23	-0.27	-0.75	0.19	0.01	-6.78	Stable	1.00
ENSSSCG00000032023	0.34	-0.16	-0.60	-0.18	-1.37	-0.22	-0.09	-0.39	-0.14	-0.14	-0.37	0.07	-0.81	-0.15	-0.09	-0.24	0.22	0.39	-1.36	Stable	6.00
URB1	-0.14	-0.05	-0.38	-0.06	-0.17	-0.16	-0.01	0.04	0.18	-0.12	0.24	-0.24	0.02	0.05	-0.04	-0.05	0.77	0.66	-0.60	Stable	13.00
ENSSSCG00000032036	0.61	0.90	0.77	0.79	-1.11	-0.11	0.80	0.91	0.28	0.21	-0.81	-0.09	0.49	0.77	0.56	-1.18	0.01	-3.10	1.63	Up	17.00
SCRG1	0.16	-0.29	-0.46	0.21	-0.54	-0.44	0.45	-0.20	-0.01	0.75	-0.46	-0.29	0.19	0.28	0.06	-0.46	0.52	0.00	-11.57	Stable	14.00
ST8SIA5	-0.26	0.14	0.03	0.27	0.19	0.14	0.08	0.10	0.20	0.19	0.27	-0.15	0.10	0.38	0.35	0.03	0.97	0.97	-0.04	Stable	1.00
RO60	0.10	0.37	0.43	0.43	0.43	0.37	0.00	0.43	0.37	0.19	0.37	0.24	0.48	0.43	0.24	0.43	0.33	0.81	-0.31	Stable	10.00
THEM6	-0.25	-0.33	-0.64	-0.45	-0.76	-0.72	-0.32	-0.40	-0.09	-0.15	-0.25	-0.35	-0.15	-0.55	-0.14	-0.41	0.56	0.84	-0.25	Stable	4.00
MRPL37	-1.17	-0.70	-1.43	-1.83	-0.96	-0.42	-1.16	-1.16	-1.03	-1.46	-1.61	-1.63	-1.85	-0.73	-1.63	-1.83	0.16	0.78	-0.36	Stable	6.00
ENSSSCG00000032108	-0.12	-0.27	-0.15	-0.05	-0.11	-0.05	-0.12	-0.27	-0.20	-0.11	-0.11	-0.16	-0.11	-0.31	-0.09	-0.11	0.02	1.89	0.92	Stable	4.00
ENSSSCG00000032110	0.17	0.85	-0.23	0.43	-0.26	0.31	0.09	0.09	0.43	0.66	0.09	0.17	0.09	0.15	-0.29	0.13	0.79	0.79	-0.34	Stable	1.00
OGFOD3	0.15	0.07	0.10	0.16	0.40	0.26	0.21	-0.24	-0.06	0.26	-0.19	0.34	0.20	-0.04	0.24	0.41	0.07	0.23	-2.13	Stable	12.00
DUT	0.00	0.01	0.01	0.01	0.00	0.00	0.00	0.03	0.03	0.02	-0.01	0.01	0.00	0.01	0.00	0.02	0.40	1.70	0.77	Stable	1.00
SMURF2	-0.06	-0.11	-0.06	0.00	-0.03	-0.08	-0.12	-0.04	-0.04	0.00	-0.03	-0.05	-0.11	-0.08	-0.27	-0.04	0.10	2.25	1.17	Stable	12.00
NKAIN1	0.04	-0.15	0.05	0.21	-0.20	-0.57	0.08	0.28	-0.37	-0.27	0.02	0.08	0.21	-0.01	-0.11	0.20	0.89	0.57	-0.80	Stable	6.00
DBI	-0.21	-0.27	0.28	-0.57	-0.17	0.04	-0.37	-0.27	-0.18	0.40	-0.36	0.11	-0.20	-0.02	-0.30	0.13	0.50	2.18	1.13	Stable	15.00

ENSSSCG00000032231	-0.22	-1.18	-0.62	-0.07	-0.69	-0.06	-0.44	-0.92	-1.02	-0.41	-0.23	-0.44	-0.46	-0.57	-0.44	-0.56	0.05	1.85	0.89	Stable	6.00
LIMA1	0.00	0.00	0.00	0.00	0.00	0.11	0.00	0.00	0.11	0.00	0.00	0.11	0.00	0.00	0.11	0.00	1.00	1.00	0.00	Stable	5.00
ENSSSCG00000032297	-0.04	-0.32	-0.13	-0.05	0.31	0.00	0.90	1.06	1.15	0.62	0.23	0.61	0.31	1.12	-0.04	-0.35	0.35	2.19	1.13	Stable	1.00
ENSSSCG00000032311	0.24	-0.19	0.05	-0.01	0.11	0.24	-0.10	-0.19	-0.19	0.07	-0.06	-0.10	0.08	0.50	-0.19	0.50	0.33	-0.11	-3.18	Stable	15.00
POGLUT1	0.00	0.00	0.06	-0.28	-0.34	0.00	0.06	0.00	-0.15	-0.10	-0.34	-0.21	-0.42	-0.31	0.06	0.00	0.03	0.16	-2.65	Down	13.00
SULT1B1	-0.24	-0.08	0.09	0.09	0.01	0.09	-0.24	0.09	-0.08	0.09	0.09	0.09	0.09	-0.05	0.09	0.09	0.03	-0.66	-0.60	Stable	8.00
LITAF	0.17	0.44	-0.38	-0.26	0.09	0.43	0.01	-0.12	0.18	0.46	0.11	0.42	0.06	0.06	0.49	0.25	0.51	0.54	-0.89	Stable	3.00
ENSSSCG00000032422	0.32	0.14	0.23	-0.04	0.12	0.46	0.23	0.54	0.29	0.47	-0.27	0.26	0.02	0.05	0.49	-0.01	0.17	2.26	1.18	Stable	18.00
NUDT16	-0.34	-0.77	-0.77	-0.91	-0.46	-0.68	-0.17	-0.30	-0.42	-0.46	-0.46	-0.46	-0.46	0.25	-0.76	-0.20	0.49	0.80	-0.33	Stable	13.00
ENSSSCG00000032436	-0.33	0.03	0.04	-0.11	0.09	0.03	-0.04	0.11	0.07	-0.02	-0.05	-0.05	0.00	-0.57	0.00	0.04	0.40	10.47	3.39	Stable	2.00
KIAA0895L	-0.53	-0.28	0.00	-0.28	-0.27	-0.27	-0.27	-0.22	0.00	-0.53	-0.28	-0.27	-0.26	-0.27	-0.28	-0.31	0.29	0.75	-0.41	Stable	6.00
ENSSSCG00000032450	-0.47	-0.13	-0.15	-0.50	-0.18	-0.29	-0.39	-0.48	-0.39	-0.52	-0.25	-0.46	-0.32	-0.31	-0.46	-0.25	0.98	1.00	-0.01	Stable	12.00
WFS1	-0.52	0.09	0.17	0.41	0.37	0.09	-0.50	0.10	0.01	-0.02	0.36	0.32	0.18	-0.02	0.00	0.42	0.01	-0.32	-1.64	Down	8.00
ZADH2	0.40	0.27	0.24	-0.02	-0.02	0.30	0.20	0.21	0.19	0.24	0.22	0.15	0.41	0.20	-0.01	0.41	0.97	1.01	0.02	Stable	1.00
ING5	0.33	0.23	-0.21	-0.33	-0.21	0.33	-0.34	-0.25	0.09	0.32	-0.21	-0.21	-0.03	-0.52	0.13	-0.21	0.98	0.95	-0.08	Stable	15.00
ZNF317	0.00	0.00	0.00	0.00	0.00	0.00	0.00	0.00	0.00	0.03	0.00	0.00	0.00	0.00	0.00	0.00	0.35	0.00	#NAME?	Stable	2.00
TIGD2	0.29	-0.44	0.01	0.32	-0.49	-0.04	0.00	-0.24	-0.42	-0.74	-0.62	-0.55	-0.38	-0.33	-0.60	-0.32	0.41	0.62	-0.70	Stable	8.00
GNA12	-0.08	0.12	0.26	0.15	0.21	0.19	-0.10	0.16	-0.04	-0.07	0.48	0.19	0.48	-0.04	-0.12	0.09	0.04	0.10	-3.38	Down	3.00
ENSSSCG00000032573	0.43	0.73	0.93	0.81	0.43	0.11	0.43	-0.07	-0.08	0.09	0.44	0.21	0.43	0.87	0.43	0.43	0.58	1.24	0.32	Stable	4.00
TNFRSF17	0.35	-0.49	-0.19	-0.43	-0.07	-0.41	0.11	0.72	0.14	-0.08	0.32	-0.18	0.03	0.17	-0.19	-0.01	0.27	-0.77	-0.37	Stable	3.00
ENSSSCG00000032591	0.10	0.50	0.39	0.13	0.55	0.35	0.30	0.60	0.27	0.19	0.39	0.16	0.00	0.57	0.00	0.38	0.49	1.26	0.34	Stable	2.00
RAP1B	-0.24	-0.19	-0.22	-0.27	-0.18	-0.21	-0.23	-0.11	-0.05	-0.31	-0.40	-0.14	-0.27	0.01	-0.40	-0.17	0.27	0.74	-0.43	Stable	5.00
ALPL	0.13	-0.04	-0.13	0.16	-0.24	0.16	0.05	-0.22	0.11	0.16	0.17	0.05	-0.04	-0.05	0.11	-0.07	0.51	-0.07	-3.77	Stable	6.00
SNAI1	-0.28	-0.51	-0.71	-0.38	-0.55	-0.61	-0.27	-0.45	-0.57	-0.61	-0.96	-0.48	-0.39	0.56	-0.23	-0.27	0.18	0.58	-0.79	Stable	17.00
ENSSSCG00000032650	-0.50	-0.58	-0.52	-0.39	-0.39	-0.33	-0.60	-0.66	-0.51	-0.39	-0.38	-0.48	-0.41	-0.09	-0.64	-0.26	0.08	1.36	0.44	Stable	16.00
BOK	-0.23	-0.37	-0.56	-0.70	-0.56	-0.32	-0.64	-0.79	-0.60	-0.29	-0.56	-0.56	-0.21	-0.66	-0.37	-0.56	0.54	1.12	0.16	Stable	15.00
CYP4V2	-0.96	-0.89	-0.63	-0.43	-0.79	-0.42	-0.63	-0.98	-1.15	-0.53	-0.59	-0.96	-0.17	-0.63	-0.91	-0.18	0.01	1.67	0.74	Stable	15.00
ANKRD66	-0.18	-0.31	0.00	-0.31	-0.31	-0.15	-0.16	-0.01	-0.02	-0.16	-0.17	-0.16	-0.30	-0.18	-0.30	-0.30	0.12	0.62	-0.69	Stable	7.00
ACER3	-0.12	-0.06	-0.30	0.12	-0.25	0.67	0.20	0.31	0.87	0.78	0.27	0.16	0.78	-0.28	-0.14	0.31	0.14	0.17	-2.57	Stable	9.00
NDST1	-0.06	0.05	-0.10	0.05	-0.16	-0.05	-0.10	-0.10	-0.03	-0.05	0.02	-0.09	-0.07	0.00	0.06	-0.10	0.54	0.62	-0.68	Stable	2.00
ZBTB43	0.26	0.38	0.21	0.18	0.07	0.13	0.19	0.30	0.33	0.48	-0.02	0.25	0.10	0.39	0.12	0.09	0.10	1.71	0.78	Stable	1.00
PYCR3	-0.11	-0.13	-0.07	-0.14	-0.06	-0.05	-0.07	-0.11	0.03	-0.15	-0.11	-0.14	-0.15	-0.08	-0.13	-0.12	0.19	0.72	-0.47	Stable	4.00
PNPO	-0.25	-0.05	-0.23	-0.64	-0.89	-0.64	-0.42	0.06	-0.10	-0.62	-0.10	-0.41	-0.61	-0.85	-0.42	-0.84	0.03	0.48	-1.07	Down	12.00
ZC3HAV1L	-0.30	0.04	-0.62	-0.60	-0.60	-0.23	-0.33	-0.65	-0.06	-0.38	-0.18	-0.60	-0.65	-0.28	-0.58	-0.54	0.29	0.74	-0.44	Stable	18.00
GTF3C2	-0.35	0.12	-0.13	-0.41	-0.41	-0.43	-0.35	-0.23	-0.30	-0.32	-0.20	-0.46	-0.39	-0.14	-0.34	-0.31	0.04	0.58	-0.78	Stable	3.00
FAM167A	0.42	0.63	0.50	0.63	0.45	0.39	0.54	0.35	0.25	0.89	0.51	0.48	0.59	0.69	0.86	0.39	0.90	0.98	-0.03	Stable	14.00
FAAP20	-0.17	-0.69	-0.72	0.13	-0.56	0.02	-0.75	-0.34	-0.43	-0.04	0.26	-0.75	-0.09	-0.31	-0.75	-0.50	0.05	2.70	1.43	Stable	6.00
USP30	-0.18	-0.16	0.33	0.26	0.27	0.13	-0.24	-0.17	-0.07	-0.16	0.27	0.03	0.04	-0.37	-0.30	0.13	0.01	-1.23	0.30	Stable	14.00
ENKUR	-0.23	-0.21	-0.22	-0.46	-0.23	-0.46	-0.20	-0.46	-0.46	0.00	-0.45	-0.23	-0.45	-0.23	-0.46	-0.20	0.99	1.00	0.00	Stable	10.00
SLC25A34	-0.31	-0.13	-0.16	-0.42	-0.22	-0.42	-0.41	-0.19	-0.31	-0.32	-0.42	-0.43	-0.18	-0.06	-0.31	-0.28	0.08	0.69	-0.53	Stable	6.00
ZNF664	0.27	0.21	-0.15	-0.14	0.64	0.73	0.34	-0.04	0.15	0.14	0.50	0.20	0.61	0.47	-0.15	0.73	0.06	0.32	-1.64	Stable	14.00
ENSSSCG00000032969	-0.22	-0.37	-0.43	-0.28	-0.40	-0.24	-0.50	0.15	-0.37	-0.26	-0.50	-0.50	0.07	-0.22	-0.50	-0.38	0.95	0.98	-0.03	Stable	6.00
ENSSSCG00000032978	-0.21	-0.02	-0.21	-0.01	-0.02	0.02	-0.29	-0.46	-0.46	-0.26	-0.06	-0.29	-0.08	0.01	-0.29	-0.17	0.10	2.20	1.14	Stable	6.00
GOT2	0.07	0.18	0.09	0.18	0.18	0.18	0.02	0.09	0.04	0.15	0.13	0.09	0.07	0.10	0.13	0.21	0.03	0.61	-0.72	Stable	6.00
FXN	-0.17	0.18	-0.19	-0.22	-0.25	0.11	-0.50	-0.02	-0.20	0.16	-0.22	-0.40	-0.03	-0.06	0.08	0.09	0.89	1.16	0.21	Stable	1.00

CIAO2B	0.16	0.08	0.00	0.00	0.08	0.08	0.08	0.00	0.00	0.16	0.00	0.08	0.00	0.08	0.08	0.08	0.99	1.00	0.01	Stable	6.00
ZBTB14	-0.34	-0.06	-0.15	-0.15	-0.25	-0.17	-0.34	-0.34	-0.17	-0.31	-0.27	-0.34	-0.15	-0.23	-0.34	-0.25	0.85	1.04	0.05	Stable	6.00
HGS	0.00	0.00	-0.04	0.00	0.00	0.00	0.00	-0.58	-0.04	0.00	-0.33	0.00	0.00	-0.29	0.00	0.00	0.38	2.88	1.53	Stable	12.00
KIN	-0.54	-0.21	0.22	-0.21	-0.98	-0.31	-0.14	-0.19	-0.62	-0.53	-0.21	-0.84	-0.21	-0.53	-0.06	-0.89	0.11	0.50	-1.01	Stable	10.00
DYNLT1	0.55	0.16	0.14	0.57	0.55	0.80	0.21	0.28	0.48	0.09	0.03	0.44	0.76	0.14	0.49	0.61	0.17	0.64	-0.65	Stable	1.00
PALM2AKAP2	-0.54	-0.85	-0.58	-0.55	-0.19	0.00	-0.14	-0.65	-0.46	-0.21	-0.51	-0.33	-0.51	-0.54	-0.61	-0.41	0.05	1.62	0.69	Stable	1.00
YTHDF3	0.50	0.48	0.51	0.44	0.50	0.23	0.50	0.51	0.51	0.30	0.32	0.50	0.50	0.51	0.50	0.50	0.05	1.22	0.29	Stable	4.00
TPO	0.41	0.17	0.06	0.15	0.10	0.10	0.56	0.06	0.21	0.17	0.37	0.25	0.99	0.58	0.50	0.00	0.71	1.19	0.26	Stable	3.00
TAFA4	0.20	0.15	0.04	0.35	0.02	0.09	0.10	0.13	0.22	0.16	0.04	0.19	0.26	0.08	0.08	0.04	0.70	0.87	-0.20	Stable	13.00
LYRM7	0.16	0.26	0.08	0.12	0.45	0.22	0.06	0.34	0.05	-0.02	0.23	0.50	0.58	0.15	0.20	0.13	0.20	0.59	-0.76	Stable	2.00
ENSSSCG00000033232	0.08	0.22	0.12	0.00	-0.13	0.00	0.19	0.47	0.46	0.23	-0.22	0.19	-0.26	0.12	0.19	0.13	0.01	-32.32	5.01	Up	6.00
LZIC	-0.22	-0.47	-0.02	0.21	0.48	0.26	-0.31	-0.32	-0.82	-0.45	0.22	-0.31	0.11	0.09	-0.29	-0.05	0.03	-5.07	2.34	Up	6.00
ARMH1	-0.37	0.02	-0.60	0.10	-0.51	-0.04	-0.93	-0.10	-0.10	-0.02	-0.10	-0.80	-0.28	-0.30	-0.25	-0.51	0.72	1.21	0.28	Stable	6.00
ATP6V1D	-0.07	0.17	0.44	-0.24	0.22	-0.24	0.67	-0.30	-0.30	0.67	0.51	-0.07	-0.24	-0.07	0.17	0.06	0.99	1.03	0.04	Stable	7.00
ENSSSCG00000033310	0.09	0.26	0.54	0.47	0.30	-0.05	0.21	0.35	0.23	0.59	0.48	0.52	0.49	0.35	0.15	0.48	0.14	0.66	-0.59	Stable	5.00
RPL37	0.51	0.49	0.59	0.26	0.29	0.48	0.53	0.36	0.14	0.49	0.17	0.60	0.19	0.60	0.57	0.38	0.16	1.32	0.40	Stable	16.00
AKIP1	-0.57	0.14	0.27	0.11	-0.25	-0.12	-0.57	-0.57	-0.15	-0.47	-0.18	-0.57	-0.25	0.17	0.17	-0.25	0.50	0.57	-0.81	Stable	9.00
LGI3	0.01	0.03	0.32	0.05	0.16	0.07	-0.01	0.42	0.05	0.06	0.05	0.00	0.11	0.06	0.05	0.11	0.51	1.55	0.63	Stable	14.00
CUEDC2	0.44	0.18	-0.01	0.07	0.02	0.24	0.18	0.42	0.42	0.36	0.12	0.33	0.24	0.23	0.30	-0.01	0.21	1.58	0.66	Stable	14.00
ENSSSCG00000033368	0.14	0.07	0.06	-0.02	0.41	0.28	0.31	0.57	0.07	-0.08	-0.10	-0.16	-0.14	0.19	-0.16	-0.17	0.18	38.57	5.27	Stable	5.00
MRPL20	-0.21	-0.52	-0.35	-0.25	-0.41	-0.22	-0.10	-0.45	-0.48	-0.51	-0.28	-0.10	-0.54	-0.55	-0.10	-0.32	0.85	1.05	0.07	Stable	6.00
SARAF	-0.30	-0.63	0.00	-0.62	-0.32	-0.62	0.00	-0.62	-0.62	-0.30	-0.31	-0.30	-0.31	0.00	-0.32	-0.63	0.35	0.73	-0.45	Stable	15.00
ENSSSCG00000033421	-0.18	0.07	-0.21	0.22	-0.01	0.10	-0.15	-0.22	0.04	0.24	0.14	0.05	-0.02	-0.18	-0.14	-0.34	0.05	-2.60	1.38	Up	12.00
SNRPD2	-0.38	0.00	0.01	-0.12	0.06	-0.30	0.01	0.00	0.00	-0.32	0.00	0.01	-0.32	-0.41	0.01	0.06	0.82	0.82	-0.28	Stable	6.00
SAMD11	-0.49	-0.67	-0.68	-0.28	-0.43	-0.31	-0.59	-0.32	-0.47	-0.27	-0.18	-0.64	-0.59	-0.64	-0.64	-0.67	0.10	1.34	0.42	Stable	6.00
BAG2	-0.06	0.02	0.01	0.52	0.28	0.03	0.05	0.02	-0.01	0.02	0.27	0.00	0.38	-0.06	-0.06	0.40	0.01	-0.04	-4.49	Down	7.00
UBXN11	-0.21	-0.16	-0.22	-0.01	-0.14	-0.01	-0.29	-0.16	-0.21	-0.16	-0.04	-0.29	-0.01	-0.09	-0.14	-0.14	0.06	1.82	0.86	Stable	6.00
DUSP23	-0.02	-0.83	-0.34	-0.05	-0.24	-0.41	0.11	-0.98	-0.21	-0.24	-0.24	-0.44	-0.24	-0.49	-0.40	-0.24	0.37	1.49	0.58	Stable	4.00
DBNDD2	0.15	0.16	0.26	0.00	0.10	0.13	-0.04	0.21	-0.04	0.14	0.21	-0.07	0.08	-0.02	0.03	0.11	0.99	1.01	0.01	Stable	17.00
ZNF793	0.34	0.03	0.23	-0.23	0.13	0.06	0.38	0.00	-0.05	0.21	-0.19	0.38	0.19	0.36	0.38	0.27	0.31	2.05	1.03	Stable	6.00
ENSSSCG00000033620	0.02	-0.06	0.04	-0.01	0.04	0.02	0.02	-0.63	-0.01	0.04	0.00	0.04	-0.01	-0.19	0.02	0.04	0.18	-4.63	2.21	Stable	12.00
DERL1	0.15	0.26	0.29	0.46	0.30	0.47	0.47	0.13	0.29	0.23	0.24	0.47	0.46	0.28	0.02	0.13	0.14	0.69	-0.54	Stable	4.00
B3GALNT2	1.10	0.86	0.43	0.49	0.52	0.33	0.75	0.98	0.40	0.44	0.39	0.95	0.44	0.88	0.33	0.41	0.10	1.44	0.53	Stable	14.00
ENSSSCG00000033697	-0.09	-0.39	-0.17	-0.06	-0.17	-0.17	-0.17	-0.09	-0.51	-0.17	-0.17	-0.37	-0.17	-0.29	-0.71	-0.17	0.19	1.65	0.72	Stable	3.00
FAM111A	-0.19	-0.24	-0.28	-0.15	-0.21	-0.22	-0.19	-0.17	-0.15	-0.24	-0.27	-0.31	-0.23	-0.20	-0.29	-0.31	0.31	0.88	-0.18	Stable	2.00
GPX1	1.02	0.24	0.32	0.73	1.06	0.86	1.02	-0.06	-0.25	0.14	0.08	0.83	1.02	1.02	1.02	1.16	0.42	0.73	-0.45	Stable	13.00
FABP9	0.29	0.10	0.10	0.08	0.19	0.12	0.02	0.24	0.17	0.07	0.03	0.07	0.03	0.10	-0.01	0.03	0.23	1.68	0.75	Stable	4.00
ANKRD10	-0.21	0.00	0.00	0.00	0.00	0.00	0.00	0.00	0.00	0.00	0.00	0.00	0.00	0.00	0.00	0.00	0.35	#NAME?	Inf	Stable	11.00
NIP7	-0.22	-0.19	-0.19	-0.22	-0.22	-0.22	-0.21	-0.21	-0.22	-0.22	0.00	-0.22	-0.22	-0.21	-0.22	-0.22	0.57	1.09	0.12	Stable	6.00
ENSSSCG00000033750	-0.48	0.13	0.41	0.07	-0.32	-0.36	-0.23	0.11	-0.18	0.24	-0.35	0.04	0.21	-0.17	-0.25	-0.03	0.89	1.28	0.36	Stable	17.00
THRSP	0.40	-0.02	0.23	-0.10	0.22	-0.06	-0.03	0.07	0.07	0.10	-0.07	-0.02	0.10	0.46	0.18	0.04	0.08	7.08	2.82	Stable	9.00
XPNPEP3	-0.29	-0.05	-0.32	-0.11	-0.57	0.01	-0.08	-0.17	-0.16	0.16	-0.44	-0.08	-0.43	-0.08	0.00	-0.35	0.41	0.63	-0.67	Stable	5.00
TRMT44	-1.73	-0.91	-0.39	-0.60	-1.12	-1.00	-1.91	0.26	-1.07	-0.49	-1.05	-0.17	-0.81	-0.28	-0.46	-1.31	0.98	0.99	-0.01	Stable	8.00
SMIM2	0.38	-0.16	0.00	0.58	0.43	0.54	0.14	0.17	0.35	-0.03	0.94	-0.03	0.43	0.27	0.28	0.64	0.08	0.41	-1.29	Stable	11.00
IFN-OMEGA-2	0.00	-0.08	0.00	0.03	0.92	0.60	0.00	1.07	0.00	0.52	0.14	0.00	0.07	1.28	0.48	-0.14	0.74	1.29	0.37	Stable	1.00

C1RL	0.54	0.66	0.70	-0.02	-0.02	0.06	0.58	0.21	0.02	-0.02	-0.06	0.13	-0.02	0.29	0.17	0.18	0.00	12.48	3.64	Up	5.00
ENSSSCG00000033918	-0.28	-0.03	-0.23	-0.41	-0.26	-0.26	-0.20	0.01	-0.03	-0.37	-0.15	0.05	-0.27	-0.20	-0.02	0.05	0.30	0.61	-0.72	Stable	12.00
DYNC2H1	0.07	0.07	0.00	0.07	0.00	0.00	0.07	0.00	0.00	0.00	0.00	0.07	0.10	0.00	0.00	0.03	0.72	0.79	-0.35	Stable	9.00
CYB5B	0.18	-0.20	-0.18	-0.13	-0.06	-0.13	0.40	-0.13	0.25	-0.24	0.04	0.07	-0.15	-0.12	0.17	-0.16	0.15	-0.47	-1.09	Stable	6.00
RGS18	-0.07	-0.04	0.04	0.04	-0.09	0.02	-0.09	-0.10	0.23	-0.06	0.02	0.00	-0.01	-0.09	0.14	0.09	0.97	2.04	1.03	Stable	10.00
ENSSSCG00000033954	0.45	-0.26	-0.11	-0.61	-0.26	-0.24	-0.75	0.04	-0.06	-0.14	0.08	-0.02	-0.66	0.41	0.36	-0.09	0.17	-0.04	-4.66	Stable	7.00
PLCXD3	0.06	-0.13	0.18	0.33	-0.28	0.08	0.06	0.47	0.22	-0.11	-0.59	0.16	0.61	0.16	-0.25	-0.22	0.52	-19.57	4.29	Stable	16.00
TMEM69	-0.51	-0.30	-0.21	0.03	-0.16	-0.51	-0.34	-0.10	-0.06	-0.66	-0.13	-0.42	-0.23	-0.40	-0.13	-0.21	0.77	0.90	-0.15	Stable	6.00
ZCRB1	0.28	0.28	0.28	0.28	0.28	0.28	0.28	0.28	0.28	0.28	0.14	0.28	0.28	0.14	0.00	0.14	0.69	0.93	-0.11	Stable	5.00
TNFSF15	-0.82	-0.89	-0.53	-0.32	-0.01	-0.31	-0.14	-1.15	-1.30	-0.07	0.16	-0.23	-1.05	-0.36	-0.81	-0.22	0.02	2.94	1.56	Up	1.00
ENSSSCG00000034119	0.24	0.06	0.25	0.11	-0.03	-0.10	0.24	0.25	0.25	0.02	0.16	0.24	-0.10	0.12	-0.15	-0.10	0.08	6.12	2.61	Stable	10.00
TMEM128	-0.06	0.30	-0.08	0.21	-0.26	-0.26	0.24	-0.14	-0.26	-0.26	-0.26	-0.26	-0.26	-0.24	0.04	-0.26	0.08	0.12	-3.02	Stable	8.00
TXNDC5	-0.03	-0.02	-0.14	-0.26	0.02	-0.13	-0.12	0.00	-0.02	-0.10	-0.11	-0.23	-0.43	-0.12	0.08	-0.15	0.04	0.27	-1.89	Down	7.00
DTYMK	-0.10	-0.09	0.00	0.27	0.00	0.23	0.00	0.11	0.12	0.33	0.00	0.00	0.16	-0.03	-0.32	0.00	0.04	-0.32	-1.66	Down	15.00
GTF2F2	-0.04	-0.13	0.05	-0.32	-0.45	-0.29	-0.10	-0.12	-0.33	-0.02	-0.18	-0.34	-0.36	-0.31	-0.31	-0.31	0.10	0.58	-0.80	Stable	11.00
WDR64	-0.39	-0.34	-0.50	0.17	-0.34	-0.54	-0.28	-0.30	-0.88	0.26	-0.23	0.10	-0.10	-0.17	-0.06	-0.77	0.26	1.99	0.99	Stable	10.00
AIF1L	0.00	0.00	0.00	0.00	0.00	0.00	0.00	0.00	0.00	-0.01	-0.01	0.00	-0.01	0.00	0.00	0.00	0.04	0.00	#NAME?	Down	1.00
SEC22C	0.14	-0.03	0.30	-0.08	0.14	0.30	0.14	-0.05	0.19	0.33	0.00	-0.09	-0.29	0.13	0.14	0.21	0.56	1.81	0.86	Stable	13.00
ACER2	0.00	0.00	0.00	-0.48	0.00	-0.09	0.00	0.00	0.00	-0.24	-0.48	-0.24	-0.48	0.00	0.00	-0.48	0.00	0.00	#NAME?	Down	1.00
ZAR1L	-0.03	-0.01	-0.03	-0.03	-0.03	0.39	-0.03	0.00	-0.21	0.02	0.30	-0.04	-0.03	0.12	0.32	-0.03	0.52	0.24	-2.06	Stable	11.00
LRMDA	-0.44	-0.72	-0.66	-0.25	-0.34	-0.44	-0.34	-0.43	-0.25	-0.34	-0.25	-0.28	-0.26	-0.44	-0.92	-0.38	0.03	1.65	0.72	Stable	14.00
TP53INP1	0.00	0.00	0.00	0.03	0.03	0.05	-0.11	0.03	-0.02	0.00	0.06	0.03	0.06	0.00	0.18	0.06	0.33	0.22	-2.16	Stable	4.00
CELSR2	-0.14	-0.15	0.61	-0.45	0.65	-0.01	0.36	-0.60	-0.44	-0.60	-0.66	0.89	-1.08	-0.34	-0.22	0.52	0.94	1.26	0.33	Stable	4.00
SPECC1	0.11	0.11	0.21	0.33	0.15	0.13	0.14	0.14	0.05	0.30	0.65	0.14	0.50	0.75	0.48	0.05	0.78	0.89	-0.17	Stable	12.00
ENSSSCG00000034367	0.00	0.01	0.00	0.16	0.08	0.00	0.00	0.01	0.00	0.16	0.08	0.00	0.16	0.00	0.03	0.08	0.01	0.07	-3.86	Down	6.00
ENSSSCG00000034371	0.27	-0.42	0.04	-0.04	0.13	0.18	0.49	-0.23	0.48	0.60	0.22	0.10	-0.51	-0.84	-0.50	-0.17	0.47	-1.42	0.50	Stable	12.00
IFNGR2	-0.01	-0.01	-0.13	0.00	-0.22	-0.25	-0.12	-0.01	-0.18	0.00	0.00	-0.12	0.00	-0.13	-0.13	-0.25	0.75	0.85	-0.23	Stable	13.00
ENSSSCG00000034387	0.66	0.67	1.03	0.65	0.22	0.62	0.84	0.19	-0.05	0.84	0.31	0.36	0.06	0.96	-0.17	0.28	0.62	1.23	0.30	Stable	2.00
MEI1	0.02	0.07	-0.02	0.09	0.29	0.24	-0.11	0.01	-0.07	0.07	0.22	-0.15	0.26	-0.04	-0.13	0.12	0.01	-0.24	-2.07	Down	5.00
MRGPRF	0.06	0.06	0.05	0.05	0.05	0.00	0.00	0.00	0.00	0.06	0.06	0.05	0.00	0.06	0.00	0.05	0.38	0.69	-0.53	Stable	2.00
BAG4	-0.40	0.12	-0.01	-0.24	0.01	-0.18	-0.01	-0.32	-0.83	-0.14	-0.36	-0.41	0.40	-0.01	-0.42	0.31	0.32	3.07	1.62	Stable	15.00
NUDT21	0.23	0.21	0.09	0.00	0.02	0.08	0.12	0.08	0.08	0.23	0.12	0.12	0.00	0.21	0.00	0.06	0.26	1.58	0.66	Stable	6.00
ENSSSCG00000034572	0.28	0.28	0.28	0.25	0.25	0.25	0.28	-0.04	0.27	0.25	0.17	0.27	0.25	0.27	-0.08	0.25	0.39	0.79	-0.34	Stable	6.00
ENSSSCG00000034623	-0.85	-0.78	-0.74	-0.27	-0.90	-0.12	-0.74	-0.84	-0.85	-0.26	0.39	-0.91	0.65	-0.74	-0.80	-0.16	0.02	4.03	2.01	Up	15.00
MRPS15	0.06	-0.07	-0.33	-0.39	-0.39	-0.33	-0.53	-0.39	-0.46	-0.39	-0.39	-0.53	-0.39	0.06	-0.20	-0.39	0.08	0.58	-0.80	Stable	6.00
ENSSSCG00000034664	0.01	0.02	0.03	-0.04	0.02	-0.07	0.00	0.02	-0.02	-0.08	0.02	0.01	-0.04	0.13	0.02	-0.04	0.03	-0.96	-0.06	Stable	7.00
MFAP3	0.10	0.08	0.52	0.35	0.04	0.10	0.31	0.31	0.08	0.15	0.26	0.04	0.19	0.17	0.05	0.04	0.43	1.40	0.49	Stable	16.00
OTULIN	-0.91	-0.67	-0.81	-0.78	-0.17	-0.07	-0.91	-0.80	-0.80	-0.40	-0.34	-0.93	-0.81	-0.61	-0.90	-0.17	0.02	1.75	0.80	Stable	16.00
IQCK	0.29	0.02	0.09	0.01	0.01	0.10	0.18	0.28	0.04	0.09	0.04	0.47	0.18	0.43	0.32	0.18	0.37	1.51	0.60	Stable	3.00
CCL14	0.25	0.07	0.35	0.29	0.70	0.49	0.13	0.33	0.25	0.41	0.28	0.38	0.14	0.34	0.28	0.53	0.05	0.62	-0.69	Stable	12.00
CCDC24	-0.43	-0.18	-0.13	-0.33	-0.17	-0.53	-0.29	-0.08	-0.12	-0.35	-0.08	-0.29	-0.17	-0.19	-0.29	-0.27	0.35	0.78	-0.37	Stable	6.00
ENSSSCG00000034743	-0.50	-0.74	-0.17	-1.02	-0.76	-0.67	-0.58	-0.82	-0.58	-0.84	-1.02	-0.76	-0.84	-0.58	-0.83	-0.75	0.02	0.72	-0.47	Stable	8.00
TAFA5	-0.23	-0.31	-0.31	0.00	-0.09	-0.15	-0.18	-0.31	-0.27	-0.31	0.00	-0.24	0.12	-0.24	-0.31	0.03	0.01	3.32	1.73	Up	5.00
NT5C3B	0.25	0.15	0.15	0.61	0.01	0.55	0.22	0.25	-0.06	0.11	0.72	0.06	-0.08	0.44	-0.11	0.14	0.43	0.61	-0.72	Stable	12.00
U2AF2	-0.10	-0.10	-0.10	-0.05	-0.32	-0.01	-0.10	-0.10	-0.10	-0.10	-0.01	-0.10	-0.42	-0.10	-0.10	-0.10	0.47	0.70	-0.52	Stable	6.00

CHP2	-0.47	-0.25	-0.15	-0.20	-0.42	-0.12	-0.15	-0.47	-0.11	-0.48	-0.48	-0.20	-0.20	-0.20	-0.47	-0.17	0.99	1.00	0.00	Stable	3.00
ENSSSCG00000034843	-0.48	-0.34	-0.64	-1.08	-0.70	-0.72	-0.63	-0.34	-0.73	-0.34	-0.65	-0.66	-1.06	-0.54	-0.72	-0.66	0.10	0.75	-0.41	Stable	5.00
ENSSSCG00000034844	0.15	-0.08	-0.10	0.15	0.15	0.16	0.15	0.15	0.19	0.19	0.15	0.11	0.15	0.18	0.12	0.15	0.20	0.62	-0.69	Stable	14.00
PARD6A	-0.08	-0.03	0.02	-0.02	-0.05	-0.12	-0.04	0.00	0.02	-0.08	-0.01	-0.03	0.00	-0.02	-0.03	-0.15	0.11	0.33	-1.58	Stable	6.00
FANCG	0.00	0.00	0.00	0.00	0.00	0.00	0.00	0.00	0.00	0.00	0.00	0.00	0.00	0.00	0.00	0.00	0.54	0.82	-0.29	Stable	1.00
AMIGO1	0.09	0.09	0.00	0.09	0.42	0.30	0.00	0.19	0.01	0.19	0.01	0.21	0.18	0.09	0.01	0.42	0.02	0.27	-1.90	Down	4.00
HAPLN4	-0.29	-0.28	0.03	-0.28	-0.14	-0.29	-0.28	-0.28	-0.28	-0.28	-0.29	-0.28	-0.28	-0.28	-0.28	-0.28	0.60	0.91	-0.13	Stable	2.00
ENSSSCG00000034891	0.16	0.12	0.04	0.08	0.16	0.11	0.11	0.13	-0.07	0.18	0.13	0.21	0.06	0.14	0.13	0.16	0.21	0.69	-0.54	Stable	12.00
NKAIN3	-0.16	-0.62	-0.29	-0.17	-0.16	-0.03	-0.16	-0.25	-0.19	-0.44	-0.26	-0.16	-0.16	-0.20	-0.16	-0.16	0.39	1.33	0.41	Stable	4.00
GINS1	0.14	0.18	0.41	0.68	0.98	0.33	0.55	0.22	0.30	0.13	1.11	0.63	0.49	0.58	0.09	0.98	0.03	0.47	-1.10	Down	17.00
CLDN19	0.51	0.23	0.26	0.19	0.16	-0.07	0.57	-0.11	-0.03	0.21	-0.11	0.57	0.12	0.23	0.15	0.32	0.65	1.30	0.38	Stable	6.00
CNIH3	-0.35	0.32	-0.33	-0.36	-0.48	-0.44	-0.09	0.42	0.51	-0.37	-0.24	-1.03	-0.27	-0.44	-0.10	-0.26	0.02	0.02	-5.53	Down	10.00
ENSSSCG00000034976	0.20	-0.19	-0.61	-0.95	-0.22	-0.61	0.20	-0.61	-0.32	-0.61	-0.61	-0.61	-0.61	-0.20	-0.61	-0.61	0.03	0.44	-1.18	Down	8.00
IQANK1	0.46	0.12	0.41	0.24	0.14	0.05	0.51	0.64	0.12	0.17	0.25	0.12	0.19	0.52	0.58	0.31	0.01	2.26	1.18	Up	4.00
ENSSSCG00000035000	-0.18	-0.06	-0.53	-0.65	-0.90	-0.72	-0.24	-0.27	-0.10	-0.97	-0.72	-0.05	-0.47	0.18	-0.43	-0.13	0.02	0.36	-1.49	Down	3.00
DEPDC1B	0.00	0.00	0.00	0.00	0.00	0.00	0.00	0.00	0.00	0.00	0.13	0.00	0.00	0.00	0.00	0.00	0.35	0.00	#NAME?	Stable	16.00
GABRD	-0.31	-0.83	-0.47	-0.16	-0.51	-0.16	-0.19	-0.83	-0.87	-0.59	-0.31	-0.19	-0.52	-0.55	-0.19	-0.34	0.15	1.52	0.60	Stable	6.00
ADGRV1	0.44	0.81	0.61	0.08	0.18	0.81	0.08	0.34	0.08	0.44	0.70	0.74	0.66	0.81	0.25	0.11	0.80	0.92	-0.13	Stable	2.00
CGNL1	-0.12	-0.12	-0.12	0.25	-0.12	0.00	-0.12	-0.12	0.00	-0.12	0.06	-0.12	-0.12	-0.12	-0.12	0.19	0.09	-47.89	5.58	Stable	1.00
CHSY3	0.00	0.21	-0.01	-0.35	-0.70	-0.60	-0.14	-0.17	-0.13	-0.15	-0.51	-0.62	-0.75	-0.71	-0.27	-0.12	0.02	0.32	-1.64	Down	2.00
DCST1	0.31	0.26	0.83	0.75	0.64	0.30	0.40	-0.01	0.00	0.64	0.64	0.42	0.64	0.34	0.33	0.64	0.02	0.52	-0.93	Stable	4.00
MTMR6	0.34	0.23	-0.01	0.40	0.34	0.26	0.41	-0.07	0.39	0.03	0.28	0.31	0.27	-0.20	0.15	0.34	0.20	0.55	-0.85	Stable	11.00
RHBG	0.18	0.19	-0.24	-1.13	-0.82	-0.32	0.60	0.77	0.33	-0.82	-0.82	0.25	-0.82	0.02	0.40	-0.82	0.00	-0.42	-1.24	Down	4.00
IDNK	-0.01	-0.28	0.50	-0.07	-0.03	0.10	-0.27	1.16	0.71	0.29	-0.51	-0.28	0.67	-0.28	-0.10	-0.37	0.40	-7.54	2.91	Stable	10.00
SDR42E1	-0.32	-0.29	-0.29	-0.41	-0.47	-0.41	-0.48	-0.26	-0.30	-0.41	-0.29	-0.40	-0.30	-0.28	-0.35	-0.30	0.15	0.86	-0.22	Stable	6.00
MRPS23	-0.23	0.02	-0.50	0.10	0.17	0.25	-0.15	-0.62	-0.22	-0.14	-0.27	-0.19	0.86	-0.25	-0.48	-0.09	0.02	-3.53	1.82	Up	12.00
BTBD8	-0.32	-0.24	-0.53	0.12	0.01	0.10	0.29	-0.01	0.26	0.12	0.16	-0.16	0.12	0.21	0.12	-0.24	0.65	-0.98	-0.02	Stable	4.00
PTGES3L	0.20	-0.22	0.04	0.09	0.35	0.06	0.20	-0.18	-0.17	-0.14	0.37	0.15	0.34	0.36	0.14	0.04	0.29	0.29	-1.79	Stable	12.00
ANP32E	0.02	0.43	0.33	0.53	0.51	0.25	0.16	0.18	0.01	0.67	0.67	0.02	0.64	0.27	0.01	0.64	0.01	0.36	-1.48	Down	4.00
ENSSSCG00000035224	0.60	0.07	-0.15	-0.06	0.02	0.09	0.36	0.28	0.27	0.09	0.09	0.27	-0.07	0.13	0.12	0.29	0.23	2.27	1.19	Stable	12.00
ENSSSCG00000035226	-0.29	-0.02	-0.24	-0.35	-0.27	-0.33	-0.42	0.04	-0.02	-0.11	-0.42	-0.42	-0.19	-0.02	-0.42	-0.48	0.10	0.55	-0.86	Stable	6.00
GPR63	0.00	0.00	0.00	0.03	0.05	0.00	0.00	0.47	0.44	0.03	0.03	0.03	0.00	0.00	0.44	0.05	0.13	6.23	2.64	Stable	1.00
BMF	0.36	0.13	0.18	0.18	0.18	0.00	0.36	0.18	0.36	0.06	0.12	0.18	0.18	0.13	0.06	0.00	0.06	1.95	0.97	Stable	1.00
ENSSSCG00000035337	0.48	0.09	0.35	0.07	0.15	0.37	0.37	0.09	0.09	0.36	0.39	0.15	0.15	0.09	0.09	0.59	0.41	0.74	-0.44	Stable	3.00
C6orf89	-0.08	0.00	0.00	0.00	-0.08	0.00	0.00	0.00	0.00	0.00	0.00	-0.08	0.00	-0.08	0.00	0.00	1.00	1.00	0.00	Stable	7.00
F2R	-0.14	-0.14	-0.07	0.02	-0.14	-0.14	0.10	-0.14	-0.07	-0.04	-0.13	0.01	-0.13	-0.14	-0.14	-0.14	0.89	1.06	0.09	Stable	2.00
MFHAS1	-0.66	0.27	-0.92	-0.11	-0.72	-0.03	-0.95	-0.38	-0.32	-0.66	-0.52	-0.64	-0.52	-0.92	-0.58	-0.32	0.51	1.27	0.34	Stable	15.00
RD3	-0.42	-0.28	-0.27	-0.37	-0.50	-0.13	-0.45	-0.42	-0.36	-0.52	-0.58	-0.42	-0.44	-0.43	-0.48	-0.52	0.45	0.90	-0.15	Stable	9.00
ENSSSCG00000035371	0.17	0.03	-0.02	0.12	0.09	-0.08	0.10	-0.06	0.00	0.08	-0.03	0.16	0.18	0.19	-0.08	0.03	0.58	0.59	-0.75	Stable	17.00
SAAL1	-0.33	-0.17	0.14	-0.24	-0.03	-0.49	-0.34	-0.32	-0.05	-0.19	-0.25	-0.04	-0.32	-0.25	-0.22	-0.24	0.69	0.86	-0.22	Stable	2.00
ENSSSCG00000035404	0.00	0.09	0.13	0.00	0.00	0.00	0.00	0.00	0.00	0.00	0.13	0.00	0.00	0.00	0.00	0.00	0.65	1.70	0.77	Stable	5.00
XXYLT1	-0.61	-0.76	-0.75	-0.42	-0.11	-0.49	-0.78	-1.05	-0.47	-0.71	-0.07	-0.54	-0.35	-0.76	-0.81	-0.78	0.01	1.72	0.78	Stable	13.00
SS18L1	-0.21	0.11	0.08	0.22	-0.14	-0.16	-0.30	0.11	0.44	0.00	-0.13	-0.10	0.08	-0.31	-0.23	-0.22	0.88	0.70	-0.51	Stable	17.00
ENSSSCG00000035460	-0.06	0.41	0.27	0.31	0.21	0.35	0.08	-0.01	-0.12	0.52	0.52	-0.12	0.23	0.10	0.08	0.27	0.06	0.33	-1.60	Stable	17.00
DISP1	-0.06	-0.45	-0.07	-0.11	-0.28	-0.19	-0.29	-0.14	-0.53	-0.28	-0.14	-0.04	-0.38	-0.15	-0.33	-0.42	0.78	1.10	0.13	Stable	10.00

IFTAP	0.04	0.10	0.12	0.00	0.00	0.00	0.00	0.09	0.00	0.00	0.00	0.05	0.00	0.14	0.09	0.02	0.01	8.50	3.09	Up	2.00
ENSSSCG00000035527	-0.31	-0.76	-0.49	-0.20	-0.51	-0.14	-0.26	-0.78	-0.78	-0.43	-0.21	-0.26	-0.53	-0.52	-0.26	-0.38	0.08	1.56	0.64	Stable	6.00
ENSSSCG00000035561	-0.03	-0.02	0.04	-0.09	-0.04	-0.01	-0.11	-0.10	-0.13	-0.15	-0.08	-0.10	-0.06	-0.13	-0.10	-0.11	0.88	0.95	-0.07	Stable	3.00
CCDC134	-0.40	-0.22	-0.12	-0.22	-0.20	-0.02	-0.30	-0.15	0.16	-0.22	-0.19	-0.25	-0.32	-0.27	-0.25	-0.09	0.93	1.03	0.05	Stable	5.00
ENSSSCG00000035585	0.05	0.00	0.02	-0.03	0.04	0.14	0.05	0.05	0.01	-0.02	0.24	0.01	0.07	0.02	-0.01	0.12	0.20	0.34	-1.54	Stable	2.00
ENSSSCG00000035596	0.01	0.05	0.08	0.06	0.00	-0.05	0.05	0.25	0.24	0.06	0.03	0.21	0.00	0.02	-0.02	0.00	0.33	2.19	1.13	Stable	7.00
GSTK1	0.00	0.00	0.00	0.00	0.00	0.00	0.00	0.00	-0.09	-0.21	0.00	0.00	0.00	0.00	0.00	0.00	0.63	0.45	-1.15	Stable	18.00
ENSSSCG00000035617	0.00	0.23	-0.06	-0.02	0.00	-0.14	0.05	0.14	-0.07	0.14	0.03	0.25	-0.10	0.06	-0.09	0.07	0.91	1.26	0.33	Stable	9.00
CCDC115	0.13	0.05	0.41	0.37	0.14	0.29	-0.06	0.28	0.25	0.32	0.39	0.18	0.28	0.17	0.49	0.41	0.28	0.72	-0.47	Stable	15.00
TERB1	0.33	0.30	0.16	-0.23	-0.34	-0.33	0.31	-0.15	0.00	0.33	-0.26	0.24	-0.09	0.36	0.01	0.03	0.05	-2.02	1.02	Up	6.00
ENSSSCG00000035649	-0.10	0.00	-0.15	-0.25	-0.25	-0.49	-0.25	-0.49	-0.49	-0.49	-0.24	-0.49	-0.25	-0.25	-0.15	-0.25	0.21	0.70	-0.52	Stable	2.00
ENSSSCG00000035650	-0.43	-0.90	-0.29	-0.75	-0.30	-0.13	-0.54	-0.87	-0.22	-0.89	-0.35	-0.35	-0.90	-0.90	-0.90	-0.90	0.69	1.11	0.15	Stable	2.00
VPS36	0.05	-0.16	-0.27	-0.15	0.04	-0.37	0.21	-0.22	-0.46	-0.06	-0.31	-0.17	-0.21	-0.19	-0.06	-0.24	0.61	0.75	-0.41	Stable	11.00
C9orf135	-0.18	0.06	-0.21	0.11	-0.21	-0.39	-0.22	-0.05	-0.13	-0.15	0.04	0.16	0.41	-0.09	-0.06	-0.25	0.47	3.12	1.64	Stable	1.00
KCTD8	0.00	0.00	0.00	0.00	0.18	0.00	0.00	0.00	0.00	0.00	0.00	0.00	0.00	0.00	0.00	0.00	0.35	0.00	#NAME?	Stable	8.00
ENSSSCG00000035762	0.55	0.01	0.20	0.15	0.57	0.23	0.31	-0.03	-0.30	0.56	0.18	0.56	-0.30	0.55	-0.26	0.57	0.27	0.41	-1.28	Stable	14.00
ENSSSCG00000035788	0.25	-0.13	0.06	-0.07	0.06	0.20	0.18	-0.09	-0.13	-0.09	0.18	0.18	-0.05	-0.18	0.18	0.28	0.41	0.22	-2.18	Stable	6.00
BTG1	0.11	0.00	0.22	0.00	0.22	0.11	0.22	0.22	0.00	0.22	0.00	0.11	0.00	0.22	0.11	0.00	0.28	1.67	0.74	Stable	5.00
RPL32	0.25	0.34	0.17	0.07	0.25	0.17	0.25	0.25	0.20	0.20	0.15	0.12	0.07	0.25	0.15	0.17	0.02	1.53	0.61	Stable	13.00
CATSPERE	0.30	0.67	0.22	0.15	-0.14	0.39	0.30	0.13	0.76	0.69	-0.08	-0.16	0.25	0.08	-0.20	0.24	0.45	1.71	0.77	Stable	10.00
PLIN2	0.00	0.00	0.00	-0.24	0.00	0.00	0.00	0.00	0.00	-0.24	0.00	0.00	0.00	0.00	0.00	0.00	0.17	0.00	#NAME?	Stable	1.00
OS9	-0.24	-0.73	-0.18	-0.27	-0.21	0.10	-0.04	-0.04	-0.20	-0.07	-0.36	-0.07	-0.27	-0.18	0.09	-0.19	0.81	1.15	0.20	Stable	5.00
PSMB2	-0.29	0.00	-0.25	-0.53	-0.51	-0.25	-0.60	-0.61	-0.60	-0.57	-0.55	-0.60	-0.53	0.00	-0.46	-0.57	0.14	0.68	-0.55	Stable	6.00
ABHD16B	0.00	-0.41	-0.29	-0.29	-0.14	-0.33	-0.13	-0.29	-0.29	-0.22	-0.22	-0.05	-0.29	-0.13	-0.13	-0.14	0.93	0.98	-0.03	Stable	17.00
ENSSSCG00000035929	-0.42	-0.35	0.38	-0.23	-0.40	-0.70	-0.43	0.39	0.29	-0.70	0.56	-0.08	-0.08	-0.52	-0.50	-0.78	0.49	0.49	-1.03	Stable	14.00
C3orf80	0.09	0.00	0.00	0.00	0.00	0.23	0.23	0.00	0.02	0.01	0.01	0.01	0.02	0.00	0.46	0.00	0.35	2.83	1.50	Stable	13.00
DPT	0.31	0.21	0.27	0.06	0.00	0.39	0.33	0.20	0.18	0.27	0.27	0.48	0.23	0.22	0.25	0.00	0.59	1.17	0.23	Stable	4.00
FDX1	-0.22	-0.83	-0.33	-0.67	0.24	-0.48	-0.22	-0.41	-0.78	-0.52	-1.07	-0.23	-0.54	-0.07	-0.72	-0.70	0.78	0.90	-0.15	Stable	9.00
CENPM	0.02	0.34	0.32	0.50	0.21	0.41	-0.06	-0.69	-0.23	0.72	-0.42	0.46	-0.18	0.22	0.54	0.14	0.38	0.24	-2.04	Stable	5.00
ITPA	-0.32	-0.11	-0.47	-0.56	-0.50	-0.20	-0.59	0.18	-0.26	-0.77	-0.76	-0.65	-0.61	-0.33	-0.37	-0.50	0.02	0.50	-1.01	Down	17.00
MFAP4	-0.45	-0.45	-0.15	-0.54	-0.49	-0.58	-0.53	-0.38	-0.34	-0.53	-0.38	-0.52	-0.69	-0.18	-0.43	-0.48	0.02	0.69	-0.53	Stable	12.00
TACR1	-0.18	-0.20	-0.07	-0.15	-0.18	-0.17	-0.18	-0.15	-0.09	-0.17	-0.15	-0.20	-0.15	-0.18	-0.19	-0.17	0.59	0.94	-0.09	Stable	3.00
RPLP0	0.34	-0.11	0.22	0.23	0.17	1.27	0.14	0.03	0.06	-0.07	0.35	0.35	0.63	-0.11	-0.76	1.32	0.02	-0.04	-4.54	Down	14.00
ENSSSCG00000036017	-0.07	0.02	-0.21	-0.40	-0.25	-0.32	-0.17	0.26	0.17	0.18	-0.49	-0.17	0.12	-0.02	-0.17	0.03	0.22	0.16	-2.68	Stable	6.00
ENSSSCG00000036022	0.58	0.97	0.39	0.71	0.46	0.51	0.15	0.46	1.04	0.43	0.33	0.92	0.30	0.38	0.68	0.63	0.74	1.08	0.11	Stable	4.00
RAB4A	-0.10	-0.21	-0.13	-0.05	-0.06	0.01	-0.09	-0.10	-0.02	-0.26	0.04	-0.10	0.04	-0.09	-0.19	-0.08	0.19	1.97	0.98	Stable	14.00
PEG10	-0.43	-0.54	-0.29	-0.38	-0.62	-0.29	-0.41	-0.18	-0.24	-0.51	-0.62	-0.35	-0.42	-0.49	-0.62	-0.62	0.31	0.84	-0.25	Stable	9.00
RRAD	-0.14	-0.07	-0.07	-0.14	-0.07	-0.07	-0.14	-0.14	-0.07	-0.14	-0.14	-0.14	-0.14	0.00	-0.14	-0.07	0.45	0.85	-0.24	Stable	6.00
WASHC3	0.00	-0.09	0.53	-0.23	-0.42	-0.50	-0.10	0.00	-0.17	-0.11	-0.10	-0.19	-0.48	-0.10	0.42	0.17	0.03	-0.27	-1.88	Down	5.00
TUBG1	-0.65	-0.91	-0.82	-0.95	-1.03	-0.77	-0.60	-1.17	-1.38	-0.38	-0.55	-0.60	-0.75	-0.90	-1.08	-0.77	0.10	1.29	0.37	Stable	12.00
GSAP	0.87	0.87	0.84	0.67	0.38	0.22	0.58	0.90	0.84	0.73	0.84	0.76	0.84	0.10	0.21	0.84	0.96	0.99	-0.02	Stable	9.00
ENSSSCG00000036123	0.01	0.03	0.00	0.25	0.13	0.07	0.01	0.03	0.11	0.01	0.20	0.01	0.25	0.00	0.00	0.13	0.02	0.17	-2.52	Down	8.00
RNF114	0.03	-0.18	-0.27	-0.17	-0.26	-0.11	0.26	-0.25	-0.23	-0.19	-0.18	-0.02	-0.07	0.08	0.03	-0.25	0.25	0.42	-1.24	Stable	17.00
ENSSSCG00000036130	-0.03	0.05	0.03	0.03	0.06	0.01	0.06	0.05	-0.01	0.01	0.05	0.01	0.00	-0.03	0.05	-0.01	0.99	0.99	-0.02	Stable	7.00
CCDC167	-0.04	-0.13	-0.09	-0.66	-0.47	-0.81	0.14	-0.05	0.10	-0.12	-0.53	0.06	-0.75	-0.20	-0.43	-0.82	0.01	0.17	-2.55	Down	7.00

C7orf26	-0.14	-0.18	-0.10	-0.32	-0.18	-0.18	-0.23	-0.10	-0.14	-0.22	-0.36	-0.36	-0.36	-0.28	-0.28	-0.14	0.06	0.67	-0.57	Stable	3.00
GJA8	-0.91	-0.59	-0.67	-0.47	-1.14	-1.80	-1.35	-1.09	-1.01	-0.47	-0.47	-1.41	-0.51	-1.21	-1.22	-0.44	0.45	1.20	0.26	Stable	4.00
ENSSSCG00000036247	-0.30	-0.51	-0.58	-0.52	-0.39	-0.48	-0.34	-0.51	-0.39	-0.39	-0.57	-0.41	-0.57	-0.39	-0.34	-0.26	0.56	0.93	-0.10	Stable	3.00
APMAP	-0.09	0.17	0.31	0.32	0.35	0.17	0.35	0.35	0.35	0.31	0.35	0.03	0.09	0.35	0.02	0.35	0.80	0.92	-0.12	Stable	17.00
NUDT19	0.72	0.14	0.18	0.24	0.56	0.15	1.81	0.86	0.89	0.32	0.85	1.81	0.31	-0.52	1.81	1.70	0.98	0.99	-0.01	Stable	6.00
ENSSSCG00000036297	-0.50	0.30	-0.29	1.32	0.38	-0.15	-0.05	0.82	0.96	-0.10	0.77	-0.19	0.32	0.17	-0.40	1.24	0.29	0.28	-1.82	Stable	1.00
SMIM1	0.22	-0.46	-0.42	0.29	0.04	0.32	-0.22	-0.46	-0.46	0.04	0.29	-0.22	0.06	0.03	-0.22	-0.32	0.03	-3.91	1.97	Up	6.00
FAM126B	0.00	0.00	0.00	-0.37	-0.37	-0.37	-0.29	0.00	-0.13	-0.29	-0.29	-0.37	-0.37	0.00	-0.37	-0.37	0.00	0.28	-1.83	Down	15.00
SLC43A1	-1.35	-0.86	-1.38	-0.75	-0.49	-0.71	-1.35	-1.35	-1.35	0.04	-0.80	-1.18	-0.44	-0.72	-0.23	-0.77	0.05	1.68	0.75	Stable	2.00
ATPSCKMT	-0.13	-0.14	-0.23	-0.05	0.24	0.42	-0.21	-0.16	-0.45	0.01	0.12	0.05	0.19	-0.41	-0.67	-0.07	0.00	-2.63	1.40	Up	16.00
HECW1	-0.06	-0.25	-0.66	-0.16	-0.06	-0.19	-0.04	-0.33	-0.31	-0.32	-0.08	-0.33	0.10	-0.33	-0.59	-0.06	0.07	2.35	1.23	Stable	18.00
PALD1	0.00	0.15	0.32	-0.23	0.02	0.04	-0.03	0.15	0.03	-0.14	0.05	-0.03	-0.02	0.12	0.02	0.03	0.03	-2.77	1.47	Up	14.00
METTL21A	-0.26	0.28	-0.22	-0.10	0.02	-0.72	0.07	0.25	0.34	0.49	-0.10	0.00	0.05	-0.72	-0.16	-0.72	0.67	0.38	-1.41	Stable	15.00
ENSSSCG00000036469	-0.89	-0.80	-0.85	-0.56	-0.59	-0.75	-0.82	-0.73	-0.34	-0.66	-0.77	-0.89	-0.77	-0.94	-0.86	-0.77	0.46	1.08	0.11	Stable	4.00
GJB6	0.17	0.09	-0.05	0.05	0.29	-0.40	0.36	-0.09	1.33	0.51	0.20	0.55	0.72	0.07	0.46	-0.02	0.79	1.24	0.30	Stable	11.00
HS3ST2	0.20	-0.23	0.02	-0.27	-0.11	-0.08	-0.23	0.18	-0.32	-0.10	-0.20	0.30	-0.23	0.32	0.24	-0.23	0.23	-0.19	-2.43	Stable	3.00
ENSSSCG00000036483	-0.31	-0.75	-1.07	-0.61	-0.74	-1.32	-0.17	-1.02	-0.94	-0.52	-0.56	-0.26	-1.26	-0.84	-1.13	-1.06	0.94	0.98	-0.02	Stable	14.00
ENSSSCG00000036516	0.18	0.08	0.09	0.12	0.05	0.03	-0.01	0.08	0.01	0.30	0.27	0.23	0.10	0.14	0.14	0.05	0.25	0.62	-0.70	Stable	2.00
DNASE1	-0.83	-0.69	-0.63	-0.74	-0.63	-0.63	-0.65	-0.30	-0.78	-0.63	-0.65	-0.78	-0.63	-0.33	-0.93	-0.65	0.77	0.96	-0.05	Stable	3.00
WDR53	-0.12	-0.04	-0.06	-0.04	-0.16	-0.05	-0.15	-0.37	-0.18	-0.21	-0.12	-0.06	-0.04	-0.04	-0.11	-0.01	0.31	1.57	0.65	Stable	13.00
SAMD12	0.42	0.29	0.64	0.35	0.00	0.29	0.00	0.71	0.71	0.35	0.00	0.00	0.00	0.00	0.71	0.71	0.14	2.04	1.03	Stable	4.00
ENSSSCG00000036556	0.26	0.32	0.26	0.18	0.47	0.24	0.03	0.83	0.51	0.66	0.09	0.01	0.21	0.12	0.26	0.10	0.49	1.33	0.42	Stable	13.00
BCO1	-0.07	-0.33	-0.33	0.59	0.63	0.59	0.69	0.41	-0.23	0.59	0.72	0.24	0.50	-0.18	-0.53	0.50	0.00	-0.13	-2.92	Down	6.00
SRSF2	0.07	0.14	0.07	0.00	0.00	0.00	0.14	0.07	0.00	0.15	0.00	0.00	0.00	0.14	0.00	0.00	0.05	4.28	2.10	Up	12.00
ENSSSCG00000036652	0.00	-0.20	-0.21	-0.20	0.00	-0.21	-0.21	-0.21	0.00	0.00	-0.41	0.00	0.00	0.00	-0.21	-0.15	0.92	1.05	0.08	Stable	7.00
PPT1	0.24	0.19	0.33	0.19	0.30	0.02	0.30	0.35	0.35	0.30	0.33	0.30	0.19	0.28	0.35	0.30	0.19	1.24	0.31	Stable	6.00
ZNF658	-0.32	-0.63	-0.51	-0.35	-0.20	-0.22	-0.30	-0.57	-0.57	-0.22	-0.57	-0.32	-0.56	-0.47	-0.21	-0.47	0.29	1.23	0.30	Stable	10.00
ENSSSCG00000036721	0.00	-0.06	-0.06	-0.12	-0.12	-0.12	-0.03	0.00	-0.06	-0.07	0.00	-0.12	-0.12	-0.06	-0.12	0.00	0.17	0.58	-0.78	Stable	5.00
CRYAB	0.07	0.40	0.18	0.37	0.50	0.68	0.13	0.17	0.17	0.17	0.29	0.66	0.32	0.69	0.53	0.20	0.33	0.73	-0.45	Stable	9.00
ENSSSCG00000036736	0.00	-0.11	0.40	-0.11	-0.11	-0.08	0.00	-0.11	-0.11	-0.22	-0.22	-0.11	-0.11	0.00	-0.11	-0.11	0.08	0.04	-4.59	Stable	8.00
PITX1	-0.15	-0.15	-0.15	-0.05	-0.15	-0.15	-0.15	-0.05	-0.15	-0.15	-0.15	-0.15	-0.15	-0.08	-0.15	-0.15	0.64	0.93	-0.10	Stable	2.00
RASL10B	0.36	0.09	0.51	0.59	1.53	0.85	0.94	0.16	0.59	1.66	0.52	1.20	0.43	-0.28	-0.04	0.40	0.02	0.32	-1.62	Down	12.00
SLC48A1	0.66	0.42	1.02	-0.54	-0.71	0.11	0.94	-0.34	-0.35	0.10	-0.06	0.55	-0.49	0.50	0.10	-0.13	0.05	-2.49	1.32	Up	5.00
MTMR7	0.40	0.31	0.64	0.04	0.63	0.05	0.56	0.57	0.65	0.56	0.14	0.17	0.14	0.37	0.34	0.52	0.07	1.71	0.77	Stable	17.00
VWA7	0.42	0.41	0.52	0.34	0.16	0.46	0.64	0.24	0.59	0.32	0.17	0.49	0.30	0.45	0.15	0.23	0.12	1.39	0.47	Stable	7.00
C6orf132	0.91	0.91	0.69	0.90	0.83	0.64	0.21	0.78	0.76	0.49	0.56	0.77	0.64	0.97	0.65	0.62	0.60	1.08	0.11	Stable	7.00
AVPI1	0.00	-0.02	-0.13	-0.13	-0.08	-0.22	-0.02	-0.12	0.00	-0.06	-0.04	-0.06	-0.12	-0.11	0.00	-0.04	0.18	0.55	-0.87	Stable	14.00
STK40	0.28	0.21	-0.08	-0.06	-0.06	0.03	-0.20	-0.13	-0.13	-0.06	0.06	-0.20	-0.06	0.49	0.05	-0.13	0.22	-1.03	0.04	Stable	6.00
C11orf71	-0.08	0.12	0.19	-0.08	0.39	0.22	0.00	-0.08	0.00	-0.08	0.49	-0.15	-0.08	-0.15	0.26	0.38	0.34	0.24	-2.07	Stable	9.00
ENSSSCG00000036877	0.00	-0.19	-0.40	-0.40	-0.19	-0.38	0.00	-0.19	-0.19	-0.38	-0.38	-0.14	-0.41	-0.05	-0.19	-0.38	0.01	0.45	-1.14	Down	15.00
FABP3	0.00	0.09	0.09	0.19	0.10	0.19	0.00	0.10	0.00	0.07	0.09	0.00	0.19	0.06	0.00	0.10	0.03	0.37	-1.45	Down	6.00
CLPB	0.18	0.04	-0.03	-0.18	-0.05	0.24	-0.01	0.28	0.61	0.30	-0.16	-0.01	0.56	0.26	-0.10	-0.03	0.57	1.85	0.89	Stable	9.00
C17orf107	1.42	0.42	1.08	0.93	-0.04	0.19	0.59	0.61	0.66	1.10	0.70	1.10	0.32	0.27	1.00	0.32	0.40	1.31	0.39	Stable	12.00
WNT6	0.01	-0.03	-0.17	0.03	-0.03	-0.05	-0.06	0.00	-0.23	-0.09	-0.13	-0.11	0.00	0.19	-0.19	0.30	0.51	4.99	2.32	Stable	15.00
AKR1E2	0.86	0.78	0.52	0.61	-0.04	0.62	-0.04	1.24	1.05	0.23	0.65	-0.23	0.40	0.32	1.88	0.14	0.05	2.78	1.48	Up	10.00

MORN4	-0.27	-0.89	-1.21	-0.16	-1.33	-0.06	0.07	0.53	-0.27	-1.03	-1.23	-0.29	-1.10	0.43	0.66	-1.30	0.04	0.15	-2.77	Down	14.00
PCDHB7	-0.90	-0.99	-0.25	-0.94	0.13	0.11	-1.34	-1.08	0.31	-0.28	-1.02	-0.11	-0.61	-0.38	0.82	-0.11	0.69	1.36	0.44	Stable	2.00
SUGP1	-0.02	0.39	0.24	0.60	0.24	-0.55	0.18	0.23	0.38	0.23	0.02	0.17	0.30	0.31	0.32	0.32	0.50	1.53	0.61	Stable	2.00
GALR1	0.67	0.42	0.67	0.92	0.36	0.50	0.67	0.37	0.37	-0.08	0.88	0.97	0.59	0.67	-0.25	0.50	0.44	0.77	-0.37	Stable	1.00
UVSSA	-0.43	-0.36	-0.34	-0.41	-0.49	-0.44	-0.50	-0.20	-0.17	-0.12	-0.44	-0.44	-0.43	-0.26	-0.31	-0.44	0.17	0.79	-0.33	Stable	8.00
AK6	0.08	0.41	0.15	0.29	0.21	0.16	0.15	0.29	0.29	0.41	0.08	0.16	0.28	0.15	0.16	0.08	0.98	1.01	0.01	Stable	16.00
RRP1	-0.61	-0.45	-0.45	-0.57	-0.62	-0.42	-0.61	-0.55	-0.36	-0.50	-0.74	-0.57	-0.62	-0.57	-0.40	-0.69	0.08	0.84	-0.25	Stable	13.00
GRID2IP	0.15	0.33	0.14	0.28	0.36	0.13	0.14	0.22	0.09	0.26	0.76	0.56	0.73	-0.02	0.12	0.22	0.02	0.36	-1.49	Down	3.00
CDKN2AIPNL	0.12	0.35	0.22	0.42	0.15	0.11	0.18	0.00	0.14	0.05	0.14	0.13	0.09	0.04	0.13	0.13	0.93	0.97	-0.05	Stable	2.00
CLTA	0.00	0.00	0.00	0.35	0.31	0.22	0.00	0.00	0.00	0.07	0.30	0.08	0.23	0.00	0.00	0.39	0.00	0.00	#NAME?	Down	1.00
MTURN	-0.44	-0.22	-0.56	-0.37	-0.47	-0.97	-0.44	-0.22	-0.91	-0.55	-0.46	-0.45	-0.55	-0.18	-0.36	-0.47	0.29	0.78	-0.36	Stable	18.00
POMP	0.66	0.40	-0.02	0.25	0.62	0.39	0.59	-0.07	0.21	-0.02	-0.24	0.02	0.18	-0.04	-0.10	0.76	0.80	0.83	-0.27	Stable	11.00
TLCD3B	-0.14	0.04	-0.02	-0.27	-0.03	-0.11	-0.15	-0.22	-0.25	-0.06	-0.04	0.03	0.00	-0.23	0.11	-0.29	0.84	1.14	0.18	Stable	3.00
MPV17	-0.13	0.38	-0.69	-0.78	-0.97	-1.18	-1.45	-1.23	-0.03	-0.58	-1.25	-1.74	-0.96	-0.89	0.04	-1.04	0.06	0.47	-1.09	Stable	3.00
TLNRD1	0.30	0.01	0.32	0.04	0.08	0.08	0.07	0.05	0.05	0.09	0.06	0.06	0.07	0.30	0.06	0.07	0.15	2.09	1.07	Stable	7.00
COMTD1	-0.63	-0.74	-0.70	-0.64	-0.05	-0.24	-0.32	-0.63	-0.67	-0.64	-0.23	-0.37	-0.70	-0.63	-0.74	-0.01	0.03	1.76	0.82	Stable	14.00
ENSSSCG00000037257	0.14	0.56	0.21	-0.06	-0.03	-0.06	0.41	1.06	1.19	0.53	0.69	0.41	-0.11	-0.12	0.41	0.32	0.18	2.30	1.20	Stable	6.00
MTIF3	0.17	0.26	0.47	0.00	-0.06	0.18	0.15	0.20	0.53	0.46	0.35	0.25	0.01	0.69	0.26	-0.05	0.07	2.39	1.26	Stable	11.00
TMCO1	0.00	-0.16	-0.16	0.00	0.00	-0.11	0.00	-0.33	-0.16	0.00	0.00	-0.16	0.00	0.00	0.00	0.00	0.19	2.98	1.58	Stable	4.00
GID4	-0.37	-0.56	-0.67	-0.33	-0.26	-0.27	-0.52	-0.47	-0.49	-0.22	-0.29	-0.31	-0.27	-0.25	-0.03	-0.34	0.10	1.47	0.55	Stable	12.00
HPS5	-0.16	-0.06	-0.12	-0.36	0.20	-0.10	-0.05	-0.39	-0.17	-0.01	-0.13	-0.19	-0.09	-0.31	-0.16	-0.10	0.27	1.82	0.87	Stable	2.00
MTG2	0.27	0.52	0.72	0.28	0.53	0.28	0.43	0.64	0.83	0.19	0.33	0.59	0.43	0.41	0.20	0.31	0.17	1.36	0.45	Stable	17.00
GET1	-0.29	0.16	0.16	-0.09	0.00	-0.16	-0.36	-0.35	-0.21	0.16	0.00	0.00	0.00	0.00	0.00	0.00	0.26	9.64	3.27	Stable	13.00
TCTA	0.09	0.12	0.07	0.20	0.04	0.08	0.09	0.07	0.00	0.08	0.08	0.13	0.19	0.09	0.09	0.25	0.08	0.58	-0.78	Stable	13.00
SPTBN4	0.20	0.09	0.10	0.06	0.23	0.03	0.20	0.26	0.19	0.23	0.03	0.20	0.20	0.14	0.20	0.20	0.52	1.17	0.23	Stable	6.00
BTBD9	0.23	-0.41	-0.40	-0.30	-0.40	0.35	-0.47	-0.12	-0.29	0.56	-0.08	-0.32	-0.20	0.20	-0.22	-0.20	0.49	2.48	1.31	Stable	7.00
LY6D	0.30	0.64	0.46	0.91	0.39	0.71	0.30	-0.10	0.38	0.48	0.08	0.61	0.70	-0.18	0.69	0.35	0.16	0.59	-0.76	Stable	4.00
SLC2A4RG	0.07	0.18	0.25	0.02	0.12	0.08	0.07	0.19	0.45	0.10	0.10	0.12	0.04	0.07	0.07	0.02	0.09	2.28	1.19	Stable	17.00
ENSSSCG00000037566	0.17	-0.18	-0.30	-0.22	-0.21	0.21	0.03	-0.05	0.03	-0.20	-0.03	0.03	-0.22	-0.39	0.10	0.02	0.96	0.95	-0.08	Stable	6.00
FKBP14	-0.02	0.03	-0.13	-0.03	-0.03	-0.03	-0.03	0.04	-0.19	-0.03	-0.03	-0.03	-0.03	0.03	-0.01	-0.03	0.90	1.12	0.16	Stable	18.00
F2RL2	0.41	0.29	0.14	0.19	0.13	0.29	0.36	0.36	0.15	0.13	0.44	0.21	0.44	0.30	0.37	0.43	0.80	1.05	0.08	Stable	2.00
AP3S1	0.29	0.24	0.08	0.21	0.23	0.24	-0.06	0.24	0.14	0.17	0.28	0.23	0.32	0.24	0.20	0.10	0.29	0.77	-0.38	Stable	2.00
SNX10	0.31	0.22	0.47	-0.03	0.09	-0.07	0.31	0.08	0.06	0.47	0.31	0.36	0.31	0.12	0.36	0.09	0.57	1.26	0.34	Stable	18.00
C1orf216	0.19	0.12	0.14	-0.22	0.09	-0.06	0.34	0.23	0.03	0.09	-0.77	0.34	-0.22	0.22	0.45	-0.12	0.03	-2.03	1.02	Up	6.00
ENSSSCG00000037609	0.01	-0.22	-0.09	-0.62	-0.37	-0.18	-0.18	0.10	0.41	-0.10	-0.39	-0.20	-0.60	-0.11	-0.06	-0.05	0.01	0.06	-4.08	Down	5.00
EEF1AKMT3	-0.02	-0.27	0.00	0.00	-0.08	-0.08	-0.06	0.00	-0.27	0.00	-0.02	0.00	0.00	0.00	-0.27	0.00	0.10	5.15	2.37	Stable	5.00
TIMMDC1	0.00	0.00	0.00	0.00	-0.27	0.00	0.00	0.00	0.00	0.00	-0.24	0.00	0.00	-0.27	0.00	0.00	0.58	0.53	-0.92	Stable	13.00
PRKAR2B	0.30	0.30	0.25	0.26	0.13	0.10	0.13	0.13	0.13	0.27	0.17	0.30	0.13	0.16	0.28	0.22	0.74	1.06	0.09	Stable	9.00
ZNF473	0.15	0.21	0.58	-0.03	-0.50	-0.03	0.55	0.01	0.15	-0.07	0.26	0.55	-0.53	0.03	0.51	0.24	0.09	-22.03	4.46	Stable	6.00
ANAPC16	-0.28	0.21	0.05	0.60	0.78	0.07	0.03	-0.06	0.41	0.48	0.20	0.19	0.59	-0.59	0.80	0.65	0.05	0.16	-2.64	Stable	14.00
NSMCE2	-0.42	-0.27	-0.03	-0.17	-0.43	-0.28	-0.28	0.04	-0.41	-0.09	-0.28	-0.28	-0.28	-0.31	-0.35	-0.41	0.74	0.91	-0.13	Stable	4.00
ATP5PB	-0.23	0.00	0.00	-0.31	0.02	-0.12	0.00	-0.30	-0.46	-0.30	0.01	-0.23	-0.26	0.11	-0.23	-0.03	0.87	0.91	-0.14	Stable	4.00
ENSSSCG00000037808	0.02	-0.29	-0.09	-0.23	1.07	0.38	0.01	-0.30	-0.44	-0.30	-0.74	0.42	-0.50	-0.43	-0.15	1.07	0.20	-1.42	0.50	Stable	4.00
TRIM7	0.11	-0.03	-0.01	0.03	0.06	0.20	0.16	-0.03	0.26	-0.03	0.08	0.23	0.05	-0.03	-0.03	-0.03	0.66	0.68	-0.55	Stable	2.00
YEATS4	0.05	-0.25	0.06	-1.13	-0.50	0.19	0.40	0.42	0.19	-0.78	-0.51	-0.93	-1.17	-0.02	0.46	-0.48	0.00	-0.24	-2.04	Down	5.00

MOB2	0.41	0.11	0.14	-0.07	0.21	0.09	0.08	0.27	0.05	-0.03	-0.04	0.17	0.00	0.56	0.00	0.11	0.08	3.73	1.90	Stable	2.00
L3HYPDH	0.58	0.82	0.58	0.02	0.03	-0.25	0.58	0.82	0.58	0.09	-0.42	-0.28	0.04	1.01	0.67	-0.48	0.00	-4.46	2.16	Up	1.00
ENSSSCG00000037879	0.08	-0.22	-0.32	0.16	-0.30	0.25	-0.10	-1.00	-0.52	-0.33	0.25	-0.10	0.19	-0.16	-0.10	-0.09	0.06	-102.17	6.67	Stable	6.00
ITGB5	0.08	0.00	0.00	0.01	0.00	0.08	0.08	0.00	0.08	0.08	0.00	0.00	0.08	0.00	0.00	0.00	0.96	0.97	-0.05	Stable	13.00
FITM2	-0.21	-0.10	-0.12	-0.09	0.18	-0.11	0.10	-0.23	-0.12	-0.16	-0.18	0.18	-0.09	0.18	0.25	0.11	0.92	1.38	0.47	Stable	17.00
TRIM28	-0.09	-0.10	-0.06	0.04	-0.58	0.08	-0.09	-0.18	-0.18	-0.05	0.08	-0.09	-0.62	-0.06	-0.09	-0.62	0.35	0.47	-1.09	Stable	6.00
MS4A10	0.59	0.10	0.42	0.64	0.24	0.20	0.59	0.29	0.29	0.14	-0.20	0.34	0.08	0.03	-0.20	0.09	0.59	1.38	0.46	Stable	2.00
RCVRN	0.06	0.12	0.11	0.09	0.07	0.11	0.16	0.07	0.18	0.07	0.09	0.16	0.18	-0.04	0.07	-0.04	0.99	1.01	0.01	Stable	12.00
RBKS	0.34	0.52	0.38	0.13	0.41	-0.16	0.00	0.52	0.35	0.26	0.20	0.15	0.41	0.19	-0.20	-0.22	0.37	1.77	0.82	Stable	3.00
SLC25A33	0.49	0.26	0.70	0.71	0.78	0.55	1.27	0.17	0.11	0.28	0.62	1.27	1.16	0.29	0.51	1.28	0.08	0.57	-0.80	Stable	6.00
HACD1	0.00	0.29	0.00	0.03	-0.10	0.01	0.00	0.00	0.27	0.74	-0.06	0.00	0.37	0.00	0.00	-0.17	0.78	0.68	-0.56	Stable	10.00
NRBP2	0.24	0.70	0.22	0.99	-0.06	0.37	-0.06	0.17	0.23	0.89	0.64	0.82	0.91	0.14	0.33	0.76	0.01	0.37	-1.44	Down	4.00
RAB12	0.17	0.09	0.24	0.29	0.23	0.24	0.17	0.24	0.04	0.27	0.23	0.17	0.29	0.23	0.23	0.23	0.05	0.72	-0.46	Stable	6.00
SPTY2D1OS	-0.12	-0.12	0.32	0.12	-0.12	-0.12	-0.12	0.41	0.12	-0.05	0.11	0.60	-0.12	0.23	0.15	0.08	0.68	1.79	0.84	Stable	2.00
MRPS18C	-0.18	-0.04	-0.11	-0.04	-0.11	-0.04	-0.18	-0.03	-0.08	-0.10	-0.04	-0.06	-0.04	-0.10	-0.10	-0.03	0.08	1.79	0.84	Stable	8.00
ENSSSCG00000038043	0.13	-0.11	0.03	0.04	-0.12	0.09	0.16	0.17	-0.11	-0.06	0.05	0.16	0.13	0.08	0.16	0.08	0.73	1.42	0.51	Stable	6.00
ENSSSCG00000038067	0.03	0.06	0.03	0.03	0.03	0.00	0.00	0.00	0.00	0.00	0.00	0.07	0.03	0.03	0.03	0.00	0.78	1.16	0.22	Stable	2.00
PPP1R14A	0.18	0.31	0.12	0.30	0.37	0.36	0.44	-0.01	0.26	0.08	0.29	0.45	0.25	0.11	0.45	0.51	0.22	0.71	-0.50	Stable	6.00
ECHS1	-0.28	-0.61	-0.31	-0.80	-0.51	-0.18	-0.83	-0.83	-0.30	-0.44	-0.82	-0.53	-0.57	-0.38	-1.39	-0.58	0.70	1.11	0.15	Stable	14.00
ENSSSCG00000038095	-0.21	-0.21	0.15	0.48	-0.33	-0.05	-0.21	-0.21	-0.21	0.48	-0.21	0.14	-0.21	-0.21	0.14	-0.21	0.32	-8.38	3.07	Stable	8.00
ENSSSCG00000038107	0.10	-0.28	-0.85	0.60	0.12	-0.09	0.10	-0.09	-0.27	-0.64	-0.42	-0.24	-0.12	0.31	-0.21	0.14	0.72	1.82	0.87	Stable	8.00
CANT1	0.12	0.11	-0.25	0.14	0.33	0.08	0.28	-0.22	-0.14	-0.05	0.24	-0.37	0.09	0.15	-0.01	-0.03	0.66	0.13	-2.98	Stable	12.00
TFEC	-0.34	-0.34	-0.38	-0.24	-0.09	-0.19	-0.34	-0.29	-0.38	-0.38	-0.24	-0.38	-0.24	-0.29	-0.38	-0.09	0.03	1.48	0.57	Stable	18.00
CMTM3	-0.02	-0.01	0.00	0.08	0.13	0.09	-0.01	0.04	0.05	-0.02	0.22	-0.01	0.13	0.00	-0.02	0.04	0.03	0.02	-5.87	Down	6.00
TMED7	0.24	0.33	0.13	0.21	0.12	0.33	0.15	0.33	0.36	0.29	0.37	0.16	0.18	0.33	0.21	0.29	0.74	1.06	0.09	Stable	2.00
THAP5	-0.16	-0.05	-0.36	-0.30	-0.37	-0.41	-0.09	-0.05	-0.28	-0.27	-0.38	-0.09	-0.49	-0.04	-0.08	-0.37	0.01	0.42	-1.25	Down	18.00
ACBD7	-0.37	-0.57	-0.50	-0.37	-0.41	-0.41	-0.10	-0.48	-0.50	-0.33	-0.48	-0.25	-0.10	-0.24	-0.33	-0.41	0.58	1.11	0.16	Stable	10.00
MPST	0.37	0.27	0.39	-0.03	0.38	0.42	0.51	0.49	0.40	0.27	0.08	0.43	0.19	0.33	0.24	0.21	0.07	1.54	0.62	Stable	5.00
ABT1	-0.26	-0.05	0.06	0.06	-0.18	0.41	-0.16	-0.02	0.14	0.23	0.25	-0.01	0.05	-0.12	-0.16	0.05	0.04	-0.65	-0.61	Stable	7.00
CACUL1	0.13	-0.91	-0.34	-0.57	-0.53	-0.46	-0.02	-0.02	-0.37	-0.88	-0.24	-0.30	-0.67	-0.18	-0.11	-0.31	0.07	0.46	-1.13	Stable	14.00
TMEM175	1.26	1.01	0.86	1.06	1.32	1.21	1.38	0.49	0.66	0.72	1.19	1.08	1.27	0.68	0.44	1.19	0.07	0.75	-0.41	Stable	8.00
NSUN4	-0.16	0.00	0.00	-0.16	0.00	0.00	0.00	0.00	0.00	-0.16	0.00	0.00	0.00	0.00	-0.16	-0.16	0.62	0.67	-0.58	Stable	6.00
RDH13	0.58	0.98	0.24	0.12	0.82	0.42	0.58	0.86	1.51	1.19	0.13	0.58	0.61	0.24	0.58	1.02	0.68	1.14	0.19	Stable	6.00
RANBP1	0.14	-0.13	-0.25	-0.13	0.14	0.14	0.00	0.14	0.14	0.14	-0.13	0.00	0.00	0.14	0.14	0.01	0.80	1.73	0.79	Stable	14.00
POR	-0.21	-0.41	-0.18	-0.03	-0.21	-0.21	-0.21	-0.22	-0.30	-0.05	-0.12	-0.21	-0.16	-0.25	-0.12	-0.22	0.07	1.53	0.62	Stable	3.00
ENSSSCG00000038326	-1.29	-0.40	0.02	-0.35	-0.06	-1.15	-0.73	-0.42	-0.46	-0.69	-0.71	-0.96	-0.24	-1.07	-0.02	-0.39	0.91	0.96	-0.06	Stable	16.00
ENSSSCG00000038327	0.59	0.19	0.53	0.20	0.21	0.20	0.96	0.34	0.34	0.35	0.35	0.96	0.11	0.20	0.96	0.55	0.32	1.41	0.50	Stable	6.00
PIGK	-0.09	-0.37	-0.29	-0.13	-0.15	0.29	-0.17	-0.15	0.38	-0.08	-0.15	-0.24	0.08	-0.22	-0.22	-0.22	0.53	1.86	0.90	Stable	6.00
NME1	-0.02	0.04	-0.06	-0.03	-0.03	0.03	0.05	0.18	0.05	0.14	0.09	0.18	0.10	0.10	-0.03	0.00	0.59	0.65	-0.61	Stable	12.00
ENSSSCG00000038360	0.08	0.34	0.13	0.30	0.05	0.30	0.34	0.02	0.25	0.25	0.30	0.33	0.33	0.34	0.22	0.17	0.54	0.86	-0.22	Stable	10.00
ZCWPW2	0.25	-0.51	-1.22	-1.06	-0.09	-1.00	0.02	-0.47	-0.22	-0.31	-0.09	0.19	-0.81	0.02	0.12	0.29	0.67	0.69	-0.53	Stable	13.00
DYNC2LI1	0.47	0.13	0.10	-0.01	0.33	0.43	-0.32	0.23	-0.30	-0.26	-0.22	0.38	0.05	0.25	-0.15	-0.05	0.82	0.61	-0.72	Stable	3.00
SERPINB6	0.09	0.11	0.11	0.05	-0.26	0.21	-0.19	0.21	0.25	0.11	-0.09	-0.18	0.02	0.05	-0.06	-0.01	0.24	-4.02	2.01	Stable	7.00
STMN3	0.02	0.03	0.09	0.05	0.04	0.01	0.02	0.16	0.19	0.01	0.00	0.04	0.04	0.02	0.02	0.04	0.16	2.48	1.31	Stable	17.00
LRRC8D	0.53	0.11	0.16	-0.28	0.10	-0.35	-0.35	0.42	0.36	-0.28	0.19	0.84	-0.28	-0.03	-0.08	0.07	0.44	-901.52	9.82	Stable	4.00

TMX1	0.32	0.24	0.39	0.08	0.31	0.49	0.32	0.25	0.32	-0.29	0.12	0.47	0.46	0.29	0.32	0.70	0.90	1.05	0.07	Stable	1.00
GTPBP3	-0.27	0.00	-0.02	-0.04	-0.14	-0.61	-0.19	-0.14	-0.14	-0.10	-0.14	-0.01	0.00	-0.05	-0.05	0.02	0.77	0.82	-0.29	Stable	2.00
SURF2	-0.26	-0.30	-0.50	-0.11	-0.27	-0.11	-0.07	-0.07	-0.11	-0.30	-0.07	-0.14	-0.07	-0.07	-0.31	-0.07	0.31	1.48	0.57	Stable	1.00
VAPA	-0.56	-0.04	-0.42	-0.57	-0.18	-0.48	-0.97	-0.76	-0.42	-0.09	-0.77	-0.97	-0.57	0.01	-0.83	-0.92	0.69	0.88	-0.19	Stable	6.00
ENSSSCG00000038475	-0.66	-0.55	-0.69	-0.20	-1.40	-1.53	-0.72	-2.15	-0.52	-0.40	-0.02	-0.80	-0.69	-0.33	-1.18	-0.99	0.74	1.13	0.17	Stable	6.00
HEATR6	0.62	-0.04	-0.09	0.50	0.27	0.18	0.91	-0.02	-0.09	0.12	0.36	0.57	1.45	-0.07	-0.12	0.07	0.17	0.32	-1.66	Stable	12.00
TMPO	-0.69	-0.26	-0.50	-0.76	-0.76	-1.29	-0.31	0.14	-0.75	-1.18	-0.20	-0.76	-0.76	-0.29	-0.14	-1.23	0.01	0.40	-1.31	Down	5.00
LPIN2	-0.07	-0.01	-0.08	-0.07	-0.07	0.00	-0.07	-0.07	0.00	-0.07	-0.07	-0.07	-0.07	-0.07	-0.07	-0.07	0.56	0.87	-0.20	Stable	6.00
ENSSSCG00000038506	-0.19	-0.43	-0.56	-0.30	-0.47	-0.69	0.22	0.15	-0.76	-0.75	-0.67	-0.52	-0.67	0.33	0.11	-0.10	0.04	0.27	-1.88	Down	9.00
SPTBN2	0.24	0.07	-0.28	0.01	0.22	-0.25	0.00	0.26	-0.11	0.06	-0.08	0.19	-0.14	0.16	-0.17	0.06	0.91	1.97	0.98	Stable	2.00
ARSB	0.36	0.65	0.74	0.01	0.35	0.65	0.33	0.38	0.08	0.58	0.11	0.58	0.08	0.39	0.51	0.39	0.47	1.25	0.32	Stable	2.00
GPR158	0.00	0.14	0.03	0.12	0.12	0.15	0.14	0.29	0.29	0.00	0.00	0.12	0.00	0.15	0.03	0.03	0.17	1.98	0.98	Stable	10.00
C10orf105	-0.29	-0.51	-0.42	-0.39	-0.24	-0.25	-0.20	-0.19	-0.61	-0.34	-0.26	-0.37	-0.37	-0.23	-0.41	-0.06	0.30	1.26	0.33	Stable	14.00
FUT1	0.37	0.00	0.14	0.23	0.09	0.11	0.28	-0.08	-0.04	0.22	0.03	0.28	0.38	0.18	0.28	0.12	0.58	0.78	-0.37	Stable	6.00
ENSSSCG00000038588	-0.42	0.55	0.44	-0.04	-0.17	-0.29	-0.17	0.76	-0.22	0.46	-0.30	0.46	0.24	0.37	-0.06	0.52	0.83	1.39	0.47	Stable	9.00
ENSSSCG00000038594	-0.45	0.16	0.52	0.06	-0.31	0.24	-0.35	0.65	0.39	0.64	-0.35	0.12	0.08	-0.07	-0.10	-0.30	0.71	3.99	2.00	Stable	17.00
ADRB2	0.58	0.45	0.38	1.05	0.92	0.05	0.61	0.57	0.15	0.07	0.42	0.93	1.14	0.52	0.13	0.18	0.35	0.71	-0.49	Stable	2.00
INHBB	1.05	0.69	0.25	1.26	0.53	-0.23	0.41	0.44	0.75	0.58	0.79	1.09	0.95	0.62	0.50	0.66	0.54	0.83	-0.26	Stable	15.00
TAF9	0.14	0.12	0.12	-0.04	-0.04	-0.04	0.28	0.12	-0.04	-0.04	-0.04	0.12	-0.02	0.28	0.12	-0.04	0.00	-9.23	3.21	Up	16.00
PCMTD2	0.13	0.00	0.13	0.26	0.26	0.13	0.13	0.00	0.00	0.26	0.26	0.25	0.26	0.13	0.13	0.26	0.00	0.33	-1.58	Down	17.00
ENSSSCG00000038633	0.00	0.00	0.00	0.00	0.00	-0.08	0.00	0.00	0.00	-0.04	-0.04	-0.04	-0.08	0.00	-0.08	0.00	0.13	0.29	-1.81	Stable	14.00
ZBED8	0.00	0.00	-0.53	0.76	0.34	0.33	0.00	0.00	0.34	0.34	0.56	0.00	0.89	0.00	0.00	0.00	0.01	-0.06	-4.13	Down	16.00
ENSSSCG00000038708	-0.97	-0.91	-1.03	-0.10	-0.52	-0.60	-1.13	-0.71	-0.42	-0.91	-0.68	-0.35	-0.32	-1.00	-0.88	-0.32	0.00	1.86	0.89	Stable	7.00
ZNF577	0.55	0.03	0.43	0.11	0.50	0.27	0.86	0.03	0.03	0.28	0.35	0.86	-0.01	0.28	0.86	0.12	0.65	1.24	0.31	Stable	6.00
FLCN	0.14	0.18	-0.15	0.31	0.38	0.46	0.21	0.00	0.09	0.13	0.12	0.38	0.36	0.14	0.22	0.37	0.00	0.34	-1.57	Down	12.00
ENSSSCG00000038717	-0.15	-0.10	-0.08	-0.19	-0.21	-0.17	-0.07	-0.14	-0.10	-0.10	-0.09	0.21	-0.10	0.29	0.40	0.11	0.43	-0.11	-3.18	Stable	7.00
ENSSSCG00000038719	0.57	1.04	0.73	0.96	-0.18	0.49	0.86	0.72	0.72	0.85	0.79	0.48	0.59	0.43	1.30	0.03	0.11	1.59	0.67	Stable	14.00
FAM89A	-0.09	-0.28	-0.38	-0.26	-0.31	-0.16	-0.08	-0.09	-0.09	-0.10	-0.20	-0.17	-0.20	-0.11	0.01	-0.30	0.18	0.65	-0.62	Stable	14.00
MAD1L1	0.01	-0.08	-0.03	-0.14	-0.05	-0.07	0.02	-0.06	0.00	0.04	-0.14	-0.05	-0.14	0.00	0.02	-0.04	0.04	0.17	-2.55	Down	3.00
IKBIP	-0.03	0.30	0.14	0.84	0.84	0.62	0.30	-0.11	0.18	0.45	0.48	0.84	0.84	0.29	0.51	-0.01	0.01	0.32	-1.63	Down	5.00
MAPK13	0.19	0.01	0.38	0.16	0.18	0.55	-0.37	-0.05	-0.21	0.05	0.06	-0.10	0.49	0.20	-0.17	0.49	0.07	-0.01	-6.34	Stable	7.00
ENSSSCG00000038805	0.13	0.47	-0.28	0.43	0.01	0.57	0.18	-0.28	0.44	0.30	0.51	0.18	0.46	0.13	0.18	0.40	0.07	0.34	-1.54	Stable	6.00
MOB3B	-0.47	-0.95	-0.70	-0.35	-0.51	-0.09	-0.09	-0.47	-0.47	-0.08	-0.35	-0.32	-0.70	-0.56	0.06	-0.41	0.44	1.30	0.38	Stable	10.00
FRRS1L	0.92	-0.22	-0.30	-0.08	0.25	0.97	-0.43	0.12	-0.14	0.26	0.26	0.04	0.04	-0.43	0.54	0.16	0.29	0.04	-4.70	Stable	1.00
ENSSSCG00000038829	0.24	-0.13	-0.04	-0.02	-0.28	-0.12	0.24	0.09	0.00	0.09	-0.28	-0.27	0.04	0.07	0.31	-0.16	0.01	-0.76	-0.40	Stable	8.00
PSMF1	0.17	0.08	-0.07	0.40	0.32	0.31	0.33	-0.08	0.28	0.27	0.34	0.07	-0.01	0.32	0.15	0.32	0.19	0.58	-0.78	Stable	17.00
SLC36A3	-0.11	0.06	0.30	-0.13	-0.15	0.12	0.26	0.33	0.73	0.42	-0.02	-0.12	-0.19	0.27	0.00	-0.16	0.05	-8.38	3.07	Up	16.00
CAGE1	0.14	0.20	0.24	0.35	0.14	0.14	0.13	0.20	0.20	0.02	0.20	0.06	0.32	0.10	0.14	0.09	0.94	1.02	0.03	Stable	7.00
IFITM3	0.05	0.24	0.20	0.20	0.23	0.32	0.16	-0.02	0.04	0.20	0.30	0.26	0.10	0.25	0.10	0.14	0.06	0.59	-0.76	Stable	2.00
CTSF	0.67	1.21	0.57	0.82	-0.34	0.37	1.33	-0.06	-0.16	1.32	0.01	0.57	-0.02	0.56	0.53	0.81	0.61	1.32	0.40	Stable	2.00
C6orf120	-0.31	-0.64	-0.66	-0.37	-0.62	-0.37	-0.41	-0.12	-0.53	-0.24	-0.54	-0.39	-0.41	-0.37	-0.33	-0.52	0.87	0.97	-0.04	Stable	1.00
PURG	1.99	1.65	1.91	2.01	2.66	0.98	1.91	1.81	1.04	1.90	2.44	2.02	2.12	1.91	2.13	2.32	0.24	0.87	-0.20	Stable	15.00
SLC17A8	-0.19	-0.55	-0.09	-0.34	-0.34	-0.45	-0.55	-0.76	-0.66	-0.17	-0.55	-0.34	-0.34	-0.08	-0.36	0.05	0.41	1.31	0.39	Stable	5.00
NALCN	-0.15	-0.21	-0.27	-0.33	-0.20	-0.22	-0.22	-0.22	-0.21	-0.59	-0.11	-0.26	-0.13	-0.39	-0.27	-0.32	0.64	0.89	-0.16	Stable	11.00
TMEM263	-0.49	-0.21	-0.50	-0.28	-0.13	0.01	-0.49	-0.47	-0.11	-0.28	-0.01	-0.31	-0.17	-0.24	0.10	-0.32	0.23	1.62	0.70	Stable	5.00

PPP1R12A	-0.10	0.00	-0.08	0.31	0.05	-0.03	-0.10	0.64	0.00	0.00	0.62	0.49	0.56	-0.10	0.10	-0.13	0.19	0.19	-2.38	Stable	5.00
PTPRT	0.45	0.44	0.03	0.14	0.41	0.06	0.18	0.21	0.39	0.44	0.08	0.01	0.43	0.34	0.34	0.41	0.55	1.21	0.28	Stable	17.00
ENSSSCG00000038991	-0.14	-0.11	-0.19	-0.07	-0.17	-0.19	-0.25	-0.05	0.00	-0.17	-0.17	-0.14	-0.17	-0.14	-0.14	-0.17	0.40	0.82	-0.28	Stable	4.00
DNALI1	-0.42	0.02	-0.35	-0.16	-0.16	-0.23	-0.68	-0.60	-0.42	-0.16	-0.42	-0.68	-0.16	-0.09	-0.29	-0.42	0.64	1.17	0.23	Stable	6.00
SLC26A2	0.28	0.03	0.23	0.13	0.76	-0.17	0.35	0.26	0.17	0.08	0.16	0.39	0.84	0.24	0.00	-0.03	0.58	0.72	-0.48	Stable	2.00
DHX33	-0.14	-0.03	0.66	-0.10	-0.02	-0.12	-0.01	0.47	0.93	1.24	-0.10	-0.13	1.01	-0.03	-0.12	-0.02	0.99	0.98	-0.02	Stable	12.00
P4HB	0.37	0.41	0.58	0.33	0.80	0.80	0.65	-0.54	0.13	0.71	0.39	0.83	0.40	0.06	0.58	0.57	0.07	0.46	-1.11	Stable	12.00
ENSSSCG00000039159	-0.62	0.00	0.00	-0.14	-0.16	-0.62	-0.62	0.00	-0.31	-0.62	-0.49	-0.92	-0.23	-1.18	0.00	-0.62	0.47	0.72	-0.48	Stable	13.00
ENSSSCG00000039160	0.00	0.14	0.35	0.40	0.14	0.14	0.00	0.16	0.27	0.38	0.38	0.10	0.38	0.00	0.00	0.19	0.05	0.44	-1.19	Down	17.00
WTIP	-0.02	-0.03	0.02	0.08	-0.01	0.08	0.04	-0.08	-0.08	-0.05	0.06	0.04	-0.05	-0.02	0.04	-0.02	0.25	-1.02	0.03	Stable	6.00
AK8	-0.41	-0.17	-0.62	-0.18	0.07	-0.11	0.07	0.07	-0.11	-0.15	0.07	-0.32	0.07	-0.02	-0.68	-0.26	0.30	2.26	1.17	Stable	1.00
POLR2I	-0.68	0.03	-0.29	-0.60	-0.81	-0.44	-0.49	-0.06	0.15	-0.22	-0.50	-0.49	-0.05	-0.41	-0.49	0.04	0.49	0.73	-0.45	Stable	6.00
PKD1L2	0.14	0.00	0.00	0.69	0.69	0.69	0.35	0.35	0.35	0.69	0.69	0.31	0.69	0.54	0.20	0.69	0.00	0.37	-1.43	Down	6.00
CENPN	-0.05	0.32	0.32	-0.30	-0.41	-0.30	0.17	-0.29	0.01	-0.30	-0.41	-0.08	-0.30	0.09	-0.44	-0.30	0.01	-0.05	-4.29	Down	6.00
SLC38A8	-1.95	-2.10	-2.10	-2.17	-1.45	-2.11	-1.98	-2.04	-2.10	-2.13	-1.50	-2.09	-2.10	-1.87	-2.11	-2.10	0.52	1.04	0.05	Stable	6.00
MC2R	0.03	-0.07	-0.12	0.13	0.19	-0.05	-0.02	0.00	0.10	0.16	0.15	-0.02	0.13	0.08	0.00	0.10	0.03	-0.03	-5.08	Down	6.00
POLR2D	-0.40	-0.52	-0.71	-0.40	-0.51	-0.36	-0.71	-0.49	-0.11	-0.56	-0.40	-0.56	-0.40	-0.71	-0.40	-0.31	0.41	1.16	0.22	Stable	15.00
CD81	0.11	0.03	-0.07	-0.08	-0.02	0.30	-0.16	0.09	0.10	0.15	0.03	0.02	0.11	0.01	-0.41	-0.05	0.23	-0.67	-0.58	Stable	2.00
PHYH	0.10	-0.14	0.22	-0.16	0.09	-0.16	0.08	-0.33	-0.32	0.49	-0.15	0.23	-0.13	0.10	-0.14	0.09	0.42	-1.47	0.56	Stable	10.00
ENSSSCG00000039259	0.29	-0.38	-0.28	0.13	-0.29	-0.07	-0.18	-0.42	-0.61	0.25	0.21	-0.54	0.40	0.17	-0.38	-0.01	0.15	-22.43	4.49	Stable	12.00
HS3ST3B1	0.25	0.26	-0.04	0.70	0.33	0.93	0.51	-0.05	-0.03	0.13	0.37	0.47	1.31	-0.35	0.48	-0.32	0.11	0.27	-1.91	Stable	12.00
C1D	0.12	-0.44	-0.48	-0.26	-0.17	-0.13	-0.69	-0.38	-0.43	-0.70	-0.17	-0.40	-0.24	0.12	-0.06	-0.58	0.71	0.85	-0.23	Stable	3.00
GNA13	-0.27	-0.18	-0.25	-0.26	-0.58	0.05	-0.27	-0.53	-0.47	-0.19	-0.53	-0.52	-0.52	0.05	-0.21	-0.63	0.23	0.67	-0.58	Stable	12.00
PIGH	0.04	0.22	0.56	0.02	0.16	-0.02	0.43	-0.34	-0.34	0.58	0.65	0.43	-0.05	0.13	-0.02	0.26	0.29	0.34	-1.56	Stable	7.00
EMC10	0.19	-0.41	0.76	0.47	-0.10	0.45	0.82	-0.45	-0.71	0.65	0.33	0.82	0.77	0.49	0.82	0.25	0.31	0.41	-1.27	Stable	6.00
ENSSSCG00000039405	-0.23	-0.12	0.35	-0.02	-0.26	-0.23	-0.46	-0.10	0.14	-0.45	0.11	-0.36	0.03	0.14	-0.35	-0.01	0.58	0.54	-0.89	Stable	3.00
ERLIN2	-0.20	-0.70	-0.92	0.04	-0.92	-0.25	-0.92	-0.38	0.01	-0.36	-0.62	-0.41	-0.75	-0.92	-0.20	-0.63	0.82	1.08	0.12	Stable	15.00
DHTKD1	0.11	-0.23	-0.11	-0.23	-0.27	-0.16	-0.01	0.21	-0.16	-0.20	-0.23	-0.01	-0.23	0.21	-0.01	-0.17	0.02	-0.01	-6.14	Down	10.00
PTGFR	0.13	0.02	-0.09	0.06	0.04	0.04	0.11	0.04	0.06	-0.05	0.09	-0.19	0.06	0.06	0.06	-0.08	0.24	-20.03	4.32	Stable	6.00
TDRP	0.10	0.01	-0.17	0.09	-0.05	-0.82	-0.11	-0.10	0.04	-0.40	-0.48	0.01	-0.44	0.02	-0.32	0.01	0.15	0.25	-2.00	Stable	15.00
ENSSSCG00000039569	-0.04	-0.15	-0.28	0.28	-0.10	0.00	-0.21	-0.13	0.19	-0.02	0.10	-0.04	-0.16	-0.03	0.04	-0.07	0.31	67.85	6.08	Stable	12.00
SLPI	0.70	0.49	0.78	0.89	1.38	1.21	1.19	1.09	0.76	-0.04	1.55	0.40	0.90	0.45	1.04	1.33	0.53	0.85	-0.23	Stable	17.00
ENSSSCG00000039587	1.29	-0.20	0.23	1.33	0.98	0.34	1.21	0.68	0.42	0.99	1.47	1.03	1.14	0.25	-0.03	1.27	0.02	0.45	-1.14	Down	5.00
PIN1	0.03	0.06	0.00	0.06	0.03	0.00	0.26	0.16	0.03	0.06	0.02	-0.13	0.06	0.11	0.11	0.11	0.11	3.47	1.80	Stable	2.00
ENSSSCG00000039656	-0.41	-0.20	-0.56	-0.39	-0.26	-0.30	-0.10	-0.02	0.05	-0.15	-0.26	-0.10	-0.05	-0.61	-0.03	-0.56	0.83	0.91	-0.14	Stable	9.00
CHRNA4	0.12	0.66	1.12	1.03	0.95	0.09	0.25	0.69	0.85	0.11	0.16	0.59	0.79	0.27	0.41	0.96	0.83	0.93	-0.11	Stable	17.00
TMEM269	-0.06	-0.21	-0.20	0.31	-0.04	0.31	-0.40	0.30	0.04	0.08	0.30	-0.40	-0.04	-0.27	-0.02	-0.36	0.36	-5.74	2.52	Stable	6.00
ENSSSCG00000039712	-0.34	0.22	0.20	-0.72	-0.72	-0.45	-0.72	-0.22	-0.73	-0.72	-0.72	-0.31	-0.72	-0.23	0.23	-0.72	0.02	0.31	-1.69	Down	3.00
ZDHHC4	-0.11	-0.37	-0.36	-0.17	-0.05	0.05	-0.10	-0.39	-0.03	-0.29	-0.19	-0.11	-0.19	-0.07	-0.37	-0.18	0.24	1.60	0.68	Stable	3.00
ENSSSCG00000039731	0.18	0.06	0.16	-0.22	-0.04	0.06	0.05	-0.01	0.23	0.11	-0.33	-0.26	-0.59	0.06	0.07	0.14	0.03	-0.73	-0.45	Stable	2.00
NLRC5	-0.35	-0.07	-0.09	-0.09	-0.07	-0.05	-0.35	-0.25	-0.38	-0.12	-0.28	-0.39	-0.23	-0.06	0.01	-0.05	0.66	1.20	0.26	Stable	6.00
CMBL	0.04	-0.11	0.37	0.18	0.01	0.09	0.21	0.00	-0.11	-0.06	0.23	0.00	-0.04	0.12	0.16	-0.07	0.56	1.97	0.98	Stable	16.00
SPIRE1	0.52	0.07	0.20	0.69	0.47	0.35	0.44	0.46	0.24	0.51	0.69	0.44	0.69	0.26	0.48	0.57	0.01	0.61	-0.72	Stable	6.00
RTN4RL1	0.21	0.01	0.11	0.86	-0.02	0.04	-0.02	0.83	0.91	0.63	0.66	0.59	0.04	-0.07	-0.11	-0.07	0.59	0.68	-0.56	Stable	12.00
PATJ	0.00	0.09	0.05	0.23	0.02	0.41	0.01	0.53	0.00	0.02	0.20	0.01	0.29	0.03	0.27	0.03	0.75	0.82	-0.29	Stable	6.00

COL25A1	0.17	-0.01	-0.08	0.10	-0.01	-0.01	-0.41	-0.35	-0.44	-0.29	-0.01	0.03	-0.35	-0.29	0.06	-0.35	0.58	1.54	0.62	Stable	8.00
ZSCAN25	-0.33	0.28	0.14	-0.23	0.12	-0.06	-0.16	-0.05	-0.26	-0.69	-0.02	0.32	-0.03	-0.19	-0.38	-0.08	0.79	1.44	0.52	Stable	3.00
PVALEF	-0.35	-0.26	0.24	-0.31	-0.27	-0.47	-0.34	-0.49	-0.33	-0.33	0.01	-0.16	-0.41	-0.40	-0.35	-0.21	0.86	1.07	0.09	Stable	12.00
HDHD3	0.29	1.30	1.29	-0.01	0.02	-0.16	0.06	0.54	0.36	0.13	-0.11	0.51	0.78	0.30	0.76	0.42	0.06	3.11	1.64	Stable	1.00
RASL11A	0.34	1.01	0.42	0.98	1.52	0.76	0.40	0.79	1.16	1.18	0.28	1.37	0.95	0.36	0.93	1.25	0.07	0.65	-0.61	Stable	11.00
ENSSSCG00000039915	-0.19	0.03	-0.14	0.34	0.06	-0.44	-0.11	0.19	-0.36	-0.25	0.35	-0.11	-0.10	0.05	-0.50	0.11	0.34	26.33	4.72	Stable	14.00
NXPH4	0.12	0.22	-0.23	0.10	0.10	-0.11	-0.15	-0.10	-0.08	0.26	0.21	0.26	0.17	0.06	0.00	0.04	0.05	-0.16	-2.65	Stable	5.00
NANOS1	-0.04	0.39	0.13	-0.18	-0.13	-0.27	0.27	0.00	-0.13	0.29	0.10	0.33	-0.13	0.25	-0.16	0.25	0.62	2.73	1.45	Stable	14.00
LSMEM1	0.00	0.00	0.00	0.01	0.00	0.00	0.00	0.00	0.00	0.00	0.00	0.00	0.00	0.00	0.00	0.00	0.35	0.00	#NAME?	Stable	18.00
ENSSSCG00000040056	0.21	-0.16	0.34	-0.20	0.47	0.04	0.66	0.80	-0.02	0.55	-0.21	0.28	-0.02	0.34	0.29	-0.24	0.18	3.63	1.86	Stable	5.00
APEX1	-0.07	-0.18	-0.01	-0.35	-0.12	-0.36	-0.32	-0.38	-0.07	-0.15	-0.22	-0.14	-0.10	-0.07	-0.24	-0.20	0.57	0.83	-0.27	Stable	7.00
GPR35	0.23	0.13	0.25	0.17	0.25	0.02	-0.13	-0.03	0.31	0.13	0.25	0.25	0.33	-0.21	0.16	0.25	0.15	0.43	-1.20	Stable	15.00
ENSSSCG00000040103	0.00	0.06	-0.08	-0.11	0.06	-0.11	0.00	-0.02	0.00	0.00	-0.27	-0.19	-0.27	0.00	0.00	0.06	0.08	0.04	-4.61	Stable	3.00
GMEB2	0.00	-0.05	-0.02	-0.11	0.00	0.00	0.00	-0.05	-0.09	0.00	0.00	0.00	0.00	0.00	0.00	0.00	0.51	1.88	0.91	Stable	17.00
ENSSSCG00000040118	0.01	0.21	0.19	0.31	-0.11	0.00	-0.17	0.25	0.43	0.11	0.11	-0.10	0.17	-0.28	-0.17	-0.12	0.92	1.23	0.30	Stable	17.00
NOL3	-0.50	-0.18	0.11	0.10	0.18	0.15	0.10	0.52	0.18	-0.50	0.31	-0.15	0.28	0.02	-0.37	0.13	0.61	-0.25	-1.98	Stable	6.00
B3GNT9	-0.41	-0.37	-0.16	0.18	0.24	0.06	-0.21	0.33	0.05	-0.41	0.22	-0.21	0.44	-0.16	-0.34	0.30	0.07	-1.53	0.61	Stable	6.00
BCDIN3D	-0.45	0.27	-0.28	-0.03	-0.53	0.67	-0.45	0.60	0.44	0.36	0.04	0.46	0.48	1.02	0.55	-0.19	0.81	1.38	0.46	Stable	5.00
PRSS45P	-0.04	-0.34	-0.28	-0.23	-0.13	-0.30	-0.04	-0.31	-0.33	-0.22	-0.29	-0.13	-0.23	-0.04	-0.04	-0.27	0.46	0.80	-0.32	Stable	13.00
MRPL36	0.11	-0.13	0.17	-0.07	-0.13	-0.04	0.11	-0.13	-0.13	-0.13	-0.10	-0.04	-0.07	0.04	-0.07	-0.07	0.13	0.04	-4.48	Stable	16.00
MMP24	0.00	0.13	0.19	0.00	0.12	0.06	0.03	0.25	0.19	0.00	0.19	0.09	0.00	0.19	0.13	0.12	0.13	1.85	0.89	Stable	17.00
CDK6	0.25	0.17	0.24	0.23	0.25	0.36	0.09	0.12	0.10	0.17	0.17	0.00	0.09	0.01	0.17	0.25	0.39	0.77	-0.38	Stable	9.00
C12orf57	0.28	0.31	0.26	0.07	0.07	0.25	0.22	0.15	0.19	0.07	-0.24	0.16	0.07	0.30	0.30	0.20	0.01	3.09	1.63	Up	5.00
P2RY2	-1.16	-0.77	-1.25	-1.30	-0.20	0.00	-0.08	0.24	0.69	0.25	-0.91	-0.08	0.26	-1.41	-0.63	-0.83	0.57	1.56	0.64	Stable	9.00
ZNF862	0.01	-0.28	0.01	0.01	0.17	0.17	0.01	0.05	-0.21	0.01	0.21	0.01	0.01	-0.24	-0.13	0.09	0.01	-1.15	0.21	Stable	18.00
PSKH1	0.05	0.01	0.01	0.11	0.12	0.05	0.05	0.05	0.05	0.05	0.14	0.05	0.14	0.01	0.05	-0.04	0.09	0.44	-1.19	Stable	6.00
COX7C	0.31	0.42	0.41	0.41	0.42	0.42	0.03	0.20	0.14	0.16	0.59	0.50	0.73	0.42	0.31	0.26	0.08	0.64	-0.64	Stable	2.00
NXNL1	-0.49	-0.12	0.00	-0.13	-0.02	-0.90	-0.08	-0.15	-0.20	-0.06	-0.66	-0.20	-0.14	-0.14	-0.13	-0.13	0.37	0.58	-0.78	Stable	2.00
TES	0.09	0.18	0.09	0.13	0.66	0.29	-0.03	0.18	-0.10	-0.06	0.08	0.06	0.33	0.18	0.06	0.66	0.10	0.30	-1.71	Stable	18.00
GOLGA7B	-0.03	-0.05	-0.02	0.01	-0.01	-0.01	-0.05	0.03	0.00	-0.04	0.01	0.01	0.00	0.00	-0.10	-0.01	0.16	6.75	2.75	Stable	14.00
DCAF16	0.00	0.24	-0.04	0.54	0.36	0.50	0.51	0.76	1.14	1.14	-0.01	-0.28	-0.14	0.09	0.08	0.76	0.96	0.97	-0.05	Stable	8.00
ENSSSCG00000040416	-0.14	-0.04	-0.10	-0.03	0.04	-0.26	-0.09	-0.23	-0.31	0.04	-0.38	-0.09	-0.18	-0.24	-0.05	0.04	0.48	1.47	0.56	Stable	2.00
ENSSSCG00000040419	0.30	0.29	0.39	0.67	0.49	0.86	0.89	0.04	0.39	0.38	0.61	0.89	0.85	0.01	0.79	0.67	0.04	0.57	-0.81	Stable	6.00
YRDC	-0.18	0.36	0.20	0.59	0.25	-0.04	0.05	-0.05	-0.05	0.27	0.35	0.05	0.59	0.00	-0.20	0.31	0.02	0.06	-4.11	Down	6.00
TMED5	0.27	0.25	0.66	0.36	0.24	0.31	0.18	0.23	0.16	0.29	0.33	0.48	0.30	0.15	0.28	0.42	0.30	0.79	-0.33	Stable	4.00
ENSSSCG00000040448	0.48	0.24	0.39	-0.16	0.05	-0.01	0.48	0.61	0.56	0.50	-0.07	0.46	-0.23	0.34	0.15	0.21	0.02	4.30	2.10	Up	8.00
ABHD12	0.19	-0.02	0.15	0.23	0.34	0.29	0.53	0.14	0.25	0.11	0.41	0.20	0.13	0.18	0.56	0.34	0.92	0.97	-0.05	Stable	17.00
AQP3	-0.15	-0.33	-0.18	-0.11	-0.15	0.00	-0.08	-0.15	-0.28	-0.22	-0.15	-0.32	-0.24	-0.32	-0.15	-0.17	0.46	1.21	0.28	Stable	10.00
TTC1	0.00	0.00	-0.37	-0.37	-0.24	-0.05	-0.18	-0.18	-0.05	-0.05	-0.37	-0.18	-0.17	0.00	-0.18	-0.23	0.19	0.58	-0.78	Stable	16.00
C19orf54	0.05	-0.53	-0.17	0.01	0.49	-0.08	0.16	-0.31	-0.37	-0.22	-0.13	0.16	0.24	-0.24	0.16	0.55	0.05	-1.24	0.31	Stable	6.00
ENSSSCG00000040535	0.97	1.23	0.42	0.65	0.36	0.40	0.73	0.44	0.44	0.77	0.61	1.43	0.65	0.17	0.21	0.57	0.56	0.84	-0.24	Stable	4.00
ENSSSCG00000040556	-0.10	-0.19	-0.03	-0.10	-0.27	-0.04	-0.07	-0.07	-0.07	-0.10	0.00	-0.07	-0.26	-0.06	-0.07	-0.07	0.47	0.74	-0.43	Stable	6.00
ZNF691	0.00	0.08	0.14	0.02	0.08	0.13	-0.06	0.14	0.05	-0.08	0.12	-0.06	0.05	0.12	0.15	0.00	0.26	2.31	1.21	Stable	6.00
N4BP2L1	0.15	0.19	0.12	0.12	0.19	0.14	0.15	0.11	0.33	0.06	0.20	0.15	0.19	0.21	-0.17	0.27	0.59	0.82	-0.29	Stable	11.00
PLA2G10	0.31	0.09	0.07	0.06	0.00	0.06	0.06	0.07	0.10	0.12	0.07	0.24	0.07	0.25	0.38	0.06	0.14	1.96	0.97	Stable	3.00

ENSSSCG00000040586	-0.36	0.52	-0.05	0.79	0.41	-0.27	0.46	0.64	-0.05	0.26	0.46	0.18	0.07	-0.28	0.98	0.65	0.68	0.73	-0.46	Stable	14.00
C3orf20	-0.07	-0.27	-0.02	-0.09	-0.26	-0.34	-0.02	0.03	-0.16	-0.14	-0.45	-0.10	-0.16	-0.03	-0.14	-0.13	0.05	0.41	-1.29	Stable	13.00
TNFAIP8	0.02	0.40	0.36	0.54	0.40	0.40	0.12	0.40	0.30	0.63	0.92	0.57	0.85	0.40	0.38	0.33	0.01	0.52	-0.96	Stable	2.00
REXO1	0.20	0.21	0.67	0.19	0.51	0.51	0.06	0.19	0.19	0.19	0.20	0.33	0.34	0.20	0.20	0.20	0.41	0.78	-0.36	Stable	2.00
THAP12	0.12	0.32	0.29	0.16	0.42	0.48	0.13	0.61	0.32	0.61	0.49	0.29	0.61	0.47	0.49	0.22	0.45	0.84	-0.26	Stable	9.00
SURF4	0.00	0.00	-0.40	0.00	-0.22	0.00	-0.01	-0.01	0.00	-0.08	-0.01	-0.01	-0.08	-0.01	0.00	-0.01	0.97	1.05	0.07	Stable	1.00
ASPSCR1	-0.49	0.06	-0.15	-0.23	-0.49	-0.17	-0.49	-0.73	-0.26	-0.24	-0.29	-0.04	-0.49	-0.56	-0.28	-0.33	0.48	1.27	0.35	Stable	12.00
C11orf86	0.15	0.15	0.15	0.15	0.15	0.04	0.15	0.15	0.11	0.15	0.02	0.15	0.11	0.15	0.07	0.15	0.40	1.17	0.23	Stable	2.00
DDAH1	-0.47	-0.38	-0.16	-0.57	-0.55	-0.17	-0.31	-0.44	-0.27	-0.31	-0.51	-0.23	-0.83	-0.57	-0.31	0.11	0.88	0.95	-0.07	Stable	4.00
LRP2	-0.08	-0.09	-0.34	-0.41	-0.34	-0.51	-0.34	-0.34	0.13	-0.32	0.02	-0.39	0.02	-0.34	-0.11	-0.65	0.22	0.58	-0.78	Stable	15.00
IPP	0.33	0.07	0.38	-0.45	0.09	-0.07	-0.11	0.33	-0.01	0.44	-0.01	0.02	0.14	0.19	0.46	0.47	0.34	2.56	1.36	Stable	6.00
CNTN2	-0.42	-0.51	-0.54	-0.42	-0.52	-0.33	-0.54	-0.47	-0.64	-0.42	-0.49	-0.52	-0.43	-0.41	-0.11	-0.43	0.86	1.02	0.04	Stable	9.00
CFAP298-TCP10L	-0.50	-0.23	-0.01	0.18	0.10	0.15	-0.25	-0.49	-0.33	-0.16	-0.04	-0.20	-0.16	-0.16	-0.25	0.21	0.00	-25.52	4.67	Up	13.00
CNBP	-0.21	0.48	0.00	0.36	0.84	0.33	-0.28	0.58	0.45	0.44	0.55	-0.47	0.19	-0.23	-0.16	0.33	0.21	0.25	-2.02	Stable	13.00
KCTD13	0.23	0.04	0.18	0.57	0.23	0.39	0.46	0.34	0.53	0.23	0.26	0.29	0.23	0.60	0.00	0.46	0.70	0.89	-0.16	Stable	3.00
ENSSSCG00000040889	0.05	0.05	-0.20	-0.46	-0.37	-0.10	-0.20	0.31	0.08	0.31	-0.66	-0.12	-0.06	0.05	0.05	-0.37	0.06	-0.12	-3.10	Stable	4.00
ENSSSCG00000040918	0.02	0.00	0.00	0.11	-0.02	0.21	0.02	0.00	0.00	0.05	0.49	0.03	0.00	0.03	0.00	-0.02	0.15	0.08	-3.62	Stable	1.00
CCL16	0.01	0.00	0.31	0.32	0.86	0.59	0.42	0.37	0.35	0.79	0.32	0.11	0.00	0.00	0.00	0.43	0.08	0.42	-1.24	Stable	12.00
ENSSSCG00000040956	-1.01	-0.76	-0.67	-0.97	-0.86	-0.96	-1.19	-0.69	-0.37	-0.71	-1.12	-0.69	-0.87	-0.58	-0.31	-1.19	0.10	0.76	-0.40	Stable	2.00
PPIL1	0.04	0.03	0.06	-0.17	0.15	-0.08	0.30	0.12	-0.09	0.45	-0.06	0.22	-0.20	0.08	-0.22	-0.21	0.77	3.54	1.82	Stable	7.00
ENSSSCG00000041010	0.13	0.14	0.00	0.16	0.16	0.18	0.15	0.16	0.16	0.18	0.15	0.16	0.14	0.31	0.10	0.14	0.68	0.92	-0.13	Stable	12.00
ENSSSCG00000041114	0.11	0.17	0.12	-0.02	-0.20	-0.01	0.06	0.69	0.24	0.42	-0.09	0.06	-0.14	0.18	0.06	-0.10	0.05	-19.54	4.29	Up	6.00
ENSSSCG00000041248	0.03	0.20	0.15	-0.08	0.10	0.20	0.01	0.11	-0.09	0.10	0.00	0.02	0.00	0.11	0.12	0.12	0.62	1.40	0.48	Stable	2.00
ENSSSCG00000041364	0.01	0.07	-0.17	-0.06	-0.06	-0.40	0.13	-0.04	0.13	-0.09	-0.29	-0.04	-0.16	0.39	0.13	-0.27	0.00	-0.46	-1.10	Down	7.00
ENSSSCG00000041378	-0.15	-0.33	-0.24	-0.38	-0.37	-0.43	0.37	0.13	-0.36	0.23	-0.25	-0.16	-0.54	-0.22	0.32	-0.43	0.11	0.20	-2.29	Stable	1.00
MFSD4B	0.11	-0.02	0.28	-0.36	0.22	0.44	0.03	0.19	-0.13	-0.10	0.10	0.41	0.05	0.19	-0.02	0.04	0.84	0.78	-0.36	Stable	1.00
ENSSSCG00000041442	0.06	0.48	0.10	0.34	0.22	0.08	0.06	0.18	0.49	0.03	0.55	0.33	0.34	0.32	0.58	0.06	0.69	1.17	0.22	Stable	14.00
ENSSSCG00000041465	0.27	0.15	0.49	0.27	0.25	0.31	0.25	0.61	0.24	0.16	0.59	0.52	0.48	0.52	0.44	0.61	0.75	0.93	-0.10	Stable	13.00
CCDC194	-0.23	-0.37	-0.27	-0.31	-0.26	-0.08	0.08	-0.64	-0.06	-0.02	-0.16	-0.23	-0.52	-0.49	-0.51	-0.51	0.65	1.19	0.25	Stable	2.00
DMKN	0.62	0.21	0.61	-0.03	-0.09	0.18	1.22	0.51	0.57	0.21	0.42	1.22	0.20	0.18	1.22	0.70	0.18	1.83	0.87	Stable	6.00
ENSSSCG00000041595	0.01	0.01	0.01	0.86	0.29	0.86	0.01	0.01	0.01	0.15	0.86	0.30	0.86	0.01	0.01	0.86	0.00	0.01	-6.36	Down	1.00
REELD1	0.00	0.64	0.00	0.51	0.38	0.00	0.00	0.23	0.00	0.53	0.52	0.43	0.90	0.35	0.73	0.38	0.14	0.53	-0.91	Stable	8.00
ENSSSCG00000041628	-0.05	-0.38	-0.11	-0.45	-0.74	-0.75	-0.27	-0.86	-0.37	-0.74	-0.74	-0.47	-0.74	-0.51	-0.47	-0.74	0.01	0.56	-0.83	Stable	4.00
ENSSSCG00000041731	0.20	-0.07	0.08	0.06	-0.18	0.00	-0.01	0.36	0.33	0.23	-0.17	0.21	0.28	0.37	0.07	-0.18	0.17	5.08	2.35	Stable	17.00
ENSSSCG00000041764	-0.07	0.01	0.05	0.00	0.01	0.01	-0.07	-0.10	0.01	0.01	0.02	-0.17	0.01	0.11	0.56	-0.12	0.28	-2.09	1.07	Stable	5.00
ENSSSCG00000041783	0.00	0.00	0.00	0.00	-0.01	-0.01	0.02	0.00	-0.04	-0.02	0.05	0.03	-0.01	-0.02	-0.01	-0.02	0.49	-2.80	1.49	Stable	3.00
ENSSSCG00000041863	-0.02	-0.04	0.24	0.04	0.05	0.57	0.00	0.09	0.12	0.08	0.05	0.04	0.16	-0.07	-0.04	0.32	0.13	0.22	-2.20	Stable	13.00
ENSSSCG00000041954	-1.11	-0.50	-0.85	-0.62	-1.34	-0.65	-0.32	-0.07	-0.43	-0.32	-0.92	-0.61	-0.74	-0.58	-0.79	-1.36	0.19	0.71	-0.50	Stable	2.00
ENSSSCG00000042026	0.07	0.45	0.13	-0.04	-0.31	-0.04	0.20	0.48	0.48	0.19	0.07	0.16	-0.07	0.08	0.16	0.09	0.01	42.52	5.41	Up	6.00
ENSSSCG00000042040	0.54	-0.24	0.10	0.47	0.54	0.50	0.54	-0.24	-0.24	0.16	0.38	0.54	0.17	0.08	0.54	0.02	0.18	0.39	-1.35	Stable	6.00
ENSSSCG00000042067	-0.43	0.06	-0.49	-0.22	-0.31	-0.16	0.05	-0.35	0.12	1.05	-0.35	-1.00	-0.25	-0.38	0.07	-0.53	0.82	0.76	-0.39	Stable	10.00
ENSSSCG00000042157	1.12	0.74	0.80	0.93	0.56	0.50	1.12	0.77	0.65	0.77	0.78	1.08	0.96	1.12	1.12	0.86	0.24	1.15	0.21	Stable	13.00
ENSSSCG00000042169	-0.13	0.15	-0.10	0.24	-0.02	0.04	-0.16	-0.04	-0.24	0.06	0.23	0.16	0.26	-0.04	0.00	0.14	0.00	-0.52	-0.95	Stable	11.00
ENSSSCG00000042192	-0.14	-0.20	-0.12	-0.39	-0.49	-0.01	-0.04	-0.05	-0.84	0.21	-0.29	-0.10	-0.46	-0.32	-0.82	-0.17	0.49	1.49	0.57	Stable	14.00
ENSSSCG00000042255	0.24	0.18	0.16	0.50	0.37	0.38	0.17	-0.20	0.71	0.65	0.58	0.10	0.35	0.19	0.21	0.15	0.13	0.54	-0.90	Stable	2.00

ENSSSCG00000042730	0.57	0.13	-0.02	0.58	0.13	0.58	-0.08	0.15	-0.03	0.30	0.18	0.25	0.31	0.49	0.25	0.58	0.12	0.50	-1.00	Stable	15.00
ENSSSCG00000042801	-0.12	-0.05	-0.13	-0.11	0.92	0.78	0.04	-0.01	-0.07	-0.12	-0.27	-0.03	0.72	-0.01	-0.06	0.92	0.07	-0.14	-2.81	Stable	18.00
C1orf146	-0.09	-0.07	-0.35	-0.15	-0.20	-0.02	-0.04	-0.06	-0.07	0.02	-0.12	-0.21	0.02	0.12	0.02	-0.16	0.55	0.65	-0.61	Stable	4.00
ENSSSCG00000042809	-0.75	0.09	0.11	-0.98	-0.62	-0.85	-0.34	-0.23	0.02	-0.25	-0.40	-0.34	-0.22	-0.01	-0.34	-0.11	0.08	0.39	-1.37	Stable	6.00
ENSSSCG00000042830	0.18	0.15	0.28	0.85	1.01	1.29	0.35	1.27	-0.10	0.73	0.64	0.74	0.94	0.37	1.16	0.64	0.06	0.53	-0.90	Stable	6.00
ENSSSCG00000042941	-0.23	-0.45	0.02	-0.12	-0.05	0.11	-0.21	0.20	0.35	-0.33	-0.66	-0.08	0.07	-0.22	-0.11	-0.19	0.55	0.51	-0.98	Stable	16.00
ENSSSCG00000043116	0.17	0.45	0.37	0.00	0.24	0.00	0.41	0.67	0.67	0.33	0.09	0.41	0.27	0.15	0.41	0.59	0.11	1.72	0.78	Stable	6.00
ENSSSCG00000043289	-0.14	-0.08	-0.18	-0.11	0.26	0.21	-0.08	-0.07	-0.04	0.26	0.27	-0.29	-0.04	-0.52	-0.19	-0.01	0.03	-2.41	1.27	Up	3.00
IFN-ALPHA-8	0.00	0.04	0.00	0.64	0.28	0.90	0.00	0.52	0.00	0.46	0.60	0.35	0.52	0.84	-0.05	0.90	0.01	0.29	-1.77	Down	1.00
ENSSSCG00000043327	1.09	0.79	0.78	0.68	0.55	0.35	1.09	0.79	0.88	0.78	0.78	0.95	0.69	1.09	1.09	0.70	0.01	1.39	0.48	Stable	13.00
ENSSSCG00000043356	0.14	0.12	0.17	0.04	0.10	0.12	0.09	0.09	0.10	0.18	0.09	0.07	0.09	0.09	0.18	0.18	0.53	1.13	0.17	Stable	1.00
ENSSSCG00000043374	0.00	0.00	0.01	0.01	0.01	0.00	0.00	0.00	0.00	0.01	0.00	0.01	0.01	0.01	0.00	0.01	0.08	0.33	-1.58	Stable	9.00
ENSSSCG00000043413	-0.45	-0.28	-0.43	-0.14	-0.38	-0.02	-0.31	-0.03	-0.18	-0.41	-0.13	-0.28	0.04	-0.33	-0.44	0.03	0.10	1.90	0.92	Stable	7.00
ENSSSCG00000043485	0.05	0.26	0.26	0.02	0.07	0.14	0.34	0.50	0.40	0.16	0.10	0.19	0.11	0.21	0.19	0.06	0.01	2.58	1.37	Up	6.00
ENSSSCG00000043694	-0.31	-0.14	0.21	0.14	-0.05	-0.07	-0.11	-0.27	-0.10	-0.18	0.10	0.13	0.33	-0.04	-0.25	-0.06	0.06	-2.99	1.58	Stable	12.00
ENSSSCG00000043876	-0.04	-0.10	0.00	-0.02	-0.15	0.00	-0.04	-0.15	-0.04	-0.04	-0.04	0.00	-0.02	-0.02	-0.04	-0.15	0.99	1.01	0.01	Stable	13.00
ENSSSCG00000043893	0.04	0.57	0.20	0.15	-0.05	-0.01	0.29	0.44	0.51	0.36	0.35	0.66	0.15	0.38	0.85	0.30	0.16	1.74	0.80	Stable	5.00
ENSSSCG00000043909	-0.54	-0.23	-0.57	-0.03	-0.08	0.06	-0.43	-0.18	-0.04	0.11	0.26	-0.20	0.06	-0.29	-0.24	0.28	0.00	-5.58	2.48	Up	5.00
ENSSSCG00000044125	0.09	0.09	0.09	0.32	-0.25	-0.35	0.09	0.09	0.09	0.37	-0.13	-0.04	-0.32	0.09	0.09	-0.09	0.15	-1.51	0.59	Stable	1.00
ENSSSCG00000044155	0.00	0.00	0.04	0.00	0.00	0.00	0.00	0.00	0.00	0.00	0.00	0.00	0.00	0.04	0.00	0.00	0.17	Inf	Inf	Stable	2.00
ENSSSCG00000044206	-0.45	-0.22	0.00	-0.25	-0.38	-0.46	-0.21	-0.23	-0.01	-0.45	-0.20	-0.23	-0.22	-0.22	-0.25	-0.20	0.15	0.67	-0.58	Stable	6.00
SMKR1	0.04	-0.03	0.00	0.02	0.04	0.02	0.04	0.02	-0.09	0.02	0.02	-0.01	0.02	-0.07	0.00	0.04	0.12	-0.60	-0.74	Stable	18.00
EEA1	-0.04	0.21	-0.65	0.08	-0.67	-0.32	-0.34	-0.13	-0.60	-0.37	-0.20	-0.65	-0.81	-0.82	0.58	-0.35	0.35	0.54	-0.88	Stable	5.00
ENSSSCG00000044372	0.50	0.14	-0.15	0.17	0.01	0.15	0.22	0.16	0.37	0.40	-0.01	0.26	0.14	0.34	0.26	0.02	0.31	1.63	0.71	Stable	14.00
COL19A1	-0.11	0.14	-0.06	0.00	0.00	0.00	0.00	0.00	0.14	0.00	0.00	-0.11	0.00	-0.11	0.00	0.00	0.70	-0.07	-3.91	Stable	1.00
ENSSSCG00000044574	0.90	0.49	1.03	0.24	0.19	0.73	1.02	-0.08	0.40	-0.23	0.30	0.12	0.39	0.63	0.16	0.40	0.11	2.13	1.09	Stable	7.00
ENSSSCG00000045084	-0.46	-0.53	-0.58	-0.23	-0.10	0.05	-0.44	-0.39	-0.16	-0.41	-0.02	-0.42	-0.54	-0.47	-0.16	-0.40	0.16	1.55	0.64	Stable	3.00
ENSSSCG00000045256	-0.05	0.01	-0.11	-0.07	0.23	-0.03	-0.09	0.11	0.19	0.26	0.02	0.00	0.06	0.06	0.06	0.11	0.42	0.35	-1.52	Stable	2.00
ENSSSCG00000045290	0.30	0.00	-0.08	0.00	-0.17	0.33	0.20	0.00	0.16	0.00	0.08	0.23	0.00	0.00	0.00	0.00	0.84	1.24	0.32	Stable	2.00
ENSSSCG00000045496	0.24	0.20	0.31	0.00	0.26	0.00	0.47	0.20	0.20	0.07	0.24	0.47	0.21	0.10	0.47	0.48	0.49	1.27	0.35	Stable	6.00
CCDC180	0.01	-0.20	-0.16	-0.24	-0.05	-0.05	-0.19	-0.06	-0.54	-0.24	-0.06	0.10	-0.06	0.13	-0.07	-0.18	0.66	1.37	0.45	Stable	1.00
EEF1AKMT4	0.00	0.46	0.72	0.00	0.00	0.46	0.00	0.30	0.00	0.00	0.00	0.00	0.00	0.00	0.00	0.00	0.29	3.21	1.68	Stable	13.00
ENSSSCG00000045560	-0.18	-0.36	-0.36	-0.14	-0.12	-0.11	-0.15	-0.49	-0.36	-0.10	-0.36	-0.15	-0.36	-0.14	-0.15	-0.14	0.17	1.47	0.56	Stable	10.00
ENSSSCG00000045652	-0.26	-0.26	-0.26	-0.03	-0.08	0.10	-0.26	-0.26	-0.26	-0.14	0.18	-0.04	0.18	-0.26	-0.26	0.08	0.00	-8.48	3.08	Up	1.00
ENSSSCG00000045703	0.59	0.56	0.51	0.59	0.56	0.63	0.64	0.27	0.48	0.56	0.59	0.48	0.71	0.48	0.51	0.59	0.09	0.86	-0.22	Stable	8.00
PXMP2	0.08	-0.08	0.22	0.11	0.20	0.04	0.17	0.01	-0.20	-0.05	0.68	0.16	0.11	0.08	-0.21	0.07	0.13	0.04	-4.49	Stable	14.00
ENSSSCG00000045776	-0.27	-0.44	-0.45	0.07	-0.11	0.07	-0.57	-0.23	-0.61	-0.11	-0.13	-0.57	0.07	-0.16	-0.51	-0.22	0.01	3.53	1.82	Up	6.00
ENSSSCG00000045794	-0.70	-0.42	-0.30	-0.11	-0.27	-0.37	-0.45	-0.50	-0.32	-0.30	-0.30	-0.56	-0.47	-0.55	-0.24	-0.42	0.27	1.24	0.31	Stable	12.00
ENSSSCG00000045883	0.14	0.30	0.11	0.08	0.15	0.08	0.12	0.23	0.30	0.16	0.06	0.12	0.16	0.16	0.12	0.11	0.05	1.63	0.70	Stable	6.00
ENSSSCG00000045892	-0.47	-0.37	-0.39	-0.23	-0.33	-0.24	-0.25	-0.33	-0.09	-0.26	-0.37	-0.24	-0.29	-0.38	-0.26	-0.23	0.34	1.17	0.22	Stable	3.00
ADAM2	-0.37	-0.37	-0.37	-0.18	-0.37	-0.18	-0.37	-0.37	-0.37	-0.21	-0.16	-0.37	0.00	-0.37	-0.37	-0.16	0.01	1.81	0.85	Stable	15.00
TSC1	-1.03	-0.32	-1.33	0.15	-0.36	-0.05	-0.51	-0.51	0.02	-0.10	-0.51	-0.68	-0.51	-0.40	-1.16	-0.55	0.12	2.01	1.01	Stable	1.00
IFT22	1.02	0.00	0.64	0.43	0.37	0.53	1.02	0.09	0.53	0.91	0.74	0.74	0.46	1.28	1.32	0.35	0.39	1.30	0.38	Stable	3.00
TEX26	-0.40	-0.75	-1.20	-1.08	-0.76	-0.72	-0.45	-1.11	-0.40	0.47	-1.32	-0.68	-2.20	-0.18	-0.34	-2.38	0.20	0.56	-0.84	Stable	11.00
PSMD2	-0.63	-0.06	-0.10	-0.14	-0.38	-0.12	-0.51	-0.01	-0.31	-0.38	-0.38	-0.32	-0.47	-0.28	-0.29	-0.33	0.66	0.87	-0.20	Stable	13.00

OAS1	1.37	1.49	1.05	-0.09	-0.05	0.51	1.28	1.51	1.32	1.33	-0.06	0.63	0.84	1.61	0.56	0.80	0.00	2.61	1.38	Up	14.00
ENSSSCG00000046656	0.09	-0.31	0.29	0.46	0.36	0.53	0.47	-0.10	-0.50	-0.12	0.23	0.47	0.21	-0.10	0.26	0.31	0.05	0.04	-4.52	Stable	6.00
ENSSSCG00000046700	0.17	0.17	0.35	0.35	0.35	0.17	0.35	0.17	0.17	0.00	0.00	0.00	0.17	0.35	0.17	0.35	0.34	1.38	0.46	Stable	12.00
ZNF391	0.34	0.09	0.27	0.19	0.28	0.36	0.11	0.17	0.15	0.21	0.31	0.34	0.20	0.36	0.32	0.18	0.48	0.87	-0.20	Stable	7.00
ENSSSCG00000047029	-2.69	-0.63	-0.80	-0.69	0.52	-1.73	-1.38	-0.94	-1.79	-1.73	-0.74	-0.96	-1.63	-0.61	-1.93	-1.05	0.37	1.34	0.43	Stable	15.00
ENSSSCG00000047199	0.27	0.24	0.13	0.09	0.39	0.57	-0.02	0.45	0.52	0.44	0.25	0.24	0.20	0.11	0.33	0.49	0.37	0.76	-0.40	Stable	10.00
ENSSSCG00000047274	0.00	-0.08	0.00	-0.08	-0.08	-0.08	-0.04	-0.12	-0.04	-0.08	-0.12	-0.17	-0.17	-0.08	-0.08	-0.12	0.01	0.50	-1.01	Down	8.00
ENSSSCG00000047307	-0.03	0.18	0.04	0.04	0.04	0.00	0.00	0.14	-0.08	0.14	0.06	0.15	-0.15	-0.13	-0.28	-0.15	0.59	-1.17	0.23	Stable	7.00
OTOS	-0.14	-0.04	0.13	0.08	0.13	0.00	-0.10	-0.41	0.12	-0.07	0.13	0.13	-0.05	-0.06	-0.13	0.13	0.07	-1.37	0.45	Stable	15.00
ENSSSCG00000047475	0.37	0.28	0.37	1.20	1.20	0.38	0.37	0.53	0.37	0.26	1.08	0.94	1.24	0.51	0.18	0.61	0.01	0.43	-1.21	Down	1.00
ENSSSCG00000047493	-0.06	-0.32	-0.14	-0.16	0.01	-0.20	0.17	0.11	-0.27	-0.42	0.19	-0.14	0.13	-0.31	-0.64	0.17	0.29	3.52	1.81	Stable	3.00
ENSSSCG00000047560	-0.56	-0.47	-0.56	-0.14	-0.27	-0.26	-0.38	-0.19	-0.29	-0.28	-0.29	-0.19	-0.07	-0.35	-0.56	0.13	0.00	2.47	1.30	Up	1.00
ENSSSCG00000047595	-0.05	-0.05	-0.26	-0.41	-0.67	-0.26	-0.41	-0.41	-0.05	-0.08	-0.41	0.02	-0.41	-0.67	0.02	-0.34	0.48	0.74	-0.43	Stable	14.00
ENSSSCG00000047749	-0.74	0.02	-0.49	-0.43	-0.18	0.25	-0.49	-0.25	-0.64	-0.77	-0.44	0.54	-0.53	0.13	0.25	-0.83	0.93	0.93	-0.10	Stable	9.00
ENSSSCG00000047850	1.16	1.32	0.86	0.92	0.89	1.43	1.37	0.90	0.87	0.95	1.30	1.30	0.81	1.23	1.22	1.83	0.66	0.95	-0.08	Stable	5.00
ENSSSCG00000047958	-2.92	-0.91	-1.54	-1.62	-2.45	-2.90	-1.90	-1.49	-1.79	-2.58	-1.72	-1.33	-1.19	-1.39	-1.91	-2.44	0.35	0.85	-0.23	Stable	13.00
ENSSSCG00000048081	-0.13	-0.09	-0.03	-0.13	-0.13	0.28	0.04	0.11	0.08	0.04	-0.06	-0.09	0.11	-0.03	-0.09	0.05	0.65	-2.25	1.17	Stable	8.00
ENSSSCG00000048509	-0.23	-0.27	-0.29	-0.05	-0.05	-0.36	-0.18	-0.29	-0.05	-0.31	-0.22	-0.13	-0.27	-0.23	-0.25	-0.16	0.57	1.15	0.21	Stable	18.00
ENSSSCG00000048729	0.25	0.25	0.33	-0.16	0.13	0.26	-0.34	-0.42	-0.15	-0.50	0.23	-0.41	-0.16	-0.09	-0.12	-0.06	0.74	0.42	-1.26	Stable	2.00
ENSSSCG00000048802	-0.18	0.25	-0.07	0.19	0.22	0.14	-0.04	0.00	0.13	-0.07	0.16	0.13	0.22	-0.18	0.31	0.27	0.11	0.18	-2.45	Stable	7.00
ENSSSCG00000048944	0.02	0.10	0.07	-0.17	0.10	0.10	-0.02	0.10	-0.09	0.03	-0.13	0.10	-0.33	0.10	0.06	0.00	0.23	-1.21	0.27	Stable	2.00
ENSSSCG00000049039	0.22	0.24	0.14	0.52	-0.01	-0.18	0.22	-0.07	-0.13	0.23	0.32	-0.11	0.06	-0.08	0.03	0.13	0.61	0.58	-0.78	Stable	1.00
ENSSSCG00000049278	0.88	0.63	0.71	0.53	0.61	0.36	0.88	0.61	0.51	0.49	0.56	0.75	0.53	0.88	0.88	0.61	0.01	1.36	0.44	Stable	13.00
ENSSSCG00000049444	-0.16	-0.51	-0.31	-0.14	-0.05	0.25	-0.05	0.02	0.10	-0.19	-0.05	-0.05	-0.05	-0.43	-0.34	-0.02	0.08	5.53	2.47	Stable	4.00
ENSSSCG00000049515	-0.62	0.02	-0.13	-0.15	-0.62	-0.03	-0.62	-0.72	-0.36	-0.30	-0.38	0.12	-0.62	-0.51	-0.20	-0.18	0.38	1.45	0.54	Stable	12.00
ENSSSCG00000049525	0.00	0.08	0.00	0.00	0.00	0.00	0.00	0.00	0.00	0.00	0.00	0.00	0.00	0.00	0.00	0.00	0.35	Inf	Inf	Stable	13.00
ENSSSCG00000049578	0.10	0.36	0.31	0.09	0.09	0.36	0.06	0.36	0.12	0.27	-0.18	-0.18	-0.18	0.09	0.36	-0.18	0.05	15.20	3.93	Up	4.00
ENSSSCG00000049711	-0.14	0.06	0.26	0.22	0.27	0.03	0.09	0.49	0.59	0.04	0.22	-0.09	0.05	0.61	-0.11	0.27	0.39	1.84	0.88	Stable	17.00
ENSSSCG00000049725	-0.29	-0.57	-0.22	-0.18	-0.34	-0.37	-0.37	-0.57	-0.32	-0.49	-0.16	-0.37	-0.17	-0.43	-0.30	-0.32	0.20	1.28	0.36	Stable	5.00
ENSSSCG00000049885	-0.28	-0.13	-0.21	0.06	-0.36	-0.59	-0.05	-0.55	-0.26	-0.55	-0.06	-0.32	-0.13	-0.28	-0.26	-0.36	0.71	0.87	-0.20	Stable	4.00
TCF24	0.11	0.27	0.19	0.16	0.11	0.28	0.06	-0.07	0.32	0.19	-0.01	0.06	0.11	0.19	0.11	0.11	0.71	1.16	0.21	Stable	4.00
CCDC201	-0.46	-0.10	-0.48	0.00	-0.46	-0.50	-0.39	-0.08	-0.47	0.02	0.00	-0.17	-0.89	-0.08	-0.23	-0.57	0.80	0.89	-0.17	Stable	18.00
ENSSSCG00000050097	-0.02	-0.06	-0.42	-0.35	-0.29	-0.58	0.00	-0.32	-0.29	-0.12	-0.35	-0.29	-0.12	0.13	-0.27	-0.35	0.09	0.50	-1.00	Stable	15.00
ENSSSCG00000050121	-1.31	-1.49	-0.32	-1.37	-1.53	-1.51	-1.49	0.21	0.41	-1.24	-0.52	-1.10	-1.33	-1.28	-1.27	-1.53	0.17	0.65	-0.63	Stable	14.00
ENSSSCG00000050264	0.05	0.13	0.15	0.14	0.22	0.43	0.09	0.25	0.11	0.13	0.14	0.08	0.08	0.07	0.14	0.14	0.35	0.73	-0.45	Stable	9.00
PSORS1C2	0.03	-0.24	-0.37	0.08	0.03	-0.13	-0.32	-0.07	-0.24	-0.15	-0.07	-0.04	0.09	0.21	0.03	0.13	0.19	16.55	4.05	Stable	7.00
ENSSSCG00000050436	-0.57	0.06	-0.12	-0.73	-0.67	-0.73	-0.59	0.00	0.06	-0.29	-0.63	-0.59	-0.47	-0.27	-0.59	-0.30	0.03	0.46	-1.12	Down	6.00
ENSSSCG00000050469	0.05	-0.13	-0.38	-0.24	-0.08	-0.24	-0.08	0.01	0.07	-0.27	-0.24	0.18	-0.24	-0.13	0.11	-0.24	0.18	0.35	-1.50	Stable	1.00
OR6K3	0.00	-0.16	-0.03	-0.13	-0.26	-0.34	-0.13	-0.60	-0.13	-0.26	-0.26	-0.13	-0.26	-0.44	-0.13	-0.26	0.68	0.86	-0.22	Stable	4.00
ENSSSCG00000050791	0.02	-0.59	-0.24	-0.22	-0.26	-0.56	-0.07	-0.18	-0.18	0.01	-0.08	-0.22	-0.27	0.00	0.01	0.00	0.65	0.78	-0.36	Stable	9.00
ENSSSCG00000050878	0.35	-0.41	0.09	-0.16	0.28	-0.20	0.50	-0.20	-0.41	-0.20	0.01	0.50	-0.02	-0.17	0.50	0.25	0.88	0.57	-0.82	Stable	6.00
TOMM6	-0.41	-1.17	-0.87	-0.72	-0.49	-1.02	-1.14	-1.30	-0.70	-0.49	-0.58	-0.88	-0.95	-0.39	-0.65	-0.88	0.60	1.10	0.14	Stable	7.00
ENSSSCG00000050958	0.02	-0.15	0.08	-0.09	-0.15	-0.37	-0.06	0.11	0.35	-0.33	-0.12	-0.06	-0.21	0.19	-0.06	0.08	0.01	-0.41	-1.30	Down	9.00
ENSSSCG00000051006	0.15	0.86	0.31	0.36	0.18	0.36	0.15	0.32	0.85	0.58	0.13	0.99	0.18	0.53	0.69	0.22	0.47	1.29	0.37	Stable	5.00
ENSSSCG00000051032	0.48	0.17	0.17	0.31	0.24	0.35	0.03	0.32	0.46	0.29	0.46	0.33	0.46	0.17	0.24	0.46	0.12	0.70	-0.51	Stable	4.00

P2RX4	0.37	0.82	0.75	0.77	0.78	0.63	0.37	0.42	0.21	0.05	1.00	0.27	0.71	0.32	0.82	0.61	0.50	0.84	-0.25	Stable	14.00
ENSSSCG00000051213	0.00	0.00	0.00	0.00	0.00	0.33	0.01	0.00	0.01	0.00	0.00	0.01	0.00	0.02	0.00	0.00	0.40	0.07	-3.86	Stable	9.00
ENSSSCG00000051243	-0.53	-0.49	-0.33	-0.25	-0.12	0.02	-0.53	-0.20	0.03	0.14	0.02	-0.53	-0.23	-0.53	-0.53	-0.14	0.03	2.85	1.51	Up	16.00
SYCP2L	0.04	-0.03	-0.22	-0.05	0.15	0.12	0.04	0.10	-0.42	-0.27	-0.05	-0.22	-0.07	0.07	0.28	-0.03	0.69	0.31	-1.68	Stable	7.00
ENSSSCG00000051561	-0.42	-0.45	-0.42	-0.42	-0.48	-0.42	-0.28	-0.21	-0.42	-0.42	-0.42	-0.42	-0.48	-0.42	-0.45	-0.42	0.16	0.88	-0.18	Stable	7.00
ENSSSCG00000051671	-0.18	0.15	0.15	-0.06	0.00	0.01	-0.13	-0.07	0.02	0.13	0.00	0.00	0.00	0.00	0.00	0.00	0.73	-0.65	-0.63	Stable	13.00
CHRNB1	0.38	-0.29	-0.23	-0.03	-0.19	0.32	-0.02	0.27	0.28	0.56	-0.41	-0.10	0.25	-0.23	0.06	0.00	0.88	0.54	-0.88	Stable	12.00

Table S9: Strong correlation between predicted genes and *Treponema*.

Var1	Significant	Var2	cor	p_value
PSMB2	Stable	Treponema	-0.83604957	5.47E-05
MRPS15	Stable	Treponema	-0.80353206	0.000177059
CALR3	Stable	Treponema	-0.78734385	0.000294261
ZFP69B	Stable	Treponema	0.75791043	0.000669784
STK40	Stable	Treponema	-0.75395023	0.000741761
CDCP2	Stable	Treponema	-0.71281373	0.001940064
RGP1	Down	Treponema	-0.70986822	0.002065604
GNA13	Stable	Treponema	-0.70493029	0.002290799
C9orf78	Stable	Treponema	-0.7	0.002535095
ALDH1B1	Stable	Treponema	-0.69411765	0.002853706
ANKRD34B	Stable	Treponema	0.67844021	0.003863343
MEST	Stable	Treponema	-0.66764706	0.004711982
VAPA	Stable	Treponema	-0.66568555	0.004881113
AGPS	Stable	Treponema	0.66026632	0.005373779
LTA	Stable	Treponema	0.65828727	0.005563295
SEC61A1	Stable	Treponema	0.65636515	0.005752432
ENSSSCG00000049578	Up	Treponema	-0.65579388	0.005809624
SPIDR	Stable	Treponema	0.64801249	0.006634838
MRPL37	Stable	Treponema	-0.64705882	0.006742077
CEP89	Stable	Treponema	-0.64649887	0.006805684
RGS22	Down	Treponema	0.64533703	0.006939183
PARP12	Stable	Treponema	-0.64259553	0.007262452
F2R	Stable	Treponema	0.64254693	0.007268289
GFUS	Stable	Treponema	-0.64117647	0.007434419
PTPRB	Stable	Treponema	0.64017677	0.007557496
BAALC	Down	Treponema	0.63566357	0.008133422
ENSSSCG00000025565	Up	Treponema	0.63469067	0.008262012
NUDT19	Stable	Treponema	0.62832132	0.009144172
ENSSSCG00000048729	Stable	Treponema	-0.62546007	0.009563926
LRMDA	Stable	Treponema	0.62490941	0.009646426
NKAIN1	Stable	Treponema	0.62444839	0.009715928
ATP6V0A4	Stable	Treponema	-0.62242159	0.010026187
C3	Stable	Treponema	-0.62137378	0.010189618
ORC1	Stable	Treponema	0.61912131	0.010548058
ENSSSCG00000047493	Stable	Treponema	0.61764706	0.010787985
NRP1	Stable	Treponema	-0.61733672	0.010839034
FCGRT	Stable	Treponema	0.61652186	0.01097398
POGLUT2	Stable	Treponema	0.60382982	0.013251771
CPNE5	Stable	Treponema	0.60294118	0.013424125
ZFAND1	Stable	Treponema	0.60191333	0.013625662
FAM172A	Stable	Treponema	-0.6015864	0.01369026
KLHL42	Stable	Treponema	0.6002976	0.013947249

Table S10: Functional enrichment annotation for Fst and RNA using GO and KEGG.

	ID	GeneRatio	Description	pvale	Type
Fst	ssc04976	0.084745763	Bile secretion	0.014074626	KEGG pathway
	ssc04120	0.061403509	Ubiquitin mediated proteolysis	0.018925764	KEGG pathway
	ssc02010	0.076923077	ABC transporters	0.036272463	KEGG pathway
	ssc04975	0.1	Fat digestion and absorption	0.036825721	KEGG pathway
	GO:0042407	0.333333333	cristae formation	0.002095247	GO term
	GO:0030676	0.25	Rac guanyl-nucleotide exchange factor activity	0.004123204	GO term
	GO:0050684	0.25	regulation of mRNA processing	0.004123204	GO term
	GO:0006979	0.108695652	response to oxidative stress	0.005479746	GO term
	GO:0016361	0.4	activin receptor activity, type I	0.009709993	GO term
	GO:0042753	0.4	positive regulation of circadian rhythm	0.009709993	GO term
RNA	ssc01100	0.032176122	Metabolic pathways	0.000314336	KEGG pathway
	ssc04022	0.058823529	cGMP-PKG signaling pathway	0.003740854	KEGG pathway
	ssc00230	0.065420561	Purine metabolism	0.003798127	KEGG pathway
	ssc04146	0.074074074	Peroxisome	0.004103872	KEGG pathway
	ssc00500	0.1	Starch and sucrose metabolism	0.019394919	KEGG pathway
	ssc04020	0.04516129	Calcium signaling pathway	0.023452233	KEGG pathway
	ssc04744	0.117647059	Phototransduction	0.04253867	KEGG pathway
	ssc00564	0.054054054	Glycerophospholipid metabolism	0.047163233	KEGG pathway
	ssc00330	0.068181818	Arginine and proline metabolism	0.048329545	KEGG pathway
	GO:0055114	0.046448087	oxidation-reduction process	0.000406086	GO term
	GO:0061003	0.4	positive regulation of dendritic spine morphogenesis	0.005991087	GO term
	GO:0007178	0.4	brane receptor protein serine/threonine kinase signalin	0.005991087	GO term
	GO:1902494	0.4	catalytic complex	0.005991087	GO term
	GO:0097755	0.333333333	positive regulation of blood vessel diameter	0.007896709	GO term
	GO:0005102	0.056451613	signaling receptor binding	0.00804259	GO term

Table S11: NCBI SRA Accession column for PRJNA747893.

Sample ID	sample name	Biosample	SRA	NCBI Sequence Read Archive
Sample 1	LW-F-1	SAMN20299484	SRS9867244	https://identifiers.org/ncbi/insdc.sra:SRS9867244
Sample 2	LW-F-2	SAMN20299485	SRS9867245	https://identifiers.org/ncbi/insdc.sra:SRS9867245
Sample 3	LW-F-3	SAMN20299486	SRS9867252	https://identifiers.org/ncbi/insdc.sra:SRS9867252
Sample 4	LW-F-4	SAMN20299487	SRS9867253	https://identifiers.org/ncbi/insdc.sra:SRS9867253
Sample 5	LW-F-5	SAMN20299488	SRS9867254	https://identifiers.org/ncbi/insdc.sra:SRS9867254
Sample 6	LW-F-6	SAMN20299489	SRS9867255	https://identifiers.org/ncbi/insdc.sra:SRS9867255
Sample 7	LW-F-7	SAMN20299490	SRS9867256	https://identifiers.org/ncbi/insdc.sra:SRS9867256
Sample 8	LW-F-8	SAMN20299491	SRS9867257	https://identifiers.org/ncbi/insdc.sra:SRS9867257
Sample 9	LL-F-1	SAMN20299492	SRS9867258	https://identifiers.org/ncbi/insdc.sra:SRS9867258
Sample 10	LL-F-2	SAMN20299493	SRS9867259	https://identifiers.org/ncbi/insdc.sra:SRS9867259
Sample 11	LL-F-3	SAMN20299494	SRS9867246	https://identifiers.org/ncbi/insdc.sra:SRS9867246
Sample 12	LL-F-4	SAMN20299495	SRS9867247	https://identifiers.org/ncbi/insdc.sra:SRS9867247
Sample 13	LL-F-5	SAMN20299496	SRS9867248	https://identifiers.org/ncbi/insdc.sra:SRS9867248
Sample 14	LL-F-6	SAMN20299497	SRS9867249	https://identifiers.org/ncbi/insdc.sra:SRS9867249
Sample 15	LL-F-7	SAMN20299498	SRS9867250	https://identifiers.org/ncbi/insdc.sra:SRS9867250
Sample 16	LL-F-8	SAMN20299499	SRS9867251	https://identifiers.org/ncbi/insdc.sra:SRS9867251

Table S12: NCBI SRA Accession column for PRJNA749115.

Sample ID	sample name	Biosample	SRA	NCBI Sequence Read Archive
Sample 1	LW-1	SAMN20353412	SRS9562900	https://identifiers.org/ncbi/insdc.sra:SRS9562900
Sample 2	LW-2	SAMN20353413	SRS9562901	https://identifiers.org/ncbi/insdc.sra:SRS9562901
Sample 3	LW-3	SAMN20353414	SRS9562908	https://identifiers.org/ncbi/insdc.sra:SRS9562908
Sample 4	LW-4	SAMN20353415	SRS9562909	https://identifiers.org/ncbi/insdc.sra:SRS9562909
Sample 5	LW-5	SAMN20353416	SRS9562910	https://identifiers.org/ncbi/insdc.sra:SRS9562910
Sample 6	LW-6	SAMN20353417	SRS9562911	https://identifiers.org/ncbi/insdc.sra:SRS9562911
Sample 7	LW-7	SAMN20353418	SRS9562912	https://identifiers.org/ncbi/insdc.sra:SRS9562912
Sample 8	LW-8	SAMN20353419	SRS9562913	https://identifiers.org/ncbi/insdc.sra:SRS9562913
Sample 9	LL-1	SAMN20353420	SRS9562914	https://identifiers.org/ncbi/insdc.sra:SRS9562914
Sample 10	LL-2	SAMN20353421	SRS9562915	https://identifiers.org/ncbi/insdc.sra:SRS9562915
Sample 11	LL-3	SAMN20353422	SRS9562902	https://identifiers.org/ncbi/insdc.sra:SRS9562902
Sample 12	LL-4	SAMN20353423	SRS9562903	https://identifiers.org/ncbi/insdc.sra:SRS9562903
Sample 13	LL-5	SAMN20353424	SRS9562904	https://identifiers.org/ncbi/insdc.sra:SRS9562904
Sample 14	LL-6	SAMN20353425	SRS9562905	https://identifiers.org/ncbi/insdc.sra:SRS9562905
Sample 15	LL-7	SAMN20353426	SRS9562906	https://identifiers.org/ncbi/insdc.sra:SRS9562906
Sample 16	LL-8	SAMN20353427	SRS9562907	https://identifiers.org/ncbi/insdc.sra:SRS9562907
